# Supplementary material for: Valence-isomer selective cycloaddition reaction of cycloheptatrienes-norcaradienes
Source: Nat Commun. 2024 Mar 14;15:2309. doi: 10.1038/s41467-024-46523-1 (PMC10940685; doi:10.1038/s41467-024-46523-1)
Supplement: Supplementary file 1 — Supplementary Information [file 41467_2024_46523_MOESM1_ESM.pdf]

Supplementary Information

Valence-isomer selective cycloaddition reaction of cycloheptatrienes-norcaradienes

Shingo Harada[1]*, Hiroki Takenaka[1,†], Tsubasa Ito[1,†], Haruki Kanda[1], and Tetsuhiro Nemoto[1]*

[†]These authors contributed equally to this work.

[1] Graduate School of Pharmaceutical Sciences, Chiba University, 1-8-1, Inohana, Chuo-ku, Chiba 260-8675 (Japan)

Tel/Fax:81-43-226-2920.

E-mail: Sharada@chiba-u.jp, tnemoto@faculty.chiba-u.jp

1. General Information..S3
2. Additional Data..S4
3. Synthesis and Characterization of Cycloadducts with Nitroso Compounds (*Conditions A*)..S25
4. Synthesis and Characterization of Cycloadducts with Nitroso Compounds (*Conditions C*)..S48
5. Synthesis and Characterization of Cycloadducts with Aryne (*Conditions B*)..S65
6. Synthesis and Characterization of Cycloadducts with Arynes (*Conditions D*)..S92
7. Characterization of Derivatives...S110
8. Single Crystal X-Ray Diffraction Analysis..S122
9. Synthesis and Characterization of Substrates.....................................S137
10. Computational Details (Mechanistic Investigation)...........................S161
11. Computational Details (Training Data)..S164
12. Charts of ^{1}H- and ^{13}C-NMR Spectra..S189
13. Copy of HPLC Spectra...S366
14. References..S416

1. General Information

NMR spectra were recorded at 400 or 600 MHz for ^{1}H NMR, 100 or 150 MHz for ^{13}C NMR, and 376 MHz or 564 MHz for ^{19}F NMR. Chemical shifts in $CDCl_3$ or CD_3OD, were reported downfield from TMS (= 0 ppm) or solvent signals [CD_3OD (3.31 ppm)] for ^{1}H NMR. Data are reported as follows: chemical shift, multiplicity (s = singlet, d = doublet, t = triplet, m = multiplet, and br = broad), integration and coupling constants in Hz. For ^{13}C NMR, chemical shifts were reported in the scale relative to the solvent signal [$CHCl_3$ (77.00 ppm) and CH_3OH (49.00 ppm)] as an internal reference. ESI mass spectra were measured on JEOL AccuTOF LC-plus JMS-T100LP. Optical rotations were measured on a JASCO P-1020 polarimeter. The enantiomeric ratio (er) was determined by HPLC analysis. HPLC was performed on JASCO HPLC systems consisting of the following: pump, PU-980; detector, UV-970; column DAICEL CHIRALPAK IH-3, CHIRALPAK IBN-3; mobile phase, *n*-hexane/DCM. Melting points were measured with a SIBATA NEL-270 melting point apparatus. The UV-Vis spectrometer was a JASCO V-730 spectrometer. Analytical thin layer chromatography was performed on Kieselgel 60F254, 0.25 mm-thick plates. Column chromatography was performed with silica gel 60 N (spherical, neutral 63-210 mesh). Reactions were conducted in dry solvent. Other reagents were purified by the usual methods.

2. [Additional Data]

2-1 Mechanistic Studies

Non-covalent interaction was visualized using Visual Molecular Dynamics (VMD) software (ver. 1.9.4a57). The source files were created from the corresponding XYZ files using NCIPLOT (ver. 4.0)[1]. The program option “multigrid level: ultrafine” was selected, and default values were used for other parameters (Density (r1) 0.05 and reduced density gradient (RDG, r2) 0.5 cutoffs). All the plots of RDG vs sign(λ_2)ρ was created using GNUPLOT (ver. 5.4.3).

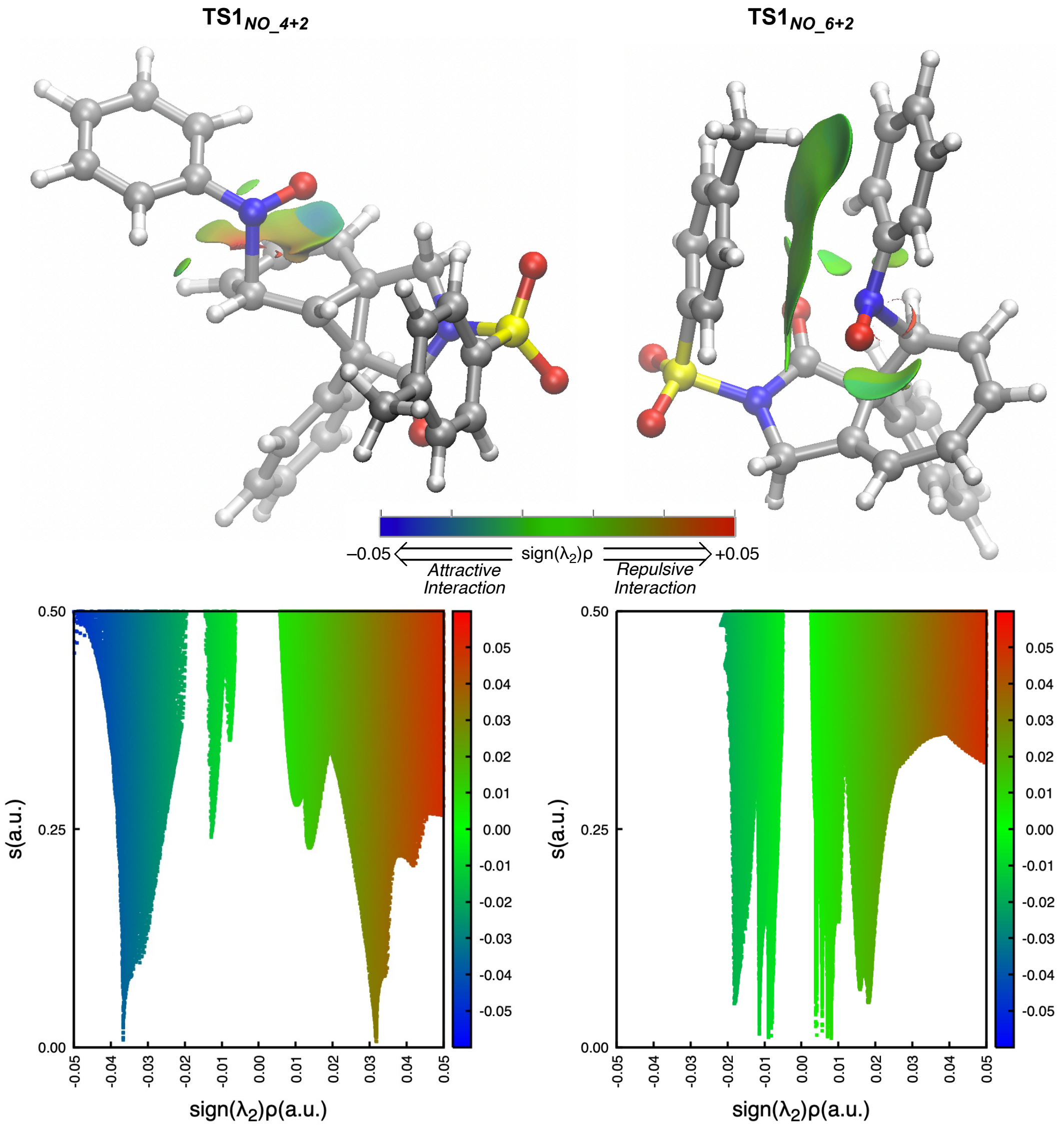

Supplementary Fig. 1 | NCIPLOT of transition states for cycloaddition steps with nitroso compounds and plots of RDG vs sign(λ_2)ρ.

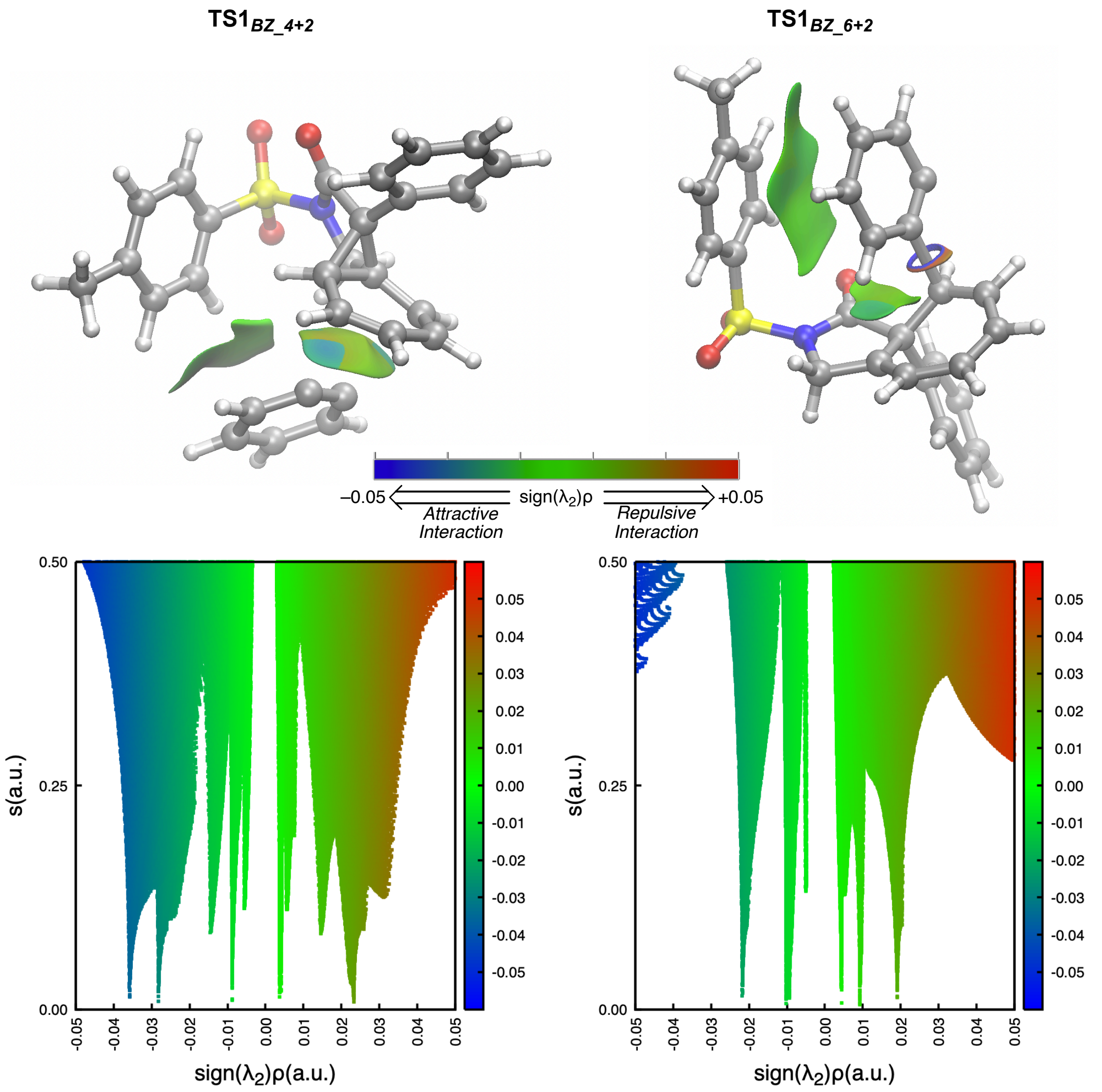

Supplementary Fig. 2 | NCIPLOT of transition states for cycloaddition steps with arynes and plots of RDG vs sign(λ_2)ρ.

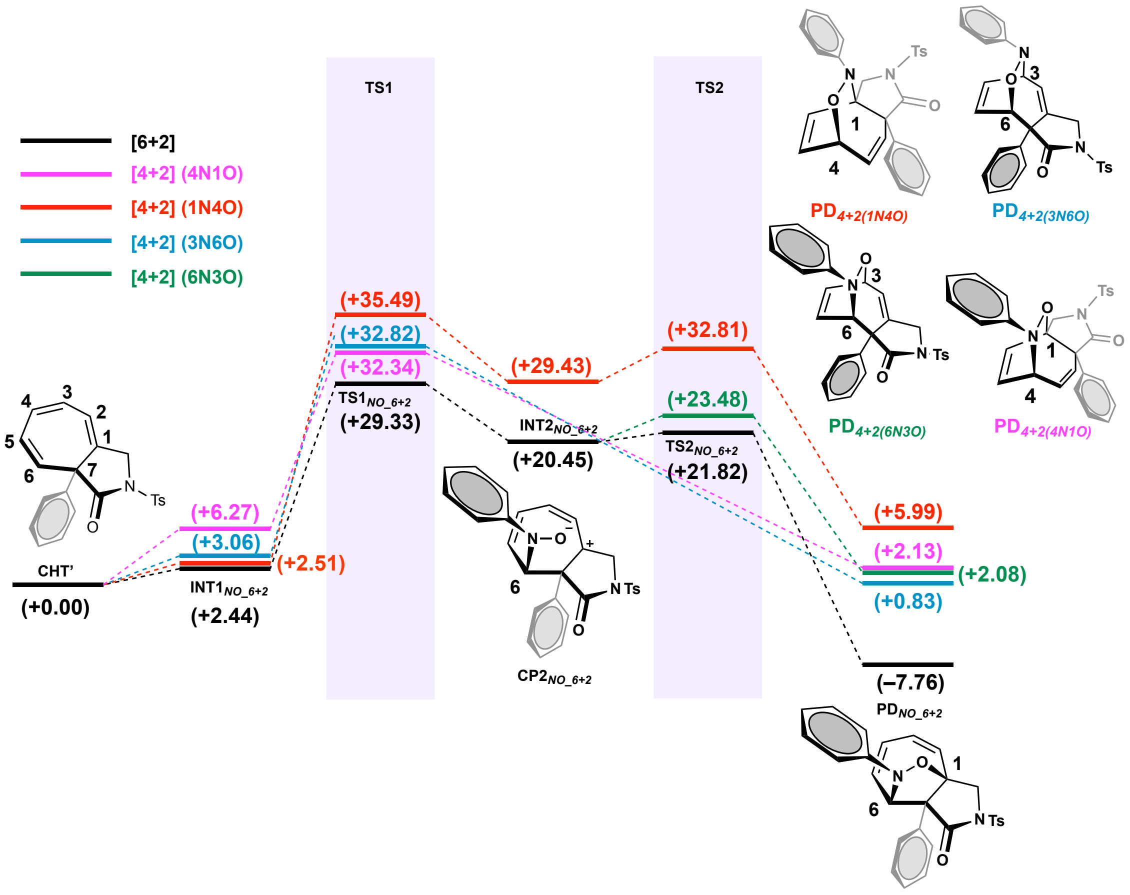

Supplementary Fig. 3 | Gibbs free-energy profile for the reaction process to produce various isomers. Computation was carried out based on RωB97X-D/6-311+G**//RωB97X-D/6-31G* in chlorobenzene solvent.

2-2 Optimization of Machine Learning Model

The specific bond orbital data were obtained using the following steps.

1) Density functional theory calculations were performed with Gaussian 16 program to optimize the molecular structure and generate comprehensive bond orbital information. The vibrational frequencies were computed to check whether each optimized structure is at an energy minimum on the potential energy surfaces (no imaginary frequency).

2) By using "formchk" command in a terminal, checkpoint files (.chk) were converted into Gaussian formatted checkpoint file format (.fchk) with the program.

3) The obtained FCHK files were opened in Avogadro software. https://avogadro.cc/install/

4) When the obtained FCHK files are opened, Avogadro automatically opens the orbitals toolbar containing all potential molecular orbitals.

5) By visualizing the orbitals, we identified the occupied orbital at the highest energy level of the functional groups of interest (HOBO), rather than the entire molecule. Similarly, the unoccupied orbital at the lowest energy level of the functional groups of interest was also identified (LUBO), and the energy level information was obtained.

6) Molecular structures were drawn in ChemDraw and converted to molecular fingerprint *via* SMILES. The fingerprint was used as the independent variable and HOBO/LUBO was used as the dependent variable.

7) (1)-(6) were repeated to obtain a dataset containing about 500 molecules.

8) The dataset was divided into training data, validation data, and test data. By using the training data and the validation data, regression models were constructed and the hyperparameters were adjusted.

9) Algorithm combinations of fingerprints and supervised machine learnings were examined using the test data. As a result, the combination of a three hidden-layer Neural Network, and a 4096-bit Avalon fingerprint yielded a high coefficient of determination. (Approximately 650,000 predictive models were examined.)

10) The accuracy of the bond orbital data was validated by other data not included in training dataset.

Model selection plays a crucial role in training machine learning models. We used 3-fold cross-validation to test the performance of candidate machine learning algorithms, and the mean absolute error (MAE) and the coefficient of determination (R^2) to examine the regression performance of these models. The following six algorithms were selected as candidates. Five regression models commonly used in the Scikit-learn package: Elastic Nets (EN), Decision Trees (DT), Random Forests (RF), Support Vector Machines (SVM) and Neural Network (NN), and Light Gradient Boosting Machine Regression model

included in the Light Gradient Boosting Machine (LGBM) package. The workflow of hyperparameter tuning for each regression model is shown in Supplementary Fig. 4.

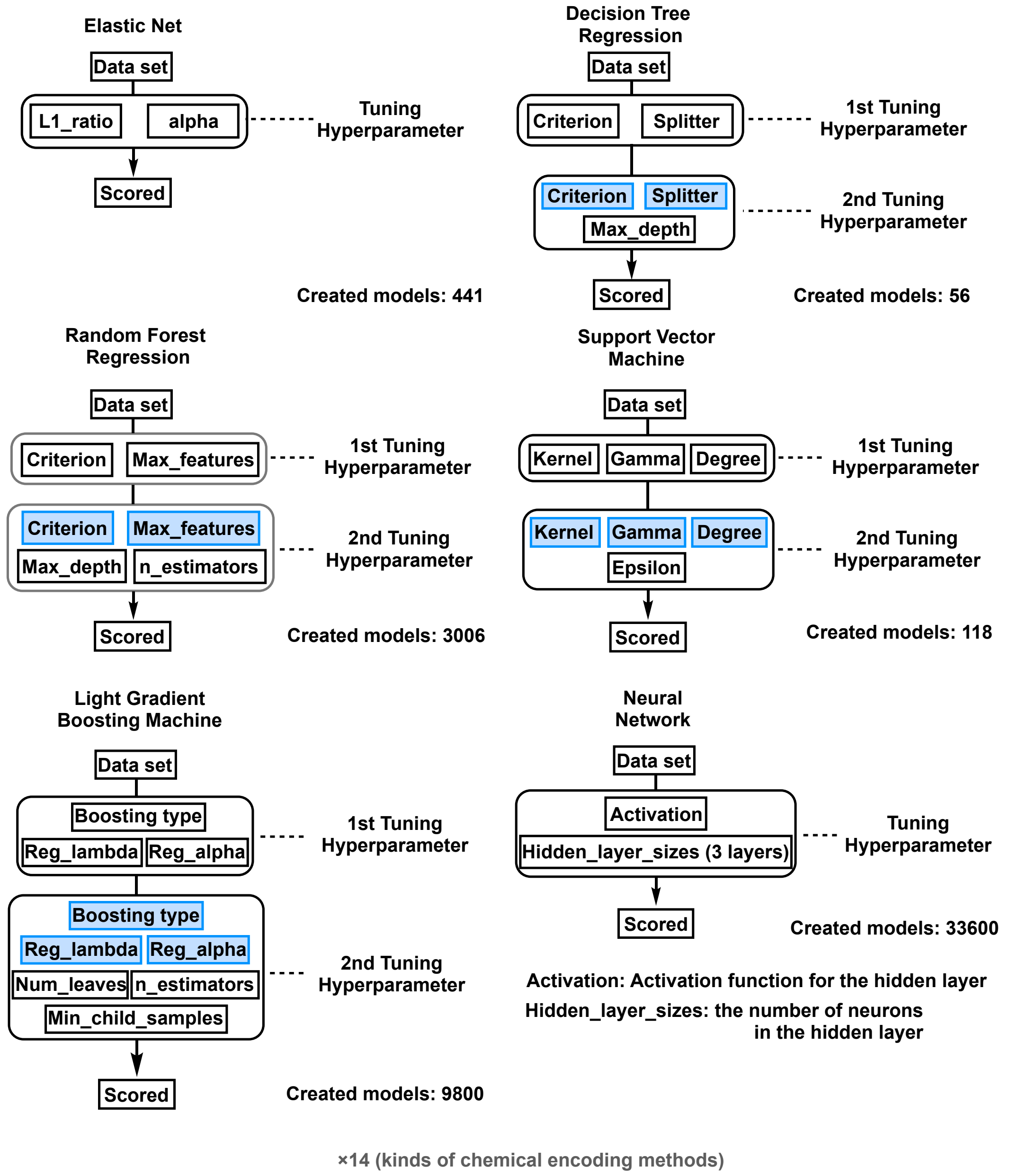

Supplementary Fig. 4 | The workflow of hyperparameter tuning for each regression models.

Coefficient of Determination (R^2)														
Enophile	Avalon 1024	Avalon 2048	Avalon 4096	Morgan 1024 r = 2	Morgan 2048 r = 2	Morgan 4096 r = 2	Morgan 1024 r = 3	Morgan 2048 r = 3	Morgan 4096 r = 3	Morgan 1024 r = 4	Morgan 2048 r = 4	Morgan 4096 r = 4	MACCS	Topo logical
Elastic Net	0.779	0.797	0.803	0.763	0.763	0.769	0.743	0.757	0.766	0.742	0.756	0.766	0.763	0.359
DTR	0.634	0.683	0.606	0.634	0.647	0.663	0.589	0.626	0.665	0.539	0.618	0.675	0.704	0.112
RFR	0.759	0.751	0.714	0.705	0.712	0.713	0.689	0.696	0.713	0.681	0.690	0.713	0.772	0.401
SVM	0.757	0.776	0.780	0.720	0.729	0.731	0.692	0.715	0.724	0.690	0.713	0.725	0.757	0.399
LGBM	0.778	**0.828**	0.785	0.772	0.776	0.770	0.761	0.758	0.766	0.754	0.757	0.768	0.808	0.432
NNET	**0.821**	**0.835**	**0.829**	0.784	0.791	0.792	0.769	0.782	0.792	0.764	0.779	0.792	**0.818**	0.436

Supplementary Fig. 5 | R^2 value in the preliminary study of the machine learning models for predicting the LUBO of enophiles.

MAE														
Enophile	Avalon 1024	Avalon 2048	Avalon 4096	Morgan r = 2 1024	Morgan r = 2 2048	Morgan r = 2 4096	Morgan r = 3 1024	Morgan r = 3 2048	Morgan r = 3 4096	Morgan r = 4 1024	Morgan r = 4 2048	Morgan r = 4 4096	MACCS	Topo logical
Elastic Net	0.483	0.456	0.449	0.504	0.503	0.498	0.527	0.517	0.505	0.530	0.516	0.504	0.517	0.831
DTR	0.628	0.535	0.627	0.612	0.598	0.581	0.632	0.609	0.587	0.671	0.617	0.562	0.510	1.023
RFR	0.493	0.500	0.541	0.533	0.528	0.533	0.545	0.541	0.529	0.554	0.546	0.528	0.449	0.804
SVM	0.512	0.490	0.477	0.544	0.532	0.537	0.568	0.553	0.543	0.574	0.554	0.543	0.516	0.819
LGBM	0.457	**0.416**	0.437	0.487	0.482	0.487	0.495	0.512	0.486	0.504	0.499	0.483	0.425	0.745
NNET	**0.419**	**0.404**	**0.420**	0.470	0.467	0.466	0.480	0.469	0.458	0.499	0.471	0.460	**0.411**	0.734

Supplementary Fig. 6 | MAE value in the preliminary study of the machine learning models for predicting the LUBO of enophiles.

Coefficient of Determination (R^2)														
CHT / NCD	Avalon 1024	Avalon 2048	Avalon 4096	Morgan 1024 r = 2	Morgan 2048 r = 2	Morgan 4096 r = 2	Morgan 1024 r = 3	Morgan 2048 r = 3	Morgan 4096 r = 3	Morgan 1024 r = 4	Morgan 2048 r = 4	Morgan 4096 r = 4	MACCS	Topo logical
Elastic Net	*0.827*	*0.813*	***0.851***	*0.775*	*0.813*	*0.805*	*0.747*	*0.760*	*0.776*	*0.646*	*0.712*	*0.770*	*0.713*	*0.772*
DTR	*0.574*	*0.691*	*0.758*	*0.671*	*0.713*	*0.718*	*0.513*	*0.686*	*0.609*	*0.429*	*0.579*	*0.561*	*0.742*	*0.247*
RFR	*0.729*	*0.740*	*0.818*	*0.754*	*0.787*	*0.779*	*0.680*	*0.714*	*0.717*	*0.581*	*0.679*	*0.687*	*0.733*	*0.577*
SVM	*0.819*	*0.811*	*0.828*	*0.745*	*0.753*	*0.761*	*0.665*	*0.647*	*0.666*	*0.544*	*0.565*	*0.589*	*0.776*	*0.697*
LGBM	*0.793*	*0.785*	***0.856***	*0.772*	*0.811*	*0.791*	*0.744*	*0.764*	*0.750*	*0.635*	*0.708*	*0.759*	*0.729*	*0.708*
NNET	***0.873***	***0.859***	***0.872***	*0.804*	*0.794*	*0.809*	*0.726*	*0.705*	*0.725*	*0.609*	*0.628*	*0.648*	*0.720*	*0.797*

Supplementary Fig. 7 | R^2 value in the preliminary study of the machine learning models for predicting the HOBO of cycloheptatrienes and norcaradienes.

MAE														
CHT / NCD	Avalon 1024	Avalon 2048	Avalon 4096	Morgan r = 2 1024	Morgan r = 2 2048	Morgan r = 2 4096	Morgan r = 3 1024	Morgan r = 3 2048	Morgan r = 3 4096	Morgan r = 4 1024	Morgan r = 4 2048	Morgan r = 4 4096	MACCS	Topo logical
Elastic Net	*0.117*	*0.115*	***0.105***	*0.128*	*0.115*	*0.118*	*0.139*	*0.132*	*0.129*	*0.169*	*0.148*	*0.133*	*0.153*	*0.125*
DTR	*0.161*	*0.136*	*0.128*	*0.152*	*0.144*	*0.144*	*0.184*	*0.148*	*0.164*	*0.200*	*0.178*	*0.175*	*0.148*	*0.237*
RFR	*0.141*	*0.133*	*0.121*	*0.139*	*0.130*	*0.132*	*0.159*	*0.147*	*0.147*	*0.182*	*0.163*	*0.157*	*0.148*	*0.182*
SVM	*0.118*	*0.108*	*0.103*	*0.109*	*0.115*	*0.118*	*0.115*	*0.117*	*0.123*	*0.142*	*0.123*	*0.120*	*0.155*	*0.127*
LGBM	*0.127*	*0.120*	***0.100***	*0.132*	*0.121*	*0.127*	*0.140*	*0.131*	*0.134*	*0.172*	*0.145*	*0.136*	*0.148*	*0.150*
NNET	***0.100***	***0.103***	***0.099***	*0.122*	*0.121*	*0.118*	*0.148*	*0.150*	*0.151*	*0.176*	*0.175*	*0.169*	*0.153*	*0.125*

Supplementary Fig. 8 | MAE value in the preliminary study of a machine learning model for predicting the HOBO of cycloheptatrienes and norcaradienes.

The main study used five combinations of fingerprints and regression models that showed the high predictive accuracy in preliminary studies. We performed 30 tests with different random state values that determine the training data and summarized the average prediction accuracy and maximum prediction accuracy in Supplementary Fig. 9.

	Enophile		Average		Best	
	Model	Finger Print	R^2	MAE	R^2	MAE
1st	NNET	Avalon 2048	*0.859*	*0.366*	*0.932*	*0.263*
2nd	NNET	Avalon 4096	*0.844*	*0.381*	*0.932*	*0.274*
3rd	LGBM	Avalon 2048	*0.849*	*0.369*	*0.919*	*0.269*
4th	NNET	Avalon 1024	*0.812*	*0.424*	*0.917*	*0.304*
5th	NNET	MACCS	*0.803*	*0.412*	*0.890*	*0.382*

	CHT/NCD		Average		Best	
	Model	Finger Print	R^2	MAE	R^2	MAE
1st	NNET	Avalon 1024	*0.881*	*0.089*	*0.933*	*0.070*
2nd	NNET	Avalon 4096	*0.903*	*0.081*	*0.946*	*0.071*
3rd	LGBM	Avalon 2048	*0.875*	*0.094*	*0.938*	*0.078*
4th	LGBM	Avalon 4096	*0.885*	*0.085*	*0.948*	*0.067*
5th	Elastic Net	Avalon 4096	*0.884*	*0.085*	*0.939*	*0.076*

Supplementary Fig. 9 | The result of the main study.

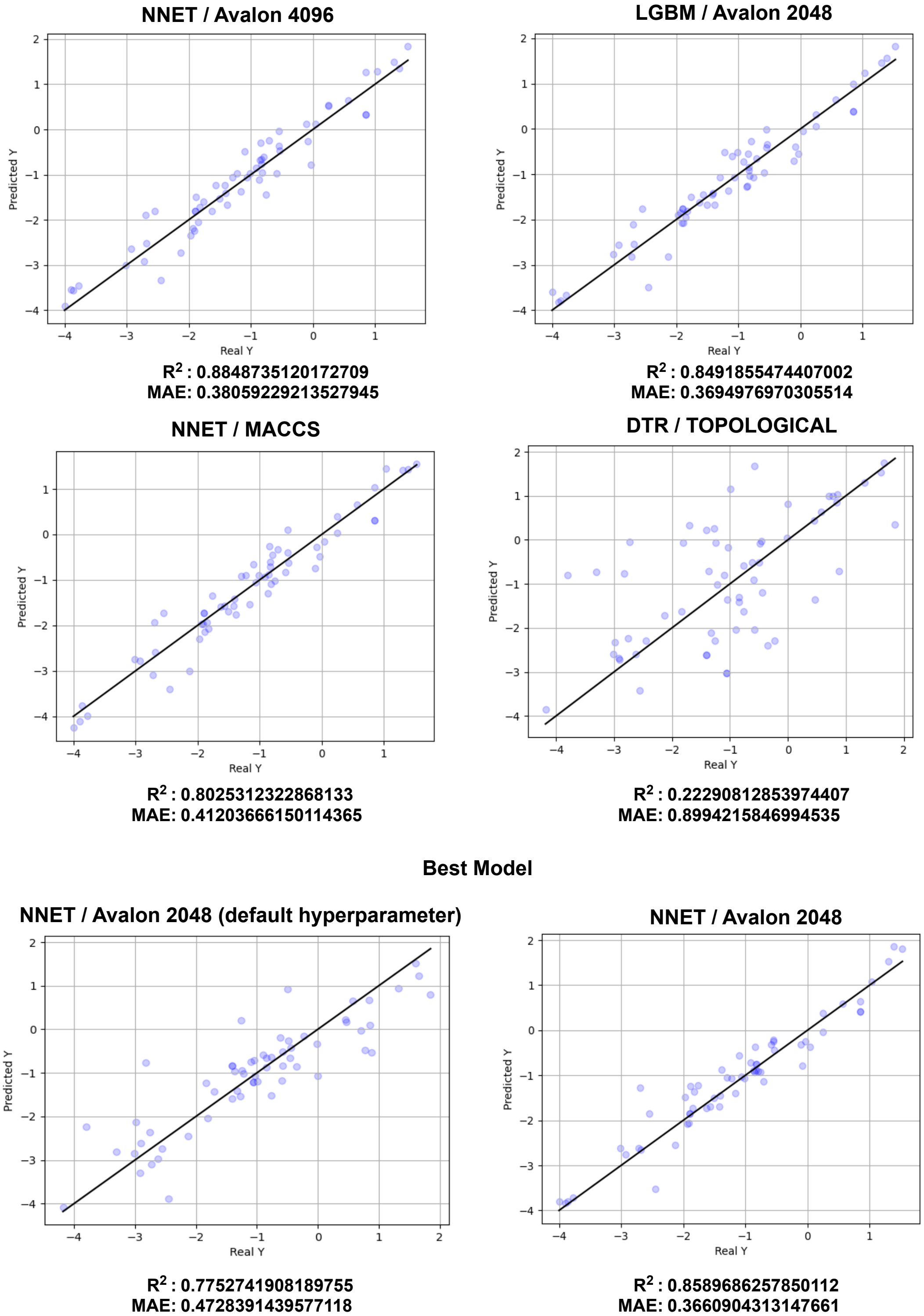

Supplementary Fig. 10 | Correlation plots of enophile's LUBO level.

NNET / Avalon 1024

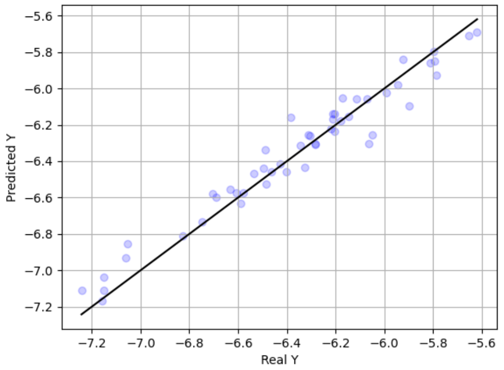

R^2 : 0.8813526777544015
MAE: 0.08929784923407687

Elastic Net / Avalon 4096

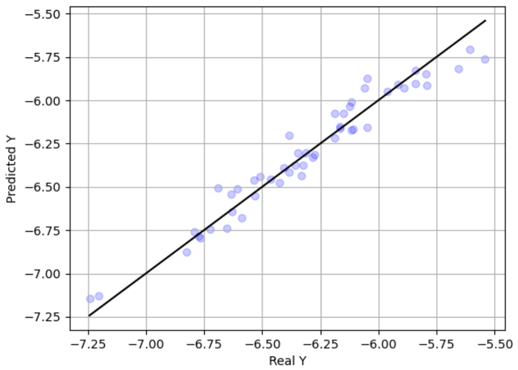

R^2 : 0.8841236731357154
MAE: 0.0848057372387941

LGBM / Avalon 4096

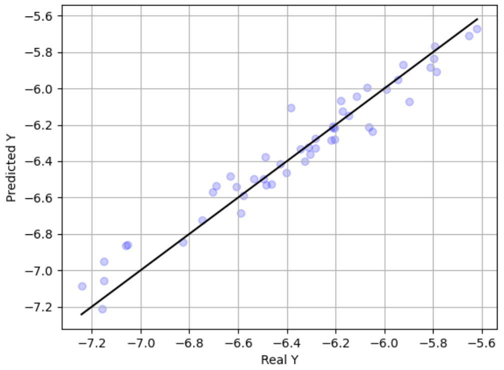

R^2 : 0.8848735120172709
MAE: 0.08480948444182726

RFR / Morgan 1024 (radius 2)

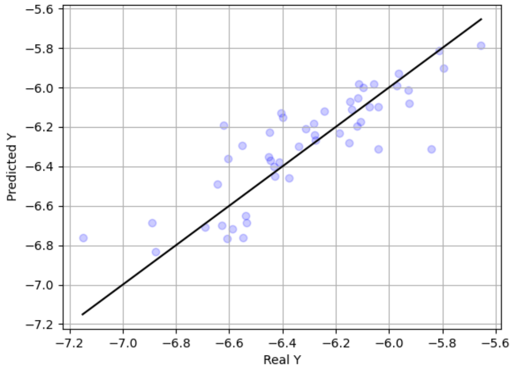

R^2 : 0.712784102854777
MAE: 0.12431314000000021

Best Model

NNET / Avalon 4096 (default hyperparameter)

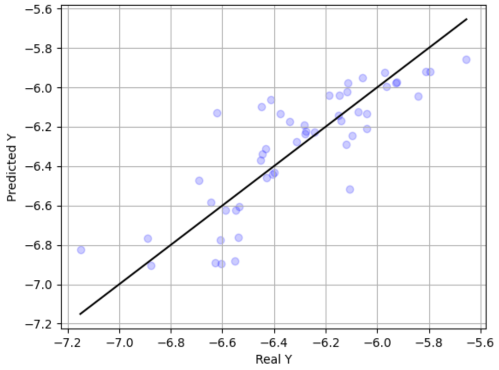

R^2 : 0.6574701172031288
MAE: 0.13881074822895387

NNET / Avalon 4096

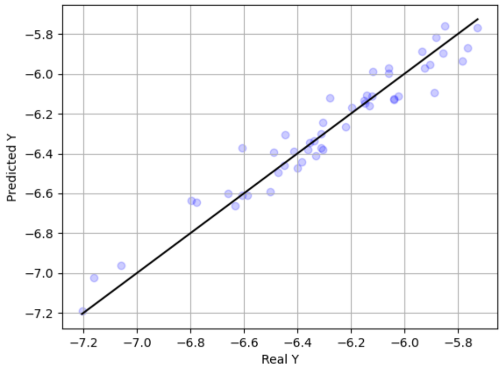

R^2 : 0.9025727030479597
MAE: 0.08136963053696959

Supplementary Fig. 11 | Correlation plots of CHT/NCD's HOBO level.

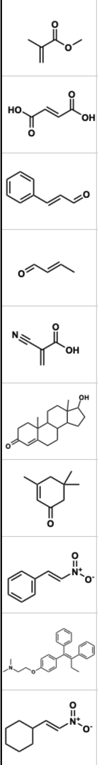
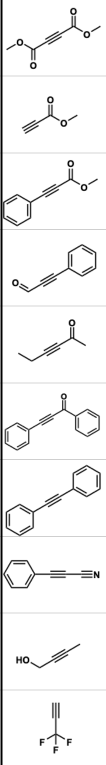
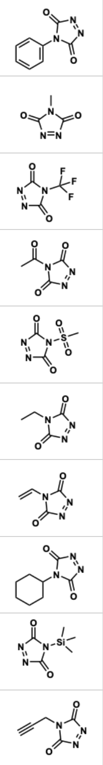

Alkene		HOBO LUBO GAP	Alkyne		HOBO LUBO GAP	Triazoline		HOBO LUBO GAP
	C=C(C)C(OC)=O	5.8941		O=C(C#CC(OC)=O)OC	4.8901		O=C1N(C2=CC=CC=C2)C(N=N1)=O	2.8331
	O=C(O)/C=C/C(O)=O	4.1371		C#CC(OC)=O	5.7161		O=C1N(C)C(N=N1)=O	2.8281
	O=C/C=C/C1=CC=CC=C1	4.2991		O=C(C#CC1=CC=CC=C1)OC	4.1091		O=C1N(C(F)(F)F)C(N=N1)=O	1.6921
	O=C/C=C/C	4.6991		O=CC#CC1=CC=CC=C1	4.3721		O=C1N(C(C)=O)C(N=N1)=O	2.1951
	C=C(C#N)C(O)=O	4.3011		CC(C#CCC)=O	5.4791		O=C1N(S(C)(=O)=O)C(N=N1)=O	2.1211
	O=C1C=C2CCC3C(CCC4(C)C(O)CCC34)C2(	4.5221		O=C(C#CC=1C=CC=CC1)C=2C=CC=CC2	3.6481		O=C1N(CC)C(N=N1)=O	2.6131
	O=C1C=C(C)CC(C)(C)C1	5.2911		C(#CC=1C=CC=CC1)C=2C=CC=CC2	5.1371		O=C1N(C=C)C(N=N1)=O	2.3451
	O=[N+](/C=C/C1=CC=CC=C1)[O-]	4.1001		N#CC#CC1=CC=CC=C1	4.8221		O=C1N(C2CCCCC2)C(N=N1)=O	2.4431
	O(C1=CC=C(C=C1)C(C=2C=CC=CC2)=C(C=3C=CC=CC3)CC)CCN(C)C	5.5781		OCC#CC	5.9191		O=C1N([Si](C)(C)C)C(N=N1)=O	2.3481
	O=[N+](/C=C/C1CCCCC1)[O-]	4.9371		C#CC(F)(F)F	6.7441		O=C1N(CC#C)C(N=N1)=O	2.8091

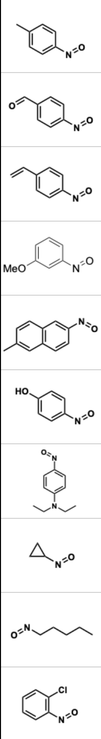
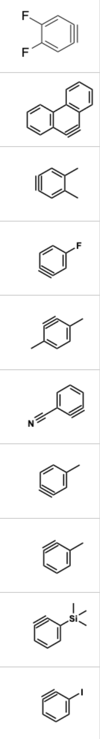
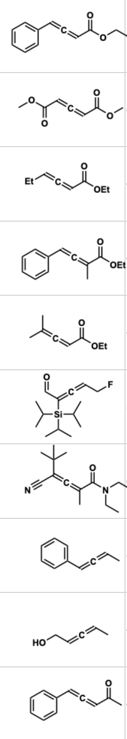

Nitroso		HOBO LUBO GAP	Aryne		HOBO LUBO GAP	Allene		HOBO LUBO GAP
	O=NC1=CC=C(C)C=C1	3.8331		FC1=CC#CC=C1F	3.4111		O=C(C=C=CC1=CC=CC=C1)OCC	5.7001
	O=NC1=CC=C(C=O)C=C1	3.1301		C12=C(C=CC=C2)C3=C(C=CC=C3)C#C1	4.6561		O=C(C=C=CC(OC)=O)OC	5.5571
	O=NC1=CC=C(C=C)C=C1	3.4771		CC1=CC#CC=C1C	4.5941		O=C(C=C=CCC)OCC	5.7671
	O=NC1=CC(OC)=CC=C1	4.0281		FC1=CC#CC=C1	4.0071		O=C(C(C)=C=CC1=CC=CC=C1)OCC	5.5921
	O=NC1=CC(C=CC(C)=C2)=C2C=C1	3.5561		CC1=CC=C(C)C#C1	4.5001		CC(C)=C=CC(OCC)=O	5.9081
	O=NC1=CC=C(O)C=C1	4.1891		N#CC1=CC=CC#C1	3.8481		FCC=C=C([Si](C(C)C)(C(C)C)C(C)C)C=O	5.5631
	O=NC1=CC=C(C=C1)N(CC)CC	4.6981		CC1=CC#CC=C1	4.3311		O=C(N(CC)CC)C(C)=C=C(C#N)C(C)(C)C	5.5021
	O=NC1CC1	4.4471		CC1=CC=CC#C1	4.6091		C(=CC=1C=CC=CC1)=CC	6.4941
	O=NCCCCC	4.3911		C[Si](C1=CC=CC#C1)(C)C	4.6651		OCC=C=CC	7.1531
	O=NC=1C=CC=CC1Cl	3.6051		IC1=CC=CC#C1	4.2671		O=C(C=C=CC=1C=CC=CC1)C	5.2461

Supplementary Fig. 12 | Predicted orbital energy gap.

Ketone		HOBO LUBO GAP	Ester		HOBO LUBO GAP	CHT/NCD		HOBO
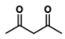	O=C(C)CC(=O)C	4.9361	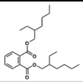	O=C(OCC(CC)CCCC)C=1C=CC=CC1C(=O)OCC(CC)CCCC	4.6801	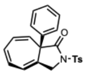	O=C1N(S(C2=CC=C(C)C=C2)(=O)=O)CC3=CC=CC=C[C@]13C4=CC=CC=C4	-6.278
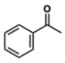	O=C(C=1C=CC=CC1)C	4.9101	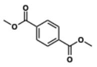	O=C(OC)C1=CC=C(C=C1)C(=O)OC	4.8221	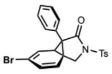	O=C1N(S(C2=CC=C(C)C=C2)(=O)=O)C[C@]34C=CC(Br)=CC3C14C5=CC=CC=C5	-6.307
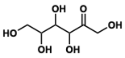	O=C(CO)C(O)C(O)C(O)CO	5.2491	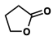	O=C1OCCC1	5.7581	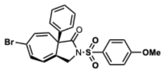	O=C1N(S(=O)(C2=CC=C(OC)C=C2)=O)CC3=CC=C(Br)C=C[C@]13C4=CC=CC=C4	-6.391
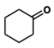	O=C1CCCCC1	5.4521	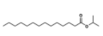	O=C(OC(C)C)CCCCCCCCCCCCC	6.1511	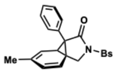	O=C1N(S(C2=CC=C(Br)C=C2)(=O)=O)C[C@]34C=CC(C)=CC3C14C5=CC=CC=C5	-6.18
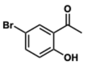	O=C(C1=CC(Br)=CC=C1O)C	5.1541	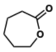	O=C1OCCCCC1	5.5461	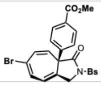	O=C1N(S(C2=CC=C(Br)C=C2)(=O)=O)CC3=CC=C(Br)C=C[C@]13C4=CC=C(C(OC)=O)C=C4	-6.607
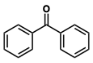	O=C(C=1C=CC=CC1)C=2C=CC=CC2	4.9181	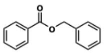	O=C(OCC=1C=CC=CC1)C=2C=CC=CC2	4.3231	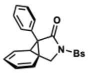	O=C1N(S(C2=CC=C(Br)C=C2)(=O)=O)C[C@]34C=CC=CC3C14C5=CC=CC=C5	-6.216
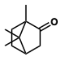	O=C1CC2CCC1(C)C2(C)C	5.2991	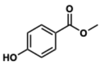	O=C(OC)C1=CC=C(O)C=C1	5.1631	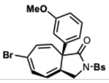	O=C1N(S(C2=CC=C(Br)C=C2)(=O)=O)CC3=CC=C(Br)C=C[C@]13C4=CC=CC(OC)=C4	-6.552
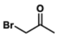	O=C(C)CBr	5.0841	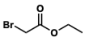	O=C(OCC)CBr	5.3471	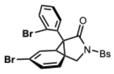	O=C1N(S(C2=CC=C(Br)C=C2)(=O)=O)C[C@]34C=CC(Br)=CC3C14C5=CC=CC=C5Br	-6.435
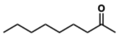	O=C(C)CCCCCCC	5.4931	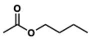	O=C(OCCCC)C	5.9651	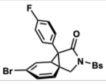	O=C1N(S(C2=CC=C(Br)C=C2)(=O)=O)C[C@]34C=CC(Br)=CC3C14C5=CC=C(F)C=C5	-6.38
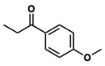	O=C(C1=CC=C(OC)C=C1)CC	4.9111	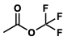	O=C(OC(F)(F)F)C	4.7131	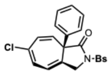	O=C1N(S(C2=CC=C(Br)C=C2)(=O)=O)CC3=CC=C(Cl)C=C[C@]13C4=CC=CC=C4	-6.475

Supplementary Fig. 13 | Predicted orbital energy gap.

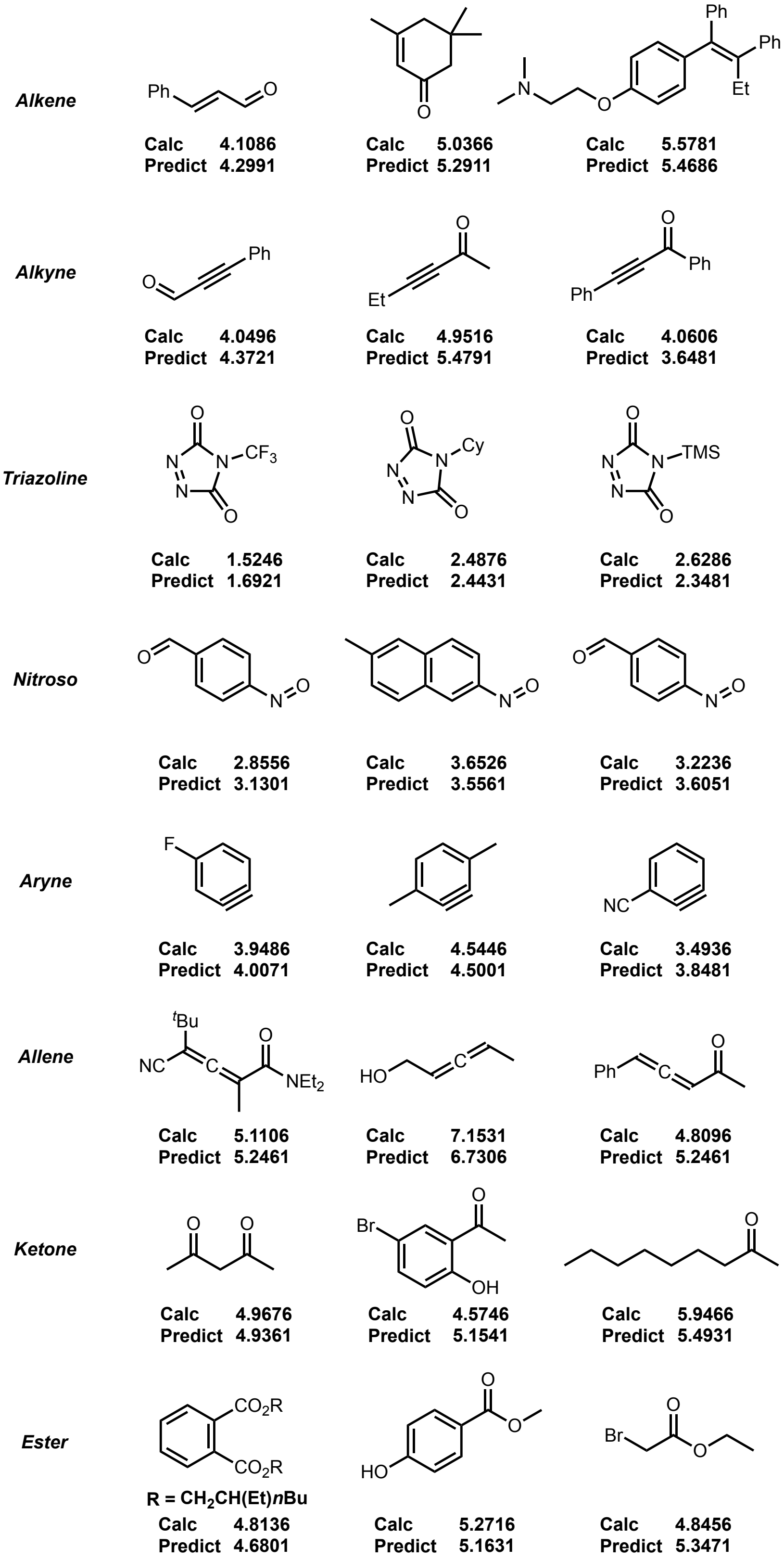

Supplementary Fig. 14 | Comparison of calculated and predicted energy gaps between enophile's LUBO and CHT/NCD's HOBO (10-case average).

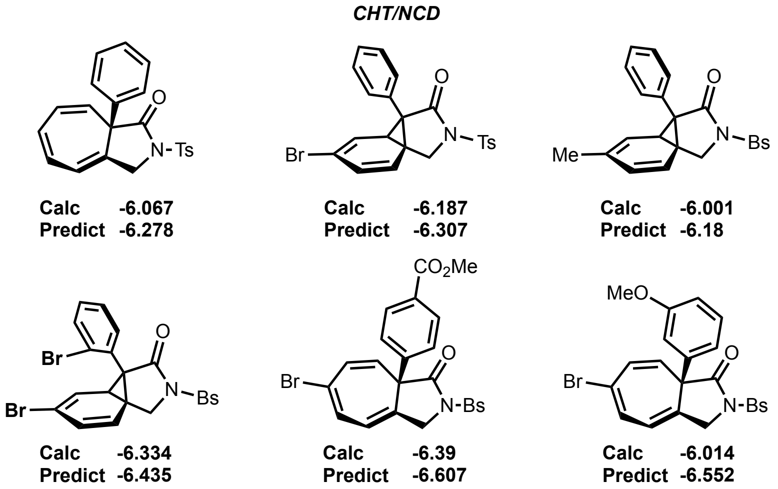

Supplementary Fig. 15 | Comparison of calculated and predicted HOBO energy values for CHT/NCD not included in training data.

2-3 Experimental Studies

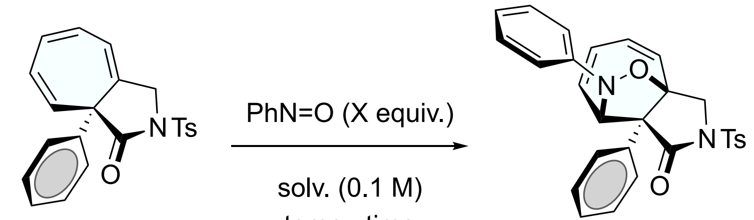

entry	X (equiv.)	solv.	temp. (°C)	time (h)	yield (%)[a]
1	2	THF	60	48	59
2	2	DCM	60	48	63
3	2	Toluene	60	48	60
4	2	PhCl	60	48	66
5	2	PhCl (0.05 M)	60	48	52
6	2	PhCl (0.2 M)	60	48	42
7	3	PhCl	60	48	72
8	5	PhCl	60	48	72
9	3	PhCl	80	48	trace
10	3	PhCl	50	48	54
11	3	PhCl	50	30	72
12	3	PhCl	50	24	59

a) Yields were determined by ^{1}H NMR using benzhydrol as an internal standard.

Supplementary Fig. 16 | Optimization of the cycloaddition reaction with nitroso compounds.

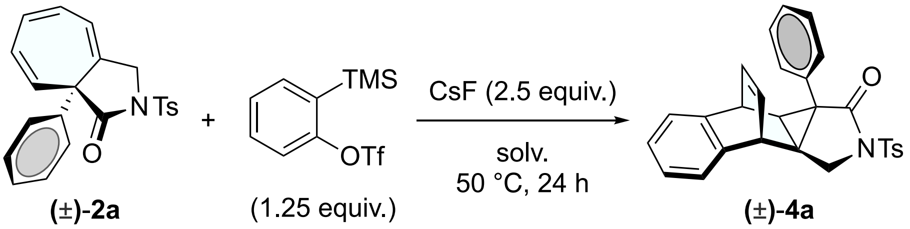

entry	solv.	(±)-4a yield (%)	(±)-2a recovered (%)
1	PhCl (0.2 M)	0	98
2	PhCl (0.2 M) + MeCN (0.2 M)	28	60
3	PhCl (0.1 M) + MeCN (0.05 M)	32	68
4	PhCl (0.1 M) + MeCN (0.025 M)	88	7
5	PhCl (0.1 M) + MeCN (0.01 M)	92	0

Supplementary Fig. 17 | Solvent effect of the cycloaddition reaction with arynes.

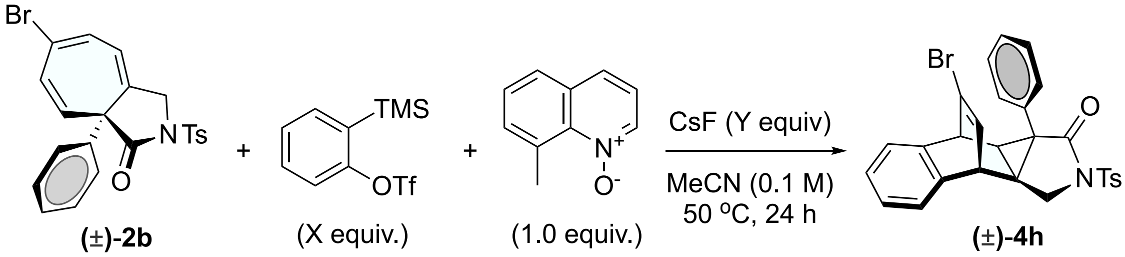

entry	X (equiv.)	Y (equiv.)	(±)-4h yield (%)	(±)-2b recovered (%)
1	1.25	2.5	17	74
2	3	6	48	50
3	5	10	85	0

Supplementary Fig. 18 | Optimization of the cycloaddition reaction with arynes.

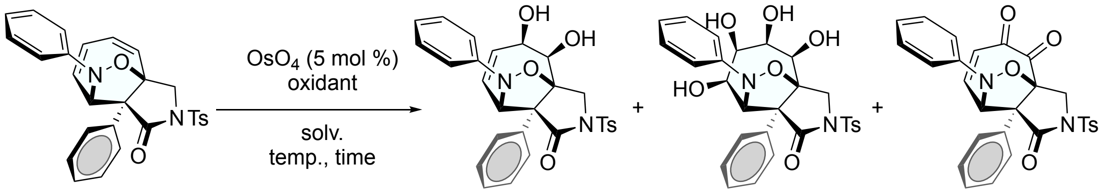

(±)-**3a** (±)-**10** (±)-**11** side product **A**

entry	oxidant (equivalent)	solv. (ratio, concentration)	temp. (°C)	time (h)	yield (%)[a] **10**	**11**	**A**
1	$K_3Fe(CN)_6$ (5 equiv.), K_2CO_3 (5 equiv.)	^{t}BuOH/H_2O (1:1, 0.05 M)	rt	42	0	0	0
2	^{t}BuOOH (2.5 equiv.)	^{t}BuOH/H_2O (4:1, 0.05 M)	rt	17	0	0	0
3[b]	none	^{t}BuOH/H_2O (4:1, 0.025 M)	rt	17	0	0	0
4	NMO (3 equiv.)	THF/^{t}BuOH/H_2O (6:8:1, 0.03 M)	rt	70	5	29	32
5	NMO (3 equiv.)	THF/^{t}BuOH/H_2O (6:8:1, 0.03 M)	50	27	49	5	-
6[c]	NMO (10 equiv.)	THF/^{t}BuOH/H_2O (6:8:1, 0.06 M)	60	48	-	39	58
7	NMO (1.2 equiv.)	THF/^{t}BuOH/H_2O (6:8:1, 0.06 M)	rt	4	72	-	-
8	NMO (1 equiv.)	THF/^{t}BuOH/H_2O (6:8:1, 0.06 M)	rt	2	82	-	-

[a)] Yields were determined by ^{1}H NMR using triphenylmethane as an internal standard.

[b)] OsO_4 (2.5 equiv.) was used.

[c)] OsO_4 (20 mol %) was used.

Supplementary Fig. 19 | Optimization of the dihydroxylation reaction.

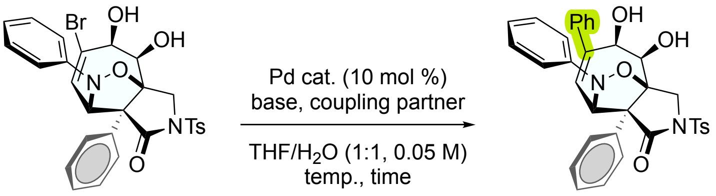

entry	Pd cat.	base (equivalent)	coupling partner (equivalent)	temp. (°C)	time (h)	yield (%)[a]
1	$Pd(OAc)_2/PPh_3$ (20 mol %)	K_2CO_3 (4 equiv.)	$PhB(OH)_2$ (1.5 equiv.)	80	20	0
2[b]	$Pd(OAc)/PPh_3$ (50 mol %)	TBAF (2 equiv.)	$PhSi(OMe)_3$ (2 equiv.)	80	24	0
3	$Pd(PPh_3)_4$	K_2CO_3 (4 equiv.)	$PhB(OH)_2$ (3 equiv.)	80	3	0
4[c]	$Pd(PPh_3)_4$	none	PhMgBr (4 equiv.)	50	24	0
5	$Pd_2Cl_2(PPh_3)_2$	K_2CO_3 (4 equiv.)/TEA (3 equiv.)	$PhB(OH)_2$ (3 equiv.)	80	24	0
6	$PdCl_2$(dppf)	K_2CO_3 (4 equiv.)	$PhB(OH)_2$ (3 equiv.)	80	4	0
7	$Pd_2(dba)_3$	K_2CO_3 (3 equiv.)	PhB(pin) (2 equiv.)	80	7	40
8	$Pd_2(dba)_3$	Cs_2CO_3 (3 equiv.)	PhB(pin) (2 equiv.)	80	7	44
9	$Pd_2(dba)_3$	CsF (3 equiv.)	PhB(pin) (2 equiv.)	80	7	trace
10	$Pd_2(dba)_3$	Na_2CO_3 (3 equiv.)	PhB(pin) (2 equiv.)	80	7	62
11	$Pd_2(dba)_3$	Na_2CO_3 (3 equiv.)	PhB(pin) (2 equiv.)	80	7	trace
12	$Pd_2(dba)_3$	Na_2CO_3 (3 equiv.)	PhB(pin) (2 equiv.)	100	7	trace
13[d]	$Pd_2(dba)_3$	Na_2CO_3 (3 equiv.)	PhB(pin) (2 equiv.)	100	7	20
14[e]	$Pd_2(dba)_3$	Na_2CO_3 (3 equiv.)	$PhB(OH)_2$ (2 equiv.)	80	7	86

a) Yields were determined by ^1H NMR using benzhydrol as an internal standard.
b) THF (0.1 M) was used.
c) THF (0.05 M) was used.
d) 2-Methyl-THF was used instead of THF.
e) Dioxane was used instead of THF.

Supplementary Fig. 20 | Optimization of the coupling reaction using Pd catalyst.

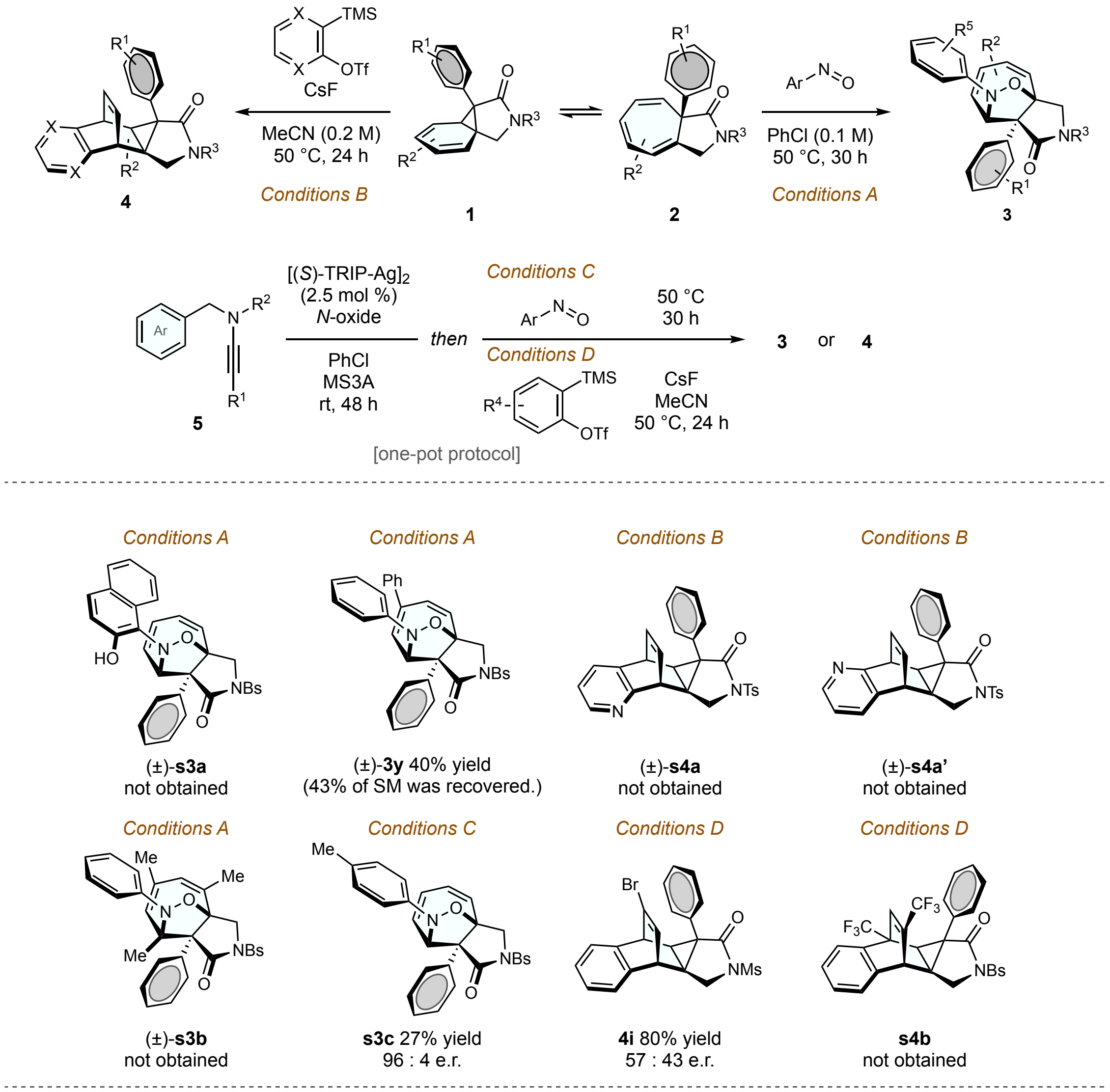

Supplementary Fig. 21 | Unsuccessful examples.

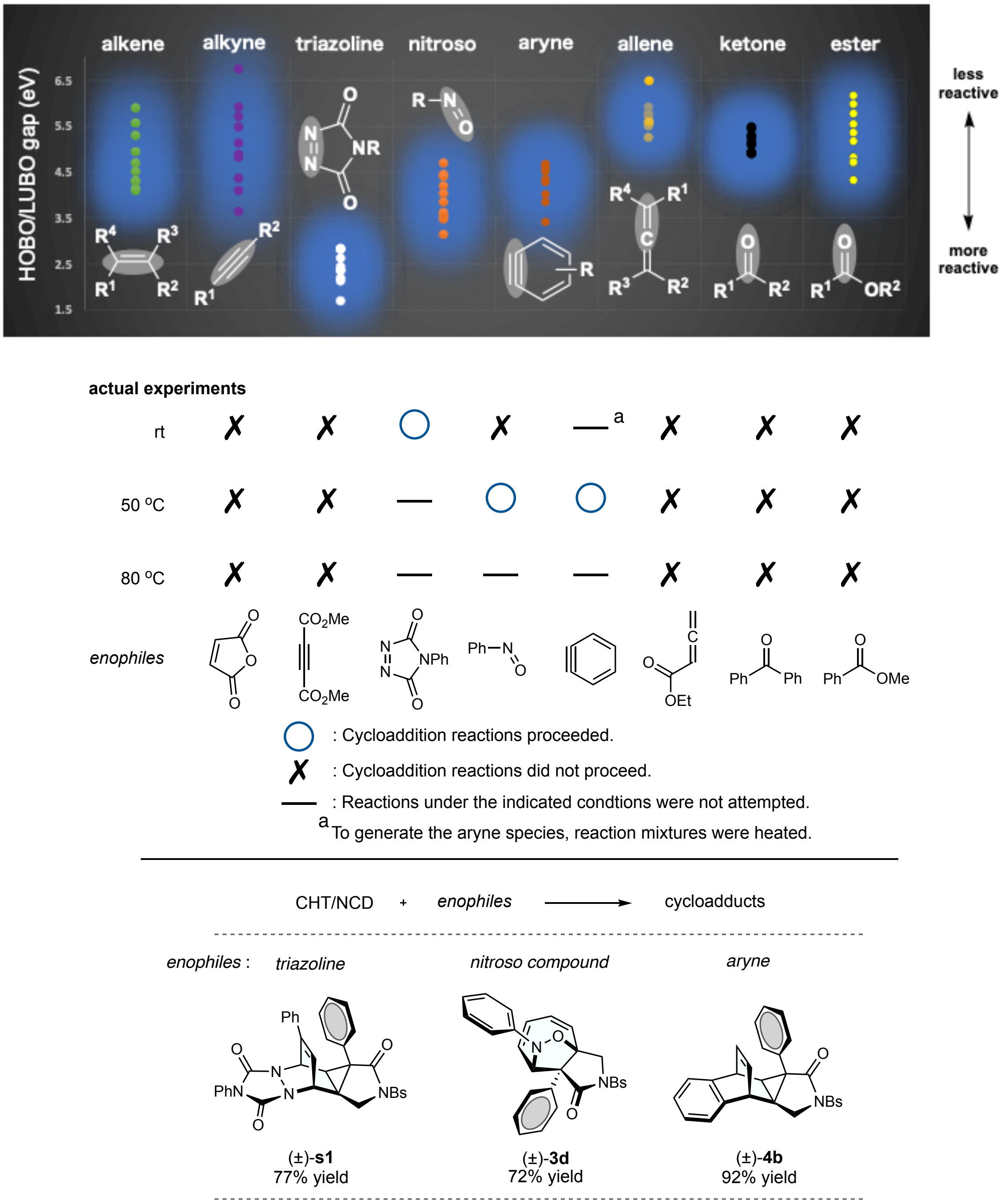

Supplementary Fig. 22 | Predictions and experiments.

3. Synthesis and Characterization of Cycloadducts with Nitroso Compounds (*Conditions A*)

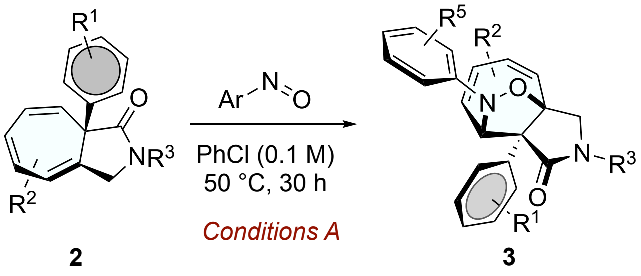

A pre-dried 10 mL test tube equipped with a magnetic stir bar was charged with cycloheptatriene compound **2** (0.1 mmol, 1 equiv.) and nitrosobenzene (0.3 mmol, 3 equiv.). Dry PhCl (1 mL, 0.1 M) was injected into the test tube under an argon gas atmosphere. The reaction mixture was stirred for 30 h at 50 °C. After the solvent was evaporated in vacuo, the resulting residue was purified by flash chromatography (*n*-hexane/EtOAc = 5/1, v/v) to afford desired product (±)-**3**.

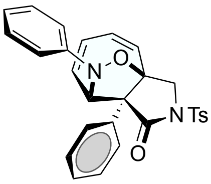

8a,9-Diphenyl-2-tosyl-2,3,8,8a-tetrahydro-1*H*-3a,8-(epoxyimino)cyclohepta[*c*]pyrrol-1-one ((±)-3a)

Prepared according to the general procedure A using **2a** (37.7 mg, 0.1 mmol) and isolated as yellow solid (34.9 mg, 72% yield): TLC R_f = 0.2 (*n*-hexane/EtOAc, 3/1, v/v); m.p. 110

ºC (decomp.); ^{1}H NMR (400 MHz, $CDCl_3$) δ 7.78 (d, *J* = 8.2 Hz, 2H), 7.29–7.24 (m, 3H), 7.20–7.17 (m, 4H), 7.05 (d, *J* = 8.2 Hz, 2H), 6.94 (t, *J* = 7.3 Hz, 1H), 6.76 (d, *J* = 7.8 Hz, 2H), 5.91 (d, *J* = 10.1 Hz, 1H), 5.80–5.73 (m, 3H), 5.02 (d, *J* = 5.5 Hz, 1H), 4.51 (d, *J* = 11.9 Hz, 1H), 4.43 (d, *J* = 11.9 Hz, 1H), 2.22 (s, 3H); ^{13}C NMR (100 MHz, $CDCl_3$) δ 172.1, 146.0, 145.0, 134.3, 132.9, 131.3, 130.3, 129.4, 129.3 (2C), 128.4 (2C), 128.2 (2C), 128.2, 127.8 (4C), 127.7, 122.4, 115.9 (2C), 82.1, 66.8, 64.3, 55.7, 21.5; IR (ATR) ν 3031, 2360, 1741, 1596, 1490, 1469, 1452, 1359, 1292, 1247 cm^{-1}; HRMS (ESI-TOF) [M + Na]$^+$ calcd for $C_{28}H_{24}N_2NaO_4S^+$ m/z 507.1349, found 507.1341.

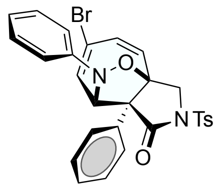

6-Bromo-8a,9-diphenyl-2-tosyl-2,3,8,8a-tetrahydro-1*H*-3a,8-(epoxyimino)cyclohepta[*c*]pyrrol-1-one ((±)-3b)

Prepared according to the general procedure A using **2b** (45.6 mg, 0.1 mmol) and isolated as yellow solid (37.8 mg, 67% yield): TLC R_f = 0.25 (*n*-hexane/EtOAc, 3/1, v/v); m.p. 95 ºC (decomp.); ^{1}H NMR (400 MHz, $CDCl_3$) δ 7.78 (d, *J* = 8.7 Hz, 2H), 7.34–7.29 (m, 3H), 7.24–6.98 (m, 7H), 6.76 (d, *J* = 7.8 Hz, 2H), 6.22 (d, *J* = 6.4 Hz, 1H), 5.98 (d, *J* = 11.2, 1H), 5.80 (d, *J* = 11.2 Hz, 1H), 4.90 (d, *J* = 6.4 Hz, 1H), 4.52 (d, *J* = 12.0 Hz, 1H), 4.42

(d, J = 12.0 Hz, 1H), 2.22 (s, 3H); ^{13}C NMR (100 MHz, $CDCl_3$) δ 171.5, 145.2, 145.1, 136.5 (2C), 134.0, 132.1, 130.2, 129.3 (2C), 128.6 (2C), 128.5 (2C), 127.8 (2C), 127.5 (2C), 123.6, 123.3 (2C), 116.3 (2C), 81.4, 67.6, 64.7, 55.7, 21.5; IR (ATR) ν 2925, 1741, 1672, 1595, 1493, 1452, 1402, 1363, 1309, 1236 cm^{-1}; HRMS (ESI-TOF) $[M + Na]^+$ calcd for $C_{28}H_{23}BrN_2NaO_4S^+$ m/z 585.0454, found 585.0470.

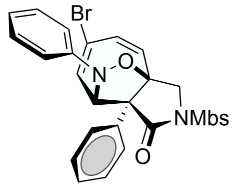

6-Bromo-2-((4-methoxyphenyl)sulfonyl)-8a,9-diphenyl-2,3,8,8a-tetrahydro-1*H*-3a,8-(epoxyimino)cyclohepta[*c*]pyrrol-1-one ((±)-3c)

Prepared according to the general procedure A using **2c** (47.2 mg, 0.1 mmol) and isolated as yellow solid (41.7 mg, 72% yield): TLC R_f = 0.1 (*n*-hexane/EtOAc, 3/1, v/v); m.p. 100 ºC (decomp.); ^{1}H NMR (400 MHz, $CDCl_3$) δ 7.83 (d, J = 8.2 Hz, 2H), 7.31–7.27 (m, 3H), 7.21 (t, J = 7.3 Hz, 2H), 7.14–7.12 (m, 2H), 6.99 (dt, J = 7.3, 0.8 Hz, 1H), 6.76 (d, J = 8.2 Hz, 2H), 6.71 (d, J = 8.2 Hz, 2H), 6.23 (d, J = 6.9 Hz, 1H), 5.98 (d, J = 11.0 Hz, 1H), 5.82 (d, J = 11.0 Hz, 1H), 4.91 (d, J = 6.9 Hz, 1H), 4.52 (d, J = 12.0 Hz, 1H), 4.43 (d, J = 12.0 Hz, 1H), 3.66 (s, 3H); ^{13}C NMR (100 MHz, $CDCl_3$) δ 171.4, 164.0, 145.2, 136.5 (2C), 132.1, 130.3, 130.2 (2C), 128.6 (2C), 128.6 (2C), 128.5, 127.5 (2C), 123.6, 123.1 (2C),

116.2 (2C), 113.8 (2C), 81.4, 67.6, 64.6, 55.6, 55.5; IR (ATR) ν 3014, 3014, 2360, 1745, 1595, 1495, 1363, 1219, 1165, 1016 cm^{-1}; HRMS (ESI-TOF) $[M + H]^+$ calcd for $C_{28}H_{24}BrN_2O_5S^+$ m/z 579.0584, found 579.0594.

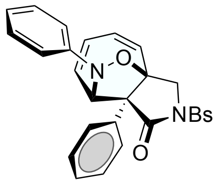

2-((4-Bromophenyl)sulfonyl)-8a,9-diphenyl-2,3,8,8a-tetrahydro-1*H*-3a,8-(epoxyimino)cyclohepta[*c*]pyrrol-1-one ((±)-3d)

Prepared according to the general procedure A using **2d** (44.2 mg, 0.1 mmol) and isolated as yellow solid (39.6 mg, 72% yield): TLC R_f = 0.25 (*n*-hexane/EtOAc, 3/1, v/v); m.p. 100 ºC (decomp.); ^{1}H NMR (400 MHz, $CDCl_3$) δ 7.74 (d, *J* = 8.2 Hz, 2H), 7.37 (d, *J* = 8.2 Hz, 2H), 7.30–7.25 (m, 3H), 7.23–7.18 (m, 4H), 6.97 (t, *J* = 7.6 Hz, 1H), 6.76 (d, *J* = 7.6 Hz, 2H), 5.90 (d, *J* = 11.5 Hz, 1H), 5.80–5.70 (m, 3H), 5.01 (d, *J* = 5.0 Hz, 1H), 4.50 (d, *J* = 12.0 Hz, 1H), 4.44 (d, *J* = 12.0 Hz, 1H); ^{13}C NMR (100 MHz, $CDCl_3$) 172.3, 145.9, 136.2, 132.7, 132.0 (2C), 131.4, 130.3, 129.4, 129.3 (3C), 128.6 (2C), 128.3, 128.3 (2C), 127.8 (2C), 127.4, 122.7, 115.8 (2C), 82.3, 66.9, 64.3, 55.6; IR (ATR) ν 3031, 1743, 1597, 1574, 1491, 1390, 1365, 1281, 1248, 1230 cm^{-1}; HRMS (ESI-TOF) $[M + Na]^+$ calcd for $C_{27}H_{21}BrN_2NaO_4S^+$ m/z 571.0295, found 571.0315.

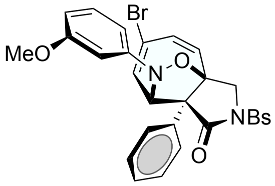

6-Bromo-2-((4-bromophenyl)sulfonyl)-9-(3-methoxyphenyl)-8a-phenyl-2,3,8,8a-tetrahydro-1*H*-3a,8-(epoxyimino)cyclohepta[*c*]pyrrol-1-one ((±)-3e)

Prepared according to the general procedure A using **2e** (50.5 mg, 0.1 mmol), methoxy 3-nitrosobenzoate (68.6 mg, 0.3 mmol) and isolated as yellow solid (49.1 mg, 75% yield): TLC R_f = 0.2 (*n*-hexane/EtOAc, 3/1, v/v); m.p. 100 ºC (decomp.); ^{1}H NMR (400 MHz, $CDCl_3$) δ 7.74 (d, *J* = 8.7 Hz, 2H), 7.39 (d, *J* = 8.7 Hz, 2H), 7.34–7.27 (m, 3H), 7.17–7.11 (m, 3H), 6.58 (dd, *J* = 8.2, 2.3 Hz, 1H), 6.37 (t, *J* = 2.3 Hz, 1H), 6.32 (d, *J* = 8.2 Hz, 1H), 6.23 (d, *J* = 7.2 Hz, 1H), 5.98 (dd, *J* = 11.2, 1.2 Hz, 1H), 5.80 (d, *J* = 11.2 Hz, 1H), 4.90 (d, *J* = 7.2 Hz, 1H), 4.53 (d, *J* = 12.0 Hz, 1H), 4.44 (d, *J* = 12.0 Hz, 1H), 3.80 (s, 3H); ^{13}C NMR (100 MHz, $CDCl_3$) δ 171.6, 160.0, 146.1, 136.6, 135.9, 132.0 (2C), 131.8, 130.0, 129.6, 129.5, 129.3 (2C), 128.7 (3C), 128.3, 127.5 (2C), 123.7, 108.9, 108.7, 102.4, 81.5, 67.7, 64.6, 55.7, 55.3; IR (ATR) ν 3689, 3648, 3025, 2365, 2356, 2314, 1745, 1599, 1486, 1391 cm^{-1}; HRMS (ESI-TOF) $[M + Na]^+$ calcd for $C_{28}H_{22}Br_2N_2NaO_5S^+$ m/z 678.9508, found 678.9510.

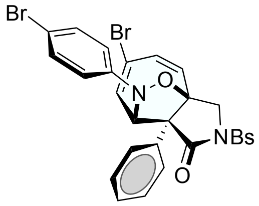

6-Bromo-9-(4-bromophenyl)-2-((4-bromophenyl)sulfonyl)-8a-phenyl-2,3,8,8a-tetrahydro-1*H*-3a,8-(epoxyimino)cyclohepta[*c*]pyrrol-1-one ((±)-3f)

Prepared according to the general procedure A using **2e** (52.1 mg, 0.1 mmol), 1-bromo-4-nitrosobenzene (55.8 mg, 0.3 mmol) and isolated as yellow solid (60.8 mg, 86% yield): TLC R_f = 0.2 (*n*-hexane/EtOAc, 3/1, v/v); m.p. 100 ºC (decomp.); ^{1}H NMR (400 MHz, $CDCl_3$) δ 7.74 (d, *J* = 8.7 Hz, 2H), 7.41 (d, *J* = 8.7 Hz, 2H), 7.36–7.29 (m, 5H), 7.12 (d, *J* = 6.4 Hz, 2H), 6.62 (d, *J* = 8.7 Hz, 2H), 6.19 (d, *J* = 6.9 Hz, 1H), 5.98 (dd, *J* = 11.2, 1.4 Hz, 1H), 5.80 (d, *J* = 11.2 Hz, 1H), 4.87 (d, *J* = 6.9 Hz, 1H), 4.50 (d, *J* = 12.0 Hz, 1H), 4.44 (d, *J* = 12.0 Hz, 1H); ^{13}C NMR (100 MHz, $CDCl_3$) δ 171.5, 144.1, 136.6, 135.9, 132.0 (2C), 131.7 (2C), 131.6, 129.7, 129.6, 129.3 (2C), 128.8, 128.7 (2C), 128.2, 127.4 (2C), 124.0, 117.8 (2C), 116.3, 81.7, 67.7, 64.5, 55.5; IR (ATR) ν 3089, 2337, 1574, 1369, 1279, 1234, 1136, 1086, 1068, 1009 cm^{-1}; HRMS (ESI-TOF) $[M + Na]^+$ calcd for $C_{27}H_{19}Br_3N_2NaO_4S^+$ m/z 726.8508, found 726.8508.

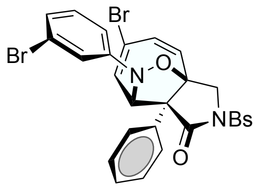

6-Bromo-9-(3-bromophenyl)-2-((4-bromophenyl)sulfonyl)-8a-phenyl-2,3,8,8a-tetrahydro-1*H*-3a,8-(epoxyimino)cyclohepta[*c*]pyrrol-1-one ((±)-3g)

Prepared according to the general procedure A using **2e** (52.1 mg, 0.1 mmol), 1-bromo-3-nitrosobenzene (55.8 mg, 0.3 mmol) and isolated as yellow solid (58.0 mg, 82% yield): TLC R_f = 0.2 (*n*-hexane/EtOAc, 3/1, v/v); m.p. 90 ºC (decomp.); ^{1}H NMR (600 MHz, $CDCl_3$) δ 7.74 (d, *J* = 9.0 Hz, 2H), 7.42(d, *J* = 8.3 Hz, 2H), 7.34–7.30 (m, 3H), 7.16–7.09 (m, 4H), 6.97 (d, *J* = 2.1 Hz, 1H), 6.64 (dd, *J* = 8.3, 1.4 Hz, 1H), 6.24 (d, *J* = 6.9 Hz, 1H), 6.00 (dd, *J* = 11.7, 1.4 Hz, 1H), 5.81 (d, *J* = 11.7 Hz, 1H), 4.91 (d, *J* = 6.9 Hz, 1H), 4.51 (d, *J* = 12.4 Hz, 1H), 4.43 (d, *J* = 12.4 Hz, 1H); ^{13}C NMR (100 MHz, $CDCl_3$) δ 171.4, 146.1, 136.7, 135.8, 132.0 (2C), 131.5, 130.1, 129.6, 129.4, 129.3 (2C), 128.8, 128.7 (2C), 128.2, 127.4 (2C), 126.3, 124.1, 122.8, 119.1, 114.4, 81.7, 67.3, 64.5, 55.5; IR (ATR) ν 3062, 1745, 1587, 1574, 1471, 1425, 1367, 1234, 1171, 1136 cm^{-1}; HRMS (ESI-TOF) $[M + Na]^+$ calcd for $C_{27}H_{19}Br_3N_2NaO_4S^+$ m/z 726.8508, found 726.8513.

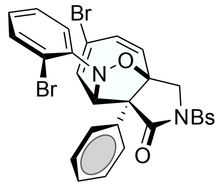

6-Bromo-9-(2-bromophenyl)-2-((4-bromophenyl)sulfonyl)-8a-phenyl-2,3,8,8a-tetrahydro-1*H*-3a,8-(epoxyimino)cyclohepta[*c*]pyrrol-1-one ((±)-3h)

Prepared according to the general procedure A using **2e** (52.1 mg, 0.1 mmol), 1-bromo-2-nitrosobenzene (55.8 mg, 0.3 mmol) and isolated as yellow solid (52.3 mg, 74% yield): TLC R_f = 0.2 (*n*-hexane/EtOAc, 3/1, v/v); m.p. 130 ºC (decomp.); ^{1}H NMR (400 MHz, $CDCl_3$) δ 7.80 (d, *J* = 8.7 Hz, 2H), 7.51 (dd, *J* = 8.2, 1.4 Hz, 2H) , 7.43 (d, *J* = 8.7 Hz, 2H), 7.37 (dd, *J* = 8.2, 1.4 Hz, 1H), 7.32–7.27 (m, 4H), 7.17–7.15 (m, 2H), 7.03 (dt, *J* = 8.0, 2.0 Hz, 1H), 6.00 (dd, *J* = 11.4, 1.4 Hz, 1H), 5.91 (d, *J* = 6.9 Hz, 1H), 5.82 (d, *J* = 11.4 Hz, 1H), 5.39 (d, *J* = 6.9 Hz, 1H), 4.50 (d, *J* = 12.0 Hz, 1H), 4.45 (d, *J* = 12.0 Hz, 1H); ^{13}C NMR (100 MHz, $CDCl_3$) δ 170.6, 142.6, 136.5, 136.3, 133.2, 132.1 (2C), 131.9, 130.1, 129.6, 129.4 (2C), 128.7 (3C), 128.6, 127.8, 127.5 (2C), 126.6, 124.2, 120.9, 113.9, 81.0, 66.1, 64.8, 55.9; IR (ATR) ν 3062, 2308, 1749, 1574, 1468, 1367, 1304, 1279, 1232, 1173 cm^{-1}; HRMS (ESI-TOF) [M + Na]$^+$ calcd for $C_{27}H_{19}Br_3N_2NaO_4S^+$ m/z 726.8508, found 726.8505.

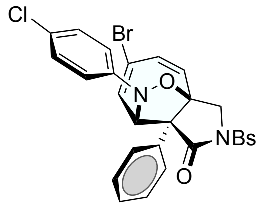

6-Bromo-2-((4-bromophenyl)sulfonyl)-9-(4-chlorophenyl)-8a-phenyl-2,3,8,8a-tetrahydro-1*H*-3a,8-(epoxyimino)cyclohepta[*c*]pyrrol-1-one ((±)-3i)

Prepared according to the general procedure A using **2e** (52.1 mg, 0.1 mmol), 1-chloro-4-nitrosobenzene (42.5 mg, 0.3 mmol) and isolated as yellow solid (51.0 mg, 77% yield): TLC R_f = 0.2 (*n*-hexane/EtOAc, 3/1, v/v); m.p. 80 ºC (decomp.); ^{1}H NMR (400 MHz, $CDCl_3$) δ 7.74 (d, *J* = 8.4 Hz, 2H), 7.41 (d, *J* = 8.4 Hz, 2H), 7.33–7.27 (m, 3H), 7.20 (d, *J* = 8.7 Hz, 2H), 7.12 (dd, *J* = 6.4, 1.6 Hz, 2H), 6.69 (d, *J* = 8.7 Hz, 2H), 6.19 (d, *J* = 6.9 Hz, 1H), 5.98 (dd, *J* = 11.0, 0.9 Hz, 1H), 5.80 (d, *J* = 11.0 Hz, 1H), 4.87 (d, *J* = 6.9 Hz, 1H), 4.51(d, *J* = 12.0 Hz, 1H), 4.44 (d, *J* = 12.0 Hz, 1H); ^{13}C NMR (100 MHz, $CDCl_3$) δ 171.5, 143.6, 136.6, 135.9, 132.0 (2C), 131.6, 129.7, 129.6, 129.3 (2C), 128.8 (2C), 128.8 (2C), 128.7 (2C), 128.3, 127.4 (2C), 124.0, 117.5 (2C), 81.7, 67.9, 64.6, 55.6; IR (ATR) ν 3087, 2360, 1743, 1574, 1487, 1367, 1281, 1236, 1173, 1088 cm^{-1}; HRMS (ESI-TOF) $[M + Na]^+$ calcd for $C_{27}H_{19}Br_2ClN_2NaO_4S^+$ m/z 682.9013, found 682.9044.

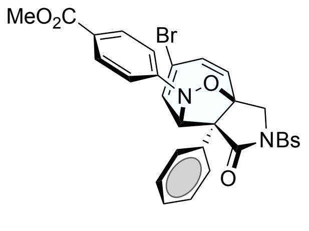

Methyl 4-(6-bromo-2-((4-bromophenyl)sulfonyl)-1-oxo-8a-phenyl-2,3,8,8a-tetrahydro-1*H*-3a,8-(epoxyimino)cyclohepta[*c*]pyrrol-9-yl)benzoate ((±)-3j)

Prepared according to the general procedure A using **2e** (52.1 mg, 0.1 mmol), methyl 4-nitrosobenzoate (49.5 mg, 0.3 mmol) and isolated as brown solid (55.6 mg, 81% yield): TLC R_f = 0.1 (*n*-hexane/EtOAc, 3/1, v/v); m.p. 130 ºC (decomp.); ^{1}H NMR (400 MHz, $CDCl_3$) δ 7.93 (d, *J* = 8.7 Hz, 2H), 7.73 (d, *J* = 8.7 Hz, 2H), 7.39 (d, *J* = 8.7 Hz, 2H), 7.36–7.30 (m, 3H), 7.15 (d, *J* = 6.4 Hz, 2H), 6.74 (d, *J* = 8.7 Hz, 2H), 6.29 (d, *J* = 7.3 Hz, 1H), 6.01 (dd, *J* = 11.2, 1.0 Hz, 1H), 5.85 (d, *J* = 11.2 Hz, 1H), 5.02 (d, *J* = 7.3 Hz, 1H), 4.52(d, *J* = 12.4 Hz, 1H), 4.45 (d, *J* = 12.4 Hz, 1H), 3.92 (s, 3H); ^{13}C NMR (100 MHz, $CDCl_3$) δ 171.2, 166.6, 149.0, 136.7, 135.7, 132.1 (2C), 131.4, 130.7 (2C), 129.7, 129.5, 129.3 (2C), 128.9, 128.8 (2C), 128.0, 127.3 (2C), 124.4, 124.1, 114.7 (2C), 81.9, 66.8, 64.3, 55.1, 52.0; IR (ATR) ν 3073, 3070, 2360, 1745, 1712, 1603, 1502, 1435, 1369, 1281 cm^{-1}; HRMS (ESI-TOF) [M + Na]$^+$ calcd for $C_{29}H_{22}Br_2N_2NaO_6S^+$ m/z 706.9458, found 706.9455.

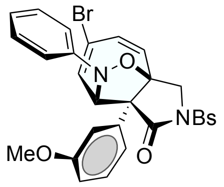

6-Bromo-2-((4-bromophenyl)sulfonyl)-8a-(3-methoxyphenyl)-9-phenyl-2,3,8,8a-tetrahydro-1*H*-3a,8-(epoxyimino)cyclohepta[*c*]pyrrol-1-one ((±)-3k)

Prepared according to the general procedure A using **2k** (55.1 mg, 0.1 mmol) and isolated as brown solid (40.2 mg, 61% yield): TLC R_f = 0.2 (*n*-hexane/EtOAc, 3/1, v/v); m.p. 110 ºC (decomp.); ^{1}H NMR (400 MHz, $CDCl_3$) δ 7.74 (d, *J* = 8.7 Hz, 2H), 7.39 (d, *J* = 8.7 Hz, 2H), 7.22 (t, *J* = 8.2 Hz, 3H), 7.02 (t, *J* = 7.3 Hz, 1H), 6.85 (dd, *J* = 8.2, 2.3 Hz, 1H), 6.75 (d, *J* = 7.8 Hz, 2H), 6.70 (d, *J* = 7.8 Hz, 1H), 6.64 (t, *J* = 2.3 Hz, 1H), 6.21 (d, *J* = 6.9 Hz, 1H), 6.01 (dd, *J* = 11.5, 0.9 Hz, 1H), 5.79 (d, *J* = 11.5 Hz, 1H), 4.87 (d, *J* = 6.9 Hz, 1H), 4.51 (d, *J* = 12.4 Hz, 1H), 4.41 (d, *J* = 12.4 Hz, 1H), 3.77 (s, 3H); ^{13}C NMR (100 MHz, $CDCl_3$) δ 171.5, 159.4, 145.0, 136.6, 136.0, 133.1, 132.0 (2C), 130.2, 129.7, 129.5, 129.3 (2C), 128.8 (2C), 128.3, 123.7, 123.5, 119.8, 116.2 (2C), 114.8, 113.0, 81.5, 67.8, 64.5, 55.5, 55.3; IR (ATR) ν 2968, 2360, 1745, 1597, 1491, 1471, 1369, 1234, 1173, 1138 cm^{-1}; HRMS (ESI-TOF) [M + Na]$^+$ calcd for $C_{28}H_{22}Br_2N_2NaO_5S^+$ m/z 678.9508, found 678.9516.

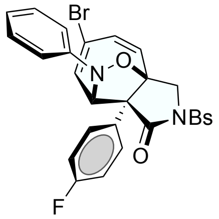

6-Bromo-2-((4-bromophenyl)sulfonyl)-8a-(4-fluorophenyl)-9-phenyl-2,3,8,8a-tetrahydro-1*H*-3a,8-(epoxyimino)cyclohepta[*c*]pyrrol-1-one ((±)-3l)

Prepared according to the general procedure A using **2l** (53.9 mg, 0.1 mmol) and isolated as yellow solid (46.5 mg, 72% yield): TLC R_f = 0.1 (*n*-hexane/EtOAc, 3/1, v/v); m.p. 120 ºC (decomp.); ^{1}H NMR (400 MHz, $CDCl_3$) δ 7.74 (d, *J* = 8.7 Hz, 2H), 7.39 (d, *J* = 8.7 Hz, 2H), 7.25 (t, *J* = 7.8 Hz, 2H), 7.16–7.12 (m, 2H), 7.06–6.99 (m, 3H), 6.74 (d, *J* = 7.8 Hz, 2H), 6.21 (d, *J* = 6.9 Hz, 1H), 6.02 (dd, *J* = 11.0, 1.4 Hz, 1H), 5.81 (d, *J* = 11.0 Hz, 1H), 4.86 (d, *J* = 6.9 Hz, 1H), 4.52 (d, *J* = 11.9 Hz, 1H), 4.42 (d, *J* = 11.9 Hz, 1H); ^{13}C NMR (150 MHz, $CDCl_3$) δ 171.4, 162.6 (d, *J* = 248.5 Hz, 1C), 144.9, 136.9, 136.0, 132.1 (2C), 130.1, 129.6, 129.4 (2C), 129.3 (d, *J* = 8.7 Hz, 2C), 128.8 (2C), 128.2, 127.8 (d, *J* = 2.9 Hz, 1C), 123.9, 123.7, 116.2 (2C), 115.9 (d, *J* = 21.7 Hz, 2C), 81.4, 68.1, 64.0, 55.5; ^{19}F NMR (564 MHz, $CDCl_3$) δ –111.8; IR (ATR) ν 3070, 1745, 1597, 1574, 1512, 1491, 1471, 1390, 1347, 1309 cm^{-1}; HRMS (ESI-TOF) [M + Na]$^+$ calcd for $C_{27}H_{19}Br_2FN_2NaO_4S^+$ m/z 666.9309, found 666.9303.

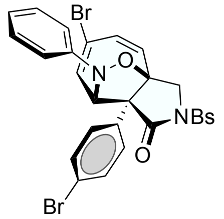

6-Bromo-8a-(4-bromophenyl)-2-((4-bromophenyl)sulfonyl)-9-phenyl-2,3,8,8a-tetrahydro-1*H*-3a,8-(epoxyimino)cyclohepta[*c*]pyrrol-1-one ((±)-3m)

Prepared according to the general procedure A using **2m** (60.0 mg, 0.1 mmol) and isolated as yellow solid (30.4 mg, 43% yield): TLC R_f = 0.3 (*n*-hexane/EtOAc, 3/1, v/v); m.p. 120 ºC (decomp.); ^{1}H NMR (400 MHz, $CDCl_3$) δ 7.74 (d, *J* = 8.2 Hz, 2H), 7.45 (d, *J* = 8.2 Hz, 2H), 7.39 (d, *J* = 8.2 Hz, 2H), 7.25 (t, *J* = 8.0 Hz, 2H), 7.05–7.01 (m, 3H), 6.74 (d, *J* = 8.7 Hz, 2H), 6.20 (d, *J* = 6.9 Hz, 1H), 6.02 (d, *J* = 11.2 Hz, 1H), 5.80 (d, *J* = 11.2 Hz, 1H), 4.85 (d, *J* = 6.9 Hz, 1H), 4.52 (d, *J* = 12.4 Hz, 1H), 4.40 (d, *J* = 12.4 Hz, 1H); ^{13}C NMR (100 MHz, $CDCl_3$) δ 171.1, 144.7, 136.9, 135.9, 132.1 (2C), 132.0 (2C), 130.9, 130.0, 129.6, 129.4 (2C), 129.1(2C), 128.8 (2C), 128.2, 123.9, 123.7, 123.1, 116.2 (2C), 81.3, 67.8, 64.3, 55.6; IR (ATR) ν 3062, 1745, 1595, 1574, 1491, 1471, 1390, 1347, 1277, 1234 cm^{-1}; HRMS (ESI-TOF) $[M + Na]^+$ calcd for $C_{27}H_{19}Br_3N_2NaO_4S^+$ m/z 726.8508, found 726.8524.

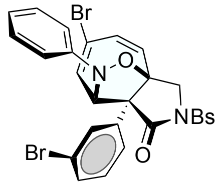

6-Bromo-8a-(3-bromophenyl)-2-((4-bromophenyl)sulfonyl)-9-phenyl-2,3,8,8a-tetrahydro-1*H*-3a,8-(epoxyimino)cyclohepta[*c*]pyrrol-1-one ((±)-3n)

Prepared according to the general procedure A using **2n** (60.0 mg, 0.1 mmol) and isolated as yellow solid (33.9 mg, 48% yield): TLC R_f = 0.4 (*n*-hexane/EtOAc, 3/1, v/v); m.p. 120

ºC (decomp.); ^{1}H NMR (400 MHz, $CDCl_3$) δ 7.74 (d, J = 7.8 Hz, 2H), 7.48 (dt, J = 8.2, 0.9 Hz, 1H), 7.40 (d, J = 8.7 Hz, 2H), 7.28–7.23 (m, 3H), 7.20 (t, J = 7.8 Hz, 1H), 7.10–7.03 (m, 2H), 6.75 (d, J = 7.8 Hz, 2H), 6.22 (d, J = 6.8 Hz, 1H), 6.04 (dd, J = 11.0, 1.2 Hz, 1H), 5.84 (d, J = 11.0 Hz, 1H), 4.85 (d, J = 6.8 Hz, 1H), 4.53 (d, J = 12.0 Hz, 1H), 4.41 (d, J = 12.0 Hz, 1H); ^{13}C NMR (100 MHz, $CDCl_3$) δ 171.0, 144.7, 137.0, 135.8, 133.9, 132.1 (2C), 131.9, 130.5, 130.2, 130.0, 129.7, 129.3 (2C), 128.8 (2C), 128.0, 126.2, 124.0, 123.8, 122.7, 116.3 (2C), 81.3, 67.8, 64.3, 55.5; IR (ATR) ν 3029, 1743, 1593, 1572, 1572, 1473, 1367, 1234, 1171, 1138 cm^{-1}; HRMS (ESI-TOF) $[M + Na]^+$ calcd for $C_{27}H_{19}Br_3N_2NaO_4S^+$ m/z 726.8508, found 726.8500.

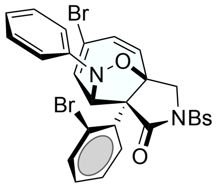

6-Bromo-8a-(2-bromophenyl)-2-((4-bromophenyl)sulfonyl)-9-phenyl-2,3,8,8a-tetrahydro-1*H*-3a,8-(epoxyimino)cyclohepta[*c*]pyrrol-1-one ((±)-3o)

Prepared according to the general procedure A using **1o** (60.0 mg, 0.1 mmol) and isolated as brown solid (49.9 mg, 71% yield): TLC R_f = 0.2 (*n*-hexane/EtOAc, 3/1, v/v); m.p. 130 ºC (decomp.); ^{1}H NMR (400 MHz, $CDCl_3$) δ 7.74 (d, J = 8.8 Hz, 2H), 7.48 (dq, J = 7.8, 0.9 Hz, 1H), 7.40 (d, J = 8.8 Hz, 2H), 7.28–7.23 (m, 3H), 7.20 (t, J = 7.8 Hz, 1H), 7.10–

7.02 (m, 2H), 6.75 (dd, J = 8.7, 0.9 Hz, 2H), 6.22 (d, J = 6.8 Hz, 1H), 6.04 (dd, J = 11.2, 1.4 Hz, 1H), 5.84 (d, J = 11.2 Hz, 1H), 4.85 (d, J = 6.8 Hz, 1H), 4.53 (d, J = 12.4 Hz, 1H), 4.41 (d, J = 12.4 Hz, 1H); ^{13}C NMR (150 MHz, $CDCl_3$) δ 177.4, 144.7, 137.0, 135.9, 134.0, 132.1 (2C), 132.0, 130.6, 130.2, 130.0, 129.7, 129.4 (2C), 128.8 (2C), 128.0, 126.2, 124.0, 123.8, 122.7, 116.3 (2C), 81.3, 67.9, 64.3, 55.6; IR (ATR) ν 3089, 1732, 1622, 1574, 1471, 1437, 1389, 1367, 1308, 1277 cm^{-1}; HRMS (ESI-TOF) [M + Na]$^+$ calcd for $C_{27}H_{19}Br_3N_2NaO_4S^+$ m/z 726.8508, found 726.8503.

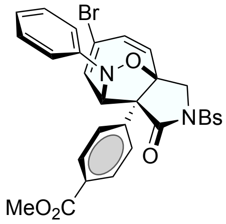

Methyl 4-(6-bromo-2-((4-bromophenyl)sulfonyl)-1-oxo-9-phenyl-2,3-dihydro-1*H*-3a,8-(epoxyimino)cyclohepta[*c*]pyrrol-8a(8*H*)-yl)benzoate ((±)-3p)

Prepared according to the general procedure A using **2p** (57.9 mg, 0.1 mmol) and isolated as yellow solid (35.0 mg, 51% yield): TLC R_f = 0.3 (*n*-hexane/EtOAc, 3/1, v/v); m.p. 100 ºC (decomp.); ^{1}H NMR (400 MHz, $CDCl_3$) δ 7.97 (d, J = 8.2 Hz, 2H), 7.74 (d, J = 8.2 Hz, 2H), 7.39 (d, J = 8.2 Hz, 2H), 7.27–7.22 (m, 4H), 7.04 (t, J = 7.3 Hz, 1H), 6.75 (d, J = 8.2 Hz, 2H), 6.22 (d, J = 6.9 Hz, 1H), 5.99 (d, J = 11.2 Hz, 1H), 5.83 (d, J = 11.2 Hz, 1H), 4.92 (d, J = 6.9 Hz, 1H), 4.55 (d, J = 12.4 Hz, 1H), 4.46 (d, J = 12.4 Hz, 1H), 3.92

(s, 3H); ^{13}C NMR (100 MHz, $CDCl_3$) δ 170.9, 166.1, 144.7, 136.8, 136.6, 135.8, 132.1 (2C), 130.4, 130.0, 129.8 (2C), 129.6, 129.4 (2C), 128.8 (2C), 128.2, 127.7 (2C), 123.9, 123.7, 116.2 (2C), 81.4, 67.9, 64.8, 55.7, 52.3; IR (ATR) ν 2952, 1722, 1597, 1574, 1491, 1471, 1435, 1367, 1282, 1236 cm^{-1}; HRMS (ESI-TOF) $[M + Na]^+$ calcd for $C_{29}H_{22}Br_2N_2NaO_6S^+$ m/z706.9458, found 706.9461.

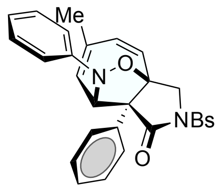

2-((4-Bromophenyl)sulfonyl)-6-methyl-8a,9-diphenyl-2,3,8,8a-tetrahydro-1*H*-3a,8-(epoxyimino)cyclohepta[*c*]pyrrol-1-one ((±)-3q)

Prepared according to the general procedure A using **2q** (45.6 mg, 0.1 mmol) and isolated as brown solid (19.4 mg, 34% yield): TLC R_f = 0.2 (*n*-hexane/EtOAc, 3/1, v/v); m.p. 125 ºC (decomp.); ^1H NMR (400 MHz, $CDCl_3$) δ 7.74 (d, *J* = 8.7 Hz, 2H), 7.40 (d, *J* = 8.7 Hz, 2H), 7.28–7.25 (m, 3H), 7.21–7.16 (m, 4H), 6.95 (t, *J* = 7.2 Hz, 1H), 6.69 (d, *J* = 7.2 Hz, 2H), 5.88 (d, *J* = 11.2 Hz, 1H), 5.69 (d, *J* = 11.2 Hz, 1H), 5.58 (d, *J* = 6.4 Hz, 1H), 4.90 (d, *J* = 6.4 Hz, 1H), 4.46 (d, *J* = 11.9 Hz, 1H), 4.41 (d, *J* = 11.9 Hz, 1H), 1.56 (s, 3H); ^{13}C NMR (100 MHz, $CDCl_3$) δ 172.3, 146.5, 138.0, 136.2, 135.6, 132.8, 132.0 (2C), 129.4 (2C), 129.3, 128.5 (2C), 128.3 (2C), 128.2, 127.7 (2C), 126.3, 124.3, 122.5, 115.6

(2C), 81.9, 66.9, 64.4, 55.0, 26.4; IR (ATR) ν 3383, 3060, 3032, 2921, 2853, 2363, 2353, 2332, 1742, 1672 cm^{-1}; HRMS (ESI-TOF) [M + Na]$^+$ calcd for $C_{28}H_{23}BrN_2NaO_4S^+$ m/z 585.0454, found 585.0460.

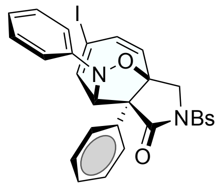

2-((4-Bromophenyl)sulfonyl)-6-iodo-8a,9-diphenyl-2,3,8,8a-tetrahydro-1*H*-3a,8-(epoxyimino)cyclohepta[*c*]pyrrol-1-one ((±)-3r)

Prepared according to the general procedure A using **2r** (50.3 mg, 0.1 mmol) and isolated as yellow solid (35.4 mg, 58% yield): TLC R_f = 0.15 (*n*-hexane/EtOAc, 3/1, v/v); m.p. 95 ºC (decomp.); ^{1}H NMR (400 MHz, $CDCl_3$) δ 7.74 (d, *J* = 8.7 Hz, 2H), 7.39 (d, *J* = 8.7 Hz, 2H), 7.34–7.28 (m, 3H), 7.27–7.23 (m, 2H), 7.15–7.13 (m, 2H), 7.03 (t, *J* = 7.3 Hz, 1H), 6.74 (d, *J* = 8.2 Hz, 2H), 6.49 (d, *J* = 6.9 Hz, 1H), 6.18 (d, *J* = 11.0 Hz, 1H), 5.60 (d, *J* = 11.0 Hz, 1H), 4.77 (d, *J* = 6.9 Hz, 1H), 4.50 (d, *J* = 12.4 Hz, 1H), 4.43 (d, *J* = 12.4 Hz, 1H); ^{13}C NMR (100 MHz, $CDCl_3$) δ 171.6, 145.0, 141.0, 138.6, 135.9, 132.0 (2C), 131.9, 129.5, 129.3 (2C), 128.8 (2C), 128.7 (3C), 127.6, 127.4 (2C), 123.5, 116.1 (2C), 97.7, 81.6, 69.7, 64.7, 55.4; IR (ATR) ν 3032, 1597, 1574, 1491, 1390, 1281, 1248, 1230,

1088, 1068 cm^{-1}; HRMS (ESI-TOF) [M + Na]$^+$ calcd for $C_{27}H_{20}BrIN_2NaO_4S^+$ m/z 696.9264, found 696.9260.

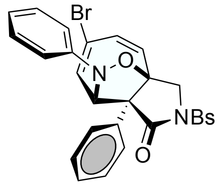

6-Bromo-2-((4-bromophenyl)sulfonyl)-8a,9-diphenyl-2,3,8,8a-tetrahydro-1*H*-3a,8-(epoxyimino)cyclohepta[*c*]pyrrol-1-one ((±)-3s)

Prepared according to the general procedure A using **2e** (52.1 mg, 0.1 mmol) and isolated as brown solid (41.5 mg, 66% yield): TLC R_f = 0.3 (*n*-hexane/EtOAc, 3/1, v/v); m.p. 75 ºC (decomp.); ^{1}H NMR (600 MHz, $CDCl_3$) δ 7.74 (d, *J* = 9.0 Hz, 2H), 7.38 (d, *J* = 9.0 Hz, 2H), 7.34-7.29 (m, 3H), 7.25 (dd, *J* = 9.0, 7.6 Hz, 2H), 7.14 (dd, *J* = 8.3, 1.4 Hz, 2H), 7.03 (dd, *J* = 7.6, 6.9 Hz, 1H), 6.75 (d, *J* = 7.6 Hz, 2H), 6.22 (d, *J* = 6.9 Hz, 1H), 6.00 (dd, *J* = 11.0, 1.4 Hz, 1H), 5.82 (d, *J* = 11.0 Hz, 1H), 4.90 (d, *J* = 7.6 Hz, 1H), 4.52 (d, *J* = 12.4 Hz, 1H), 4.44 (d, *J* = 12.4 Hz, 1H); ^{13}C NMR (100 MHz, $CDCl_3$) δ 171.6, 144.9, 136.6, 135.9, 132.0 (2C), 131.8, 130.2, 129.5, 129.3 (2C), 128.8 (2C), 128.7 (3C), 128.3, 127.5 (2C), 123.7, 123.5, 116.2 (2C), 81.5, 67.8, 64.7, 55.6; IR (ATR) ν 3062, 1743, 1672, 1628, 1595, 1574, 1491, 1471, 1390, 1367 cm^{-1}; HRMS (ESI-TOF) [M + Na]$^+$ calcd for $C_{27}H_{20}Br_2N_2NaO_4S^+$ m/z 648.9403, found 648.9397.

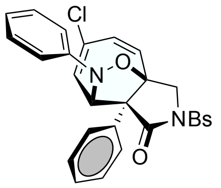

2-((4-Bromophenyl)sulfonyl)-6-chloro-8a,9-diphenyl-2,3,8,8a-tetrahydro-1*H*-3a,8-(epoxyimino)cyclohepta[*c*]pyrrol-1-one ((±)-3t)

Prepared according to the general procedure A using **2t** (47.7 mg, 0.1 mmol) and isolated as brown solid (36.2 mg, 62% yield): TLC R_f = 0.3 (*n*-hexane/EtOAc, 3/1, v/v); m.p. 100 ºC (decomp.); ^{1}H NMR (400 MHz, $CDCl_3$) δ 7.74 (d, *J* = 8.7 Hz, 2H), 7.37 (d, *J* = 8.7 Hz, 2H), 7.30 (d, *J* = 7.3 Hz, 3H), 7.23 (d, *J* = 8.2 Hz, 2H), 7.15–7.12 (m, 2H), 7.02 (dt, *J* = 7.3, 1.2 Hz, 1H), 6.75 (d, *J* = 7.3 Hz, 2H), 5.98 (dd, *J* = 7.3, 1.2 Hz, 1H), 5.93 (d, *J* = 11.5 Hz, 1H), 5.83 (dd, *J* = 11.5, 1.2 Hz, 1H), 4.96 (d, *J* = 7.3 Hz, 1H), 4.53 (d, *J* = 12.0 Hz, 1H), 4.45 (d, *J* = 12.0 Hz, 1H); ^{13}C NMR (100 MHz, $CDCl_3$) δ 171.6, 145.0, 136.0, 134.7, 134.6, 132.0 (2C), 131.9, 129.5, 129.3 (2C), 128.8 (2C), 128.7 (3C), 128.6, 127.5 (2C), 126.1, 123.5, 116.2 (2C), 81.5, 66.6, 64.6, 55.7; IR (ATR) ν 3032, 1595, 1574, 1491, 1471, 1390, 1317, 1278, 1088, 968 cm^{-1}; HRMS (ESI-TOF) $[M + Na]^+$ calcd for $C_{27}H_{20}BrClN_2NaO_4S^+$ m/z 604.9908, found 604.9911.

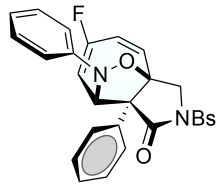

2-((4-Bromophenyl)sulfonyl)-6-fluoro-8a,9-diphenyl-2,3,8,8a-tetrahydro-1*H*-3a,8-(epoxyimino)cyclohepta[*c*]pyrrol-1-one ((±)-3u)

Prepared according to the general procedure A using **2u** (46.0 mg, 0.1 mmol) and isolated as yellow solid (40.2 mg, 71% yield): TLC R_f = 0.25 (*n*-hexane/EtOAc, 3/1, v/v), m.p. 100 ºC (decomp.); ^{1}H NMR (400 MHz, $CDCl_3$) δ 7.74 (d, *J* = 8.7 Hz, 2H), 7.35 (d, *J* = 8.7 Hz, 2H), 7.31–7.23 (m, 5H), 7.15–7.13 (m, 2H), 7.02 (t, *J* = 7.3 Hz, 1H), 6.75 (d, *J* = 7.8 Hz, 2H), 6.06 (dd, *J* = 11.5, 4.1 Hz, 1H), 5.79–5.72 (m, 1H), 5.45 (ddd, *J* = 20.6, 7.3, 2.3 Hz, 1H), 4.99 (dd, *J* = 7.3, 3.2 Hz, 1H), 4.56 (d, *J* = 12.0 Hz, 1H), 4.46 (d, *J* = 12.0 Hz, 1H); ^{13}C NMR (100 MHz, $CDCl_3$) δ 171.8, 159.7 (d, *J* = 244.4 Hz, 1C), 144.8, 136.0, 132.0 (2C), 130.1, 130.0, 129.4, 129.3 (2C), 129.0, 128.7 (2C), 128.6 (2C), 128.5, 127.7 (2C), 123.3, 116.3 (2C), 106.3 (d, *J* = 28.8 Hz, 1C), 81.4, 64.6, 64.0 (d, *J* = 11.5 Hz, 1C), 56.1; ^{19}F NMR (376 MHz, $CDCl_3$) δ −91.5; IR (ATR) ν 3062, 1651, 1624, 1595, 1574, 1491, 1471, 1414, 1390, 1323 cm^{-1}; HRMS (ESI-TOF) [M + Na]$^+$ calcd for $C_{27}H_{20}BrFN_2NaO_4S^+$ m/z 589.0203, found 589.0217.

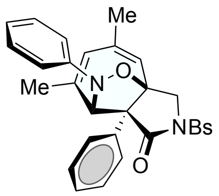

2-((4-Bromophenyl)sulfonyl)-5,7-dimethyl-8a,9-diphenyl-2,3,8,8a-tetrahydro-1*H*-3a,8-(epoxyimino)cyclohepta[*c*]pyrrol-1-one ((±)-3v)

Prepared according to the general procedure A using **1v** (47.1 mg, 0.1 mmol) and isolated as yellow solid (19.9 mg, 34% yield): TLC R_f = 0.5 (*n*-hexane/EtOAc, 5/1, v/v); m.p. 84 ºC (decomp.); ^{1}H NMR (600 MHz, $CDCl_3$) δ 7.73 (d, *J* = 8.3 Hz, 2H), 7.44 (d, *J* = 8.3 Hz, 2H), 7.30–7.27 (m, 3H), 7.22–7.21 (m, 2H), 7.14 (t, *J* = 7.6 Hz, 2H), 6.95 (t, *J* = 7.6 Hz, 1H), 6.68 (d, *J* = 7.6 Hz, 2H), 5.65 (s, 1H), 5.40 (s, 1H), 4.72 (s, 1H), 4.39 (d, *J* = 11.7 Hz, 1H), 4.34 (d, *J* = 11.7 Hz, 1H), 1.67 (s, 3H), 1.66 (s, 3H); ^{13}C NMR (150 MHz, $CDCl_3$) δ 172.2, 147.7, 139.7, 138.1, 136.2, 132.7, 132.1 (2C), 129.8, 129.5 (2C), 129.3, 128.6 (2C), 128.4 (2C), 128.3, 127.2 (2C), 122.8, 120.0, 115.4 (2C), 82.3, 73.1, 64.0, 54.5, 27.5, 25.7; IR (ATR) ν 2972, 2357, 2336, 1744, 1651, 1597, 1574, 1488, 1470, 1447 cm^{-1}; HRMS (ESI-TOF) $[M + Na]^+$ calcd for $C_{29}H_{25}BrN_2NaO_4S^+$ m/z 599.0611, found 599.0630.

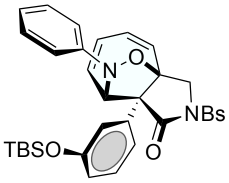

2-((4-Bromophenyl)sulfonyl)-8a-(3-((tert-butyldimethylsilyl)oxy)phenyl)-9-phenyl-2,3,8,8a-tetrahydro-1*H*-3a,8-(epoxyimino)cyclohepta[c]pyrrol-1-one ((±)-3w)

Prepared according to the general procedure A using **2w** (57.3 mg, 0.1 mmol) and isolated as white solid (54.0 mg, 79% yield): TLC R_f = 0.2 (*n*-hexane/EtOAc, 5/1, v/v); m.p. 144 ºC (decomp.); ^{1}H NMR (600 MHz, $CDCl_3$) δ 7.74 (d, *J* = 8.3 Hz, 2H), 7.38 (d, *J* = 8.3 Hz, 2H), 7.21 (t, *J* = 7.6 Hz, 2H), 7.13 (t, *J* = 7.6 Hz, 1H), 6.97 (t, *J* = 6.9 Hz, 1H), 6.78–6.74 (m, 4H), 6.67 (s, 1H), 5.87 (d, *J* = 10.3 Hz, 1H), 5.82–5.79 (m, 1H), 5.75 (t, *J* = 4.1 Hz, 2H), 4.94 (t, *J* = 4.1 Hz, 1H), 4.48 (d, *J* = 12.4 Hz, 1H), 4.39 (d, *J* = 12.4 Hz, 1H), 0.96 (s, 9H), 0.17 (s, 6H); ^{13}C NMR (150 MHz, $CDCl_3$) δ 172.1, 155.4, 145.9, 136.2, 133.8, 132.0 (2C), 131.4, 130.2, 129.5, 129.3 (3C), 129.2, 128.6 (2C), 127.4, 122.7, 120.8, 120.3, 120.1, 115.8 (2C), 82.3, 67.4, 64.1, 55.5, 25.7 (3C), 18.2, −4.4 (2C); IR (ATR) ν 3093, 3063, 3034, 2954, 2929, 2897, 2857, 1743, 1598, 1583 cm^{-1}; HRMS (ESI-TOF) $[M + Na]^+$ calcd for $C_{33}H_{35}BrN_2NaO_5SSi^+$ m/z 701.1112, found 701.1111.

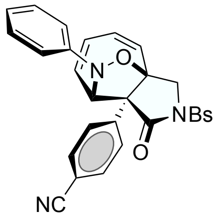

4-(2-((4-Bromophenyl)sulfonyl)-1-oxo-9-phenyl-2,3-dihydro-1*H*-3a,8-(epoxyimino)cyclohepta[c]pyrrol-8a(8*H*)-yl)benzonitrile ((±)-3x)

Prepared according to the general procedure A using **2x** (46.7 mg, 0.1 mmol) and isolated as yellow solid (38.5 mg, 67% yield): TLC R_f = 0.2 (*n*-hexane/EtOAc, 4/1, v/v); m.p. 152 ºC (decomp.); ^{1}H NMR (600 MHz, $CDCl_3$) δ 7.74 (d, *J* = 8.3 Hz, 2H), 7.59 (d, *J* = 8.3 Hz, 2H), 7.39 (d, *J* = 9.0 Hz, 2H), 7.34 (d, *J* = 9.0 Hz, 2H), 7.23 (t, *J* = 7.6 Hz, 2H), 7.00 (t, *J* = 7.6 Hz, 1H), 6.74 (d, *J* = 7.6 Hz, 2H), 5.93 (d, *J* = 10.3 Hz, 1H), 5.83–5.80 (m, 1H), 5.79–5.73 (m, 2H), 4.99 (d, *J* = 6.9 Hz, 1H), 4.53 (d, 12.4 Hz, 1H), 4.44 (d, 12.4 Hz, 1H); ^{13}C NMR (150 MHz, $CDCl_3$) δ 171.2, 145.3, 137.7, 135.9, 132.1 (2C), 132.0 (2C), 131.9, 130.8, 129.6, 129.3 (2C), 129.1, 128.7 (2C), 128.6 (2C), 127.1, 123.2, 118.0, 115.9 (2C), 112.4, 82.0, 67.0, 64.6, 55.7; IR (ATR) ν 3093, 3063, 3036, 2956, 2925, 2855, 2230, 1742, 1637, 1596 cm^{-1}; HRMS (ESI-TOF) $[M + Na]^+$ calcd for $C_{28}H_{20}BrN_3NaO_4S^+$ m/z 596.0250, found 596.0266.

4. Synthesis and Characterization of Cycloadducts with Nitroso Compounds (*Conditions C*)

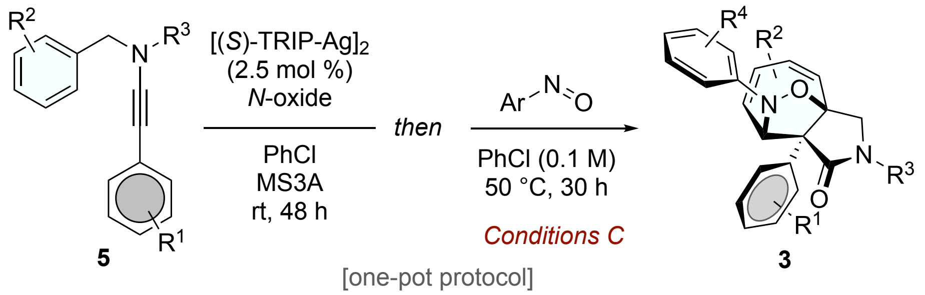

A pre-dried 10-mL test tube equipped with a magnetic stir bar was covered with aluminum foil to avoid light exposure. The test tube was charged with ynamide compound **5** (0.1 mmol, 1 equiv.), 8-methylquinoline *N*-oxide (0.2 mmol, 2 equiv.), [(*S*)-TRIP-Ag]$_2$ (0.025 mmol, 2.5 mol %) and MS3A (100 mg, 1 g/mmol), which were subsequently dissolved partially in dry PhCl (1 mL, 0.1 M) under an argon gas atmosphere. The reaction mixture was stirred for 48 h at room temperature, and then the aluminum foil was removed. Nitrosobenzene (0.3 mmol, 3 equiv.) was added to the reaction mixture, and the reaction mixture was stirred at 50 °C for 30 h. Subsequently, the reaction mixture was passed through Celite to remove MS3A. After the solvent was evaporated in *vacuo*, the resulting residue was purified by flash chromatography (*n*-hexane/EtOAc = 5/1, v/v) to afford desired product **3**.

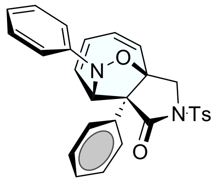

(3a*R*,8*S*,8a*S*)-8a,9-Diphenyl-2-tosyl-2,3,8,8a-tetrahydro-1*H*-3a,8-(epoxyimino)cyclohepta[*c*]pyrrol-1-one ((+)-3a)

Prepared according to the general procedure C using **5a** (36.1 mg, 0.1 mmol) and isolated (33.0 mg, 68% yield, 90 : 10 er). ^{1}H NMR, ^{13}C NMR, IR, and MS data were identical to the racemic compound.; $[\alpha]^{25}_{D}$ +107.4 (c 1.5, $CHCl_3$). The enantiomeric ratio was determined by analytical chiral HPLC. Retention time: 12.3 min (minor isomer), 14.1 min (major isomer), IH-3 column, 60/40 *n*-hexane/DCM, 0.5 mL/min, 254 nm.

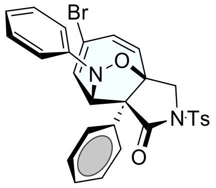

(3a*R*,8*S*,8a*S*)-6-Bromo-8a,9-diphenyl-2-tosyl-2,3,8,8a-tetrahydro-1*H*-3a,8-(epoxyimino)cyclohepta[*c*]pyrrol-1-one ((+)-3b)

Prepared according to the general procedure C using **5b** (44.0 mg, 0.1 mmol) and isolated (38.3 mg, 68% yield, 90 : 10 er). ^{1}H NMR, ^{13}C NMR, IR, and MS data were identical to the racemic compound.; $[\alpha]^{25}_{D}$ +206.7 (c 0.8, $CHCl_3$). The enantiomeric ratio was

determined by analytical chiral HPLC. Retention time: 14.2 min (minor isomer), 17.2 min (major isomer), IH-3 column, 60/40 *n*-hexane/DCM, 0.5 mL/min, 254 nm.

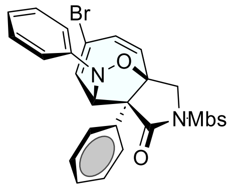

(3a*R*,8*S*,8a*S*)-6-Bromo-2-((4-methoxyphenyl)sulfonyl)-8a,9-diphenyl-2,3,8,8a-tetrahydro-1*H*-3a,8-(epoxyimino)cyclohepta[*c*]pyrrol-1-one ((+)-3c)

Prepared according to the general procedure C using **5c** (45.6 mg, 0.1 mmol) and isolated (40.6 mg, 70% yield, 93 : 7 er). ^{1}H NMR, ^{13}C NMR, IR, and MS data were identical to the racemic compound.; $[\alpha]^{25}_{D}$ +138.0 (c 0.8, $CHCl_3$). The enantiomeric ratio was determined by analytical chiral HPLC. Retention time: 14.9 min (minor isomer), 17.9 min (major isomer), IH-3 column, 60/40 *n*-hexane/DCM, 0.5 mL/min, 254 nm.

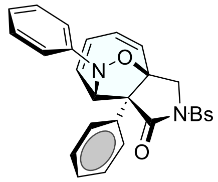

(3a*R*,8*S*,8a*S*)-2-((4-Bromophenyl)sulfonyl)-8a,9-diphenyl-2,3,8,8a-tetrahydro-1*H*-3a,8-(epoxyimino)cyclohepta[*c*]pyrrol-1-one ((+)-3d)

Prepared according to the general procedure C using **5d** (42.6 mg, 0.1 mmol) and isolated (40.1 mg, 73% yield, 94 : 6 er). ^{1}H NMR, ^{13}C NMR, IR, and MS data were identical to the racemic compound.; $[\alpha]^{25}_{D}$ +313.0 (c 1.2, $CHCl_3$). The enantiomeric ratio was determined by analytical chiral HPLC. Retention time: 12.0 min (minor isomer), 13.3 min (major isomer), IH-3 column, 60/40 *n*-hexane/DCM, 0.5 mL/min, 254 nm.

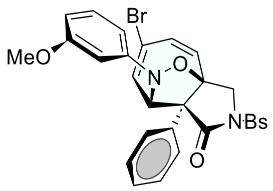

(3a*R*,8*S*,8a*S*)-6-Bromo-2-((4-bromophenyl)sulfonyl)-9-(3-methoxyphenyl)-8a-phenyl-2,3,8,8a-tetrahydro-1*H*-3a,8-(epoxyimino)cyclohepta[*c*]pyrrol-1-one ((+)-3e)

Prepared according to the general procedure C using **5e** (50.5 mg, 0.1 mmol), methoxy 3-nitrosobenzoate (68.6 mg, 0.3 mmol) and isolated (45.7 mg, 69% yield, 95 : 5 er). ^{1}H NMR, ^{13}C NMR, IR, and MS data were identical to the racemic compound.; $[\alpha]^{25}_{D}$ +241.3 (c 1.6, $CHCl_3$). The enantiomeric ratio was determined by analytical chiral HPLC. Retention time: 19.2 min (minor isomer), 21.7 min (major isomer), IH-3 column, 60/40 *n*-hexane/DCM, 0.5 mL/min, 254 nm.

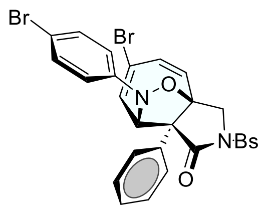

(3a*R*,8*S*,8a*S*)-6-Bromo-9-(4-bromophenyl)-2-((4-bromophenyl)sulfonyl)-8a-phenyl-2,3,8,8a-tetrahydro-1*H*-3a,8-(epoxyimino)cyclohepta[*c*]pyrrol-1-one ((+)-3f)

Prepared according to the general procedure C using **5e** (50.5 mg, 0.1 mmol), 1-bromo-4-nitrosobenzene (55.8 mg, 0.3 mmol) and isolated (49.5 mg, 70% yield, 96 : 4 er). ^{1}H NMR, ^{13}C NMR, IR, and MS data were identical to the racemic compound.; $[\alpha]^{25}_{D}$ +236.0 (c 2.1, $CHCl_3$). The enantiomeric ratio was determined by analytical chiral HPLC. Retention time: 15.4 min (minor isomer), 20.2 min (major isomer), IH-3 column, 60/40 *n*-hexane/DCM, 0.5 mL/min, 254 nm.

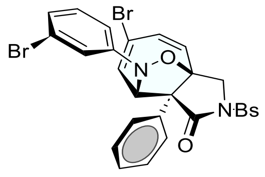

(3a*R*,8*S*,8a*S*)-6-Bromo-9-(3-bromophenyl)-2-((4-bromophenyl)sulfonyl)-8a-phenyl-2,3,8,8a-tetrahydro-1*H*-3a,8-(epoxyimino)cyclohepta[*c*]pyrrol-1-one ((+)-3g)

Prepared according to the general procedure C using **5e** (50.5 mg, 0.1 mmol), 1-bromo-3-nitrosobenzene (55.8 mg, 0.3 mmol) and isolated (50.9 mg, 72% yield, 96 : 4 er). ^{1}H NMR, ^{13}C NMR, IR, and MS data were identical to the racemic compound.; $[\alpha]^{25}_{D}$ +200.3

(c 2.5, $CHCl_3$). The enantiomeric ratio was determined by analytical chiral HPLC. Retention time: 14.9 min (minor isomer), 17.9 min (major isomer), IH-3 column, 60/40 *n*-hexane/DCM, 0.5 mL/min, 254 nm.

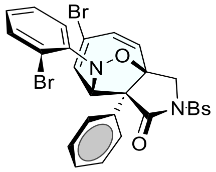

(3a*R*,8*S*,8a*S*)-6-Bromo-9-(2-bromophenyl)-2-((4-bromophenyl)sulfonyl)-8a-phenyl-2,3,8,8a-tetrahydro-1*H*-3a,8-(epoxyimino)cyclohepta[*c*]pyrrol-1-one ((+)-3h)

Prepared according to the general procedure C using **5e** (50.5 mg, 0.1 mmol), 1-bromo-2-nitrosobenzene (55.8 mg, 0.3 mmol) and isolated (50.2 mg, 71% yield, 96 : 4 er). ^{1}H NMR, ^{13}C NMR, IR, and MS data were identical to the racemic compound.; $[\alpha]^{25}_{D}$ +229.2 (c 2.2, $CHCl_3$). The enantiomeric ratio was determined by analytical chiral HPLC. Retention time: 15.0 min (major isomer), 16.7 min (minor isomer), IH-3 column, 60/40 *n*-hexane/DCM, 0.5 mL/min, 254 nm.

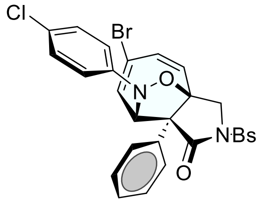

(3a*R*,8*S*,8a*S*)-6-Bromo-2-((4-bromophenyl)sulfonyl)-9-(4-chlorophenyl)-8a-phenyl-2,3,8,8a-tetrahydro-1*H*-3a,8-(epoxyimino)cyclohepta[*c*]pyrrol-1-one ((+)-3i)

Prepared according to the general procedure C using **5e** (50.5 mg, 0.1 mmol), 1-chloro-4-nitrosobenzene (42.5 mg, 0.3 mmol) and isolated (45.1 mg, 68% yield, 96 : 4 er). ^{1}H NMR, ^{13}C NMR, IR, and MS data were identical to the racemic compound.; $[\alpha]^{25}_{D}$ +222.0 (c 1.9, $CHCl_3$). The enantiomeric ratio was determined by analytical chiral HPLC. Retention time: 15.4 min (minor isomer), 20.5 min (major isomer), IH-3 column, 60/40 *n*-hexane/DCM, 0.5 mL/min, 254 nm.

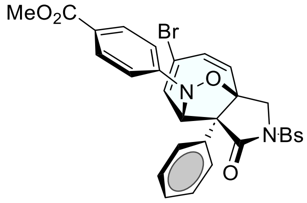

Methyl 4-((3a*R*,8*S*,8a*S*)-6-bromo-2-((4-bromophenyl)sulfonyl)-8a-phenyl-1-oxo-2,3,8,8a-tetrahydro-1*H*-3a,8-(epoxyimino)cyclohepta[*c*]pyrrol-9-yl)benzoate ((+)-3j)

Prepared according to the general procedure C using **5e** (50.5 mg, 0.1 mmol), methyl 4-nitrosobenzoate (49.5 mg, 0.3 mmol) and isolated (53.5 mg, 78% yield, 92 : 8 er). ^{1}H NMR, ^{13}C NMR, IR, and MS data were identical to the racemic compound.; $[\alpha]^{25}_{D}$ +102.4 (c 1.9, $CHCl_3$). The enantiomeric ratio was determined by analytical chiral HPLC.

Retention time: 19.1 min (minor isomer), 22.5 min (major isomer), IH-3 column, 60/40 *n*-hexane/DCM, 0.5 mL/min, 254 nm.

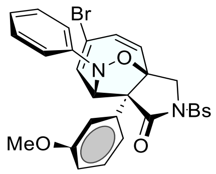

(3a*R*,8*S*,8a*S*)-6-Bromo-2-((4-bromophenyl)sulfonyl)-8a-(3-methoxyphenyl)-9-phenyl-2,3,8,8a-tetrahydro-1*H*-3a,8-(epoxyimino)cyclohepta[*c*]pyrrol-1-one ((+)-3k)

Prepared according to the general procedure C using **5k** (53.5 mg, 0.1 mmol) and isolated (41.5 mg, 63% yield, 96 : 4 er). ^{1}H NMR, ^{13}C NMR, IR, and MS data were identical to the racemic compound.; $[\alpha]^{25}_{D}$ +147.1 (c 2.3, $CHCl_3$). The enantiomeric ratio was determined by analytical chiral HPLC. Retention time: 14.0 min (minor isomer), 25.4 min (major isomer), IH-3 column, 60/40 *n*-hexane/DCM, 0.5 mL/min, 254 nm.

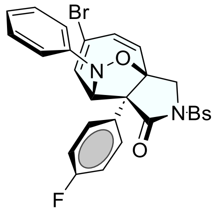

(3a*R*,8*S*,8a*S*)-6-Bromo-2-((4-bromophenyl)sulfonyl)-8a-(4-fluorophenyl)-9-phenyl-2,3,8,8a-tetrahydro-1*H*-3a,8-(epoxyimino)cyclohepta[*c*]pyrrol-1-one ((+)-3l)

Prepared according to the general procedure C using **5l** (52.3 mg, 0.1 mmol) and isolated (30.4 mg, 47% yield, 95 : 5 er). ^{1}H NMR, ^{13}C NMR, ^{19}F NMR, IR, and MS data were identical to the racemic compound.; $[\alpha]^{25}_{D}$ +206.1 (c 0.9, $CHCl_3$). The enantiomeric ratio was determined by analytical chiral HPLC. Retention time: 15.4 min (minor isomer), 16.5 min (major isomer), IH-3 column, 60/40 *n*-hexane/DCM, 0.5 mL/min, 254 nm.

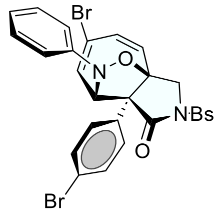

(3a*R*,8*S*,8a*S*)-6-bromo-8a-(4-bromophenyl)-2-((4-bromophenyl)sulfonyl)-9-phenyl-2,3,8,8a-tetrahydro-1*H*-3a,8-(epoxyimino)cyclohepta[*c*]pyrrol-1-one ((+)-3m)

Prepared according to the general procedure C using **5m** (58.4 mg, 0.1 mmol) and isolated (41.7 mg, 59% yield, 95 : 5 er). ^{1}H NMR, ^{13}C NMR, IR, and MS data were identical to the racemic compound.; $[\alpha]^{25}_{D}$ +157.4 (c 1.0, $CHCl_3$). The enantiomeric ratio was determined by analytical chiral HPLC. Retention time: 13.7 min (major isomer), 18.7 min (minor isomer), IH-3 column, 60/40 *n*-hexane/DCM, 0.5 mL/min, 254 nm.

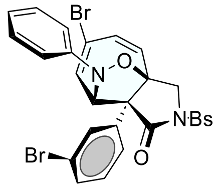

(3a*R*,8*S*,8a*S*)-6-Bromo-8a-(3-bromophenyl)-2-((4-bromophenyl)sulfonyl)-9-phenyl-2,3,8,8a-tetrahydro-1*H*-3a,8-(epoxyimino)cyclohepta[*c*]pyrrol-1-one ((+)-3n)

Prepared according to the general procedure C using **5n** (58.4 mg, 0.1 mmol) and isolated (25.5 mg, 36% yield, 93 : 7 er). ^{1}H NMR, ^{13}C NMR, IR, and MS data were identical to the racemic compound.; $[\alpha]^{25}_D$ +149.6 (c 0.9, $CHCl_3$). The enantiomeric ratio was determined by analytical chiral HPLC. Retention time: 15.0 min (minor isomer), 18.0 min (major isomer), IH-3 column, 60/40 *n*-hexane/DCM, 0.5 mL/min, 254 nm.

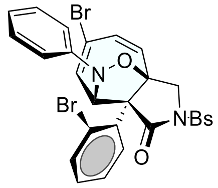

(3a*R*,8*S*,8a*S*)-6-Bromo-8a-(2-bromophenyl)-2-((4-bromophenyl)sulfonyl)-9-phenyl-2,3,8,8a-tetrahydro-1*H*-3a,8-(epoxyimino)cyclohepta[*c*]pyrrol-1-one ((+)-3o)

Prepared according to the general procedure C using **5o** (58.4 mg, 0.1 mmol) and isolated (42.4 mg, 60% yield, 85 : 15 er). ^{1}H NMR, ^{13}C NMR, IR, and MS data were identical to the racemic compound.; $[\alpha]^{25}_D$ +130.7 (c 1.1, $CHCl_3$). The enantiomeric ratio was

determined by analytical chiral HPLC. Retention time: 13.0 min (minor isomer), 15.1 min (major isomer), IH-3 column, 60/40 *n*-hexane/DCM, 0.5 mL/min, 254 nm.

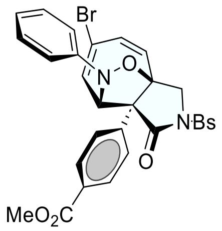

Methyl 4-((3a*R*,8*S*,8a*S*)-6-bromo-2-((4-bromophenyl)sulfonyl)-1-oxo-9-phenyl-2,3-dihydro-1*H*-3a,8-(epoxyimino)cyclohepta[*c*]pyrrol-8a(8*H*)-yl)benzoate ((+)-3p)

Prepared according to the general procedure C using **5p** (56.3 mg, 0.1 mmol) and isolated (41.5 mg, 33% yield, 96 : 4 er). ^{1}H NMR, ^{13}C NMR, IR, and MS data were identical to the racemic compound.; $[\alpha]^{25}_{D}$ +138.9 (c 1.1, $CHCl_3$). The enantiomeric ratio was determined by analytical chiral HPLC. Retention time: 39.1 min (minor isomer), 44.2 min (major isomer), IH-3 column, 70/30 *n*-hexane/DCM, 0.5 mL/min, 254 nm.

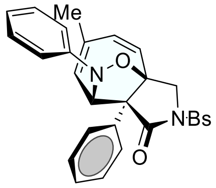

(3a*R*,8*S*,8a*S*)-2-((4-Bromophenyl)sulfonyl)-6-methyl-8a,9-diphenyl-2,3,8,8a-tetrahydro-1*H*-3a,8-(epoxyimino)cyclohepta[*c*]pyrrol-1-one ((+)-3q)

Prepared according to the general procedure C using **5q** (44.0 mg, 0.1 mmol) and isolated (18.0 mg, 32% yield, 95 : 5 er). ^{1}H NMR, ^{13}C NMR, IR, and MS data were identical to the racemic compound.; $[\alpha]^{25}_{D}$ +175.8 (c 0.67, $CHCl_3$). The enantiomeric ratio was determined by analytical chiral HPLC. Retention time: 18.8 min (minor isomer), 21.7 min (major isomer), IH-3 column, 70/30 *n*-hexane/DCM, 0.5 mL/min, 254 nm.

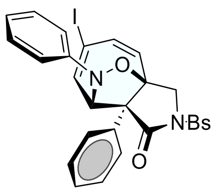

(3a*R*,8*S*,8a*S*)-2-((4-Bromophenyl)sulfonyl)-6-iodo-8a,9-diphenyl-2,3,8,8a-tetrahydro-1*H*-3a,8-(epoxyimino)cyclohepta[*c*]pyrrol-1-one ((+)-3r)

Prepared according to the general procedure C using **5r** (48.7 mg, 0.1 mmol) and isolated (34.8 mg, 57% yield, 96 : 4 er). ^{1}H NMR, ^{13}C NMR, IR, and MS data were identical to the racemic compound.; $[\alpha]^{25}_{D}$ +104.4 (c 2.5, $CHCl_3$). The enantiomeric ratio was determined by analytical chiral HPLC. Retention time: 15.2 min (minor isomer), 17.0 min (major isomer), IH-3 column, 60/40 *n*-hexane/DCM, 0.5 mL/min, 254 nm.

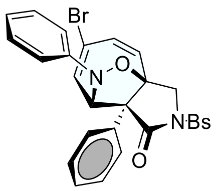

(3a*R*,8*S*,8a*S*)-6-Bromo-2-((4-bromophenyl)sulfonyl)-8a,9-diphenyl-2,3,8,8a-tetrahydro-1*H*-3a,8-(epoxyimino)cyclohepta[*c*]pyrrol-1-one ((+)-3s)

Prepared according to the general procedure C using **5e** (50.5 mg, 0.1 mmol) and isolated (44.0 mg, 70% yield, 96 : 4 er). ^{1}H NMR, ^{13}C NMR, IR, and MS data were identical to the racemic compound.; $[\alpha]^{25}_{D}$ +179.2 (c 1.3, $CHCl_3$). The enantiomeric ratio was determined by analytical chiral HPLC. Retention time: 14.5 min (minor isomer), 16.4 min (major isomer), IH-3 column, 60/40 *n*-hexane/DCM, 0.5 mL/min, 254 nm.

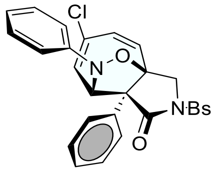

(3a*R*,8*S*,8a*S*)-2-((4-Bromophenyl)sulfonyl)-6-chloro-8a,9-diphenyl-2,3,8,8a-tetrahydro-1*H*-3a,8-(epoxyimino)cyclohepta[*c*]pyrrol-1-one ((+)-3t)

Prepared according to the general procedure C using **5t** (46.1 mg, 0.1 mmol) and isolated (38.5 mg, 66% yield, 96 : 4 er). ^{1}H NMR, ^{13}C NMR, IR, and MS data were identical to the racemic compound.; $[\alpha]^{25}_{D}$ +164.2 (c 2.0, $CHCl_3$). The enantiomeric ratio was determined by analytical chiral HPLC. Retention time: 14.2 min (minor isomer), 16.0 min (major isomer), IH-3 column, 60/40 *n*-hexane/DCM, 0.5 mL/min, 254 nm.

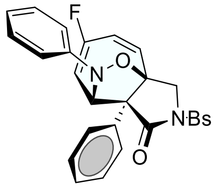

(3a*R*,8*S*,8a*S*)-2-((4-Bromophenyl)sulfonyl)-6-fluoro-8a,9-diphenyl-2,3,8,8a-tetrahydro-1*H*-3a,8-(epoxyimino)cyclohepta[*c*]pyrrol-1-one ((+)-3u)

Prepared according to the general procedure C using **5u** (44.0 mg, 0.1 mmol) and isolated (41.4 mg, 73% yield, 95 : 5 er). ^{1}H NMR, ^{13}C NMR, ^{19}F NMR, IR, and MS data were identical to the racemic compound.; $[\alpha]^{25}_{D}$ +184.1 (c 1.4, $CHCl_3$). The enantiomeric ratio was determined by analytical chiral HPLC. Retention time: 14.8 min (minor isomer), 15.8 min (major isomer), IH-3 column, 60/40 *n*-hexane/DCM, 0.5 mL/min, 254 nm.

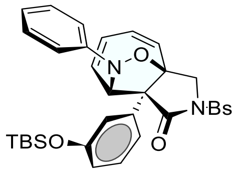

(3a*R*,8*S*,8a*S*)-2-((4-Bromophenyl)sulfonyl)-8a-(3-((tert-butyldimethylsilyl)oxy)phenyl)-9-phenyl-2,3,8,8a-tetrahydro-1*H*-3a,8-(epoxyimino)cyclohepta[c]pyrrol-1-one ((+)-3w)

Prepared according to the general procedure C using **5w** (55.7 mg, 0.1 mmol) and isolated (49.8 mg, 73% yield, 91 : 9 er). The reaction time in the first step to synthesize CHT/NCD is 72 h.: ^{1}H NMR, ^{13}C NMR, IR, and MS data were identical to the racemic compound.;

$[\alpha]^{25}_{D}$ +207.8 (c 2.5, $CHCl_3$). The enantiomeric ratio was determined by analytical chiral HPLC. Retention time: 22.2 min (minor isomer), 24.2 min (major isomer), IH-3 column, 80/20 *n*-hexane/DCM, 0.5 mL/min, 254 nm.

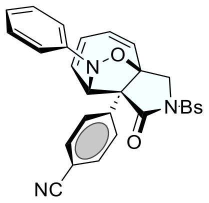

4-((3a*R*,8*S*,8a*S*)-2-((4-Bromophenyl)sulfonyl)-1-oxo-9-phenyl-2,3-dihydro-1*H*-3a,8-(epoxyimino)cyclohepta[c]pyrrol-8a(8*H*)-yl)benzonitrile ((+)-3x)

Prepared according to the general procedure C using **5x** (45.1 mg, 0.1 mmol) and isolated (37.4 mg, 65% yield, 89 : 11 er): ^{1}H NMR, ^{13}C NMR, IR, and MS data were identical to the racemic compound.; $[\alpha]^{25}_{D}$ +219.8 (c 1.8, $CHCl_3$). The enantiomeric ratio was determined by analytical chiral HPLC. Retention time: 11.5 min (minor isomer), 14.3 min (major isomer), IH-3 column, 60/40 *n*-hexane/DCM, 1.0 mL/min, 254 nm.

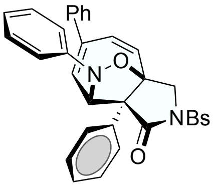

(3a*R*,8*S*,8a*S*)-2-((4-Bromophenyl)sulfonyl)-6,8a,9-triphenyl-2,3,8,8a-tetrahydro-1*H*-3a,8-(epoxyimino)cyclohepta[*c*]pyrrol-1-one ((+)-3y)

Prepared according to the general procedure C using **5y** (50.2 mg, 0.1 mmol) and isolated as yellow solid (35.4 mg, 57% yield, 96 : 4 er): TLC R_f = 0.3 (*n*-hexane/EtOAc, 5/1, v/v); ^{1}H NMR (600 MHz, $CDCl_3$) δ 7.79 (d, *J* = 8.2 Hz, 2H), 7.45 (d, *J* = 8.2 Hz, 2H), 7.29-7.27 (m, 5H), 7.20 (t, *J* = 7.6 Hz, 2H), 7.17-7.14 (m, 3H), 6.97 (t, *J* = 7.6 Hz, 1H), 6.84 (dd, *J* = 7.6, 2.1 Hz, 2H), 6.74 (d, *J* = 7.6 Hz, 2H), 6.11 (d, *J* = 11.0 Hz, 1H), 6.02 (d, *J* = 11.0 Hz, 1H), 5.83 (d, *J* = 6.9 Hz, 1H), 5.08 (d, *J* = 6.9 Hz, 1H), 5.00 (d, *J* = 11.7 Hz, 1H), 4.48 (d, *J* = 11.7 Hz, 1H); ^{13}C NMR (150 MHz, $CDCl_3$) δ 172.2, 146.6, 143.4, 143.2, 136.2, 134.6, 132.6, 132.1, 132.1, 129.4 (2C), 128.6, 128.6, 128.4 (3C), 128.2 (2C), 127.9, 127.6 (3C), 127.0, 126.4 (2C), 122.8, 115.8 (2C), 82.0, 67.6, 67.5, 64.2, 54.7; IR (ATR) ν 3058, 2374, 2351, 2325, 1744, 1596, 1574, 1490, 1471, 1447 cm^{-1}; HRMS (ESI-TOF) $[M + Na]^+$ calcd for $C_{33}H_{25}BrN_2NaO_4S^+$ m/z 647.0611, found 647.0638; $[\alpha]^{25}_D$ +170.9 (c 1.3, $CHCl_3$). The enantiomeric ratio was determined by analytical chiral HPLC. Retention time: 8.6 min (major isomer), 18.2 min (major isomer), IBN-3 column, 70/30 *n*-hexane/DCM, 1.0 mL/min, 254 nm.

The procedure for gram-scale cycloaddition reaction with nitrosobenzene

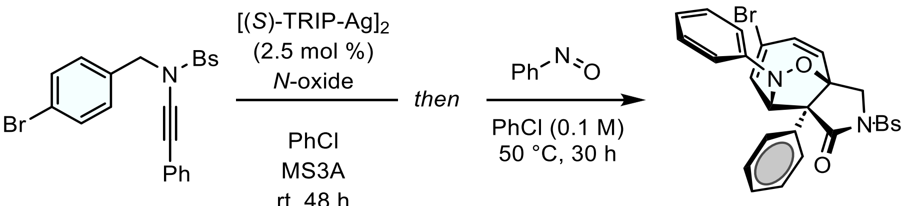

5e
1.26 g

3s
1.13 g
(72% yield, 96 : 4 er)

A pre-dried 50 mL flask equipped with a magnetic stir bar was covered with aluminum foil to avoid light exposure. The flask was charged with ynamide compound **5e** (2.5 mmol, 1 equiv.), 8-methylquinoline *N*-oxide (5 mmol, 2 equiv.), [(*S*)-TRIP-Ag]$_2$ (0.00625 mmol, 2.5 mol %) and MS3A (2.5 g, 1 g/mmol), which were subsequently dissolved partially in dry PhCl (25 mL, 0.1 M) under an argon gas atmosphere. The reaction mixture was stirred for 48 h at room temperature, and then the aluminum foil was removed. Nitrosobenzene (12.5 mmol, 5 equiv.) was added to the reaction mixture, and the reaction mixture was stirred at 50 °C for 30 h. Subsequently, the reaction mixture was passed through Celite to remove MS3A. After the solvent was evaporated in *vacuo*, the resulting residue was purified by flash chromatography (*n*-hexane/EtOAc = 5/1, v/v) to afford desired product **3s** (1131.0 mg, 72% yield, 96 : 4 er).

5. Synthesis and Characterization of Cycloadducts with Arynes (*Conditions B*)

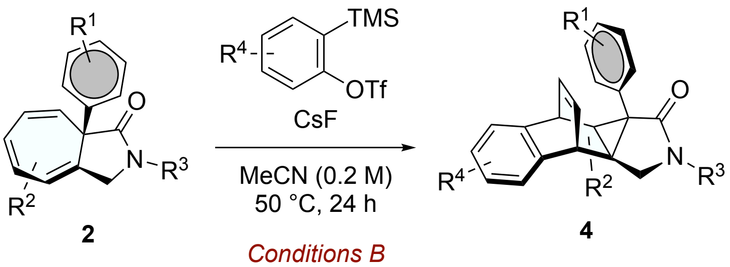

A pre-dried 10-mL test tube equipped with a magnetic stir bar was charged with cycloheptatriene compound 2 (0.1 mmol, 1 equiv.), 2-(trimethylsilyl)phenyl trifluoromethanesulfonate (0.125 mmol, 1.25 equiv.) and CsF (0.25 mmol, 2.5 equiv.). Dry MeCN (0.5 mL, 0.2 M) was injected into the test tube under an argon gas atmosphere. The reaction mixture was stirred for 24 h at 50 °C. The mixture was then diluted with DCM and filtered through a short pad of silica gel. After the solvent was evaporated in vacuo, the resulting residue was purified by flash chromatography (*n*-hexane/EtOAc = 5/1, v/v) to afford desired product (±)-**4**.

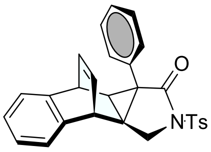

3a-Phenyl-2-tosyl-1,2,3a,3b,4,9-hexahydro-3*H*-4,9-ethenonaphtho[2',3':1,3]cyclopropa[1,2-c]pyrrol-3-one ((±)-4a)

Prepared according to the general procedure B using **2a** (37.7 mg, 0.1 mmol) and isolated as white solid (42.6 mg, 94% yield): TLC R_f = 0.2 (*n*-hexane/EtOAc, 3/1, v/v); m.p. 95–97 ºC; ^{1}H NMR (400 MHz, $CDCl_3$) δ 7.83 (d, *J* = 8.2 Hz, 2H), 7.29-7.14 (m, 8H), 7.12–7.06 (m, 2H), 6.86–6.84 (m, 1H), 5.77 (td, *J* = 6.8, 1.4 Hz, 1H), 5.53 (t, *J* = 6.8 Hz, 1H), 4.23 (d, *J* = 5.0 Hz, 1H), 4.09 (d, *J* = 6.8 Hz, 1H), 4.06 (d, *J* = 10.1 Hz, 1H), 3.63 (d, *J* = 10.1 Hz, 1H), 2.40 (s, 3H), 1.52 (d, *J* = 3.4 Hz, 1H); ^{13}C NMR (100 MHz, $CDCl_3$) δ 171.8, 145.0, 144.9, 144.8, 134.8, 133.9, 133.8, 133.7, 130.6, 130.3, 129.6 (2C), 128.4, 128.3, 128.2 (2C), 127.2, 125.6, 125.1, 123.7, 123.5, 52.4, 51.1, 42.7, 41.2, 40.6, 35.1, 21.7; IR (ATR) ν 3062, 1732, 1599, 1464, 1358, 1290, 1259, 1171, 1134, 1092 cm^{-1}; HRMS (ESI-TOF) $[M + H]^+$ calcd for $C_{28}H_{24}NO_3S^+$ m/z 454.1471, found 454.1469.

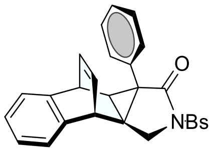

2-((4-Bromophenyl)sulfonyl)-3a-phenyl-1,2,3a,3b,4,9-hexahydro-3*H*-4,9-ethenonaphtho[2',3':1,3]cyclopropa[1,2-c]pyrrol-3-one ((±)-4b)

Prepared according to the general procedure B using **2d** (44.2 mg, 0.1 mmol) and isolated as white solid (47.7 mg, 92% yield): TLC R_f = 0.35 (*n*-hexane/EtOAc, 3/1, v/v); m.p. 233–235 ºC; ^{1}H NMR (400 MHz, $CDCl_3$) δ 7.81 (d, *J* = 8.8 Hz, 2H), 7.60 (d, *J* = 8.8 Hz,

2H), 7.31–7.08 (m, 8H), 6.86–6.84 (m, 1H), 5.78 (td, J = 7.2, 1.4 Hz, 1H), 5.53 (t, J = 7.2 Hz, 1H), 4.25 (t, J = 4.6 Hz, 1H), 4.10 (d, J = 6.0 Hz, 1H), 4.05 (d, J = 10.1 Hz, 1H), 3.62 (d, J = 10.1 Hz, 1H), 1.53 (d, J = 3.7 Hz, 1H); ^{13}C NMR (100 MHz, $CDCl_3$) δ 171.9, 144.8, 136.6, 133.9, 133.8, 133.4, 132.3 (2C), 130.6, 130.3, 129.7 (3C), 129.4, 128.5, 128.4, 127.3, 125.7, 125.2, 123.7, 123.6, 52.3, 51.1, 42.6, 41.2, 40.8, 35.2; IR (ATR) ν 3024, 1730, 1594, 1498, 1468, 1390, 1363, 1311, 1257, 1219 cm^{-1}; HRMS (ESI-TOF) $[M + H]^+$ calcd for $C_{27}H_{21}BrNO_3S^+$ m/z 518.0420, found 518.0427.

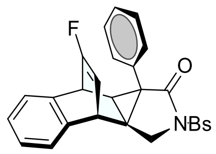

2-((4-Bromophenyl)sulfonyl)-11-fluoro-3a-phenyl-1,2,3a,3b,4,9-hexahydro-3*H*-4,9-ethenonaphtho[2',3':1,3]cyclopropa[1,2-c]pyrrol-3-one ((±)-4c)

Prepared according to the general procedure B using **2u** (46.0 mg, 0.1 mmol) and isolated as white solid (39.2 mg, 73% yield): TLC R_f = 0.2 (*n*-hexane/EtOAc, 3/1, v/v); m.p. 240 ºC (decomp.); ^{1}H NMR (400 MHz, $CDCl_3$) δ 7.82 (d, J = 8.2 Hz, 2H), 7.62 (d, J = 8.2 Hz, 2H), 7.38–7.22 (m, 6H), 7.16–7.11 (m, 2H), 6.90 (d, J = 6.9 Hz, 1H), 5.02–4.99 (m, 1H), 4.13–4.04 (m, 3H), 3.61 (d, J = 10.1 Hz, 1H), 1.56 (dd, J = 6.0, 2.8 Hz, 1H); ^{13}C NMR (100 MHz, $CDCl_3$) δ 171.4, 144.8, 143.7 (d, J = 207.0, 1C), 136.5, 132.4 (2C),

131.4, 130.2, 130.2 (2C), 129.8 (2C), 129.5, 128.8, 128.3, 127.8, 126.1, 125.9, 123.9, 123.5, 108.0 (d, J = 10.5 Hz, 1C), 52.8, 50.6, 42.9 (d, J = 24.0 Hz, 1C), 41.6, 41.1 (d, J = 6.7 Hz, 1C), 33.5; ^{19}F NMR (376 MHz, CDCl$_3$) δ −105.9; IR (ATR) ν 3023, 2360, 1732, 1676, 1574, 1469, 1390, 1362, 1269, 1230 cm^{-1}; HRMS (ESI-TOF) [M + Na]$^+$ calcd for $C_{27}H_{19}BrFNNaO_3S^+$ m/z 558.0145, found 558.0150.

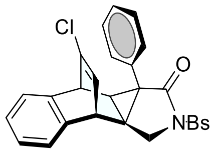

2-((4-Bromophenyl)sulfonyl)-11-chloro-3a-phenyl-1,2,3a,3b,4,9-hexahydro-3*H*-4,9-ethenonaphtho[2',3':1,3]cyclopropa[1,2-c]pyrrol-3-one ((±)-4d)

Prepared according to the general procedure B using **2t** (46.0 mg, 0.1 mmol) and isolated as white solid (39.2 mg, 73% yield): TLC R_f = 0.2 (*n*-hexane/EtOAc, 3/1, v/v); m.p. 240 ºC (decomp.); ^{1}H NMR (400 MHz, CDCl$_3$) δ 7.81 (d, J = 8.7 Hz, 2H), 7.61 (d, J = 8.2 Hz, 2H), 7.36–7.14 (m, 8H), 6.85 (d, J = 4.6 Hz, 1H), 5.76 (dd, J = 7.3, 2.3 Hz, 1H), 4.15 (d, J = 6.4 Hz, 2H), 4.06 (d, J = 9.6 Hz, 1H), 3.60 (d, J = 9.6 Hz, 1H), 1.56 (d, J = 4.6 Hz, 1H); ^{13}C NMR (100 MHz, CDCl$_3$) δ 171.2, 143.3, 143.3, 137.0, 136.5, 132.3 (2C), 131.3, 131.2, 131.0, 130.1, 129.8 (2C), 129.5, 128.8, 128.0, 127.6, 126.1 (2C), 123.9, 123.8, 52.7, 50.5, 49.1, 43.4, 40.8, 34.6; IR (ATR) ν 3026, 2360, 1732, 1622, 1574, 1470, 1390,

1363, 1269, 1225 cm^{-1}; HRMS (ESI-TOF) [M + Na]$^+$ calcd for $C_{27}H_{19}BrClNNaO_3S^+$ m/z 573.9850, found 573.9851.

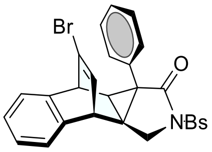

11-Bromo-2-((4-bromophenyl)sulfonyl)-3a-phenyl-1,2,3a,3b,4,9-hexahydro-3*H*-4,9-ethenonaphtho[2',3':1,3]cyclopropa[1,2-c]pyrrol-3-one ((±)-4e)

Prepared according to the general procedure B using **2e** (52.1 mg, 0.1 mmol) and isolated as white solid (52.0 mg, 87% yield): TLC R_f = 0.3 (*n*-hexane/EtOAc, 3/1, v/v); m.p. 240 ºC (decomp.); ^{1}H NMR (400 MHz, $CDCl_3$) δ 7.81 (d, *J* = 8.7 Hz, 2H), 7.61 (d, *J* = 8.2 Hz, 2H), 7.38–7.37 (m, 2H), 7.32–7.23 (m, 4H), 7.16–7.14 (m, 2H), 6.84 (t, *J* = 4.1 Hz, 1H), 6.01 (dd, *J* = 6.9, 2.3 Hz, 1H), 4.27 (t, *J* = 2.3 Hz, 1H), 4.17 (d, *J* = 6.9 Hz, 1H), 4.04 (d, *J* = 10.1 Hz, 1H), 3.60 (d, *J* = 10.1 Hz, 1H), 1.56 (d, *J* = 5.0 Hz, 1H); ^{13}C NMR (100 MHz, $CDCl_3$) δ 171.1, 143.6, 143.0, 136.5, 135.4, 132.4 (2C), 131.6, 131.5, 130.2, 129.8 (2C), 129.5, 129.0, 128.0, 127.6, 126.1, 126.1, 125.5, 123.9, 123.8, 52.7, 50.8, 50.5, 44.5, 40.3, 35.2; IR (ATR) ν 3024, 2360, 1730, 1574, 1498, 1390, 1363, 1269, 1225, 1171 cm^{-1}; HRMS (ESI-TOF) [M + Na]$^+$ calcd for $C_{27}H_{19}Br_2NNaO_3S^+$ m/z 617.9345, found 617.9334.

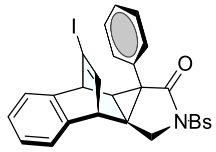

2-((4-Bromophenyl)sulfonyl)-11-iodo-3a-phenyl-1,2,3a,3b,4,9-hexahydro-3*H*-4,9-ethenonaphtho[2',3':1,3]cyclopropa[1,2-c]pyrrol-3-one ((±)-4f)

Prepared according to the general procedure B using **2r** (56.8 mg, 0.1 mmol) and isolated as white solid (48.3 mg, 75% yield): TLC R_f = 0.35 (*n*-hexane/EtOAc, 3/1, v/v); m.p. 215 ºC (decomp.); ^{1}H NMR (400 MHz, $CDCl_3$) δ 7.81 (d, *J* = 8.7 Hz, 2H), 7.61 (d, *J* = 8.7 Hz, 2H), 7.44–7.37 (m, 2H), 7.31–7.24 (m, 4H), 7.17–7.12 (m, 2H), 6.82–6.81 (m, 1H), 6.35 (dd, *J* = 6.9, 2.3 Hz, 1H), 4.38 (s, 1H), 4.12 (d, *J* = 6.9 Hz, 1H), 4.02 (d, *J* = 9.6 Hz, 1H), 3.59 (d, *J* = 9.6 Hz, 1H), 1.53 (d, *J* = 3.7 Hz, 1H); ^{13}C NMR (100 MHz, $CDCl_3$) δ 171.0, 143.9 (2C), 142.7, 136.5, 132.3 (2C), 132.3, 131.9, 130.3, 129.8 (2C), 129.5, 129.2, 128.1, 127.6, 126.0, 126.0, 123.9, 123.9, 97.0, 54.0, 52.6, 50.6, 45.8, 39.9, 36.1; IR (ATR) ν 3062, 2362, 1732, 1574, 1469, 1390, 1363, 1269, 1223, 1173 cm^{-1}; HRMS (ESI-TOF) $[M + Na]^+$ calcd for $C_{27}H_{19}BrINNaO_3S^+$ m/z 665.9206, found 665.9190.

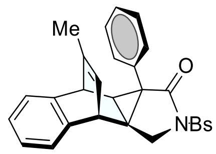

2-((4-Bromophenyl)sulfonyl)-11-methyl-3a-phenyl-1,2,3a,3b,4,9-hexahydro-3*H*-4,9-ethenonaphtho[2',3':1,3]cyclopropa[1,2-c]pyrrol-3-one ((±)-4g)

Prepared according to the general procedure B using **2q** (45.6 mg, 0.1 mmol) and isolated as white solid (37.3 mg, 70% yield): TLC $R_f = 0.4$ (*n*-hexane/EtOAc, 3/1, v/v); m.p. 205 ºC (decomp.); ^{1}H NMR (400 MHz, $CDCl_3$) δ 7.81 (d, *J* = 8.7 Hz, 2H), 7.60 (d, *J* = 8.7 Hz, 2H), 7.32–7.19 (m, 6H), 7.10–7.08 (m, 2H), 6.88–6.86 (m, 1H), 5.50 (d, *J* = 6.4 Hz, 1H), 4.05–4.02 (m, 2H), 3.88 (s, 1H), 3.59 (d, *J* = 10.1 Hz, 1H), 1.50 (d, *J* = 3.7 Hz, 1H), 1.06 (s, 3H); ^{13}C NMR (100 MHz, $CDCl_3$) δ 171.8, 145.2, 144.9, 142.8, 136.6, 132.7, 132.3 (2C), 131.0, 130.6, 130.2, 129.8 (2C), 129.3, 128.2, 127.7, 127.2, 125.5, 125.3, 123.4, 123.4, 52.1, 50.9, 46.4, 42.7, 41.1, 35.3, 19.3; IR (ATR) ν 3060, 2360, 1730, 1574, 1469, 1390, 1363, 1279, 1254, 1254 cm^{-1}; HRMS (ESI-TOF) $[M + H]^+$ calcd for $C_{28}H_{23}BrNO_3S^+$ m/z 532.0577, found 532.0585.

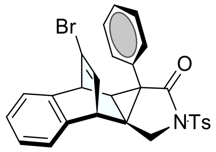

11-Bromo-3a-phenyl-2-tosyl-1,2,3a,3b,4,9-hexahydro-3*H*-4,9-ethenonaphtho[2',3':1,3]cyclopropa[1,2-c]pyrrol-3-one ((±)-4h)

Prepared according to the general procedure B using **2b** (45.6 mg, 0.1 mmol) and isolated as white solid (47.9 mg, 90% yield): TLC R_f = 0.2 (*n*-hexane/EtOAc, 3/1, v/v); m.p. 108–110 ºC; ^{1}H NMR (400 MHz, $CDCl_3$) δ 7.82 (d, *J* = 8.2 Hz, 2H), 7.38–7.35 (m, 2H), 7.31–7.21 (m, 6H), 7.14 (dd, *J* = 5.0, 2.8 Hz, 2H), 6.84–6.82 (m, 1H), 6.00 (dd, *J* = 6.9, 2.3 Hz, 1H), 4.26–4.24 (m, 1H), 4.17 (d, *J* = 6.9 Hz, 1H), 4.05 (d, *J* = 10.1 Hz, 1H), 3.60 (d, *J* = 10.1 Hz, 1H), 2.40 (s, 3H), 1.54 (d, *J* = 4.1 Hz, 1H); ^{13}C NMR (100 MHz, $CDCl_3$) δ 171.0, 145.2, 143.6, 143.1, 135.4, 134.6, 131.7, 131.7, 130.2, 129.6 (2C), 128.9, 128.2 (2C), 127.9, 127.4, 126.0, 126.0, 125.5, 123.9, 123.8, 52.8, 50.8, 50.5, 44_others.6, 40.2, 35.1, 21.7; IR (ATR) ν 3014, 1732, 1597, 1464, 1358, 1269, 1225, 1171, 1132, 1092 cm^{-1}; HRMS (ESI-TOF) $[M + H]^+$ calcd for $C_{28}H_{23}BrNO_3S^+$ m/z 532.0577, found 532.0574.

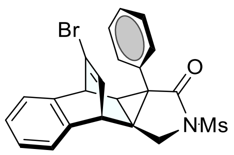

11-bromo-2-(methylsulfonyl)-3a-phenyl-1,2,3a,3b,4,9-hexahydro-3*H*-4,9-ethenonaphtho[2',3':1,3]cyclopropa[1,2-c]pyrrol-3-one ((±)-4i)

Prepared according to the general procedure B using **2i** (38.0 mg, 0.1 mmol) and isolated as white solid (35.1 mg, 77% yield): TLC R_f = 0.1 (*n*-hexane/EtOAc, 3/1, v/v); m.p. 220 ºC (decomp.); ^{1}H NMR (400 MHz, $CDCl_3$) δ 7.48–7.40 (m, 2H), 7.32–7.25 (m, 4H),

7.16–7.11 (m, 2H), 6.97 (dd, *J* = 5.0, 3.2 Hz, 1H), 6.05 (dd, *J* = 6.9, 2.3 Hz, 1H), 4.33–4.31 (m, 1H), 4.20 (d, *J* = 6.9 Hz, 1H), 4.02 (d, *J* = 10.1 Hz, 1H), 3.54 (d, *J* = 10.1 Hz, 1H), 3.19 (s, 3H), 1.68 (d, *J* = 4.1 Hz, 1H); ^{13}C NMR (100 MHz, $CDCl_3$) δ 172.2, 143.5, 142.9, 135.4, 131.7, 131.5, 130.2, 129.0, 128.1, 127.6, 126.1, 126.1, 125.5, 123.9, 123.8, 52.8, 50.8, 49.8, 44.5, 40.6, 40.3, 35.3; IR (ATR) ν 3022, 2360, 1726, 1512, 1354, 1271, 1221, 1167, 1136, 1011 cm^{-1}; HRMS (ESI-TOF) $[M + H]^+$ calcd for $C_{22}H_{19}BrNO_3S^+$ m/z 456.0264, found 456.0270.

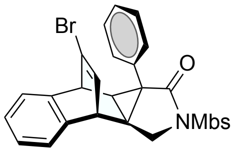

11-Bromo-2-((4-methoxyphenyl)sulfonyl)-3a-phenyl-1,2,3a,3b,4,9-hexahydro-3*H*-4,9-ethenonaphtho[2',3':1,3]cyclopropa[1,2-c]pyrrol-3-one ((±)-4j)

Prepared according to the general procedure B using **2c** (47.2 mg, 0.1 mmol) and isolated as white solid (49.9 mg, 91% yield): TLC R_f = 0.2 (*n*-hexane/EtOAc, 3/1, v/v); m.p. 210 ºC (decomp.); ^{1}H NMR (400 MHz, $CDCl_3$) δ 7.88 (d, *J* = 9.2 Hz, 2H), 7.40–7.21 (m, 6H), 7.16–7.13 (m, 2H), 6.91 (d, *J* = 9.2 Hz, 2H), 6.84–6.82 (m, 1H), 6.00 (dd, *J* = 7.2, 2.3 Hz, 1H), 4.26–4.25 (m, 1H), 4.16 (d, *J* = 7.2 Hz, 1H), 4.05 (d, *J* = 10.1 Hz, 1H), 3.83 (s, 3H), 3.59 (d, *J* = 10.1 Hz, 1H), 1.53 (d, *J* = 4.1 Hz, 1H); ^{13}C NMR (100 MHz, $CDCl_3$) δ 171.0,

164.0, 143.6, 143.1, 135.4, 131.8, 131.6, 130.6 (2C), 130.2, 128.9, 128.9, 127.9, 127.4, 126.0, 126.0, 125.5, 123.8, 123.8, 114.1 (2C), 55.6, 52.8, 50.8, 50.5, 44.6, 40.2, 35.2; IR (ATR) ν 3024, 1728, 1595, 1496, 1462, 1415, 1358, 1309, 1265, 1225 cm^{-1}; HRMS (ESI-TOF) $[M + H]^+$ calcd for $C_{28}H_{23}BrNO_4S^+$ m/z 548.0526, found 548.0528.

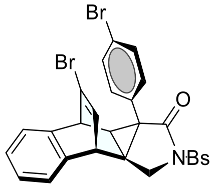

11-Bromo-3a-(4-bromophenyl)-2-((4-bromophenyl)sulfonyl)-1,2,3a,3b,4,9-hexahydro-3*H*-4,9-ethenonaphtho[2',3':1,3]cyclopropa[1,2-c]pyrrol-3-one ((±)-4k)

Prepared according to the general procedure B using **2m** (60.0 mg, 0.1 mmol) and isolated as white solid (61.5 mg, 91% yield): TLC R_f = 0.3 (*n*-hexane/EtOAc, 3/1, v/v); m.p. 245 ºC (decomp.); ^{1}H NMR (400 MHz, $CDCl_3$) δ 7.80 (d, *J* = 8.7 Hz, 2H), 7.62 (d, *J* = 8.7 Hz, 2H), 7.50 (dd, *J* = 8.2, 1.8 Hz, 1H), 7.37 (dd, *J* = 8.2, 1.8 Hz, 1H), 7.31–7.24 (m, 3H), 7.17–7.13 (m, 2H), 6.73 (dd, *J* = 8.2, 1.8 Hz, 1H), 6.02 (dd, *J* = 6.9, 2.3 Hz, 1H), 4.27–4.25 (m, 1H), 4.16 (d, *J* = 6.9 Hz, 1H), 4.02 (d, *J* = 10.1 Hz, 1H), 3.59 (d, *J* = 10.1 Hz, 1H), 1.57 (d, *J* = 4.1 Hz, 1H); ^{13}C NMR (100 MHz, $CDCl_3$) δ 170.7, 143.3, 142.7, 136.3, 135.5, 133.2, 132.4 (2C), 132.0, 131.8, 131.2, 130.6, 129.7 (2C), 129.7, 126.2, 126.2, 125.6, 124.0, 123.9, 121.7, 52.1, 50.7, 50.4, 44.4, 40.5, 35.1; IR (ATR) ν 3078, 2360,

1732, 1574, 1491, 1469, 1391, 1363, 1269, 1223 cm^{-1}; HRMS (ESI-TOF) [M + Na]$^+$ calcd for $C_{27}H_{18}Br_3NNaO_3S^+$ m/z 695.8450, found 695.8473.

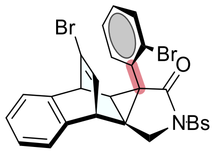

11-Bromo-3a-(2-bromophenyl)-2-((4-bromophenyl)sulfonyl)-1,2,3a,3b,4,9-hexahydro-3*H*-4,9-ethenonaphtho[2',3':1,3]cyclopropa[1,2-c]pyrrol-3-one ((±)-4l)

Prepared according to the general procedure B using **1o** (60.0 mg, 0.1 mmol) and isolated as white solid (49.4 mg, 73% yield): TLC R_f = 0.25 (*n*-hexane/EtOAc, 3/1, v/v); m.p. 180 ºC (decomp.); ^{1}H NMR (400 MHz, $CDCl_3$) δ 7.85–7.82 (m, 2H), 7.65–7.61 (m, 2H), 7.43 (d, J = 7.3 Hz, 1H), 7.40–7.31 (m, 3H), 7.29–7.25 (m, 1H), 7.20–7.09 (m, 2.87H) , 6.81 (dd, J = 7.6, 1.6 Hz, 0.13H), 6.08 (dd, J = 6.9, 2.3 Hz, 0.87H), 5.98 (dd, J = 6.9, 2.3 Hz, 0.13H), 4.48–4.47 (m, 0.13H), 4.35–4.33 (m, 0.87H), 4.17–4.12 (m, 1H), 4.03 (d, J = 10.1 Hz, 0.87H), 3.98 (d, J = 10.1 Hz, 0.13H), 3.68 (d, J = 10.1 Hz, 0.87H), 3.61 (d, J = 10.1 Hz, 0.13H), 1.88 (d, J = 4.1 Hz, 0.13H), 1.80 (d, J = 4.1 Hz, 0.87H); ^{13}C NMR (100 MHz, $CDCl_3$) δ 170.7, 143.1, 136.1, 133.8, 133.7, 132.8, 132.4, 132.3 (2C), 132.0, 129.7 (2C), 129.5, 129.4, 128.1, 126.5, 126.2, 126.1, 124.1, 124.0, 123.9, 52.8, 51.1, 50.1, 45.7,

42.3, 34.5; IR (ATR) ν 3076, 2360, 1737, 1574, 1469, 1390, 1365, 1271, 1223, 1174 cm^{-1}; HRMS (ESI-TOF) $[M + Na]^+$ calcd for $C_{27}H_{18}Br_3NNaO_3S^+$ m/z 695.8450, found 695.8462.

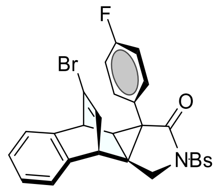

11-Bromo-2-((4-bromophenyl)sulfonyl)-3a-(4-fluorophenyl)-1,2,3a,3b,4,9-hexahydro-3*H*-4,9-ethenonaphtho[2',3':1,3]cyclopropa[1,2-c]pyrrol-3-one ((±)-4m)

Prepared according to the general procedure B using **2l** (53.9 mg, 0.1 mmol) and isolated as white solid (44.3 mg, 72% yield): TLC R_f = 0.3 (*n*-hexane/EtOAc, 3/1, v/v); m.p. 240 ºC (decomp.); ^{1}H NMR (400 MHz, $CDCl_3$) δ 7.81 (d, *J* = 8.7 Hz, 2H), 7.62 (d, *J* = 8.7 Hz, 2H), 7.38–7.27 (m, 3H), 7.18–7.13 (m, 2H), 7.06 (td, *J* = 8.6, 2.4 Hz, 1H), 6.95 (td, *J* = 8.6, 2.4 Hz, 1H), 6.83–6.79 (m, 1H), 6.03 (dd, *J* = 7.0, 2.2 Hz, 1H), 4.27–4.25 (m, 1H), 4.16 (d, *J* = 7.0 Hz, 1H), 4.03 (d, *J* = 10.1 Hz, 1H), 3.59 (d, *J* = 10.1 Hz, 1H), 1.56 (d, *J* = 5.0 Hz, 1H); ^{13}C NMR (100 MHz, $CDCl_3$) δ 171.0, 162.1 (d, *J* = 246.3 Hz, 1C), 143.4, 142.9, 136.5, 135.2, 133.2 (d, *J* = 8.6 Hz, 1C), 132.4 (2C), 131.8 (d, *J* = 8.6 Hz, 1C), 129.7 (2C), 129.6, 127.4 (d, *J* = 3.8 Hz, 1C), 126.2, 126.1, 125.6, 124.0, 123.9, 115.7 (d, *J* = 22.0 Hz, 1C), 115.3 (d, *J* = 22.0 Hz, 1C), 52.0, 50.8, 50.5, 44.5, 40.4, 35.3; ^{19}F

NMR (376 MHz, $CDCl_3$) δ −114.2; IR (ATR) ν 3022, 2360, 1730, 1608, 1574, 1512, 1478, 1390, 1362, 1269 cm^{-1}; HRMS (ESI-TOF) $[M + H]^+$ calcd for $C_{27}H_{19}Br_2FNO_3S^+$ m/z 613.9431, found 613.9441.

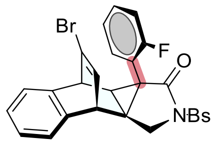

11-Bromo-2-((4-bromophenyl)sulfonyl)-3a-(2-fluorophenyl)-1,2,3a,3b,4,9-hexahydro-3*H*-4,9-ethenonaphtho[2',3':1,3]cyclopropa[1,2-c]pyrrol-3-one ((±)-4n)

major rotamer; Prepared according to the general procedure B using **2j** (53.9 mg, 0.1 mmol) and isolated as white solid (33.1 mg, 54% yield): TLC R_f = 0.4 (*n*-hexane/EtOAc, 3/1, v/v); m.p. 200 ºC (decomp.); ^{1}H NMR (400 MHz, $CDCl_3$) δ 7.81 (d, *J* = 8.2 Hz, 2H), 7.63 (d, *J* = 8.2 Hz, 2H), 7.38 (t, *J* = 7.3 Hz, 1H), 7.32–7.24 (m, 3H), 7.20–7.14 (m, 3H), 6.93 (t, *J* = 8.2 Hz, 1H), 6.11 (dd, *J* = 6.9, 1.4 Hz, 1H), 4.28 (s, 1H), 4.16 (d, *J* = 6.9 Hz, 1H), 4.03 (d, *J* = 10.1 Hz, 1H), 3.64 (d, *J* = 10.1 Hz, 1H), 1.65–1.64 (m, 1H); ^{13}C NMR (100 MHz, $CDCl_3$) δ 170.5, 143.3, 143.1, 136.4, 134.1, 133.3 (d, *J* = 2.9 Hz, 1C), 132.4 (2C), 130.0, 129.9, 129.7 (2C), 129.6, 126.2, 126.1, 125.9, 124.6 (d, *J* = 3.8 Hz, 1C), 124.0, 123.9, 119.5 (d, *J* = 14.4 Hz, 1C), 114.6 (d, *J* = 21.1 Hz, 1C), 50.9, 50.3, 46.4, 45.0, 40.6, 34.1; ^{19}F NMR (376 MHz, $CDCl_3$) δ −115.5; IR (ATR) ν 3084, 2927, 2360, 1732,

1574, 1495, 1469, 1390, 1365, 1271 cm^{-1}; HRMS (ESI-TOF) [M + Na]$^+$ calcd for $C_{27}H_{18}Br_2FNNaO_3S^+$ m/z 635.9250, found 635.9244.

minor rotamer; Prepared according to the general procedure B using **2j** (53.9 mg, 0.1 mmol) and isolated as white solid (19.1 mg, 31% yield): TLC R_f = 0.3 (*n*-hexane/EtOAc, 3/1, v/v); m.p. 200 ºC (decomp.); ^{1}H NMR (400 MHz, $CDCl_3$) δ 7.82 (d, *J* = 8.7 Hz, 2H), 7.63 (d, *J* = 8.7 Hz, 2H), 7.40–7.23 (m, 2H), 7.20–7.03 (m, 5H), 6.83 (td, *J* = 7.3, 1.4 Hz, 1H), 6.03 (dd, *J* = 6.9, 2.3 Hz, 1H), 4.35 (s, 1H), 4.16 (d, *J* = 6.9 Hz, 1H), 4.05 (d, *J* = 10.1 Hz, 1H), 3.62 (d, *J* = 10.1 Hz, 1H), 1.73 (d, *J* = 4.1 Hz, 1H); ^{13}C NMR (100 MHz, $CDCl_3$) δ 170.1, 143.6, 142.6, 136.4, 135.5, 132.4 (2C), 132.1 (d, *J* = 2.9 Hz, 1C), 129.8 (2C), 129.7, 129.6 , 129.5 (d, *J* = 7.7 Hz, 1C), 126.1 (d, *J* = 2.9 Hz, 1C), 125.4, 124.1, 124.0, 123.9, 123.8, 123.8, 116.8 (d, *J* = 21.1 Hz, 1C), 51.1, 50.4, 47.2, 44.6, 39.2, 35.6; ^{19}F NMR (376 MHz, $CDCl_3$) δ −114.5; IR (ATR) ν 3084, 2927, 2360, 1732, 1574, 1495, 1469, 1390, 1365, 1271 cm^{-1}; HRMS (ESI-TOF) [M + Na]$^+$ calcd for $C_{27}H_{18}Br_2FNNaO_3S^+$ m/z 635.9250, found 635.9244.

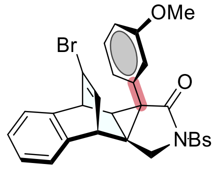

11-Bromo-2-((4-bromophenyl)sulfonyl)-3a-(3-methoxyphenyl)-1,2,3a,3b,4,9-hexahydro-3*H*-4,9-ethenonaphtho[2',3':1,3]cyclopropa[1,2-c]pyrrol-3-one ((±)-4o)

Prepared according to the general procedure B using **2k** (55.1 mg, 0.1 mmol) and isolated as white solid (56.5 mg, 90% yield): TLC R_f = 0.25 (*n*-hexane/EtOAc, 3/1, v/v); m.p. 180 ºC (decomp.); ^{1}H NMR (400 MHz, $CDCl_3$) δ 7.83–7.80 (m, 2H), 7.61 (d, *J* = 8.7 Hz, 2H), 7.31–7.27 (m, 2H), 7.17–7.13 (m, 3H), 6.98 (d, *J* = 7.8 Hz, 0.45H), 6.90 (s, 0.55H), 6.80 (dd, *J* = 8.2, 2.3 Hz, 0.45H), 6.76 (dd, *J* = 8.2, 2.3 Hz, 0.55H), 6.42 (d, *J* = 8.2 Hz, 0.55H), 6.35 (s, 0.45H), 6.05 (dd, *J* = 6.9, 2.3 Hz, 0.45H), 5.99 (dd, *J* = 6.9, 2.3 Hz, 0.55H), 4.30 (t, *J* = 3.2 Hz, 0.55H), 4.27 (t, *J* = 3.2 Hz, 0.45H), 4.16–4.13 (m, 1H), 4.04–4.00 (m, 1H), 3.85 (s, 1.65H), 3.76 (s, 1.35H), 3.61–3.57 (m, 1H), 1.57 (d, *J* = 4.1 Hz, 0.55H), 1.54 (d, *J* = 3.7 Hz, 0.45H); ^{13}C NMR (100 MHz, $CDCl_3$) δ 171.0, 159.4 (0.55C), 159.2 (0.45C), 143.6 (0.45C), 143.6 (0.55C), 143.0 (0.45C), 143.0 (0.55C), 136.4 (0.55C), 136.4 (0.45C), 135.5 (0.55C), 135.3 (0.45C), 133.0 (0.55C), 132.8 (0.45C), 132.3 (2C), 130.0, 129.8 (2C), 129.5, 129.2, 126.1 (0.45C), 126.0 (0.55C), 125.3 (0.45C), 125.1(0.55C), 124.3, 123.9 (0.45C), 123.8 (0.55C), 122.2, 117.2 (0.55C), 116.1 (0.45C), 113.4 (0.55C), 112.5 (0.45C), 55.2, 55.2, 52.6, 50.8 (0.55C), 50.5 (0.45C), 44.4, 40.7 (0.55C), 40.4 (0.45C), 35.2; IR (ATR) ν 2933, 2360, 1732, 1576, 1471, 1468, 1431, 1390, 1362, 1271 cm^{-1};

HRMS (ESI-TOF) $[M + Na]^+$ calcd for $C_{28}H_{21}Br_2NNaO_4S^+$ m/z 647.9450, found 647.9450.

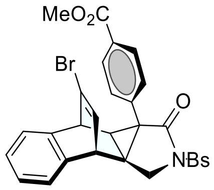

Methyl 4-((3b*R*,4*S*,9*S*,9a*R*)-11-bromo-2-((4-bromophenyl)sulfonyl)-3-oxo-2,3,4,9-tetrahydro-1*H*-4,9-ethenonaphtho[2',3':1,3]cyclopropa[1,2-c]pyrrol-3a(3bH)-yl)benzoate ((±)-4p)

Prepared according to the general procedure B using **2p** (57.9 mg, 0.1 mmol) and isolated as white solid (58.3 mg, 89% yield): TLC $R_f = 0.2$ (*n*-hexane/EtOAc, 3/1, v/v); m.p. 144–146 ºC; ^{1}H NMR (400 MHz, $CDCl_3$) δ 8.06 (d, *J* = 7.8 Hz, 1H), 7.91 (d, *J* = 7.8 Hz, 1H), 7.80 (d, *J* = 8.7 Hz, 2H), 7.62 (d, *J* = 8.7 Hz, 2H), 7.46 (d, *J* = 8.2 Hz, 1H), 7.32–7.26 (m, 3H), 7.17–7.14 (m, 2H), 6.94 (d, *J* = 8.2 Hz, 1H), 6.01 (dd, *J* = 6.9, 2.3 Hz, 1H), 4.29–4.27 (m, 1H), 4.19 (d, *J* = 6.9 Hz, 1H), 4.06 (d, *J* = 9.6 Hz, 1H), 3.90 (s, 3H), 3.61 (d, *J* = 9.6 Hz, 1H), 1.61 (d, *J* = 4.1 Hz, 1H); ^{13}C NMR (100 MHz, $CDCl_3$) δ 170.4, 166.7, 143.2, 142.6, 136.9, 136.3, 136.0, 132.4 (2C), 131.7, 130.3 (2C), 129.7 (2C), 129.7, 129.2, 129.1, 126.2, 126.2, 125.7, 124.0, 123.9, 52.3, 52.1, 50.7, 50.4, 44.4, 40.7, 35.2; IR (ATR) ν

3022, 2360, 1722, 1612, 1574, 1512, 1468, 1435, 1390, 1363 cm^{-1}; HRMS (ESI-TOF) [M + H]$^+$ calcd for $C_{29}H_{22}Br_2NO_5S^+$ m/z 653.9580, found 653.9577.

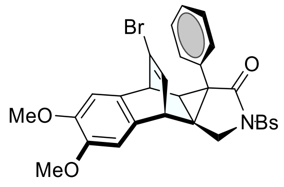

11-Bromo-2-((4-bromophenyl)sulfonyl)-6,7-dimethoxy-3a-phenyl-1,2,3a,3b,4,9-hexahydro-3*H*-4,9-ethenonaphtho[2',3':1,3]cyclopropa[1,2-c]pyrrol-3-one ((±)-4q)

Prepared according to the general procedure B using **2e** (52.1 mg, 0.1 mmol), 4,5-dimethoxy-2-(trimethylsilyl)phenyl trifluoromethanesulfonate (44.8 mg, 0.125 mmol) and isolated as white solid (57.2 mg, 87% yield): TLC R_f = 0.1 (*n*-hexane/EtOAc, 3/1, v/v); m.p. 230 ºC (decomp.); ^{1}H NMR (400 MHz, $CDCl_3$) δ 7.82 (d, *J* = 8.2 Hz, 2H), 7.61 (d, *J* = 8.2 Hz, 2H), 7.37 (s, 2H), 7.24 (s, 2H), 6.93 (s, 1H), 6.90 (s, 1H), 6.83 (s, 1H), 6.02 (d, *J* = 6.0 Hz, 1H), 4.19 (s, 1H), 4.10–4.05 (m, 2H), 3.90 (s, 6H), 3.62 (d, *J* = 9.6 Hz, 1H), 1.52 (s, 1H); ^{13}C NMR (100 MHz, $CDCl_3$) δ 171.0, 146.4, 146.4, 136.4, 136.1, 135.7, 135.5, 132.3 (2C), 131.6, 131.4, 130.2, 129.7 (2C), 129.5, 128.9, 128.0, 127.5, 125.9, 108.9, 108.7, 56.3 (2C), 53.2, 50.6 (2C), 44.4, 40.6, 35.5; IR (ATR) ν 3018, 2954, 1730, 1604, 1574, 1498, 1466, 1392, 1362, 1292 cm^{-1}; HRMS (ESI-TOF) [M + H]$^+$ calcd for $C_{29}H_{24}Br_2NO_5S^+$ m/z 655.9736, found 655.9745.

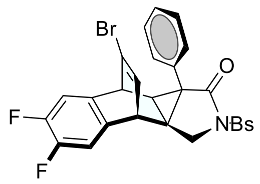

11-Bromo-2-((4-bromophenyl)sulfonyl)-6,7-difluoro-3a-phenyl-1,2,3a,3b,4,9-hexahydro-3*H*-4,9-ethenonaphtho[2',3':1,3]cyclopropa[1,2-c]pyrrol-3-one ((±)-4r)

Prepared according to the general procedure B using **2e** (52.1 mg, 0.1 mmol, 1 equiv.), 4,5-difluoro-2-(trimethylsilyl)phenyl trifluoromethanesulfonate (100.2 mg, 0.3 mmol, 3 equiv.), CsF (95.4 mg, 0.6 mmol, 6 equiv.) and isolated as white solid (44.3 mg, 70% yield): TLC R_f = 0.3 (*n*-hexane/EtOAc, 3/1, v/v); m.p. 240 ºC (decomp.); ^{1}H NMR (400 MHz, $CDCl_3$) δ 7.82 (d, *J* = 8.7 Hz, 2H), 7.62 (d, *J* = 8.7 Hz, 2H), 7.37–7.36 (m, 2H), 7.25-7.23 (m, 2H), 7.16 (d, *J* = 9.6 Hz, 1H), 7.11 (d, *J* = 9.6 Hz, 1H), 6.83–6.79 (m, 1H), 6.01 (dd, *J* = 6.9, 2.3 Hz, 1H), 4.24–4.23 (m, 1H), 4.15 (d, *J* = 6.9 Hz, 1H), 4.05 (d, *J* = 10.1 Hz, 1H), 3.60 (d, *J* = 10.1 Hz, 1H), 1.53 (d, *J* = 4.1 Hz, 1H); ^{13}C NMR (100 MHz, $CDCl_3$) δ 170.6, 146.7 (d, *J* = 4.8 Hz, 1C), 146.5 (d, *J* = 5.8 Hz, 1C), 139.8 (d, *J* = 5.8 Hz, 1C), 139.3–139.2 (m, 1C), 136.3, 135.3, 132.4 (2C), 131.5, 131.0, 130.1, 129.8 (2C), 129.7, 129.0, 128.1, 127.7, 125.5, 113.8 (d, *J* = 23.0 Hz, 1C), 113.6 (d, *J* = 22.0 Hz, 1C), 52.8, 50.3, 50.2, 43.9, 39.9, 34.7; ^{19}F NMR (376 MHz, $CDCl_3$) δ −140.5, −140.6; IR

(ATR) ν 3062, 2362, 1734, 1610, 1574, 1487, 1431, 1390, 1363, 1275 cm^{-1}; HRMS (ESI-TOF) $[M + Na]^+$ calcd for $C_{27}H_{17}Br_2F_2NNaO_3S^+$ m/z 653.9156, found 653.9180.

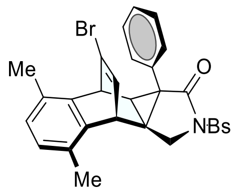

11-Bromo-2-((4-bromophenyl)sulfonyl)-5,8-dimethyl-3a-phenyl-1,2,3a,3b,4,9-hexahydro-3*H*-4,9-ethenonaphtho[2',3':1,3]cyclopropa[1,2-c]pyrrol-3-one ((±)-4s)

Prepared according to the general procedure B using **2e** (52.1 mg, 0.1 mmol), 4,5-dimethyl-2-(trimethylsilyl)phenyl trifluoromethanesulfonate (40.8 mg, 0.125 mmol) and isolated as white solid (35.0 mg, 56% yield): TLC R_f = 0.4 (*n*-hexane/EtOAc, 3/1, v/v); m.p. 245 ºC (decomp.); ^{1}H NMR (400 MHz, $CDCl_3$) δ 7.83 (d, *J* = 8.7 Hz, 2H), 7.61 (d, *J* = 8.7 Hz, 2H), 7.42–7.35 (m, 2H), 7.25–7.23 (m, 2H), 6.87–6.84 (m, 3H), 5.98 (dd, *J* = 6.9, 2.3 Hz, 1H), 4.51 (dd, *J* = 4.1, 2.3 Hz, 1H), 4.43 (d, *J* = 6.9 Hz, 1H), 4.03 (d, *J* = 9.6 Hz, 1H), 3.60 (d, *J* = 9.6 Hz, 1H), 2.36 (s, 6H), 1.52 (d, *J* = 4.1 Hz, 1H); ^{13}C NMR (100 MHz, $CDCl_3$) δ 171.2, 141.8, 141.0, 136.5, 135.3 (2C), 132.3 (2C), 131.6, 131.6, 130.2, 129.8 (2C), 129.6, 129.5, 128.9, 128.0, 127.5, 127.2, 127.2, 125.6, 52.4, 50.3, 47.5, 40.9, 40.2, 34.8, 18.3, 18.2; IR (ATR) ν 3026, 2360, 2299, 1734, 1622, 1574, 1493, 1473, 1448,

1390 cm^{-1}; HRMS (ESI-TOF) [M + H]$^+$ calcd for $C_{29}H_{24}Br_2NO_3S^+$ m/z 623.9838, found 623.9867.

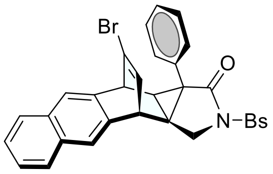

13-Bromo-2-((4-bromophenyl)sulfonyl)-3a-phenyl-1,2,3a,3b,4,11-hexahydro-3*H*-4,11-ethenoanthra[2',3':1,3]cyclopropa[1,2-c]pyrrol-3-one ((±)-4t)

Prepared according to the general procedure B using **2e** (52.1 mg, 0.1 mmol, 1 equiv.), 3-(trimethylsilyl)naphthalen-2-yl trifluoromethanesulfonate (174.2 mg, 0.5 mmol, 5 equiv.), CsF (159.1 mg, 1.0 mmol, 10 equiv.) and isolated as white solid (57.5 mg, 89% yield): TLC R_f = 0.3 (*n*-hexane/EtOAc, 3/1, v/v); m.p. 220 ºC (decomp.); ^{1}H NMR (400 MHz, $CDCl_3$) δ 7.82–7.77 (m, 4H), 7.69 (d, *J* = 8.2 Hz, 2H), 7.60 (d, *J* = 8.7 Hz, 2H), 7.53–7.49 (m, 2H), 7.40–7.37 (m, 2H), 7.28–7.26 (m, 2H), 6.91–6.89 (m, 1H), 6.04 (dd, *J* = 6.9, 2.3 Hz, 1H), 4.37–4.36 (m, 1H), 4.27 (d, *J* = 6.9 Hz, 1H), 4.10 (d, *J* = 10.1 Hz, 1H), 3.62 (d, *J* = 10.1 Hz, 1H), 1.56 (d, *J* = 4.1 Hz, 1H); ^{13}C NMR (100 MHz, $CDCl_3$) δ 171.1, 139.8, 139.3, 136.6, 134.9, 132.4 (2C), 131.9, 131.6 (2C), 130.2, 129.8 (2C), 129.5, 129.1, 128.1, 127.8, 127.7, 127.6, 126.5 (2C), 126.5, 125.1, 122.5, 122.3, 50.9, 50.6, 50.5, 44.3, 39.0, 34.5; IR (ATR) ν 3059, 2362, 2299, 1734, 1574, 1502, 1473, 1448, 1390, 1363 cm^{-1};

HRMS (ESI-TOF) $[M + Na]^+$ calcd for $C_{31}H_{21}Br_2NNaO_3S^+$ m/z 667.9501, found 667.9492.

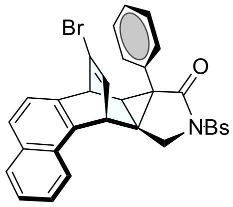

13-Bromo-2-((4-bromophenyl)sulfonyl)-3a-phenyl-1,2,3a,3b,4,11-hexahydro-3*H*-4,11-ethenocyclopenta[2,3]cyclopropa[1,2-b]phenanthren-3-one ((±)-4u)

Prepared according to the general procedure B using **2e** (52.1 mg, 0.1 mmol), 1-(trimethylsilyl)naphthalen-2-yl trifluoromethanesulfonate (43.6 mg, 0.125 mmol) and isolated as white solid (33.7 mg, 52% yield): TLC R_f = 0.4 (*n*-hexane/EtOAc, 3/1, v/v); m.p. 210 ºC (decomp.); ^{1}H NMR (400 MHz, $CDCl_3$) δ 8.03 (d, *J* = 8.5 Hz, 1H), 7.91 (d, *J* = 8.2 Hz, 1H), 7.77 (d, *J* = 8.2 Hz, 2H), 7.71 (d, *J* = 7.8 Hz, 1H), 7.61–7.56 (m, 3H), 7.54–7.47 (m, 2H), 7.44–7.38 (m, 2H), 7.29–7.27 (m, 2H), 6.94–6.92 (m, 1H), 6.12 (dd, *J* = 6.9, 2.3 Hz, 1H), 5.04 (d, *J* = 6.9 Hz, 1H), 4.46–4.44 (m, 1H), 4.09 (d, *J* = 10.1 Hz, 1H), 3.41 (d, *J* = 10.1 Hz, 1H), 1.64 (d, *J* = 4.1 Hz, 1H); ^{13}C NMR (100 MHz, $CDCl_3$) δ 170.9, 142.5, 140.1, 136.4, 135.5, 132.3 (2C), 132.1, 131.7, 131.4, 130.2, 129.8 (2C), 129.5, 129.1, 129.0 (2C), 128.0, 127.6, 126.8, 126.7, 126.0, 125.5, 122.6, 121.1, 54.3, 51.7, 50.6, 41.5, 40.2, 36.3; IR (ATR) ν 3054, 2362, 1736, 1572, 1512, 1473, 1365, 1275,

1217, 1173, 1132 cm^{-1}; HRMS (ESI-TOF) [M + Na]$^+$ calcd for $C_{31}H_{21}Br_2NNaO_3S^+$ m/z 667.9501, found 667.9512.

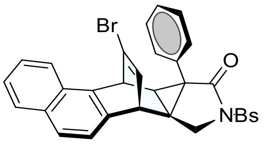

13-Bromo-2-((4-bromophenyl)sulfonyl)-3a-phenyl-1,2,3a,3b,4,11-hexahydro-3*H*-4,11-ethenophenanthro[2',3':1,3]cyclopropa[1,2-c]pyrrol-3-one ((±)-4u')

Prepared according to the general procedure B using **2e** (52.1 mg, 0.1 mmol), 1-(trimethylsilyl)naphthalen-2-yl trifluoromethanesulfonate (43.6 mg, 0.125 mmol) and isolated as white solid (26.5 mg, 41% yield): TLC R_f = 0.25 (*n*-hexane/EtOAc, 3/1, v/v); m.p. 210 ºC (decomp.); ^{1}H NMR (400 MHz, CDCl$_3$) δ 8.06 (d, *J* = 8.7 Hz, 1H), 7.91 (d, *J* = 8.2 Hz, 1H), 7.78 (d, *J* = 8.7 Hz, 2H), 7.71 (d, *J* = 8.2 Hz, 1H), 7.60–7.55 (m, 3H), 7.52–7.47 (m, 3H), 7.43–7.39 (m, 1H), 7.30–7.27 (m, 2H), 6.91–6.89 (m, 1H), 6.16 (dd, *J* = 6.9, 2.3 Hz, 1H), 5.12–5.11 (m, 1H), 4.35 (d, *J* = 6.9 Hz, 1H), 4.13 (d, *J* = 9.6 Hz, 1H), 3.52 (d, *J* = 9.6 Hz, 1H), 1.58 (d, *J* = 4.1 Hz, 1H); ^{13}C NMR (100 MHz, CDCl$_3$) δ 170.9, 142.2, 136.6, 136.4, 132.3 (2C), 132.1, 131.7, 131.5, 130.3, 129.8 (2C), 129.5, 129.0, 128.8, 128.8, 128.1, 127.6, 126.8, 126.1 (2C), 125.8, 125.5, 122.6, 121.9, 54.2, 50.6, 46.7, 45.5, 42.1, 35.7; IR (ATR) ν 3055, 2360, 1734, 1574, 1514, 1473, 1448, 1390,

1365, 1273 cm^{-1}; HRMS (ESI-TOF) [M + Na]$^+$ calcd for $C_{31}H_{21}Br_2NNaO_3S^+$ m/z 667.9501, found 667.9492.

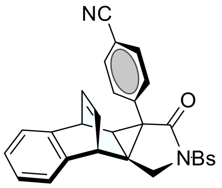

4-(2-((4-Bromophenyl)sulfonyl)-3-oxo-2,3,4,9-tetrahydro-1*H*-4,9-ethenonaphtho[2',3':1,3]cyclopropa[1,2-c]pyrrol-3a(3b*H*)-yl)benzonitrile ((±)-4v)

Prepared according to the general procedure B using **2x** (46.7 mg, 0.1 mmol) and isolated as white solid (52.4 mg, 96% yield): TLC R_f = 0.2 (*n*-hexane/EtOAc, 4/1, v/v); m.p. 190 ºC (decomp.); ^{1}H NMR (600 MHz, $CDCl_3$) δ 7.80 (d, *J* = 8.3 Hz, 2H), 7.63–7.61 (m, 3H), 7.51 (d, *J* = 8.3 Hz, 1H), 7.28 (t, *J* = 6.9 Hz, 2H), 7.26–7.25 (m, 1H), 7.14–7.09 (m, 2H), 7.01 (d, *J* = 8.3 Hz, 1H), 5.83 (t, *J* = 6.9 Hz, 1H), 5.55 (t, *J* = 6.9 Hz, 1H), 4.27 (t, *J* = 4.8 Hz, 1H), 4.13 (d, *J* = 5.5 Hz, 1H), 4.07 (d, *J* = 10.3 Hz, 1H), 3.63 (d, *J* = 10.3 Hz, 1H), 1.60 (d, *J* = 3.4 Hz, 1H); ^{13}C NMR (150 MHz, $CDCl_3$) δ 170.8, 144.2 (2C), 139.5, 136.4, 134.8, 134.4, 132.4 (2C), 132.3, 132.0, 131.4, 131.2, 129.7 (2C), 129.6, 125.9, 125.5, 123.9, 123.7, 118.5, 111.3, 52.0, 50.9, 42.5, 41.3, 41.0, 35.3; IR (ATR) ν 3067, 2958, 2889, 2362, 2253, 2227, 1921, 1727, 1607, 1574 cm^{-1}; HRMS (ESI-TOF) [M + Na]$^+$ calcd for $C_{28}H_{19}BrN_2NaO_3S^+$ m/z 565.0192, found 565.0203.

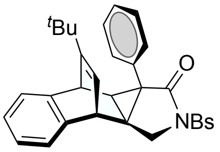

2-((4-Bromophenyl)sulfonyl)-11-(*tert*-butyl)-3a-phenyl-1,2,3a,3b,4,9-hexahydro-3*H*-4,9-ethenonaphtho[2',3':1,3]cyclopropa[1,2-*c*]pyrrol-3-one ((±)-4w)

Prepared according to the general procedure B using **2aa** (49.8 mg, 0.1 mmol) and isolated as white solid (32.2 mg, 56% yield): TLC R_f = 0.6 (*n*-hexane/EtOAc, 5/1, v/v); m.p. >250 ºC); ^{1}H NMR (600 MHz, $CDCl_3$) δ 7.79 (d, *J* = 8.3 Hz, 2H), 7.59 (d, *J* = 8.3 Hz, 2H), 7.34–7.32 (m, 2H), 7.25–7.25 (m, 1H), 7.22–7.18 (m, 3H), 7.08–7.06 (m, 2H), 6.88 (d, *J* = 5.5 Hz, 1H), 5.61 (dd, *J* = 6.9, 2.1 Hz, 1H), 4.18 (dd, *J* = 3.4, 2.1 Hz, 1H), 4.13 (d, *J* = 6.2 Hz, 1H), 4.00 (d, *J* = 9.6 Hz, 1H), 3.54 (d, *J* = 9.6 Hz, 1H), 1.42 (d, *J* = 4.1 Hz, 1H), 0.47 (s, 9H); ^{13}C NMR (150 MHz, $CDCl_3$) δ 171.7, 153.2, 146.9, 145.7, 136.7, 132.3 (2C), 131.5, 131.1, 129.8 (2C), 129.3, 128.1, 127.3, 127.1, 125.9, 125.9, 125.4, 125.1, 123.1 (2C), 51.7, 51.1, 42.6, 42.0, 39.5, 36.1, 34.1, 27.7 (3C); IR (ATR) ν 3063, 2960, 2901, 2352, 2320, 1723, 1575, 1500, 1464, 1446 cm^{-1}; HRMS (ESI-TOF) $[M + Na]^+$ calcd for $C_{31}H_{28}BrNNaO_3S^+$ m/z 596.0865, found 596.0892.

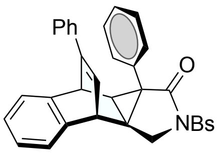

2-((4-Bromophenyl)sulfonyl)-3a,11-diphenyl-1,2,3a,3b,4,9-hexahydro-3*H*-4,9-ethenonaphtho[2',3':1,3]cyclopropa[1,2-*c*]pyrrol-3-one ((±)-4x)

Prepared according to the general procedure B using **2y** (25.9 mg, 0.05 mmol) and isolated as white solid (26.6 mg, 90% yield): TLC R_f = 0.5 (*n*-hexane/EtOAc, 5/1, v/v); m.p. 223–225 ºC (decomp.); ^{1}H NMR (600 MHz, $CDCl_3$) δ 7.81 (d, *J* = 8.3 Hz, 2H), 7.60 (d, *J* = 8.3 Hz, 2H), 7.37 (d, *J* = 8.3 Hz, 1H), 7.31 (d, *J* = 8.3 Hz, 1H), 7.19 (t, *J* = 7.6 Hz, 1H), 7.12 (m, 2H), 7.07 (t, *J* = 7.6 Hz, 1H), 7.02 (t, *J* = 7.6 Hz, 2H), 6.98 (t, *J* = 7.6 Hz, 1H), 6.91 (t, *J* = 8.3 Hz, 2H), 6.78 (t, *J* = 7.6 Hz, 1H), 6.69 (d, *J* = 6.9 Hz, 2H), 6.09 (dd, *J* = 6.2, 2.1 Hz, 1H), 4.72 (m, 1H), 4.25 (d, *J* = 6.2 Hz, 1H), 4.10 (d, *J* = 10.3 Hz, 1H), 3.66 (d, *J* = 10.3 Hz, 1H), 1.62 (d, *J* = 4.1 Hz, 1H); ^{13}C NMR (150 MHz, $CDCl_3$) δ 171.7, 144.8, 144.7, 144.3, 136.7, 135.9, 132.3 (2C), 132.1, 130.8, 130.0, 129.9, 129.8 (2C), 129.4, 128.5, 127.9, 127.7 (2C), 127.4, 127.1, 125.8, 125.5, 124.1 (2C), 123.7, 123.6, 52.1, 50.9, 43.1, 43.0, 40.6, 35.2; IR (ATR) ν 3063, 2959, 2904, 2869, 2357, 2335, 2254, 1728, 1602, 1574 cm^{-1}; HRMS (ESI-TOF) $[M + Na]^+$ calcd for $C_{33}H_{24}BrNNaO_3S^+$ m/z 616.0552, found 616.0563.

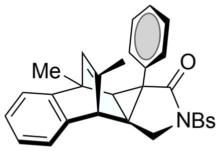

2-((4-Bromophenyl)sulfonyl)-4,10-dimethyl-3a-phenyl-1,2,3a,3b,4,9-hexahydro-3*H*-4,9-ethenonaphtho[2',3':1,3]cyclopropa[1,2-*c*]pyrrol-3-one ((±)-4y)

Prepared according to the general procedure B using **1v** (47.1 mg, 0.1 mmol) and isolated as white solid (36.2 mg, 66% yield): TLC R_f = 0.5 (*n*-hexane/EtOAc, 5/1, v/v); m.p. 198–200 ºC (decomp.); ^{1}H NMR (600 MHz, $CDCl_3$) δ 7.81 (d, *J* = 8.3 Hz, 2H), 7.60 (d, *J* = 8.3 Hz, 2H), 7.33–7.31 (m, 1H), 7.26–7.24 (m, 3H), 7.21–7.20 (m, 2H), 7.16 (t, *J* = 7.6 Hz, 1H), 7.07 (t, *J* = 7.6 Hz, 1H), 6.91–6.90 (m, 1H), 4.87 (s, 1H), 3.98 (d, *J* = 9.6 Hz, 1H), 3.69 (s, 1H), 3.58 (d, *J* = 9.6 Hz, 1H), 1.83 (s, 3H), 1.31 (s, 3H), 1.25 (s, 1H); ^{13}C NMR (150 MHz, $CDCl_3$) δ 172.3, 148.4, 144.9, 142.0, 136.8, 133.5, 133.3, 132.3 (2C), 130.6, 129.8 (2C), 129.3, 129.3, 128.1 (2C), 127.3, 125.7, 124.7, 123.3, 120.3, 52.5, 51.2, 48.2, 44.4, 41.5, 41.3, 20.1, 19.5; IR (ATR) ν 3063, 2966, 2932, 2362, 1727, 1602, 1574, 1499, 1470, 1447 cm^{-1}; HRMS (ESI-TOF) $[M + Na]^+$ calcd for $C_{29}H_{24}BrNNaO_3S^+$ m/z 568.0552, found 568.0558.

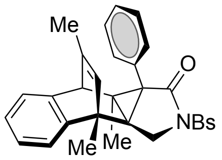

2-((4-Bromophenyl)sulfonyl)-3b,4,10-trimethyl-3a-phenyl-1,2,3a,3b,4,9-hexahydro-3*H*-4,9-ethenonaphtho[2',3':1,3]cyclopropa[1,2-*c*]pyrrol-3-one ((±)-4z)

Prepared according to the general procedure B using **2z** (24.2 mg, 0.05 mmol) and isolated as white solid (25.4 mg, 91% yield): TLC R_f = 0.6 (*n*-hexane/EtOAc, 5/1, v/v); m.p. 222–224 ºC (decomp.); ^{1}H NMR (600 MHz, $CDCl_3$) δ 7.81 (d, *J* = 8.3 Hz, 2H), 7.58 (d, *J* = 8.3 Hz, 2H), 7.29 (t, *J* = 8.3 Hz, 1H), 7.23–7.20 (m, 3H), 7.18–7.16 (m, 2H), 7.10 (dt, *J* = 7.6, 1.4 Hz, 1H), 7.05 (t, *J* = 7.6 Hz, 1H), 6.87 (d, *J* = 8.3 Hz, 1H), 5.19 (s, 1H), 4.31 (d, *J* = 10.3 Hz, 1H), 3.49 (d, *J* = 2.1 Hz, 1H), 3.16 (d, *J* = 10.3 Hz, 1H), 1.87 (s, 3H), 1.00 (d, *J* = 2.1 Hz, 3H) 0.74 (s, 3H); ^{13}C NMR (150 MHz, $CDCl_3$) δ 171.2, 147.7, 145.5, 143.6, 136.7, 136.2, 134.7, 132.2 (2C), 131.1, 130.3, 129.8 (2C), 129.4, 128.2, 127.8, 126.8, 124.9, 124.8, 124.2, 120.2, 54.6, 53.4, 45.3, 45.2, 43.6, 37.0, 19.1, 17.4, 13.1; IR (ATR) ν 3062, 2935, 2357, 2252, 1727, 1601, 1574, 1497, 1471, 1463 cm^{-1}; HRMS (ESI-TOF) $[M + Na]^+$ calcd for $C_{30}H_{26}BrNNaO_3S^+$ m/z 582.0709, found 582.0711.

6. Synthesis and Characterization of Cycloadducts with Arynes (*Conditions D*)

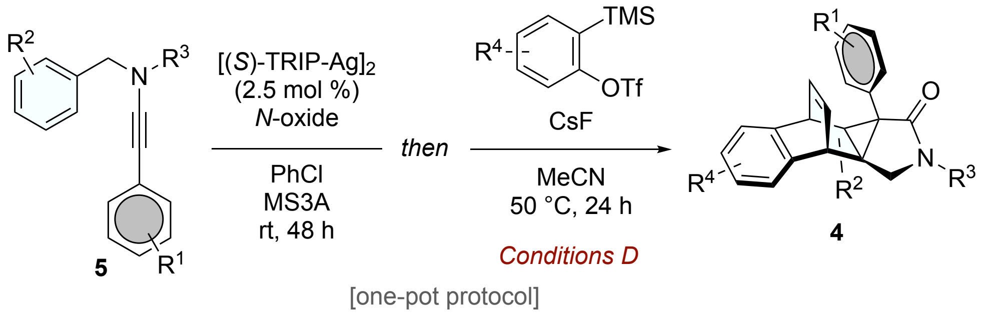

A pre-dried 10-mL test tube equipped with a magnetic stir bar was covered with aluminum foil to avoid light exposure. The test tube was charged with ynamide compound **5** (0.1 mmol, 1 equiv.), 8-methylquinoline *N*-oxide (0.2 mmol, 2 equiv.), [(*S*)-TRIP-Ag]$_2$ (0.025 mmol, 2.5 mol %) and MS3A (100 mg, 1 g/mmol), which were subsequently dissolved partially in dry PhCl (1 mL, 0.1 M) under an argon gas atmosphere. The reaction mixture was stirred for 48 h at room temperature, and then the aluminum foil was removed. 2-(trimethylsilyl)aryl trifluoromethanesulfonate (0.5 mmol, 5 equiv.), CsF (10 mmol, 10 equiv.) and dry MeCN (10 mL, 0.01 M) were added to the reaction mixture, and the reaction mixture was stirred at 50 °C for 24 hours. Subsequently, the reaction mixture was passed through Celite to remove MS3A. After the solvent was evaporated in vacuo, the resulting residue was purified by flash chromatography (*n*-hexane/EtOAc = 5/1, v/v) to afford desired product **4**.

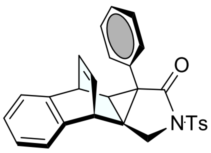

(3b*R*,4*S*,9*S*,9a*R*)-3a-Phenyl-2-tosyl-1,2,3a,3b,4,9-hexahydro-3*H*-4,9-ethenonaphtho[2',3':1,3]cyclopropa[1,2-c]pyrrol-3-one ((+)-4a)

Prepared according to the general procedure D using **5a** (36.1 mg, 0.1 mmol) and isolated (38.1 mg, 84% yield, 90 : 10 er). ^{1}H NMR, ^{13}C NMR, IR, and MS data were identical to the racemic compound.; $[\alpha]^{25}_{D}$ +69.1 (c 0.7, $CHCl_3$). The enantiomeric ratio was determined by analytical chiral HPLC. Retention time: 17.6 min (major isomer), 19.5 min (minor isomer), IH-3 column, 80/20 *n*-hexane/DCM, 1.0 mL/min, 254 nm.

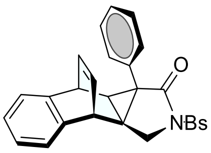

(3b*R*,4*S*,9*S*,9a*R*)-2-((4-Bromophenyl)sulfonyl)-3a-phenyl-1,2,3a,3b,4,9-hexahydro-3*H*-4,9-ethenonaphtho[2',3':1,3]cyclopropa[1,2-c]pyrrol-3-one ((+)-4b)

Prepared according to the general procedure D using **5d** (42.6 mg, 0.1 mmol) and isolated (42.0 mg, 81% yield, 91 : 9 er). ^{1}H NMR, ^{13}C NMR, IR, and MS data were identical to the racemic compound.; $[\alpha]^{25}_{D}$ +63.6 (c 1.5, $CHCl_3$). The enantiomeric ratio was

determined by analytical chiral HPLC. Retention time: 6.2 min (major isomer), 9.1 min (minor isomer), IBN-3 column, 70/30 *n*-hexane/DCM, 1.0 mL/min, 254 nm.

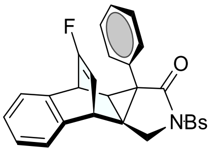

(3b*R*,4*S*,9*S*,9a*R*)-2-((4-Bromophenyl)sulfonyl)-11-fluoro-3a-phenyl-1,2,3a,3b,4,9-hexahydro-3*H*-4,9-ethenonaphtho[2',3':1,3]cyclopropa[1,2-c]pyrrol-3-one ((+)-4c)

Prepared according to the general procedure D using **5u** (44.4 mg, 0.1 mmol) and isolated (29.0 mg, 54% yield, 93 : 7 er). ^{1}H NMR, ^{13}C NMR, ^{19}F NMR, IR, and MS data were identical to the racemic compound.; $[\alpha]^{25}_D$ +74.5 (c 0.7, $CHCl_3$). The enantiomeric ratio was determined by analytical chiral HPLC. Retention time: 16.4 min (minor isomer), 20.3 min (major isomer), IH-3 column, 80/20 *n*-hexane/DCM, 1.0 mL/min, 254 nm.

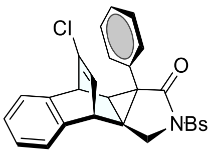

(3b*R*,4*S*,9*S*,9a*R*)-2-((4-Bromophenyl)sulfonyl)-11-chloro-3a-phenyl-1,2,3a,3b,4,9-hexahydro-3*H*-4,9-ethenonaphtho[2',3':1,3]cyclopropa[1,2-c]pyrrol-3-one ((+)-4d)

Prepared according to the general procedure D using **5t** (44.4 mg, 0.1 mmol) and isolated (29.0 mg, 54% yield, 94 : 6 er). ^{1}H NMR, ^{13}C NMR, IR, and MS data were identical to the racemic compound.; $[\alpha]^{25}_{D}$ +106.0 (c 0.8, $CHCl_3$). The enantiomeric ratio was determined by analytical chiral HPLC. Retention time: 16.8 min (minor isomer), 18.8 min (major isomer), IH-3 column, 80/20 *n*-hexane/DCM, 1.0 mL/min, 254 nm.

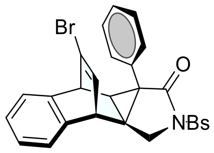

(3b*R*,4*S*,9*S*,9a*R*)-11-Bromo-2-((4-bromophenyl)sulfonyl)-3a-phenyl-1,2,3a,3b,4,9-hexahydro-3*H*-4,9-ethenonaphtho[2',3':1,3]cyclopropa[1,2-c]pyrrol-3-one ((+)-4e)

Prepared according to the general procedure D using **5e** (50.5 mg, 0.1 mmol) and isolated (47.2 mg, 79% yield, 95 : 5 er). ^{1}H NMR, ^{13}C NMR, IR, and MS data were identical to the racemic compound.; $[\alpha]^{25}_{D}$ +57.5 (c 1.3, $CHCl_3$). The enantiomeric ratio was determined by analytical chiral HPLC. Retention time: 16.4 min (major isomer), 36.0 min (minor isomer), IBN-3 column, 80/20 *n*-hexane/DCM, 1.0 mL/min, 254 nm.

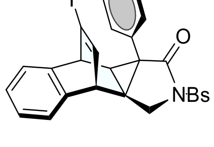

(3b*R*,4*S*,9*S*,9a*R*)-2-((4-Bromophenyl)sulfonyl)-11-iodo-3a-phenyl-1,2,3a,3b,4,9-hexahydro-3*H*-4,9-ethenonaphtho[2',3':1,3]cyclopropa[1,2-c]pyrrol-3-one ((+)-4f)

Prepared according to the general procedure D using **5r** (55.2 mg, 0.1 mmol) and isolated (47.7 mg, 74% yield, 94 : 6 er). ^{1}H NMR, ^{13}C NMR, IR, and MS data were identical to the racemic compound.; $[\alpha]^{25}_D$ +48.8 (c 1.2, $CHCl_3$). The enantiomeric ratio was determined by analytical chiral HPLC. Retention time: 6.7 min (major isomer), 11.8 min (minor isomer), IBN-3 column, 70/30 *n*-hexane/DCM, 1.0 mL/min, 254 nm.

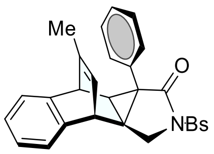

(3b*R*,4*R*,9*S*,9a*R*)-2-((4-Bromophenyl)sulfonyl)-11-methyl-3a-phenyl-1,2,3a,3b,4,9-hexahydro-3*H*-4,9-ethenonaphtho[2',3':1,3]cyclopropa[1,2-c]pyrrol-3-one ((+)-4g)

Prepared according to the general procedure D using **5q** (44.0 mg, 0.1 mmol) and isolated (35.1 mg, 66% yield, 91 : 9 er). ^{1}H NMR, ^{13}C NMR, IR, and MS data were identical to the racemic compound.; $[\alpha]^{25}_D$ +54.6 (c 1.3, $CHCl_3$). The enantiomeric ratio was determined by analytical chiral HPLC. Retention time: 11.5 min (minor isomer), 14.4 min (major isomer), IH-3 column, 80/20 *n*-hexane/DCM, 1.0 mL/min, 254 nm.

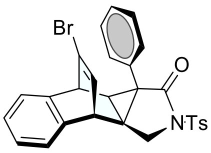

(3b*R*,4*S*,9*S*,9a*R*)-11-Bromo-3a-phenyl-2-tosyl-1,2,3a,3b,4,9-hexahydro-3*H*-4,9-ethenonaphtho[2',3':1,3]cyclopropa[1,2-c]pyrrol-3-one ((+)-4h)

Prepared according to the general procedure D using **5b** (44.0 mg, 0.1 mmol) and isolated (43.7 mg, 82% yield, 92 : 8 er). ^{1}H NMR, ^{13}C NMR, IR, and MS data were identical to the racemic compound.; $[\alpha]^{25}_{D}$ +61.8 (c 1.2, $CHCl_3$). The enantiomeric ratio was determined by analytical chiral HPLC. Retention time: 6.4 min (major isomer), 10.0 min (minor isomer), IBN-3 column, 70/30 *n*-hexane/DCM, 1.0 mL/min, 254 nm.

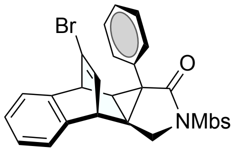

(3b*R*,4*S*,9*S*,9a*R*)-11-Bromo-2-((4-methoxyphenyl)sulfonyl)-3a-phenyl-1,2,3a,3b,4,9-hexahydro-3*H*-4,9-ethenonaphtho[2',3':1,3]cyclopropa[1,2-c]pyrrol-3-one ((+)-4j)

Prepared according to the general procedure D using **5c** (45.6 mg, 0.1 mmol) and isolated (40.6 mg, 74% yield, 92 : 8 er). ^{1}H NMR, ^{13}C NMR, IR, and MS data were identical to the racemic compound.; $[\alpha]^{25}_{D}$ +41.9 (c 1.0, $CHCl_3$). The enantiomeric ratio was

determined by analytical chiral HPLC. Retention time: 25.2 min (minor isomer), 27.6 min (major isomer), IH-3 column, 80/20 *n*-hexane/DCM, 1.0 mL/min, 254 nm.

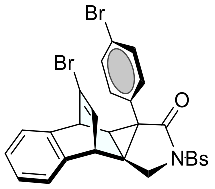

(3b*R*,4*S*,9*S*,9a*R*)-11-Bromo-3a-(4-bromophenyl)-2-((4-bromophenyl)sulfonyl)-1,2,3a,3b,4,9-hexahydro-3*H*-4,9-ethenonaphtho[2',3':1,3]cyclopropa[1,2-c]pyrrol-3-one ((+)-4k)

Prepared according to the general procedure D using **5m** (45.6 mg, 0.1 mmol) and isolated (46.7 mg, 69% yield, 93 : 7 er). ^{1}H NMR, ^{13}C NMR, IR, and MS data were identical to the racemic compound.; $[\alpha]^{25}_{D}$ +46.5 (c 1.4, $CHCl_3$). The enantiomeric ratio was determined by analytical chiral HPLC. Retention time: 18.7 min (minor isomer), 23.9 min (major isomer), IH-3 column, 80/20 *n*-hexane/DCM, 1.0 mL/min, 254 nm.

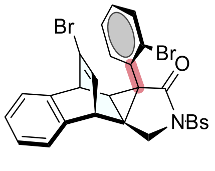

(3b*R*,4*S*,9*S*,9a*R*)-11-Bromo-3a-(2-bromophenyl)-2-((4-bromophenyl)sulfonyl)-1,2,3a,3b,4,9-hexahydro-3*H*-4,9-ethenonaphtho[2',3':1,3]cyclopropa[1,2-c]pyrrol-3-one ((+)-4l)

Prepared according to the general procedure D using **5o** (45.6 mg, 0.1 mmol) and isolated (39.2 mg, 58% yield, 96 : 4 er). ^{1}H NMR, ^{13}C NMR, IR, and MS data were identical to the racemic compound.; $[\alpha]^{25}_{D}$ +74.5 (c 1.2, $CHCl_3$). The enantiomeric ratio was determined by analytical chiral HPLC. Retention time: 14.9 min (major isomer), 31.4 min (minor isomer), IBN-3 column, 80/20 *n*-hexane/DCM, 1.0 mL/min, 254 nm.

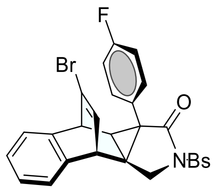

(3b*R*,4*S*,9*S*,9a*R*)-11-Bromo-2-((4-bromophenyl)sulfonyl)-3a-(4-fluorophenyl)-1,2,3a,3b,4,9-hexahydro-3*H*-4,9-ethenonaphtho[2',3':1,3]cyclopropa[1,2-c]pyrrol-3-one ((+)-4m)

Prepared according to the general procedure D using **5l** (52.3 mg, 0.1 mmol) and isolated (39.4 mg, 64% yield, 93 : 7 er). ^{1}H NMR, ^{13}C NMR, ^{19}F NMR, IR, and MS data were identical to the racemic compound.; $[\alpha]^{25}_{D}$ +52.6 (c 1.0, $CHCl_3$). The enantiomeric ratio was determined by analytical chiral HPLC. Retention time: 20.1 min (minor isomer), 22.8 min (major isomer), IH-3 column, 80/20 *n*-hexane/DCM, 1.0 mL/min, 254 nm.

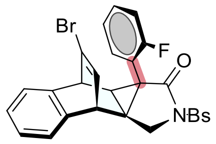

(3b*R*,4*S*,9*S*,9a*R*)-11-Bromo-2-((4-bromophenyl)sulfonyl)-3a-(2-fluorophenyl)-1,2,3a,3b,4,9-hexahydro-3*H*-4,9-ethenonaphtho[2',3':1,3]cyclopropa[1,2-c]pyrrol-3-one ((+)-4n)

major rotamer: Prepared according to the general procedure D using **5j** (52.3 mg, 0.1 mmol) and isolated (33.2 mg, 54% yield, 90 : 10 er). ^{1}H NMR, ^{13}C NMR, ^{19}F NMR, IR, and MS data were identical to the racemic compound.; $[\alpha]^{25}_{D}$ +54.1 (c 0.9, $CHCl_3$). The enantiomeric ratio was determined by analytical chiral HPLC. Retention time: 5.7 min (major isomer), 7.6 min (minor isomer), IBN-3 column, 70/30 *n*-hexane/DCM, 1.0 mL/min, 254 nm.

minor rotamer: Prepared according to the general procedure D using **5j** (52.3 mg, 0.1 mmol) and isolated (19.7 mg, 32% yield, 90 : 10 er). ^{1}H NMR, ^{13}C NMR, ^{19}F NMR, IR, and MS data were identical to the racemic compound.; $[\alpha]^{25}_{D}$ +54.1 (c 0.9, $CHCl_3$). The enantiomeric ratio was determined by analytical chiral HPLC. Retention time: 6.7 min (major isomer), 11.7 min (minor isomer), IBN-3 column, 70/30 *n*-hexane/DCM, 1.0 mL/min, 254 nm.

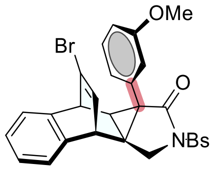

(3b*R*,4*S*,9*S*,9a*R*)-11-Bromo-2-((4-bromophenyl)sulfonyl)-3a-(3-methoxyphenyl)-1,2,3a,3b,4,9-hexahydro-3*H*-4,9-ethenonaphtho[2',3':1,3]cyclopropa[1,2-c]pyrrol-3-one ((+)-4o)

Prepared according to the general procedure D using **5k** (53.5 mg, 0.1 mmol) and isolated (45.8 mg, 73% yield, 93 : 7 er). ^{1}H NMR, ^{13}C NMR, IR, and MS data were identical to the racemic compound.; $[\alpha]^{25}_{D}$ +36.8 (c 1.6, $CHCl_3$). The enantiomeric ratio was determined by analytical chiral HPLC. Retention time: 7.0 min (major isomer), 15.7 min (minor isomer), IBN-3 column, 70/30 *n*-hexane/DCM, 1.0 mL/min, 254 nm.

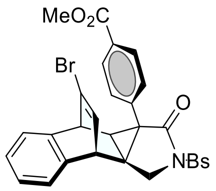

Methyl 4-((3b*R*,4*S*,9*S*,9a*R*)-11-bromo-2-((4-bromophenyl)sulfonyl)-3-oxo-2,3,4,9-tetrahydro-1*H*-4,9-ethenonaphtho[2',3':1,3]cyclopropa[1,2-c]pyrrol-3a(3bH)-yl)benzoate ((+)-4p)

Prepared according to the general procedure D using **5p** (56.3 mg, 0.1 mmol) and isolated (51.1 mg, 78% yield, 94 : 6 er). ^{1}H NMR, ^{13}C NMR, IR, and MS data were identical to

the racemic compound.; $[\alpha]^{25}_{D}$ +37.5 (c 1.0, $CHCl_3$). The enantiomeric ratio was determined by analytical chiral HPLC. Retention time: 22.1 min (minor isomer), 25.3 min (major isomer), IH-3 column, 70/30 *n*-hexane/DCM, 0.5 mL/min, 254 nm.

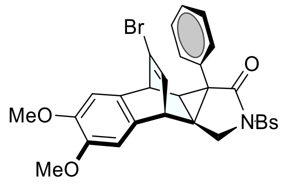

(3b*R*,4*S*,9*S*,9a*R*)-11-Bromo-2-((4-bromophenyl)sulfonyl)-6,7-dimethoxy-3a-phenyl-1,2,3a,3b,4,9-hexahydro-3*H*-4,9-ethenonaphtho[2',3':1,3]cyclopropa[1,2-c]pyrrol-3-one ((+)-4q)

Prepared according to the general procedure D using **5e** (50.5 mg, 0.1 mmol), 4,5-dimethoxy-2-(trimethylsilyl)phenyl trifluoromethanesulfonate (179.2 mg, 0.5 mmol) and isolated (59.2 mg, 90% yield, 94 : 6 er). ^{1}H NMR, ^{13}C NMR, IR, and MS data were identical to the racemic compound.; $[\alpha]^{25}_{D}$ +18.2 (c 2.1, $CHCl_3$). The enantiomeric ratio was determined by analytical chiral HPLC. Retention time: 11.4 min (major isomer), 22.5 min (minor isomer), IBN-3 column, 70/30 *n*-hexane/DCM, 1.0 mL/min, 254 nm.

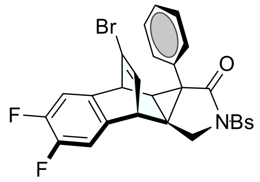

(3b*R*,4*S*,9*S*,9a*R*)-11-Bromo-2-((4-bromophenyl)sulfonyl)-6,7-difluoro-3a-phenyl-1,2,3a,3b,4,9-hexahydro-3*H*-4,9-ethenonaphtho[2',3':1,3]cyclopropa[1,2-c]pyrrol-3-one ((+)-4r)

Prepared according to the general procedure D using **5e** (50.5 mg, 0.1 mmol, 1equiv.). 4,5-difluoro-2-(trimethylsilyl)phenyl trifluoromethanesulfonate (250.7 mg, 0.75 mmol, 7.5 equiv.), CsF (238.7 mg, 1.5 mmol, 15 equiv.) and isolated (46.9 mg, 74% yield, 94 : 6 er). ^{1}H NMR, ^{13}C NMR, ^{19}F NMR, IR, and MS data were identical to the racemic compound.; $[\alpha]^{25}_{D}$ +34.7 (c 2.0, $CHCl_3$). The enantiomeric ratio was determined by analytical chiral HPLC. Retention time: 9.5 min (major isomer), 14.9 min (minor isomer), IBN-3 column, 70/30 *n*-hexane/DCM, 1.0 mL/min, 254 nm.

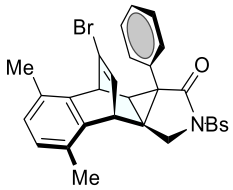

(3b*R*,4*S*,9*S*,9a*R*)-11-Bromo-2-((4-bromophenyl)sulfonyl)-5,8-dimethyl-3a-phenyl-1,2,3a,3b,4,9-hexahydro-3*H*-4,9-ethenonaphtho[2',3':1,3]cyclopropa[1,2-c]pyrrol-3-one ((+)-4s)

Prepared according to the general procedure D using **5e** (50.5 mg, 0.1 mmol), 4,5-dimethyl-2-(trimethylsilyl)phenyl trifluoromethanesulfonate (163.2 mg, 0.5 mmol) and isolated (44.4 mg, 71% yield, 93 : 7 er). ^{1}H NMR, ^{13}C NMR, IR, and MS data were

identical to the racemic compound.; $[\alpha]^{25}_{D}$ +44.2 (c 1.7, $CHCl_3$). The enantiomeric ratio was determined by analytical chiral HPLC. Retention time: 11.2 min (major isomer), 13.5 min (minor isomer), IBN-3 column, 80/20 *n*-hexane/DCM, 1.0 mL/min, 254 nm.

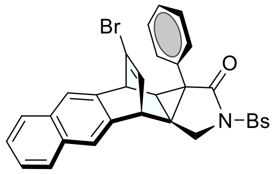

(3b*R*,4*S*,11*S*,11a*R*)-13-Bromo-2-((4-bromophenyl)sulfonyl)-3a-phenyl-1,2,3a,3b,4,11-hexahydro-3*H*-4,11-ethenoanthra[2',3':1,3]cyclopropa[1,2-c]pyrrol-3-one ((+)-4t)

Prepared according to the general procedure D using **5e** (50.5 mg, 0.1 mmol, 1equiv.), 3-(trimethylsilyl)naphthalen-2-yl trifluoromethanesulfonate (261.3 mg, 0.75 mmol, 7.5 equiv.), CsF (238.7 mg, 1.5 mmol, 15 equiv.) and isolated (42.0 mg, 64% yield, 94 : 6 er). ^{1}H NMR, ^{13}C NMR, IR, and MS data were identical to the racemic compound.; $[\alpha]^{25}_{D}$ +33.2 (c 1.6, $CHCl_3$). The enantiomeric ratio was determined by analytical chiral HPLC. Retention time: 21.9 min (major isomer), 47.4 min (minor isomer), IBN-3 column, 80/20 *n*-hexane/DCM, 1.0 mL/min, 254 nm.

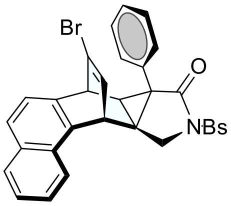

(3b*R*,4*S*,11*R*,11a*S*)-13-Bromo-2-((4-bromophenyl)sulfonyl)-3a-phenyl-1,2,3a,3b,4,11-hexahydro-3*H*-4,11-ethenocyclopenta[2,3]cyclopropa[1,2-b]phenanthren-3-one ((+)-4u)

Prepared according to the general procedure D using **5e** (50.5 mg, 0.1 mmol), 1-(trimethylsilyl)naphthalen-2-yl trifluoromethanesulfonate (174.2 mg, 0.5 mmol) and isolated (30.4 mg, 47% yield, 94 : 6 er). ^{1}H NMR, ^{13}C NMR, IR, and MS data were identical to the racemic compound.; $[\alpha]^{25}_{D}$ +17.3 (c 0.5, $CHCl_3$). The enantiomeric ratio was determined by analytical chiral HPLC. Retention time: 9.6 min (major isomer), 11.9 min (minor isomer), IBN-3 column, 70/30 *n*-hexane/DCM, 1.0 mL/min, 254 nm.

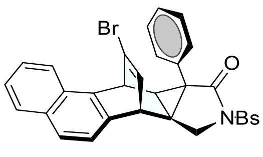

(3b*R*,4*S*,11*S*,11a*R*)-13-Bromo-2-((4-bromophenyl)sulfonyl)-3a-phenyl-1,2,3a,3b,4,11-hexahydro-3*H*-4,11-ethenophenanthro[2',3':1,3]cyclopropa[1,2-c]pyrrol-3-one ((+)-4u')

Prepared according to the general procedure D using **5e** (50.5 mg, 0.1 mmol), 1-(trimethylsilyl)naphthalen-2-yl trifluoromethanesulfonate (174.2 mg, 0.5 mmol) and isolated (25.2 mg, 39% yield, 94 : 6 er). ^{1}H NMR, ^{13}C NMR, IR, and MS data were identical to the racemic compound.; $[\alpha]^{25}_D$ +117.8 (c 0.2, $CHCl_3$). The enantiomeric ratio was determined by analytical chiral HPLC. Retention time: 7.8 min (major isomer), 11.2 min (minor isomer), IBN-3 column, 70/30 *n*-hexane/DCM, 1.0 mL/min, 254 nm.

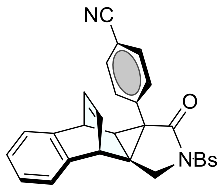

4-((3b*R*,9*R*)-2-((4-Bromophenyl)sulfonyl)-3-oxo-2,3,4,9-tetrahydro-1*H*-4,9-ethenonaphtho[2',3':1,3]cyclopropa[1,2-c]pyrrol-3a(3b*H*)-yl)benzonitrile ((+)-4v)

Prepared according to the general procedure D using **5x** (45.1 mg, 0.1 mmol) and isolated (48.3 mg, 89% yield, 89 : 11 er): ^{1}H NMR, ^{13}C NMR, IR, and MS data were identical to the racemic compound.; $[\alpha]^{25}_D$ +60.3 (c 1.9, $CHCl_3$). The enantiomeric ratio was determined by analytical chiral HPLC. Retention time: 12.2 min (major isomer), 19.7 min (minor isomer), IBN-3 column, 70/30 *n*-hexane/DCM, 1.0 mL/min, 254 nm.

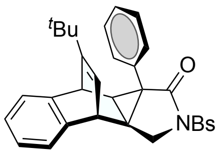

(3b*R*,9*R*)-2-((4-Bromophenyl)sulfonyl)-11-(*tert*-butyl)-3a-phenyl-1,2,3a,3b,4,9-hexahydro-3*H*-4,9-ethenonaphtho[2',3':1,3]cyclopropa[1,2-*c*]pyrrol-3-one ((+)-4w)

Prepared according to the general procedure D using **2aa** (48.2 mg, 0.1 mmol) and isolated as white solid (46 mg, 80% yield, 95 : 5 er): ^{1}H NMR, ^{13}C NMR, IR, and MS data were identical to the racemic compound.; $[\alpha]^{25}_{D}$ +108.2 (c 1.0, $CHCl_3$). The enantiomeric ratio was determined by analytical chiral HPLC. Retention time: 8.9 min (minor isomer), 11.4 min (major isomer), IH-3 column, 80/20 *n*-hexane/DCM, 1.0 mL/min, 254 nm.

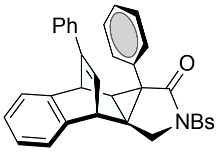

(3b*R*,9*R*)-2-((4-Bromophenyl)sulfonyl)-3a,11-diphenyl-1,2,3a,3b,4,9-hexahydro-3*H*-4,9-ethenonaphtho[2',3':1,3]cyclopropa[1,2-*c*]pyrrol-3-one ((+)-4x)

Prepared according to the general procedure D using **2y** (50.2 mg, 0.1 mmol) and isolated as white solid (47.2 mg, 79% yield, 96 : 4 er): ^{1}H NMR, ^{13}C NMR, IR, and MS data were identical to the racemic compound.; $[\alpha]^{25}_{D}$ +18.5 (c 1.0, $CHCl_3$). The enantiomeric ratio

was determined by analytical chiral HPLC. Retention time: 14.7 min (major isomer), 27.9 min (minor isomer), IBN-3 column, 80/20 *n*-hexane/DCM, 1.0 mL/min, 254 nm.

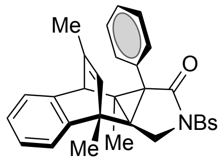

(3b*S*,9*R*)-2-((4-Bromophenyl)sulfonyl)-3b,4,10-trimethyl-3a-phenyl-1,2,3a,3b,4,9-hexahydro-3*H*-4,9-ethenonaphtho[2',3':1,3]cyclopropa[1,2-*c*]pyrrol-3-one ((+)-4z)

Prepared according to the general procedure D using **2z** (46.8 mg, 0.1 mmol) and isolated as white solid (39.0 mg, 70% yield, 97 : 3 er). The reaction time in the first step to synthesize CHT/NCD is 72 h.: ^{1}H NMR, ^{13}C NMR, IR, and MS data were identical to the racemic compound.; $[\alpha]^{25}_{D}$ +43.3 (c 1.0, $CHCl_3$). The enantiomeric ratio was determined by analytical chiral HPLC. Retention time: 9.4 min (major isomer), 12.6 min (minor isomer), IBN-3 column, 80/20 *n*-hexane/DCM, 1.0 mL/min, 254 nm.

The procedure for gram-scale cycloaddition reaction with Aryne

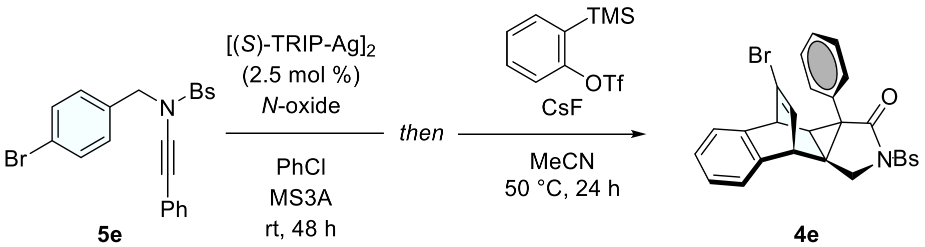

5e 1.26 g

4e 1.22 g (82% yield, 95 : 5 er)

A pre-dried 300 mL flask equipped with a magnetic stir bar was covered with aluminum foil to avoid light exposure. The flask was charged with ynamide compound **5e** (2.5 mmol, 1 equiv.), 8-methylquinoline *N*-oxide (5 mmol, 2 equiv.), [(*S*)-TRIP-Ag]$_2$ (0.00625 mmol, 2.5 mol %) and MS3A (2.5 g, 1 g/mmol), which were subsequently dissolved partially in dry PhCl (25 mL, 0.1 M) under an argon gas atmosphere. The reaction mixture was stirred for 48 h at room temperature, and then the aluminum foil was removed. 2-(trimethylsilyl)phenyl trifluoromethanesulfonate (12.5 mmol, 5 equiv.), CsF (25 mmol, 10 equiv.) and dry MeCN (250 mL, 0.01 M) were added to the reaction mixture, and the reaction mixture was stirred at 50 °C for 24 hours. Subsequently, the reaction mixture was passed through Celite to remove MS3A. After the solvent was evaporated in vacuo, the resulting residue was purified by flash chromatography (*n*-hexane/DCM = 1/1, v/v) to afford desired product **4e** (1224.5 mg, 82% yield, 95 : 5 er).

7. Characterization of Derivatives

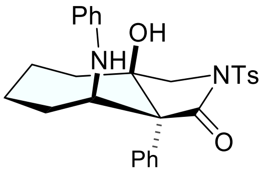

(3a*R*,8*S*,8a*S*)-3a-Hydroxy-8a-phenyl-8-(phenylamino)-2-tosyloctahydrocyclohepta[c]pyrrol-1(2*H*)-one ((–)-6)

(+)-**3a** (24.2 mg, 0.05 mmol) and 10% Pd/C (4.8 mg, 20 w/w %) were suspended in EtOH (0.5 mL, 0.1 M) under H_2 gas atmosphere. The reaction mixture was stirred at rt for 24 hours. After that, the reaction mixture was passed through Celite to remove the Pd/C. After the solvent was evaporated in *vacuo*, the resulting residue was purified by flash chromatography to give yellow solid (19.6 mg, 80% yield). TLC R_f = 0.1 (*n*-hexane/EtOAc, 3/1, v/v); m.p. 148–149 ºC; ^{1}H NMR (600 MHz, $CDCl_3$) δ 7.72 (d, *J* = 6.9 Hz, 2H), 7.42 (d, *J* = 7.6 Hz, 2H), 7.27–7.22 (m, 5H), 7.16 (d, *J* = 7.6 Hz, 2H), 6.98 (d, *J* = 6.9 Hz, 3H), 4.28 (s, 1H), 3.85 (s, 2H), 2.37 (s, 3H), 2.28–2.23 (m, 1H), 2.04 (m, 2H), 1.71–1.50 (m, 5H), 1.34–1.20 (m, 2H); ^{13}C NMR (150 MHz, $CDCl_3$) δ173.7, 145.8, 144.8, 134.8, 134.5, 129.4 (2C), 129.0 (2C), 128.8 (2C), 127.9, 127.9 (2C), 127.6 (2C), 122.3, 121.1 (2C), 81.2, 65.5, 62.2, 59.1, 35.6, 29.8, 26.4, 21.6, 20.9; IR (ATR) ν 3420, 2923, 2861, 2362, 2339, 1724, 1600, 1508, 1444, 1362 cm^{-1}; HRMS (ESI-TOF) [M + H]$^+$ calcd for $C_{28}H_{31}N_2O_4S^+$ m/z 491.1999, found 491.1999; $[\alpha]^{25}_D$ –1.0 (c 0.28, $CHCl_3$).

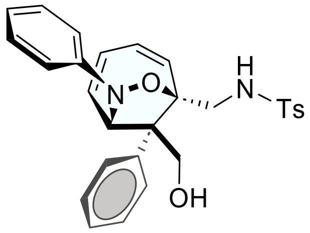

N-(((1S,6R,9R)-9-(Hydroxymethyl)-8,9-diphenyl-7-oxa-8-azabicyclo[4.2.1]nona-2,4-dien-6-yl)methyl)-4-methylbenzenesulfonamide ((+)-7)

A pre-dried 10 mL test tube equipped with a magnetic stir bar was charged with (+)-**3a** (24.2 mg, 0.05 mmol) and dry THF (0.33 mL, 0.15 M) under an argon gas atmosphere. After the solution was cooled to 0 ºC, $LiAlH_4$ solution in THF (1M, 0.2 mL, 0.2 mmol) was added to the test tube. The reaction mixture was warmed to rt and stirred for 1 hour. The reaction mixture was diluted with Et_2O and quenched with a saturated solution of NH_4Cl. Then, the aqueous layer was extracted with EtOAc three times. The combined organic layers were washed with brine, dried over Na_2SO_4, and concentrated under reduced pressure. Finally, the residue was purified by flash chromatography to give yellow solid (22.3 mg, 90% yield). TLC R_f = 0.2 (*n*-hexane/EtOAc, 2/1, v/v); m.p. 85 ºC (decomp.); ^{1}H NMR (400 MHz, $CDCl_3$) δ 7.80 (d, *J* = 8.2 Hz, 2H), 7.33 (d, *J* = 8.2 Hz, 2H), 7.28–7.21 (m, 7H), 7.02–6.97 (m, 3H), 6.12 (d, *J* = 11.4 Hz, 1H), 5.73–5.56 (m, 3H), 5.16 (m, 1H), 4.77 (d, *J* = 6.4 Hz, 1H), 4.03 (d, *J* = 11.4, 5.0 Hz, 1H), 3.88–3.72 (m, 3H), 3.00 (t, *J* = 6.0 Hz, 1H), 2.42 (s, 3H); ^{13}C NMR (100 MHz, $CDCl_3$) δ146.9, 143.6, 138.3,

136.9, 133.6, 129.9 (2C), 129.6, 129.5, 128.7 (4C), 128.4 (2C), 127.4 (2C), 127.1, 126.9 (2C), 122.6, 116.1 (2C), 83.9, 68.9, 68.7, 59.0, 48.4, 21.5; IR (ATR) ν 3280, 3032, 2922, 2360, 2322, 1705, 1596, 1489, 1445, 1427 cm^{-1}; HRMS (ESI-TOF) $[M + Na]^+$ calcd for $C_{28}H_{28}N_2NaO_4S^+$ m/z 511.1662, found 511.1657; $[\alpha]^{25}_D$ +273.3 (c 0.54, $CHCl_3$).

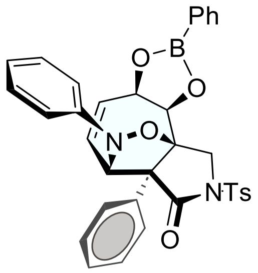

(3a*R*,6*S*,6a*S*,9a*R*,9b*R*)-2,6a,11-Triphenyl-8-tosyl-3a,6,6a,8,9,9b-hexahydro-7*H*-9a,6-(epoxyimino)[1,3,2]dioxaborolo[4',5':3,4]cyclohepta[1,2-c]pyrrol-7-one ((+)-8)

(+)-**3a** (48.5 mg, 0.1 mmol), *N*-methylmorpholine *N*-oxide (25.8 mg, 0.22 mmol), and $PhB(OH)_2$ (26.8 mg, 0.22 mmol) were suspended in DCM (2 mL, 0.05 M) under an argon gas atmosphere. After OsO_4 in *t*-BuOH (0.064 mL, 5 mol %) was added to the reaction mixture, the whole was stirred at rt for 24 hours. The reaction mixture was quenched with 1M $Na_2S_2O_3$ aqueous solution. Then, the aqueous layer was extracted with DCM three times. The combined organic layers were dried over Na_2SO_4 and concentrated under reduced pressure. Finally, the residue was purified by flash chromatography to give yellow solid (54.6 mg, 90% yield). TLC R_f = 0.2 (*n*-hexane/EtOAc, 3/1, v/v); m.p. > 200

ºC; ^{1}H NMR (600 MHz, $CDCl_3$) δ 7.85-7.83 (m, 4H), 7.53 (t, *J* = 7.3 Hz, 1H), 7.43 (t, *J* = 7.3 Hz, 2H), 7.33–7.30 (m, 3H), 7.17–7.11 (m, 4H), 7.02 (d, *J* = 8.3 Hz, 2H) 6.93 (t, *J* = 7.6 Hz, 1H), 6.67 (d, *J* = 7.6 Hz, 2H), 5.91 (dd, *J* = 12.4, 3.4 Hz, 1H), 5.52 (ddd, *J* = 12.4, 6.2, 2.1 Hz, 1H), 4.99 (d, *J* = 6.2 Hz, 1H), 4.85 (d, *J* = 8.3 Hz, 1H), 4.73 (d, *J* = 12.4 Hz, 1H), 4.57 (d, *J* = 12.4 Hz, 1H), 4.52 (ddd, *J* = 8.3, 3.4, 2.1 Hz, 1H), 2.17 (s, 3H); ^{13}C NMR (100 MHz, $CDCl_3$) δ 172.2, 145.3, 145.2, 135.0 (2C), 134.1, 133.6, 133.0, 131.8, 131.2, 129.3 (2C), 129.2 (2C), 128.7, 128.3 (2C), 127.9 (2C), 127.9 (2C), 127.9 (2C), 122.9, 120.5, 117.0 (2C), 83.9, 79.0, 75.9, 68.8, 66.0, 56.5, 21.5; IR (ATR) ν 3028, 2361, 2340, 1746, 1598, 1489, 1439, 1403, 1366, 1298 cm^{-1}; HRMS (ESI-TOF) [M + Na]$^+$ calcd for $C_{34}H_{29}BN_2NaO_6S^+$ m/z 627.1732, found 627.1733; $[\alpha]^{25}_D$ +140.9 (c 0.72, $CHCl_3$).

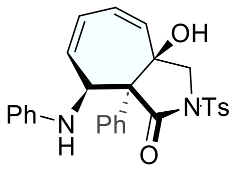

(3a*R*,8*S*,8a*S*)-3a-Hydroxy-8a-phenyl-8-(phenylamino)-2-tosyl-3,3a,8,8a-tetrahydrocyclohepta[c]pyrrol-1(2*H*)-one ((+)-9)

(+)-**3a** (24.2 mg, 0.05 mmol), Zn powder (327 mg, 5.0 mmol), and $AlCl_3·6H_2O$ (60.4 mg, 0.25 mmol) were suspended in THF (1 mL, 0.05 M) under an argon gas atmosphere. The

whole was stirred at 50 ºC for 22 hours. The reaction mixture was poured into a saturated solution of NH_4Cl. Then, the aqueous layer was extracted with EtOAc three times. The combined organic layers were washed with brine, dried over Na_2SO_4, and concentrated under reduced pressure. Finally, the residue was purified by flash chromatography to give white solid (21.0 mg, 86% yield). TLC R_f = 0.1 (*n*-hexane/EtOAc, 2/1, v/v); m.p. 192–193 ºC; ^{1}H NMR (600 MHz, $CDCl_3$) δ 7.90 (d, *J* = 8.3 Hz, 2H), 7.28 (d, *J* = 8.3 Hz, 2H), 7.21–7.18 (m, 1H), 7.15–7.13 (m, 2H), 7.10–7.06 (m, 4H), 6.75 (t, *J* = 7.6 Hz, 1H), 6.67 (d, *J* = 8.3 Hz, 2H), 6.04 (d, *J* = 11.7, 4.8 Hz, 1H), 5.98 (dd, *J* = 11.0, 6.9 Hz, 1H), 5.93 (dd, *J* = 11.7, 6.9 Hz, 1H), 5.86 (d, *J* = 11.0 Hz, 1H), 4.81 (brs, 1H), 4.66 (d, *J* = 4.8 Hz, 1H), 4.18 (d, *J* = 10.3 Hz, 1H), 3.94(d, *J* = 10.3 Hz, 1H), 3.84 (brs, 1H), 2.44 (s, 3H); ^{13}C NMR (150 MHz, $CDCl_3$) δ 172.0, 146.2, 145.1, 136.8, 136.1, 134.9, 131.7, 129.6 (2C), 129.1 (2C), 128.8, 128.5 (2C), 128.2, 128.1 (2C), 126.2 (2C), 123.8, 119.1, 115.8 (2C), 78.1, 67.3, 59.8, 56.8, 21.7; IR (ATR) ν 3427, 3012, 2361, 2322, 1730, 1598, 1502, 1433, 1355, 1235 cm^{-1}; HRMS (ESI-TOF) $[M + H]^+$ calcd for $C_{28}H_{27}N_2O_4S^+$ m/z 487.1686, found 487.1699; $[\alpha]^{25}_D$ +403.9 (c 0.15, $CHCl_3$).

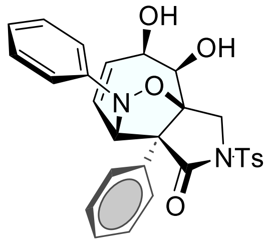

(3a*R*,4*R*,5*R*,8*S*,8a*S*)-4,5-Dihydroxy-8a,9-diphenyl-2-tosyl-2,3,4,5,8,8a-hexahydro-1*H*-3a,8-(epoxyimino)cyclohepta[c]pyrrol-1-one ((+)-10)

(+)-**3a** (126.0 mg, 0.26 mmol), and *N*-methylmorpholine *N*-oxide (30.5 mg, 0.26 mmol) were suspended in THF/^{t}BuOH/H_2O (6 : 8 : 1, 0.06 M) under an argon gas atmosphere. Then, OsO_4 in ^{t}BuOH (0.17 mL, 5 mol %) was added to the reaction mixture, and the whole was stirred at rt for 2 hours. The reaction mixture was quenched with 1 M $Na_2S_2O_3$ aqueous solution. Then, the aqueous layer was extracted with EtOAc three times. The combined organic layers were washed with brine, dried over Na_2SO_4 and concentrated under reduced pressure. Finally, the residue was purified by flash chromatography to give white solid (111.2 mg, 82% yield). TLC R_f = 0.1 (*n*-hexane/EtOAc, 3/2, v/v); m.p. 120–122 ºC; ^{1}H NMR (400 MHz, $CDCl_3$) δ 7.84 (d, *J* = 8.7 Hz, 2H), 7.30–7.26 (m, 3H), 7.19–7.10 (m, 6H), 6.95 (t, *J* = 7.3 Hz, 1H), 6.62 (d, *J* = 8.7 Hz, 2H), 6.08 (dd, *J* = 11.9, 6.9 Hz, 1H), 5.94 (dd, *J* = 11.9, 6.9 Hz, 1H), 5.11 (d, *J* = 6.9 Hz, 1H), 4.52 (s, 2H), 4.09 (dd, *J* = 10.1, 5.0 Hz, 1H), 3.99–3.93 (m, 1H), 3.52 (d, *J* = 10.1 Hz, 1H), 3.09 (d, *J* = 11.9 Hz, 1H), 2.34 (s, 3H); ^{13}C NMR (100 MHz, $CDCl_3$) δ 171.3, 147.1, 145.2, 134.2, 132.4, 132.3,

129.8, 129.6 (2C), 129.4 (2C), 128.9 (2C), 128.6, 127.9 (2C), 127.8 (2C), 122.9, 115.1 (2C), 89.4, 69.7, 68.6, 68.0, 65.6, 53.5, 21.6; IR (ATR) ν 3524, 3062, 2922, 2339, 1744, 1707, 1596, 1491, 1469, 1449 cm^{-1}; HRMS (ESI-TOF) [M + H]$^+$ calcd for $C_{28}H_{27}N_2O_6S^+$ m/z 519.1584, found 519.1583; $[\alpha]^{25}_D$ +15.4 (c 0.28, $CHCl_3$).

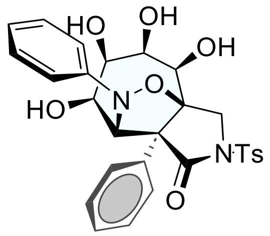

(3a*R*,4*R*,5*R*,6*R*,7*R*,8*R*,8a*S*)-4,5,6,7-Tetrahydroxy-8a,9-diphenyl-2-tosyloctahydro-1*H*-3a,8-(epoxyimino)cyclohepta[c]pyrrol-1-one ((+)-11)

(+)-**10** (37.3 mg, 0.072 mmol) and *N*-methylmorpholine *N*-oxide (10.2 mg, 0.086 mmol) were suspended in THF/^{t}BuOH/H_2O (6 : 8 : 1, 0.06 M) under an argon gas atmosphere. After OsO_4 in ^{t}BuOH (0.046 mL, 5 mol %) was added to the reaction mixture, the whole was stirred at rt for 48 hours. The reaction mixture was quenched with 1 M $Na_2S_2O_3$ aqueous solution. Then, the aqueous layer was extracted with EtOAc three times. The combined organic layers were washed with brine, dried over Na_2SO_4 and concentrated under reduced pressure. Finally, the residue was purified by flash chromatography to give white solid (22.8 mg, 57% yield). TLC R_f = 0.2 (*n*-hexane/EtOAc, 1/4, v/v); m.p. 174 ºC (decomp.); ^{1}H NMR (600 MHz, $CDCl_3$) δ 7.74 (d, *J* = 8.3 Hz, 2H), 7.36–7.35 (m, 3H),

7.25–7.23 (m, 4H), 6.98–6.91 (m, 3H), 6.80 (d, J = 8.3 Hz, 2H), 4.99 (d, J = 4.1 Hz, 1H), 4.45 (d, J = 12.4 Hz, 1H), 4.40 (d, J = 12.4 Hz, 1H), 4.38–4.33 (m, 2H), 4.16 (dd, J = 11.0, 4.8 Hz, 1H), 3.80 (dd, J = 10.3, 3.4 Hz, 1H), 3.43 (d, J = 10.3 Hz, 1H), 3.33 (d, J = 7.6 Hz, 1H), 3.27 (d, J = 6.2 Hz, 2H), 2.48 (s, 3H); ^{13}C NMR (150 MHz, $CDCl_3$) δ 169.8, 149.1, 145.4, 133.9, 131.1, 130.0 (2C), 129.8 (2C), 129.4, 128.6 (2C), 128.1 (2C), 126.8, 123.3 (2C), 115.4 (2C), 86.9, 75.1, 71.9, 70.5, 69.5, 68.9, 65.6, 52.3, 21.8; IR (ATR) ν 3419, 2923, 2360, 2339, 1747, 1707, 1596, 1489, 1449, 1364 cm^{-1}; HRMS (ESI-TOF) $[M + Na]^+$ calcd for $C_{28}H_{28}N_2NaO_8S^+$ m/z 575.1459, found 575.1465; $[\alpha]^{25}_D$ +9.3 (c 0.76, $CHCl_3$).

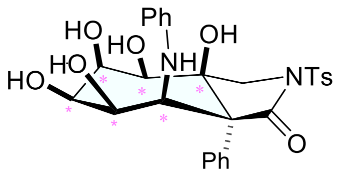

(3a*R*,4*R*,5*R*,6*R*,7*R*,8*R*,8a*S*)-3a,4,5,6,7-Pentahydroxy-8a-phenyl-8-(phenylamino)-2-tosyloctahydrocyclohepta[c]pyrrol-1(2*H*)-one ((+)-12)

(+)-**11** (27.6 mg, 0.05 mmol), Zn powder (327 mg, 5.0 mmol) and $AlCl_3 \cdot 6H_2O$ (120.7 mg, 0.5 mmol) were suspended in THF (1 mL, 0.05 M) under an argon gas atmosphere. After the whole was stirred at 50 ºC for 22 hours, the reaction mixture was poured into a saturated solution of NH_4Cl. Then the aqueous layer was extracted with EtOAc three

times. The combined organic layers were washed with brine and dried over Na_2SO_4 and concentrated under reduced pressure. Finally, the residue was purified by flash chromatography to give white solid (22.0 mg, 79% yield). TLC $R_f = 0.1$ (*n*-hexane/EtOAc, 1/5, v/v); m.p. 141 ºC (decomp.); ^1H NMR (600 MHz, CD_3OD) δ 7.64 (d, *J* = 8.3 Hz, 2H), 7.42 (d, *J* = 7.6 Hz, 2H), 7.30–7.29 (m, 3H), 7.24 (d, *J* = 7.6 Hz, 2H), 7.08 (t, *J* = 7.6 Hz, 2H), 6.66 (d, *J* = 7.6 Hz, 1H), 6.64 (d, *J* =8.3 Hz, 2H), 4.81 (d, *J* = 6.2 Hz, 1H), 4.47 (d, *J* = 5.5 Hz, 1H), 4.27 (d, *J* = 11.0 Hz, 1H), 4.16 (d, *J* = 11.0 Hz, 1H), 3.97 (s, 1H), 3.66 (dd, *J* = 5.5, 2.8 Hz, 1H), 3.59 (d, *J* = 5.5 Hz, 1H), 2.42 (s, 3H); ^{13}C NMR (150 MHz, CD_3OD) δ 173.1, 151.2, 146.6, 136.3, 135.8, 130.6 (2C), 130.4 (2C), 129.7 (2C), 129.6, 128.9 (2C), 128.6 (2C), 118.9, 115.7 (2C), 81.3, 80.8, 72.4, 72.2, 71.6, 64.5, 63.0, 59.7, 21.6; IR (ATR) ν 3398, 3056, 2922, 2360, 2339, 1744, 1704, 1599, 1498, 1442 cm^{-1}; HRMS (ESI-TOF) $[M + Na]^+$ calcd for $C_{28}H_{30}N_2NaO_8S^+$ m/z 577.1615, found 577.1637; $[\alpha]^{25}_D$ +4.6 (c 0.15, $CHCl_3$).

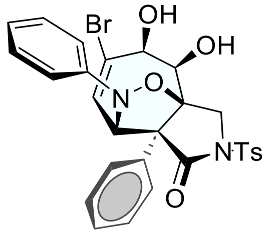

(3a*R*,4*R*,5*S*,8*S*,8a*S*)-6-Bromo-4,5-dihydroxy-8a,9-diphenyl-2-tosyl-2,3,4,5,8,8a-hexahydro-1*H*-3a,8-(epoxyimino)cyclohepta[c]pyrrol-1-one ((+)-13)

(+)-**3b** (563.5 mg, 1 mmol) and *N*-methylmorpholine *N*-oxide (117.2 mg, 1 mmol) were suspended in THF/*t*-BuOH/H_2O (6 : 8 : 1, 0.06 M) under an argon gas atmosphere. After OsO_4 in ^{t}BuOH (0.32 mL, 5 mol %) was added to the reaction mixture, the whole was stirred at rt for 5 hours. The reaction mixture was quenched with 1M $Na_2S_2O_3$ aqueous solution. Then, the aqueous layer was extracted with EtOAc three times. The combined organic layers were washed with brine and dried over Na_2SO_4 and concentrated under reduced pressure. Finally, the residue was purified by flash chromatography to give yellow solid (467.7 mg, 78% yield). TLC R_f = 0.1 (*n*-hexane/EtOAc, 2/1, v/v); m.p. 130–132 ºC; ^{1}H NMR (400 MHz, $CDCl_3$) δ 7.83 (d, *J* = 8.2 Hz, 2H), 7.35 (d, *J* = 6.9 Hz, 3H), 7.22 (d, *J* = 8.2 Hz, 2H), 7.15–7.09 (m, 4H), 6.99 (t, *J* = 7.3 Hz, 1H), 6.60 (dd, *J* = 7.3, 5.5 Hz, 3H), 5.00 (d, *J* = 7.3 Hz, 1H), 4.53 (d, *J* = 12.8, 1H), 4.46 (d, *J* = 12.8, 1H), 4.27 (d, *J* = 10.5 Hz, 2H), 3.67 (d, *J* = 11.4 Hz, 1H), 3.43 (d, *J* = 11.4 Hz, 1H), 2.38 (s, 3H); ^{13}C NMR (100 MHz, $CDCl_3$) δ 170.6, 146.8, 145.4, 134.1, 132.6, 131.7, 129.7 (4C), 129.0 (2C), 128.9, 128.0 (2C), 127.7 (2C), 127.6, 123.5, 115.3 (2C), 89.6, 77.8, 69.6, 67.9, 67.2, 52.7, 21.7; IR (ATR) ν 3849, 3519, 2959, 2364, 2350, 2332, 1746, 1596, 1490, 1364 cm^{-1}; HRMS (ESI-TOF) $[M + Na]^+$ calcd for $C_{28}H_{25}BrN_2NaO_6S^+$ m/z 619.0509, found 619.0519; $[\alpha]^{25}_D$ +46.0 (c 0.28, $CHCl_3$).

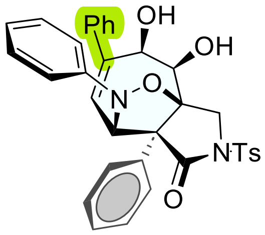

(3a*R*,4*R*,5*R*,8*S*,8a*S*)-4,5-Dihydroxy-6,8a,9-triphenyl-2-tosyl-2,3,4,5,8,8a-hexahydro-1*H*-3a,8-(epoxyimino)cyclohepta[c]pyrrol-1-one ((+)-14)

(+)-**13** (17.9 mg, 0.03 mmol), $Pd_2(dba)_3$ (2.7 mg, 0.003 mmol), $PhB(OH)_2$ (7.3 mg, 0.06 mmol), and Na_2CO_3 (9.5 mg, 0.09 mmol) were suspended in THF/H_2O (1 : 1, 0.05 M) under an argon gas atmosphere. The reaction mixture was stirred at 80 °C for 7 hours. The reaction mixture was quenched with 1 M HCl aqueous solution and passed through Celite to remove Pd catalyst. After that, the mixture was extracted with DCM three times. The combined organic layers were washed with brine, dried over Na_2SO_4 and concentrated under reduced pressure. Finally, the residue was purified by flash chromatography to give yellow solid (45.8 mg, 86% yield). TLC R_f = 0.2 (*n*-hexane/EtOAc, 2/1, v/v); m.p. 140–142 °C; ^{1}H NMR (400 MHz, $CDCl_3$) δ 7.88 (d, *J* = 8.2 Hz, 2H), 7.38–7.18 (m, 10H), 7.05–6.96 (m, 5H), 6.52 (d, *J* = 8.2 Hz, 2H), 6.24 (d, *J* = 6.9 Hz, 1H), 5.11 (d, *J* = 6.9 Hz, 1H), 4.62 (d, *J* = 12.4 Hz, 1H), 4.56 (d, *J* = 12.8 Hz, 1H), 4.47–4.42 (m, 2H), 4.35 (dd, *J* = 11.0, 4.6 Hz, 1H), 3.65 (d, *J* = 10.5 Hz, 1H), 2.49 (s, 3H); ^{13}C NMR (100 MHz, $CDCl_3$) δ170.9, 148.4, 145.2, 144.7, 142.6, 134.3, 132.6, 130.6, 129.8 (2C), 129.5 (2C), 128.8 (2C), 128.6, 128.4 (2C), 128.2 (2C), 128.0 (3C), 125.8 (2C), 123.5, 115.3 (2C), 90.1, 74.0,

69.3, 68.7, 66.9, 51.6, 21.8; IR (ATR) ν 3522, 3345, 3030, 2925, 2363, 2356, 2338, 1986, 1597, 1490 cm^{-1}; HRMS (ESI-TOF) $[M + Na]^+$ calcd for $C_{34}H_{30}N_2NaO_6S^+$ m/z 617.1717, found 617.1731; $[\alpha]^{25}{}_D$ +100.9 (c 1.3, $CHCl_3$).

8. Single Crystal X-Ray Diffraction Analysis

Supplementary Table 1. Crystal data and structure refinement for **3a**.

A. Crystal Data

Empirical Formula	$C_{28}H_{24}N_2O_4S$
Formula Weight	484.57
Crystal Color, Habit	colorless, block
Crystal Dimensions	0.100 X 0.100 X 0.100 mm
Crystal System	orthorhombic
Lattice Type	Primitive
Lattice Parameters	a = 9.06167(17) Å<br>b = 16.0681(3) Å<br>c = 16.3670(3) Å<br>V = 2383.10(8) Å^3
Space Group	$P2_12_12_1$ (#19)
Z value	4
D_{calc}	1.350 g/cm^3
F_{000}	1016.00
m(CuKa)	15.222 cm^{-1}

B. Intensity Measurements

Diffractometer	R-AXIS RAPID
Radiation	CuKa (l = 1.54187 Å)<br>multi-layer mirror monochromated
Voltage, Current	40kV, 30mA
Temperature	23.0^{o}C
Detector Aperture	460.0 x 256.0 mm
Data Images	45 exposures
w oscillation Range (c=54.0, f=0.0)	80.0 - 260.0^{o}
Exposure Rate	1.0 sec./^{o}
w oscillation Range (c=54.0, f=90.0)	80.0 - 260.0^{o}
Exposure Rate	1.0 sec./^{o}
w oscillation Range (c=54.0, f=180.0)	80.0 - 260.0^{o}
Exposure Rate	1.0 sec./^{o}
w oscillation Range (c=54.0, f=270.0)	80.0 - 260.0^{o}
Exposure Rate	1.0 sec./^{o}
w oscillation Range (c=0.0, f=0.0)	80.0 - 260.0^{o}
Exposure Rate	1.0 sec./^{o}

Detector Position	127.40 mm
Pixel Size	0.100 mm
$2q_{max}$	136.3^{o}
No. of Reflections Measured	Total: 27508<br>Unique: 4352 (R_{int} = 0.0357)<br>Friedel pairs: 1872
Corrections	Lorentz-polarization<br>Absorption<br>(trans. factors: 0.163 - 0.218)

C. Structure Solution and Refinement

Structure Solution	Direct Methods
Refinement	Full-matrix least-squares on F^2
Function Minimized	$S w (Fo^2 - Fc^2)^2$
Least Squares Weights	$w = 1/ [s^2(Fo^2) + (0.1233 \cdot P)^2$ $+ 0.4400 \cdot P]$ where $P = (Max(Fo^2,0) + 2Fc^2)/3$
$2q_{max}$ cutoff	136.3^o
Anomalous Dispersion	All non-hydrogen atoms
No. Observations (All reflections)	4352
No. Variables	316
Reflection/Parameter Ratio	13.77
Residuals: R1 (I>2.00s(I))	0.0347
Residuals: R (All reflections)	0.0428
Residuals: wR2 (All reflections)	0.1151
Goodness of Fit Indicator	0.684
Flack Parameter (Friedel pairs = 1872)	0.010(18)
Max Shift/Error in Final Cycle	0.000

Maximum peak in Final Diff. Map 0.14 e⁻/Å^3

Minimum peak in Final Diff. Map -0.25 e⁻/Å^3

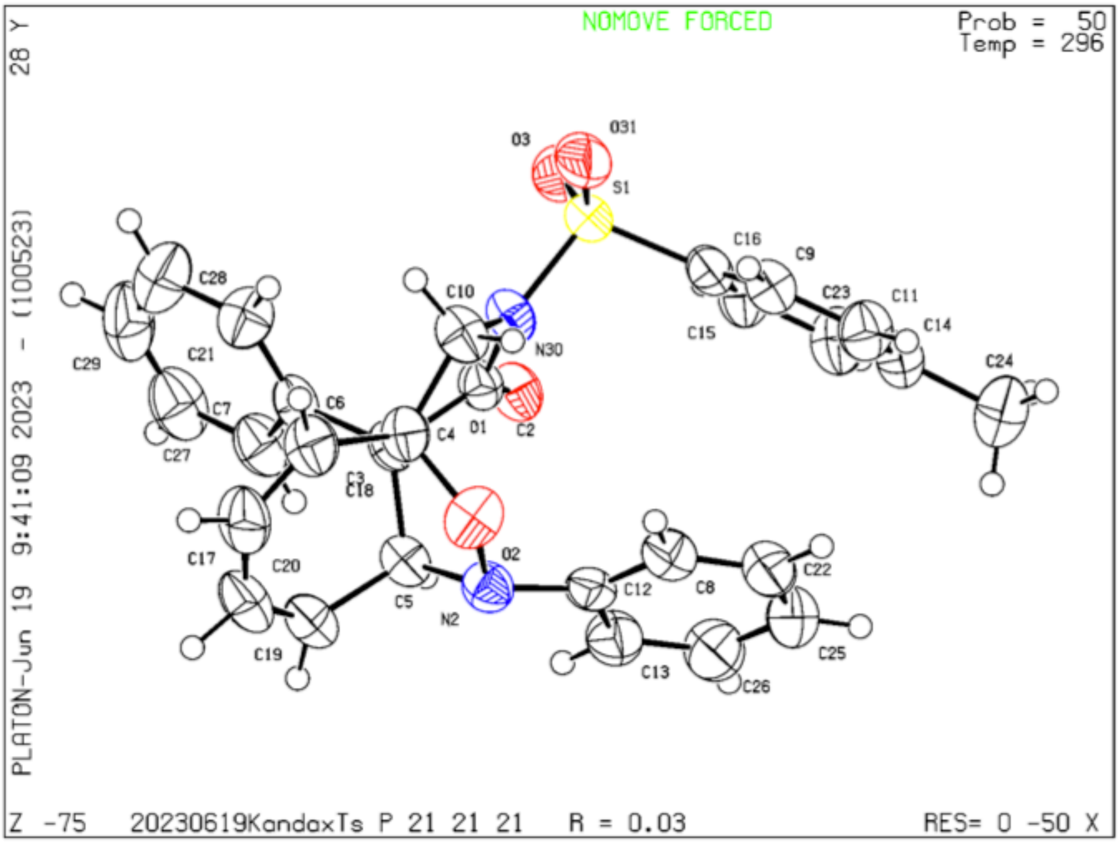

The ellipsoid contour probability level in the ORTEP is 50%.
CCDC No. 2286950

Supplementary Table 2. Crystal data and structure refinement for **4u'**.

A. Crystal Data

Empirical Formula	$C_{32}H_{23}Br_2Cl_2NO_3S$
Formula Weight	732.31
Crystal Color, Habit	colorless, block
Crystal Dimensions	0.200 X 0.200 X 0.200 mm
Crystal System	monoclinic
Lattice Type	Primitive
Lattice Parameters	a = 10.2076(3) Å<br>b = 20.4196(6) Å<br>c = 14.2492(4) Å<br>b = 97.603(7) ^{o}<br>V = 2943.91(16) $Å^3$
Space Group	$P2_1/n$ (#14)
Z value	4
D_{calc}	1.652 g/cm^3
F_{000}	1464.00
m(CuKa)	61.204 cm^{-1}

B. Intensity Measurements

Diffractometer	R-AXIS RAPID
Radiation	CuKa (l = 1.54187 Å)<br>multi-layer mirror monochromated
Voltage, Current	40kV, 30mA
Temperature	-180.0^{o}C
Detector Aperture	460.0 x 256.0 mm
Data Images	22 exposures
w oscillation Range (c=54.0, f=90.0)	80.0 - 260.0^{o}
Exposure Rate	2.0 sec./^{o}
w oscillation Range (c=54.0, f=180.0)	80.0 - 260.0^{o}
Exposure Rate	2.0 sec./^{o}
w oscillation Range (c=54.0, f=270.0)	80.0 - 260.0^{o}
Exposure Rate	2.0 sec./^{o}
w oscillation Range (c=0.0, f=0.0)	80.0 - 260.0^{o}
Exposure Rate	2.0 sec./^{o}
Detector Position	127.40 mm
Pixel Size	0.100 mm

$2q_{max}$	136.5^{o}
No. of Reflections Measured	Total: 15477<br>Unique: 5273 (R_{int} = 0.0737)
Corrections	Lorentz-polarization<br>Absorption<br>(trans. factors: 0.169 - 0.294)

C. Structure Solution and Refinement

Structure Solution	Direct Methods
Refinement	Full-matrix least-squares on F^2
Function Minimized	S w $(Fo^2 - Fc^2)^2$
Least Squares Weights	w = 1/ [$s^2(Fo^2)$ + $(0.1522 \cdot P)^2$ + 2.8473 · P]<br>where P = $(Max(Fo^2,0) + 2Fc^2)/3$
$2q_{max}$ cutoff	136.5^o
Anomalous Dispersion	All non-hydrogen atoms
No. Observations (All reflections)	5273
No. Variables	370
Reflection/Parameter Ratio	14.25
Residuals: R1 (I>2.00s(I))	0.0870
Residuals: R (All reflections)	0.1003
Residuals: wR2 (All reflections)	0.2676
Goodness of Fit Indicator	1.147
Max Shift/Error in Final Cycle	0.001
Maximum peak in Final Diff. Map	2.70 $e^-/Å^3$

Minimum peak in Final Diff. Map $\qquad$ -1.12 $e^-/Å^3$

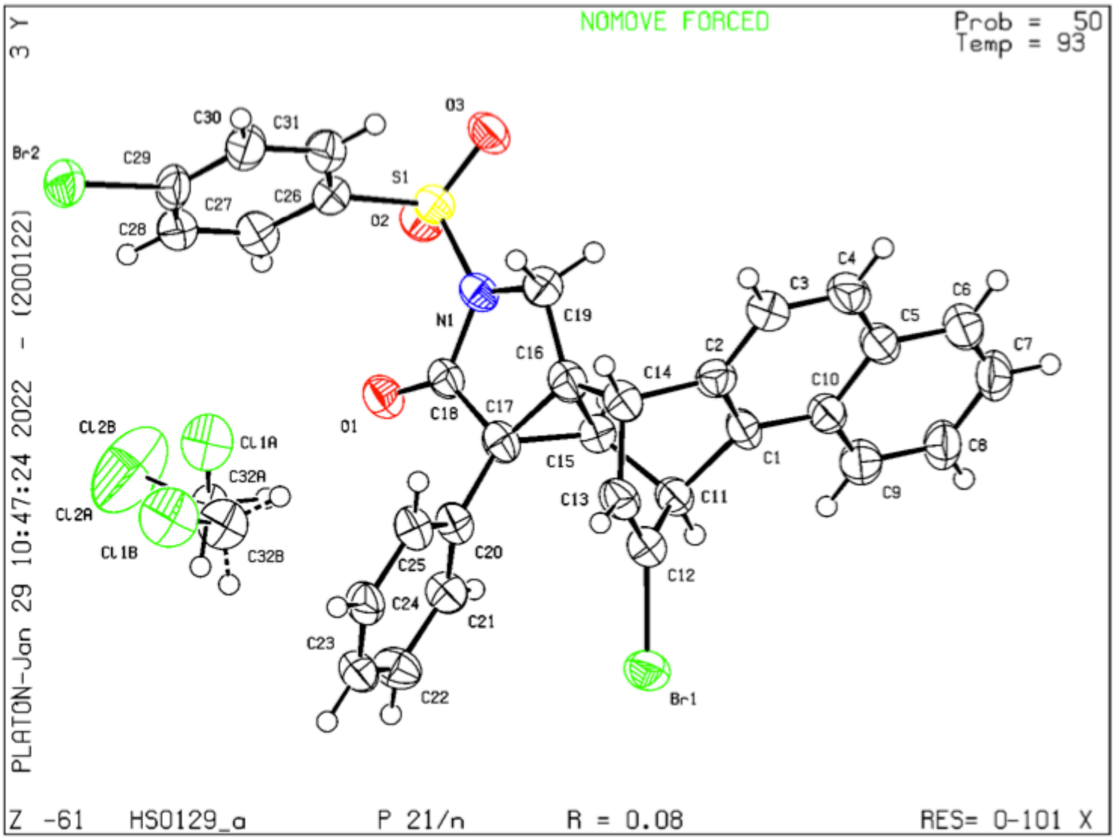

The ellipsoid contour probability level in the ORTEP is 50%.

CCDC No. 2286951

Supplementary Table 3. Crystal data and structure refinement for **11**.

A. Crystal Data

Empirical Formula	$C_{30}H_{34}N_2O_9S$
Formula Weight	598.67
Crystal Color, Habit	colorless, block
Crystal Dimensions	0.200 X 0.200 X 0.100 mm
Crystal System	monoclinic
Lattice Type	C-centered
Lattice Parameters	a = 14.1440(3) Å<br>b = 12.1070(3) Å<br>c = 32.1752(7) Å<br>b = 91.548(6) ^{o}<br>V = 5507.7(2) Å^3
Space Group	C2/c (#15)
Z value	8
D_{calc}	1.444 g/cm^3
F_{000}	2528.00
m(CuKa)	15.662 cm^{-1}

B. Intensity Measurements

Diffractometer	R-AXIS RAPID
Radiation	CuKa (l = 1.54187 Å)<br>multi-layer mirror monochromated
Voltage, Current	40kV, 30mA
Temperature	-180.0^{o}C
Detector Aperture	460.0 x 256.0 mm
Data Images	45 exposures
w oscillation Range (c=54.0, f=0.0)	80.0 - 260.0^{o}
Exposure Rate	5.0 sec./^{o}
w oscillation Range (c=54.0, f=90.0)	80.0 - 260.0^{o}
Exposure Rate	5.0 sec./^{o}
w oscillation Range (c=54.0, f=180.0)	80.0 - 260.0^{o}
Exposure Rate	5.0 sec./^{o}
w oscillation Range (c=54.0, f=270.0)	80.0 - 260.0^{o}
Exposure Rate	5.0 sec./^{o}
w oscillation Range (c=0.0, f=0.0)	80.0 - 260.0^{o}
Exposure Rate	5.0 sec./^{o}

Detector Position	127.40 mm
Pixel Size	0.100 mm
$2q_{max}$	136.4^{o}
No. of Reflections Measured	Total: 30611<br>Unique: 4975 (R_{int} = 0.0738)
Corrections	Lorentz-polarization<br>Absorption<br>(trans. factors: 0.569 - 0.855)

C. Structure Solution and Refinement

Structure Solution	Direct Methods
Refinement	Full-matrix least-squares on F^2
Function Minimized	$S\ w\ (Fo^2 - Fc^2)^2$
Least Squares Weights	$w = 1/\ [\ s^2(Fo^2) + (0.0583 \cdot P)^2$<br>$+ 8.8701 \cdot\ P\ ]$<br>where $P = (Max(Fo^2,0) + 2Fc^2)/3$
$2q_{max}$ cutoff	136.4^o
Anomalous Dispersion	All non-hydrogen atoms
No. Observations (All reflections)	4975
No. Variables	379
Reflection/Parameter Ratio	13.13
Residuals: R1 (I>2.00s(I))	0.0553
Residuals: R (All reflections)	0.0830
Residuals: wR2 (All reflections)	0.1380
Goodness of Fit Indicator	1.015
Max Shift/Error in Final Cycle	0.000
Maximum peak in Final Diff. Map	$0.52\ e^-/Å^3$

Minimum peak in Final Diff. Map -0.53 $e^{-}/Å^{3}$

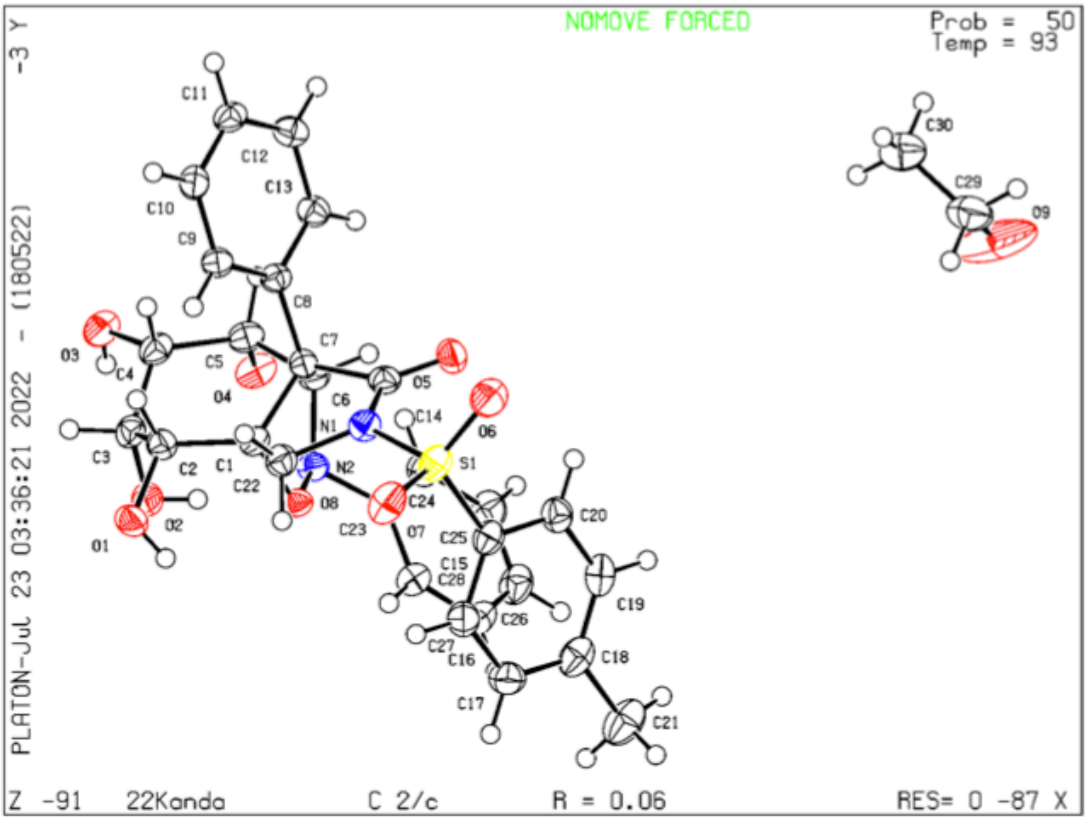

The ellipsoid contour probability level in the ORTEP is 50%.

CCDC No. 2286952

9. Synthesis and Characterization of Substrates

Ynamides[2] (**5a–e**, **5j–5m**, **5p–5u**), cycloheptatrienes[2] (**2a–2e**, **2j–2m**, **2p–2u**), nitroso compounds[3] and trimethylsilyl aryl triflates[4] were prepared according to the reported procedure. ^{1}H and ^{13}C NMR, IR, and MS of products were identical to those reported.

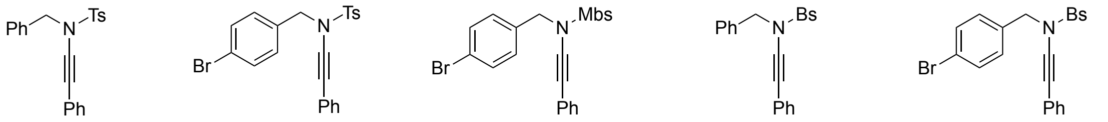

5a **5b** **5c** **5d** **5e**

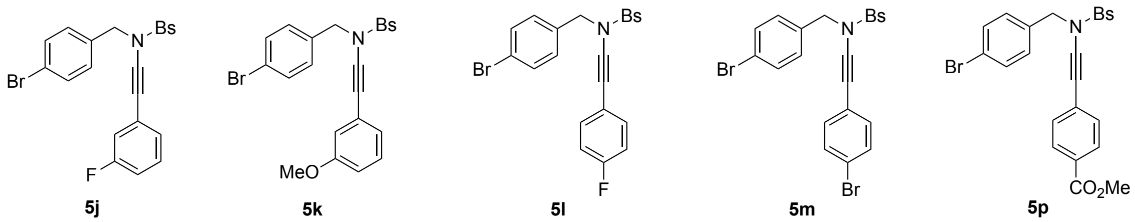

5j **5k** **5l** **5m** **5p**

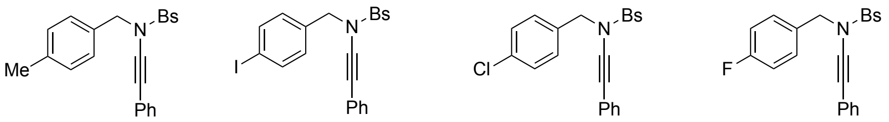

5q **5r** **5t** **5u**

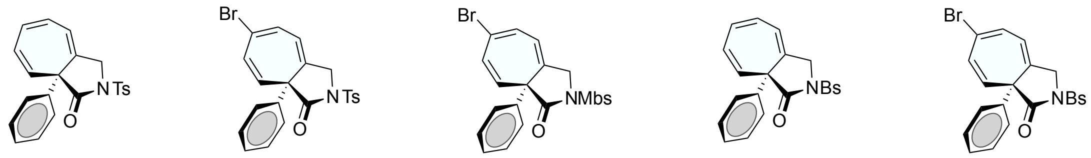

2a **2b** **2c** **2d** **2e**

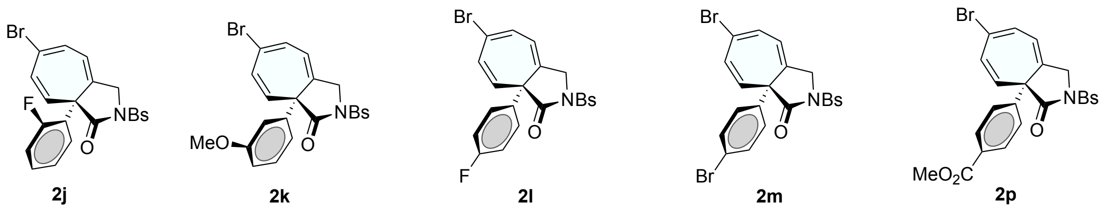

2j **2k** **2l** **2m** **2p**

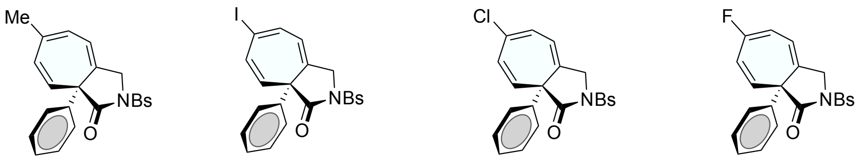

2q **2r** **2t** **2u**

General procedure for the synthesis of cycloheptatrienes

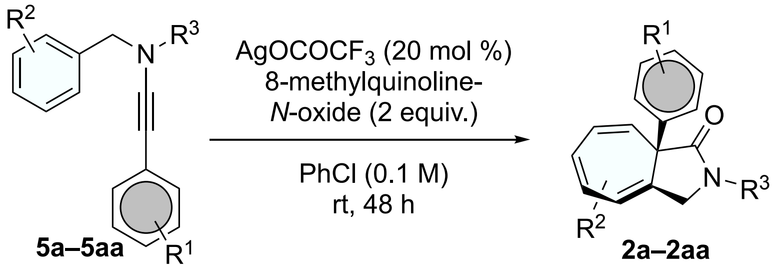

Ynamide **5** (1 equiv.), $AgOCOCF_3$ (20 mol %) and 8-methylquinoline *N*-oxide (2 equiv.) were suspended in PhCl (0.1 M) under an argon gas atmosphere. The whole was covered with aluminum foil to avoid light exposure. After the reaction mixture was stirred for 24 hours at room temperature, the resultant mixture was passed through Celite and concentrated under reduced pressure. Finally, the residue was purified by flash chromatography to give the cycloheptatriene **2**.

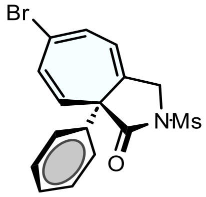

6-Bromo-2-(methylsulfonyl)-8a-phenyl-3,8a-dihydrocyclohepta[c]pyrrol-1(2*H*)-one (2i)

Prepared according to the general procedure for the synthesis of cycloheptatrienes using **5i** (36.4 mg, 0.1 mmol) and isolated as white solid (11.4 mg, 30% yield): TLC R_f = 0.1 (*n*-hexane/EtOAc, 3/1, v/v); m.p. 190°C (decomp.); ^{1}H NMR (600 MHz, $CDCl_3$) δ7.26–

7.25 (m, 3H), 7.21–7.20 (m, 2H), 6.72 (d, *J* = 6.9 Hz, 1H), 6.56 (d, *J* = 10.3 Hz, 1H), 6.34 (d, *J* = 6.9 Hz, 1H), 5.69 (d, *J* = 10.3 Hz, 1H), 4.77 (d, *J* = 15.2 Hz, 1H), 4.68 (d, *J* = 15.2 Hz, 1H), 3.28 (s, 3H); ^{13}C NMR (150 MHz, $CDCl_3$) δ174.2, 136.4, 131.1, 131.0, 128.4 (2C), 128.3, 127.7, 126.5 (2C), 124.3, 123.6, 121.4, 56.7, 49.7, 40.6; IR (ATR) ν 3023, 2362, 2344, 2330, 1732, 1352, 1230, 1171, 1144, 1056 cm^{-1}; HRMS (ESI-TOF) [M + Na]$^+$ calcd for $C_{16}H_{14}BrNNaO_3S^+$ m/z 401.9770, found 401.9764.

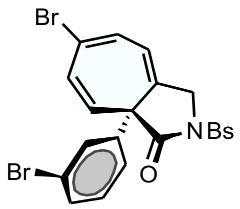

6-Bromo-8a-(3-bromophenyl)-2-((4-bromophenyl)sulfonyl)-3,8a-dihydrocyclohepta[c]pyrrol-1(2*H*)-one (2n)

Prepared according to the general procedure for the synthesis of cycloheptatrienes using **5n** (116.8 mg, 0.2 mmol) and isolated as white solid (56.8 mg, 47% yield): TLC R_f = 0.1 (*n*-hexane/EtOAc, 5/1, v/v); m.p. 197ºC (decomp.); ^{1}H NMR (400 MHz, $CDCl_3$) δ 7.88 (d, *J* = 7.3 Hz, 2H), 7.67 (d, *J* = 7.3 Hz, 2H), 7.33 (d, *J* = 7.8 Hz, 1H), 7.09–7.01 (m, 3H), 6.72 (d, *J* = 6.9 Hz, 1H), 6.52 (d, *J* = 10.1 Hz, 1H), 6.33 (d, *J* = 6.9 Hz, 1H), 5.50 (d, *J* = 10.1 Hz, 1H), 4.82 (d, *J* = 15.6 Hz, 1H), 4.68 (d, *J* = 15.6 Hz, 1H); ^{13}C NMR (100 MHz, $CDCl_3$) δ 172.5, 138.5, 136.0, 132.6 (2C), 131.4 (2C), 131.1, 130.2, 129.8, 129.6 (2C),

129.3, 126.9, 125.2, 124.5, 122.8, 122.4, 121.7, 56.3, 50.4; IR (ATR) ν 2922, 2364, 2345, 2334, 1741, 1573, 1469, 1390, 1369, 1336 cm^{-1}; HRMS (ESI-TOF) $[M + Na]^+$ calcd for $C_{21}H_{14}{}^{79}Br_2{}^{81}BrNNaO_3S^+$ m/z 621.8117, found 621.8129.

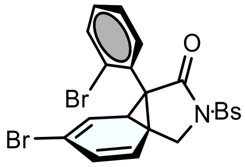

5-Bromo-3a-(2-bromophenyl)-2-((4-bromophenyl)sulfonyl)-1,2,3a,3b-tetrahydro-3*H*-benzo[1,3]cyclopropa[1,2-c]pyrrol-3-one (1o)

Prepared according to the general procedure for the synthesis of cycloheptatrienes using **5o** (58.4 mg, 0.1 mmol) and isolated as white solid (36.0 mg, 60% yield): TLC $R_f = 0.3$ (*n*-hexane/EtOAc, 3/1, v/v); m.p. >200ºC; ^1H NMR (600 MHz, $CDCl_3$) δ7.94 (d, *J* = 8.3 Hz, 2H), 7.69 (d, *J* = 8.3 Hz, 2H), 7.38 (d, *J* = 7.6 Hz, 1H), 7.20 (t, *J* = 7.6 Hz, 1H), 7.13 (d, *J* = 7.6 Hz, 1H), 7.09 (t, *J* = 7.6 Hz, 1H), 6.54 (d, *J* = 6.2 Hz, 1H), 6.04 (d, *J* = 9.6 Hz, 1H), 5.90 (d, *J* = 9.6 Hz, 1H), 4.15 (t, *J* = 12.4 Hz, 2H), 3.03 (d, *J* = 6.2 Hz, 1H); ^{13}C NMR (150 MHz, $CDCl_3$) δ172.4, 136.0, 135.3, 133.0, 132.4 (2C), 130.2, 129.9 (2C), 129.7, 128.9, 128.7, 126.9, 125.9, 124.3, 123.1, 119.4, 51.2, 40.7, 39.5, 31.1; IR (ATR) ν 3062, 2365, 2358, 1732, 1574, 1471, 1438, 1390, 1369, 1308 cm^{-1}; HRMS (ESI-TOF) $[M + H]^+$ calcd for $C_{21}H_{15}Br_3NO_3S^+$ m/z 579.8317, found 579.8320.

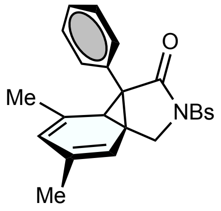

2-((4-Bromophenyl)sulfonyl)-4,6-dimethyl-3a-phenyl-1,2,3a,3b-tetrahydro-3*H*-benzo[1,3]cyclopropa[1,2-*c*]pyrrol-3-one (1v)

Prepared according to the general procedure for the synthesis of cycloheptatrienes using **5v** (227.0 mg, 0.5 mmol) and isolated as white solid (143.6 mg, 61% yield): TLC $R_f = 0.3$ (*n*-hexane/EtOAc, 5/1, v/v); m.p. 68–69 ºC; ^{1}H NMR (600 MHz, $CDCl_3$) δ 7.91 (d, J = 8.3 Hz, 2H), 7.65 (d, J = 8.3 Hz, 2H), 7.18–7.14 (m, 3H), 7.04–7.02 (m, 2H), 5.83 (s, 1H), 5.63 (s, 1H), 4.32 (s, 2H), 3.57 (s, 1H), 1.93 (s, 3H), 1.72 (s, 3H); ^{13}C NMR (150 MHz, $CDCl_3$) δ 174.2, 136.7, 135.7, 133.9, 132.4 (2C), 129.7 (2C), 129.5 (2C), 128.1, 127.8 (2C), 127.4(2C), 118.1(2C), 51.1, 31.5, 23.1, 22.6, 22.3, 14.1; IR (ATR) ν 3500, 2914, 2361, 2353, 2304, 1730, 1659, 1574, 1471, 1446 cm^{-1}; HRMS (ESI-TOF) $[M + Na]^+$ calcd for $C_{23}H_{20}BrNNaO_3S^+$ m/z 492.0239, found 492.0235.

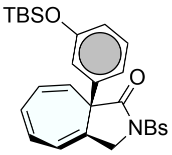

2-((4-Bromophenyl)sulfonyl)-8a-(3-((tert-butyldimethylsilyl)oxy)phenyl)-3,8a-dihydrocyclohepta[c]pyrrol-1(2*H*)-one (2w)

Prepared according to the general procedure for the synthesis of cycloheptatrienes using **5w** (278.3 mg, 0.5 mmol) and isolated as white solid (189.8 mg, 66% yield): TLC R_f = 0.2 (*n*-hexane/EtOAc, 15/1, v/v); m.p. 145 ºC (decomp.); ^{1}H NMR (600 MHz, $CDCl_3$) δ 7.88 (d, *J* = 8.3 Hz, 2H), 7.63 (d, *J* = 8.3 Hz, 2H), 6.98 (t, *J* = 7.6 Hz, 1H), 6.67 (d , *J* = 7.6 Hz, 1H), 6.63 (d, *J* = 8.3 Hz, 1H), 6.59 (s, 1H), 6.45 (d, *J* = 6.2 Hz, 1H), 6.33–6.28 (m, 2H), 6.24 (dd, *J* =10.3, 6.2 Hz, 1H), 5.54 (d, *J* = 9.6 Hz, 1H), 4.81 (d, *J* = 15.2 Hz, 1H), 4.69 (d, *J* = 15.2 Hz, 1H), 0.93 (s, 9H), 0.11 (s, 3H), 0.11 (s, 3H); ^{13}C NMR (150 MHz, $CDCl_3$) δ 173.7, 155.1, 138.1, 136.4, 132.4 (2C), 129.9, 129.6, 129.5 (2C), 128.8, 128.7, 127.1, 125.0, 122.0, 121.1, 119.6, 119.5, 118.8, 56.0, 50.4, 25.7 (3C), 18.2, –4.5 (2C); IR (ATR) ν 3091, 3025, 2929, 2885, 2857, 1741, 1596, 1575, 1482, 1470 cm^{-1}; HRMS (ESI-TOF) $[M + Na]^+$ calcd for $C_{27}H_{30}BrNNaO_4SSi^+$ m/z 594.0740, found 594.0746.

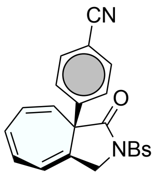

4-(2-((4-Bromophenyl)sulfonyl)-3-oxo-2,3-dihydrocyclohepta[c]pyrrol-3a(1*H*)-yl)benzonitrile (2x)

Prepared according to the general procedure for the synthesis of cycloheptatrienes using **5x** (270.8 mg, 0.6 mmol) and isolated as white solid (226.8 mg, 81% yield): TLC R_f = 0.2 (*n*-hexane/EtOAc, 3/1, v/v); m.p. 171 ºC (decomp.); ^{1}H NMR (600 MHz, $CDCl_3$) δ 7.89 (d, *J* = 8.3 Hz, 2H), 7.67 (d, *J* = 8.3 Hz, 2H), 7.44 (d, *J* = 8.3 Hz, 2H), 7.24 (d, *J* = 8.3 Hz, 2H), 6.54 (d, *J* = 6.9 Hz, 1H), 6.38–6.34 (m, 2H), 6.26 (dd, *J* = 11.0, 6.2 Hz, 1H), 5.51 (d, *J* = 9.6 Hz, 1H), 4.88 (d, *J* = 15.2 Hz, 1H), 4.71 (d, *J* = 15.2 Hz, 1H); ^{13}C NMR (150 MHz, $CDCl_3$) δ 172.7, 141.8, 136.1, 132.4 (2C), 131.5 (2C), 130.0, 129.9, 129.5 (2C), 128.9, 127.7 (2C), 127.6, 122.5, 122.2, 119.0, 118.3, 111.5, 55.6, 50.3; IR (ATR) ν 3092, 3025, 2886, 2254, 2229, 1738, 1650, 1604, 1574, 1498 cm^{-1}; HRMS (ESI-TOF) $[M + Na]^+$ calcd for $C_{22}H_{15}BrN_2NaO_3S^+$ m/z 488.9879, found 488.9897.

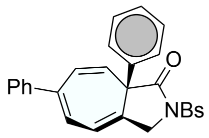

2-((4-Bromophenyl)sulfonyl)-6,8a-diphenyl-3,8a-dihydrocyclohepta[*c*]pyrrol-1(2*H*)-one (2y)

Prepared according to the general procedure for the synthesis of cycloheptatrienes using **5y** (301.4 mg, 0.6 mmol) and isolated as yellow solid (57.2 mg, 18% yield): TLC R_f = 0.4

(n-hexane/EtOAc, 5/1, v/v); m.p. 173–175 ºC; ^{1}H NMR (600 MHz, $CDCl_3$) δ 7.91 (d, J = 8.3 Hz, 2H), 7.66 (d, J = 8.3 Hz, 2H), 7.28–7.23 (m, 3H), 7.14–7.13 (m, 2H), 7.11 (s, 5H), 6.53–6.49 (m, 3H), 5.41 (d, J = 9.6 Hz, 1H), 4.85 (d, J = 14.5 Hz, 1H), 4.71 (d, J = 14.5 Hz, 1H); ^{13}C NMR (150 MHz, $CDCl_3$) δ 173.9, 142.2, 141.2, 136.5, 136.1, 132.4 (2C), 129.7 (2C), 129.6 (2C), 128.4 (2C), 127.9 (2C), 127.8, 127.7, 127.7, 127.2 (2C), 126.6 (3C), 122.0 (2C), 53.3, 50.7; IR (ATR) ν 3058, 3026, 2925, 2853, 1738, 1574, 1493, 1470, 1446, 1390 cm^{-1}; HRMS (ESI-TOF) [M + Na]$^+$ calcd for $C_{27}H_{20}BrNNaO_3S^+$ m/z 540.0239, found 540.0249.

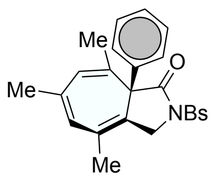

2-((4-Bromophenyl)sulfonyl)-4,6,8-trimethyl-8a-phenyl-3,8a-dihydrocyclohepta[*c*]pyrrol-1(2*H*)-one (2z)

Prepared according to the general procedure for the synthesis of cycloheptatrienes using **5z** (187.4 mg, 0.4 mmol) and isolated as white solid (100.2 mg, 53% yield): TLC R_f = 0.4 (n-hexane/EtOAc, 5/1, v/v); m.p. 119–121 ºC; ^{1}H NMR (600 MHz, $CDCl_3$) δ 7.92 (d, J = 8.3 Hz, 2H), 7.64 (d, J = 8.3 Hz, 2H), 7.09–7.03 (m, 3H), 6.91 (d, J = 6.9 Hz, 2H), 5.92 (s, 1H), 5.89 (s, 1H), 4.79 (d, J = 15.2 Hz, 1H), 4.64 (d, J = 15.2 Hz, 1H), 1.91 (s, 3H),

1.88 (s, 3H), 1.63 (s, 3H); ^{13}C NMR (150 MHz, $CDCl_3$) δ 173.2, 138.3, 138.0, 136.7, 132.3 (2C), 131.2, 129.7 (2C), 129.6, 129.2, 128.2, 127.3 (2C), 127.1, 126.7, 126.6 (2C), 119.6, 60.0, 50.1, 23.7, 22.4, 18.1; IR (ATR) ν 3087, 2977, 2915, 2356, 2262, 1737, 1630, 1574, 1493, 1471 cm^{-1}; HRMS (ESI-TOF) $[M + Na]^+$ calcd for $C_{24}H_{22}BrNNaO_3S^+$ m/z 506.0396, found 506.0391.

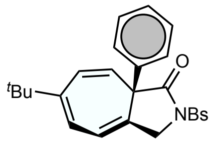

2-((4-Bromophenyl)sulfonyl)-6-(*tert*-butyl)-8a-phenyl-3,8a-dihydrocyclohepta[*c*]pyrrol-1(2*H*)-one (2aa)

Prepared according to the general procedure for the synthesis of cycloheptatrienes using **5aa** (289.5 mg, 0.6 mmol) and isolated as white solid (121.1 mg, 41% yield): TLC R_f = 0.2 (*n*-hexane/EtOAc, 5/1, v/v); m.p. 158–160 ºC; ^1H NMR (600 MHz, $CDCl_3$) δ 7.91 (d, *J* = 8.3 Hz, 2H), 7.65 (d, *J* = 8.3 Hz, 2H), 7.11–7.10 (m, 3H), 7.02–7.01 (m, 2H), 6.24 (d, *J* = 8.3 Hz, 1H), 6.17 (d, *J* = 8.3 Hz, 1H), 6.14 (d, *J* = 8.3 Hz, 1H), 4.57 (d, *J* = 13.1 Hz, 1H), 4.46 (d, *J* = 13.1 Hz, 1H), 4.43 (d, *J* = 8.3 Hz, 1H), 0.80 (s, 9H); ^{13}C NMR (150 MHz, $CDCl_3$) δ 174.1, 149.9, 136.7, 133.6, 132.4 (2C), 129.7 (2C), 129.5, 129.3 (2C), 127.6 (2C), 127.5 (2C), 123.9 (2C), 122.1, 121.8 (2C), 50.9, 35.0, 29.2 (3C); IR (ATR) ν

3088, 3030, 2962, 2905, 2870, 2362, 1733, 1680, 1574, 1493 cm^{-1}; HRMS (ESI-TOF) $[M + Na]^+$ calcd for $C_{25}H_{24}BrNNaO_3S^+$ m/z 520.0552, found 520.0555.

General procedure for the synthesis of ynamides

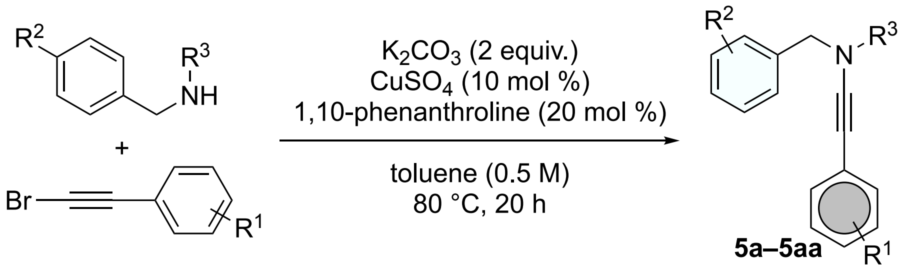

N-Alkyl substituted amide (1 equiv.), $CuSO_4$ (10 mol %), 1,10-phenanthroline (20 mol %), and K_2CO_3 (2 equiv.) were suspended in toluene (0.5 M) under an argon gas atmosphere. After alkynyl bromide (1.2 equiv.) was introduced to the reaction mixture, the whole was stirred at 80 °C for 20 hours. Then, the reaction mixture was filtered through a short pad of Celite and concentrated under reduced pressure. Finally, the residue was purified by flash chromatography to give ynamide **5**.

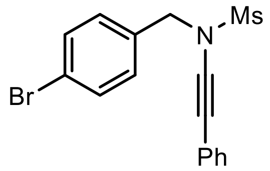

***N*-(4-Bromobenzyl)-*N*-(phenylethynyl)methanesulfonamide (5i)**

Prepared according to the general procedure for the synthesis of ynamides using *N*-(4-bromobenzyl) methanesulfonamide (246.1 mg, 1 mmol) and isolated as white solid (311.2 mg, 85% yield): TLC R_f = 0.1 (*n*-hexane/EtOAc, 5/1, v/v); m.p. 68–70 ºC; ^{1}H NMR (400 MHz, $CDCl_3$) δ7.54 (d, *J* = 8.2 Hz, 2H), 7.37–7.34 (m, 4H), 7.30–7.29 (m, 3H), 4.66 (s, 2H), 3.00 (s, 3H); ^{13}C NMR (100 MHz, $CDCl_3$) δ133.6, 132.0 (2C), 131.5 (2C), 130.6 (2C), 128.3 (2C), 128.2, 122.9, 122.2, 81.5, 71.9, 55.1, 39.0; IR (ATR) ν 3670, 3019, 2365, 2358, 2340, 2331, 2237, 1594, 1489, 1442 cm^{-1}; HRMS (ESI-TOF) $[M + Na]^+$ calcd for $C_{16}H_{14}BrNNaO_2S^+$ m/z 385.9821, found 385.9825.

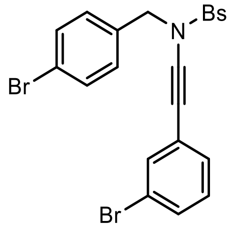

4-Bromo-*N*-(4-bromobenzyl)-*N*-((3-bromophenyl)ethynyl)benzenesulfonamide (5n)

Prepared according to the general procedure for the synthesis of ynamides using 4-bromo-*N*-(4-bromobenzyl)benzenesulfonamide (1499 mg, 3.7 mmol) and isolated as white solid(1120 mg, 52% yield): TLC R_f = 0.4 (*n*-hexane/EtOAc, 5/1, v/v); m.p, 66–68 ºC; ^{1}H NMR (400 MHz, $CDCl_3$) δ7.72 (d, *J* = 8.0 Hz, 2H), 7.68 (d, *J* = 8.0 Hz, 2H), 7.49 (d, *J* = 8.8 Hz, 2H), 7.40 (d, *J* = 8.8 Hz, 2H), 7.20–7.11 (m, 4H), 4.54 (s, 2H); ^{13}C NMR (100 MHz, $CDCl_3$) δ136.4, 133.8, 133.0, 132.6 (2C), 131.8 (2C), 131.2, 130.4 (2C), 129.7,

129.7, 129.2, 129.0 (2C), 124.2, 122.8, 122.1, 83.0, 70.5, 55.2; IR (ATR) ν 3087, 3063, 2934, 2234, 1591, 1573, 1553, 1488, 1469, 1437 cm^{-1}; HRMS (ESI-TOF) $[M + Na]^+$ calcd for $C_{21}H_{14}Br_3NNaO_2S^+$ m/z 603.8188, found 603.8184.

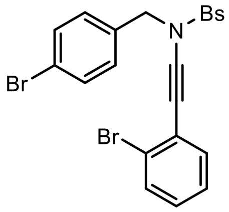

4-Bromo-*N*-(4-bromobenzyl)-*N*-((2-bromophenyl)ethynyl)benzenesulfonamide (5o)

Prepared according to the general procedure for the synthesis of ynamides using 4-bromo-*N*-(4-bromobenzyl)benzenesulfonamide (445.6 mg, 1.1 mmol) and isolated as white solid (245.5 mg, 38% yield): TLC R_f = 0.3 (*n*-hexane/EtOAc, 5/1, v/v); m.p. 89–91 ºC; ^1H NMR (400 MHz, $CDCl_3$) δ7.77 (d, *J* = 8.7 Hz, 2H), 7.65 (d, *J* = 8.7 Hz, 2H), 7.53 (d, *J* = 7.8 Hz, 1H), 7.45 (d, *J* = 8.2 Hz, 2H), 7.18–7.20 (m, 4H), 7.14–7.10 (m, 1H), 4.60 (s, 2H); ^{13}C NMR (100 MHz, $CDCl_3$) δ136.5, 133.0, 132.7, 132.5 (2C), 132.3, 131.8 (2C), 130.6 (2C), 129.1 (2C), 129.0, 127.0 (2C), 124.7, 124.6, 122.8, 86.2, 70.8, 55.4; IR (ATR) ν 3087, 2358, 2338, 2236, 1592, 1574, 1488, 1470, 1433, 1407 cm^{-1}; HRMS (ESI-TOF) $[M + Na]^+$ calcd for $C_{21}H_{14}Br_3NNaO_2S^+$ m/z 603.8188, found 603.8181.

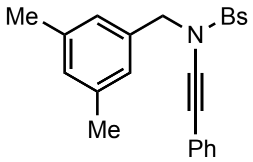

4-Bromo-*N*-(3,5-dimethylbenzyl)-*N*-(phenylethynyl)benzenesulfonamide (5v)

Prepared according to the general procedure for the synthesis of ynamides using 4-bromo-*N*-(3,5-dimethylbenzyl)benzenesulfonamide (566.8 mg, 1.6 mmol) and isolated as white solid (491 mg, 68% yield): TLC R_f = 0.6 (*n*-hexane/EtOAc, 5/1, v/v); m.p. 68–69 ºC; ^{1}H NMR (600 MHz, $CDCl_3$) δ 7.71 (d, *J* = 8.3 Hz, 2H), 7.62 (d, *J* = 8.3 Hz, 2H), 7.27 (s, 5H), 6.93 (s, 1H), 6.88 (s, 2H), 4.54 (s, 2H), 2.27 (s, 6H); ^{13}C NMR (150 MHz, $CDCl_3$) δ 138.1 (2C), 136.7, 133.8, 132.2 (2C), 131.2 (2C), 130.0, 129.1 (2C), 128.7, 128.2 (2C), 127.9, 126.6 (2C), 122.5, 82.4, 71.4, 56.0, 21.2 (2C); IR (ATR) ν 3020, 2919, 2356, 2334, 2236, 1607, 1574, 1469, 1442, 1390 cm^{-1}; HRMS (ESI-TOF) $[M + Na]^+$ calcd for $C_{23}H_{20}BrNNaO_2S^+$ m/z 476.0290, found 476.0284.

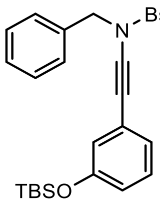

***N*-Benzyl-4-bromo-*N*-((3-((tert-butyldimethylsilyl)oxy)phenyl)ethynyl)benzenesulfonamide (5w)**

Prepared according to the general procedure for the synthesis of ynamides using *N*-benzyl-4-bromobenzenesulfonamide (391.5 mg, 1.2 mmol) and isolated as yellow oil (504.8 mg, 75% yield): TLC R_f = 0.2 (*n*-hexane/EtOAc, 30/1, v/v); ^{1}H NMR (600 MHz, $CDCl_3$) δ 7.71 (d, J = 9.0 Hz, 2H), 7.63 (d, J = 9.0 Hz, 2H), 7.32 (s, 5H), 7.12 (t, J = 7.6 Hz, 1H), 6.85 (d, J = 7.6 Hz, 1H), 6.76–6.74 (m, 1H), 6.70 (s, 1H), 4.61 (s, 2H), 0.98 (s, 9H), 0.18 (s, 6H); ^{13}C NMR (150 MHz, $CDCl_3$) δ 155.4, 136.5, 134.0, 132.3 (2C), 129.3, 129.1 (2C), 128.9 (2C), 128.8, 128.6 (2C), 128.5, 124.3, 123.4, 122.7, 120.2, 81.9, 71.5, 55.9, 25.6 (3C), 18.2, –4.5 (2C); IR (ATR) ν 3089, 3065, 3033, 2954, 2929, 2885, 2857, 2237, 1595, 1573 cm^{-1}; HRMS (ESI-TOF) $[M + Na]^+$ calcd for $C_{27}H_{30}BrNNaO_3SSi^+$ m/z 578.0791, found 578.0788.

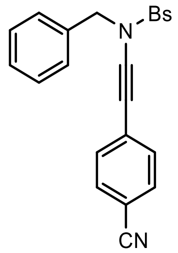

N-Benzyl-4-bromo-N-((4-cyanophenyl)ethynyl)benzenesulfonamide (5x)

Prepared according to the general procedure for the synthesis of ynamides using *N*-benzyl-4-bromobenzenesulfonamide (1141.7 mg, 3.5 mmol) and isolated as white solid (525.6 mg, 33% yield): TLC R_f = 0.2 (*n*-hexane/EtOAc, 10/1, v/v); m.p. 112–114 ºC; ^{1}H

NMR (600 MHz, $CDCl_3$) δ 7.72 (d, *J* = 9.0 Hz, 2H), 7.66 (d, *J* = 9.0 Hz, 2H), 7.53 (d, *J* = 9.0 Hz, 2H), 7.34–7.33 (m, 3H), 7.31–7.29 (m, 2H), 7.27–7.26 (m, 2H), 4.63 (s, 2H); ^{13}C NMR (150 MHz, $CDCl_3$) δ 136.4, 133.6, 132.6 (2C), 132.0 (2C), 130.9 (2C), 129.2, 129.0 (2C), 128.9 (2C), 128.8, 128.7 (2C), 127.7, 118.5, 110.7, 86.7, 71.2, 55.8; IR (ATR) ν 3089, 3065, 3033, 2223, 1604, 1574, 1497, 1471, 1456, 1407 cm^{-1}; HRMS (ESI-TOF) $[M + Na]^+$ calcd for $C_{22}H_{15}BrN_2NaO_2S^+$ m/z 472.9930, found 472.9935.

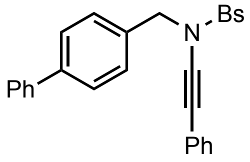

***N*-([1,1'-biphenyl]-4-ylmethyl)-4-bromo-*N*-(phenylethynyl)benzenesulfonamide (5y)**

Prepared according to the general procedure for the synthesis of ynamides using *N*-([1,1'-biphenyl]-4-ylmethyl)-4-bromobenzenesulfonamide (402.3 mg, 1.0 mmol) and isolated as white solid (229.2 mg, 44% yield): TLC R_f = 0.5 (*n*-hexane/EtOAc, 5/1, v/v); m.p. 118–120 ºC; ^1H NMR (600 MHz, $CDCl_3$) δ 7.73 (d, *J* = 8.3 Hz, 2H), 7.63 (d, *J* = 9.0 Hz, 2H), 7.57 (d, *J* = 8.3 Hz, 2H), 7.53 (d, *J* = 8.3 Hz, 2H), 7.45 (t, *J* = 7.6 Hz, 2H), 7.39 (d, *J* = 9.0 Hz, 2H), 7.36 (t, *J* = 7.6 Hz, 1H), 7.29–7.25 (m, 5H), 4.66 (s, 2H); ^{13}C NMR (150 MHz, $CDCl_3$) δ 141.5, 140.5, 136.7, 133.1, 132.3 (2C), 131.3 (2C), 129.3 (2C), 129.1

(2C), 129.3 (2C), 128.8 (2C), 128.8, 128.3 (2C), 128.0 (2C), 127.5, 127.3 (2C), 127.1 (2C), 122.4, 82.3, 71.6, 55.8; IR (ATR) ν 3057, 3031, 2359, 2334, 2235, 1910, 1746, 1599, 1573, 1520 cm^{-1}; HRMS (ESI-TOF) $[M + Na]^+$ calcd for $C_{27}H_{20}BrNNaO_2S^+$ m/z 524.0290, found 524.0294.

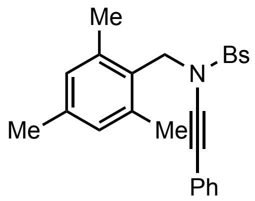

4-Bromo-*N*-(phenylethynyl)-*N*-(2,4,6-trimethylbenzyl)benzenesulfonamide (5z)

Prepared according to the general procedure for the synthesis of ynamides using 4-bromo-*N*-(2,4,6-trimethylbenzyl)benzenesulfonamide (589.3 mg, 1.6 mmol) and isolated as white solid (363.3 mg, 48% yield): TLC $R_f = 0.6$ (*n*-hexane/EtOAc, 5/1, v/v); m.p. 126–128 ºC; ^1H NMR (600 MHz, $CDCl_3$) δ 7.86 (d, $J = 9.0$ Hz, 2H), 7.73 (d, $J = 9.0$ Hz, 2H), 7.02–7.19 (m, 3H), 7.05–7.03 (m, 2H), 6.85 (s, 2H), 4.56 (s, 2H), 2.27 (s, 6H), 2.26 (s, 3H); ^{13}C NMR (150 MHz, $CDCl_3$) δ 138.8 (2C), 138.6, 135.6, 132.5 (2C), 130.6 (2C), 129.3 (2C), 129.2 (2C), 129.0, 128.1 (2C), 127.4, 126.2, 122.7, 81.5, 71.2, 48.9, 21.0, 19.8 (2C); IR (ATR) ν 2953, 2917, 2363, 2333, 2238, 1917, 1745, 1613, 1598, 1573 cm^{-1}; HRMS (ESI-TOF) $[M + Na]^+$ calcd for $C_{24}H_{22}BrNNaO_2S^+$ m/z 490.0447, found 490.0450.

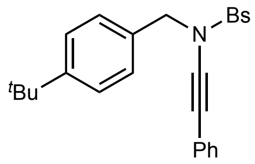

4-Bromo-*N*-(4-(*tert*-butyl)benzyl)-*N*-(phenylethynyl)benzenesulfonamide (5aa)

Prepared according to the general procedure for the synthesis of ynamides using 4-bromo-*N*-(4-(*tert*-butyl)benzyl)benzenesulfonamide (764.6 mg, 2.0 mmol) and isolated as pale yellow oil (795.0 mg, 82.4% yield): TLC R_f = 0.4 (*n*-hexane/EtOAc, 5/1, v/v); ^{1}H NMR (600 MHz, $CDCl_3$) δ 7.67 (d, *J* = 8.3 Hz, 2H), 7.57 (d, *J* = 9.0 Hz, 2H), 7.30 (d, *J* = 8.3 Hz, 2H), 7.26 (s, 5H), 7.24 (d, *J* = 9.0 Hz, 2H), 4.60 (s, 2H), 1.30 (s, 9H); ^{13}C NMR (150 MHz, $CDCl_3$) δ 151.6, 136.7, 132.2 (2C), 131.2 (2C), 131.0, 129.0 (2C), 128.6 (2C), 128.5, 128.2 (2C), 127.9, 125.4 (2C), 122.5, 82.6, 71.3, 55.8, 34.5, 31.3 (3C); IR (ATR) ν 3057, 2961, 2904, 2867, 2365, 2334, 2234, 1912, 1698, 1598 cm^{-1}; HRMS (ESI-TOF) $[M + Na]^+$ calcd for $C_{25}H_{24}BrNNaO_2S^+$ m/z 504.0603, found 504.0610.

General procedure for the synthesis of sulfonamides

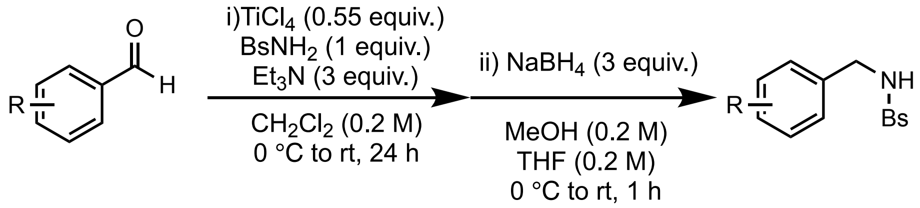

Benzaldehyde (1 eq), *p*-bromobenzenesulfonylamide (1 equiv.), and triethylamine (3 equiv.) were dissolved in anhydrous CH_2Cl_2 (0.2 M). After cooling the mixture to 0 °C, titanium tetrachloride (0.55 equiv.) was added dropwise. After 30 min, the reaction mixture was allowed to room temperature and stirred for 24 h. Then, the reaction mixture was filtered through a short pad of Celite and concentrated under reduced pressure. The residue was dissolved in THF (0.2 M) and MeOH (0.2 M). After cooling the mixture to 0 °C, $NaBH_4$ (2 equiv.) was added slowly. Then the reaction mixture was allowed to room temperature and stirred for 1 h. The reaction was quenched with water, and the solvent was removed under reduced pressure. Then, the aqueous layer was extracted with EtOAc three times. The combined organic layers were dried over Na_2SO_4 and concentrated under reduced pressure. The residue was recrystallized from DCM/Hex to give corresponding sulfonamides as a white solid.

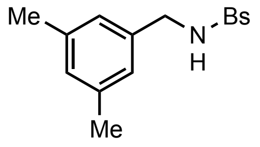

4-Bromo-*N*-(3,5-dimethylbenzyl)benzenesulfonamide

Prepared according to the general procedure for the synthesis of sulfonamides using 3,5-dimethylbenzaldehyde (402.5 mg, 3 mmol) and isolated as yellow solid (619 mg, 58% yield): TLC R_f = 0.6 (*n*-hexane/EtOAc, 2/1, v/v); m.p. 105–107 ºC ; ^{1}H NMR (600 MHz, $CDCl_3$) δ 7.68 (d, *J* = 8.3 Hz, 2H), 7.60 (d, *J* = 8.3 Hz, 2H), 6.87 (s, 1H), 6.73 (s, 2H), 4.87 (t, *J* = 5.5 Hz, 1H), 4.06 (d, *J* = 5.5 Hz, 2H), 2.22 (s, 6H); ^{13}C NMR (150 MHz, $CDCl_3$) δ 139.1, 138.3 (2C), 135.6, 132.2 (2C), 129.5, 128.6 (2C), 127.5, 125.6 (2C), 47.2, 21.1 (2C); IR (ATR) ν 3280, 3019, 2969, 2950, 2916, 2372, 2357, 2335, 1748, 1607 cm^{-1}; HRMS (ESI-TOF) [M + Na]$^+$ calcd for $C_{15}H_{16}BrNNaO_2S^+$ m/z 375.9977, found 375.9997.

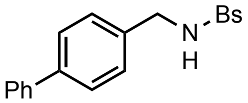

***N*-([1,1'-Biphenyl]-4-ylmethyl)-4-bromobenzenesulfonamide**

Prepared according to the general procedure for the synthesis of sulfonamides using [1,1'-biphenyl]-4-carbaldehyde (455 mg, 2.5 mmol) and isolated as white solid (544.4 mg, 54% yield): TLC R_f = 0.5 (*n*-hexane/EtOAc, 2/1, v/v); m.p. 145–146 ºC; ^{1}H NMR (600 MHz, $CDCl_3$) δ 7.71 (d, *J* = 8.3 Hz, 2H), 7.62 (d, *J* = 8.3 Hz, 2H), 7.54 (d, *J* = 7.6 Hz, 2H), 7.49 (d, *J* = 8.3 Hz, 2H), 7.43 (d, *J* = 7.6 Hz, 2H), 7.35 (d, *J* = 7.6 Hz, 1H), 7.24 (d, *J* = 8.3 Hz, 2H), 4.94 (t, *J* = 6.2 Hz, 1H), 4.19 (d, *J* = 6.2 Hz, 2H); ^{13}C NMR (150 MHz, $CDCl_3$) δ 141.1, 140.4, 139.1, 134.8, 132.4 (2C), 128.8 (2C), 128.7 (2C), 128.4, 128.3, 127.7, 127.5

(2C), 127.4, 127.4, 127.0, 47.0; IR (ATR) ν 3246, 2368, 1575, 1487, 1470, 1456, 1436, 1390, 1294, 1275 cm^{-1}; HRMS (ESI-TOF) $[M + Na]^+$ calcd for $C_{19}H_{16}BrNNaO_2S^+$ m/z 423.9977, found 423.9978.

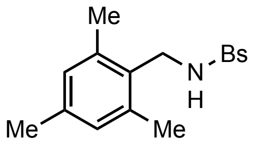

4-Bromo-*N*-(2,4,6-trimethylbenzyl)benzenesulfonamide

Prepared according to the general procedure for the synthesis of sulfonamides using 2,4,6-trimethylbenzaldehyde (435.9 mg, 3 mmol) and isolated as pale yellow solid (610.3 mg, 55% yield): TLC R_f = 0.6 (*n*-hexane/EtOAc, 2/1, v/v); m.p. 104–106 ºC; ^{1}H NMR (600 MHz, $CDCl_3$) δ 7.71 (d, *J* = 8.3 Hz, 2H), 7.63 (d, *J* = 8.3 Hz, 2H), 6.78 (s, 2H), 4.51 (t, *J* = 5.5 Hz, 1H), 4.07 (d, *J* = 5.5 Hz, 2H), 2.22 (s, 3H), 2.15 (s, 6H); ^{13}C NMR (150 MHz, $CDCl_3$) δ 138.4, 138.1, 137.2 (2C), 132.3 (2C), 129.2 (2C), 128.6 (2C), 128.5, 127.6, 41.2, 20.9, 19.2 (2C); IR (ATR) ν 3274, 3087, 2952, 2917, 2358, 2304, 1745, 1612, 1575, 1472 cm^{-1}; HRMS (ESI-TOF) $[M + Na]^+$ calcd for $C_{16}H_{18}NNaO_2S^+$ m/z 390.0134, found 390.0131.

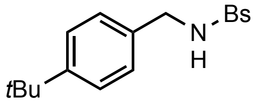

4-Bromo-*N*-(4-(*tert*-butyl)benzyl)benzenesulfonamide

Prepared according to the general procedure for the synthesis of sulfonamides using 4-(*tert*-butyl)benzaldehyde (836 mg, 5 mmol) and isolated as white solid (1508 mg, 79% yield): TLC R_f = 0.6 (*n*-hexane/EtOAc, 2/1, v/v); m.p. 133–135 ºC; ^{1}H NMR (600 MHz, $CDCl_3$) δ 7.67 (d, *J* = 9.0 Hz, 2H), 7.59 (d, *J* = 9.0 Hz, 2H), 7.27 (d, *J* = 8.3 Hz, 2H), 7.08 (d, *J* = 8.3 Hz, 2H), 4.74 (d, *J* = 6.2 Hz, 1H), 4.12 (d, *J* = 6.2 Hz, 2H), 1.28 (s, 9H); ^{13}C NMR (150 MHz, $CDCl_3$) δ 151.2, 139.2, 132.7, 132.3 (2C), 128.7 (2C), 127.7 (2C), 127.5, 125.7 (2C), 47.0, 34.5, 31.3 (3C); IR (ATR) ν 3265, 2961, 2360, 1574, 1470, 1434, 1389, 1321, 1276, 1154 cm^{-1}; HRMS (ESI-TOF) [M + Na]$^+$ calcd for $C_{17}H_{20}BrNNaO_2S^+$ m/z 404.0290, found 404.0290.

The procedure for the synthesis of cycloadduct with 4-phenyl-1,2,4-triazoline-3,5-dione

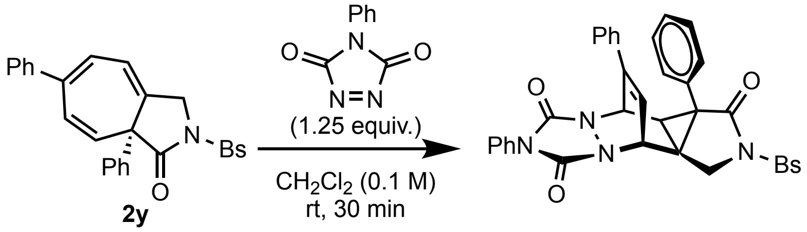

2-((4-bromophenyl)sulfonyl)-3a,7,12-triphenyl-1,2,3b,4-tetrahydro-6*H*,10*H*-4,10-ethenopyrrolo[3',4':1,3]cyclopropa[1,2-*d*][1,2,4]triazolo[1,2-*a*]pyridazine-3,6,8(3a*H*,7*H*)-trione ((±)-s1)

4-Phenyl-1,2,4-triazoline-3,5-dione (10.9 mg, 0.0625 mmol, 1.25 equiv.) was added to a solution of **2y** (25.9 mg, 0.05 mmol, 1 equiv.) in CH_2Cl_2 (0.1 M, 0.5 mL) at the room

temperature. The reaction mixture was stirred for 30 min at the room temperature. Then, the solvent was removed under reduced pressure and remaining pale yellow solid was washed with cold CH_2Cl_2 to afford (±)-**s1** as a white solid (26.7 mg, 77% yield).: TLC R_f = 0.3 (*n*-hexane/EtOAc, 2/1, v/v); m.p. 250 ºC (decomp.); ^{1}H NMR (400 MHz, $(CD_3)_2SO$) δ 7.94 (d, *J* = 8.7 Hz, 2H), 7.90 (d, *J* = 8.7 Hz, 2H), 7.45–7.36 (m, 3H), 7.26–7.16 (m, 4H), 7.10 (t, *J* = 7.8 Hz, 3H), 7.00 (t, *J* = 7.3 Hz, 1H), 6.82 (d, *J* = 7.8 Hz, 3H), 6.67 (t, *J* = 7.3 Hz, 1H), 6.16 (dd, *J* = 6.4, 1.8 Hz, 1H), 6.03–6.02 (m, 1H), 5.72 (d, *J* = 6.4 Hz, 1H), 4.69 (d, *J* = 9.2 Hz, 1H), 4.33 (d, *J* = 9.2 Hz, 1H), 2.05 (d, *J* = 4.6 Hz, 1H); ^{13}C NMR (100 MHz, $(CD_3)_2SO$) δ 170.0, 156.5, 155.9, 137.7, 136.5, 133.4 , 132.7 (2C), 131.1, 130.7 (2C), 130.6, 129.9 (2C), 129.2 (2C), 129.0, 128.6, 128.6, 128.1, 128.1, 128.0 (2C), 127.2, 125.9 (2C), 124.8 (2C), 120.8, 54.9, 54.0, 48.7, 44.6, 29.1, 24.1; IR (ATR) ν 3059, 1771, 1711, 1598, 1573, 1500, 1475, 1447, 1401, 1358 cm^{-1}; HRMS (ESI-TOF) $[M + Na]^+$ calcd for $C_{35}H_{25}BrN_4NaO_5S^+$ m/z 715.0621, found 715.0641.

General procedure for the synthesis of nitroso compounds

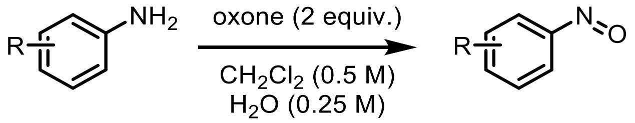

A solution of oxone (2 equiv.) in water (0.25 M) was added to a solution of aniline derivative (1 equiv.) in DCM (0.5 M) with vigorous stirring. The reaction mixture was

stirred at room temperature under an argon atmosphere for 30 min to 24 h. After that, the reaction was quenched by the addition of a saturated solution of $NaHCO_3$. The mixture was extracted with DCM. The combined organic layers were washed with 1 N HCl, brine, dried over Na_2SO_4, and concentrated under reduced pressure. The residue was purified by flash chromatography to afford nitroso compounds.

General procedure for the synthesis of trimethylsilyl aryl triflates

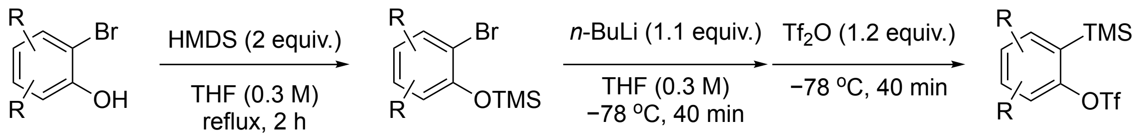

To a solution of *o*-bromophenol (1 equiv.) in anhydrous THF (0.3 M) was added HMDS (2 equiv.) under an argon atmosphere. The reaction mixture was refluxed for 2 h. After cooling to room temperature, the crude product was attained by removing the solvent in *vacuo*. The crude product was dissolved in anhydrous THF (0.3 M) under an argon atmosphere and cooled to −78 °C, then *n*-BuLi (1.1 equiv., 2 M in cyclohexane) was added dropwise to the mixture. Then, the reaction mixture was stirred for 40 min at −78 °C. After Tf_2O (1.2 equiv.) was added to the mixture dropwise at −78 °C, the reaction was stirred for another 40 min. The reaction was quenched with a cold saturated solution of $NaHCO_3$ at −78 °C and warmed to room temperature. Then, the aqueous layer was extracted with Et_2O three times. The combined organic layers were dried over Na_2SO_4

and concentrated under reduced pressure. Finally, the residue was purified by flash chromatography to afford aryne precursors.

10. Computational Details (Mechanistic Investigation)

Supplementary Table 4. Electronic energy data.

	E(RωB97X-D/6-31G*) (A.U.)	E(RωB97X-D/6-311+G**) (A.U.)
**CHT'**	-1527.926253	-1528.238238
**TS0**	-1527.913879	-1528.225949
**NCD'**	-1527.927645	-1528.238953
**PhNO**	-361.412982	-361.508769
**Aryne**	-230.819316	-230.878689
**INT1**$_{NO\_4+2}$	-1889.353689	-1889.75848
**TS1**$_{NO\_4+2}$	-1889.327704	-1889.729902
**PD**$_{NO\_4+2}$	-1889.364429	-1889.764606
**INT1**$_{NO\_6+2}$	-1889.363636	-1889.766627
**TS1**$_{NO\_6+2}$	-1889.328772	-1889.72914
**INT2**$_{NO\_6+2}$	-1889.344972	-1889.745151
**TS2**$_{NO\_4+2}$	-1889.343614	-1889.745142
**PD**$_{NO\_6+2}$	-1889.390648	-1889.791622
**INT1**$_{BZ\_4+2}$	-1758.761546	-1759.130351
**TS1**$_{BZ\_4+2}$	-1758.759113	-1759.126108

**PD**$_{BZ\_4+2}$	-1758.907237	-1759.268237
**INT1**$_{BZ\_6+2}$	-1758.760507	-1759.128952
**TS1**$_{BZ\_6+2}$	-1758.740717	-1759.108625
**INT2**$_{BZ\_6+2}$	-1758.761011	-1759.129713
**TS2**$_{BZ\_6+2}$	-1758.753957	-1759.123094
**INT3**$_{BZ\_6+2}$	-1758.892689	-1759.253416
**TS3**$_{BZ\_6+2}$	-1758.815905	-1759.176869
**PD**$_{BZ\_6+2}$	-1758.929284	-1759.291651
**INT1**$_{4+2(4N1O)}$	-1889.355841	-1889.761201
**TS1**$_{4+2(4N1O)}$	-1889.321342	-1889.723172
**PD**$_{4+2(4N1O)}$	-1889.378164	-1889.778081
**INT1**$_{4+2(1N4O)}$	-1889.359348	-1889.763289
**TS1**$_{4+2(1N4O)}$	-1889.316267	-1889.71798
**INT2**$_{4+2(1N4O)}$	-1889.325758	-1889.728326
**TS2**$_{4+2(1N4O)}$	-1889.323879	-1889.725808
**PD**$_{4+2(1N4O)}$	-1889.369365	-1889.767513
**INT1**$_{4+2(3N6O)}$	-1889.361906	-1889.765343
**TS1**$_{4+2(3N6O)}$	-1889.316268	-1889.717392

**PD**$_{4+2(3N6O)}$	-1889.374804	-1889.77463
**TS2**$_{4+2(6N3O)}$	-1889.336956	-1889.737563
**PD**$_{4+2(6N3O)}$	-1889.380664	-1889.778324

11. Computational Details (Training Data)

CHT/NCD		
HOBO	NAME	SMILES
-6.1873	001_CHT	O=C1N(S(C)(=O)=O)CC2=CC=CC=CC21C3=CC=CC=C3
-6.0572	002_CHT	O=C1N(S(C)(=O)=O)CC2=CC=C(C)C=CC21C3=CC=CC=C3
-6.6300	003_CHT	O=C1N(S(C)(=O)=O)CC2=CC=C(C(F)(F)F)C=CC21C3=CC=CC=C3
-6.1968	004_CHT	O=C1N(S(C)(=O)=O)CC2=CC=C(F)C=CC21C3=CC=CC=C3
-6.3043	005_CHT	O=C1N(S(C)(=O)=O)CC2=CC=C(Br)C=CC21C3=CC=CC=C3
-5.9280	006_CHT	O=C1N(S(C)(=O)=O)CC2=CC=C(C3=CC=CC=C3)C=CC21C4=CC=CC=C4
-6.3446	007_CHT	O=C1N(S(C)(=O)=O)CC2=CC=C(Cl)C=CC21C3=CC=CC=C3
-6.8624	008_CHT	O=C1N(S(C)(=O)=O)CC2=CC=C([N+]([O-])=O)C=CC21C3=CC=CC=C3
-5.7952	009_CHT	O=C1N(S(C)(=O)=O)CC2=CC=C(O)C=CC21C3=CC=CC=C3
-6.4145	010_CHT	O=C1N(S(C)(=O)=O)CC2=CC=C(C(OC)=O)C=CC21C3=CC=CC=C3
-5.9250	011_CHT	O=C1N(S(C)(=O)=O)CC2=CC=C(C=C)C=CC21C3=CC=CC=C3
-6.4967	012_CHT	O=C1N(S(C)(=O)=O)CC2=CC=C(C(C)=O)C=CC21C3=CC=CC=C3
-6.6896	013_CHT	O=C1N(S(C)(=O)=O)CC2=CC=C(S(C)(=O)=O)C=CC21C3=CC=CC=C3
-5.8983	014_CHT	O=C1N(S(C)(=O)=O)CC2=CC=C(S)C=CC21C3=CC=CC=C3
-6.0656	015_CHT	O=C1N(S(C)(=O)=O)CC2=CC=C(SC)C=CC21C3=CC=CC=C3
-6.1565	016_CHT	O=C1N(S(C)(=O)=O)CC2=CC=C(S(C)=O)C=CC21C3=CC=CC=C3
-6.7201	017_CHT	O=C1N(S(C)(=O)=O)CC2=CC=C(C#N)C=CC21C3=CC=CC=C3
-6.0695	018_CHT	O=C1N(S(C)(=O)=O)CC2=CC=C(C(C)(C)C)C=CC21C3=CC=CC=C3
-6.0975	019_NCD	O=C(C1(C2=CC=CC=C2)C3(C4)C=CC=CC31)N4S(C)(=O)=O
-5.9922	020_NCD	O=C(C1(C2=CC=CC=C2)C3(C4)C=CC(C)=CC31)N4S(C)(=O)=O
-6.5331	021_NCD	O=C(C1(C2=CC=CC=C2)C3(C4)C=CC(C(F)(F)F)=CC31)N4S(C)(=O)=O
-6.2126	022_NCD	O=C(C1(C2=CC=CC=C2)C3(C4)C=CC(F)=CC31)N4S(C)(=O)=O
-6.2801	023_NCD	O=C(C1(C2=CC=CC=C2)C3(C4)C=CC(Br)=CC31)N4S(C)(=O)=O
-5.9037	024_NCD	O=C(C1(C2=CC=CC=C2)C3(C4)C=CC(C5=CC=CC=C5)=CC31)N4S(C)(=O)=O
-6.3122	025_NCD	O=C(C1(C2=CC=CC=C2)C3(C4)C=CC(Cl)=CC31)N4S(C)(=O)=O
-6.7769	026_NCD	O=C(C1(C2=CC=CC=C2)C3(C4)C=CC([N+]([O-])=O)=CC31)N4S(C)(=O)=O
-5.7636	027_NCD	O=C(C1(C2=CC=CC=C2)C3(C4)C=CC(O)=CC31)N4S(C)(=O)=O
-6.3228	028_NCD	O=C(C1(C2=CC=CC=C2)C3(C4)C=CC(C(OC)=O)=CC31)N4S(C)(=O)=O
-5.9299	029_NCD	O=C(C1(C2=CC=CC=C2)C3(C4)C=CC(C=C)=CC31)N4S(C)(=O)=O
-6.3908	030_NCD	O=C(C1(C2=CC=CC=C2)C3(C4)C=CC(C(C)=O)=CC31)N4S(C)(=O)=O
-6.6314	031_NCD	O=C(C1(C2=CC=CC=C2)C3(C4)C=CC(S(C)(=O)=O)=CC31)N4S(C)(=O)=O
-6.0665	032_NCD	O=C(C1(C2=CC=CC=C2)C3(C4)C=CC(S)=CC31)N4S(C)(=O)=O
-5.9873	033_NCD	O=C(C1(C2=CC=CC=C2)C3(C4)C=CC(SC)=CC31)N4S(C)(=O)=O
-6.2477	034_NCD	O=C(C1(C2=CC=CC=C2)C3(C4)C=CC(S(C)=O)=CC31)N4S(C)(=O)=O
-6.6583	035_NCD	O=C(C1(C2=CC=CC=C2)C3(C4)C=CC(C#N)=CC31)N4S(C)(=O)=O
-5.9658	036_NCD	O=C(C1(C2=CC=CC=C2)C3(C4)C=CC(C(C)(C)C)=CC31)N4S(C)(=O)=O
-6.1179	037_CHT	O=C1N(S(C)(=O)=O)CC2=CC=CC=CC21C
-6.3759	038_CHT	O=C1N(S(C)(=O)=O)CC2=CC=CC=CC21C(F)(F)F
-6.3851	039_CHT	O=C1N(S(C)(=O)=O)CC2=CC=CC=CC21O
-6.3642	040_CHT	O=C1N(S(C)(=O)=O)CC2=CC=CC=CC21OC
-5.9549	041_CHT	O=C1N(S(C)(=O)=O)CC2=CC=CC=CC21C(C)(C)C
-6.3987	042_CHT	O=C1N(S(C)(=O)=O)CC2=CC=CC=CC21Br
-6.6050	043_CHT	O=C1N(S(C)(=O)=O)CC2=CC=CC=CC21F
-6.2262	044_CHT	O=C1N(S(C)(=O)=O)CC2=CC=CC=CC21C3=CC=CC4=CC=CC=C43
-6.6219	045_CHT	O=C1N(S(C)(=O)=O)CC2=CC=CC=CC21N3C4=CC=CC=C4C=C3
-6.1220	046_CHT	O=C1N(S(C)(=O)=O)CC2=CC=CC=CC21C3=CNC4=CC=CC=C43
-6.2812	047_CHT	O=C1N(S(C)(=O)=O)CC2=CC=CC=CC21C3=CC4=CC=CC=C4N3
-6.0760	048_CHT	O=C1N(S(C)(=O)=O)CC2=CC=CC=CC21C3=CC=CN3
-6.1263	049_CHT	O=C1N(S(C)(=O)=O)CC2=CC=CC=CC21C3=CC=CC=N3
-6.3250	050_CHT	O=C1N(S(C)(=O)=O)CC2=CC=CC=CC21C3=CN=CC=C3

Supplementary Fig. 23 \| List of CHT and NCD's HOBO level (**001_CHT–050_CHT**).

CHT/NCD		
HOBO	NAME	SMILES
-6.3966	051_CHT	O=C1N(S(C)(=O)=O)CC2=CC=CC=CC21C3=CC=NC=C3
-5.6205	052_CHT	O=C1N(S(C)(=O)=O)CC2=CC=CC=CC21[Si](C)(C)C
-6.1718	053_CHT	O=C1N(S(C)(=O)=O)CC2=CC=CC=CC21SC
-6.2058	054_CHT	O=C1N(S(C)(=O)=O)CC2=CC=CC=CC21C(C)=O
-6.7062	055_CHT	O=C1N(S(C)(=O)=O)CC2=CC=CC=CC21C#N
-6.1410	056_NCD	O=C(C1(C)C2(C3)C=CC=CC21)N3S(C)(=O)=O
-6.4765	057_NCD	O=C(C1(C(F)(F)F)C2(C3)C=CC=CC21)N3S(C)(=O)=O
-6.0216	058_NCD	O=C(C1(O)C2(C3)C=CC=CC21)N3S(C)(=O)=O
-6.0488	059_NCD	O=C(C1(OC)C2(C3)C=CC=CC21)N3S(C)(=O)=O
-6.0635	060_NCD	O=C(C1(C(C)(C)C)C2(C3)C=CC=CC21)N3S(C)(=O)=O
-6.3710	061_NCD	O=C(C1(Br)C2(C3)C=CC=CC21)N3S(C)(=O)=O
-6.2771	062_NCD	O=C(C1(F)C2(C3)C=CC=CC21)N3S(C)(=O)=O
-6.1190	063_NCD	O=C(C1(C2=CC=CC3=C2C=CC=C3)C4(C5)C=CC=CC41)N5S(C)(=O)=O
-6.4007	064_NCD	O=C(C1(N2C=CC3=C2C=CC=C3)C4(C5)C=CC=CC41)N5S(C)(=O)=O
-6.1087	065_NCD	O=C(C1(C2=CNC3=C2C=CC=C3)C4(C5)C=CC=CC41)N5S(C)(=O)=O
-6.4872	066_NCD	O=C(C1(C2=CC3=C(C=CC=C3)N2)C4(C5)C=CC=CC41)N5S(C)(=O)=O
-6.3392	067_NCD	O=C(C1(C2=CC=CN2)C3(C4)C=CC=CC31)N4S(C)(=O)=O
-6.0540	068_NCD	O=C(C1(C2=NC=CC=C2)C3(C4)C=CC=CC31)N4S(C)(=O)=O
-6.2306	069_NCD	O=C(C1(C2=CN=CC=C2)C3(C4)C=CC=CC31)N4S(C)(=O)=O
-6.3041	070_NCD	O=C(C1(C2=CC=NC=C2)C3(C4)C=CC=CC31)N4S(C)(=O)=O
-6.1141	071_NCD	O=C(C1([Si](C)(C)C)C2(C3)C=CC=CC21)N3S(C)(=O)=O
-5.9429	072_NCD	O=C(C1(SC)C2(C3)C=CC=CC21)N3S(C)(=O)=O
-6.2798	073_NCD	O=C(C1(C(C)=O)C2(C3)C=CC=CC21)N3S(C)(=O)=O
-6.6297	074_NCD	O=C(C1(C#N)C2(C3)C=CC=CC21)N3S(C)(=O)=O
-5.9252	075_NCD	O=C(C1(C2=CC=CC=C2)C3(C4)C=CC=C(C)C31)N4S(C)(=O)=O
-6.5383	076_NCD	O=C(C1(C2=CC=CC=C2)C3(C4)C=CC=C(C(F)(F)F)C31)N4S(C)(=O)=O
-6.0744	077_NCD	O=C(C1(C2=CC=CC=C2)C3(C4)C=CC=C(F)C31)N4S(C)(=O)=O
-6.1677	078_NCD	O=C(C1(C2=CC=CC=C2)C3(C4)C=CC=C(Br)C31)N4S(C)(=O)=O
-5.7260	079_NCD	O=C(C1(C2=CC=CC=C2)C3(C4)C=CC=C(C5=CC=CC=C5)C31)N4S(C)(=O)=O
-6.2025	080_NCD	O=C(C1(C2=CC=CC=C2)C3(C4)C=CC=C(Cl)C31)N4S(C)(=O)=O
-6.8126	081_NCD	O=C(C1(C2=CC=CC=C2)C3(C4)C=CC=C([N+]([O-])=O)C31)N4S(C)(=O)=O
-5.6523	082_NCD	O=C(C1(C2=CC=CC=C2)C3(C4)C=CC=C(O)C31)N4S(C)(=O)=O
-6.3560	083_NCD	O=C(C1(C2=CC=CC=C2)C3(C4)C=CC=C(C(OC)=O)C31)N4S(C)(=O)=O
-5.8109	084_NCD	O=C(C1(C2=CC=CC=C2)C3(C4)C=CC=C(C=C)C31)N4S(C)(=O)=O
-6.4115	085_NCD	O=C(C1(C2=CC=CC=C2)C3(C4)C=CC=C(C(C)=O)C31)N4S(C)(=O)=O
-6.6161	086_NCD	O=C(C1(C2=CC=CC=C2)C3(C4)C=CC=C(S(C)(=O)=O)C31)N4S(C)(=O)=O
-5.7854	087_NCD	O=C(C1(C2=CC=CC=C2)C3(C4)C=CC=C(S)C31)N4S(C)(=O)=O
-5.5979	088_NCD	O=C(C1(C2=CC=CC=C2)C3(C4)C=CC=C(SC)C31)N4S(C)(=O)=O
-6.1465	089_NCD	O=C(C1(C2=CC=CC=C2)C3(C4)C=CC=C(S(C)=O)C31)N4S(C)(=O)=O
-6.6072	090_NCD	O=C(C1(C2=CC=CC=C2)C3(C4)C=CC=C(C#N)C31)N4S(C)(=O)=O
-5.8825	091_NCD	O=C(C1(C2=CC=CC=C2)C3(C4)C=CC=C(C(C)(C)C)C31)N4S(C)(=O)=O
-5.9971	092_NCD	O=C(C1(C2=CC=CC=C2)C3(C4)C=C(C)C=CC31)N4S(C)(=O)=O
-6.5337	093_NCD	O=C(C1(C2=CC=CC=C2)C3(C4)C=C(C(F)(F)F)C=CC31)N4S(C)(=O)=O
-6.2126	094_NCD	O=C(C1(C2=CC=CC=C2)C3(C4)C=C(F)C=CC31)N4S(C)(=O)=O
-6.2820	095_NCD	O=C(C1(C2=CC=CC=C2)C3(C4)C=C(Br)C=CC31)N4S(C)(=O)=O
-5.9173	096_NCD	O=C(C1(C2=CC=CC=C2)C3(C4)C=C(C5=CC=CC=C5)C=CC31)N4S(C)(=O)=O
-6.3127	097_NCD	O=C(C1(C2=CC=CC=C2)C3(C4)C=C(Cl)C=CC31)N4S(C)(=O)=O
-6.7737	098_NCD	O=C(C1(C2=CC=CC=C2)C3(C4)C=C([N+]([O-])=O)C=CC31)N4S(C)(=O)=O
-5.7767	099_NCD	O=C(C1(C2=CC=CC=C2)C3(C4)C=C(O)C=CC31)N4S(C)(=O)=O

Supplementary Fig. 24 | List of CHT and NCD's HOBO level (**051_CHT–099_CHT**).

		CHT/NCD
HOBO	NAME	SMILES
-6.3182	100_NCD	O=C(C1(C2=CC=CC=C2)C3(C4)C=C(C(OC)=O)C=CC31)N4S(C)(=O)=O
-5.9339	101_NCD	O=C(C1(C2=CC=CC=C2)C3(C4)C=C(C=C)C=CC31)N4S(C)(=O)=O
-6.3827	102_NCD	O=C(C1(C2=CC=CC=C2)C3(C4)C=C(C(C)=O)C=CC31)N4S(C)(=O)=O
-6.6452	103_NCD	O=C(C1(C2=CC=CC=C2)C3(C4)C=C(S(C)(=O)=O)C=CC31)N4S(C)(=O)=O
-6.0599	104_NCD	O=C(C1(C2=CC=CC=C2)C3(C4)C=C(S)C=CC31)N4S(C)(=O)=O
-5.9848	105_NCD	O=C(C1(C2=CC=CC=C2)C3(C4)C=C(SC)C=CC31)N4S(C)(=O)=O
-6.2711	106_NCD	O=C(C1(C2=CC=CC=C2)C3(C4)C=C(S(C)=O)C=CC31)N4S(C)(=O)=O
-6.6537	107_NCD	O=C(C1(C2=CC=CC=C2)C3(C4)C=C(C#N)C=CC31)N4S(C)(=O)=O
-5.9723	108_NCD	O=C(C1(C2=CC=CC=C2)C3(C4)C=C(C(C)(C)C)C=CC31)N4S(C)(=O)=O
-5.9149	109_NCD	O=C(C1(C2=CC=CC=C2)C3(C4)C(C)=CC=CC31)N4S(C)(=O)=O
-6.5492	110_NCD	O=C(C1(C2=CC=CC=C2)C3(C4)C(C(F)(F)F)=CC=CC31)N4S(C)(=O)=O
-6.0820	111_NCD	O=C(C1(C2=CC=CC=C2)C3(C4)C(F)=CC=CC31)N4S(C)(=O)=O
-6.1881	112_NCD	O=C(C1(C2=CC=CC=C2)C3(C4)C(Br)=CC=CC31)N4S(C)(=O)=O
-5.8790	113_NCD	O=C(C1(C2=CC=CC=C2)C3(C4)C(C5=CC=CC=C5)=CC=CC31)N4S(C)(=O)=O
-6.2033	114_NCD	O=C(C1(C2=CC=CC=C2)C3(C4)C(Cl)=CC=CC31)N4S(C)(=O)=O
-6.7957	115_NCD	O=C(C1(C2=CC=CC=C2)C3(C4)C([N+]([O-])=O)=CC=CC31)N4S(C)(=O)=O
-5.6640	116_NCD	O=C(C1(C2=CC=CC=C2)C3(C4)C(O)=CC=CC31)N4S(C)(=O)=O
-6.4455	117_NCD	O=C(C1(C2=CC=CC=C2)C3(C4)C(C(OC)=O)=CC=CC31)N4S(C)(=O)=O
-5.8545	118_NCD	O=C(C1(C2=CC=CC=C2)C3(C4)C(C=C)=CC=CC31)N4S(C)(=O)=O
-6.4327	119_NCD	O=C(C1(C2=CC=CC=C2)C3(C4)C(C(C)=O)=CC=CC31)N4S(C)(=O)=O
-6.6322	120_NCD	O=C(C1(C2=CC=CC=C2)C3(C4)C(S(C)(=O)=O)=CC=CC31)N4S(C)(=O)=O
-5.7775	121_NCD	O=C(C1(C2=CC=CC=C2)C3(C4)C(S)=CC=CC31)N4S(C)(=O)=O
-5.6079	122_NCD	O=C(C1(C2=CC=CC=C2)C3(C4)C(SC)=CC=CC31)N4S(C)(=O)=O
-6.3242	123_NCD	O=C(C1(C2=CC=CC=C2)C3(C4)C(S(C)=O)=CC=CC31)N4S(C)(=O)=O
-6.6169	124_NCD	O=C(C1(C2=CC=CC=C2)C3(C4)C(C#N)=CC=CC31)N4S(C)(=O)=O
-5.8890	125_NCD	O=C(C1(C2=CC=CC=C2)C3(C4)C(C(C)(C)C)=CC=CC31)N4S(C)(=O)=O
-5.8401	126_NCD	O=C(C1(C2=CC=CC=C2)C3(C4)C=C(C)C=C(C)C31)N4S(C)(=O)=O
-6.8844	127_NCD	O=C(C1(C2=CC=CC=C2)C3(C4)C=C(C(F)(F)F)C=C(C(F)(F)F)C31)N4S(C)(=O)=O
-6.2142	128_NCD	O=C1C2(C3(C=C(C=C(C32)F)F)CN1S(C)(=O)=O)C4=CC=CC=C4
-6.3399	129_NCD	O=C(C1(C2=CC=CC=C2)C3(C4)C=C(Br)C=C(Br)C31)N4S(C)(=O)=O
-5.6542	130_NCD	O=C1C2(C3(C=C(C=C(C32)C4=CC=CC=C4)C5=CC=CC=C5)CN1S(C)(=O)=O)C6=CC=CC=C6
-6.4074	131_NCD	O=C(C1(C2=CC=CC=C2)C3(C4)C=C(Cl)C=C(Cl)C31)N4S(C)(=O)=O
-7.2425	132_NCD	O=C(C1(C2=CC=CC=C2)C3(C4)C=C([N+]([O-])=O)C=C([N+]([O-])=O)C31)N4S(C)(=O)=O
-5.4792	133_NCD	O=C(C1(C2=CC=CC=C2)C3(C4)C=C(O)C=C(O)C31)N4S(C)(=O)=O
-6.5307	134_NCD	O=C(C1(C2=CC=CC=C2)C3(C4)C=C(C(OC)=O)C=C(C(OC)=O)C31)N4S(C)(=O)=O
-5.7598	135_NCD	O=C1C2(C3(C=C(C=C(C32)C=C)C=C)CN1S(C)(=O)=O)C4=CC=CC=C4
-6.6450	136_NCD	O=C(C1(C2=CC=CC=C2)C3(C4)C=C(C(C)=O)C=C(C(C)=O)C31)N4S(C)(=O)=O
-6.8254	137_NCD	O=C1C2(C3(C=C(C=C(C32)S(C)(=O)=O)S(C)(=O)=O)CN1S(C)(=O)=O)C4=CC=CC=C4
-5.7924	138_NCD	O=C(C1(C2=CC=CC=C2)C3(C4)C=C(S)C=C(S)C31)N4S(C)(=O)=O
-5.5410	139_NCD	O=C(C1(C2=CC=CC=C2)C3(C4)C=C(SC)C=C(SC)C31)N4S(C)(=O)=O
-6.3103	140_NCD	O=C(C1(C2=CC=CC=C2)C3(C4)C=C(S(C)=O)C=C(S(C)=O)C31)N4S(C)(=O)=O
-7.0602	141_NCD	O=C(C1(C2=CC=CC=C2)C3(C4)C=C(C#N)C=C(C#N)C31)N4S(C)(=O)=O
-5.7851	142_NCD	O=C(C1(C2=CC=CC=C2)C3(C4)C=C(C(C)(C)C)C=C(C(C)(C)C)C31)N4S(C)(=O)=O
-5.8665	143_NCD	O=C(C1(C2=CC=CC=C2)C3(C4)C(C)=CC=CC31C)N4S(C)(=O)=O
-6.7897	144_NCD	O=C(C1(C2=CC=CC=C2)C3(C4)C(C(F)(F)F)=CC=CC31C(F)(F)F)N4S(C)(=O)=O
-6.4243	145_NCD	O=C1C2(C3(C(F)=CC=CC32F)CN1S(C)(=O)=O)C4=CC=CC=C4
-6.4482	146_NCD	O=C(C1(C2=CC=CC=C2)C3(C4)C(Br)=CC=CC31Br)N4S(C)(=O)=O
-5.8425	147_NCD	O=C1C2(C3(C(C4=CC=CC=C4)=CC=CC32C5=CC=CC=C5)CN1S(C)(=O)=O)C6=CC=CC=C6
-6.5089	148_NCD	O=C(C1(C2=CC=CC=C2)C3(C4)C(Cl)=CC=CC31Cl)N4S(C)(=O)=O
-7.1590	149_NCD	O=C1C2(C3(C([N+]([O-])=O)=CC=CC32[N+]([O-])=O)CN1S(C)(=O)=O)C4=CC=CC=C4
-5.8482	150_NCD	O=C(C1(C2=CC=CC=C2)C3(C4)C(O)=CC=CC31O)N4S(C)(=O)=O

Supplementary Fig. 25 | List of CHT and NCD's HOBO level (**100_CHT–150_CHT**).

		CHT/NCD
HOBO	NAME	SMILES
-6.4126	151_NCD	O=C(C1(C2=CC=CC=C2)C3(C4)C(C(OC)=O)=CC=CC31C(OC)=O)N4S(C)(=O)=O
-5.7960	152_NCD	O=C(C1(C2=CC=CC=C2)C3(C4)C(C=C)=CC=CC31C=C)N4S(C)(=O)=O
-6.4256	153_NCD	O=C(C1(C2=CC=CC=C2)C3(C4)C(C(C)=O)=CC=CC31C(C)=O)N4S(C)(=O)=O
-6.7922	154_NCD	O=C(C1(C2=CC=CC=C2)C3(C4)C(S(C)(=O)=O)=CC=CC31S(C)(=O)=O)N4S(C)(=O)=O
-5.9035	155_NCD	O=C1C2(C3(C(S)=CC=CC32S)CN1S(C)(=O)=O)C4=CC=CC=C4
-5.6194	156_NCD	O=C1C2(C3(C(SC)=CC=CC32SC)CN1S(C)(=O)=O)C4=CC=CC=C4
-6.2436	157_NCD	O=C1C2(C3(C(S(C)=O)=CC=CC32S(C)=O)CN1S(C)(=O)=O)C4=CC=CC=C4
-7.0540	158_NCD	O=C1C2(C3(C(C#N)=CC=CC32C#N)CN1S(C)(=O)=O)C4=CC=CC=C4
-5.7824	159_NCD	O=C1C2(C3(C(C(C)(C)C)=CC=CC32C(C)(C)C)CN1S(C)(=O)=O)C4=CC=CC=C4
-6.1470	160_CHT	O=C1N(S(C)(=O)=O)CC2=CC=CC(C)=CC21C3=CC=CC=C3
-6.5718	161_CHT	O=C1N(S(C)(=O)=O)CC2=CC=CC(C(F)(F)F)=CC21C3=CC=CC=C3
-6.3386	162_CHT	O=C1N(S(C)(=O)=O)CC2=CC=CC(F)=CC21C3=CC=CC=C3
-6.4512	163_CHT	O=C1N(S(C)(=O)=O)CC2=CC=CC(Br)=CC21C3=CC=CC=C3
-6.1250	164_CHT	O=C1N(S(C)(=O)=O)CC2=CC=CC(C3=CC=CC=C3)=CC21C4=CC=CC=C4
-6.4705	165_CHT	O=C1N(S(C)(=O)=O)CC2=CC=CC(Cl)=CC21C3=CC=CC=C3
-6.7476	166_CHT	O=C1N(S(C)(=O)=O)CC2=CC=CC([N+]([O-])=O)=CC21C3=CC=CC=C3
-6.0466	167_CHT	O=C1N(S(C)(=O)=O)CC2=CC=CC(O)=CC21C3=CC=CC=C3
-6.3587	168_CHT	O=C1N(S(C)(=O)=O)CC2=CC=CC(C(OC)=O)=CC21C3=CC=CC=C3
-6.1124	169_CHT	O=C1N(S(C)(=O)=O)CC2=CC=CC(C=C)=CC21C3=CC=CC=C3
-6.4006	170_CHT	O=C1N(S(C)(=O)=O)CC2=CC=CC(C(C)=O)=CC21C3=CC=CC=C3
-6.6902	171_CHT	O=C1N(S(C)(=O)=O)CC2=CC=CC(S(C)(=O)=O)=CC21C3=CC=CC=C3
-6.1407	172_CHT	O=C1N(S(C)(=O)=O)CC2=CC=CC(S)=CC21C3=CC=CC=C3
-5.8888	173_CHT	O=C1N(S(C)(=O)=O)CC2=CC=CC(SC)=CC21C3=CC=CC=C3
-6.2754	174_CHT	O=C1N(S(C)(=O)=O)CC2=CC=CC(S(C)=O)=CC21C3=CC=CC=C3
-6.7473	175_CHT	O=C1N(S(C)(=O)=O)CC2=CC=CC(C#N)=CC21C3=CC=CC=C3
-6.1320	176_CHT	O=C1N(S(C)(=O)=O)CC2=CC=CC(C(C)(C)C)=CC21C3=CC=CC=C3
-6.1508	177_CHT	O=C1N(S(C)(=O)=O)CC2=CC=CC([Si](C)(C)C)=CC21C3=CC=CC=C3
-6.0744	178_CHT	O=C1N(S(C)(=O)=O)CC2=CC(C)=CC=CC21C3=CC=CC=C3
-6.5878	179_CHT	O=C1N(S(C)(=O)=O)CC2=CC(C(F)(F)F)=CC=CC21C3=CC=CC=C3
-6.2178	180_CHT	O=C1N(S(C)(=O)=O)CC2=CC(F)=CC=CC21C3=CC=CC=C3
-6.3111	181_CHT	O=C1N(S(C)(=O)=O)CC2=CC(Br)=CC=CC21C3=CC=CC=C3
-5.9435	182_CHT	O=C1N(S(C)(=O)=O)CC2=CC(C3=CC=CC=C3)=CC=CC21C4=CC=CC=C4
-6.3552	183_CHT	O=C1N(S(C)(=O)=O)CC2=CC(Cl)=CC=CC21C3=CC=CC=C3
-6.8507	184_CHT	O=C1N(S(C)(=O)=O)CC2=CC([N+]([O-])=O)=CC=CC21C3=CC=CC=C3
-5.8123	185_CHT	O=C1N(S(C)(=O)=O)CC2=CC(O)=CC=CC21C3=CC=CC=C3
-6.4069	186_CHT	O=C1N(S(C)(=O)=O)CC2=CC(C(OC)=O)=CC=CC21C3=CC=CC=C3
-5.9530	187_CHT	O=C1N(S(C)(=O)=O)CC2=CC(C=C)=CC=CC21C3=CC=CC=C3
-6.4635	188_CHT	O=C1N(S(C)(=O)=O)CC2=CC(C(C)=O)=CC=CC21C3=CC=CC=C3
-6.8586	189_CHT	O=C1N(S(C)(=O)=O)CC2=CC(S(C)(=O)=O)=CC=CC21C3=CC=CC=C3
-6.1163	190_CHT	O=C1N(S(C)(=O)=O)CC2=CC(S)=CC=CC21C3=CC=CC=C3
-5.9530	191_CHT	O=C1N(S(C)(=O)=O)CC2=CC(SC)=CC=CC21C3=CC=CC=C3
-6.2899	192_CHT	O=C1N(S(C)(=O)=O)CC2=CC(S(C)=O)=CC=CC21C3=CC=CC=C3
-6.7073	193_CHT	O=C1N(S(C)(=O)=O)CC2=CC(C#N)=CC=CC21C3=CC=CC=C3
-6.0836	194_CHT	O=C1N(S(C)(=O)=O)CC2=CC(C(C)(C)C)=CC=CC21C3=CC=CC=C3
-6.1541	195_CHT	O=C1N(S(C)(=O)=O)CC2=CC([Si](C)(C)C)=CC=CC21C3=CC=CC=C3
-6.1639	196_CHT	O=C1N(S(C)(=O)=O)CC2=C(C)C=CC=CC21C3=CC=CC=C3
-6.5797	197_CHT	O=C1N(S(C)(=O)=O)CC2=C(C(F)(F)F)C=CC=CC21C3=CC=CC=C3
-6.3312	198_CHT	O=C1N(S(C)(=O)=O)CC2=C(F)C=CC=CC21C3=CC=CC=C3
-6.4529	199_CHT	O=C1N(S(C)(=O)=O)CC2=C(Br)C=CC=CC21C3=CC=CC=C3
-6.1484	200_CHT	O=C1N(S(C)(=O)=O)CC2=C(C3=CC=CC=C3)C=CC=CC21C4=CC=CC=C4

Supplementary Fig. 26 | List of CHT and NCD's HOBO level (**151_CHT–200_CHT**).

CHT/NCD		
HOBO	NAME	SMILES
-6.4839	201_CHT	O=C1N(S(C)(=O)=O)CC2=C(Cl)C=CC=CC21C3=CC=CC=C3
-6.8755	202_CHT	O=C1N(S(C)(=O)=O)CC2=C([N+]([O-])=O)C=CC=CC21C3=CC=CC=C3
-6.0588	203_CHT	O=C1N(S(C)(=O)=O)CC2=C(O)C=CC=CC21C3=CC=CC=C3
-6.4295	204_CHT	O=C1N(S(C)(=O)=O)CC2=C(C(OC)=O)C=CC=CC21C3=CC=CC=C3
-6.1895	205_CHT	O=C1N(S(C)(=O)=O)CC2=C(C=C)C=CC=CC21C3=CC=CC=C3
-6.4999	206_CHT	O=C1N(S(C)(=O)=O)CC2=C(C(C)=O)C=CC=CC21C3=CC=CC=C3
-6.7231	207_CHT	O=C1N(S(C)(=O)=O)CC2=C(S(C)(=O)=O)C=CC=CC21C3=CC=CC=C3
-6.2839	208_CHT	O=C1N(S(C)(=O)=O)CC2=C(S)C=CC=CC21C3=CC=CC=C3
-6.1780	209_CHT	O=C1N(S(C)(=O)=O)CC2=C(SC)C=CC=CC21C3=CC=CC=C3
-6.4545	210_CHT	O=C1N(S(C)(=O)=O)CC2=C(S(C)=O)C=CC=CC21C3=CC=CC=C3
-6.7642	211_CHT	O=C1N(S(C)(=O)=O)CC2=C(C#N)C=CC=CC21C3=CC=CC=C3
-6.1794	212_CHT	O=C1N(S(C)(=O)=O)CC2=C(C(C)(C)C)C=CC=CC21C3=CC=CC=C3
-6.1663	213_CHT	O=C1N(S(C)(=O)=O)CC2=C([Si](C)(C)C)C=CC=CC21C3=CC=CC=C3
-6.0406	214_CHT	O=C1N(S(C)(=O)=O)CC2=CC(C)=CC(C)=CC21C3=CC=CC=C3
-6.9035	215_CHT	O=C1N(S(C)(=O)=O)CC2=CC(C(F)(F)F)=CC(C(F)(F)F)=CC21C3=CC=CC=C3
-6.3818	216_CHT	O=C1N(S(C)(=O)=O)CC2=CC(F)=CC(F)=CC21C3=CC=CC=C3
-6.5364	217_CHT	O=C1N(S(C)(=O)=O)CC2=CC(Br)=CC(Br)=CC21C3=CC=CC=C3
-5.9236	218_CHT	O=C1N(S(C)(=O)=O)CC2=CC(C3=CC=CC=C3)=CC(C4=CC=CC=C4)=CC21C5=CC=CC=C5
-6.6061	219_CHT	O=C1N(S(C)(=O)=O)CC2=CC(Cl)=CC(Cl)=CC21C3=CC=CC=C3
-7.2523	220_CHT	O=C1N(S(C)(=O)=O)CC2=CC([N+]([O-])=O)=CC([N+]([O-])=O)=CC21C3=CC=CC=C3
-5.7739	221_CHT	O=C1N(S(C)(=O)=O)CC2=CC(O)=CC(O)=CC21C3=CC=CC=C3
-6.5533	222_CHT	O=C1N(S(C)(=O)=O)CC2=CC(C(OC)=O)=CC(C(OC)=O)=CC21C3=CC=CC=C3
-5.9241	223_CHT	O=C1N(S(C)(=O)=O)CC2=CC(C=C)=CC(C=C)=CC21C3=CC=CC=C3
-6.6572	224_CHT	O=C1N(S(C)(=O)=O)CC2=CC(C(C)=O)=CC(C(C)=O)=CC21C3=CC=CC=C3
-7.1508	225_CHT	O=C1N(S(C)(=O)=O)CC2=CC(S(C)(=O)=O)=CC(S(C)(=O)=O)=CC21C3=CC=CC=C3
-6.1944	226_CHT	O=C1N(S(C)(=O)=O)CC2=CC(S)=CC(S)=CC21C3=CC=CC=C3
-5.9633	227_CHT	O=C1N(S(C)(=O)=O)CC2=CC(SC)=CC(SC)=CC21C3=CC=CC=C3
-6.4705	228_CHT	O=C1N(S(C)(=O)=O)CC2=CC(S(C)=O)=CC(S(C)=O)=CC21C3=CC=CC=C3
-7.1517	229_CHT	O=C1N(S(C)(=O)=O)CC2=CC(C#N)=CC(C#N)=CC21C3=CC=CC=C3
-6.0392	230_CHT	O=C1N(S(C)(=O)=O)CC2=CC(C(C)(C)C)=CC(C(C)(C)C)=CC21C3=CC=CC=C3
-6.1282	231_CHT	O=C1N(S(C)(=O)=O)CC2=CC([Si](C)(C)C)=CC([Si](C)(C)C)=CC21C3=CC=CC=C3
-6.0335	232_CHT	O=C1N(S(C)(=O)=O)CC2=C(C)C=CC=C(C)C21C3=CC=CC=C3
-6.8934	233_CHT	O=C1N(S(C)(=O)=O)CC2=C(C(F)(F)F)C=CC=C(C(F)(F)F)C21C3=CC=CC=C3
-6.3614	234_CHT	O=C1N(S(C)(=O)=O)CC2=C(F)C=CC=C(F)C21C3=CC=CC=C3
-6.4409	235_CHT	O=C1N(S(C)(=O)=O)CC2=C(Br)C=CC=C(Br)C21C3=CC=CC=C3
-5.8297	236_CHT	O=C1N(S(C)(=O)=O)CC2=C(C3=CC=CC=C3)C=CC=C(C4=CC=CC=C4)C21C5=CC=CC=C5
-6.5533	237_CHT	O=C1N(S(C)(=O)=O)CC2=C(Cl)C=CC=C(Cl)C21C3=CC=CC=C3
-7.2047	238_CHT	O=C1N(S(C)(=O)=O)CC2=C([N+]([O-])=O)C=CC=C([N+]([O-])=O)C21C3=CC=CC=C3
-5.8101	239_CHT	O=C1N(S(C)(=O)=O)CC2=C(O)C=CC=C(O)C21C3=CC=CC=C3
-6.6088	240_CHT	O=C1N(S(C)(=O)=O)CC2=C(C(OC)=O)C=CC=C(C(OC)=O)C21C3=CC=CC=C3
-5.8977	241_CHT	O=C1N(S(C)(=O)=O)CC2=C(C=C)C=CC=C(C=C)C21C3=CC=CC=C3
-6.6447	242_CHT	O=C1N(S(C)(=O)=O)CC2=C(C(C)=O)C=CC=C(C(C)=O)C21C3=CC=CC=C3
-6.8915	243_CHT	O=C1N(S(C)(=O)=O)CC2=C(S(C)(=O)=O)C=CC=C(S(C)(=O)=O)C21C3=CC=CC=C3
-5.9824	244_CHT	O=C1N(S(C)(=O)=O)CC2=C(S)C=CC=C(S)C21C3=CC=CC=C3
-5.7440	245_CHT	O=C1N(S(C)(=O)=O)CC2=C(SC)C=CC=C(SC)C21C3=CC=CC=C3
-6.1686	246_CHT	O=C1N(S(C)(=O)=O)CC2=C(S(C)=O)C=CC=C(S(C)=O)C21C3=CC=CC=C3
-7.1093	247_CHT	O=C1N(S(C)(=O)=O)CC2=C(C#N)C=CC=C(C#N)C21C3=CC=CC=C3
-6.0213	248_CHT	O=C1N(S(C)(=O)=O)CC2=C(C(C)(C)C)C=CC=C(C(C)(C)C)C21C3=CC=CC=C3
-6.0983	249_CHT	O=C1N(S(C)(=O)=O)CC2=C([Si](C)(C)C)C=CC=C([Si](C)(C)C)C21C3=CC=CC=C3

Supplementary Fig. 27 | List of CHT and NCD's HOBO level (**201_CHT–249_CHT**).

Enophile		
LUBO	NAME	SMILES
-1.4969	001_EP	O=C(C#CC(OC)=O)OC
-3.1886	002_EP	O=C1OC(C=C1)=O
-3.8983	003_EP	O=C1N(C2=CC=CC=C2)C(N=N1)=O
-2.6871	004_EP	O=NC1=CC=CC=C1
0.9303	005_EP	C1=CCCCC1
-2.8256	006_EP	CN(S(C)(=O)=O)C#CC1=CC=CC=C1
-0.8073	007_EP	O=C(N=NC(C)=O)C
-1.2517	008_EP	C1(C#CC2=CC=CC=C2)=CC=CC=C1
1.8511	009_EP	CC#CC
-0.6568	010_EP	N#N
1.0027	011_EP	C/C=C¥C
0.4683	012_EP	C1C=C1
-1.3317	013_EP	C/C=C¥C(C)=O
-0.7181	014_EP	C/C=C¥C(N)=O
-0.5540	015_EP	C/C=C¥C(N(C)C)=O
-1.3728	016_EP	C/C=C¥C(N(S(C)(=O)=O)C)=O
-0.4781	017_EP	C/C(C)=C¥C(N(C)C)=O
0.1613	018_EP	C/C=C(C)¥C(N(C)C)=O
-1.1638	019_EP	C/C=C¥C(O)=O
-1.0272	020_EP	C/C=C¥C(OC)=O
-1.6800	021_EP	C/C=C¥C([H])=O
-1.0337	022_EP	C=C=CC(OC)=O
-1.0596	023_EP	O=C(C=C=CC1=CC=CC=C1)OC
0.0976	024_EP	C1=CC=CC=C1
-0.9589	025_EP	C1(C=CC=C2)=C2C=CC=C1
-1.6321	026_EP	C1(C=C(C=CC=C2)C2=C3)=C3C=CC=C1
-0.4432	027_EP	C1(C=CO2)=C2C=CC=C1
-0.0658	028_EP	C1(C=CN2)=C2C=CC=C1
-0.9393	029_EP	O=C(N1C=CC2=C1C=CC=C2)C
-0.0413	030_EP	CN1C=CC2=C1C=CC=C2
-0.6495	031_EP	CS(N1C=CC2=C1C=CC=C2)(=O)=O
0.0354	032_EP	OC1=CC=CC=C1
1.3856	033_EP	C1=CC=CN1
0.5355	034_EP	C1=CC=CO1
-0.6125	035_EP	C1=CC=CC=N1
-1.9083	036_EP	C1#CC=CC=C1
-1.7717	037_EP	CC1=CC=CC#C1
-1.8531	038_EP	CC1=CC#CC=C1
-1.5603	039_EP	CC1=C(C)C(C)=C(C)C#C1
-2.4016	040_EP	BrC1=CC=CC#C1
-2.2863	041_EP	FC1=CC=CC#C1
-0.8111	042_EP	FC1=C(F)C(F)=C(F)C(F)=C1F
-1.9956	043_EP	O=C(C#CC(C(C)C)=O)C(C)C
-2.4865	044_EP	O=C(C#CC(C1=CC=CC=C1)=O)C2=CC=CC=C2
-2.0030	045_EP	O=C(C#CC(CC1=CC=CC=C1)=O)CC2=CC=CC=C2
-3.3015	046_EP	O=C(C#CC(C(F)(F)F)=O)C(F)(F)F
-1.7840	047_EP	O=C(C#CC(O)=O)O
-2.3946	048_EP	O=C(N=NC(C(C)C)=O)C(C)C
-2.8582	049_EP	O=C(N=NC(C1=CC=CC=C1)=O)C2=CC=CC=C2
-2.4321	050_EP	O=C(N=NC(CC1=CC=CC=C1)=O)CC2=CC=CC=C2

Supplementary Fig. 28 | List of enophile's LUBO level (**001_EP–050_EP**).

		Enophile
LUBO	NAME	SMILES
-4.3081	051_EP	O=C(N=NC(C(F)(F)F)=O)C(F)(F)F
-2.6360	052_EP	O=C(N=NC(O)=O)O
-2.5527	053_EP	O=NC1=CC=C(C)C=C1
-2.7521	054_EP	O=NC1=CC=C(F)C=C1
-2.3684	055_EP	O=NC1=CC=C(OC)C=C1
-2.9227	056_EP	O=NC1=CC=C(Br)C=C1
-3.4286	057_EP	O=NC1=CC=C(C#N)C=C1
-3.8580	058_EP	O=C1N(C)C(N=N1)=O
-4.0319	059_EP	O=C1N(C=C)C(N=N1)=O
-4.1834	060_EP	O=C1N(C(C)=O)C(N=N1)=O
-3.7761	061_EP	O=C1N(CC2=CC=CC=C2)C(N=N1)=O
-4.2580	062_EP	O=C1N(S(C)(=O)=O)C(N=N1)=O
-3.9546	063_EP	O=C1N(C(OC(C)(C)C)=O)C(N=N1)=O
-3.7971	064_EP	O=C1CC(N=N1)=O
-3.6531	065_EP	O=C1C(C)(C)C(N=N1)=O
-1.8813	066_EP	C12=C(C=CC=C2)C=CC#C1
-1.8296	067_EP	C12=C(C=CC=C2)C3=C(C=CC=C3)C#C1
-1.7869	068_EP	CC1=CC#CC=C1C
-1.6669	069_EP	CC1=C(C)C#CC=C1C
-1.7244	070_EP	CC1=CC#CC(C)=C1
-1.6324	071_EP	CC1=C(C)C#CC(C)=C1
-3.3695	072_EP	FC1=C(F)C#CC(F)=C1F
-2.8269	073_EP	FC1=CC=C(F)C#C1
-1.2585	074_EP	CC#CC(C(C)C)=O
-2.1363	075_EP	O=C(C#CC1=CC=CC=C1)C2=CC=CC=C2
-2.0473	076_EP	O=C(C#CC(CC1=CC=CC=C1)=O)C
-2.1739	077_EP	O=C(C#CC1CCCCC1)C(F)(F)F
-2.8639	078_EP	N#CC#CC(O)=O
-1.8294	079_EP	CN=NC(C(C)C)=O
-2.5554	080_EP	O=C(N=NC1=CC=CC=C1)C2=CC=CC=C2
-2.6920	081_EP	O=C(/N=N¥C(CC1=CC=CC=C1)=O)C
-2.4498	082_EP	O=C(N=NC1CCCCC1)C(F)(F)F
-3.8504	083_EP	N#CN=NC(O)=O
-2.2858	084_EP	O=NC
-2.0955	085_EP	O=NC1CCCCC1
-2.6433	086_EP	O=NC1=CC=C([Si](C)(C)C)C=C1
-2.6405	087_EP	O=NC1=C(CC)C=CC=C1
-3.0207	088_EP	O=NC1=CC=CC=C1C(F)(F)F
-4.0003	089_EP	O=C1NC(N=N1)=O
-2.6218	090_EP	O=C1N(C)C(C=C1)=O
-3.0106	091_EP	O=C1N(C(C)=O)C(C=C1)=O
-2.7219	092_EP	O=C1N([H])C(C=C1)=O
-2.7296	093_EP	O=C1N(C2=CC=CC=C2)C(C=C1)=O
-2.8158	094_EP	O=C1N(C(OC(C)(C)C)=O)C(C=C1)=O
-2.6288	095_EP	O=C1N(CC2=CC=CC=C2)C(C=C1)=O
-2.5973	096_EP	O=C1N(CC)C(C=C1)=O
-1.7508	097_EP	C12=C(CCC2)C#CC=C1
-1.8038	098_EP	C12=C(C=CN2)C=CC#C1
-2.1415	099_EP	FC1=CC#CC=C1C
-2.6161	100_EP	CC1=C(C#N)C#CC=C1

Supplementary Fig. 29 | List of enophile's LUBO level (**051_EP–100_EP**).

Enophile		
LUBO	NAME	SMILES
-2.2482	101_EP	BrC1=CC#CC(C)=C1
-1.9056	102_EP	COC1=CC#CC=C1
-1.9320	103_EP	COC1=CC=CC#C1
-2.1317	104_EP	FC1=CC=C(C)C#C1
-0.9170	105_EP	C1#CCCCC1
-1.5034	106_EP	ClC1C#CCCC1
-1.1864	107_EP	OC1C#CCCC1
-1.2979	108_EP	SC1C#CCCC1
-0.8489	109_EP	C[Si](C1C#CCCC1)(C)C
-0.9004	110_EP	CC1C#CCCC1
-1.0147	111_EP	CN(C1C#CCCC1)C
-1.2672	112_EP	ClC1CC#CCC1
-1.1439	113_EP	OC1CC#CCC1
-1.2971	114_EP	SC1CC#CCC1
-0.8291	115_EP	C[Si](C1CC#CCC1)(C)C
-0.9461	116_EP	CC1CC#CCC1
-1.0302	117_EP	CN(C1CC#CCC1)C
-0.5129	118_EP	C1=CC=CCCC1
-0.4112	119_EP	C1=CC=CCC1
-0.6844	120_EP	C/C=C¥C=C
-0.5222	121_EP	C/C=C(C)¥C=C
0.0767	122_EP	C/C=C(C)¥C=C¥O[Si](C)(C)C
-0.5007	123_EP	C/C=C(C)¥C=C¥F
-0.8234	124_EP	C/C=C(Cl)¥C=C¥C
-0.8512	125_EP	ClC1=CCCC(F)=C1
-0.8683	126_EP	ClC1=CC(C)CC(F)=C1
-1.2721	127_EP	C/C=C/C(C(OC)=O)=C
-1.1387	128_EP	CC(C=C=C([Si](C)(C)C)C1=CC=CC=C1)=O
-1.7284	129_EP	CC(C=C=C(C(C)=O)C1=CC=CC=C1)=O
-1.0601	130_EP	O=C(C=C=CC1=CC=CC=C1)OC
-1.4035	131_EP	CC(C=C=C(C1=CC=CC=C1)C2=CC=CC=C2)=O
-1.2207	132_EP	CC(C=C=C(C)C1=CC=CC=C1)=O
-0.5355	133_EP	C12=CC=C(CCCCC2)C=C1
-1.0487	134_EP	ClC1=CC2=CC=C1CCCCC2
-0.5712	135_EP	OC1=CC2=CC=C1CCCCC2
-0.9021	136_EP	SC1=CC2=CC=C1CCCCC2
-0.6136	137_EP	C[Si](C1=CC2=CC=C1CCCCC2)(C)C
-0.5029	138_EP	CC1=CC2=CC=C1CCCCC2
-1.5066	139_EP	N#CC1=CC2=CC=C1CCCCC2
-0.8133	140_EP	FC1=CC2=CC=C1CCCCC2
-0.8841	141_EP	C12=CC=C(CCCCC2)C(C3=CC=CC=C3)=C1
-0.3415	142_EP	ClC1=CC=CC=C1
-0.1363	143_EP	SC1=CC=CC=C1
-0.1635	144_EP	C[Si](C1=CC=CC=C1)(C)C
0.1453	145_EP	CC1=CC=CC=C1
-1.4114	146_EP	N#CC1=CC=CC=C1
-0.2353	147_EP	FC1=CC=CC=C1
-0.6762	148_EP	C1(C2=CC=CC=C2)=CC=CC=C1
-0.0525	149_EP	C1(CC2=CC=CC=C2)=CC=CC=C1
-1.4724	150_EP	O=C(C1=CC=CC=C1)C

Supplementary Fig. 30 | List of enophile's LUBO level (**101_EP–150_EP**).

		Enophile
LUBO	NAME	SMILES
-0.7241	151_EP	FC(C1=CC=CC=C1)(F)F
-2.4283	152_EP	O=[N+](C1=CC=CC=C1)[O-]
-0.3431	153_EP	BrC1=CC=CC=C1
0.1420	154_EP	CCC1=CC=CC=C1
-1.2253	155_EP	C/C(C)=C¥C(C)=O
-1.1875	156_EP	C/C([Si](C)(C)C)=C(C)¥C(C)=O
-1.0084	157_EP	C/C(C)=C¥C(C)=O
-1.7058	158_EP	CC(/C=C¥[Si](C)(C)C)=O
-1.4269	159_EP	F/C=C¥C(C)=O
-1.9105	160_EP	CC(/C=C¥C1=CC=CC=C1)=O
-1.8998	161_EP	CC(/C=C¥C(C)=O)=O
-2.2316	162_EP	CC(/C=C¥C(F)(F)F)=O
-1.8035	163_EP	C/C=C(C(C)=O)¥C(C)=O
-1.4310	164_EP	S/C=C¥C(C)=O
-2.2786	165_EP	CC(/C=C¥[N+]([O-])=O)=O
-0.8898	166_EP	CC(/C=C¥OC)=O
0.4865	167_EP	CC1C=CCO1
0.4628	168_EP	CCC1C=CCO1
0.4587	169_EP	CC(C)C1C=CCO1
0.4359	170_EP	CC(C)(C)C1C=CCO1
-0.0764	171_EP	FC(F)(F)C1C=CCO1
-0.0008	172_EP	FC(F)C1C=CCO1
0.2429	173_EP	FCC1C=CCO1
-0.1028	174_EP	FC1C=CCO1
-0.3594	175_EP	BrCC1C=CCO1
-0.9915	176_EP	BrC1C=CCO1
-0.7096	177_EP	ClC1C=CCO1
0.5455	178_EP	C[Si](C1C=CCO1)(C)C
-0.1453	179_EP	SC1C=CCO1
0.0111	180_EP	CSC1C=CCO1
-0.3483	181_EP	O=S(C)C1C=CCO1
-0.4585	182_EP	CS(C1C=CCO1)(=O)=O
0.2511	183_EP	COC1C=CCO1
0.2119	184_EP	OC1C=CCO1
0.9962	185_EP	N#CC
0.8966	186_EP	N#CCC
0.9744	187_EP	N#CC(C)C
1.1616	188_EP	N#CC(C)(C)C
-1.4117	189_EP	N#CC1=CC=CC=C1
0.3505	190_EP	N#CBr
1.0784	191_EP	N#CF
0.4346	192_EP	N#CCl
-0.3684	193_EP	N#CCF
-0.5821	194_EP	N#CC(F)F
-0.7611	195_EP	N#CC(F)(F)F
-1.1804	196_EP	N#CCCl
-1.6193	197_EP	N#CCBr
0.2182	198_EP	N#C[Si](C)(C)C
-1.2631	199_EP	N#CS(C)(=O)=O
1.1265	200_EP	N#CN(C)C

Supplementary Fig. 31 | List of enophile's LUBO level (**151_EP–200_EP**).

		Enophile
LUBO	NAME	SMILES
1.3510	201_EP	N#CO
-0.5940	202_EP	O=CC
-0.5202	203_EP	O=CCC
-0.5717	204_EP	O=CC(C)C
-0.5379	205_EP	O=CC(C)(C)C
-1.7115	206_EP	O=CC1=CC=CC=C1
-1.6452	207_EP	O=CBr
-0.7945	208_EP	O=CF
-1.5815	209_EP	O=CCl
-0.9779	210_EP	O=CCF
-1.7684	211_EP	O=CC(F)F
-2.0422	212_EP	O=CC(F)(F)F
-1.4011	213_EP	O=CCCl
-1.6389	214_EP	O=CCBr
-0.5782	215_EP	O=CCO
-1.0882	216_EP	O=C[Si](C)(C)C
-0.7625	217_EP	O=COS(C)(=O)=O
0.8764	218_EP	O=CN(C)C
0.0016	219_EP	O=CO
0.9206	220_EP	C/C(C)=C(C)/C
0.7698	221_EP	CC/C(CC)=C(CC)/CC
-1.2035	222_EP	C(/C1=CC=CC=C1)(C2=CC=CC=C2)=C(C3=CC=CC=C3)¥C4=CC=CC=C4
0.8163	223_EP	F/C(F)=C(F)/F
-1.0949	224_EP	Cl/C(Cl)=C(Cl)/Cl
-1.0580	225_EP	Br/C(Br)=C(Br)/Br
1.3273	226_EP	CO/C(OC)=C(OC)/OC
1.6577	227_EP	N/C(N)=C(N)/N
-2.9016	228_EP	O=C(/C(C(O)=O)=C(C(O)=O)/C(O)=O)O
-2.9897	229_EP	FC(/C(C(F)(F)F)=C(C(F)(F)F)/C(F)(F)F)(F)F
-2.3184	230_EP	O=C(/C(C(C)=O)=C(C(C)=O)/C(C)=O)C
1.0340	231_EP	C/C(C)=C/C
0.8310	232_EP	CC/C(CC)=C/CC
-1.2416	233_EP	C(/C1=CC=CC=C1)(C2=CC=CC=C2)=C¥C3=CC=CC=C3
0.7246	234_EP	F/C(F)=C/F
-0.8030	235_EP	Cl/C(Cl)=C/Cl
-0.8027	236_EP	Br/C(Br)=C/Br
1.3085	237_EP	CO/C(OC)=C/OC
1.7556	238_EP	N/C(N)=C/N
-2.7241	239_EP	O=C(/C(C(O)=O)=C/C(O)=O)O
-2.2392	240_EP	FC(/C(C(F)(F)F)=C/C(F)(F)F)(F)F
-2.7601	241_EP	O=C(/C(C(C)=O)=C/C(C)=O)C
0.8482	242_EP	C=C(C)C
0.7763	243_EP	C=C(CC)CC
-0.8427	244_EP	C=C(C1=CC=CC=C1)C2=CC=CC=C2
0.7540	245_EP	C=C(F)F
-0.4585	246_EP	C=C(Cl)Cl
-0.4773	247_EP	C=C(Br)Br
1.6707	248_EP	C=C(OC)OC
1.5891	249_EP	C=C(N)N
-1.9739	250_EP	C=C(C(O)=O)C(O)=O

Supplementary Fig. 32 | List of enophile's LUBO level (**201_EP–250_EP**).

		Enophile
LUBO	NAME	SMILES
-1.3515	251_EP	C=C(C(F)(F)F)C(F)(F)F
-2.2057	252_EP	C=C(C(C)=O)C(C)=O
0.9875	253_EP	C/C=C/C
0.8446	254_EP	CC/C=C/CC
-1.3573	255_EP	C1(/C=C/C2=CC=CC=C2)=CC=CC=C1
0.5706	256_EP	F/C=C/F
-0.5200	257_EP	Cl/C=C/Cl
-0.5494	258_EP	Br/C=C/Br
1.2008	259_EP	CO/C=C/OC
1.5271	260_EP	N/C=C/N
-2.5170	261_EP	O=C(/C=C/C(O)=O)O
-1.4237	262_EP	FC(/C=C/C(F)(F)F)(F)F
-2.5524	263_EP	O=C(/C=C/C(C)=O)C
-0.3967	264_EP	C[Si](/C=C¥[Si](C)(C)C)(C)C
0.8479	265_EP	CC/C=C¥CC
-1.1502	266_EP	C1(/C=C¥C2=CC=CC=C2)=CC=CC=C1
0.6449	267_EP	F/C=C¥F
-0.3981	268_EP	Cl/C=C¥Cl
-0.4430	269_EP	Br/C=C¥Br
1.3481	270_EP	CO/C=C¥OC
1.6054	271_EP	N/C=C¥N
-1.9706	272_EP	O=C(/C=C¥C(O)=O)O
-1.5690	273_EP	FC(/C=C¥C(F)(F)F)(F)F
-1.8996	274_EP	O=C(/C=C¥C(C)=O)C
0.7701	275_EP	C=CC
0.7042	276_EP	C=CCC
-0.8307	277_EP	C=CC1=CC=CC=C1
0.6000	278_EP	C=CF
-0.0337	279_EP	C=CCl
-0.0740	280_EP	C=CBr
1.0460	281_EP	C=COC
1.3945	282_EP	C=CN
-1.3771	283_EP	C=CC(O)=O
-0.5499	284_EP	C=CC(F)(F)F
-1.4822	285_EP	C=CC(C)=O
-0.7621	286_EP	CN(S(C)(=O)=O)C#CC1=CC=CC=C1
0.5793	287_EP	CN(S(C)(=O)=O)C#CC
0.6179	288_EP	CN(S(C)(=O)=O)C#CCCCC
0.9262	289_EP	OC#CN(C)S(C)(=O)=O
0.3268	290_EP	[H]C#CN(C)S(C)(=O)=O
-0.0653	291_EP	CN(S(C)(=O)=O)C#C[Si](C)(C)C
-1.2054	292_EP	CN(S(C1=CC=C(C)C=C1)(=O)=O)C#CC2=CC=CC=C2
0.3172	293_EP	CN(C(C)=O)C#CC
0.9523	294_EP	CN(C(OC(C)(C)C)=O)C#CCCCC
1.0038	295_EP	OC#CN(C)C(C1=CC=CC=C1)=O
1.5301	296_EP	[H]C#CN(C)[H]
0.8664	297_EP	CN(C)C#C[Si](C)(C)C
-1.4258	298_EP	CS(N(S(C)(=O)=O)C#CC1=CC=CC=C1)(=O)=O
-0.5496	299_EP	CC#CN(C(C)=O)S(C)(=O)=O
-0.1338	300_EP	CCCCC#CN(C(OC(C)(C)C)=O)S(C)(=O)=O
0.0460	301_EP	OC#CN(C(C1=CC=CC=C1)=O)S(C)(=O)=O
0.4117	302_EP	[H]C#CN(C1=CC=CC=C1)S(C)(=O)=O
-0.0122	303_EP	C[Si](C#CN(CC1=CC=CC=C1)S(C)(=O)=O)(C)C

Supplementary Fig. 33 | List of enophile's LUBO level (**251_EP–303_EP**).

```python
# Tsubasa Ito, Chiba Univ.
import os
import pandas as pd
from multiprocessing import Pool, get_context
import warnings
import traceback
import itertools
from sklearn.model_selection import train_test_split
from sklearn.model_selection import KFold
from sklearn.model_selection import cross_validate
from sklearn.neural_network  import MLPRegressor

##############################################################################################################
fp_list = ["EP_Avalon_1024.csv" , "EP_Avalon_2048.csv" , "EP_Avalon_4096.csv" ,
           "EP_Morgan2_1024.csv", "EP_Morgan2_2048.csv", "EP_Morgan2_4096.csv",
           "EP_Morgan3_1024.csv", "EP_Morgan3_2048.csv", "EP_Morgan3_4096.csv",
           "EP_Morgan4_1024.csv", "EP_Morgan4_2048.csv", "EP_Morgan4_4096.csv",
           "EP_topo.csv", "EP_MACCS.csv"]

path_Current = os.path.dirname(os.path.abspath(__file__)) + "/"
path_Result = path_Current + "Result/"
path_Csv = path_Current + "csv/"

ModelName = "NN"
coment_01 = "------------------------------------------------"
coment_02 = "\nBest Data"

hidden_layer1 = range(10,31)
hidden_layer2 = range(1,21)
hidden_layer3 = range(1,21)

range_a = range(4)
patterns = list(itertools.product(hidden_layer1, hidden_layer2, hidden_layer3))
range_b = range(len(patterns))

num = "1st_"
sortsecond = (lambda val : val[1])
warnings.filterwarnings('ignore')
os.makedirs(path_Result, exist_ok=True)
##############################################################################################################

# def1 Writing result of tuning on .txt files
def Writeindex(csv, content = ModelName):
    f = open(path_Result  + num + ModelName + "_" + csv.split(".")[0] + '.txt', 'a')
    f.write(str(content) + "\n")
    f.close

# def2 Writing details of error
def WriteError():
    f = open(path_Result + 'error.txt', 'a')
    f.write(str(traceback.format_exc()))
    f.close
```

```
# def3 Creating feature values and answer data from a data frame
def Mk_xt(fn, df):
    x = df.loc[:,str(0):str(int(fn)-1)]
    t = df['LUBO']

    return x,t

# def4 Preparing x, t from csv file
def Data_set(csv):
    Writeindex(csv) # def1
    cf = path_Csv + csv # Csv File
    df = pd.read_csv(cf) # Extracting data frame from csv
    fn = int(df.shape[-1] - 4) # Extract number of finger print
    train_val, test = train_test_split(df, test_size = 0.20, random_state = 0)
    x,t = Mk_xt(fn, train_val) # def3

    return x, t

# def5 Detailed Machine learning model
def ML(x,t,a,b):
    activation_list = ["identity", "logistic", "tanh", "relu"]

    activation_type = activation_list[int(a)]
    layer1 = int(b[0]) * 5
    layer2 = int(b[1]) * 5
    layer3 = int(b[2]) * 5
    layer_list = [layer1, layer2, layer3]

    model = MLPRegressor(hidden_layer_sizes= (layer1, layer2, layer3), activation= activation_type, solver= "lbfgs", alpha= 0.001, batch_size= 'auto', learning_rate= "constant",
        learning_rate_init= 0.001, power_t= 0.5, max_iter= 200, shuffle= True, random_state= 0, tol= 0.0001, verbose= False, warm_start= False, momentum= 0.9,
        nesterovs_momentum= True, early_stopping= True, validation_fraction= 0.1, beta_1= 0.9, beta_2= 0.999, epsilon= 1e-08, n_iter_no_change= 10, max_fun= 15000)

    kf = KFold(n_splits = 3, shuffle = True, random_state = 0)
    result = cross_validate(model,x,t,cv = kf, scoring = {'r2','neg_mean_absolute_error'}, return_train_score = True)
    train_average = sum(result['train_r2']) /len(result['train_r2'])
    result_average = sum(result['test_r2']) /len(result['test_r2'])
    MAE_average = 0 - float(sum(result['test_neg_mean_absolute_error']) /len(result['test_neg_mean_absolute_error']))

    Result_list = [train_average, result_average, MAE_average, activation_type, layer_list]

    return Result_list

# def6 Supporting function for p.map()
def Wrapper(arg): return  ML(*arg) # def5

# def7 Running Model tuneing processes in parallel
def ParallelProcessing(values, range_01 = 20):
    p = get_context('fork').Pool(range_01)
    list_1= p.map(Wrapper, values)
```

```python
    p.close()

    return list_1

# def8 Writing result of tuning on .txt files
def Write_result(list1, sorting = "True", list3 = "None"):
    if sorting == "True":
        list1 = sorted(list1, reverse=True, key=sortsecond)
        list2 = list1[0]

    else:
        list2 = list1

    A = list2[0]
    B = list2[1]
    C = list2[2]
    D = list2[3]
    E = list2[4]

    coment_A = f"R2 of Train Data   :{A}"
    coment_B = f"R2 of Test  Data   :{B}"
    coment_C = f"MAE of Test Data   :{C}"
    coment_D = f"activation         :{D}"
    coment_E = f"hidden_layer_sizes :{E}"

    Writeindex(csv, coment_01)
    if list3 == "None": Writeindex(csv, coment_02)
    else: list3.append(list2)

    Writeindex(csv, coment_A)
    Writeindex(csv, coment_B)
    Writeindex(csv, coment_C)
    Writeindex(csv, coment_D)
    Writeindex(csv, coment_E)

    return list3

if __name__ =='__main__' :
    for csv in fp_list:
        Data_list = []
        print(csv.split(".")[0], "processing .", end='') # Showing progress
        x, t = Data_set(csv) # def4

        # Machine Learning
        for a in range_a:
            print('.', end='') # Showing progress
            values = [(x, t, a, patterns[b]) for b in range_b]
            list_A = ParallelProcessing(values) # def7
            for i in list_A:
                Data_list = Write_result(i, "False", Data_list) # def8

        try:
            Write_result(Data_list) # def8
            print('Success !!') # Showing progress

        except:
            WriteError() # def2
```

Supplementary Fig. 34 | Source code for preliminary study of neural network model for predicting the LUBO of enophiles.

```python
# Tsubasa Ito, Chiba Univ.
import os
import pandas as pd
from multiprocessing import Pool, get_context
import warnings
import traceback
import itertools
from sklearn.model_selection import train_test_split
from sklearn.model_selection import KFold
from sklearn.model_selection import cross_validate
from sklearn.neural_network  import MLPRegressor

##############################################################################################################
fp_list = ["CHT_Avalon_1024.csv" , "CHT_Avalon_2048.csv" , "CHT_Avalon_4096.csv" ,
           "CHT_Morgan2_1024.csv", "CHT_Morgan2_2048.csv", "CHT_Morgan2_4096.csv",
           "CHT_Morgan3_1024.csv", "CHT_Morgan3_2048.csv", "CHT_Morgan3_4096.csv",
           "CHT_Morgan4_1024.csv", "CHT_Morgan4_2048.csv", "CHT_Morgan4_4096.csv",
           "CHT_topo.csv", "CHT_MACCS.csv"]

path_Current = os.path.dirname(os.path.abspath(__file__)) + "/"
path_Result = path_Current + "Result/"
path_Csv = path_Current + "csv/"

ModelName = "NN"
coment_01 = "------------------------------------------------"
coment_02 = "\nBest Data"

hidden_layer1 = range(10,31)
hidden_layer2 = range(1,21)
hidden_layer3 = range(1,21)

range_a = range(4)
patterns = list(itertools.product(hidden_layer1, hidden_layer2, hidden_layer3))
range_b = range(len(patterns))

num = "1st_"
sortsecond = (lambda val : val[1])
warnings.filterwarnings('ignore')
os.makedirs(path_Result, exist_ok=True)
##############################################################################################################

# def1 Writing result of tuning on .txt files
def Writeindex(csv, content = ModelName):
    f = open(path_Result  + num + ModelName + "_" + csv.split(".")[0] + '.txt', 'a')
    f.write(str(content) + "\n")
    f.close

# def2 Writing details of error
def WriteError():
    f = open(path_Result + 'error.txt', 'a')
    f.write(str(traceback.format_exc()))
    f.close
```

```python

# def3 Creating feature values and answer data from a data frame
def Mk_xt(fn, df):
    x = df.loc[:,str(0):str(int(fn)-1)]
    t = df['HOBO']

    return x,t

# def4 Preparing x, t from csv file
def Data_set(csv):
    Writeindex(csv) # def1
    cf = path_Csv + csv # Csv File
    df = pd.read_csv(cf) # Extracting data frame from csv
    fn = int(df.shape[-1] - 4) # Extract number of finger print
    train_val, test = train_test_split(df, test_size = 0.20, random_state = 0)
    x,t = Mk_xt(fn, train_val) # def3

    return x, t

# def5 Detailed Machine learning model
def ML(x,t,a,b):
    activation_list = ["identity", "logistic", "tanh", "relu"]

    activation_type = activation_list[int(a)]
    layer1 = int(b[0]) * 5
    layer2 = int(b[1]) * 5
    layer3 = int(b[2]) * 5
    layer_list = [layer1, layer2, layer3]

    model = MLPRegressor(hidden_layer_sizes= (layer1, layer2, layer3), activation= activation_type, solver= "lbfgs", alpha= 0.001, batch_size= 'auto', learning_rate= "constant",
        learning_rate_init= 0.001, power_t= 0.5, max_iter= 200, shuffle= True, random_state= 0, tol= 0.0001, verbose= False, warm_start= False, momentum= 0.9,
        nesterovs_momentum= True, early_stopping= True, validation_fraction= 0.1, beta_1= 0.9, beta_2= 0.999, epsilon= 1e-08, n_iter_no_change= 10, max_fun= 15000)

    kf = KFold(n_splits = 3, shuffle = True, random_state = 0)
    result = cross_validate(model,x,t,cv = kf, scoring = {'r2','neg_mean_absolute_error'}, return_train_score = True)
    train_average = sum(result['train_r2']) /len(result['train_r2'])
    result_average = sum(result['test_r2']) /len(result['test_r2'])
    MAE_average = 0 - float(sum(result['test_neg_mean_absolute_error']) /len(result['test_neg_mean_absolute_error']))

    Result_list = [train_average, result_average, MAE_average, activation_type, layer_list]

    return Result_list

# def6 Supporting function for p.map()
def Wrapper(arg): return  ML(*arg) # def5-1

# def7 Running Model tuneing processes in parallel
def ParallelProcessing(values, range_01 = 20):
    p = get_context('fork').Pool(range_01)
    list_1= p.map(Wrapper, values)
```

```
    p.close()

    return list_1

# def8 Writing result of tuning on .txt files
def Write_result(list1, sorting = "True", list3 = "None"):
    if sorting == "True":
        list1 = sorted(list1, reverse=True, key=sortsecond)
        list2 = list1[0]

    else:
        list2 = list1

    A = list2[0]
    B = list2[1]
    C = list2[2]
    D = list2[3]
    E = list2[4]

    coment_A = f"R2 of Train Data   :{A}"
    coment_B = f"R2 of Test  Data   :{B}"
    coment_C = f"MAE of Test Data   :{C}"
    coment_D = f"activation         :{D}"
    coment_E = f"hidden_layer_sizes :{E}"

    Writeindex(csv, coment_01)
    if list3 == "None": Writeindex(csv, coment_02)
    else: list3.append(list2)

    Writeindex(csv, coment_A)
    Writeindex(csv, coment_B)
    Writeindex(csv, coment_C)
    Writeindex(csv, coment_D)
    Writeindex(csv, coment_E)

    return list3

if __name__ =='__main__' :
    for csv in fp_list:
        Data_list = []
        x, t = Data_set(csv) # def4

        # Machine Learning
        print(csv.split(".")[0], "processing .", end='') # Showing progress
        for a in range_a:
            print('.', end='') # Showing progress
            values = [(x, t, a, patterns[b]) for b in range_b]
            list_A = ParallelProcessing(values) # def7
            for i in list_A:
                Data_list = Write_result(i, "False", Data_list) # def8

        try:
            Write_result(Data_list) # def8
            print('Success !!') # Showing progress

        except:
            WriteError() # def2
```

Supplementary Fig. 35 | Source code for preliminary study of neural network model for predicting the HOBO of cycloheptatriene and norcaradiene.

```python
# Tsubasa Ito, Chiba Univ.

import pandas as pd
import matplotlib.pyplot as plt
from multiprocessing import get_context,Pool
import pickle, os, warnings
from sklearn.metrics import mean_absolute_error
from sklearn.model_selection import train_test_split
from sklearn.neural_network  import MLPRegressor
import lightgbm as lgb
from sklearn.linear_model import ElasticNet

##############################################################################################################
warnings.simplefilter('ignore')
path_1 = str(os.path.dirname(os.path.abspath(__file__)) + "/")
path_2 = path_1 + "Result/"
path_3 = path_1 + "csv/"
Range_01 = range(31)
##############################################################################################################

class Final_Try():

    # def01 Writing result of tuening on txt-files.
    def Writeindex(self, content, path = path_2):

        f = open(path + self.Name.split(".")[0] + "_Final" + ".txt", "a")
        f.write(str(content) + "\n")
        f.close

    # def02 Creating feature values and answer data from a data frame
    @classmethod
    def Mk_xt(cls, Pro_df):

        x = Pro_df.iloc[:,4:]
        t = Pro_df['LUBO']

        return x, t

    # def03 Sorting in ascending and descending order, referring to a specific order of elements in the list.
    @classmethod
    def sorting(cls,list_1, num, Keyword = "descending"):

        sortvalues = (lambda val : val[num])
        if Keyword == "ascending":
            list_2 = sorted(list_1, reverse=False, key=sortvalues)
        else:
            list_2 = sorted(list_1, reverse=True, key=sortvalues)

        return list_2

    # def04 calculating the average
    @classmethod
    def AVERAGE_STATUS(cls, list_1):

        Status = sum(list_1) / len(list_1)
        return Status
```

```
    # def05 Detailed Machine learning model
    def ML(self, train_x, train_t, test_x, test_t, Random_state_num):

        # Model information
        self.model.fit(train_x, train_t)
        train_score = self.model.score(train_x, train_t)
        test_score = self.model.score(test_x,test_t)
        MAE = mean_absolute_error(test_t, self.model.predict(test_x))
        Result_list = [train_score, test_score, MAE, self.model, Random_state_num]

        return Result_list

    # def06 Creating training data and labeled training data, and running machine learning
    def Final_Analysis(self, Random_state_num):

        train_val, test = train_test_split(self.df, test_size = 0.20, random_state = Random_state_num)
        train_x, train_t = self.Mk_xt(train_val) # def02
        test_x, test_t = self.Mk_xt(test) # def02
        Result_list = self.ML(train_x, train_t, test_x, test_t, Random_state_num) # def05
        Data_list = [Random_state_num, test_x, test_t, Result_list[3]]
        Result_list.append(Data_list)

        return Result_list

    # def07 Running Model tuneing processes in parallel
    def ParallelProcessing(self, values, range_01 = 30):

        p = get_context('forkserver').Pool(range_01) # Runing 30 processes in parallel.
        list_1 = p.map(self.Final_Analysis, values) # def06
        p.close()

        return list_1

    # def08  running def07
    def Main_func1(self):

        values = [a for a in Range_01]
        Result_list = self.ParallelProcessing(values) # def07
        Result_list = self.sorting(Result_list, 4, "ascending") # def03 refer to R2 element in Result_list and sort in descending order

        return Result_list

    # def09 Organizing and outputting machine learning results
    def Main_func2(self, Result_list):

        train_score_list = []
        test_score_list = []
        MAE_list = []

        for i in Result_list:
            coment1 = f'Random_state: {i[4]}'
            coment2 = f'R2 of all train datas: {i[0]}, R2 of all test datas: {i[1]}, MAE:{i[2]}'
```

```
            print(coment1,coment2)
            self.Writeindex(coment1) # def01
            self.Writeindex(coment2) # def01
            train_score_list.append(float(i[0]))
            test_score_list.append(float(i[1]))
            MAE_list.append(float(i[2]))

        train_last_status = self.AVERAGE_STATUS(train_score_list) # def04
        test_last_status  = self.AVERAGE_STATUS(test_score_list)  # def04
        MAE_last_status   = self.AVERAGE_STATUS(MAE_list)         # def04
        coment3 = f'\nThe average R2 of the all train datas: {train_last_status}\nThe average R2 of the all test datas: {test_last_status}\nMAE:{MAE_last_status}'
        print(coment3)
        self.Writeindex(coment3) # def01

        For_SAVE_List = self.sorting(Result_list, 1)[0][5] # def03

        return For_SAVE_List # Result

    # def10 Saving the best model, creating a diagram for predictive performance evaluation
    def MODEL_SAVE(self, Random_state_num, test_x, test_t, Learned_model):

     with open (self.pkl, 'wb') as f:
        pickle.dump(Learned_model, f)
        fig = plt.figure()
        plt.scatter(test_t, Learned_model.predict(test_x), alpha = 0.2, c = "blue")
        plt.plot([test_t.min(), test_t.max()], [test_t.min(), test_t.max()], c = "black")
        plt.grid()
        plt.xlabel("Real Y")
        plt.ylabel("Predicted Y")
        fig.savefig(self.pg + "_" + str(Random_state_num) + ".png")

    # def11
    def RUN(self):

        Result_list = self.Main_func1()
        For_Save_list = self.Main_func2(Result_list)
        self.MODEL_SAVE(For_Save_list[0], For_Save_list[1], For_Save_list[2], For_Save_list[3])

    # def12
    def __init__(self, model=None, BASE_NAME=None, PKL_NAME=None):

        self.model = model
        self.pg =  path_2 + BASE_NAME
        cf =  path_3 + BASE_NAME + ".csv"
        self.df = pd.read_csv(cf) # Extraxt Data frame
        self.Name = PKL_NAME
        self.pkl = path_2 + str(PKL_NAME) + ".pkl"


class Neural_Network(Final_Try):
    def __init__(self, BASE_NAME=None, PKL_NAME=None, Activation=None, hls = None):

```

```
        model = MLPRegressor(hidden_layer_sizes= hls, activation= Activation, solver= "lbfgs", alpha= 0.001, batch_size= 'auto', learning_rate= "constant",
            learning_rate_init= 0.001, power_t= 0.5, max_iter= 200, shuffle= True, random_state= 0, tol= 0.0001, verbose= False, warm_start= False, momentum= 0.9,
            nesterovs_momentum= True, early_stopping= True, validation_fraction= 0.1, beta_1= 0.9, beta_2= 0.999, epsilon= 1e-08, n_iter_no_change= 10, max_fun= 15000)
        super().__init__(model, BASE_NAME, PKL_NAME)


class LGBM(Final_Try):

    def __init__(self, BASE_NAME=None, PKL_NAME=None, Bo=None, Nu=None, Al=None, La=None, Mi=None, N_e=None):

        model = lgb.LGBMRegressor(boosting_type= Bo, num_leaves= Nu, max_depth= -1, learning_rate= 0.1, n_estimators= N_e,
                    subsample_for_bin= 200000, objective= None, class_weight= None, min_split_gain= 0.0, min_child_weight= 0.001,
                    min_child_samples= Mi, subsample= 1.0, subsample_freq=0, colsample_bytree= 1.0, reg_alpha= Al, reg_lambda= La,
                    random_state= 0, n_jobs= None, importance_type= "split")
        super().__init__(model, BASE_NAME, PKL_NAME)


if __name__ =='__main__' :

    No_1 = Neural_Network(BASE_NAME="EP_Avalon_2048", PKL_NAME="EP_NN_Avalon_2048", Activation="relu", hls=(70,25,90))
    No_1.RUN()
    No_2 = Neural_Network(BASE_NAME="EP_Avalon_4096", PKL_NAME="EP_NN_Avalon_4096", Activation="relu", hls=(70,35,90))
    No_2.RUN()
    No_3 = LGBM(BASE_NAME="EP_Avalon_2048", PKL_NAME="EP_LGBM_Avalon_2048", Bo="gbdt", Nu=14, Al=0, La=0.01, Mi=2, N_e=187)
    No_3.RUN()
    No_4 = Neural_Network(BASE_NAME="EP_Avalon_1024", PKL_NAME="EP_NN_Avalon_1024", Activation="relu", hls=(70,5,60))
    No_4.RUN()
    No_5 = Neural_Network(BASE_NAME="EP_MACCS", PKL_NAME="EP_NN_MACCS", Activation="logistic", hls=(80,5,5))
    No_5.RUN()
```

Supplementary Fig. 36 | Source code for main study of neural network model for predicting the LUBO of enophiles.

```python
# Tsubasa Ito, Chiba Univ.

import pandas as pd
import matplotlib.pyplot as plt
from multiprocessing import get_context,Pool
import pickle, os, warnings
from sklearn.metrics import mean_absolute_error
from sklearn.model_selection import train_test_split
from sklearn.neural_network  import MLPRegressor
import lightgbm as lgb
from sklearn.linear_model import ElasticNet

##############################################################################################################
warnings.simplefilter('ignore')
path_1 = str(os.path.dirname(os.path.abspath(__file__)) + "/")
path_2 = path_1 + "Result/"
path_3 = path_1 + "csv/"
Range_01 = range(31)
##############################################################################################################

class Final_Try():

    # def01 Writing result of tuening on txt-files.
    def Writeindex(self, content, path = path_2):

        f = open(path + self.Name.split(".")[0] + "_Final" + ".txt", "a")
        f.write(str(content) + "\n")
        f.close

    # def02 Creating feature values and answer data from a data frame
    @classmethod
    def Mk_xt(cls, Pro_df):

        x = Pro_df.iloc[:,4:]
        t = Pro_df['HOBO']

        return x, t

    # def03 Sorting in ascending and descending order, referring to a specific order of elements in the list.
    @classmethod
    def sorting(cls,list_1, num, Keyword = "descending"):

        sortvalues = (lambda val : val[num])
        if Keyword == "ascending":
            list_2 = sorted(list_1, reverse=False, key=sortvalues)
        else:
            list_2 = sorted(list_1, reverse=True, key=sortvalues)

        return list_2

    # def04 calculating the average
    @classmethod
    def AVERAGE_STATUS(cls, list_1):

        Status = sum(list_1) / len(list_1)
        return Status
```

```python

    # def05 Detailed Machine learning model
    def ML(self, train_x, train_t, test_x, test_t, Random_state_num):

        # Model information
        self.model.fit(train_x, train_t)
        train_score = self.model.score(train_x, train_t)
        test_score = self.model.score(test_x,test_t)
        MAE = mean_absolute_error(test_t, self.model.predict(test_x))
        Result_list = [train_score, test_score, MAE, self.model, Random_state_num]

        return Result_list

    # def06 Creating training data and labeled training data, and running machine learning
    def Final_Analysis(self, Random_state_num):

        train_val, test = train_test_split(self.df, test_size = 0.20, random_state = Random_state_num)
        train_x, train_t = self.Mk_xt(train_val) # def02
        test_x, test_t = self.Mk_xt(test) # def02
        Result_list = self.ML(train_x, train_t, test_x, test_t, Random_state_num) # def05
        Data_list = [Random_state_num, test_x, test_t, Result_list[3]]
        Result_list.append(Data_list)

        return Result_list

    # def07 Running Model tuneing processes in parallel
    def ParallelProcessing(self, values, range_01 = 30):

        p = get_context('forkserver').Pool(range_01) # Runing 30 processes in parallel.
        list_1 = p.map(self.Final_Analysis, values) # def06
        p.close()

        return list_1

    # def08  running def07
    def Main_func1(self):

        values = [a for a in Range_01]
        Result_list = self.ParallelProcessing(values) # def07
        Result_list = self.sorting(Result_list, 4, "ascending") # def03 refer to R2 element in Result_list and sort in descending order

        return Result_list

    # def09 Organizing and outputting machine learning results
    def Main_func2(self, Result_list):

        train_score_list = []
        test_score_list = []
        MAE_list = []

        for i in Result_list:
            coment1 = f'Random_state: {i[4]}'
            coment2 = f'R2 of all train datas: {i[0]}, R2 of all test datas: {i[1]}, MAE:{i[2]}'
```

```
            print(coment1,coment2)
            self.Writeindex(coment1) # def01
            self.Writeindex(coment2) # def01
            train_score_list.append(float(i[0]))
            test_score_list.append(float(i[1]))
            MAE_list.append(float(i[2]))

        train_last_status = self.AVERAGE_STATUS(train_score_list) # def04
        test_last_status  = self.AVERAGE_STATUS(test_score_list)  # def04
        MAE_last_status   = self.AVERAGE_STATUS(MAE_list)         # def04
        coment3 = f'\nThe average R2 of the all train datas: {train_last_status}\nThe average R2 of the all test datas: {test_last_status}\nMAE:{MAE_last_status}'
        print(coment3)
        self.Writeindex(coment3) # def01

        For_SAVE_List = self.sorting(Result_list, 1)[0][5] # def03

        return For_SAVE_List # Result

    # def10 Saving the best model, creating a diagram for predictive performance evaluation
    def MODEL_SAVE(self, Random_state_num, test_x, test_t, Learned_model):

     with open (self.pkl, 'wb') as f:
        pickle.dump(Learned_model, f)
        fig = plt.figure()
        plt.scatter(test_t, Learned_model.predict(test_x), alpha = 0.2, c = "blue")
        plt.plot([test_t.min(), test_t.max()], [test_t.min(), test_t.max()], c = "black")
        plt.grid()
        plt.xlabel("Real Y")
        plt.ylabel("Predicted Y")
        fig.savefig(self.pg + "_" + str(Random_state_num) + ".png")

    # def11
    def RUN(self):

        Result_list = self.Main_func1()
        For_Save_list = self.Main_func2(Result_list)
        self.MODEL_SAVE(For_Save_list[0], For_Save_list[1], For_Save_list[2], For_Save_list[3])

    # def12
    def __init__(self, model=None, BASE_NAME=None, PKL_NAME=None):

        self.model = model
        self.pg =  path_2 + BASE_NAME
        cf =  path_3 + BASE_NAME + ".csv"
        self.df = pd.read_csv(cf) # Extraxt Data frame
        self.Name = PKL_NAME
        self.pkl = path_2 + str(PKL_NAME) + ".pkl"


class Neural_Network(Final_Try):
    def __init__(self, BASE_NAME=None, PKL_NAME=None, Activation=None, hls = None):

```

```
        model = MLPRegressor(hidden_layer_sizes= hls, activation= Activation, solver= "lbfgs", alpha= 0.001, batch_size= 'auto', learning_rate= "constant",
            learning_rate_init= 0.001, power_t= 0.5, max_iter= 200, shuffle= True, random_state= 0, tol= 0.0001, verbose= False, warm_start= False, momentum= 0.9,
            nesterovs_momentum= True, early_stopping= True, validation_fraction= 0.1, beta_1= 0.9, beta_2= 0.999, epsilon= 1e-08, n_iter_no_change= 10, max_fun= 15000)
        super().__init__(model, BASE_NAME, PKL_NAME)


class LGBM(Final_Try):

    def __init__(self, BASE_NAME=None, PKL_NAME=None, Bo=None, Nu=None, Al=None, La=None, Mi=None, N_e=None):

        model = lgb.LGBMRegressor(boosting_type= Bo, num_leaves= Nu, max_depth= -1, learning_rate= 0.1, n_estimators= N_e,
                    subsample_for_bin= 200000, objective= None, class_weight= None, min_split_gain= 0.0, min_child_weight= 0.001,
                    min_child_samples= Mi, subsample= 1.0, subsample_freq=0, colsample_bytree= 1.0, reg_alpha= Al, reg_lambda= La,
                    random_state= 0, n_jobs= None, importance_type= "split")
        super().__init__(model, BASE_NAME, PKL_NAME)


class EN(Final_Try):

    def __init__(self, BASE_NAME=None, PKL_NAME=None, Al=None, L1=None):

        model = ElasticNet(max_iter = 10000, alpha = Al, l1_ratio = L1)
        super().__init__(model, BASE_NAME, PKL_NAME)


if __name__ =='__main__' :

    No_1 = Neural_Network(BASE_NAME="CHT_Avalon_1024", PKL_NAME="CHT_NN_Avalon_1024", Activation="tanh", hls=(145,30,45))
    No_1.RUN()
    No_2 = Neural_Network(BASE_NAME="CHT_Avalon_4096", PKL_NAME="CHT_NN_Avalon_4096", Activation="tanh", hls=(85,25,15))
    No_2.RUN()
    No_3 = Neural_Network(BASE_NAME="CHT_Avalon_2048", PKL_NAME="CHT_NN_Avalon_2048", Activation="tanh", hls=(125,85,75))
    No_3.RUN()
    No_4 = LGBM(BASE_NAME="CHT_Avalon_4096", PKL_NAME="CHT_LGBM_Avalon_4096", Bo="gbdt", Nu=5, Al=0.06, La=0.09, Mi=2, N_e=243)
    No_4.RUN()
    No_5 = EN(BASE_NAME="CHT_Avalon_4096", PKL_NAME="CHT_EN_Avalon_4096", Al=0.002, L1=0.33)
    No_5.RUN()
```

Supplementary Fig. 37 | Source code for main study of neural network model for predicting the HOBO of cycloheptatrienes and norcaradienes.

12. Charts of ^{1}H- and ^{13}C-NMR Spectra

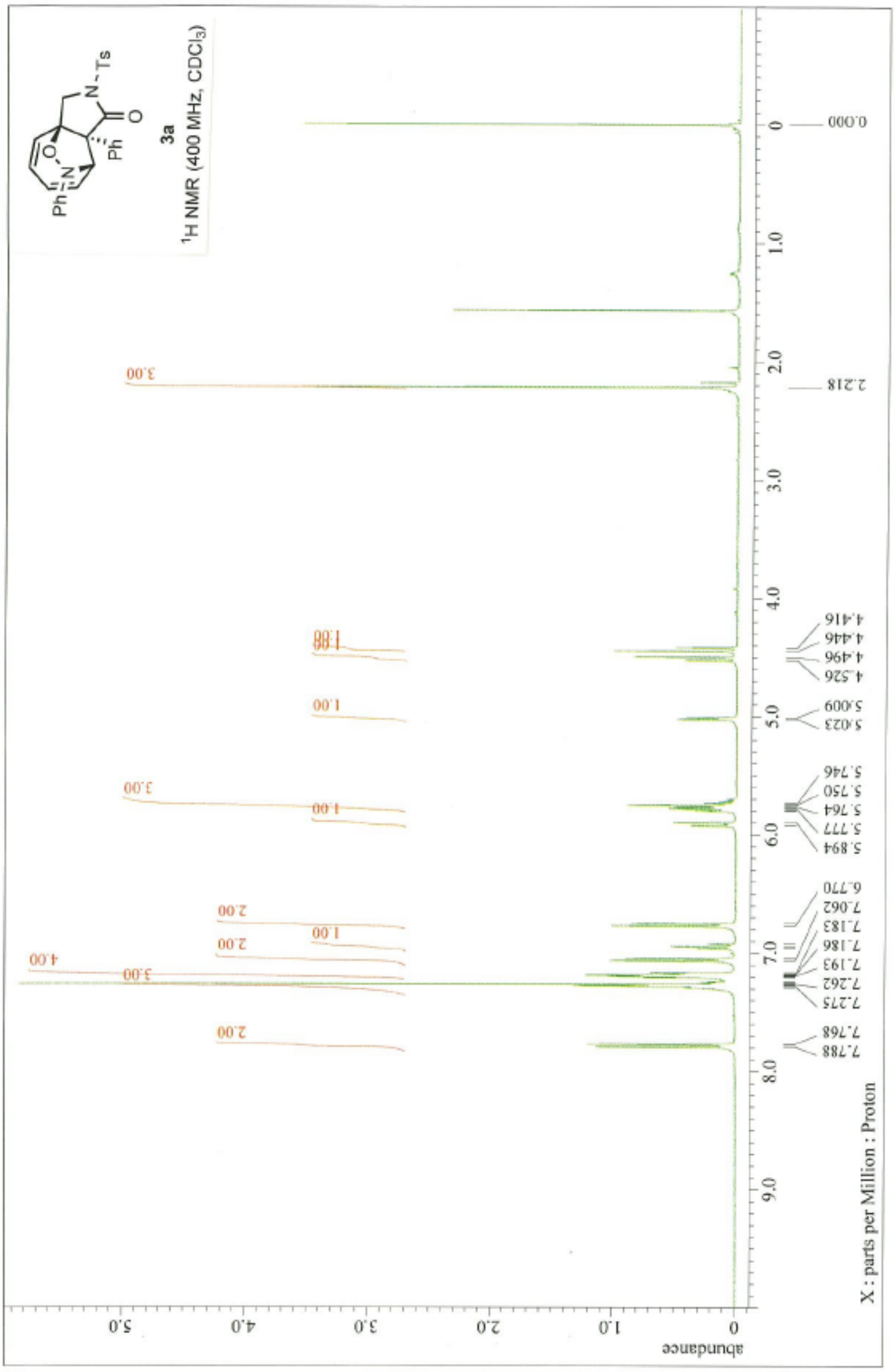

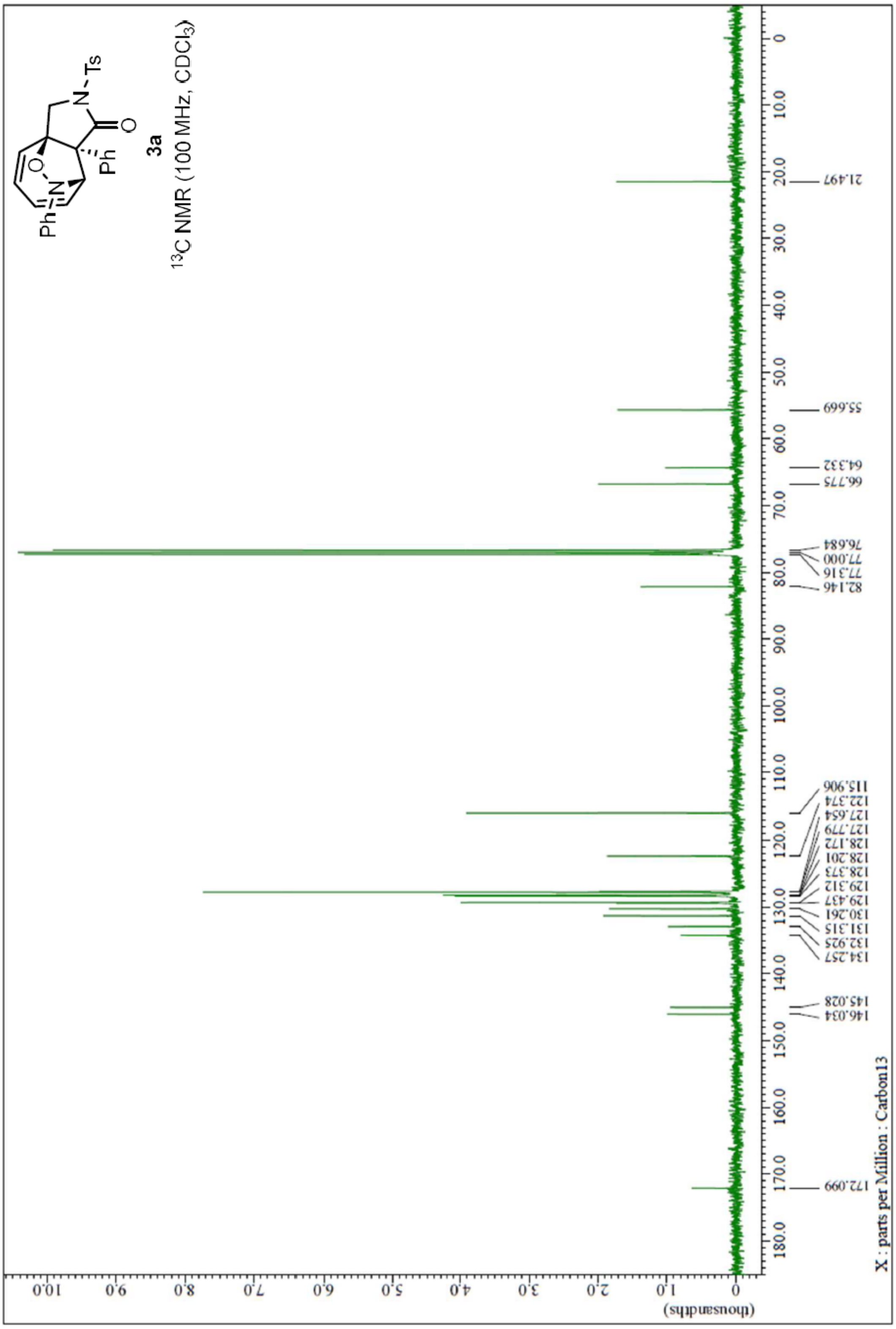

3a
13C NMR (100 MHz, CDCl3)
21.497
55.669
64.332
66.775
76.684
77.000
77.316
82.146
115.906
122.374
127.654
127.779
128.172
128.201
128.373
129.312
129.437
130.261
131.315
132.925
134.257
145.028
146.034
172.099
X : parts per Million : Carbon13
(thousandths)

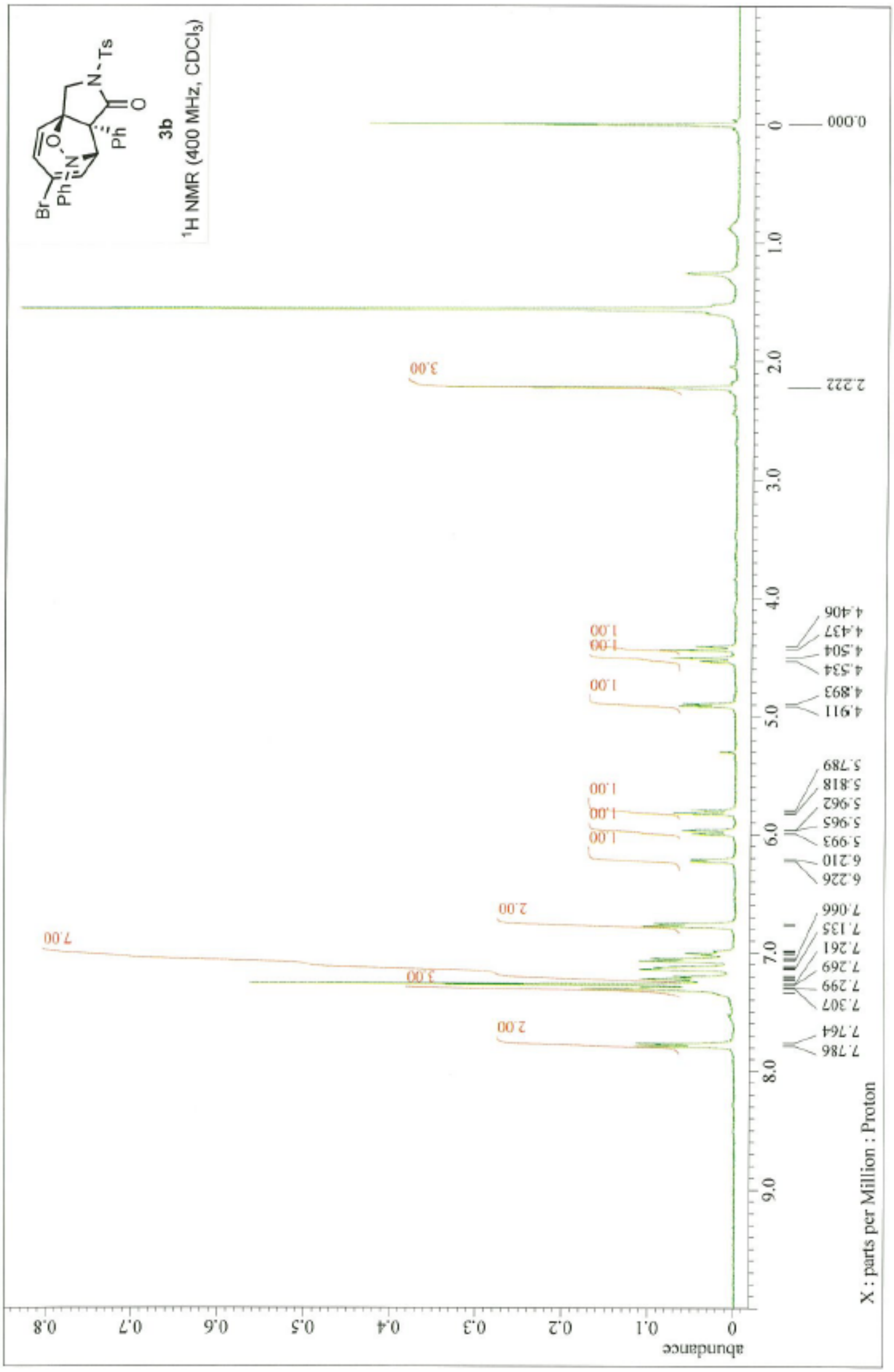
3b
1H NMR (400 MHz, CDCl3)
X : parts per Million : Proton
abundance
0.000
2.222
4.406
4.437
4.504
4.534
4.893
4.911
5.789
5.818
5.962
5.965
5.993
6.210
6.226
7.066
7.135
7.261
7.269
7.299
7.307
7.764
7.786

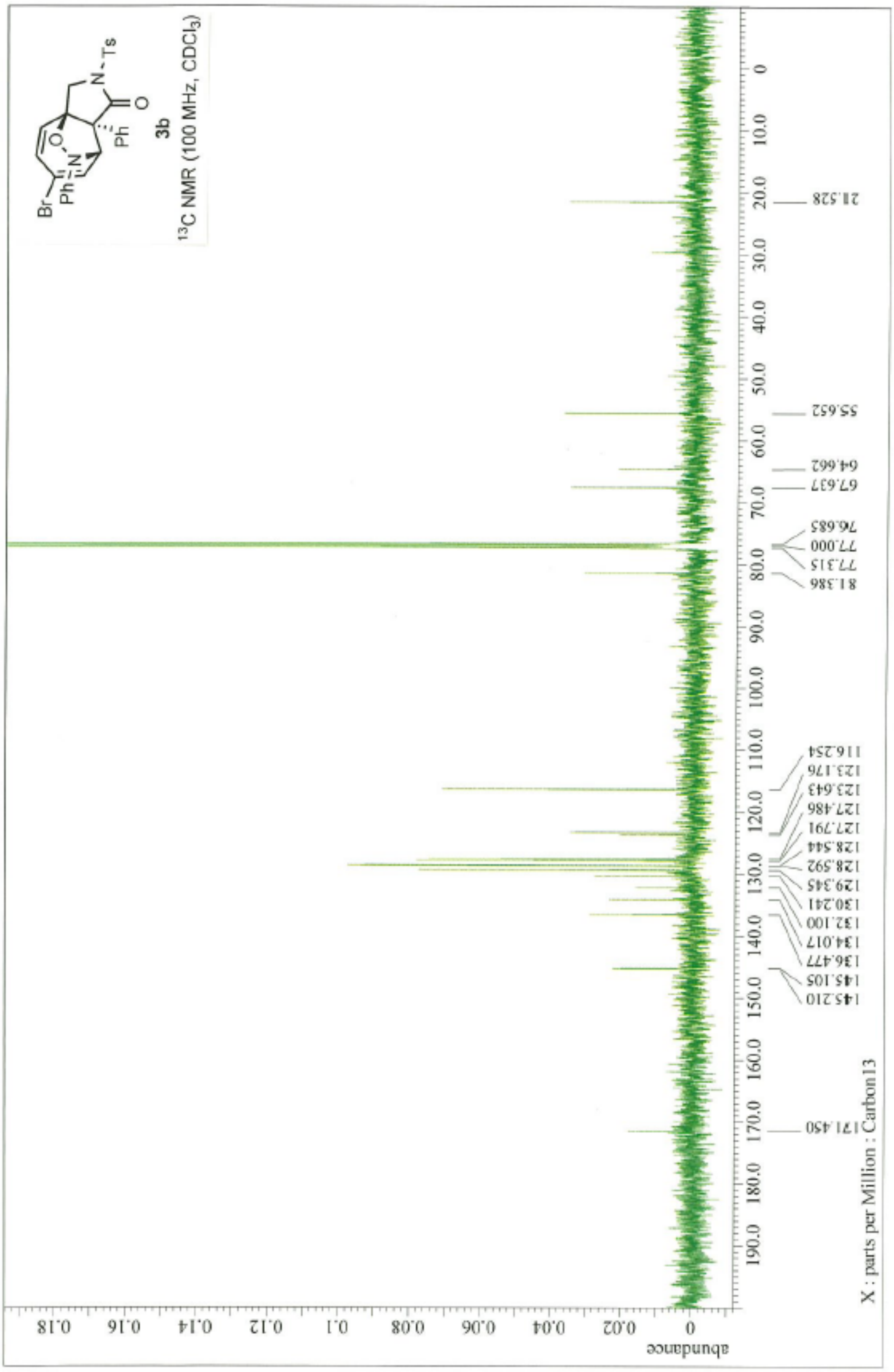
3b
13C NMR (100 MHz, CDCl3)
21.528
55.652
64.662
67.637
76.685
77.000
77.315
81.386
116.254
123.176
123.643
127.486
127.791
128.544
128.592
129.345
130.241
132.100
134.017
136.477
145.105
145.210
171.450
X : parts per Million : Carbon13
abundance

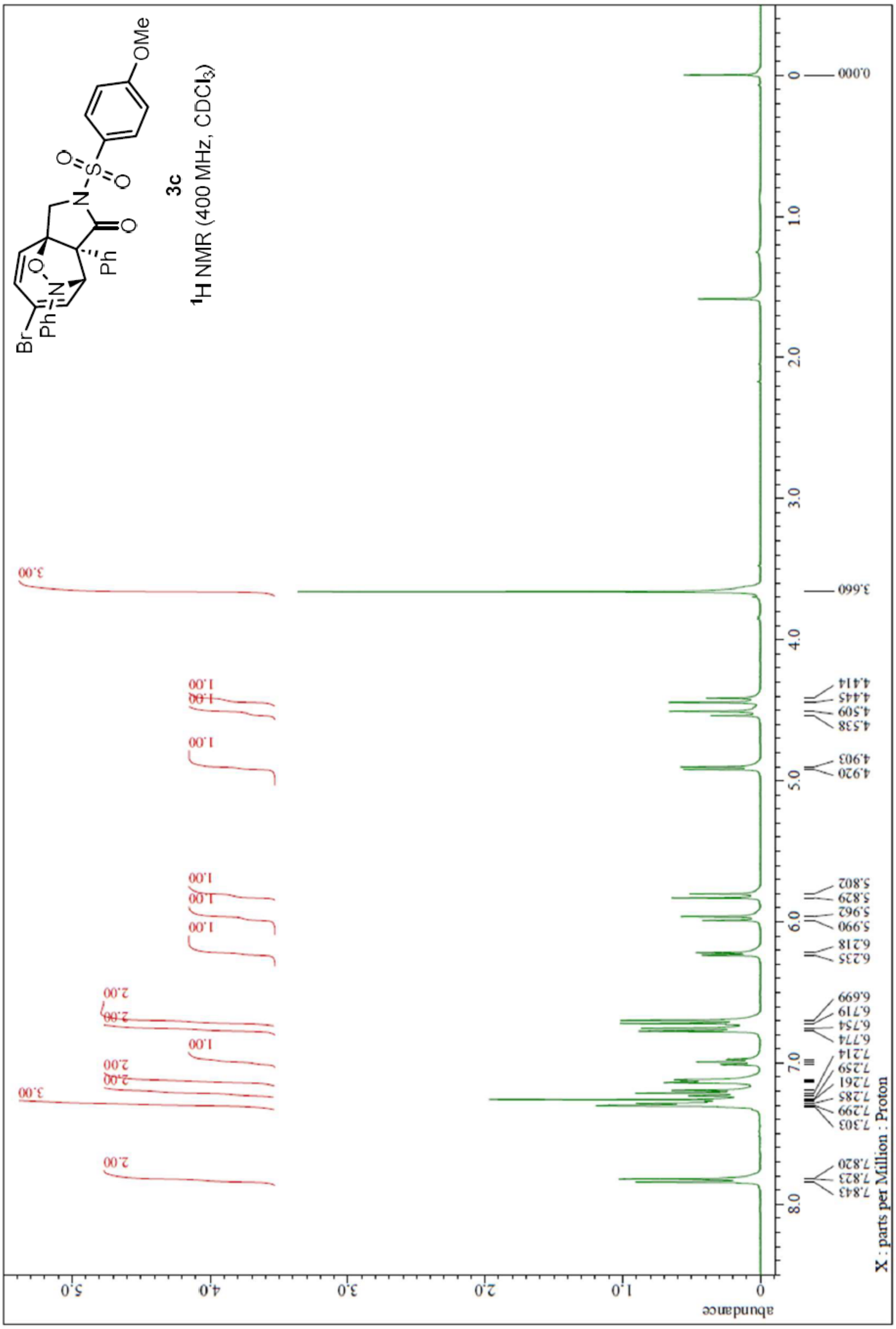

3c
1H NMR (400 MHz, CDCl3)
X : parts per Million : Proton
abundance

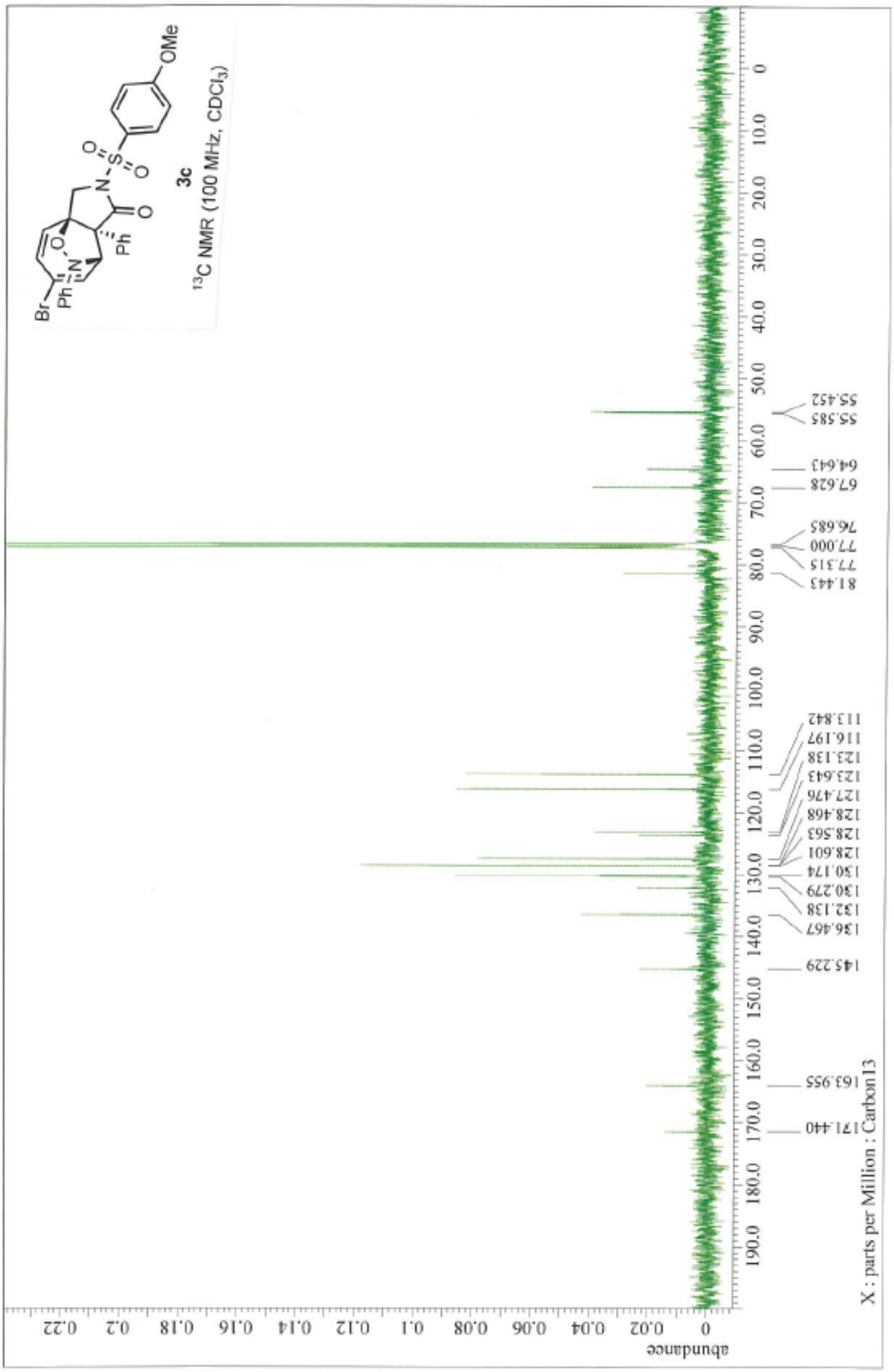
3c
13C NMR (100 MHz, CDCl3)
55.452
55.585
64.643
67.628
76.685
77.000
77.315
81.443
113.842
116.197
123.138
123.643
127.476
128.468
128.563
128.601
130.174
130.279
132.138
136.467
145.229
163.955
171.440
X : parts per Million : Carbon13
abundance

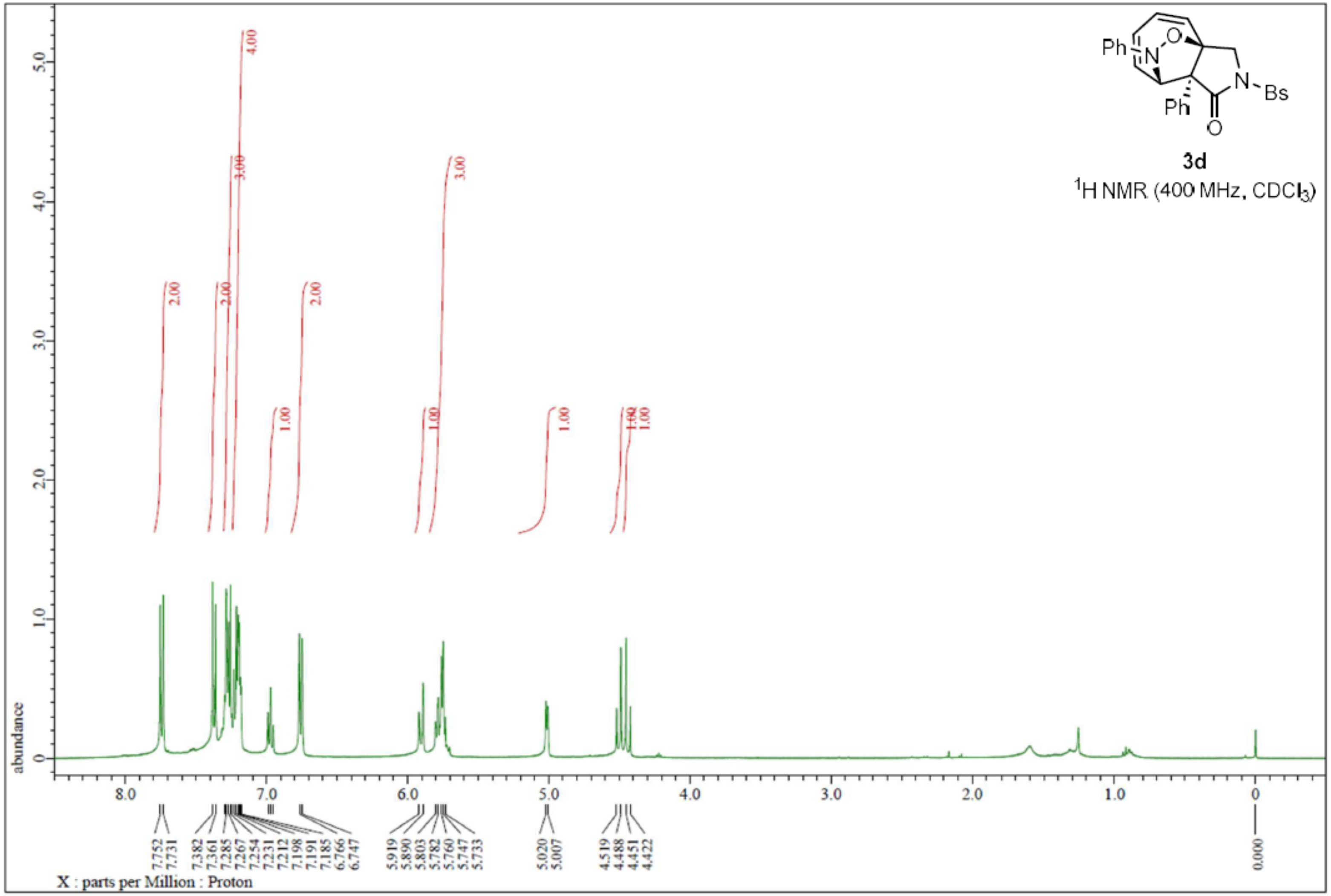
Ph
N
O
N
Bs
Ph
O
3d
^{1}H NMR (400 MHz, CDCl$_3$)
2.00
2.00
3.00
4.00
1.00
2.00
1.00
3.00
1.00
1.00
1.00
abundance
X : parts per Million : Proton
7.752
7.731
7.382
7.361
7.285
7.267
7.254
7.231
7.212
7.198
7.191
7.185
6.766
6.747
5.919
5.890
5.803
5.782
5.760
5.747
5.733
5.020
5.007
4.519
4.488
4.451
4.422
0.000

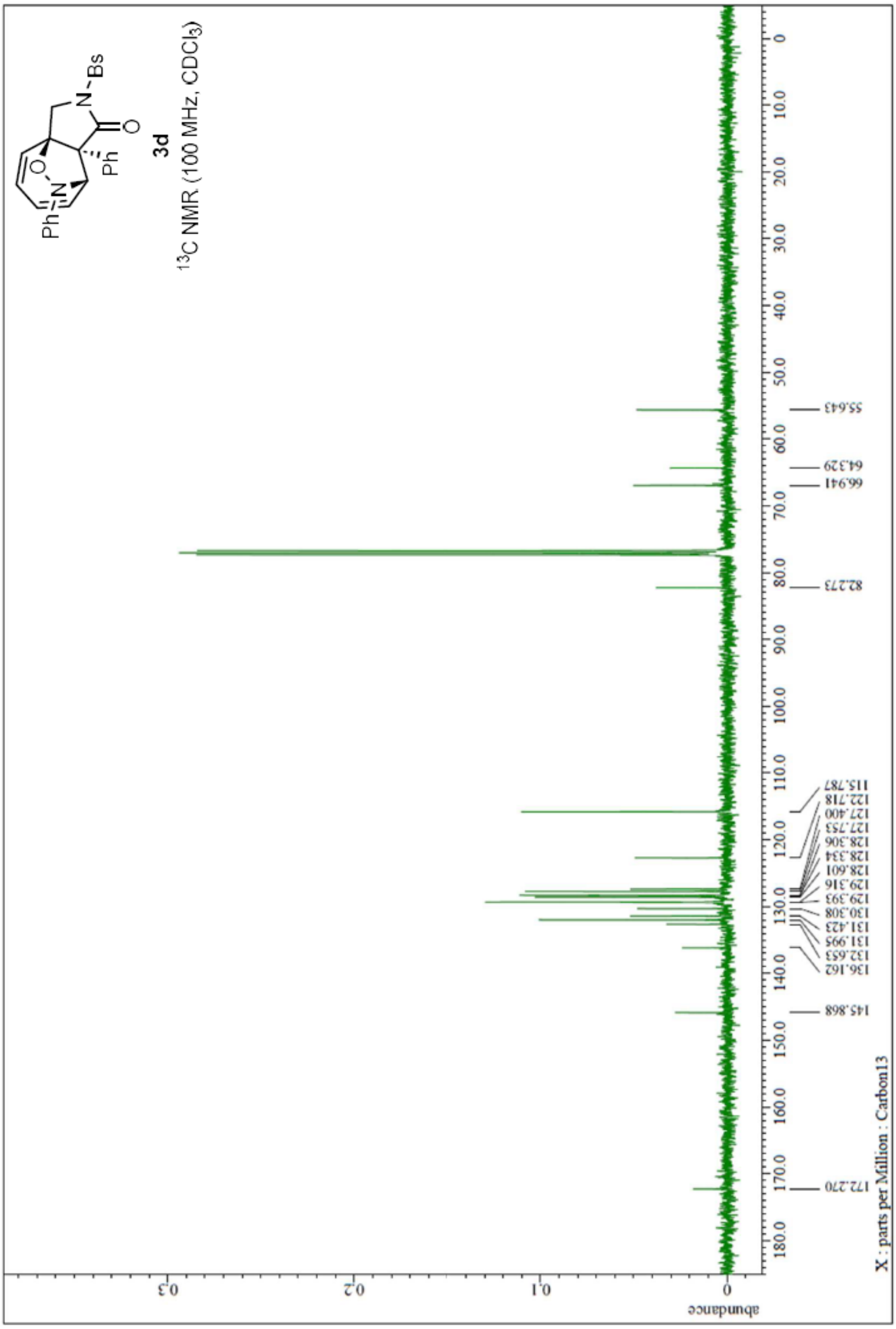
3d
13C NMR (100 MHz, CDCl3)
Ph
N
O
N
Bs
Ph
O
55.643
64.329
66.941
82.273
115.787
122.718
127.400
127.753
128.306
128.334
128.601
129.316
129.393
130.308
131.423
131.995
132.653
136.162
145.868
172.270
X : parts per Million : Carbon13
abundance

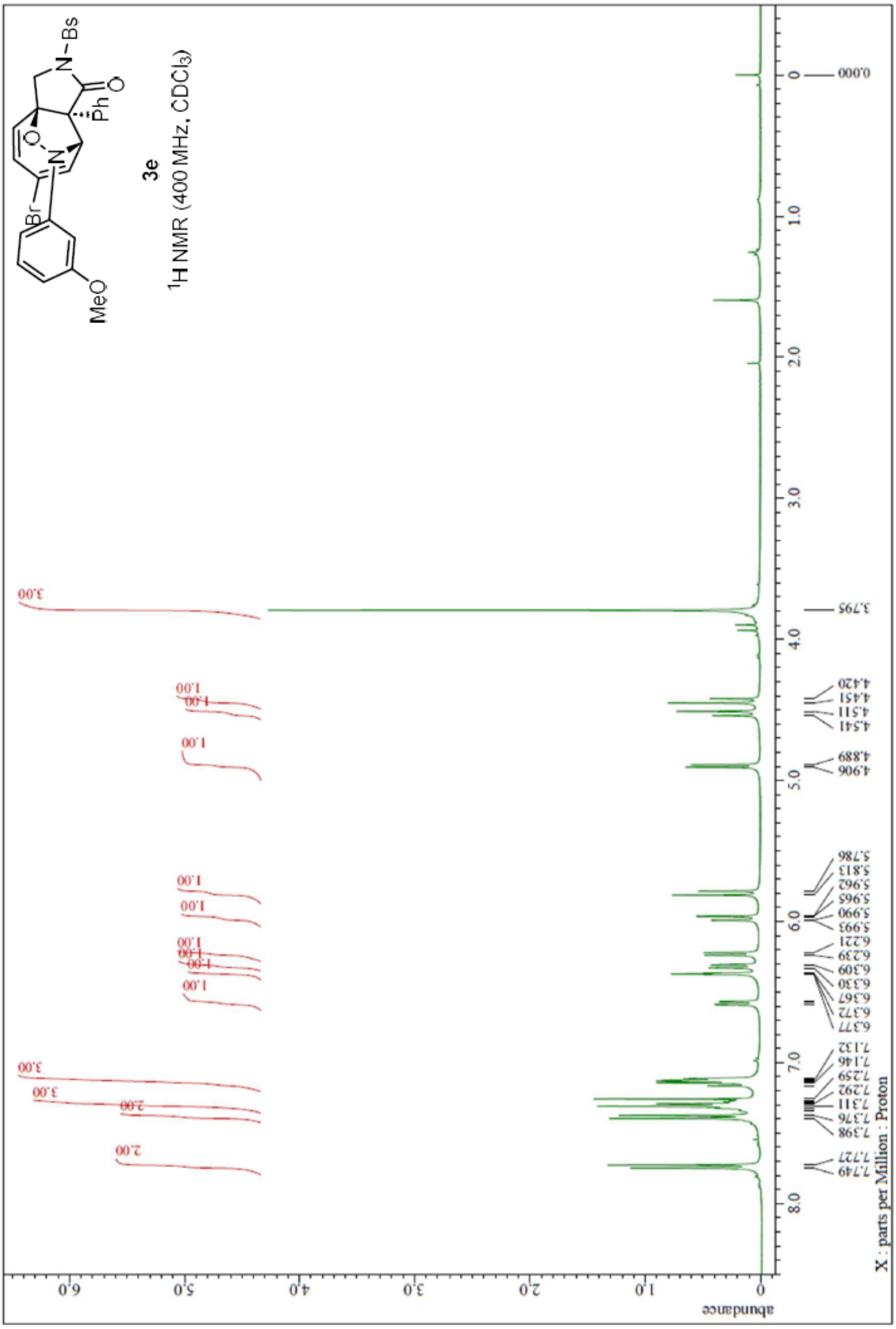

MeO
Br
N~Bs
O
N
Ph
3e
^{1}H NMR (400 MHz, CDCl$_3$)
X : parts per Million : Proton
abundance
0.000
3.795
4.420
4.451
4.511
4.541
4.889
4.906
5.786
5.813
5.962
5.965
5.990
5.993
6.221
6.239
6.309
6.330
6.367
6.372
6.377
7.132
7.146
7.259
7.292
7.311
7.376
7.398
7.727
7.749
3.00
1.00
1.00
1.00
1.00
1.00
1.00
1.00
1.00
3.00
3.00
2.00
2.00

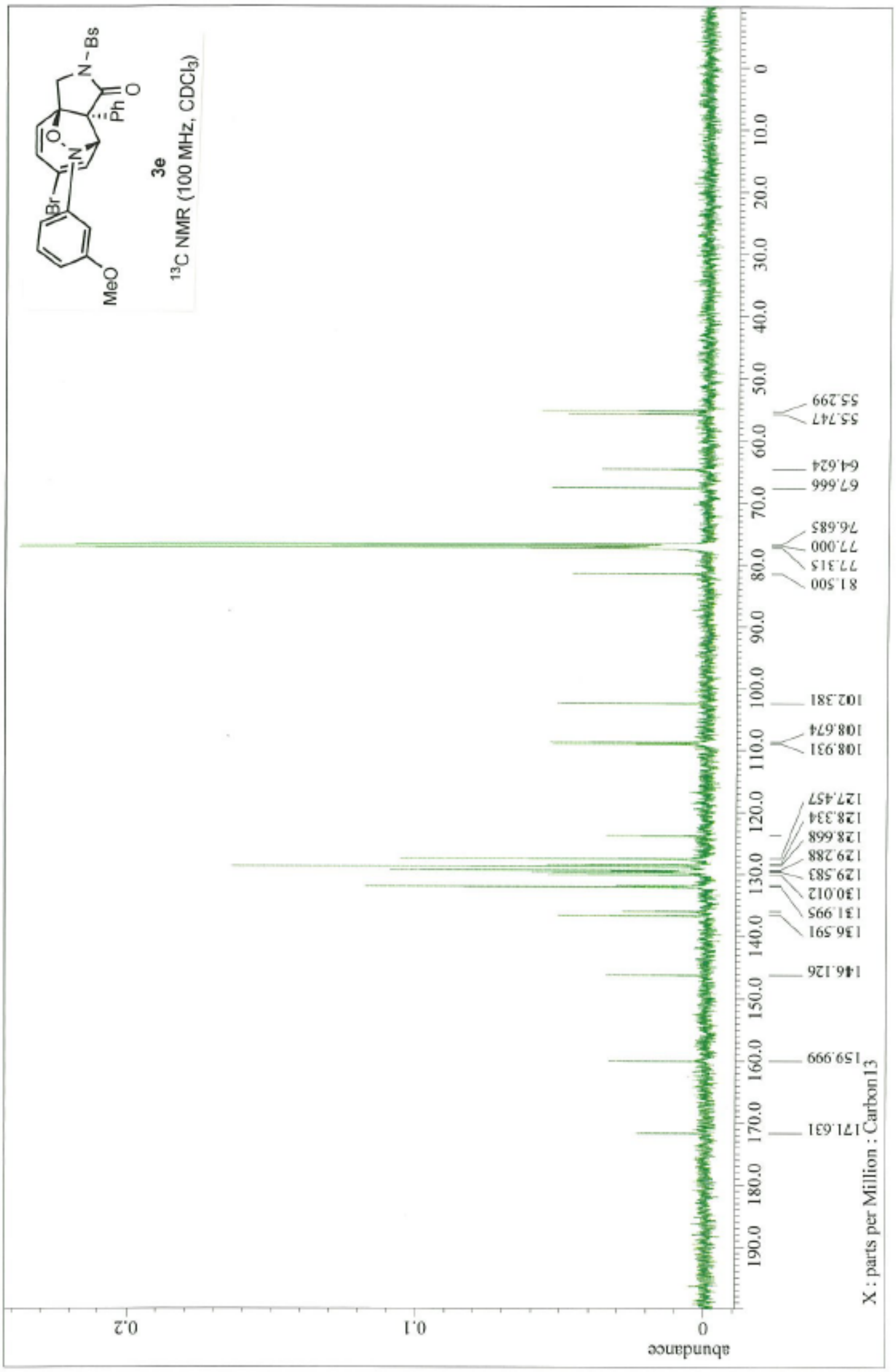
3e
13C NMR (100 MHz, CDCl3)
MeO
Br
N
O
Ph
N~Bs
O
55.299
55.747
64.624
67.666
76.685
77.000
77.315
81.500
102.381
108.674
108.931
127.457
128.334
128.668
129.288
129.583
130.012
131.995
136.591
146.126
159.999
171.631
X : parts per Million : Carbon13
abundance

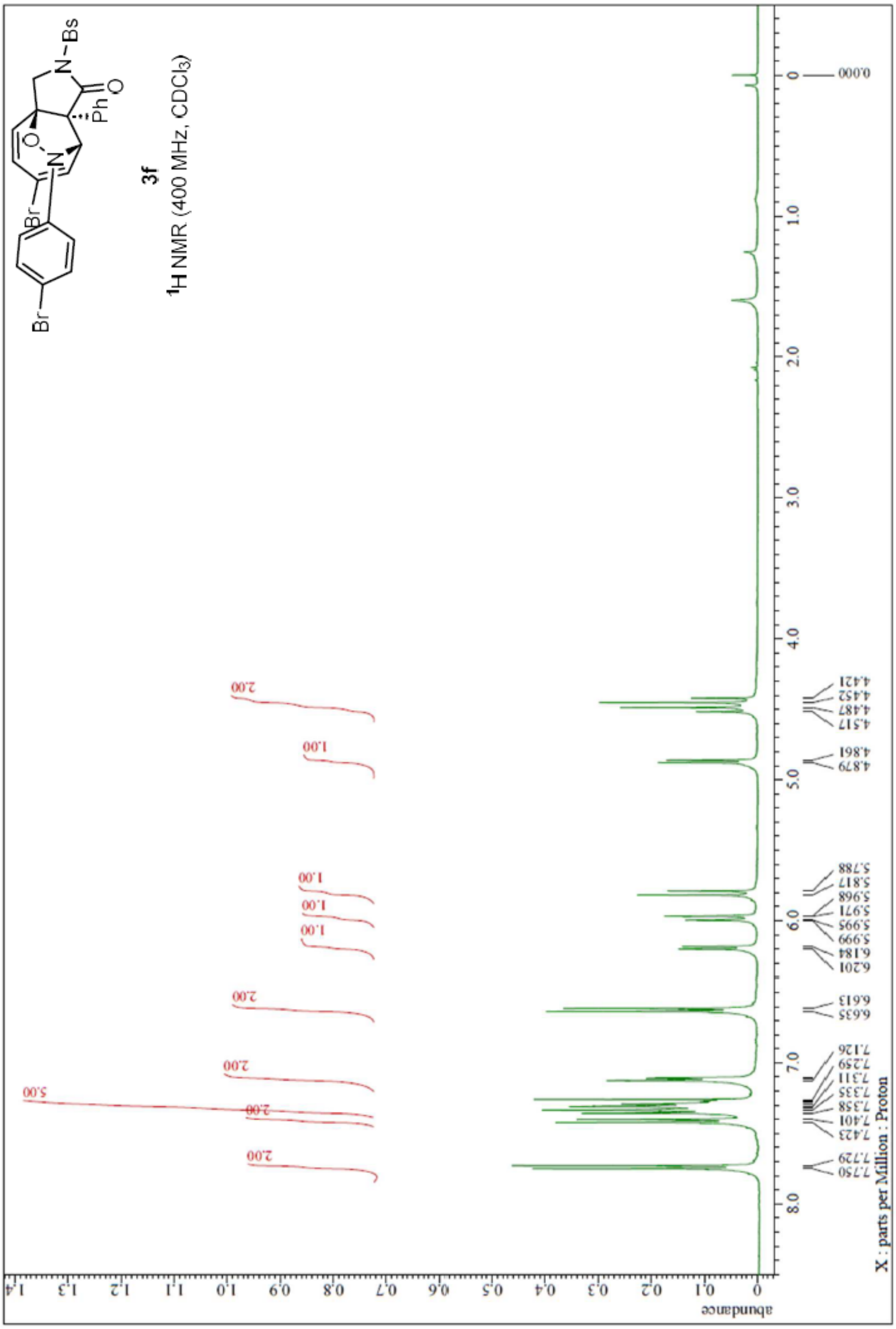
3f
1H NMR (400 MHz, CDCl3)
X : parts per Million : Proton
abundance
0.000
4.421
4.452
4.487
4.517
4.861
4.879
5.788
5.817
5.968
5.971
5.995
5.999
6.184
6.201
6.613
6.635
7.126
7.259
7.311
7.335
7.358
7.401
7.423
7.729
7.750

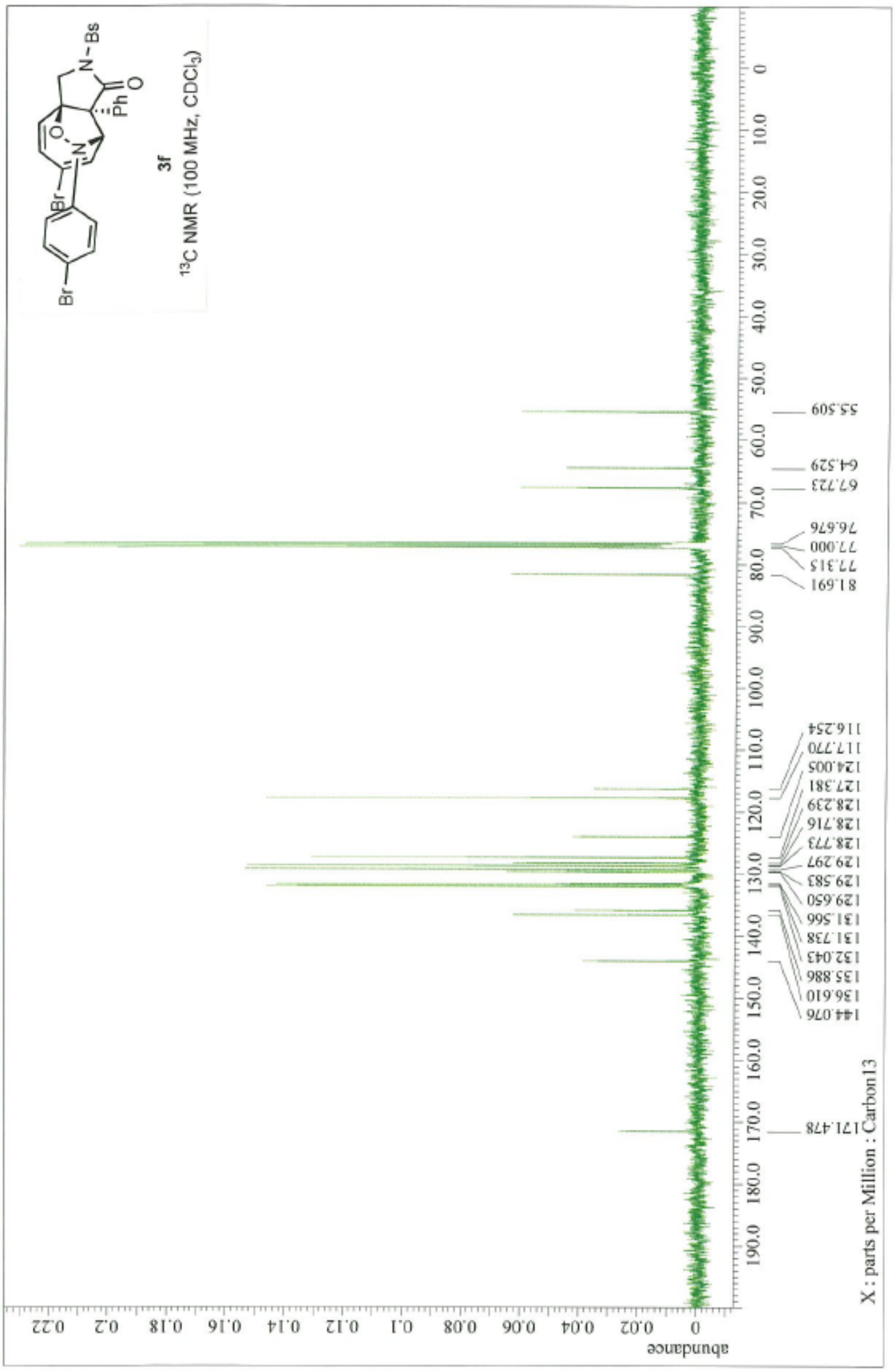
3f
13C NMR (100 MHz, CDCl3)
Br
N~Bs
Ph
O
55.509
64.529
67.723
76.676
77.000
77.315
81.691
116.254
117.770
124.005
127.381
128.239
128.716
128.773
129.297
129.583
129.650
131.566
131.738
132.043
135.886
136.610
144.076
171.478
X : parts per Million : Carbon13
abundance

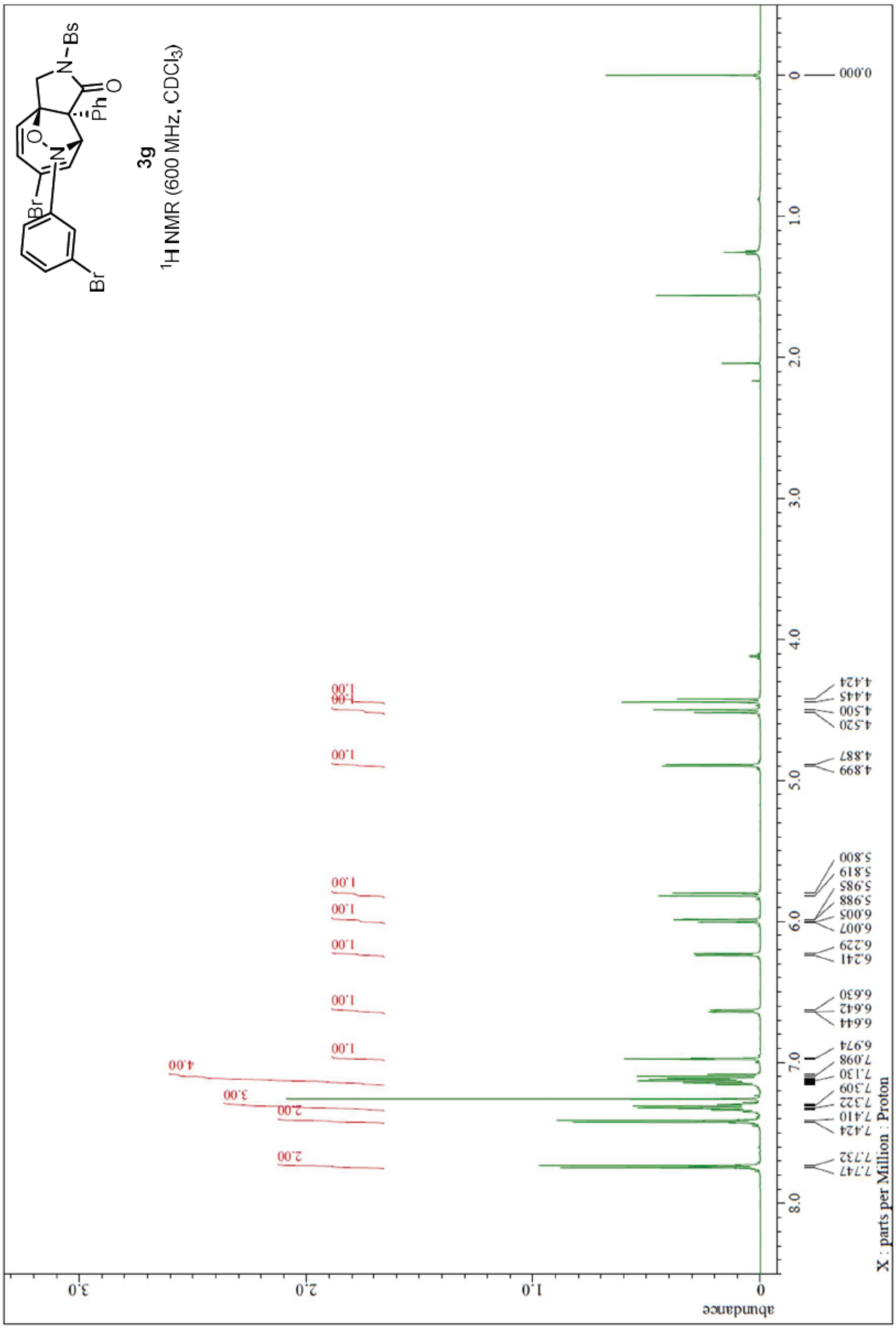

Br
Br
O
N
N–Bs
Ph
O
3g
1H NMR (600 MHz, CDCl3)
0.000
4.424
4.445
4.500
4.520
4.887
4.899
5.800
5.819
5.985
5.988
6.005
6.007
6.229
6.241
6.630
6.642
6.644
6.974
7.098
7.130
7.309
7.322
7.410
7.424
7.732
7.747
1.00
1.00
1.00
1.00
1.00
1.00
1.00
1.00
4.00
3.00
2.00
2.00
X : parts per Million : Proton
abundance

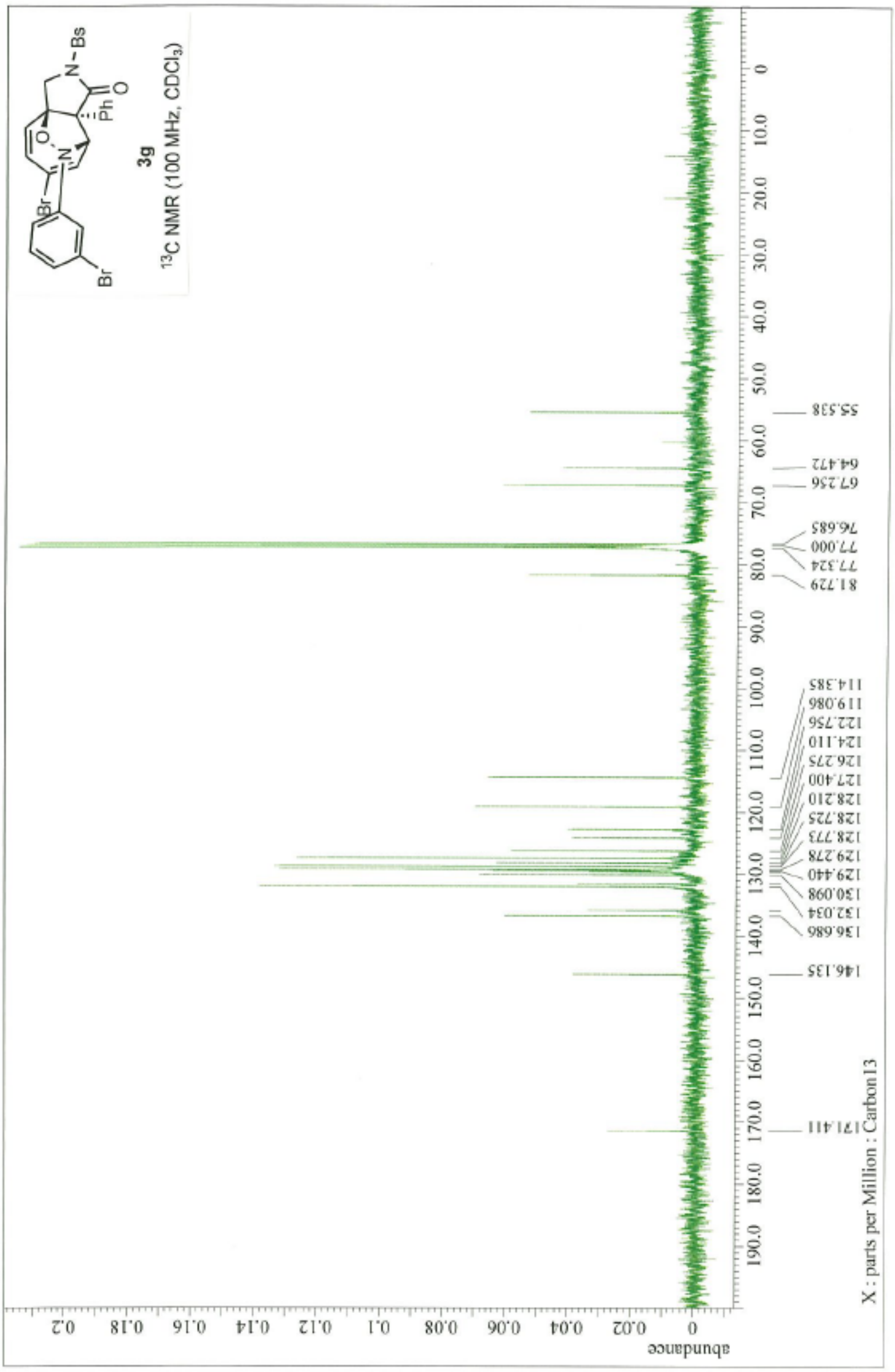
3g
13C NMR (100 MHz, CDCl3)
55.538
64.472
67.256
76.685
77.000
77.324
81.729
114.385
119.086
122.756
124.110
126.275
127.400
128.210
128.725
128.773
129.278
129.440
130.098
132.034
136.686
146.135
171.411
X : parts per Million : Carbon13
abundance

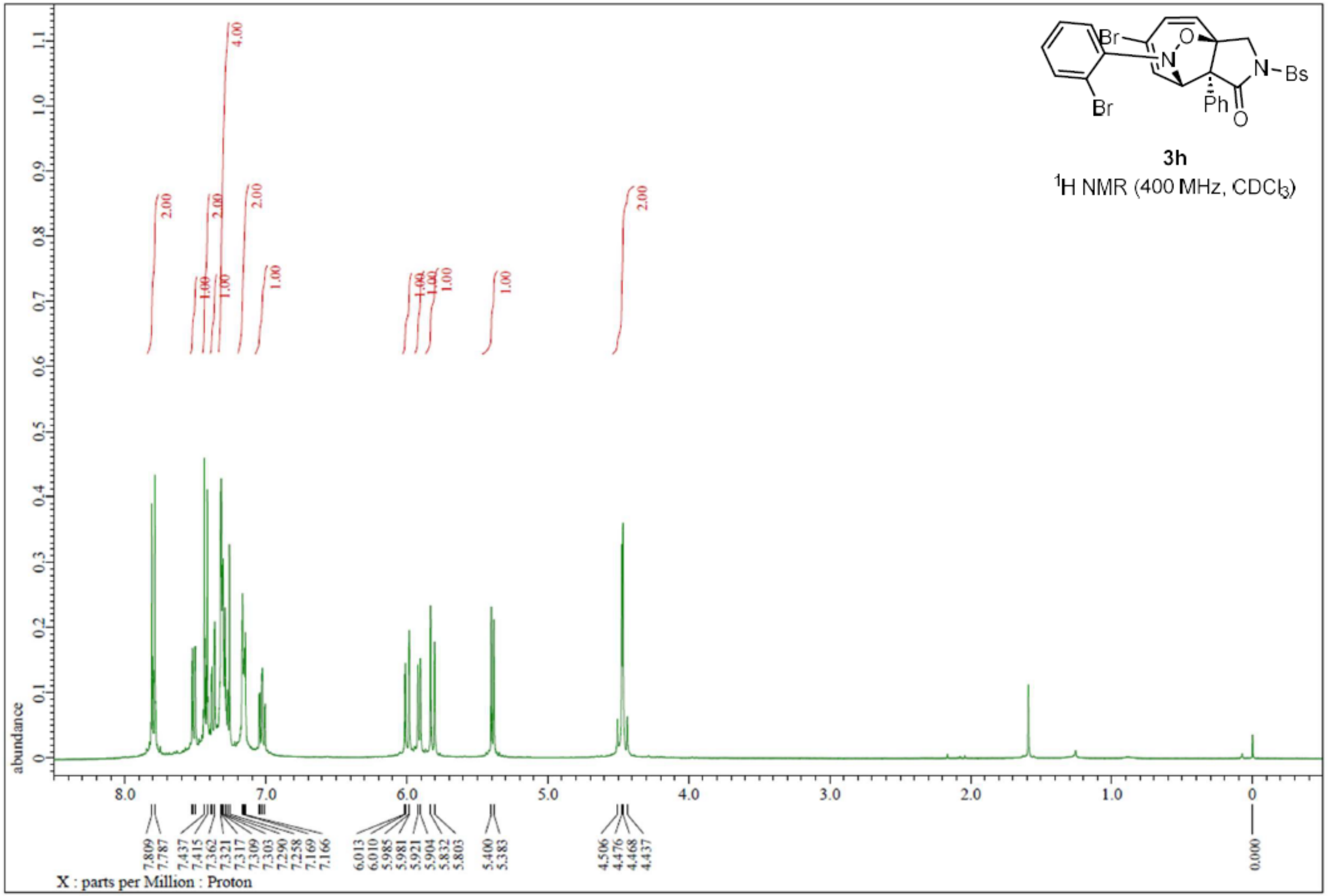

3h

^1H NMR (400 MHz, $CDCl_3$)

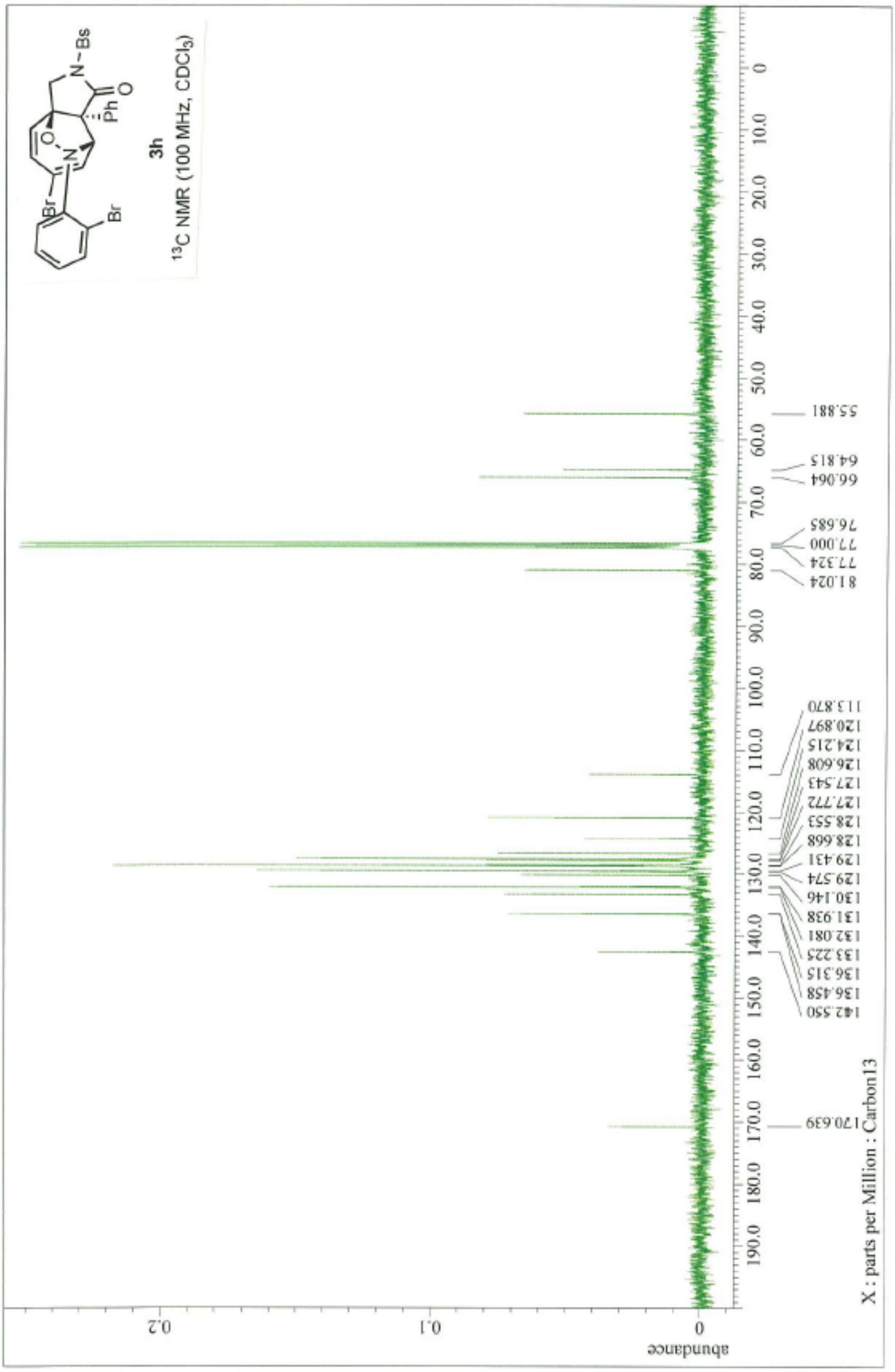
N-Bs
Br
O
Ph
3h
13C NMR (100 MHz, CDCl3)
55.881
64.815
66.064
76.685
77.000
77.324
81.024
113.870
120.897
124.215
126.608
127.543
127.772
128.553
128.668
129.431
129.574
130.146
131.938
132.081
133.225
136.315
136.458
142.550
170.639
X : parts per Million : Carbon13
abundance

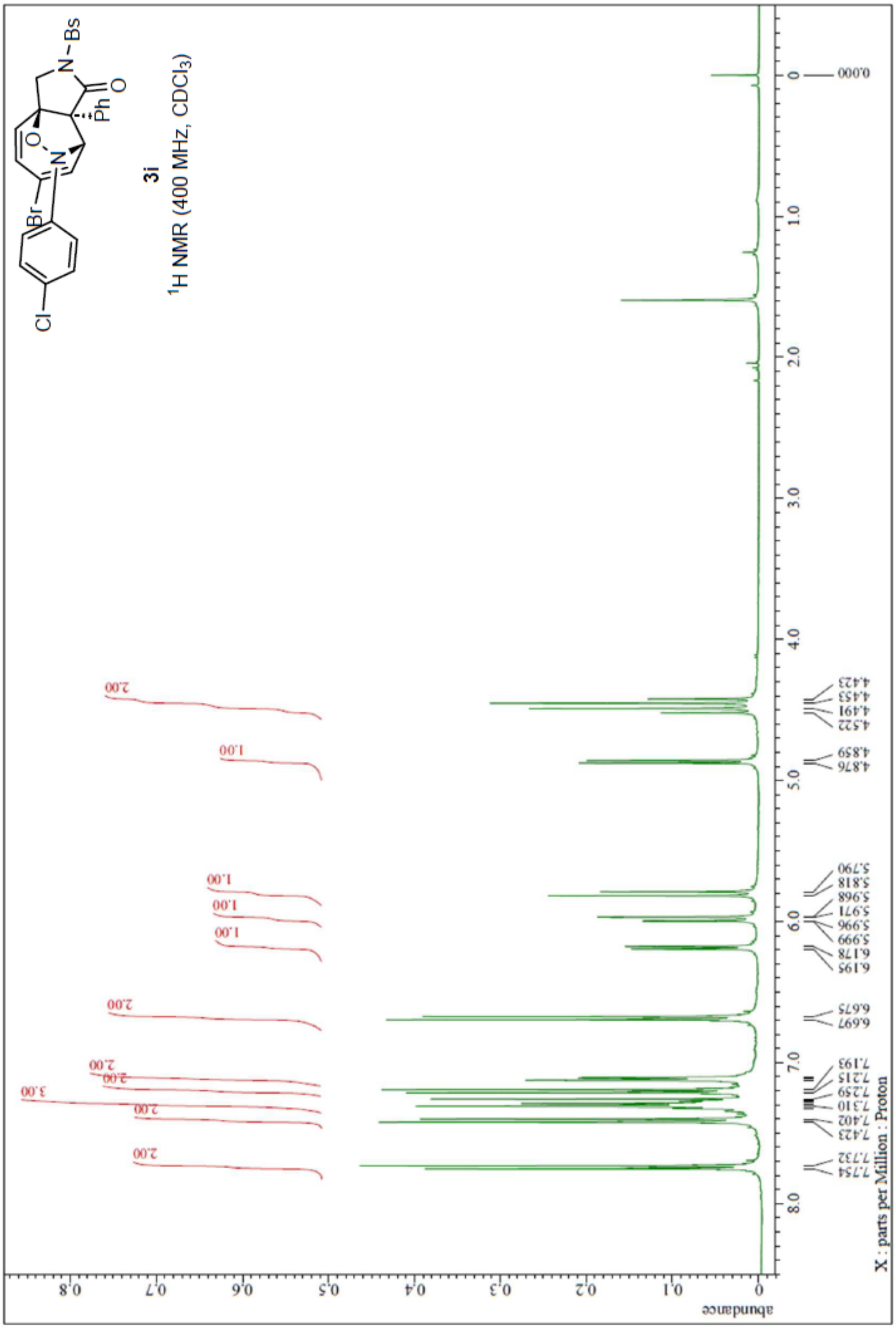
3i
^{1}H NMR (400 MHz, CDCl$_3$)
Cl
Br
N–Bs
O
Ph
X : parts per Million : Proton
abundance

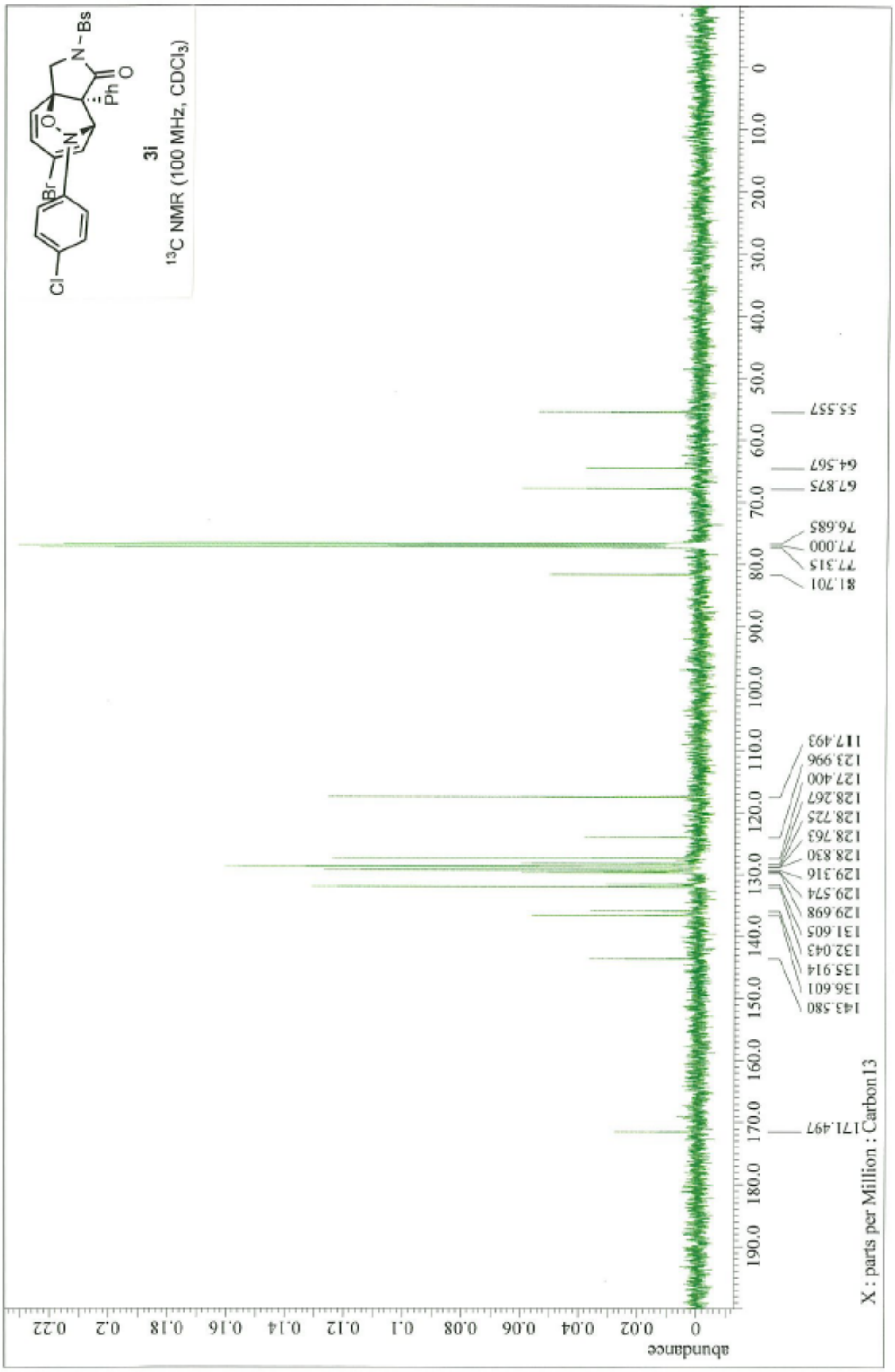

3i
13C NMR (100 MHz, CDCl3)
55.557
64.567
67.875
76.685
77.000
77.315
81.701
117.493
123.996
127.400
128.267
128.725
128.763
128.830
129.316
129.574
129.698
131.605
132.043
135.914
136.601
143.580
171.497
X : parts per Million : Carbon13
abundance

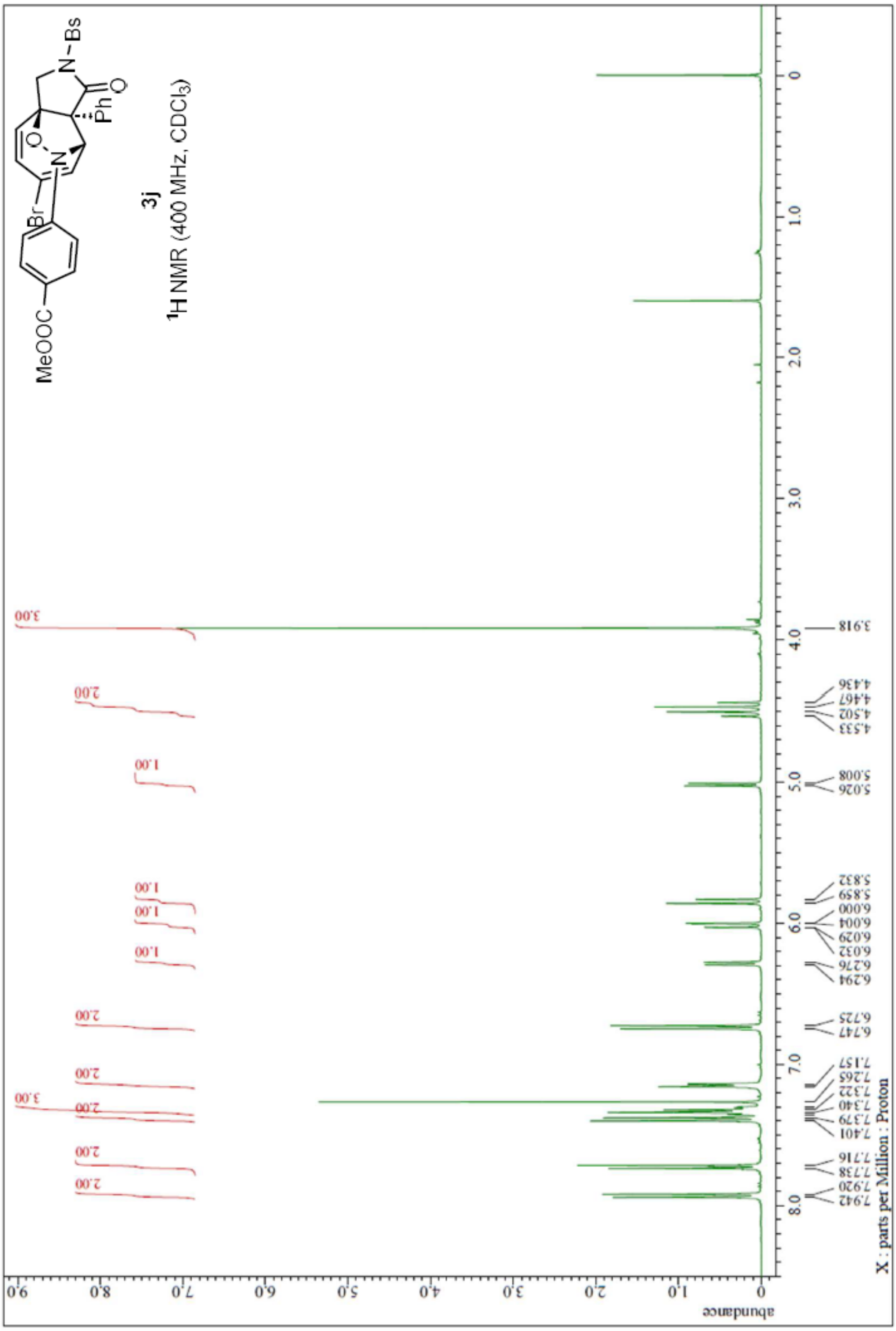
MeOOC
Br
N
O
N–Bs
Ph
O
3j
1H NMR (400 MHz, CDCl3)
3.918
4.436
4.467
4.502
4.533
5.008
5.026
5.832
5.859
6.000
6.004
6.029
6.032
6.276
6.294
6.725
6.747
7.157
7.265
7.322
7.340
7.379
7.401
7.716
7.738
7.920
7.942
X : parts per Million : Proton
abundance

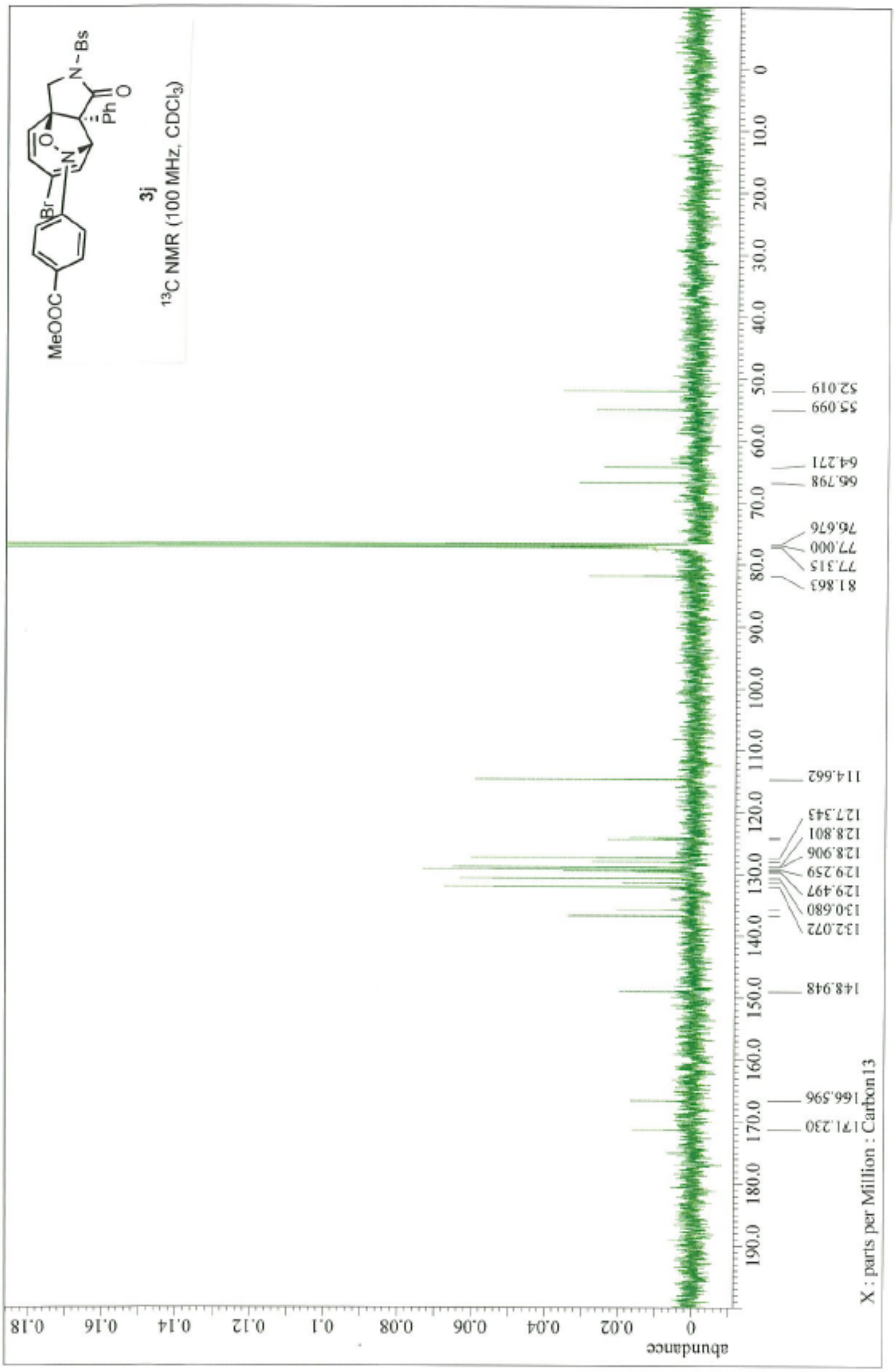
MeOOC
Br
N–Bs
O
N
Ph
3j
13C NMR (100 MHz, CDCl3)
52.019
55.099
64.271
66.798
76.676
77.000
77.315
81.863
114.662
127.343
128.801
128.906
129.259
129.497
130.680
132.072
148.948
166.596
171.230
X : parts per Million : Carbon13
abundance

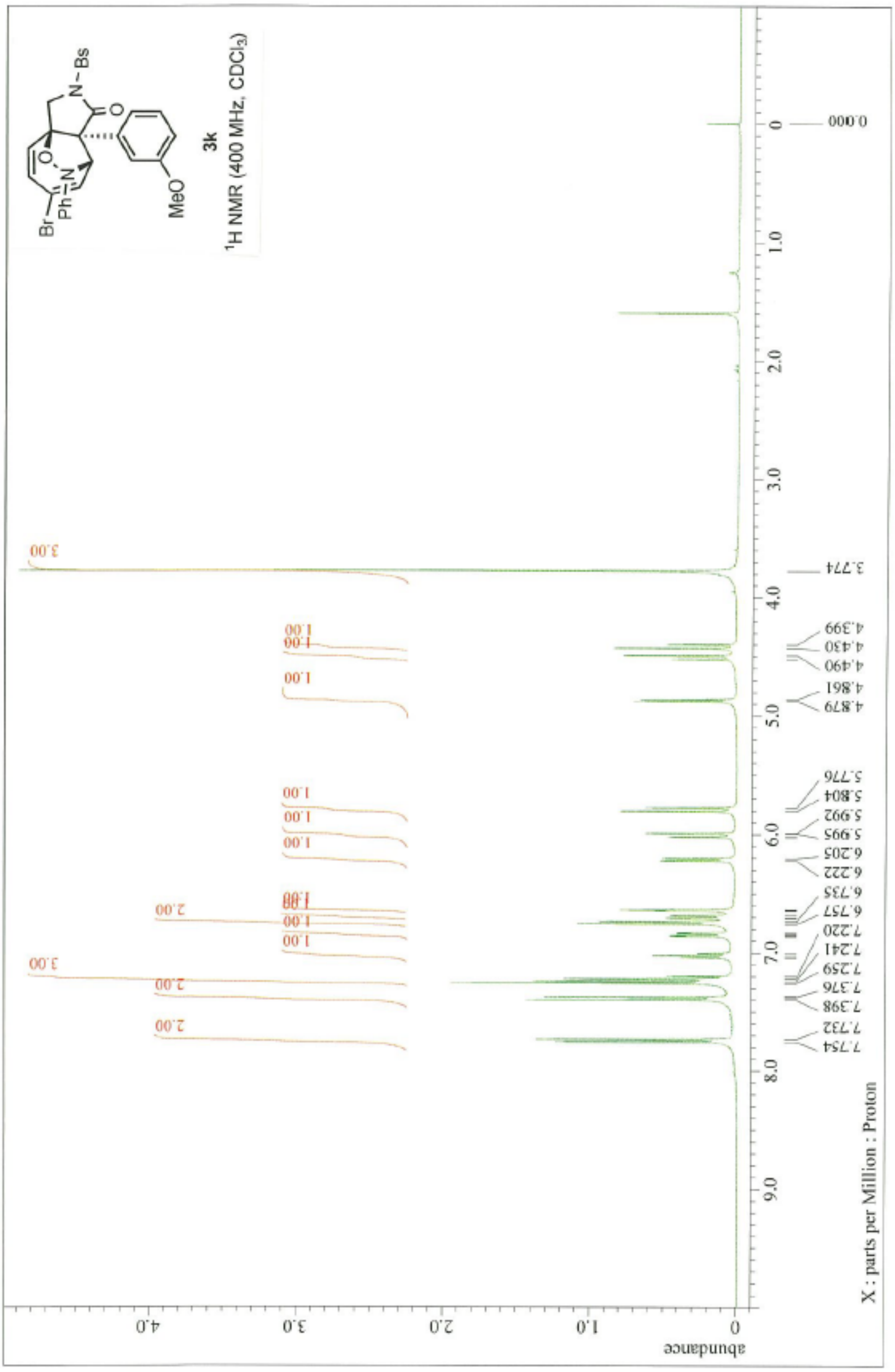

3k
1H NMR (400 MHz, CDCl3)
0.000
3.774
4.399
4.430
4.490
4.861
4.879
5.776
5.804
5.992
5.995
6.205
6.222
6.735
6.757
7.220
7.241
7.259
7.376
7.398
7.732
7.754
X : parts per Million : Proton
abundance

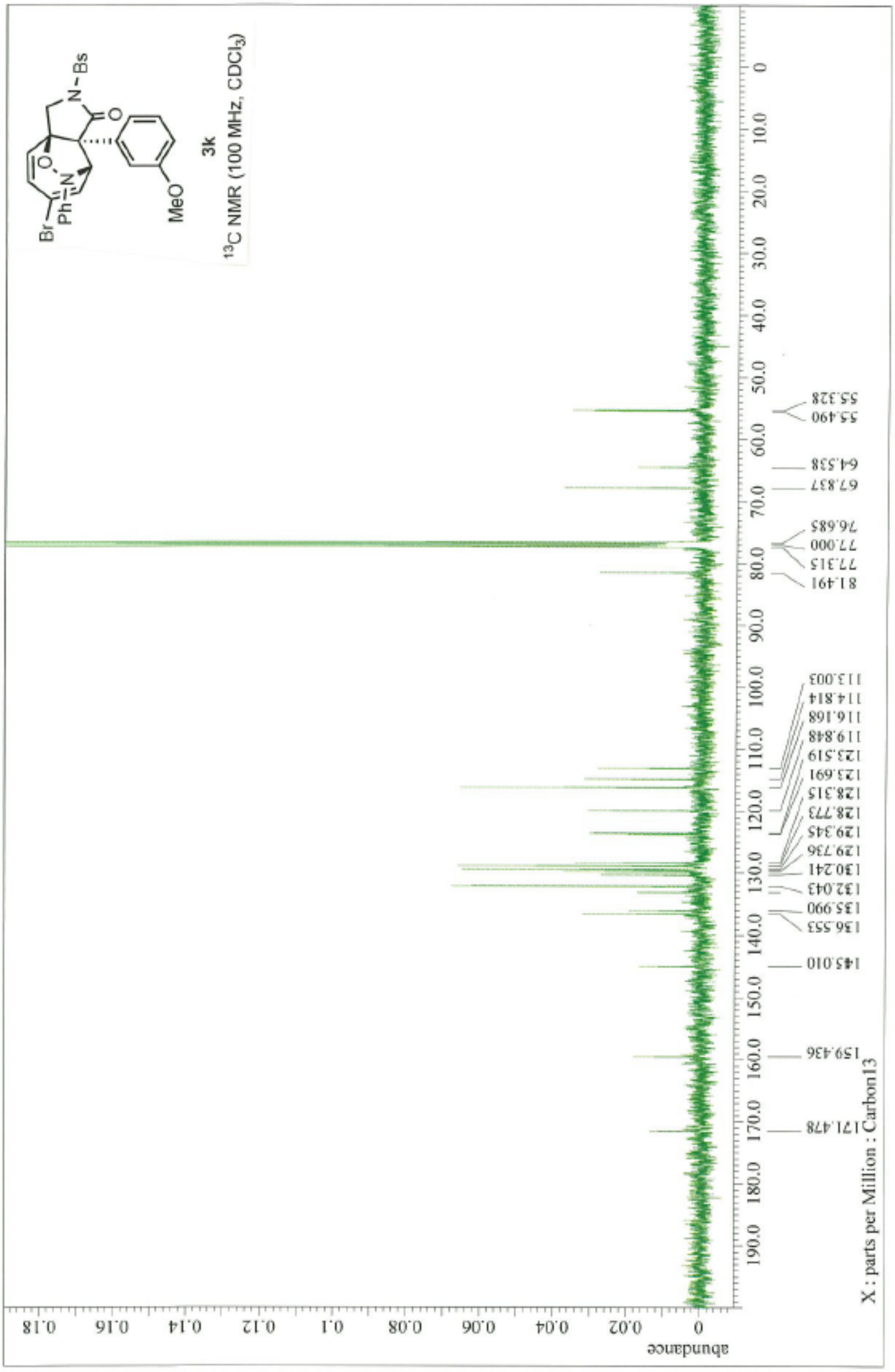

Br
Ph
N
O
N−Bs
O
MeO
3k
13C NMR (100 MHz, CDCl3)
55.328
55.490
64.538
67.837
76.685
77.000
77.315
81.491
113.003
114.814
116.168
119.848
123.519
123.691
128.315
128.773
129.345
129.736
130.241
132.043
135.990
136.553
145.010
159.436
171.478
X : parts per Million : Carbon13
abundance

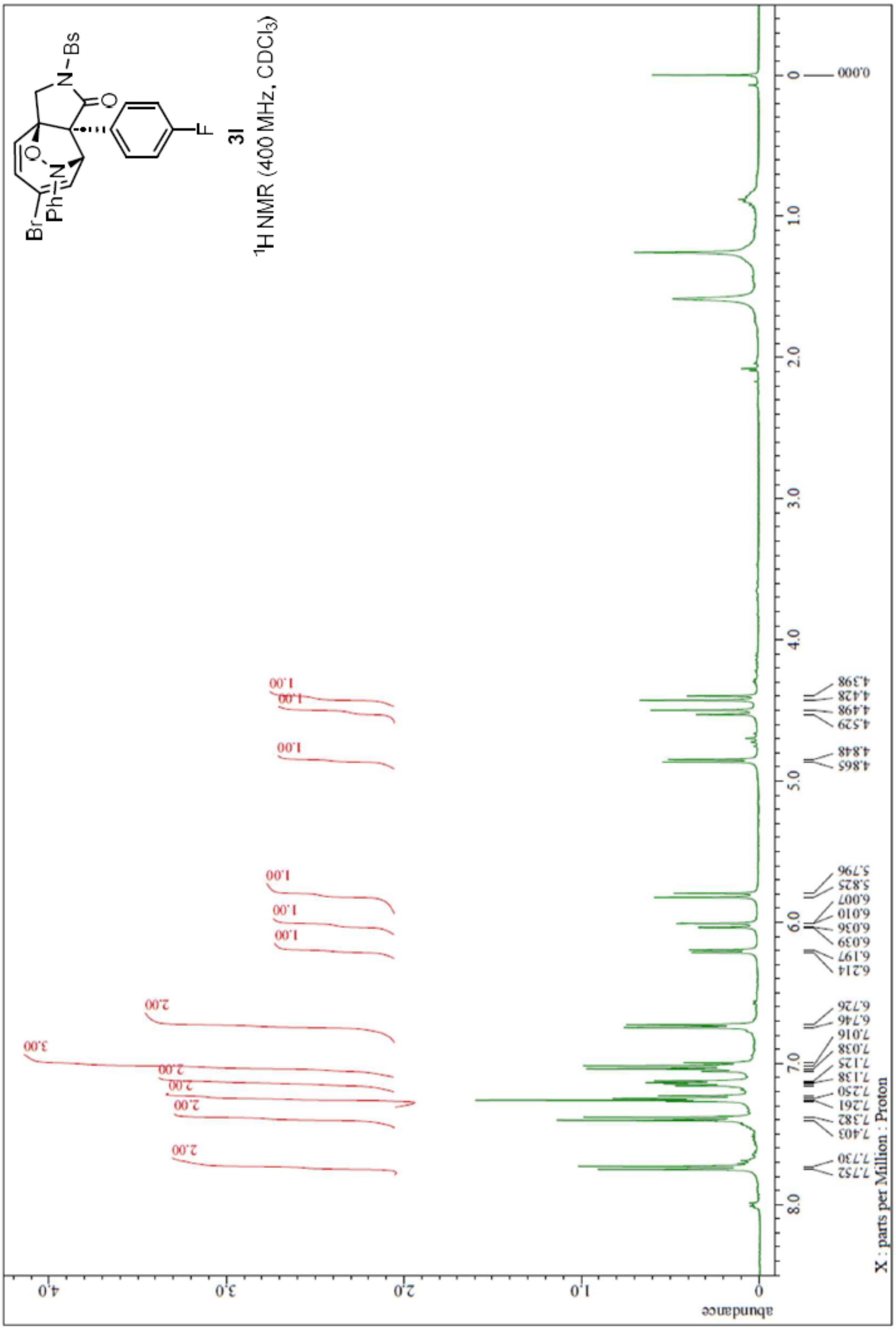
Br
Ph
N
O
N–Bs
O
F
3l
¹H NMR (400 MHz, CDCl3)
0.000
4.398
4.428
4.498
4.529
4.848
4.865
5.796
5.825
6.007
6.010
6.036
6.039
6.197
6.214
6.726
6.746
7.016
7.038
7.125
7.138
7.250
7.261
7.382
7.403
7.730
7.752
X : parts per Million : Proton
abundance

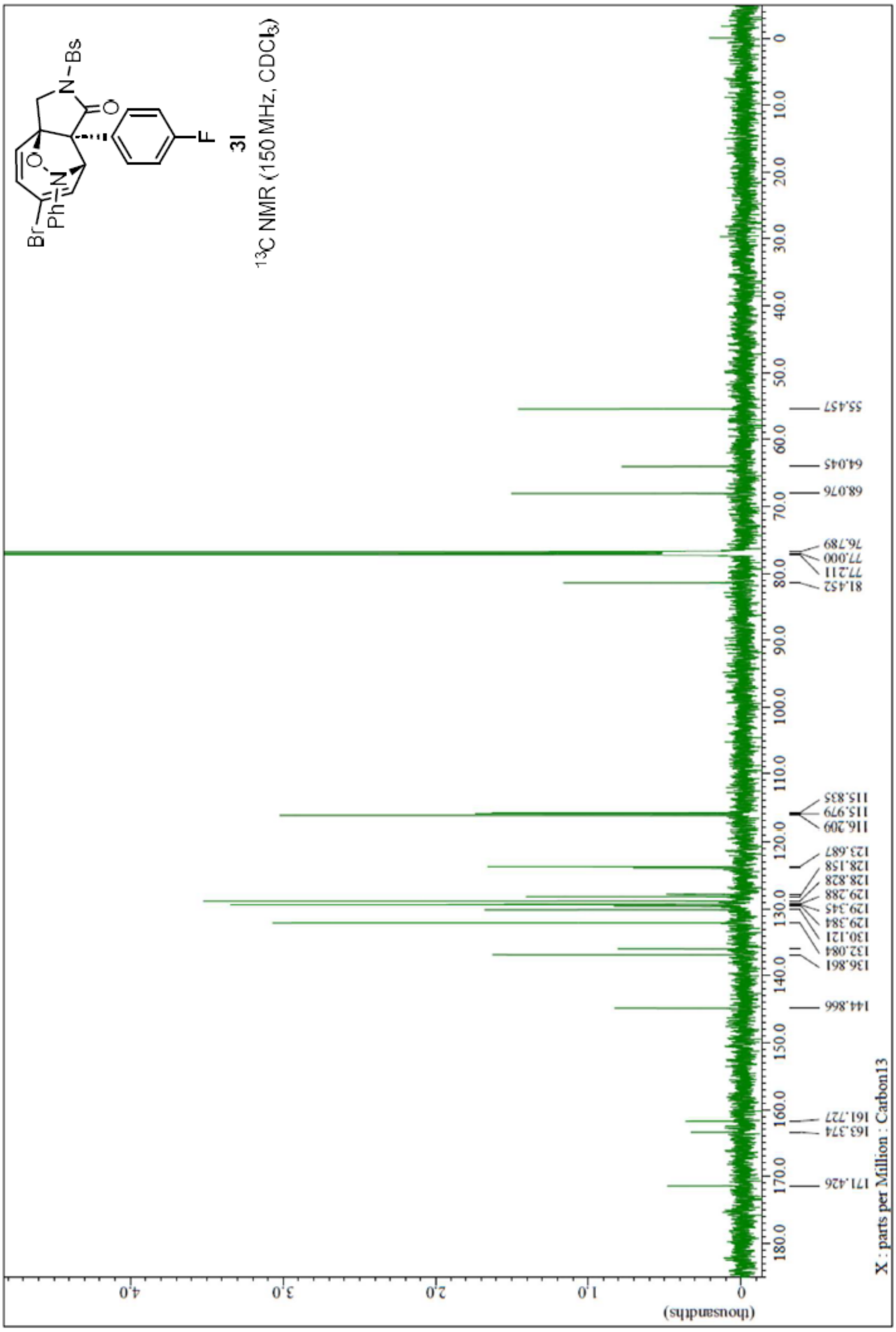

Br
Ph
N
O
N–Bs
O
F
3l
13C NMR (150 MHz, CDCl3)
55.457
64.045
68.076
76.789
77.000
77.211
81.452
115.835
115.979
116.209
123.687
128.158
128.828
129.288
129.345
129.384
130.121
132.084
136.861
144.866
161.727
163.374
171.426
X : parts per Million : Carbon13
(thousandths)

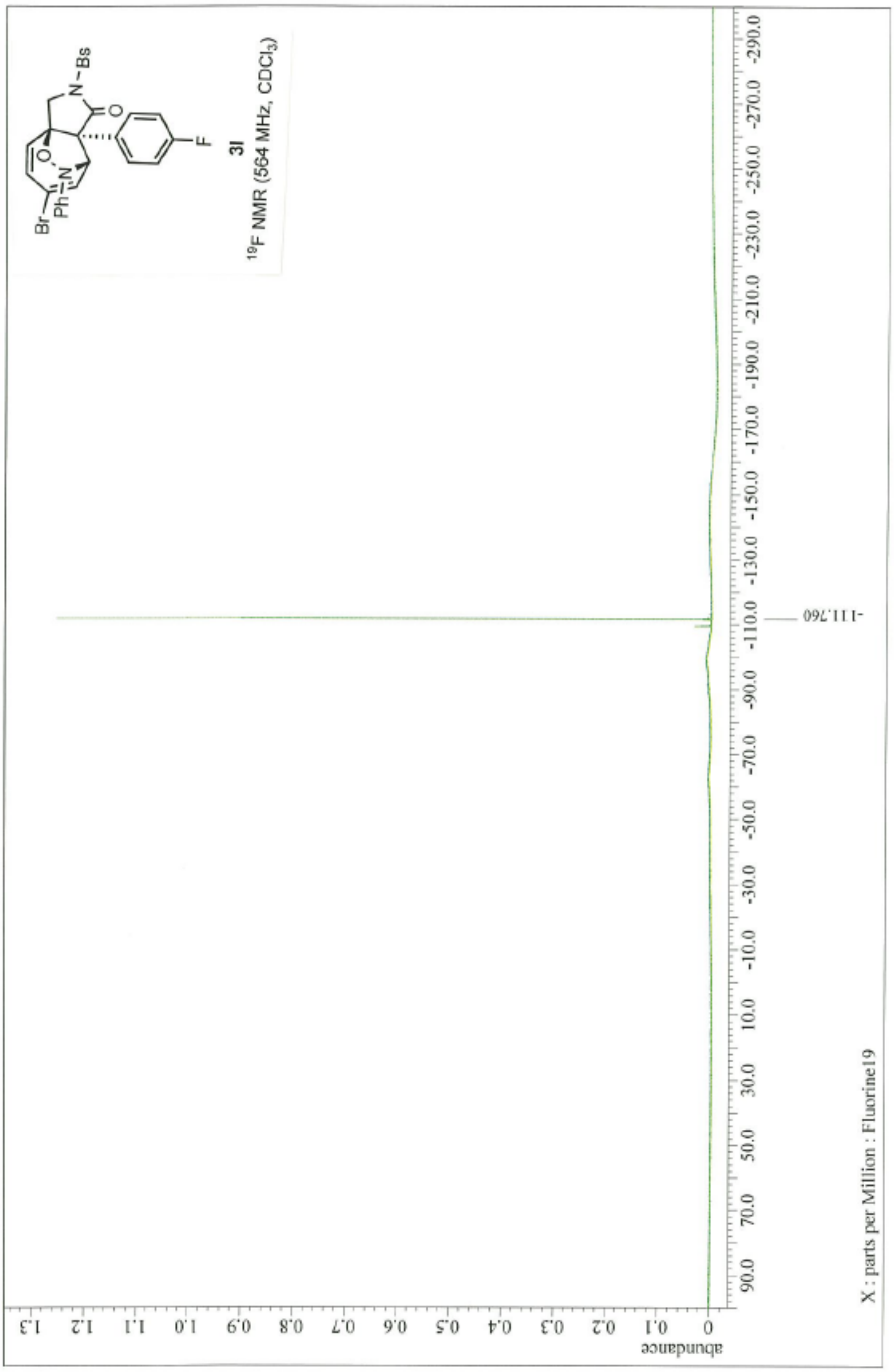
Br
Ph
N
O
N–Bs
O
F
3l
19F NMR (564 MHz, CDCl3)
-111.760
abundance
X : parts per Million : Fluorine19

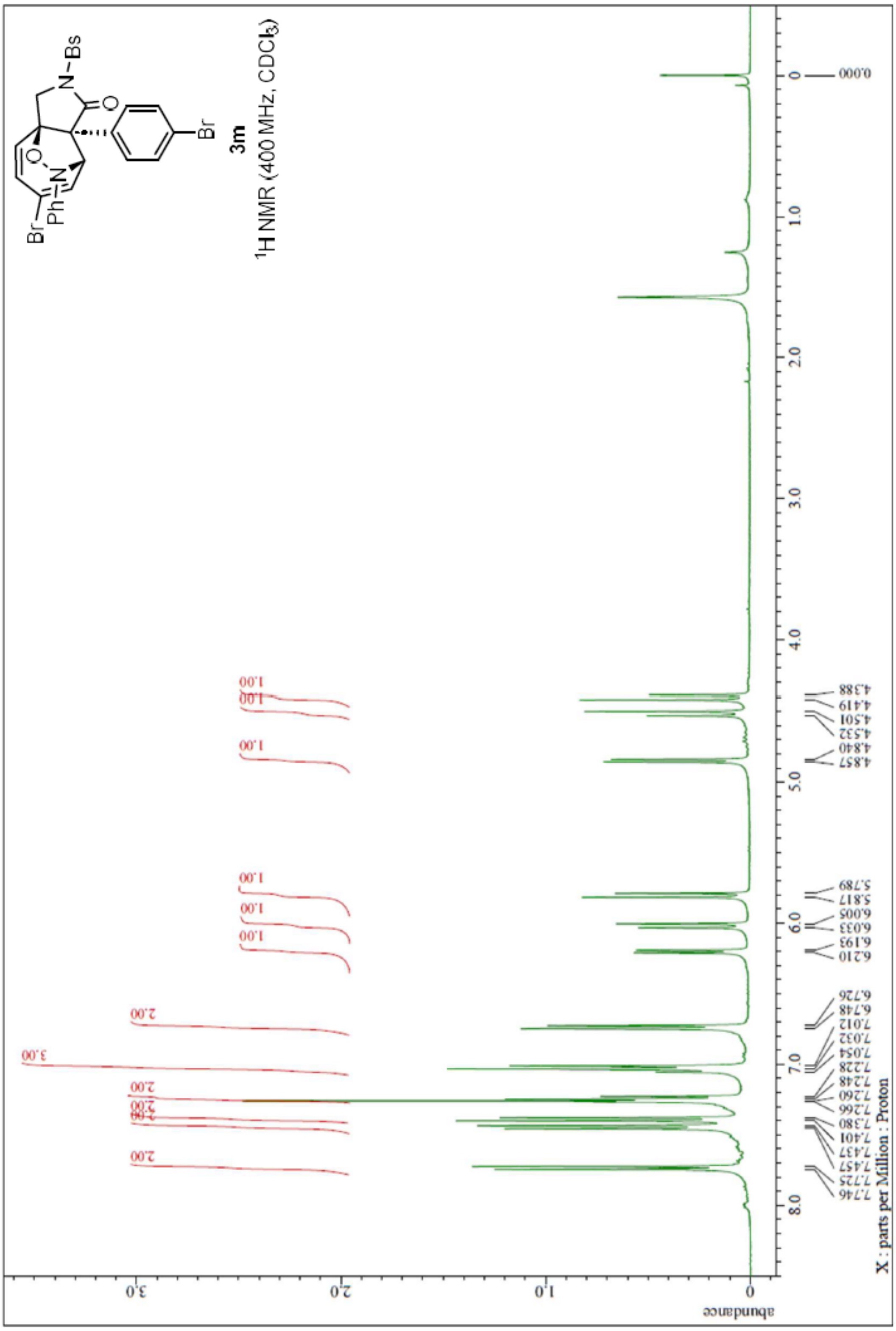
3m
1H NMR (400 MHz, CDCl3)
0.000
4.388
4.419
4.501
4.532
4.840
4.857
5.789
5.817
6.005
6.033
6.193
6.210
6.726
6.748
7.012
7.032
7.054
7.228
7.248
7.260
7.266
7.380
7.401
7.437
7.457
7.725
7.746
1.00
1.00
1.00
1.00
1.00
1.00
2.00
3.00
2.00
2.00
2.00
2.00
X : parts per Million : Proton
abundance

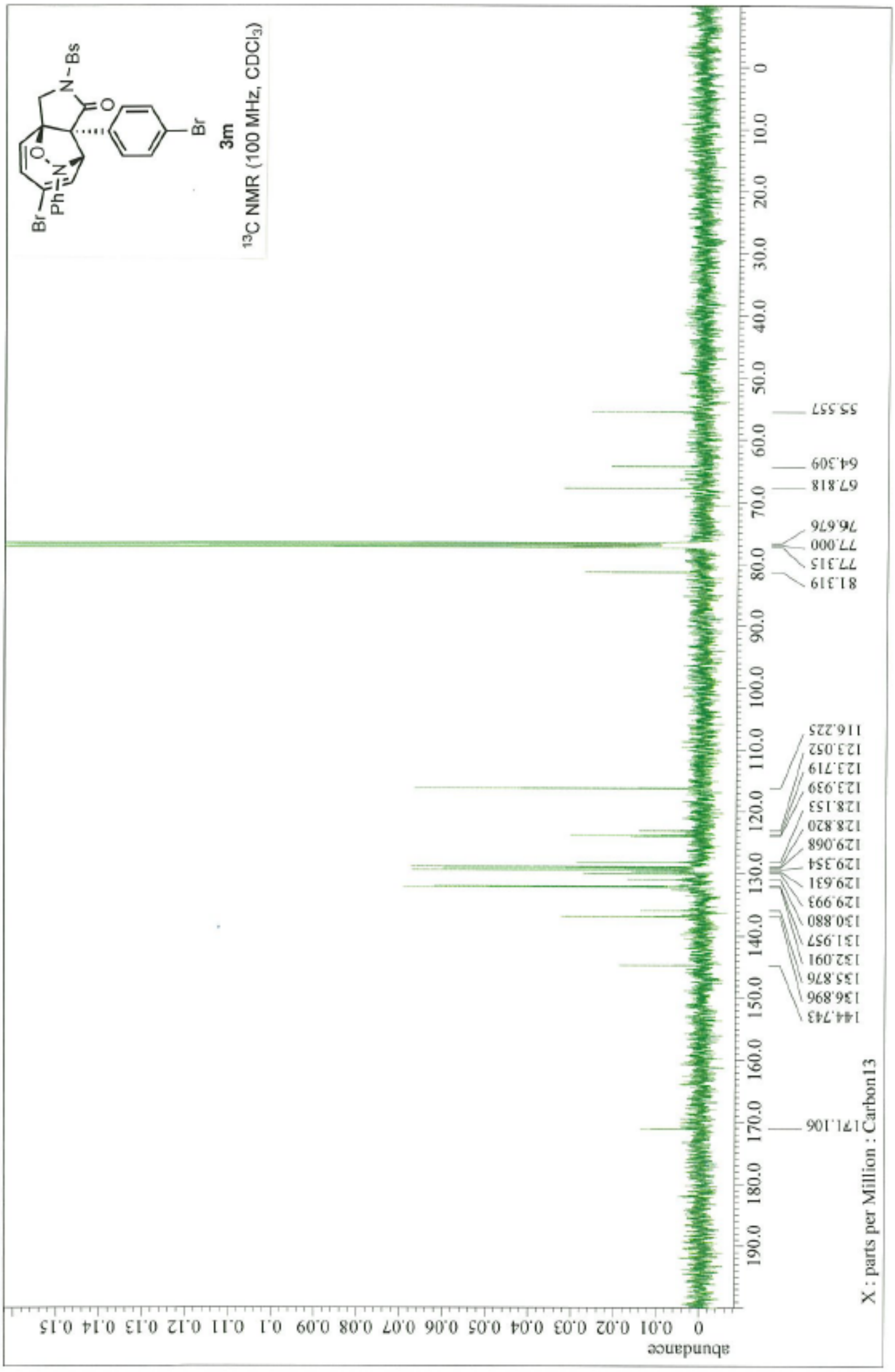

N–Bs
O
Br
Ph
3m
13C NMR (100 MHz, CDCl3)
55.557
64.309
67.818
76.676
77.000
77.315
81.319
116.225
123.052
123.719
123.939
128.153
128.820
129.068
129.354
129.631
129.993
130.880
131.957
132.091
135.876
136.896
144.743
171.106
X : parts per Million : Carbon13
abundance

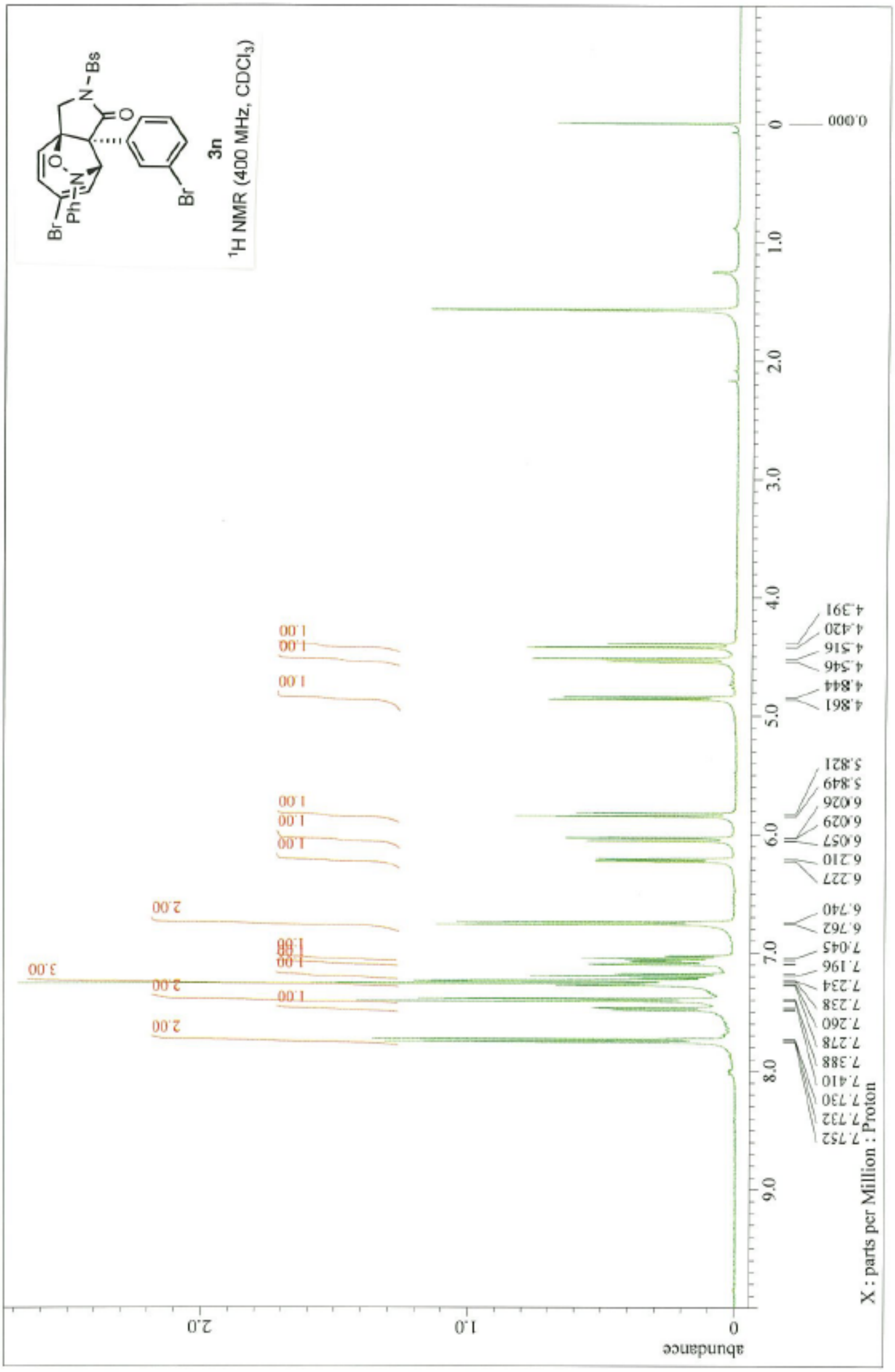
3n
^{1}H NMR (400 MHz, CDCl$_3$)
X : parts per Million : Proton
abundance

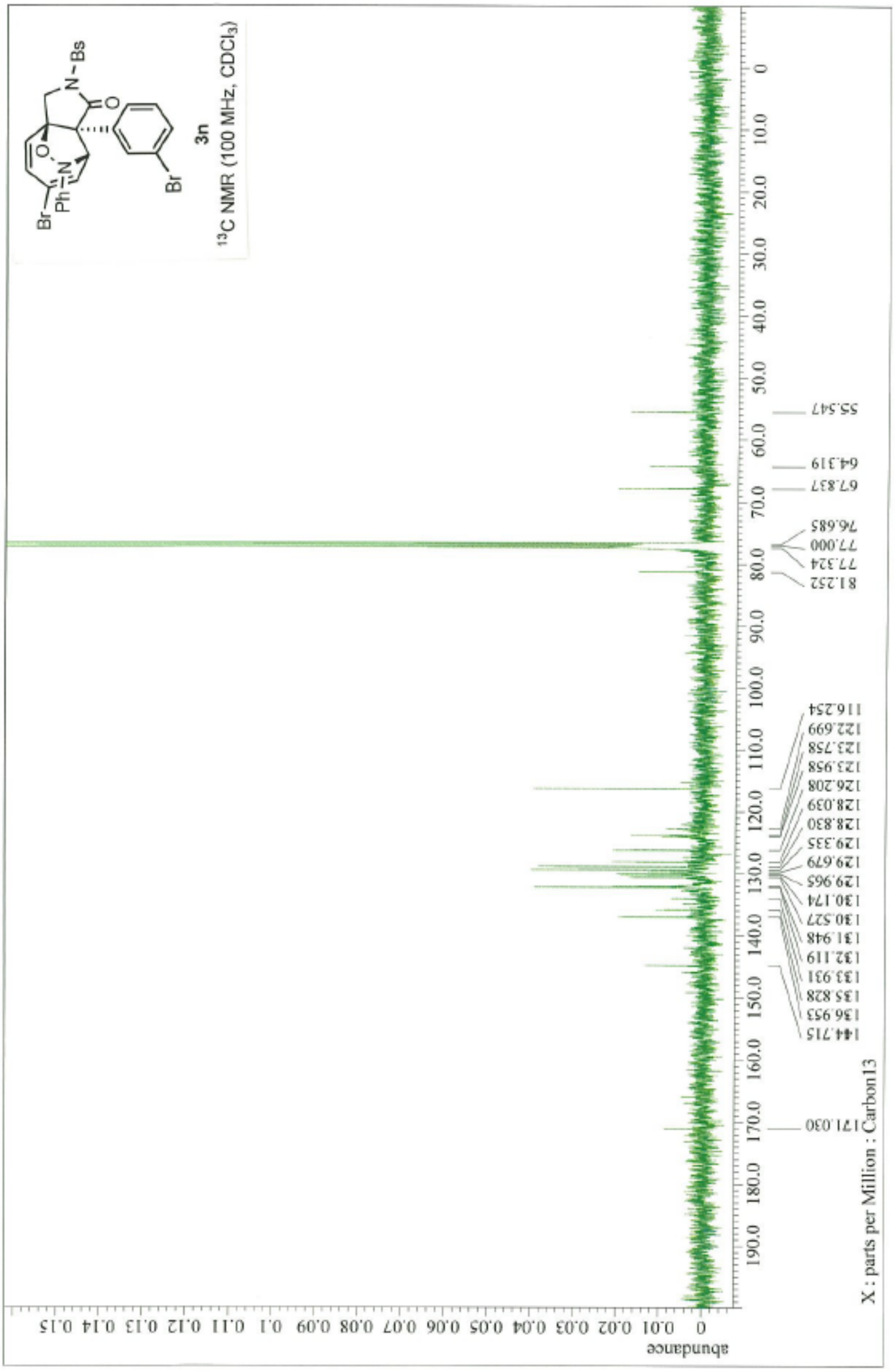
Br
Ph
N
O
N~Bs
O
Br
3n
13C NMR (100 MHz, CDCl3)
55.547
64.319
67.837
76.685
77.000
77.324
81.252
116.254
122.699
123.758
123.958
126.208
128.039
128.830
129.335
129.679
129.965
130.174
130.527
131.948
132.119
133.931
135.828
136.953
144.715
171.030
X : parts per Million : Carbon13
abundance

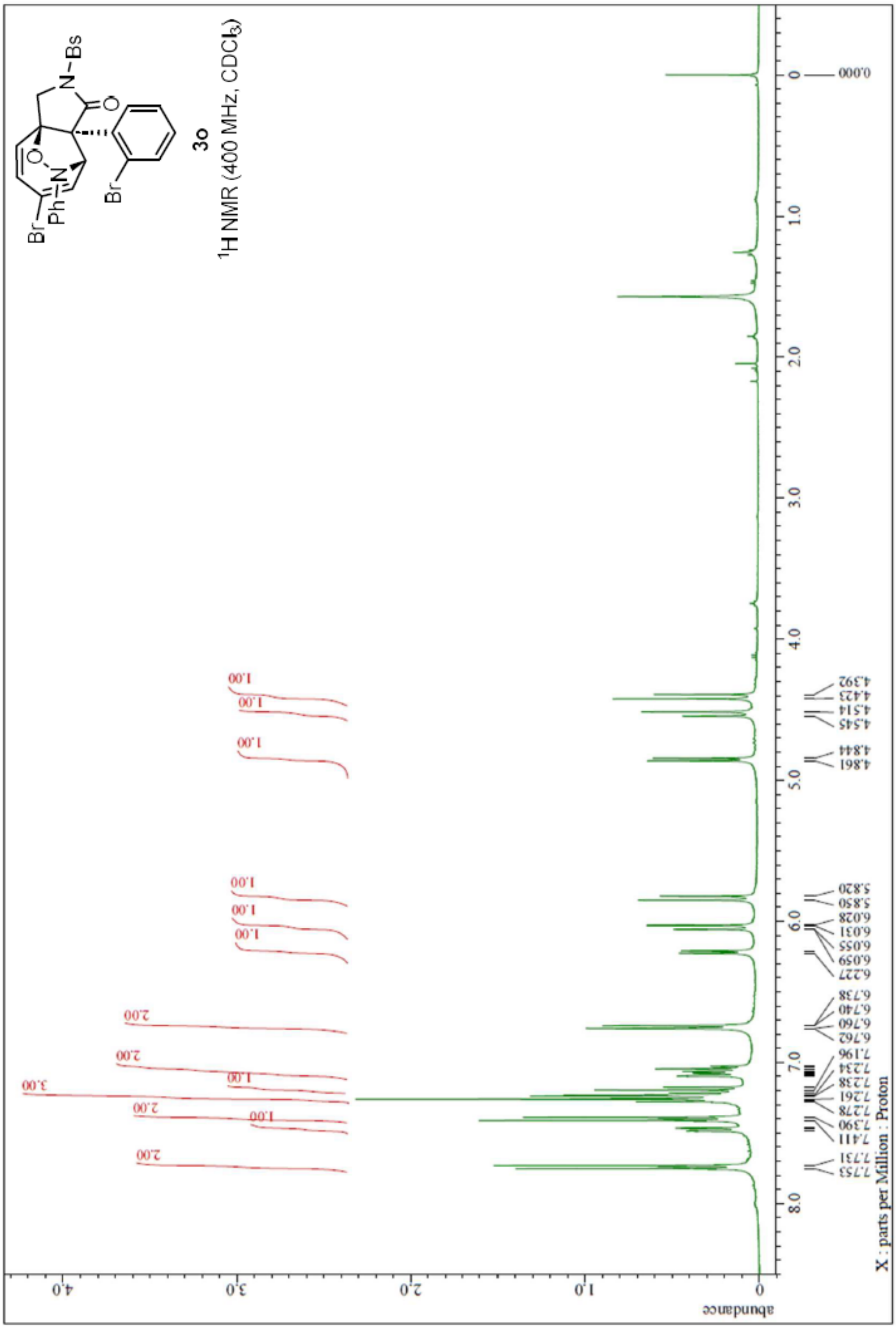
3o
1H NMR (400 MHz, CDCl3)
X : parts per Million : Proton
abundance

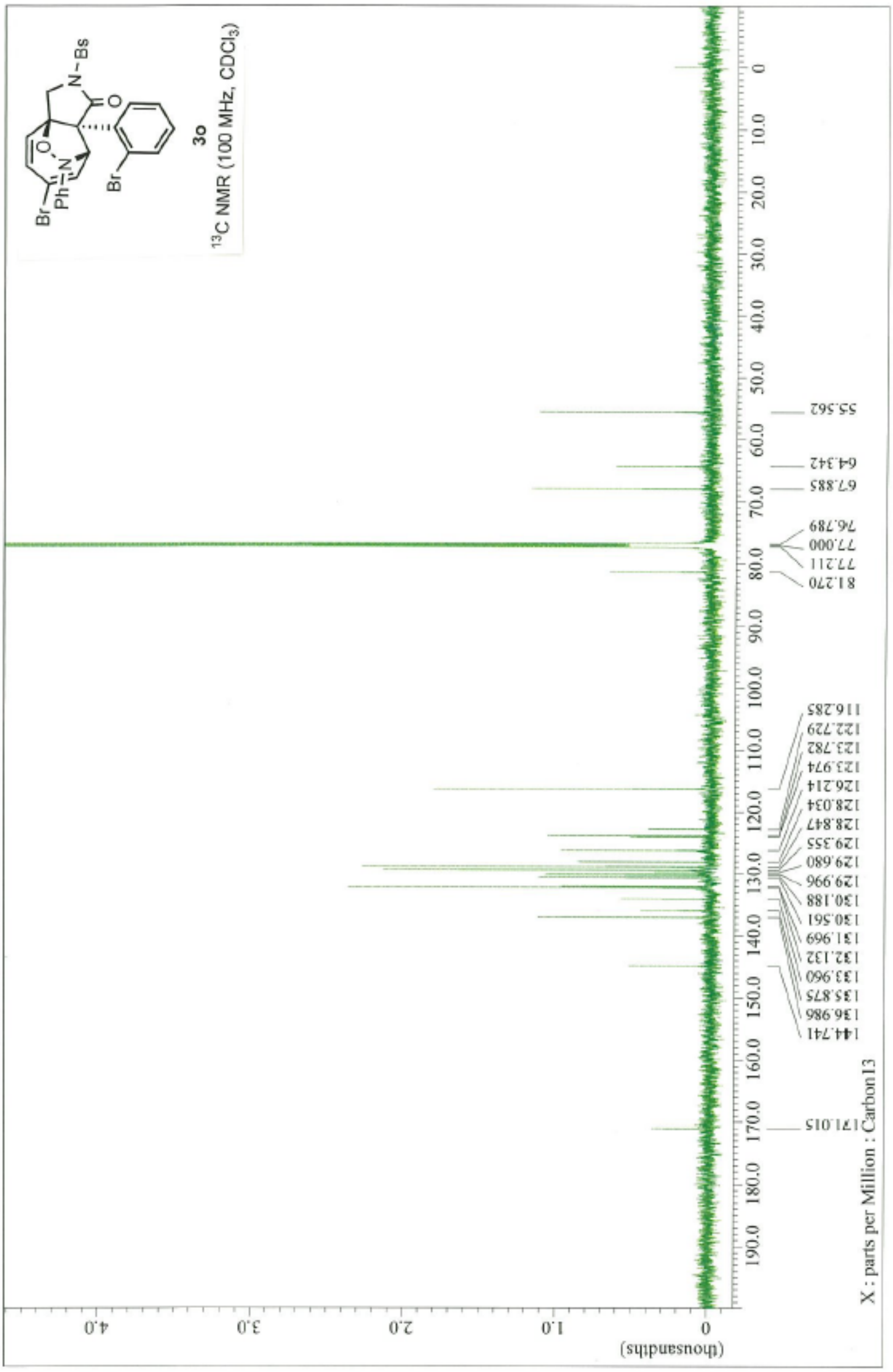

N~Bs
O
Br
Ph
N
3o
13C NMR (100 MHz, CDCl3)
55.562
64.342
67.885
76.789
77.000
77.211
81.270
116.285
122.729
123.782
123.974
126.214
128.034
128.847
129.355
129.680
129.996
130.188
130.561
131.969
132.132
133.960
135.875
136.986
144.741
171.015
X : parts per Million : Carbon13
(thousandths)

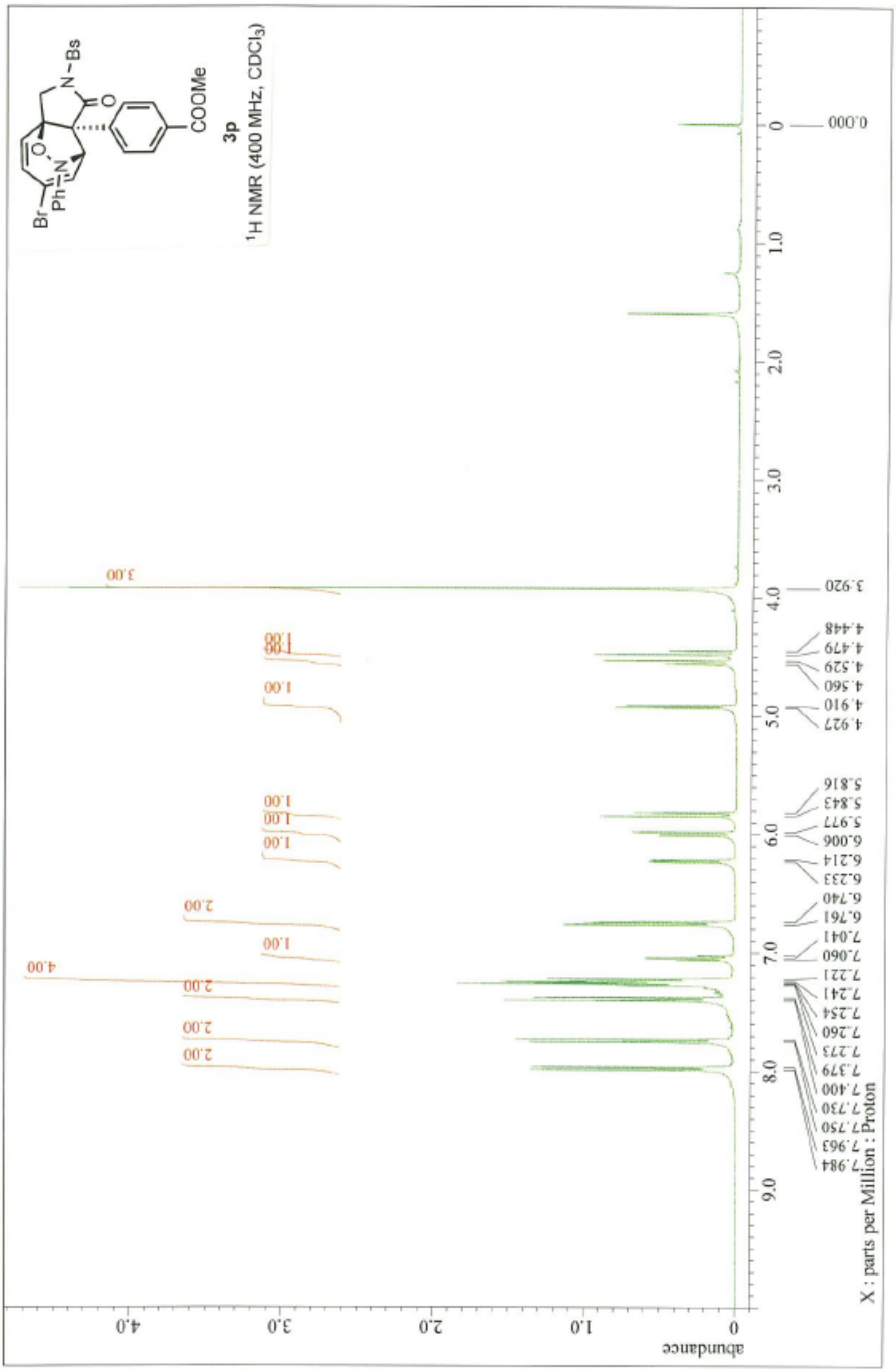
Br
Ph
N
O
N~Bs
O
COOMe
3p
1H NMR (400 MHz, CDCl3)
0.000
3.920
4.448
4.479
4.529
4.560
4.910
4.927
5.816
5.843
5.977
6.006
6.214
6.233
6.740
6.761
7.041
7.060
7.221
7.241
7.254
7.260
7.273
7.379
7.400
7.730
7.750
7.963
7.984
3.00
1.00
1.00
1.00
1.00
1.00
1.00
2.00
1.00
4.00
2.00
2.00
2.00
X : parts per Million : Proton
abundance

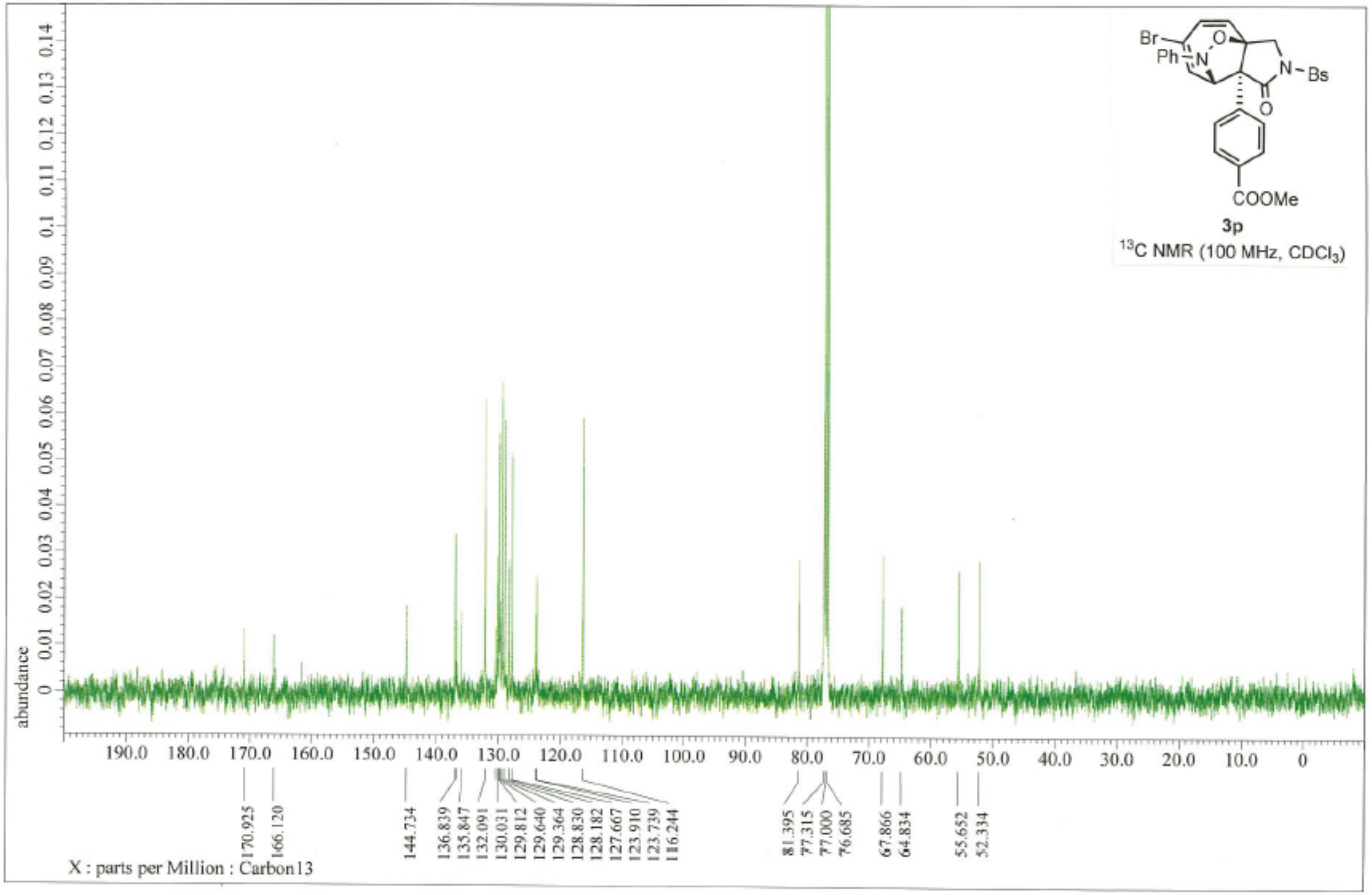

Br
Ph
N
O
N-Bs
O
COOMe
3p
^{13}C NMR (100 MHz, $CDCl_3$)
abundance
0 0.01 0.02 0.03 0.04 0.05 0.06 0.07 0.08 0.09 0.1 0.11 0.12 0.13 0.14
190.0 180.0 170.0 160.0 150.0 140.0 130.0 120.0 110.0 100.0 90.0 80.0 70.0 60.0 50.0 40.0 30.0 20.0 10.0 0
170.925
166.120
144.734
136.839
135.847
132.091
130.031
129.812
129.640
129.364
128.830
128.182
127.667
123.910
123.739
116.244
81.395
77.315
77.000
76.685
67.866
64.834
55.652
52.334
X : parts per Million : Carbon13

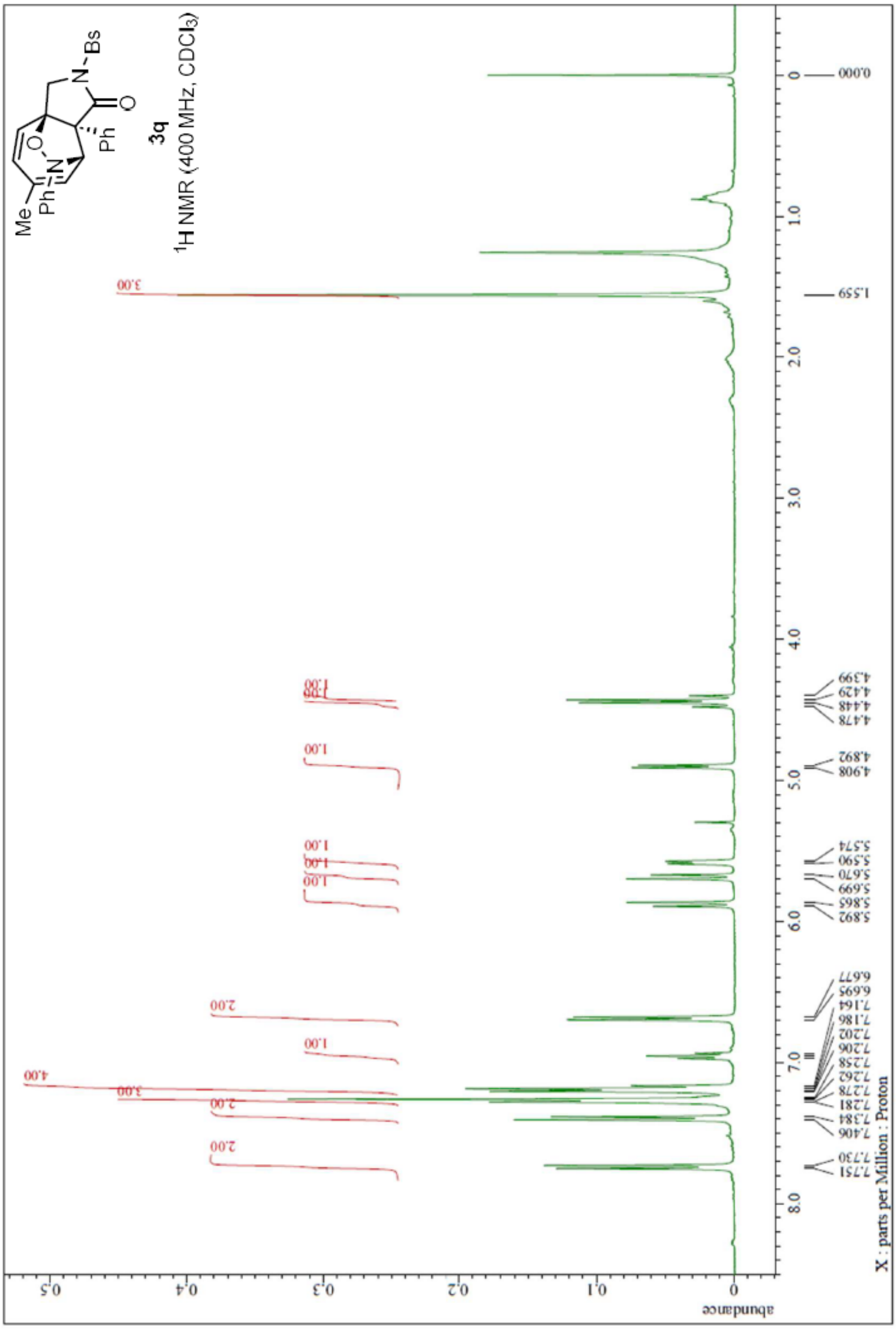
3q
1H NMR (400 MHz, CDCl3)
Me
Ph
N
O
N
Bs
Ph
O
X : parts per Million : Proton
abundance
0.000
1.559
4.399
4.429
4.448
4.478
4.892
4.908
5.574
5.590
5.670
5.699
5.865
5.892
6.677
6.695
7.164
7.186
7.202
7.206
7.258
7.262
7.278
7.281
7.384
7.406
7.730
7.751

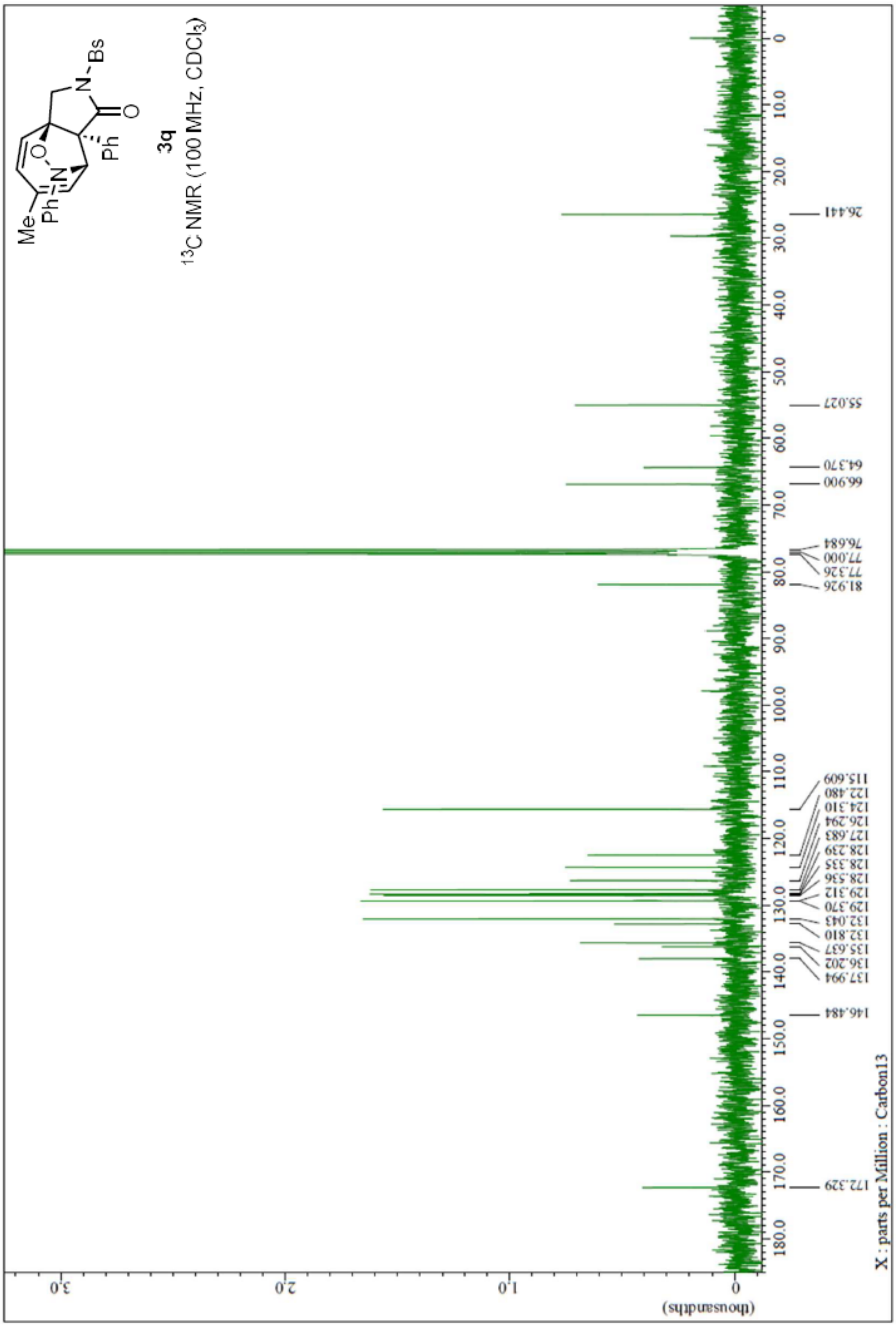
3q
13C NMR (100 MHz, CDCl3)
26.441
55.027
64.370
66.900
76.684
77.000
77.326
81.926
115.609
122.480
124.310
126.294
127.683
128.239
128.335
128.536
129.312
129.370
132.043
132.810
135.637
136.202
137.994
146.484
172.329
X : parts per Million : Carbon13
(thousandths)

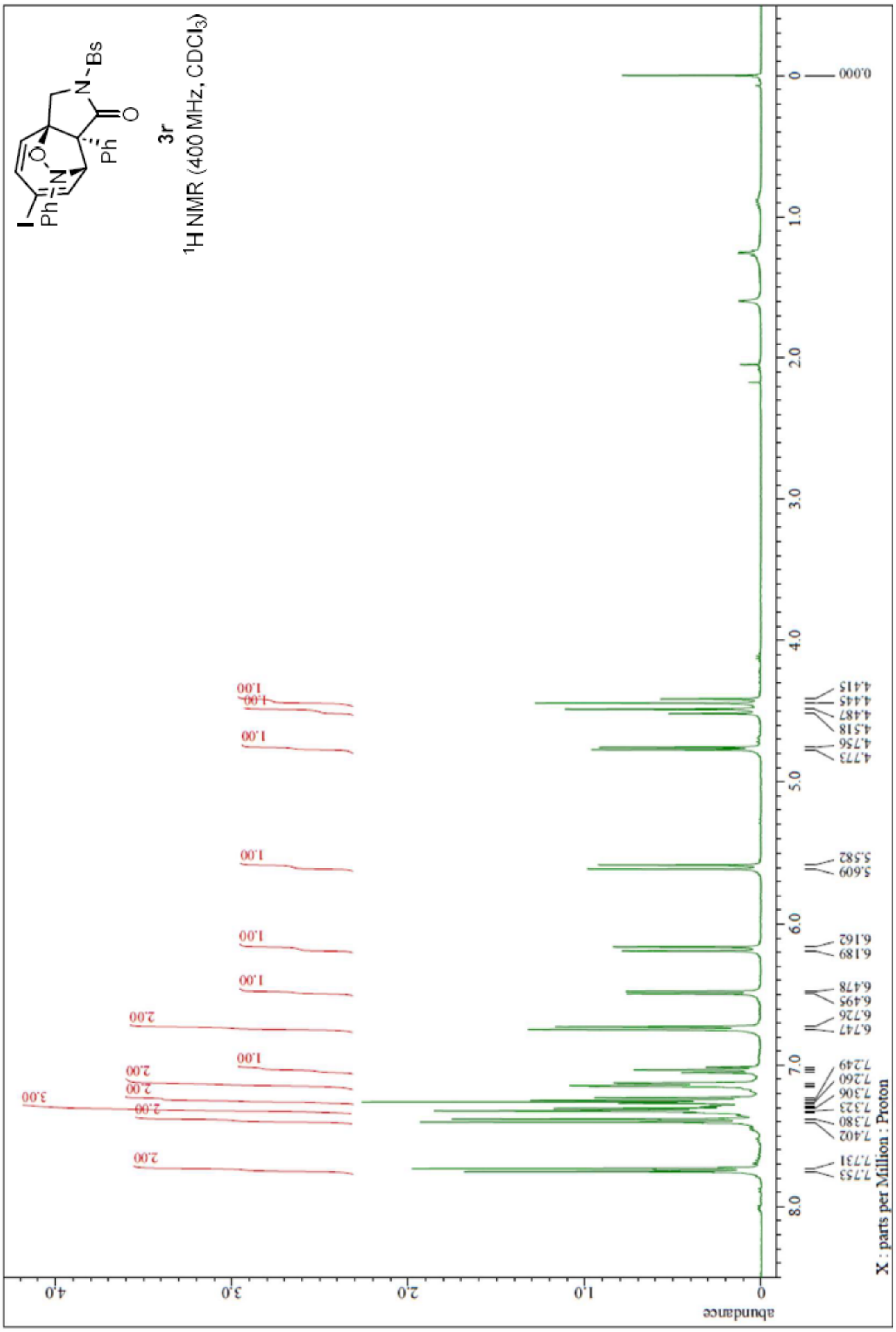

Ph
N
O
I
Ph
N~Bs
O
3r
1H NMR (400 MHz, CDCl3)
0.000
4.415
4.445
4.487
4.518
4.756
4.773
5.582
5.609
6.162
6.189
6.478
6.495
6.726
6.747
7.249
7.260
7.306
7.323
7.380
7.402
7.731
7.753
X : parts per Million : Proton
abundance

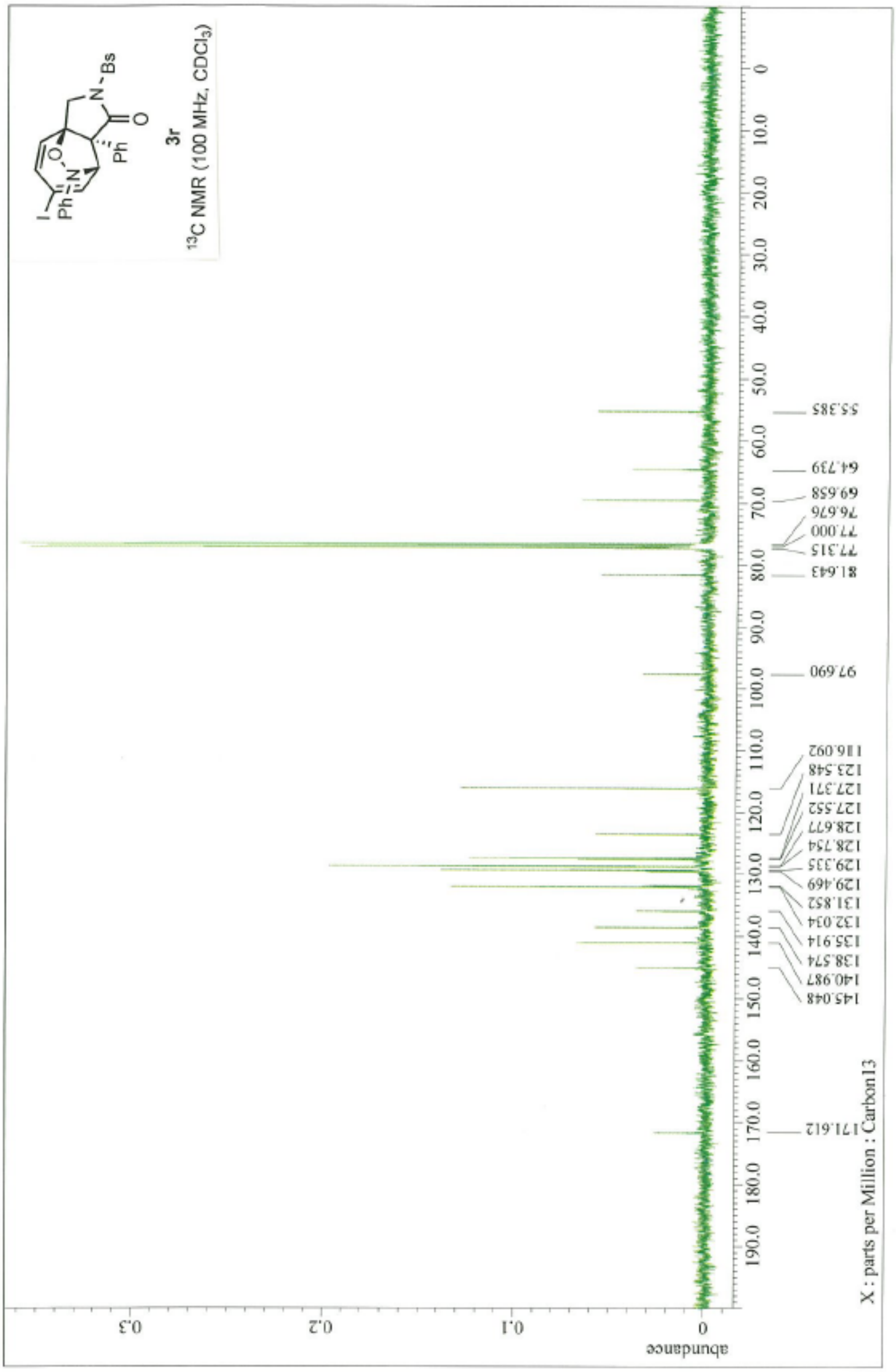
3r
13C NMR (100 MHz, CDCl3)
55.385
64.739
69.658
76.676
77.000
77.315
81.643
97.690
116.092
123.548
127.371
127.552
128.677
128.754
129.335
129.469
131.852
132.034
135.914
138.574
140.987
145.048
171.612
X : parts per Million : Carbon13
abundance

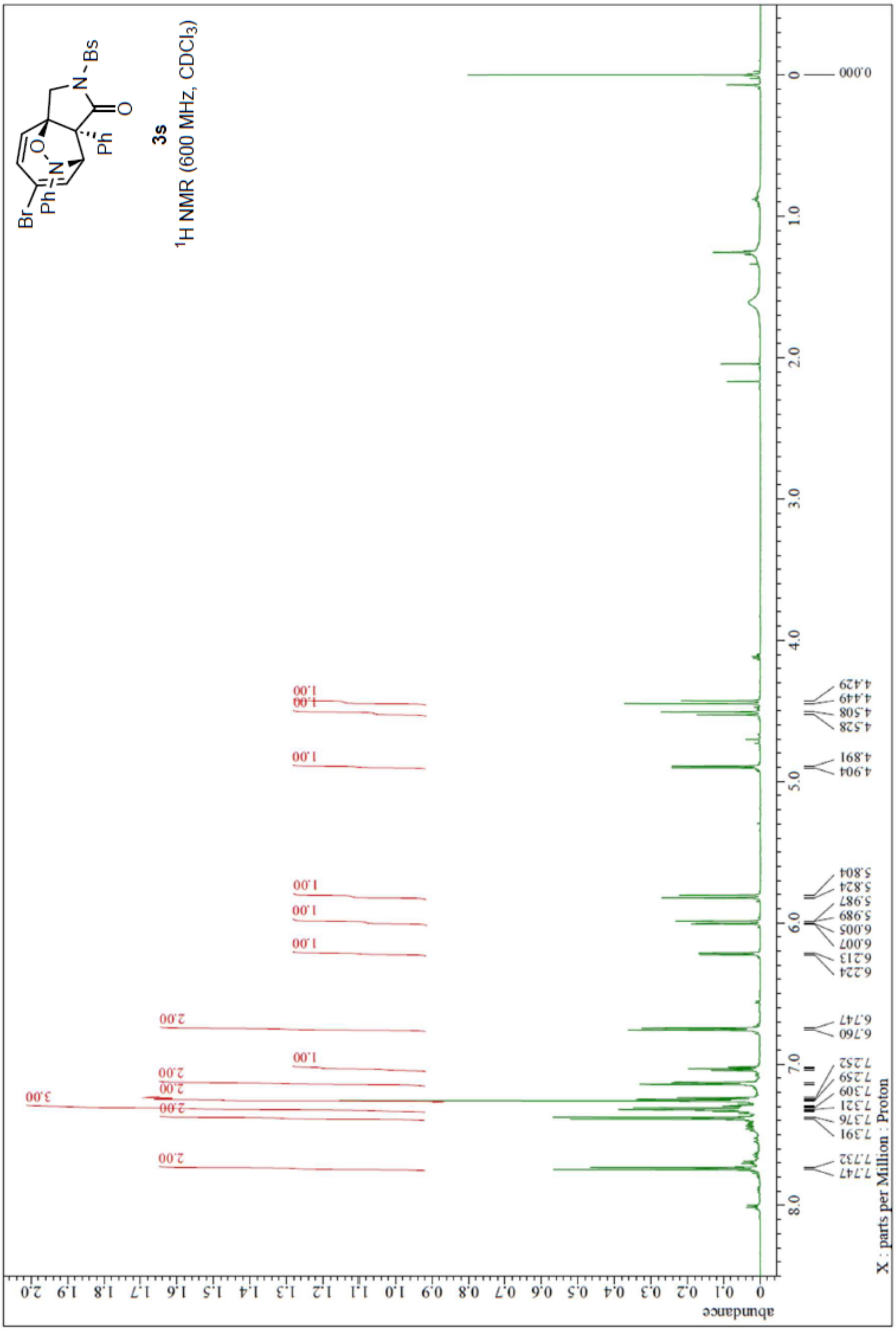
3s
1H NMR (600 MHz, CDCl3)
0.000
4.429
4.449
4.508
4.528
4.891
4.904
5.804
5.824
5.987
5.989
6.005
6.007
6.213
6.224
6.747
6.760
7.252
7.259
7.309
7.321
7.376
7.391
7.732
7.747
X : parts per Million : Proton
abundance

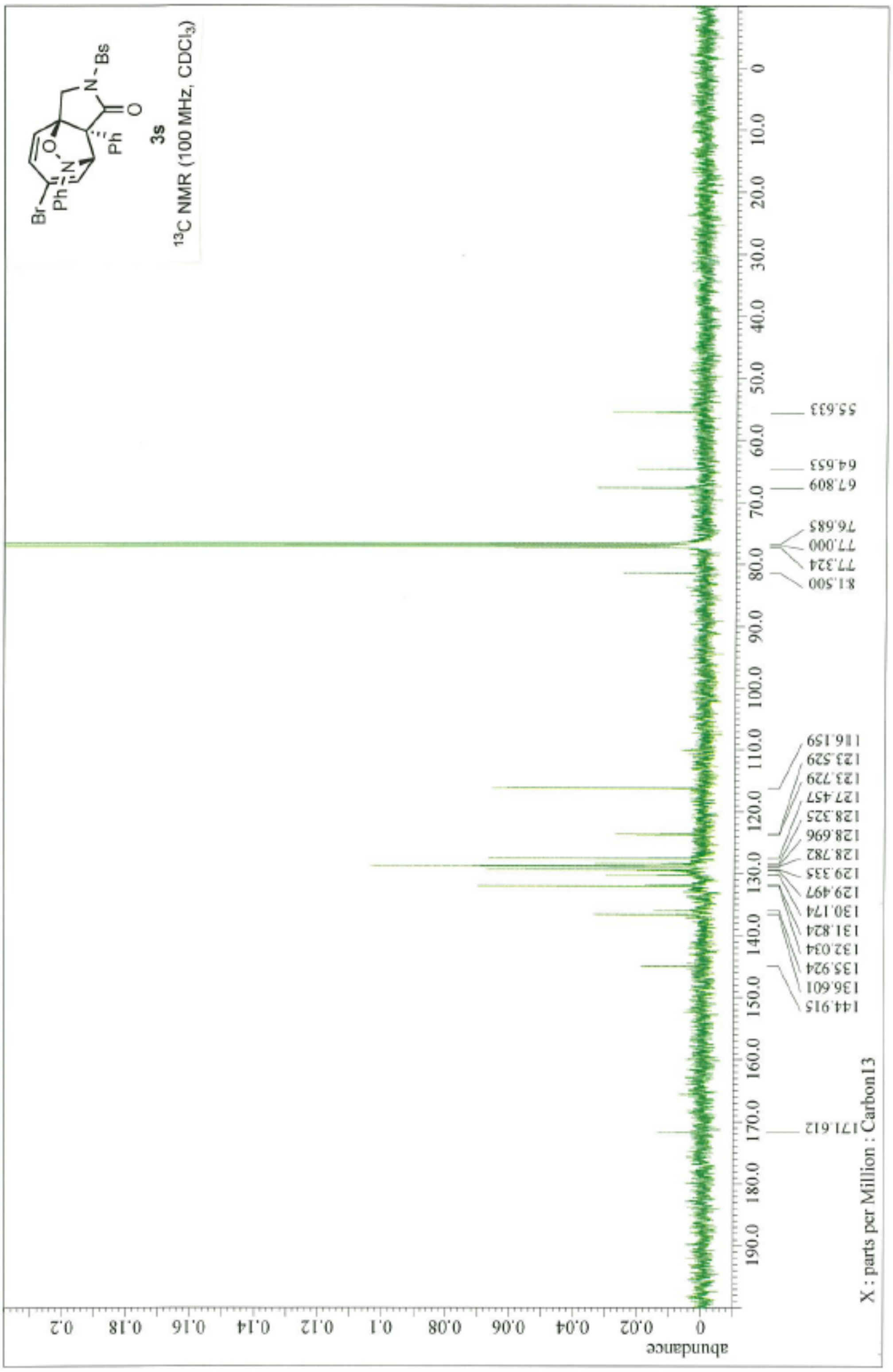
3s
13C NMR (100 MHz, CDCl3)
55.633
64.653
67.809
76.685
77.000
77.324
81.500
116.159
123.529
123.729
127.457
128.325
128.696
128.782
129.335
129.497
130.174
131.824
132.034
135.924
136.601
144.915
171.612
X : parts per Million : Carbon13
abundance

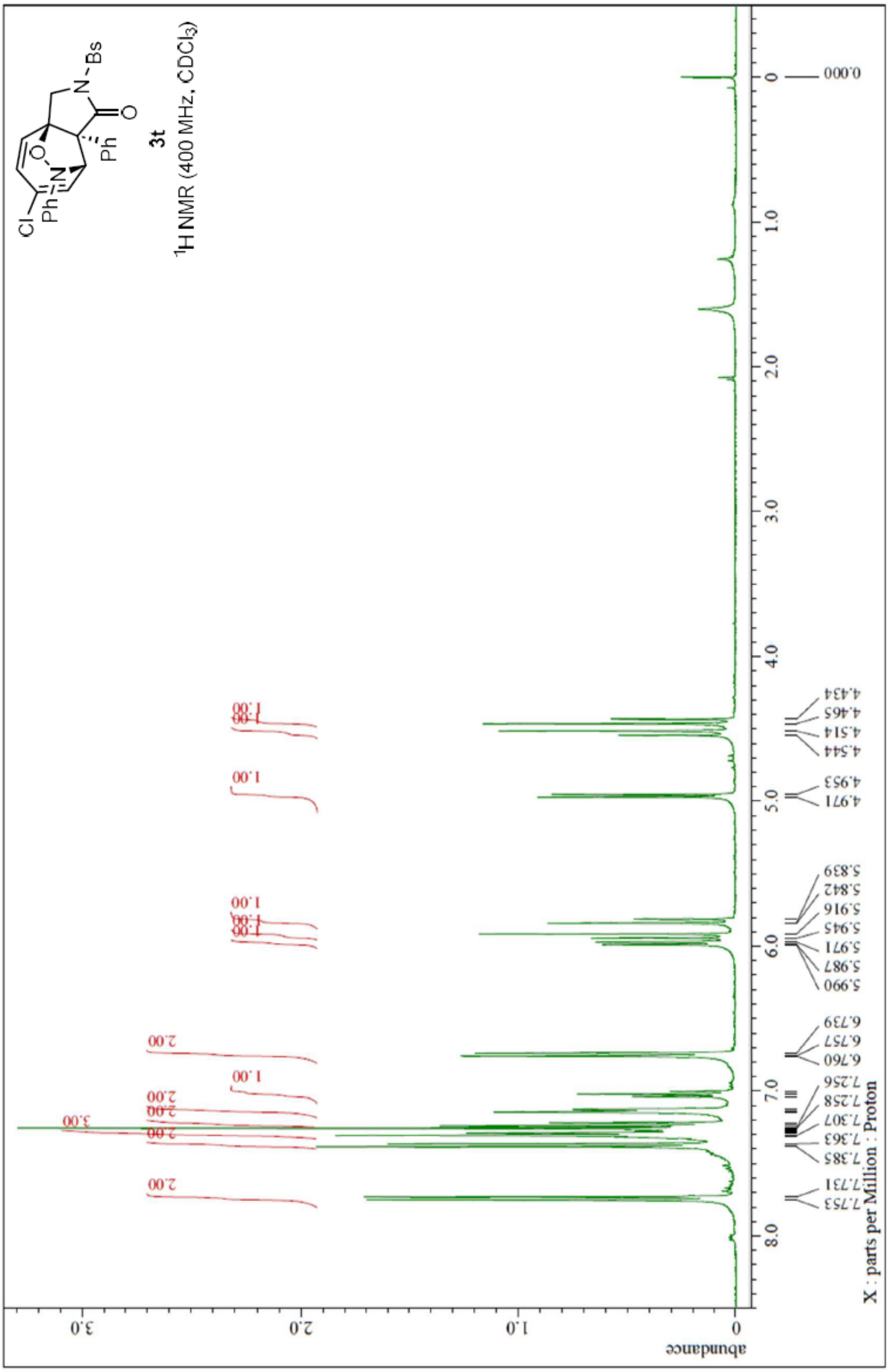

3t
1H NMR (400 MHz, CDCl3)
0.000
4.434
4.465
4.514
4.544
4.953
4.971
5.839
5.842
5.916
5.945
5.971
5.987
5.990
6.739
6.757
6.760
7.256
7.258
7.307
7.363
7.385
7.731
7.753
X : parts per Million : Proton
abundance

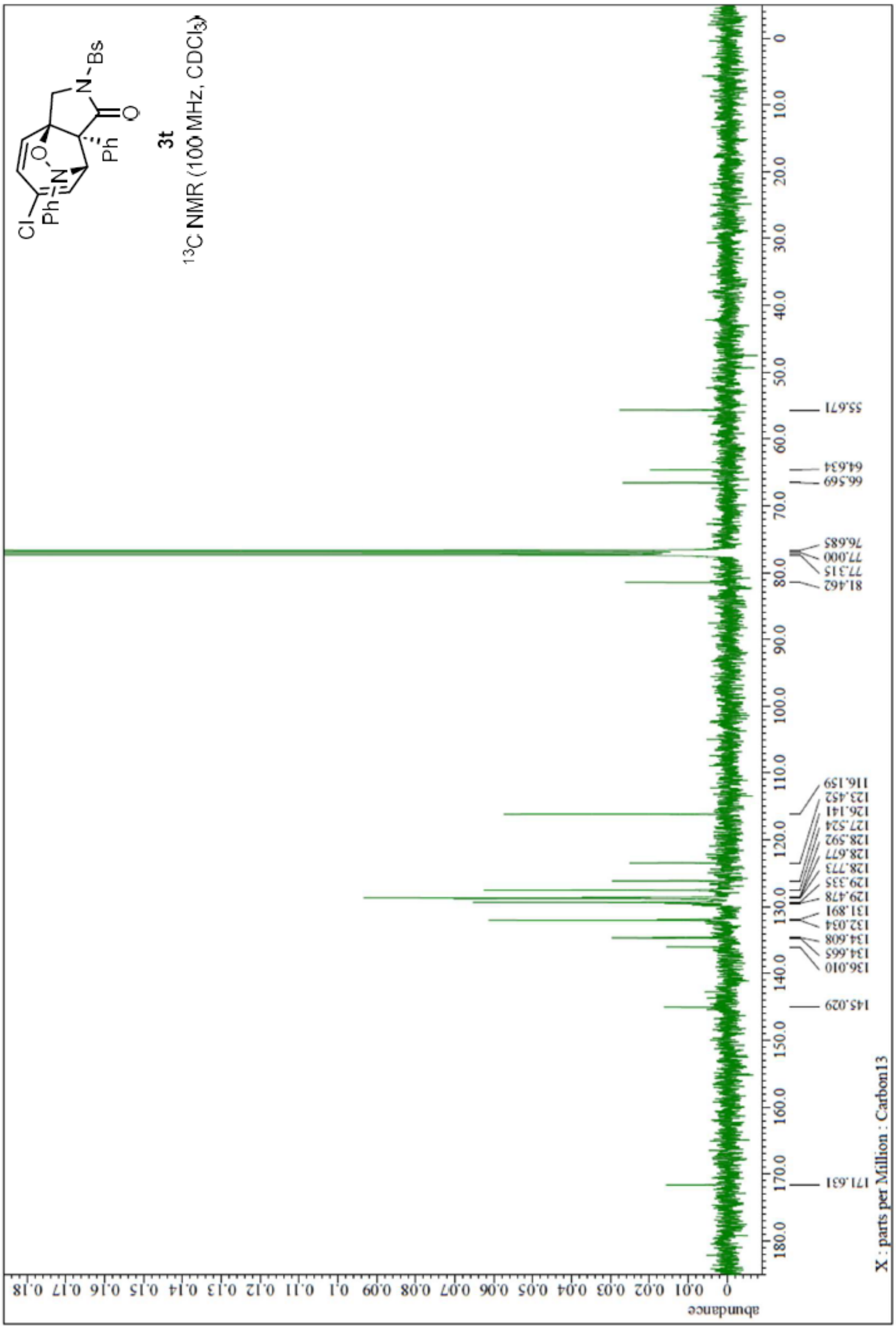

3t
13C NMR (100 MHz, CDCl3)
55.671
64.634
66.569
76.685
77.000
77.315
81.462
116.159
123.452
126.141
127.524
128.592
128.677
128.773
129.335
129.478
131.891
132.034
134.608
134.665
136.010
145.029
171.631
X : parts per Million : Carbon13
abundance

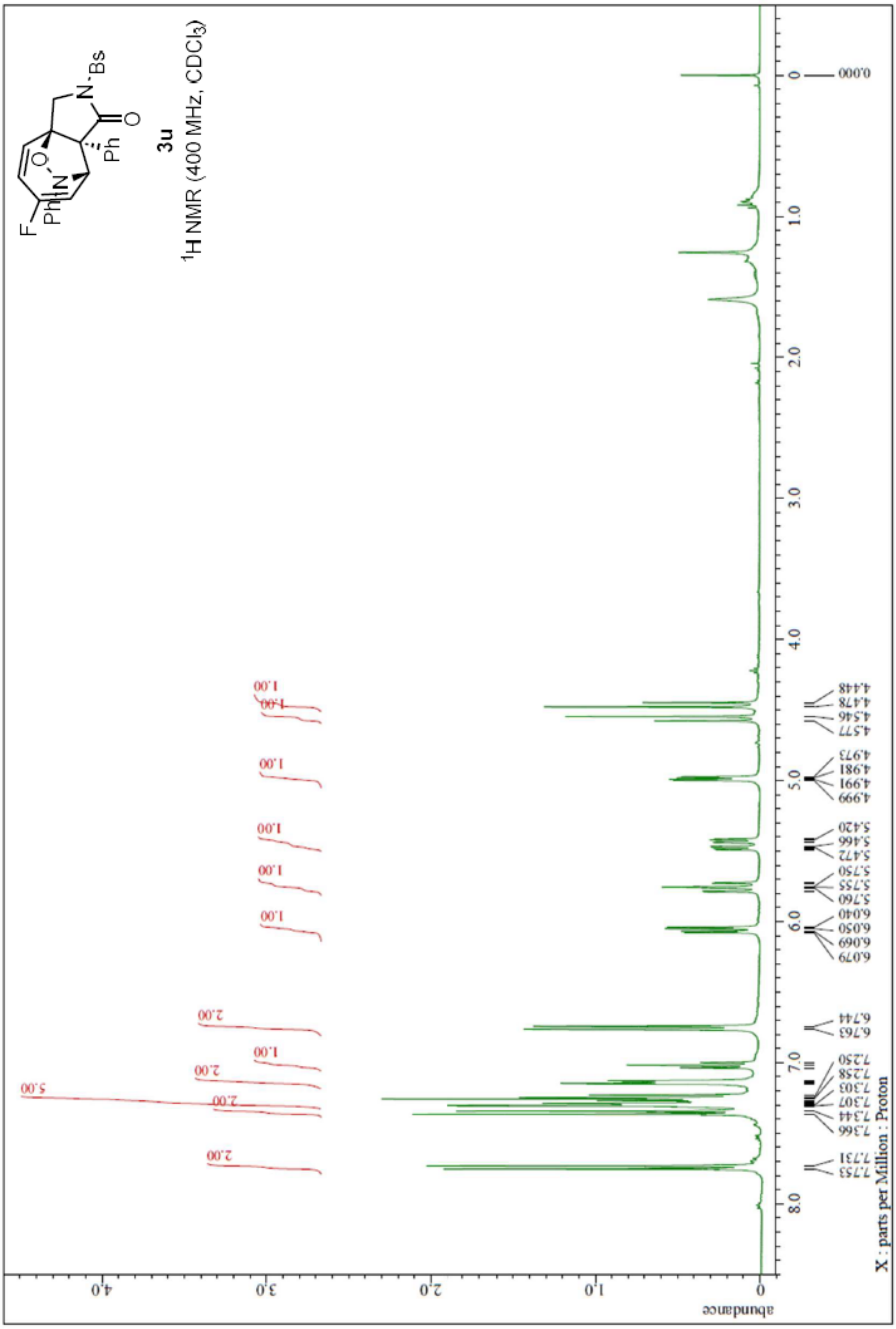
3u
1H NMR (400 MHz, CDCl3)
X : parts per Million : Proton
abundance

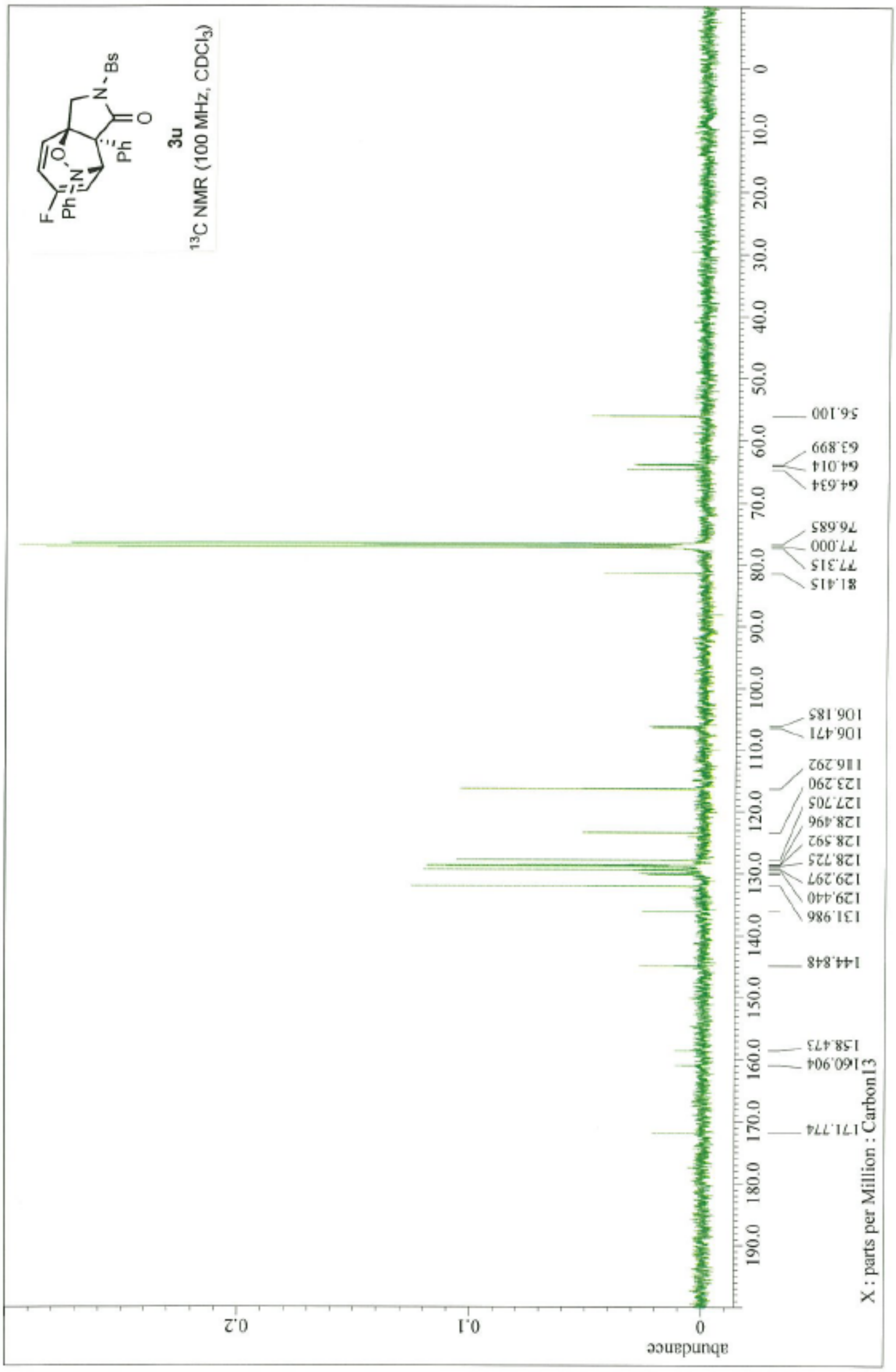

3u
13C NMR (100 MHz, CDCl3)
56.100
63.899
64.014
64.634
76.685
77.000
77.315
81.415
106.185
106.471
116.292
123.290
127.705
128.496
128.592
128.725
129.297
129.440
131.986
144.848
158.473
160.904
171.774
X : parts per Million : Carbon13
abundance

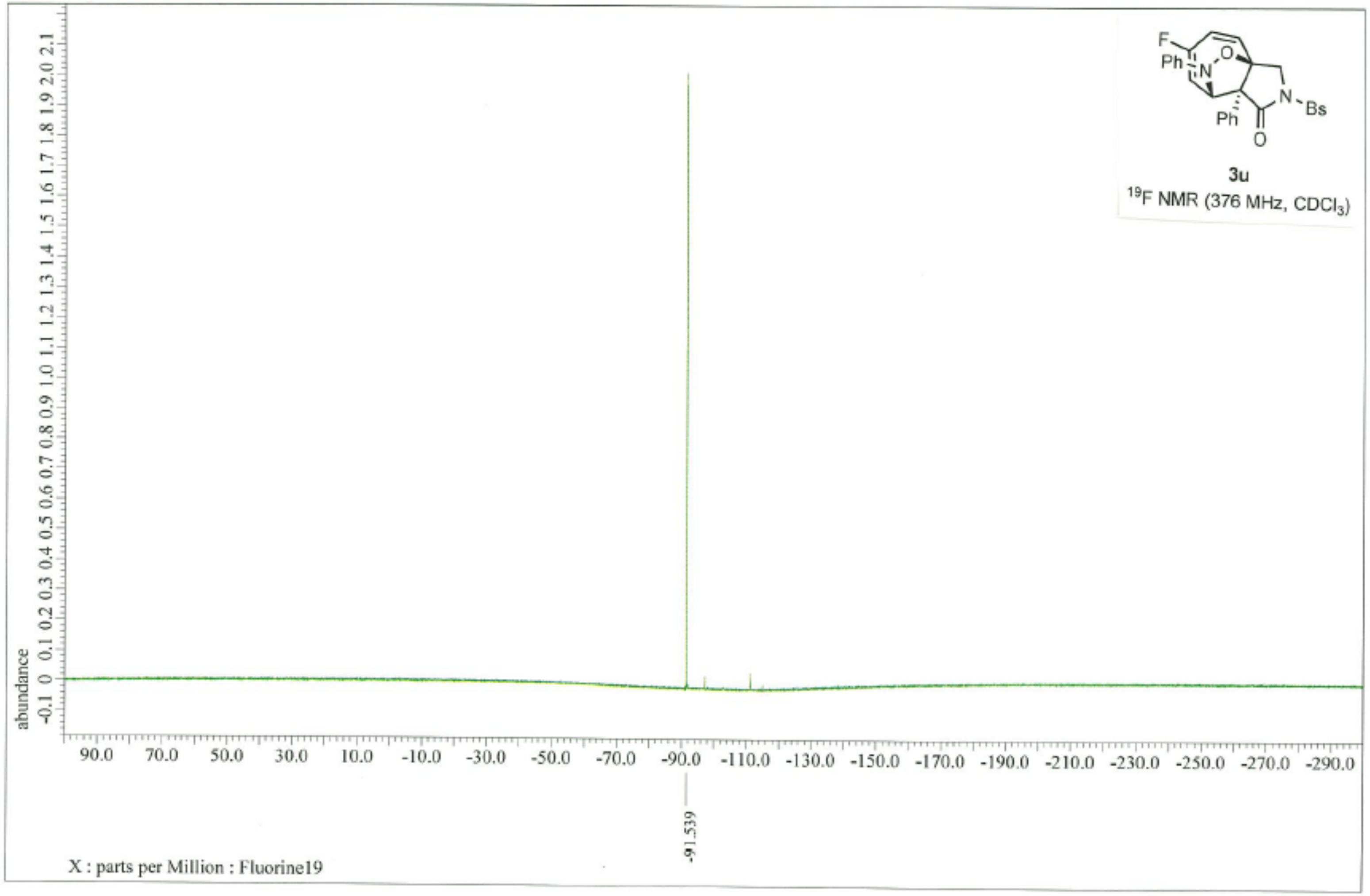

3u
^{19}F NMR (376 MHz, CDCl$_3$)
-91.539
abundance
X : parts per Million : Fluorine19

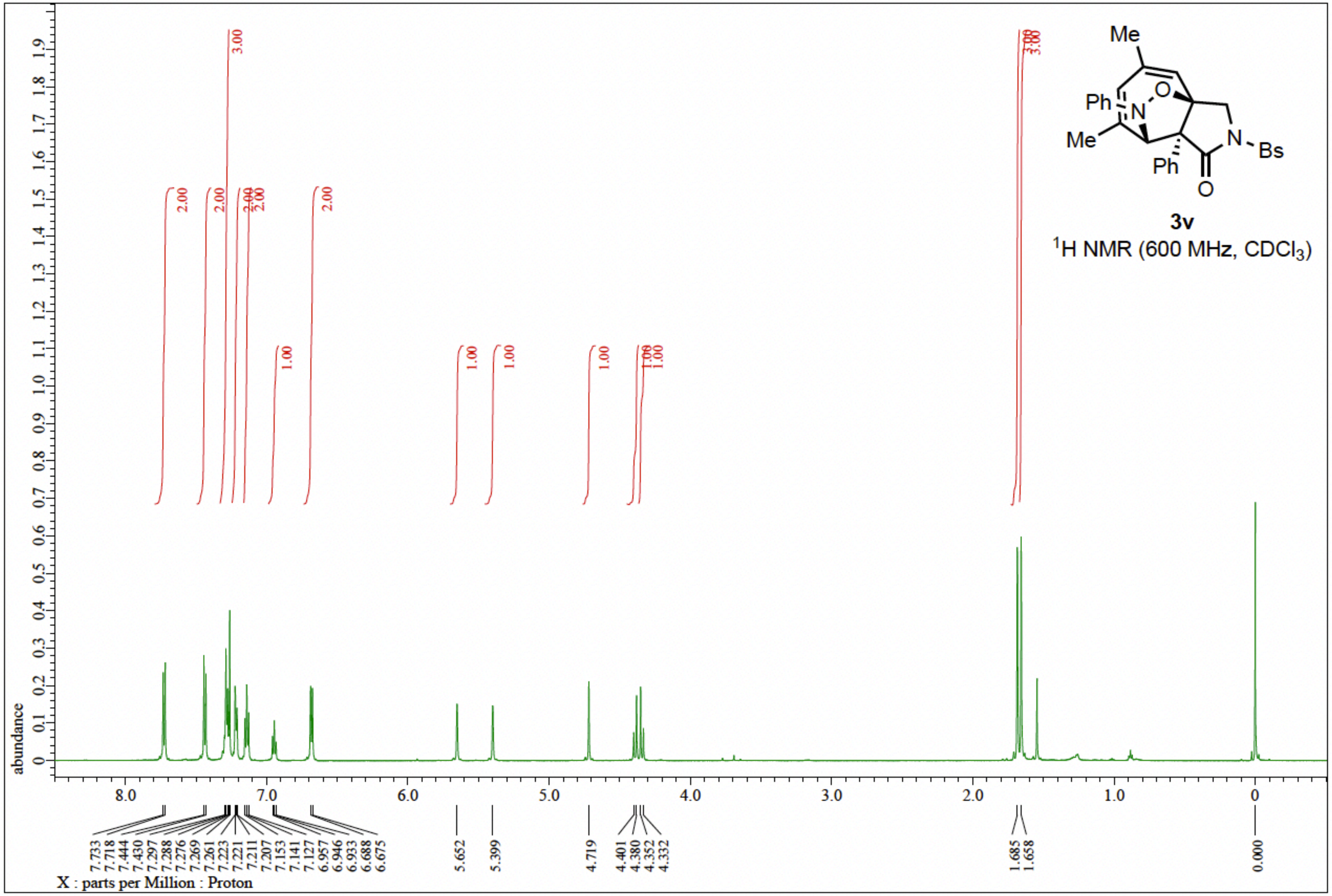

Me
Ph
N
O
Me
Ph
N
Bs
O
3v
^{1}H NMR (600 MHz, CDCl$_3$)
abundance
X : parts per Million : Proton
7.733
7.718
7.444
7.430
7.297
7.288
7.276
7.269
7.261
7.223
7.221
7.211
7.207
7.153
7.141
7.127
6.957
6.946
6.933
6.688
6.675
5.652
5.399
4.719
4.401
4.380
4.352
4.332
1.685
1.658
0.000
2.00
2.00
3.00
2.00
2.00
1.00
2.00
1.00
1.00
1.00
1.00
1.00
3.00
3.00

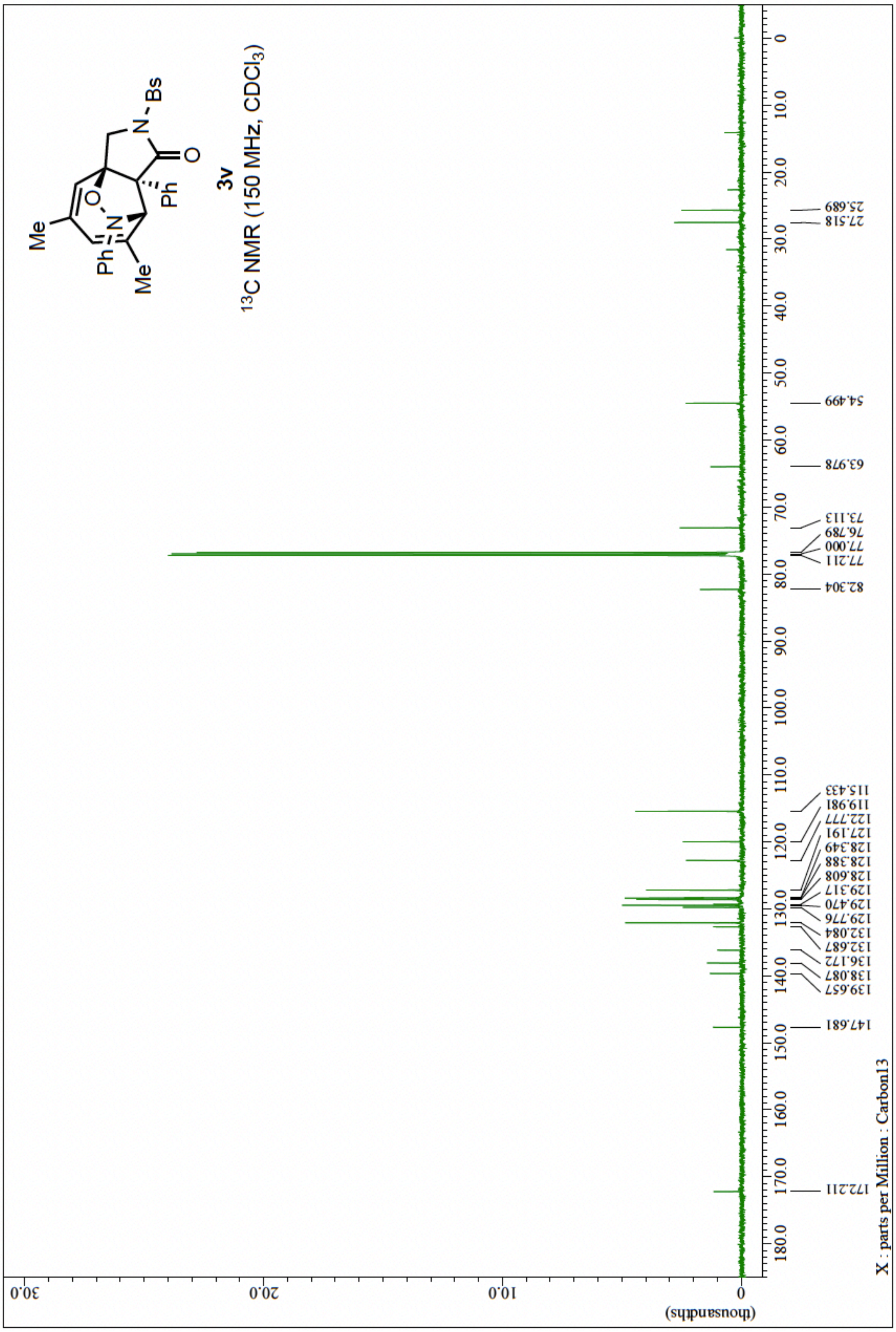

3v
^{13}C NMR (150 MHz, CDCl$_3$)
25.689
27.518
54.499
63.978
73.113
76.789
77.000
77.211
82.304
115.433
119.981
122.777
127.191
128.349
128.388
128.608
129.317
129.470
129.776
132.084
132.687
136.172
138.087
139.657
147.681
172.211
X : parts per Million : Carbon13
(thousandths)

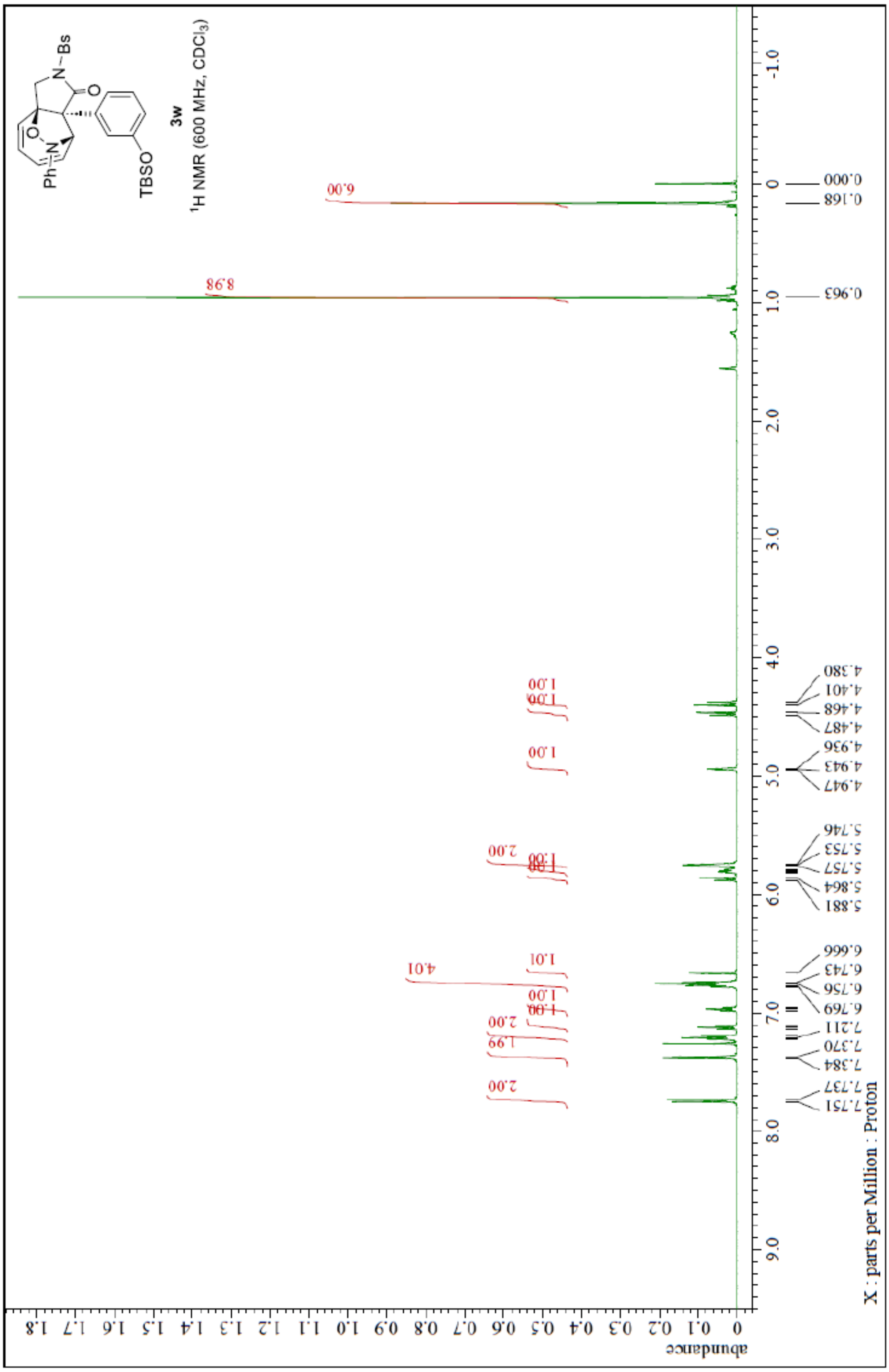

N–Bs
O
Ph
N
TBSO
3w
1H NMR (600 MHz, CDCl3)
0.000
0.168
0.963
4.380
4.401
4.468
4.487
4.936
4.943
4.947
5.746
5.753
5.757
5.864
5.881
6.666
6.743
6.756
6.769
7.211
7.370
7.384
7.737
7.751
X : parts per Million : Proton
abundance

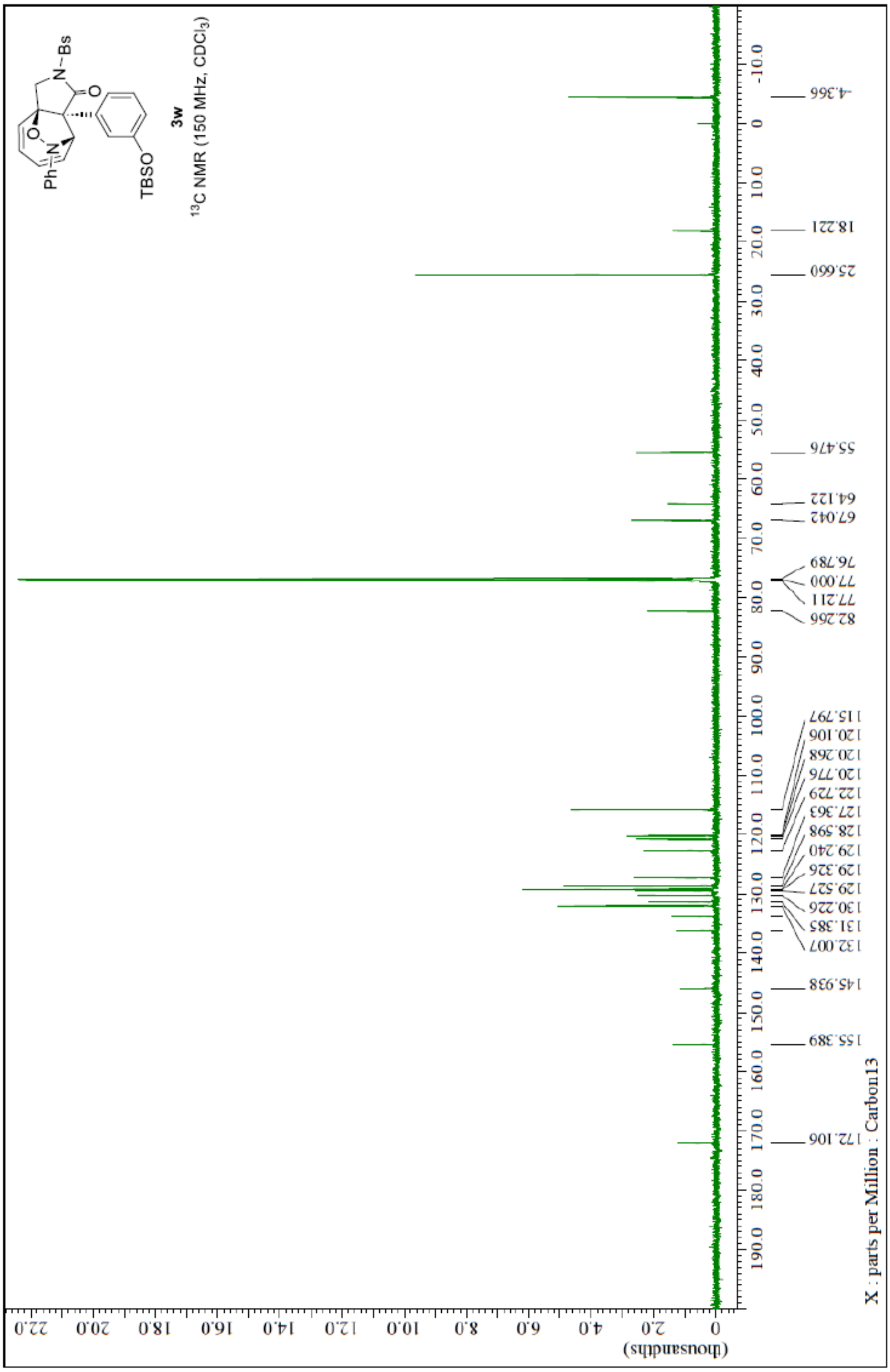
3w
13C NMR (150 MHz, CDCl3)
TBSO
Ph
N~Bs
-4.366
18.221
25.660
55.476
64.122
67.042
76.789
77.000
77.211
82.266
115.797
120.106
120.268
120.776
122.729
127.363
128.598
129.240
129.326
129.527
130.226
131.385
132.007
145.938
155.389
172.106
X : parts per Million : Carbon13
(thousandths)

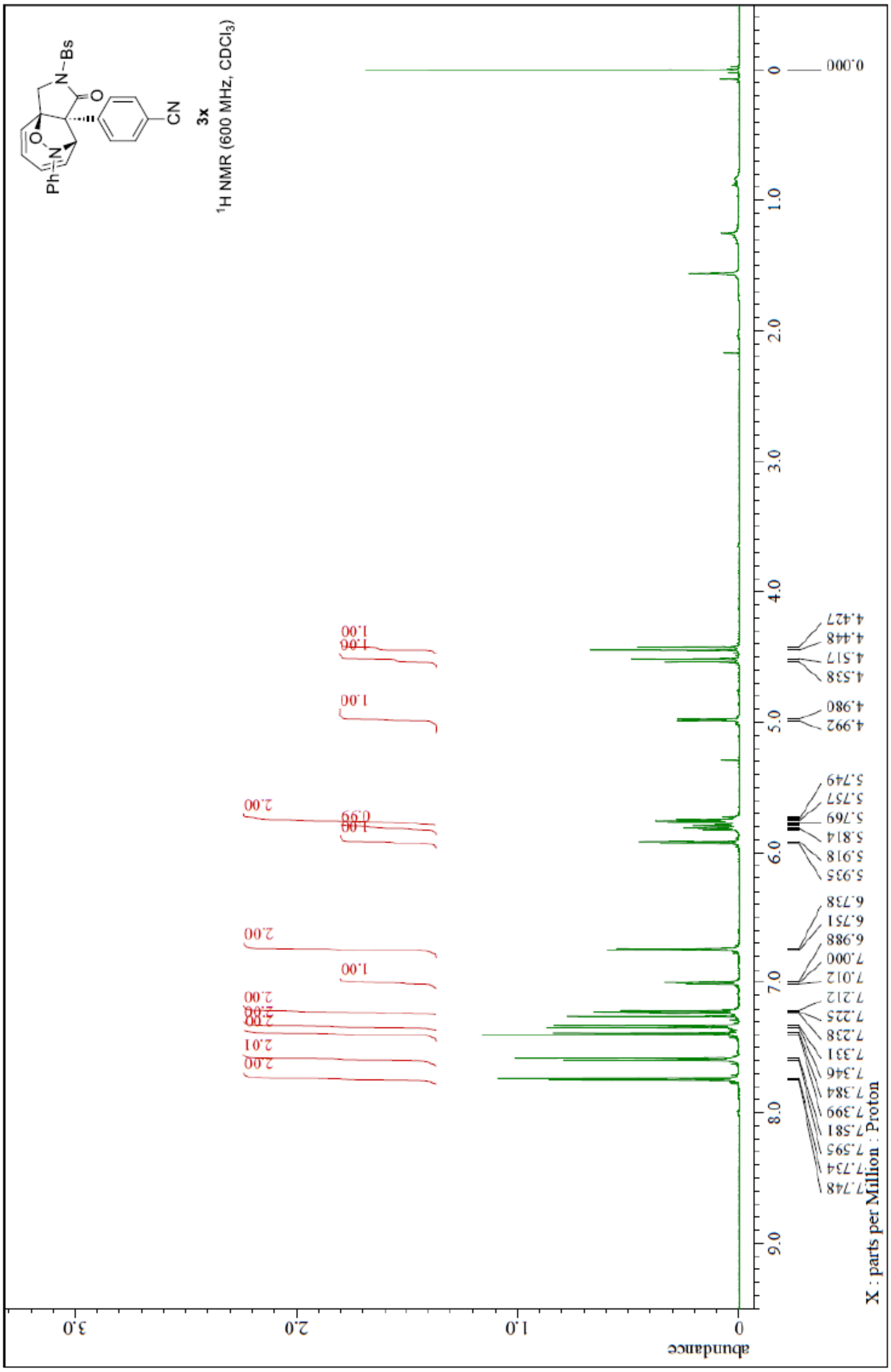

N~Bs
O
Ph
N
CN
3x
1H NMR (600 MHz, CDCl3)
X : parts per Million : Proton
abundance
0.000
4.427
4.448
4.517
4.538
4.980
4.992
5.749
5.757
5.769
5.814
5.918
5.935
6.738
6.751
6.988
7.000
7.012
7.212
7.225
7.238
7.331
7.346
7.384
7.399
7.581
7.595
7.734
7.748

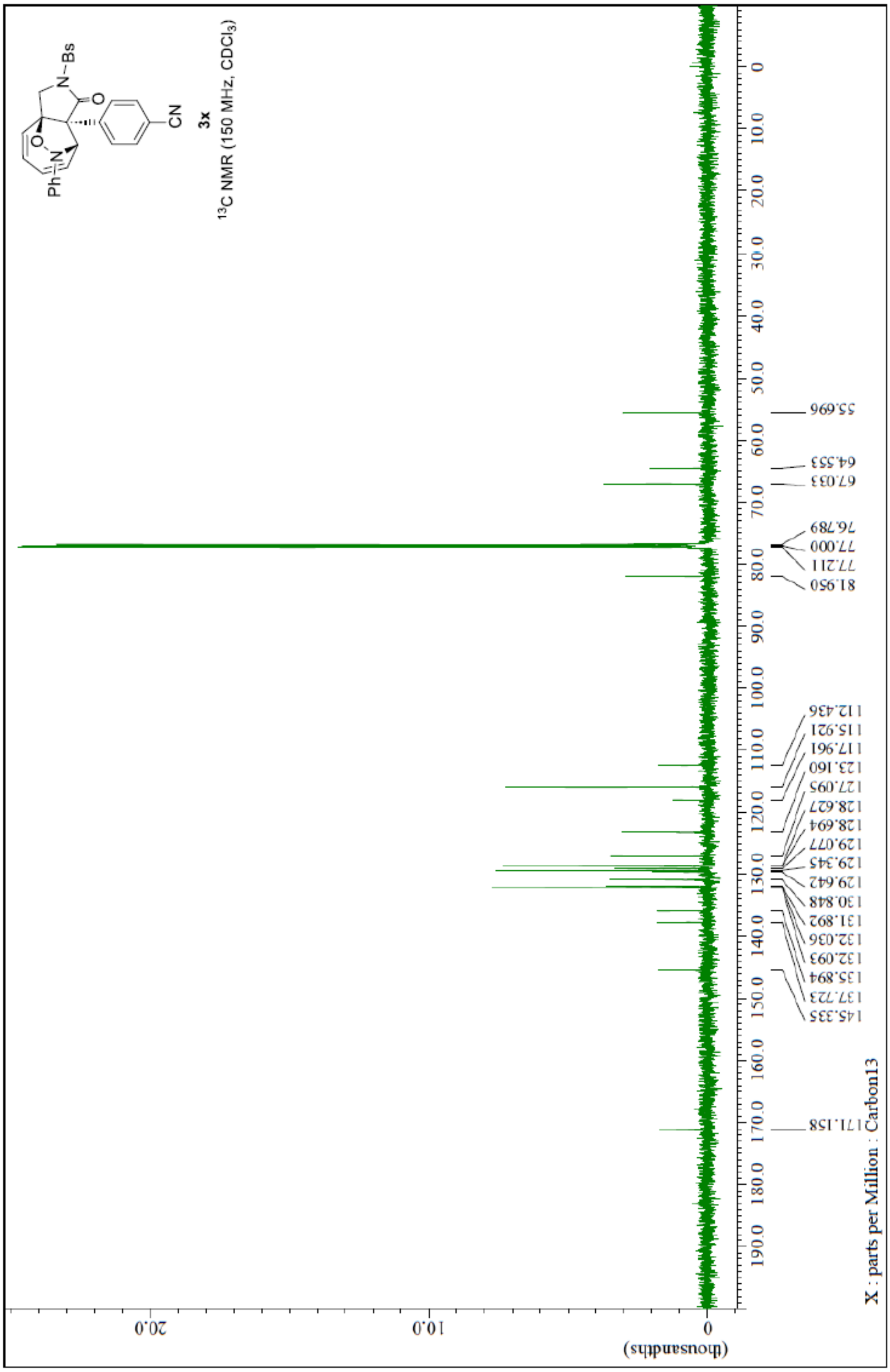

Ph
N–Bs
O
N
CN
3x
^{13}C NMR (150 MHz, CDCl$_3$)
55.696
64.553
67.033
76.789
77.000
77.211
81.950
112.436
115.921
117.961
123.160
127.095
128.627
128.694
129.077
129.345
129.642
130.848
131.892
132.036
132.093
135.894
137.723
145.335
171.158
X : parts per Million : Carbon13
(thousandths)

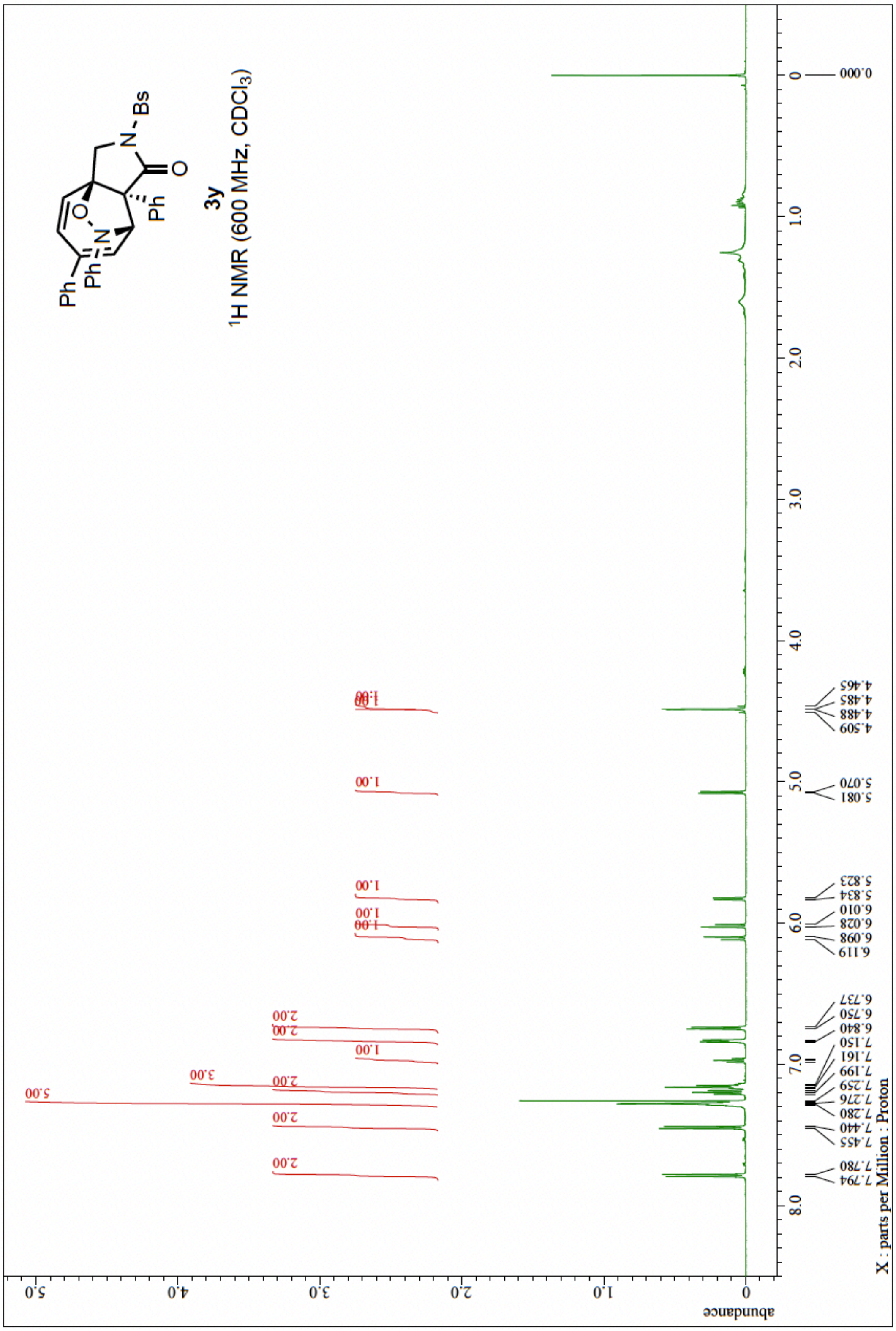

3y
1H NMR (600 MHz, CDCl3)
0.000
4.465
4.485
4.488
4.509
5.070
5.081
5.823
5.834
6.010
6.028
6.098
6.119
6.737
6.750
6.840
7.150
7.161
7.199
7.259
7.276
7.280
7.440
7.455
7.780
7.794
X : parts per Million : Proton
abundance

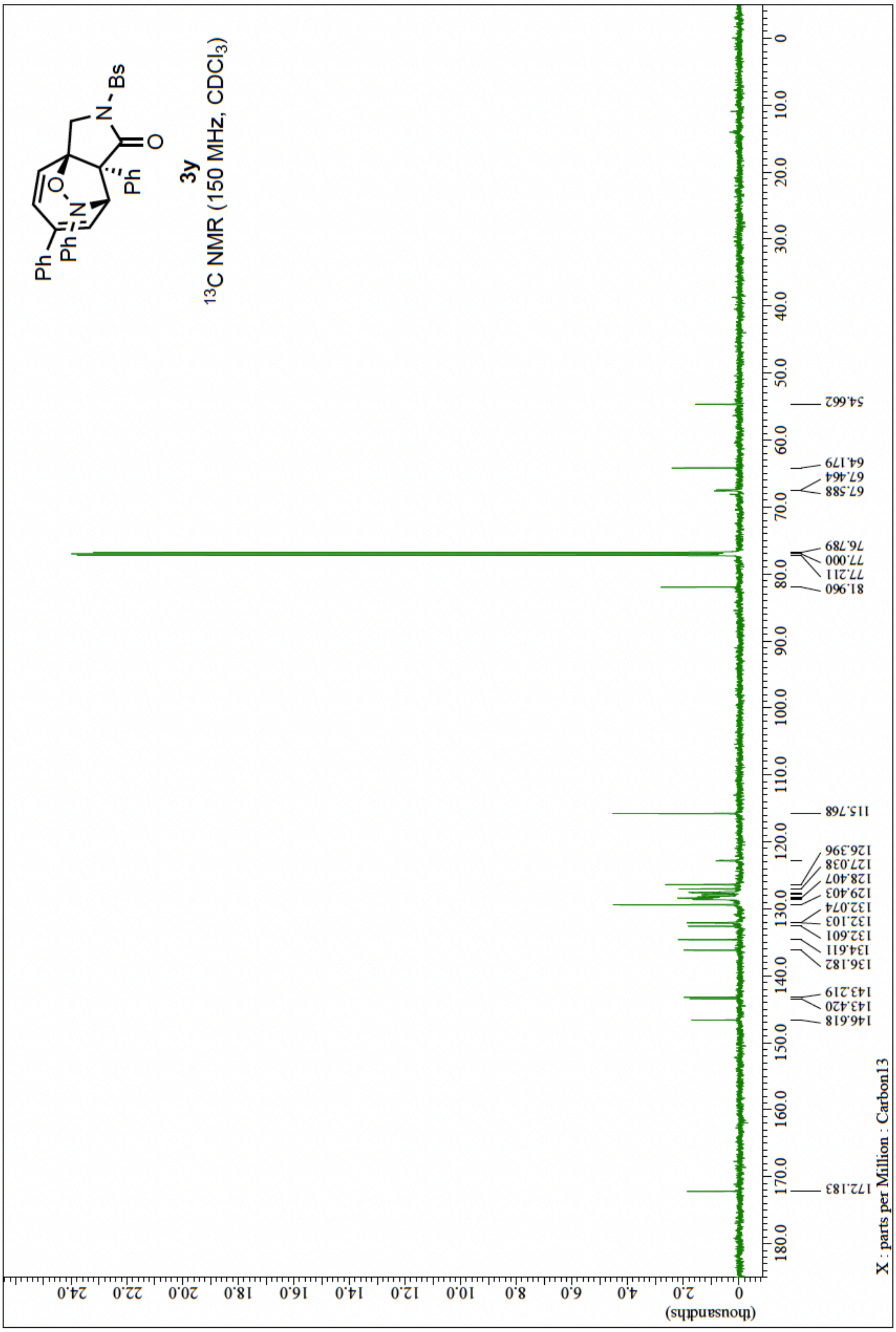

Ph
Ph
O
N
N
Bs
O
Ph
3y
^{13}C NMR (150 MHz, CDCl$_3$)
54.662
64.179
67.464
67.588
76.789
77.000
77.211
81.960
115.768
126.396
127.038
128.407
129.403
132.074
132.103
132.601
134.611
136.182
143.219
143.420
146.618
172.183
X : parts per Million : Carbon13
(thousandths)

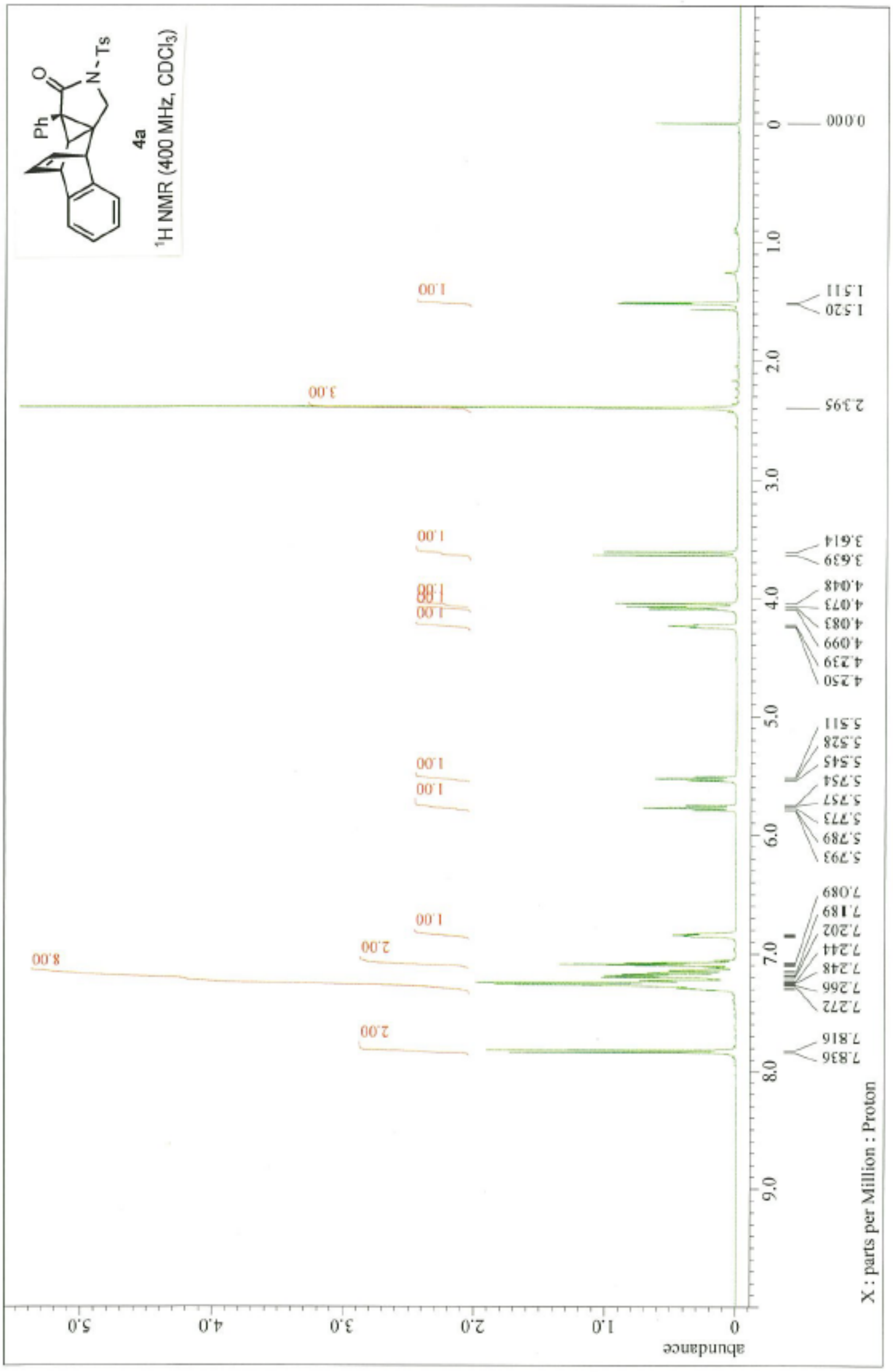

Ph
O
N-Ts
4a
¹H NMR (400 MHz, CDCl3)
X : parts per Million : Proton
abundance
0.000
1.511
1.520
2.395
3.614
3.639
4.048
4.073
4.083
4.099
4.239
4.250
5.511
5.528
5.545
5.754
5.757
5.773
5.789
5.793
7.089
7.189
7.202
7.244
7.248
7.266
7.272
7.816
7.836

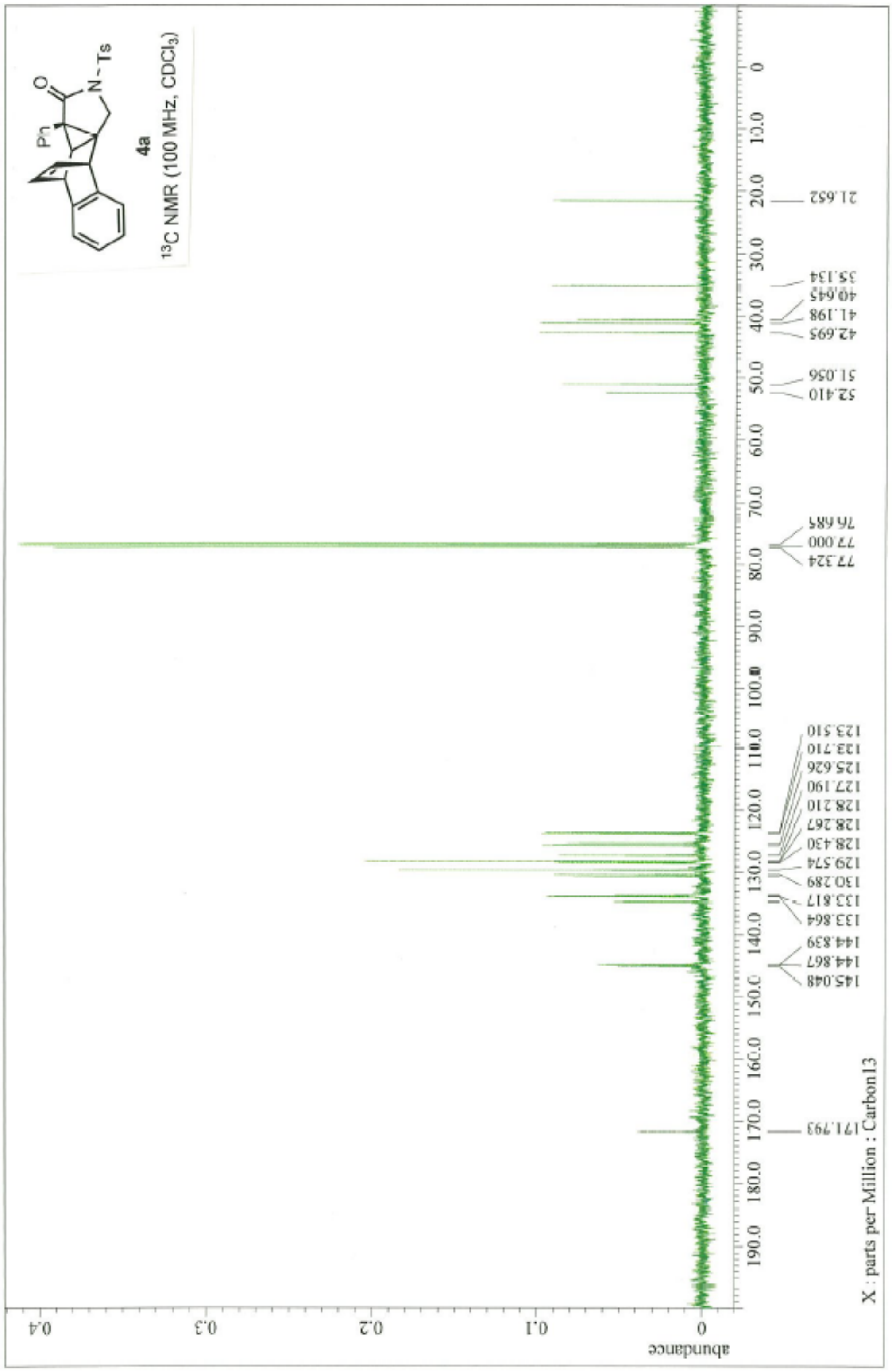
4a
^{13}C NMR (100 MHz, CDCl$_3$)
Ph
O
N~Ts
21.652
33.134
40.645
41.198
42.695
51.056
52.410
76.685
77.000
77.324
123.510
123.710
125.626
127.190
128.210
128.267
128.430
129.574
130.289
133.817
133.864
144.839
144.867
145.048
171.793
X : parts per Million : Carbon13
abundance

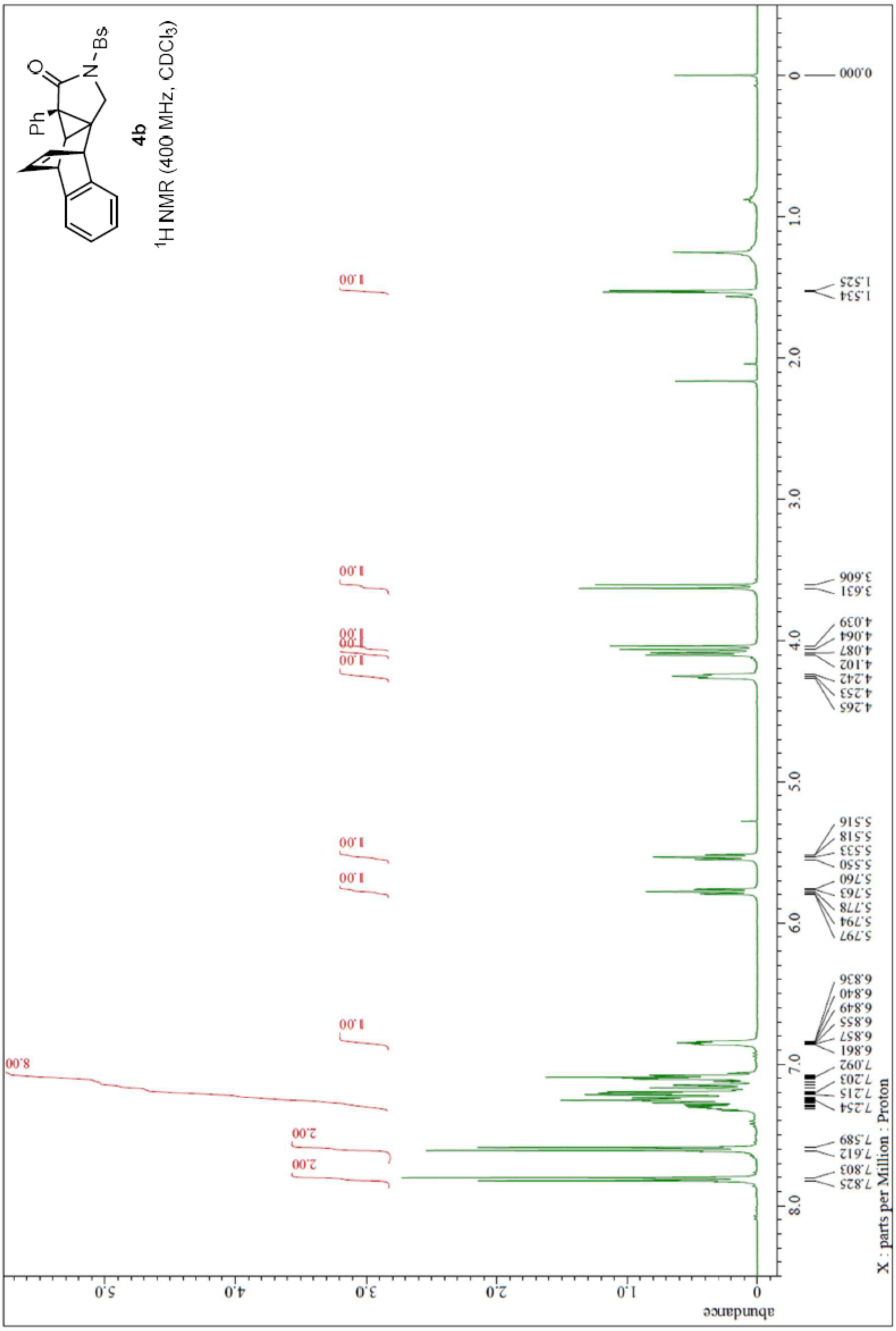
Ph
O
N−Bs
4b
1H NMR (400 MHz, CDCl3)
X : parts per Million : Proton
abundance

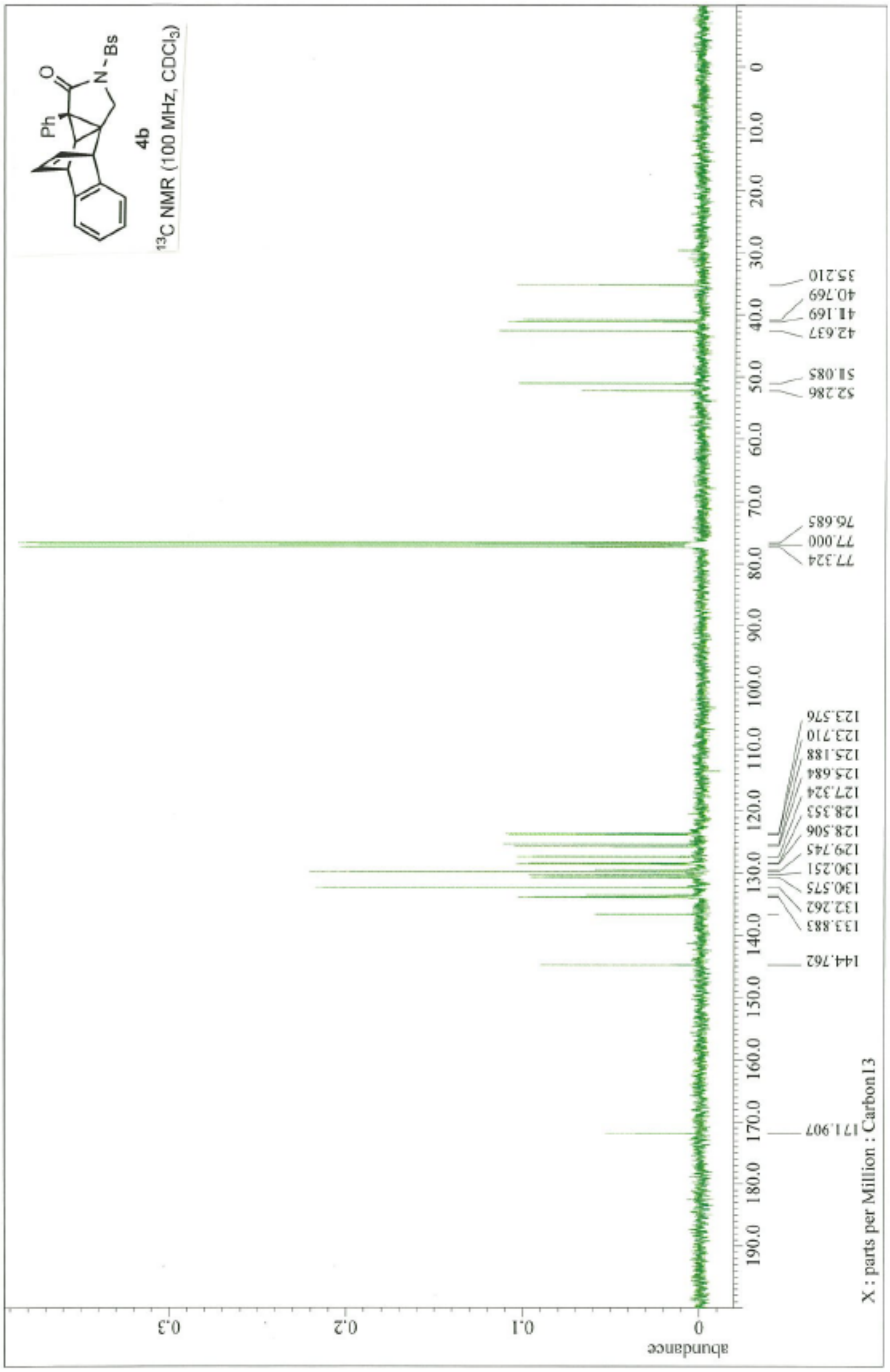

4b
¹³C NMR (100 MHz, CDCl3)
Ph
O
N
Bs
35.210
40.769
41.169
42.637
51.085
52.286
76.685
77.000
77.324
123.576
123.710
125.188
125.684
127.324
128.353
128.506
129.745
130.251
130.575
132.262
133.883
144.762
171.907
X : parts per Million : Carbon13
abundance

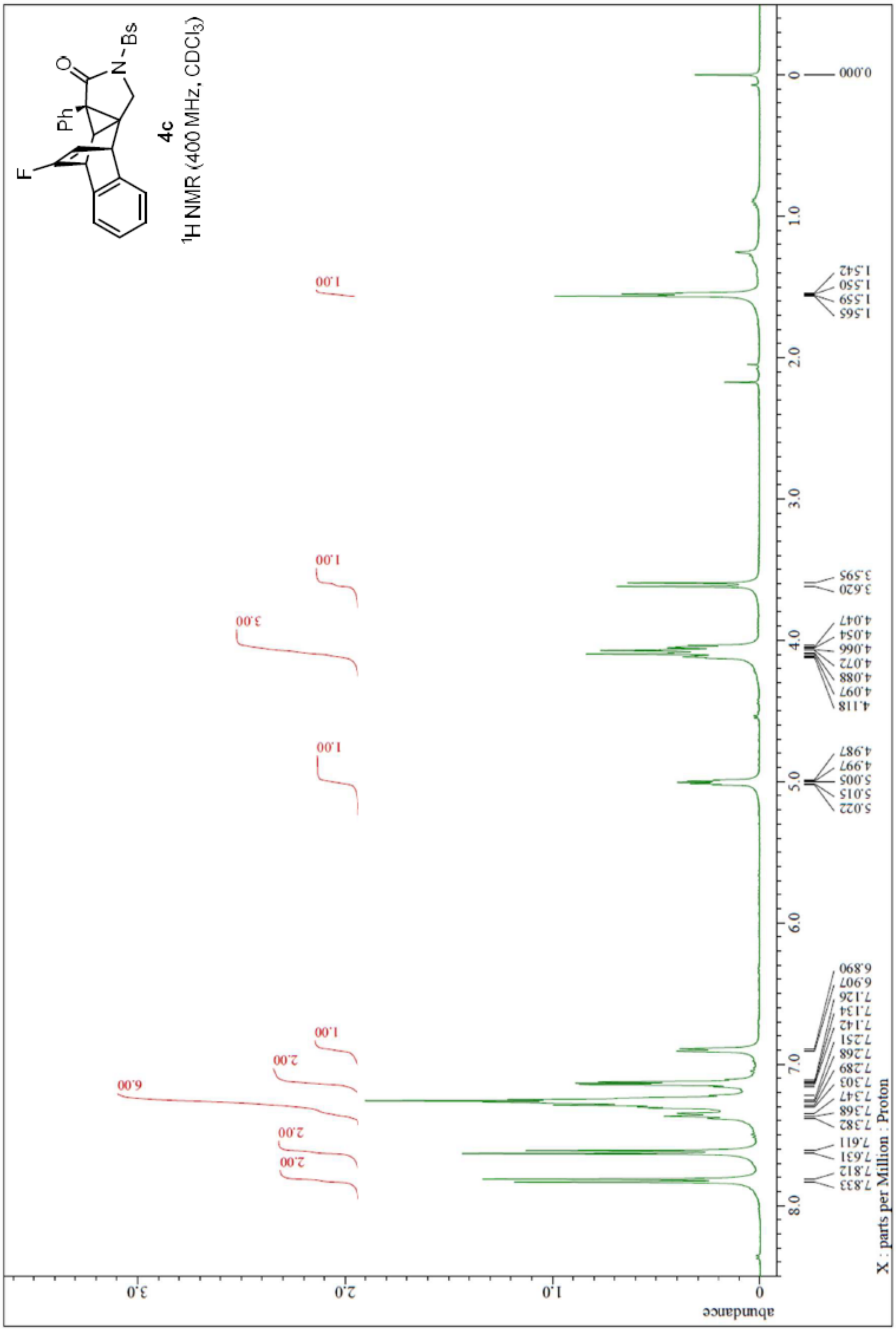
F
Ph
O
N-Bs
4c
1H NMR (400 MHz, CDCl3)
1.00
1.00
3.00
1.00
6.00
2.00
1.00
2.00
2.00
0.000
1.542
1.550
1.559
1.565
3.595
3.620
4.047
4.054
4.066
4.072
4.088
4.097
4.118
4.987
4.997
5.005
5.015
5.022
6.890
6.907
7.126
7.134
7.142
7.251
7.268
7.289
7.303
7.347
7.368
7.382
7.611
7.631
7.812
7.833
X : parts per Million : Proton
abundance

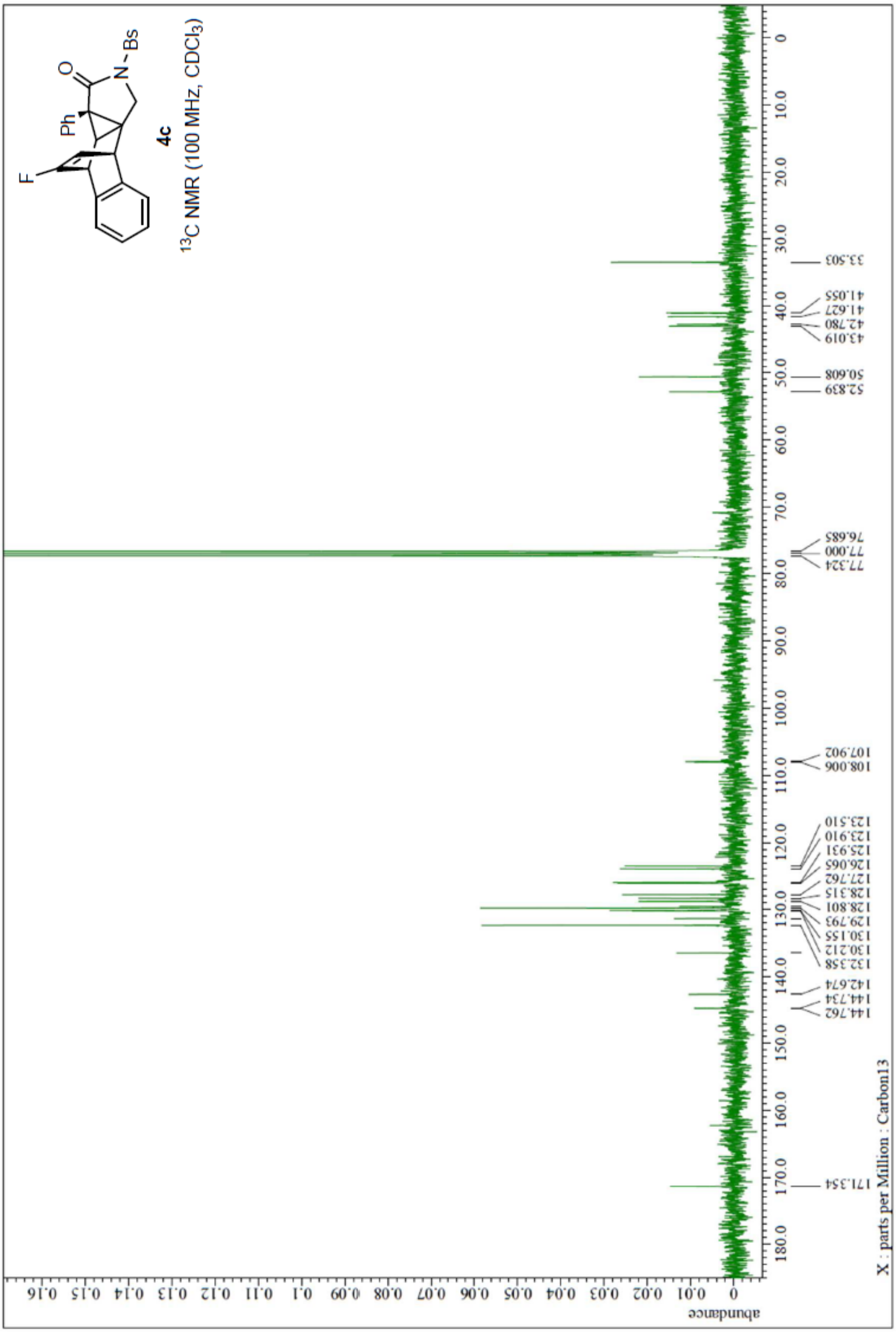

F
Ph
O
N
Bs
4c
13C NMR (100 MHz, CDCl3)
33.503
41.055
41.627
42.780
43.019
50.608
52.839
76.685
77.000
77.324
107.902
108.006
123.510
123.910
125.931
126.065
127.762
128.315
128.801
129.793
130.155
130.212
132.358
142.674
144.734
144.762
171.354
X : parts per Million : Carbon13
abundance

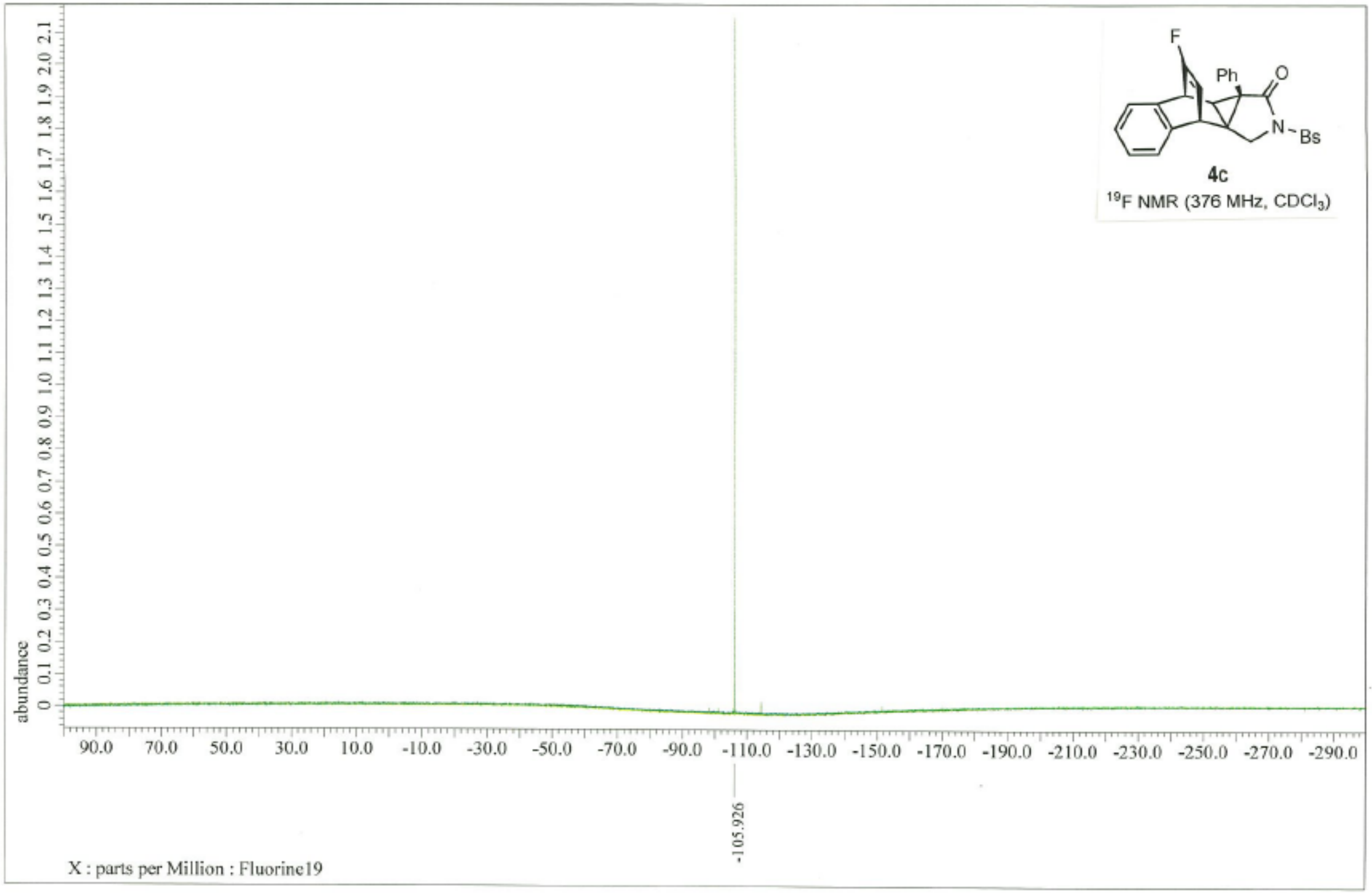
F
Ph
O
N~Bs
4c
^{19}F NMR (376 MHz, $CDCl_3$)
abundance
0 0.1 0.2 0.3 0.4 0.5 0.6 0.7 0.8 0.9 1.0 1.1 1.2 1.3 1.4 1.5 1.6 1.7 1.8 1.9 2.0 2.1
90.0 70.0 50.0 30.0 10.0 -10.0 -30.0 -50.0 -70.0 -90.0 -110.0 -130.0 -150.0 -170.0 -190.0 -210.0 -230.0 -250.0 -270.0 -290.0
-105.926
X : parts per Million : Fluorine19

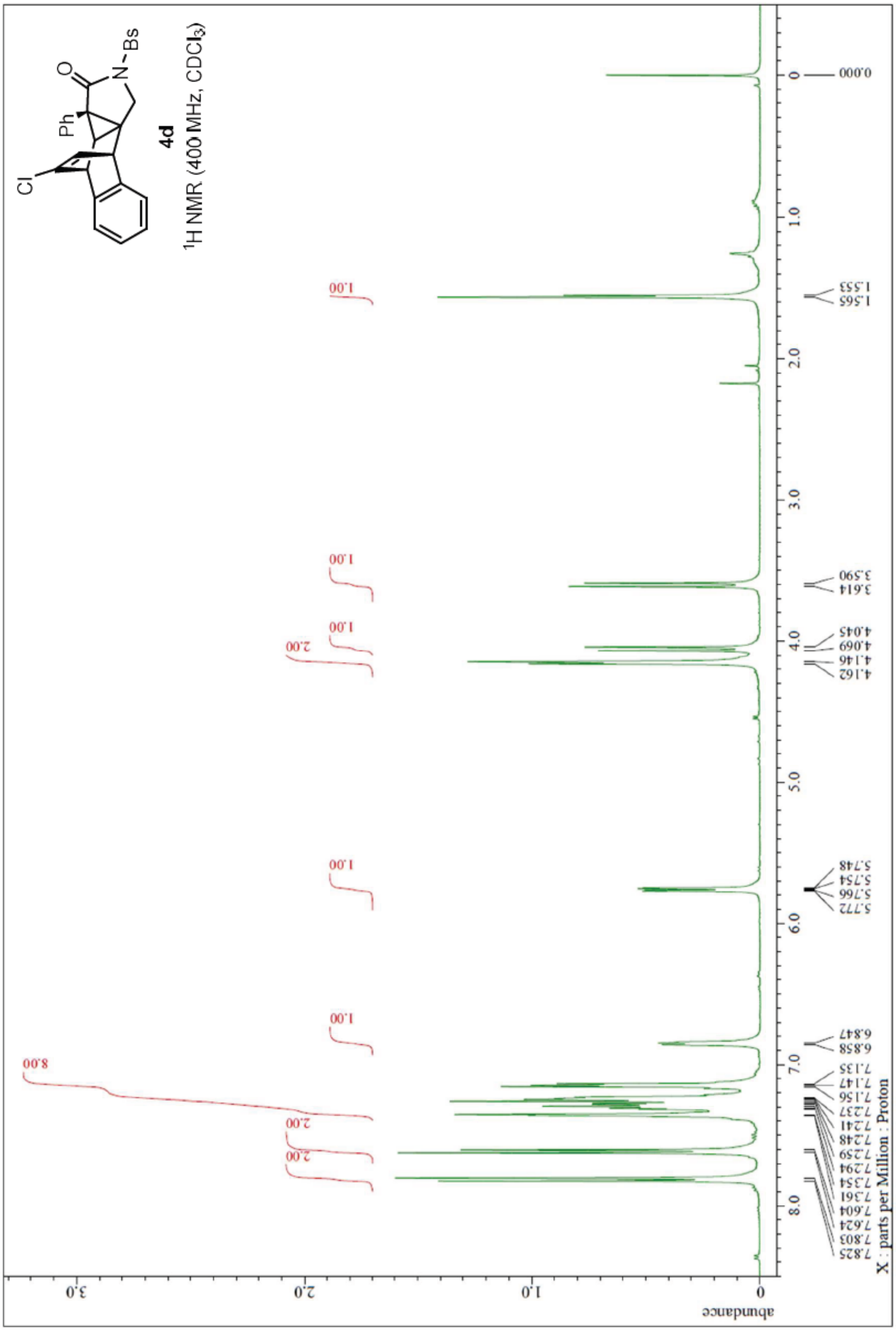
4d
1H NMR (400 MHz, CDCl3)
X : parts per Million : Proton
abundance

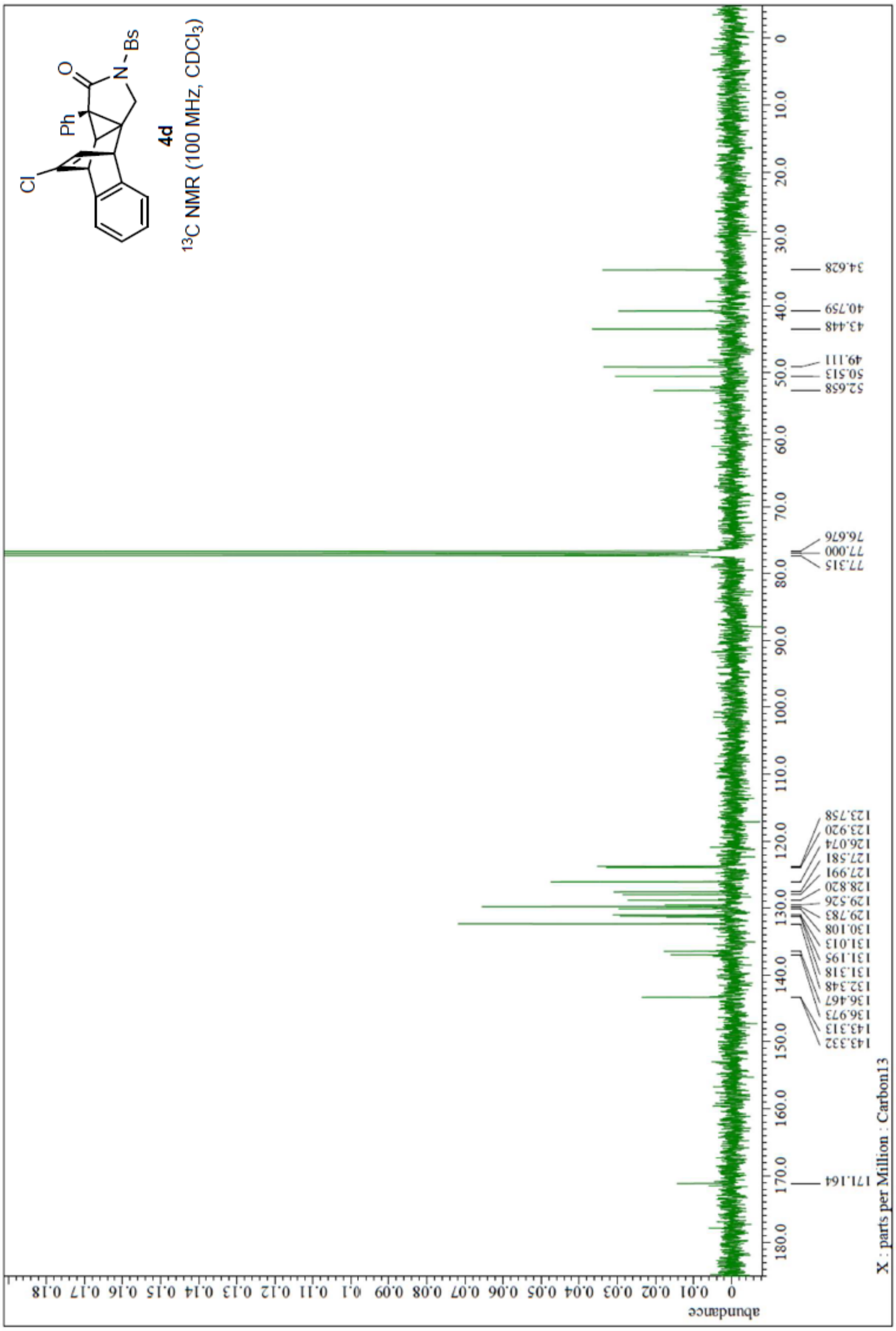
Cl
Ph
O
N-Bs
4d
13C NMR (100 MHz, CDCl3)
171.164
143.332
143.313
136.973
136.467
132.348
131.318
131.195
131.013
130.108
129.783
129.526
128.820
127.991
127.581
126.074
123.920
123.758
77.315
77.000
76.676
52.658
50.513
49.111
43.448
40.759
34.628
X : parts per Million : Carbon13
abundance

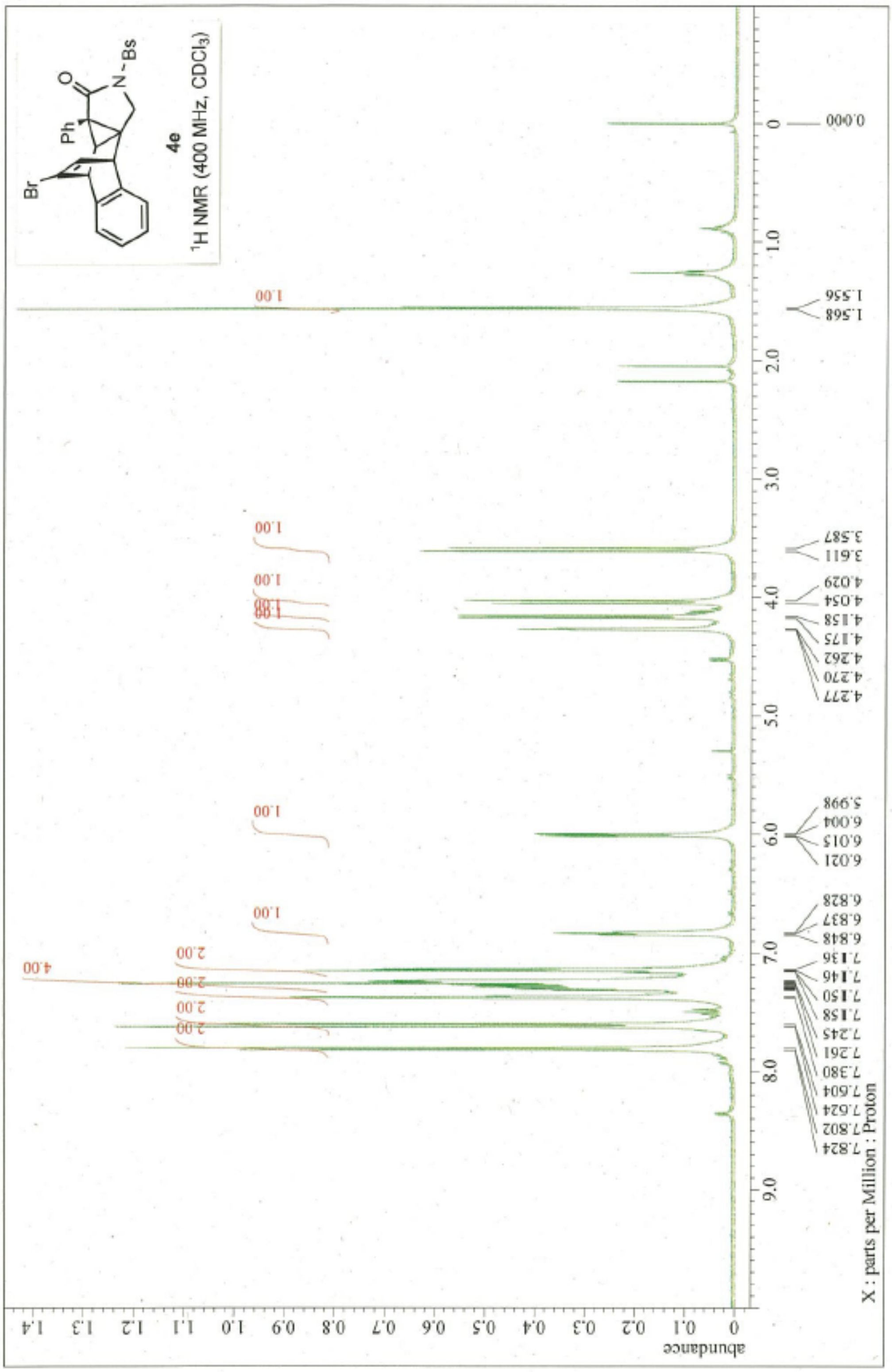

Br
Ph
O
N~Bs
4e
1H NMR (400 MHz, CDCl3)
0.000
1.556
1.568
3.587
3.611
4.029
4.054
4.158
4.175
4.262
4.270
4.277
5.998
6.004
6.015
6.021
6.828
6.837
6.848
7.136
7.146
7.150
7.158
7.245
7.261
7.380
7.604
7.624
7.802
7.824
1.00
1.00
1.00
1.00
1.00
1.00
1.00
2.00
2.00
2.00
2.00
4.00
X : parts per Million : Proton
abundance

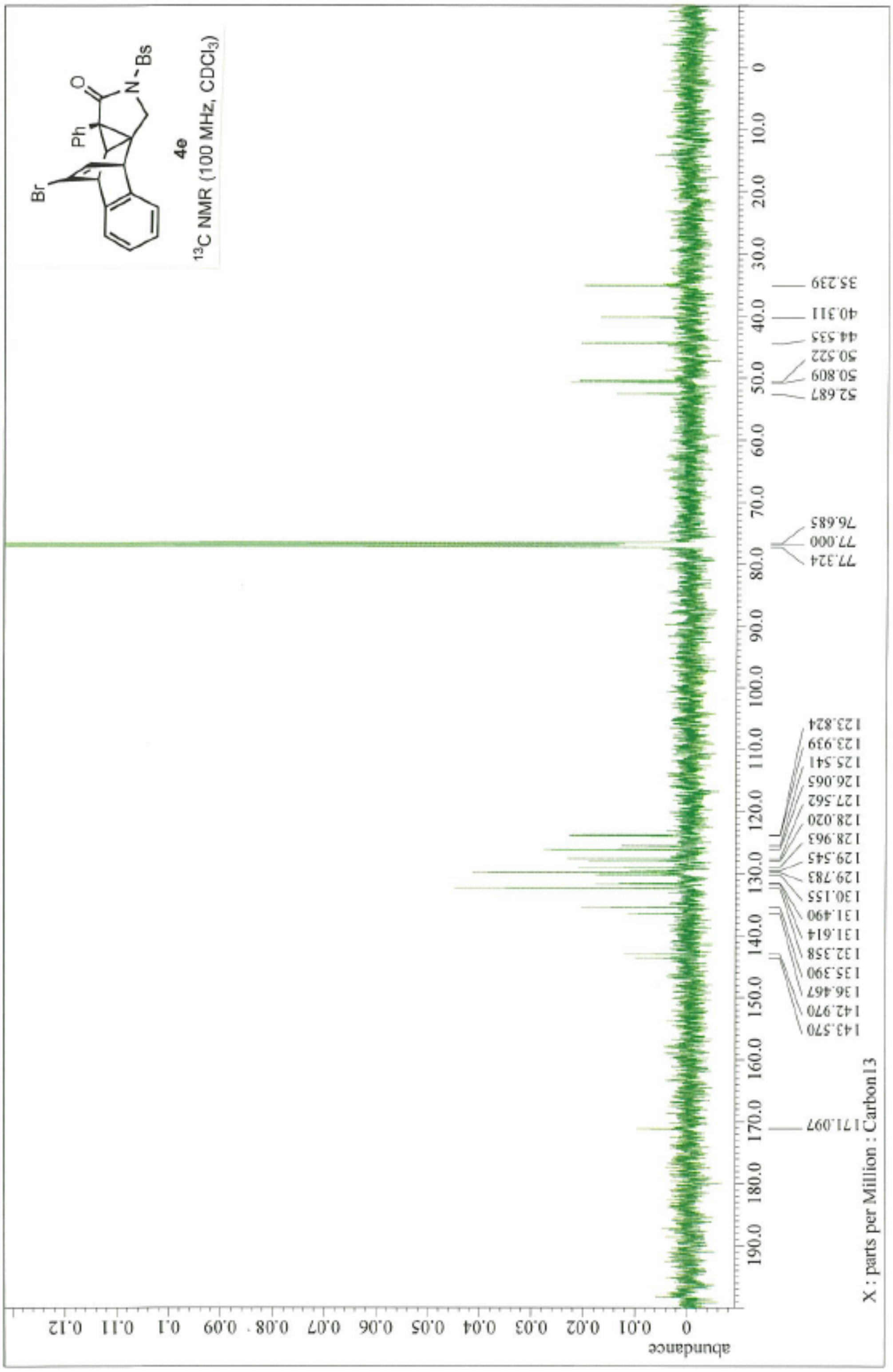
Br
Ph
O
N~Bs
4e
^{13}C NMR (100 MHz, CDCl$_3$)
35.239
40.311
44.535
50.522
50.809
52.687
76.685
77.000
77.324
123.824
123.939
125.541
126.065
127.562
128.020
128.963
129.545
129.783
130.155
131.490
131.614
132.358
135.390
136.467
142.970
143.570
171.097
X : parts per Million : Carbon13
abundance

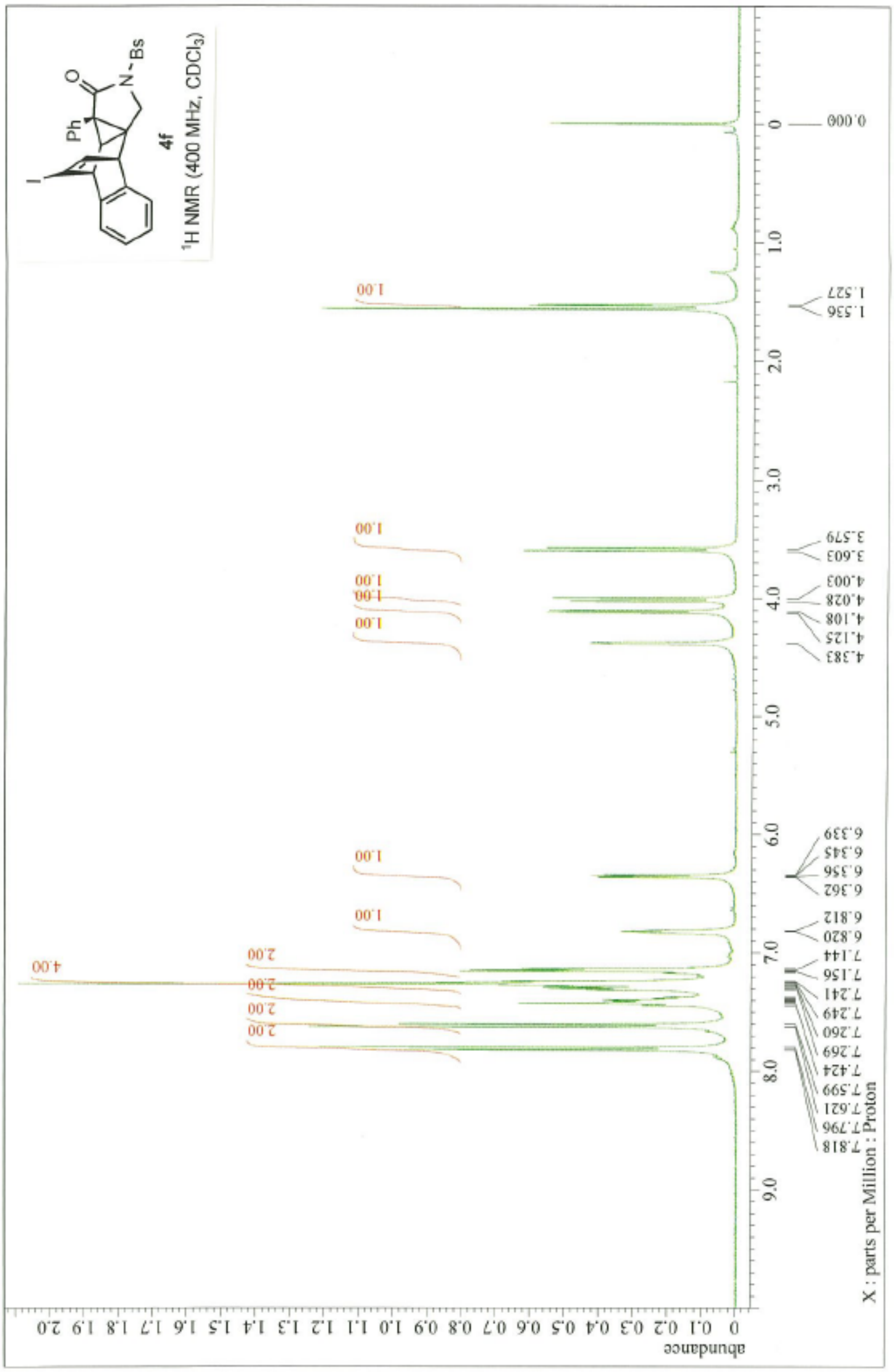

Ph
O
N~Bs
I
4f
1H NMR (400 MHz, CDCl3)
0.000
1.527
1.536
3.579
3.603
4.003
4.028
4.108
4.125
4.383
6.339
6.345
6.356
6.362
6.812
6.820
7.144
7.156
7.241
7.249
7.260
7.269
7.424
7.599
7.621
7.796
7.818
X : parts per Million : Proton
abundance

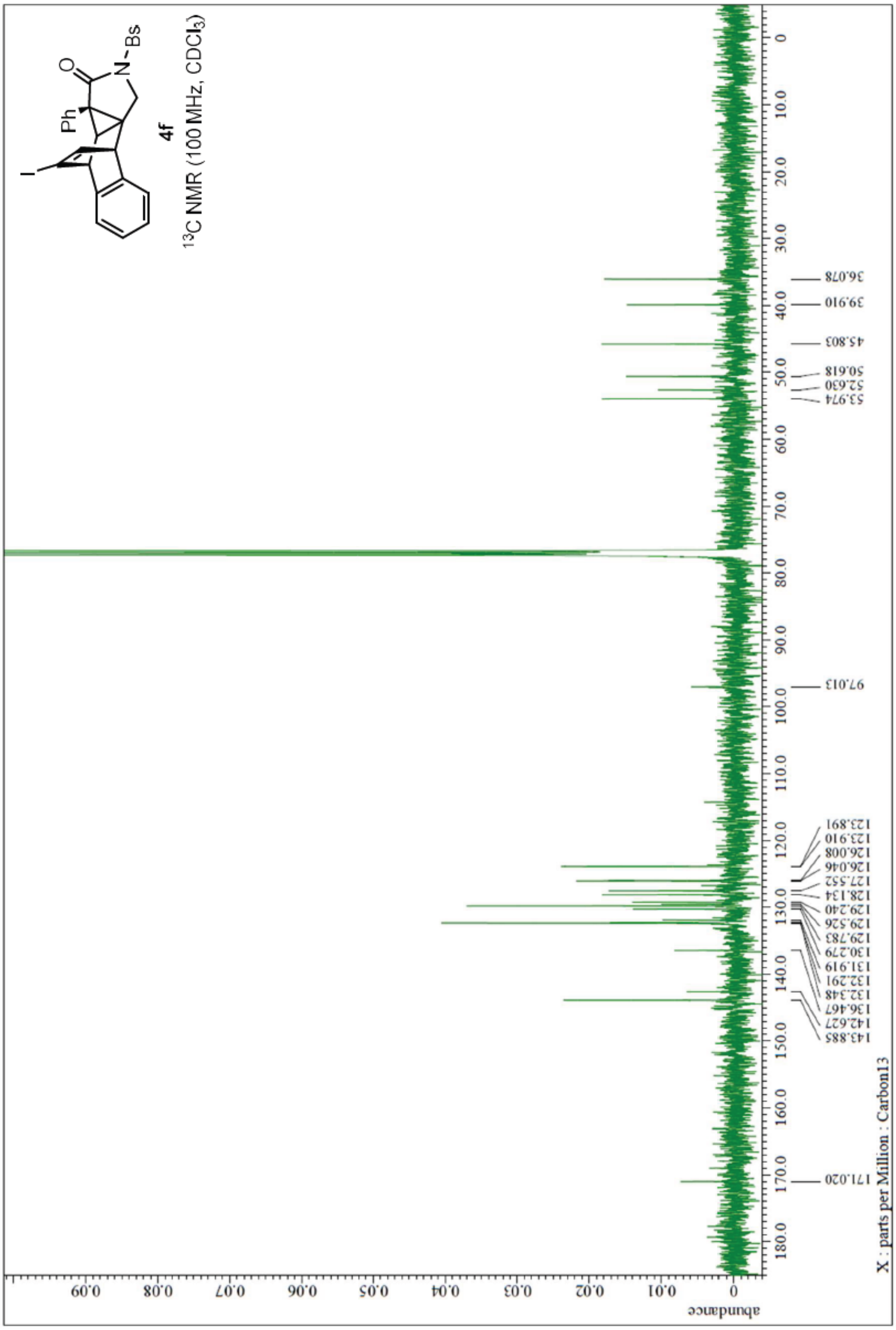

4f
13C NMR (100 MHz, CDCl3)
Ph
O
N
Bs
I
171.020
143.885
142.627
136.467
132.348
132.291
131.919
130.279
129.783
129.526
129.240
128.134
127.552
126.046
126.008
123.910
123.891
97.013
53.974
52.630
50.618
45.803
39.910
36.078
X : parts per Million : Carbon13
abundance

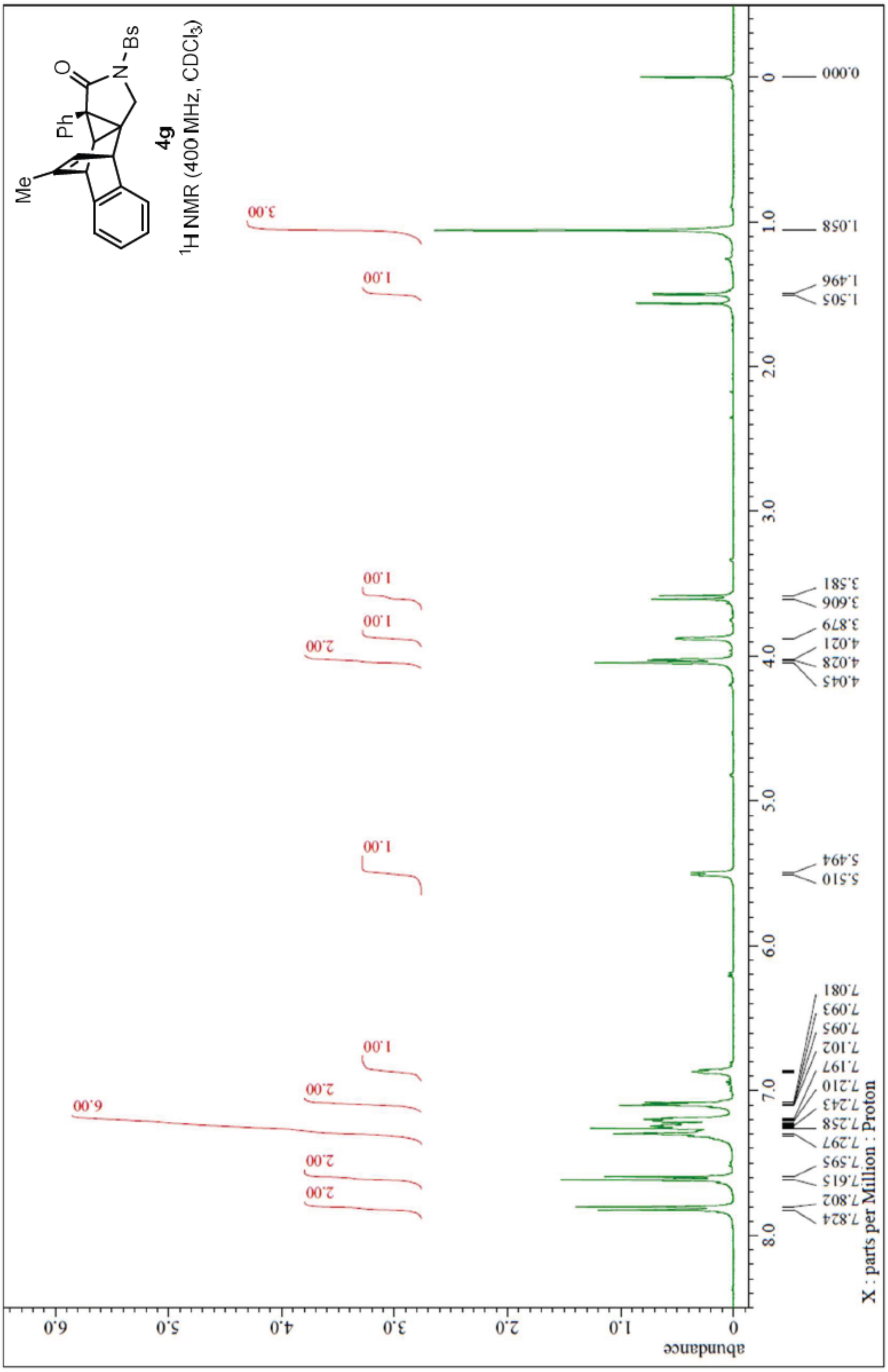
Me
Ph
O
N-Bs
4g
1H NMR (400 MHz, CDCl3)
X : parts per Million : Proton
abundance

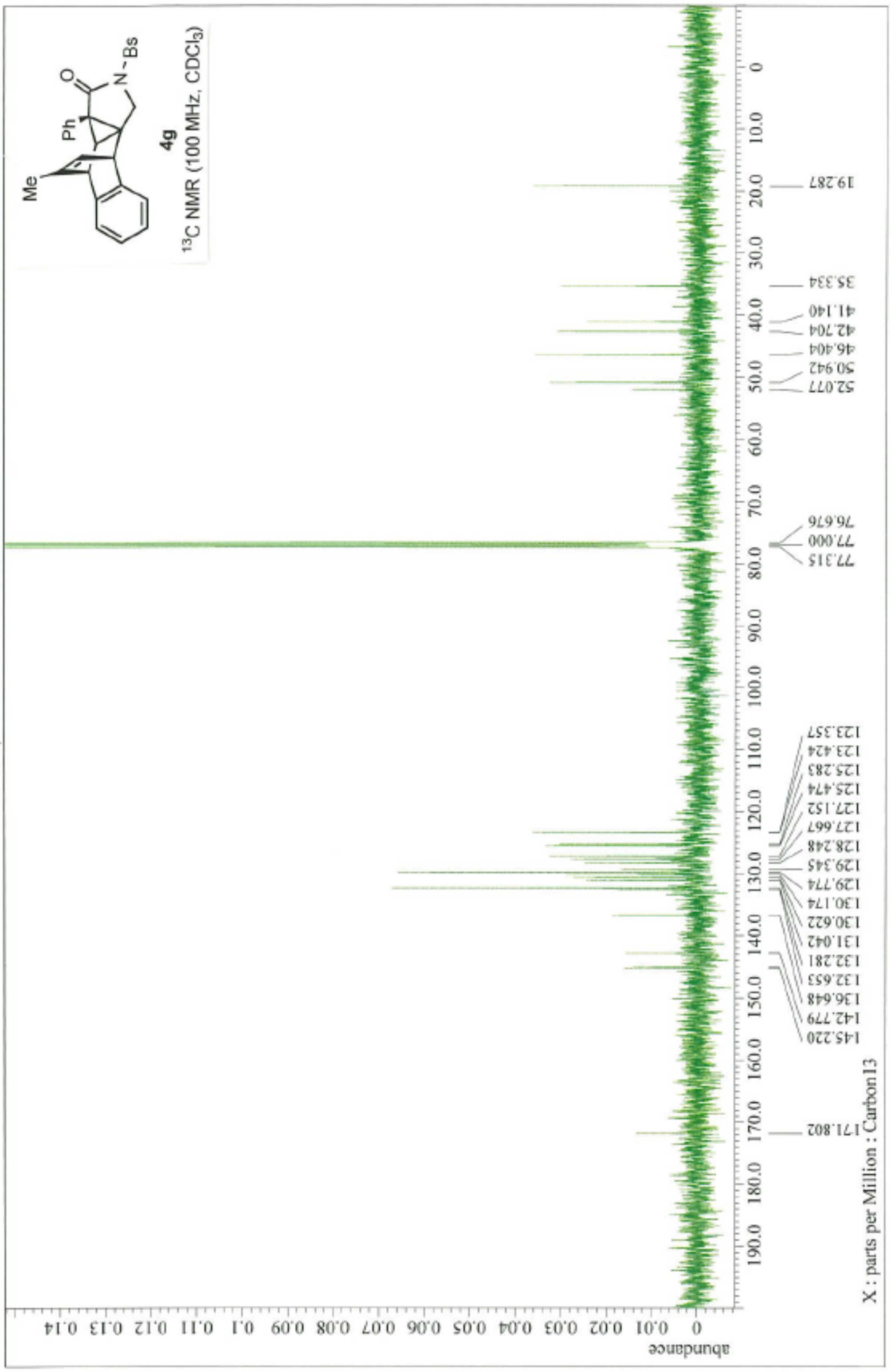

Me
Ph
O
N~Bs
4g
13C NMR (100 MHz, CDCl3)
19.287
35.334
41.140
42.704
46.404
50.942
52.077
76.676
77.000
77.315
123.357
123.424
125.283
125.474
127.152
127.667
128.248
129.345
129.774
130.174
130.622
131.042
132.281
132.653
136.648
142.779
145.220
171.802
X : parts per Million : Carbon13
abundance

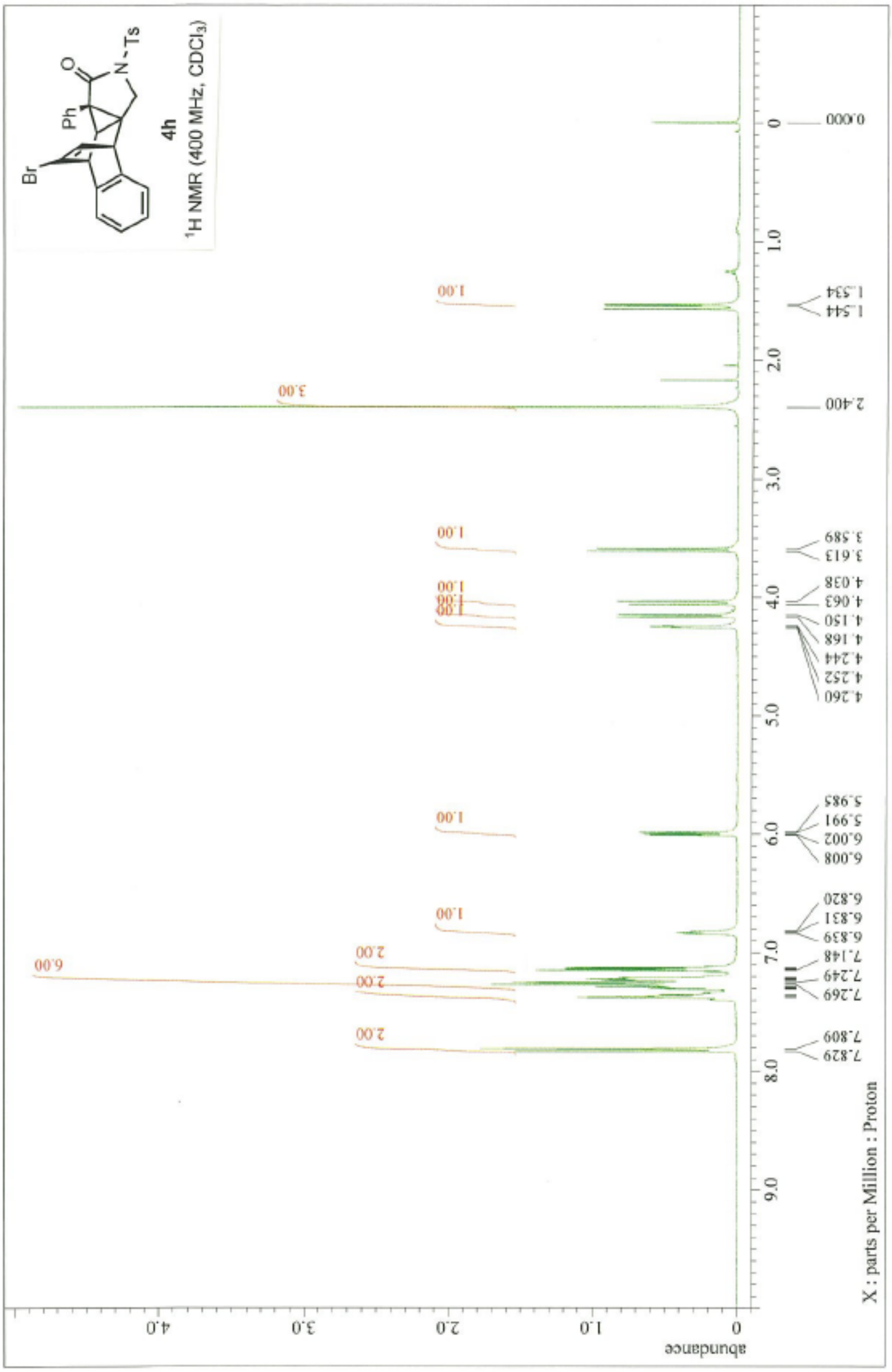

Br
Ph
O
N~Ts
4h
1H NMR (400 MHz, CDCl3)
0.000
1.534
1.544
2.400
3.589
3.613
4.038
4.063
4.150
4.168
4.244
4.252
4.260
5.985
5.991
6.002
6.008
6.820
6.831
6.839
7.148
7.249
7.269
7.809
7.829
1.00
3.00
1.00
1.00
1.00
1.00
1.00
1.00
2.00
6.00
2.00
2.00
X : parts per Million : Proton
abundance

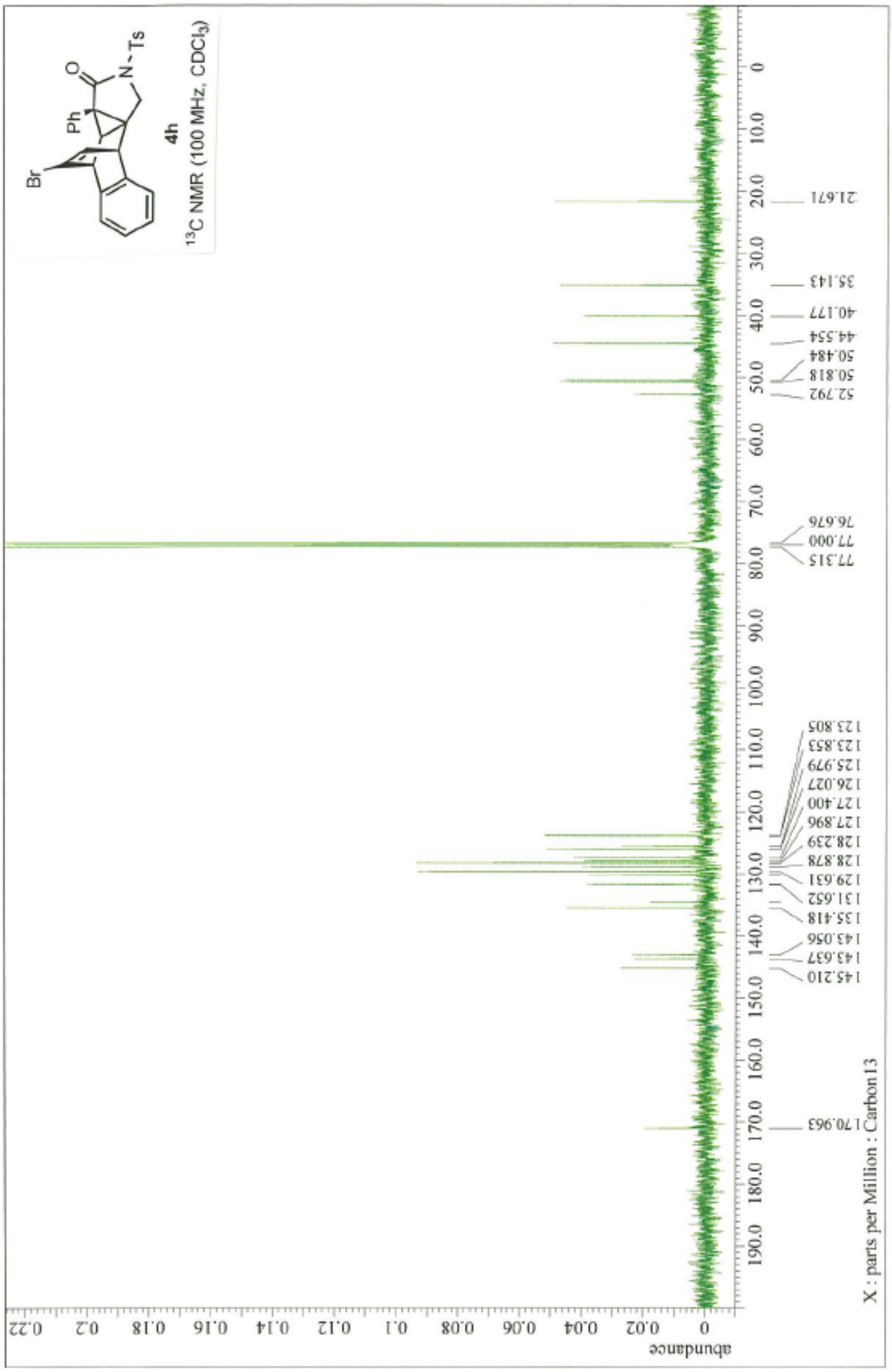

4h
13C NMR (100 MHz, CDCl3)
Br
Ph
O
N~Ts
21.671
35.143
40.177
44.554
50.484
50.818
52.792
76.676
77.000
77.315
123.805
123.853
125.979
126.027
127.400
127.896
128.239
128.878
129.631
131.652
135.418
143.056
143.637
145.210
170.963
X : parts per Million : Carbon13
abundance

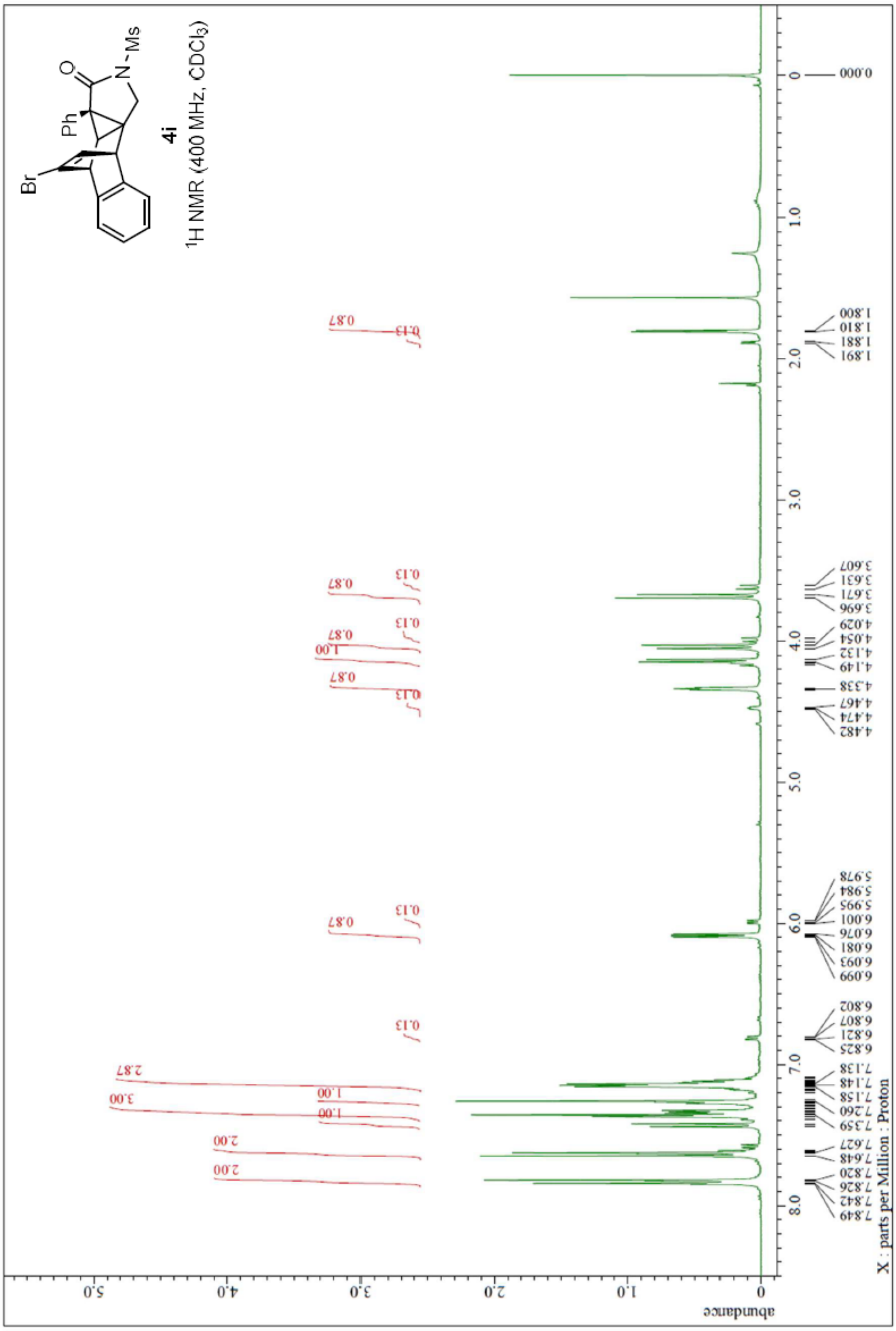

Br
Ph
O
N-Ms
4i
1H NMR (400 MHz, CDCl3)
X : parts per Million : Proton
abundance

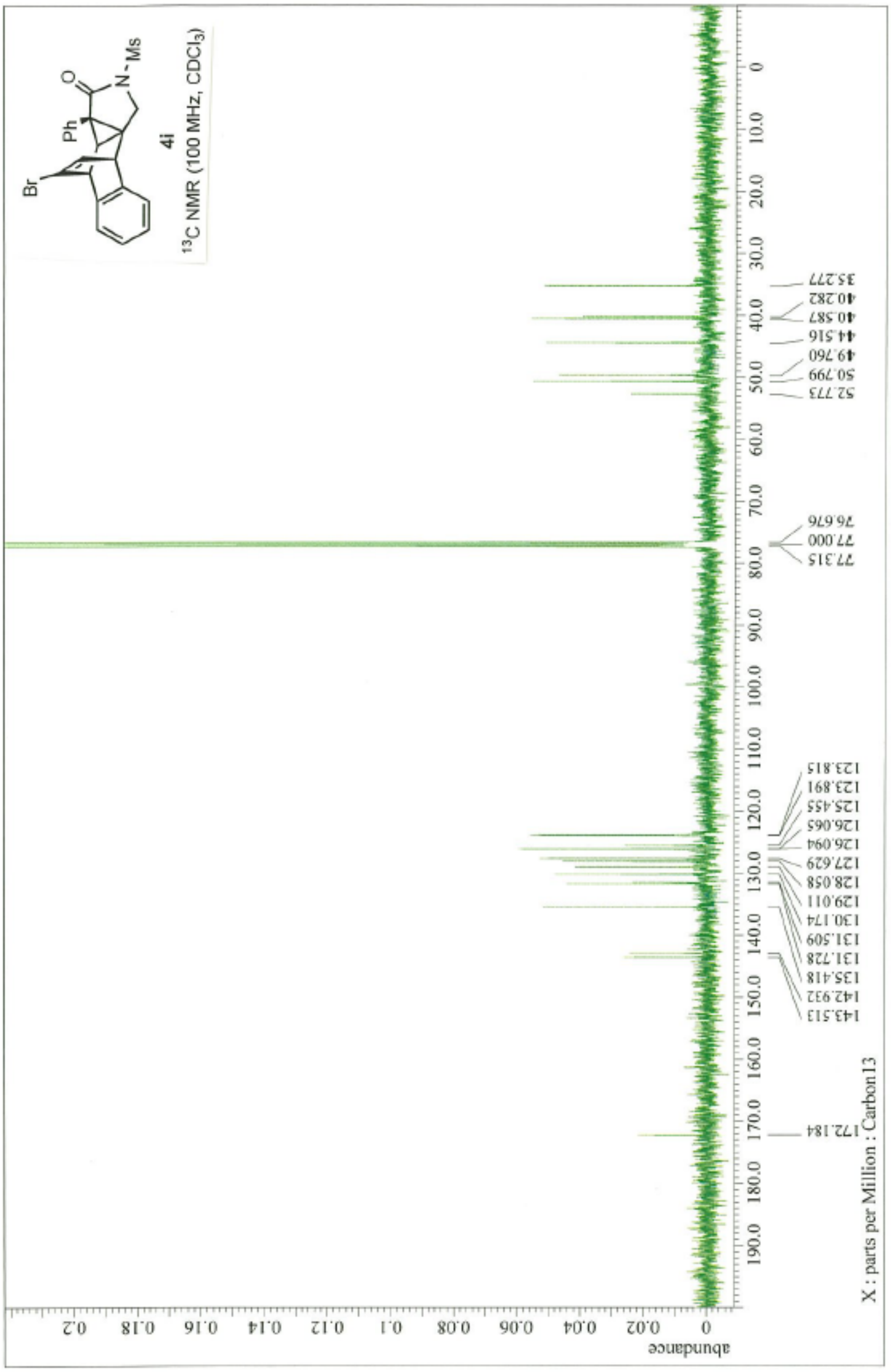
4i
^{13}C NMR (100 MHz, CDCl$_3$)
Br
Ph
O
N-Ms
35.277
40.282
40.587
44.516
49.760
50.799
52.773
76.676
77.000
77.315
123.815
123.891
125.455
126.065
126.094
127.629
128.058
129.011
130.174
131.509
131.728
135.418
142.932
143.513
172.184
X : parts per Million : Carbon13
abundance

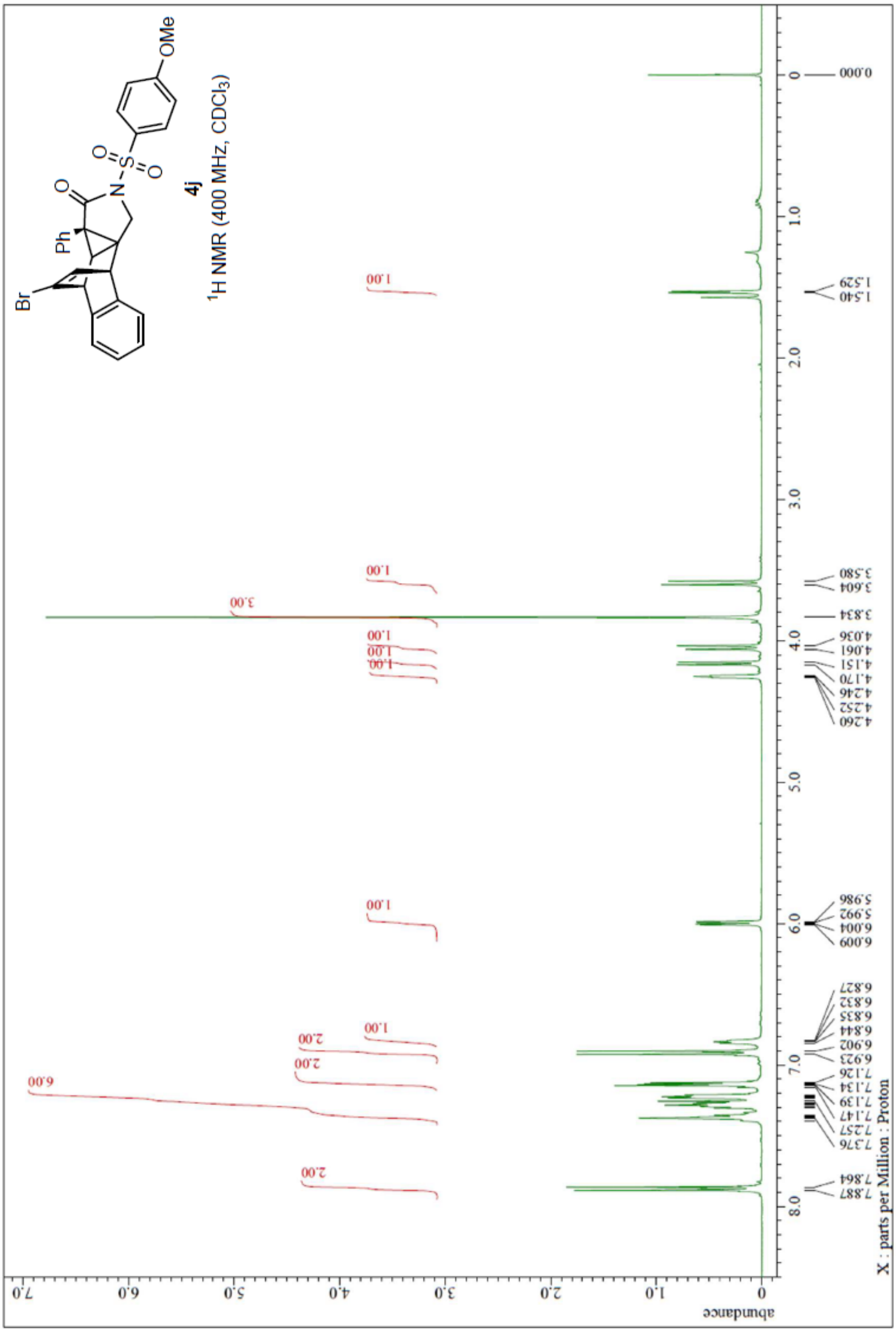
4j
^{1}H NMR (400 MHz, CDCl$_3$)
X : parts per Million : Proton
abundance

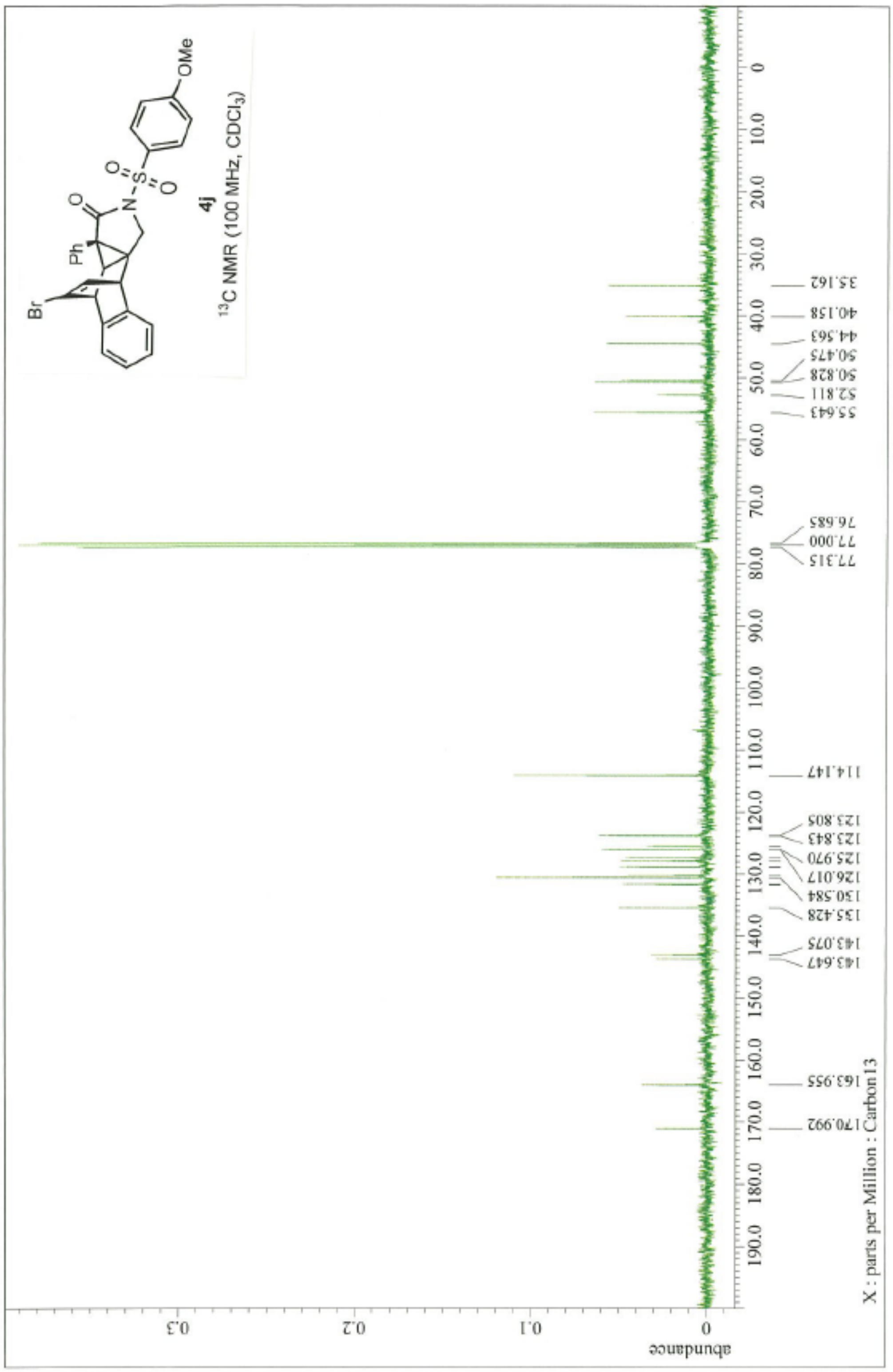
Br
Ph
O
N
S
O
O
OMe
4j
^{13}C NMR (100 MHz, CDCl$_3$)
35.162
40.158
44.563
50.475
50.828
52.811
55.643
76.685
77.000
77.315
114.147
123.805
123.843
125.970
126.017
130.584
135.428
143.075
143.647
163.955
170.992
X : parts per Million : Carbon13
abundance

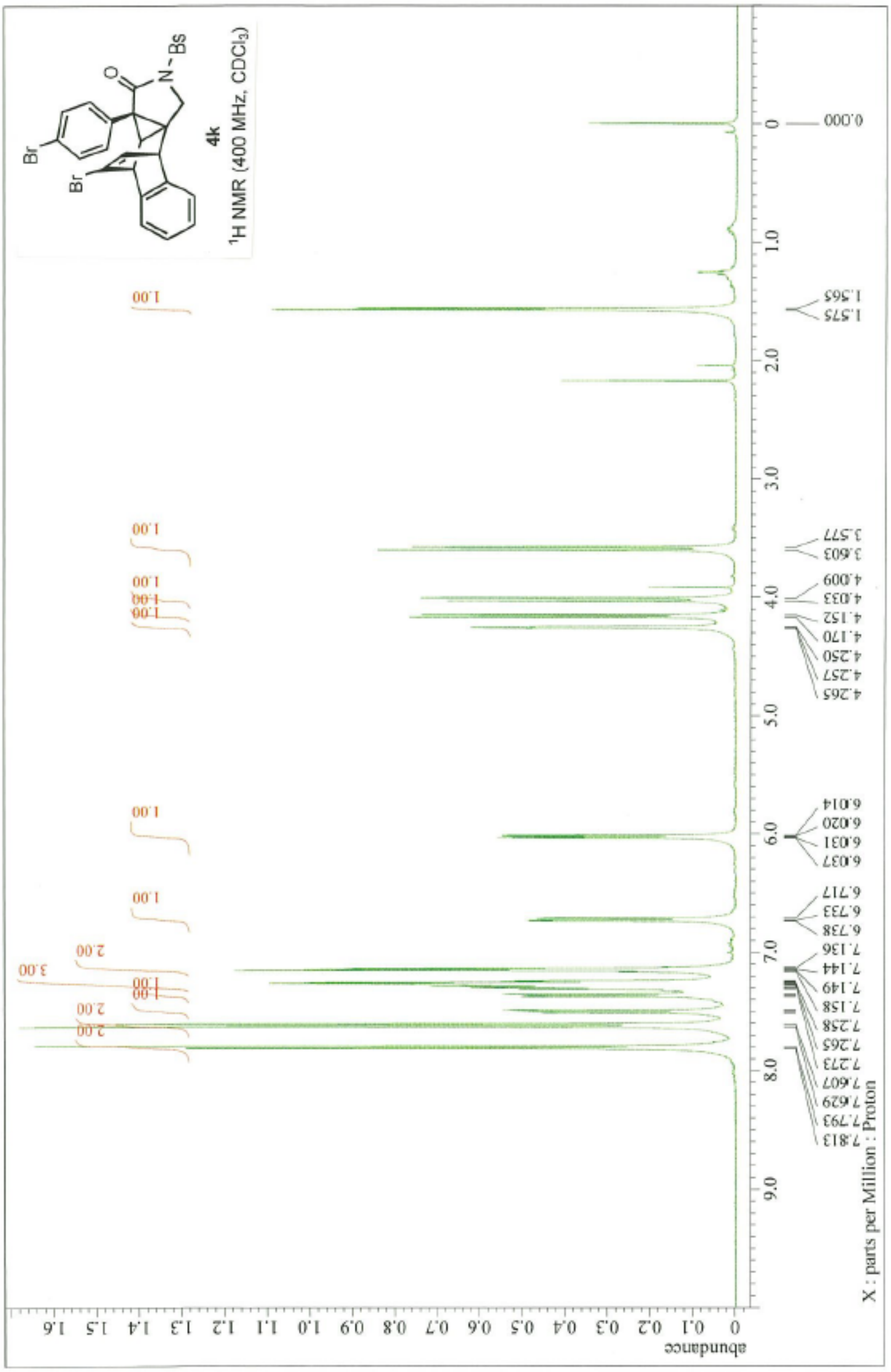
4k
1H NMR (400 MHz, CDCl3)
X : parts per Million : Proton
abundance

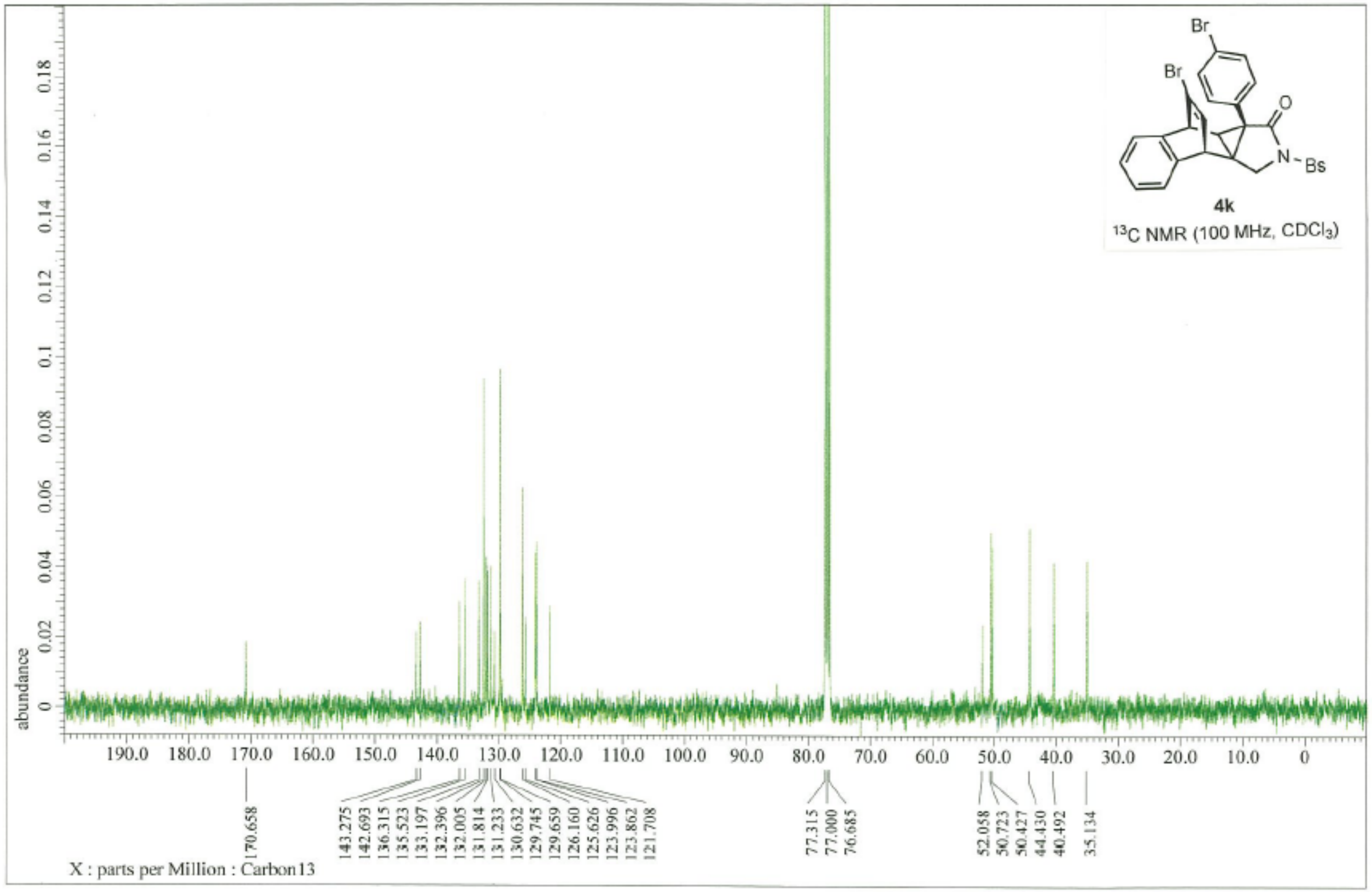
Br
Br
O
N~Bs
4k
13C NMR (100 MHz, CDCl3)
abundance
0.18 0.16 0.14 0.12 0.1 0.08 0.06 0.04 0.02 0
190.0 180.0 170.0 160.0 150.0 140.0 130.0 120.0 110.0 100.0 90.0 80.0 70.0 60.0 50.0 40.0 30.0 20.0 10.0 0
170.658
143.275
142.693
136.315
135.523
133.197
132.396
132.005
131.814
131.233
130.632
129.745
129.659
126.160
125.626
123.996
123.862
121.708
77.315
77.000
76.685
52.058
50.723
50.427
44.430
40.492
35.134
X : parts per Million : Carbon13

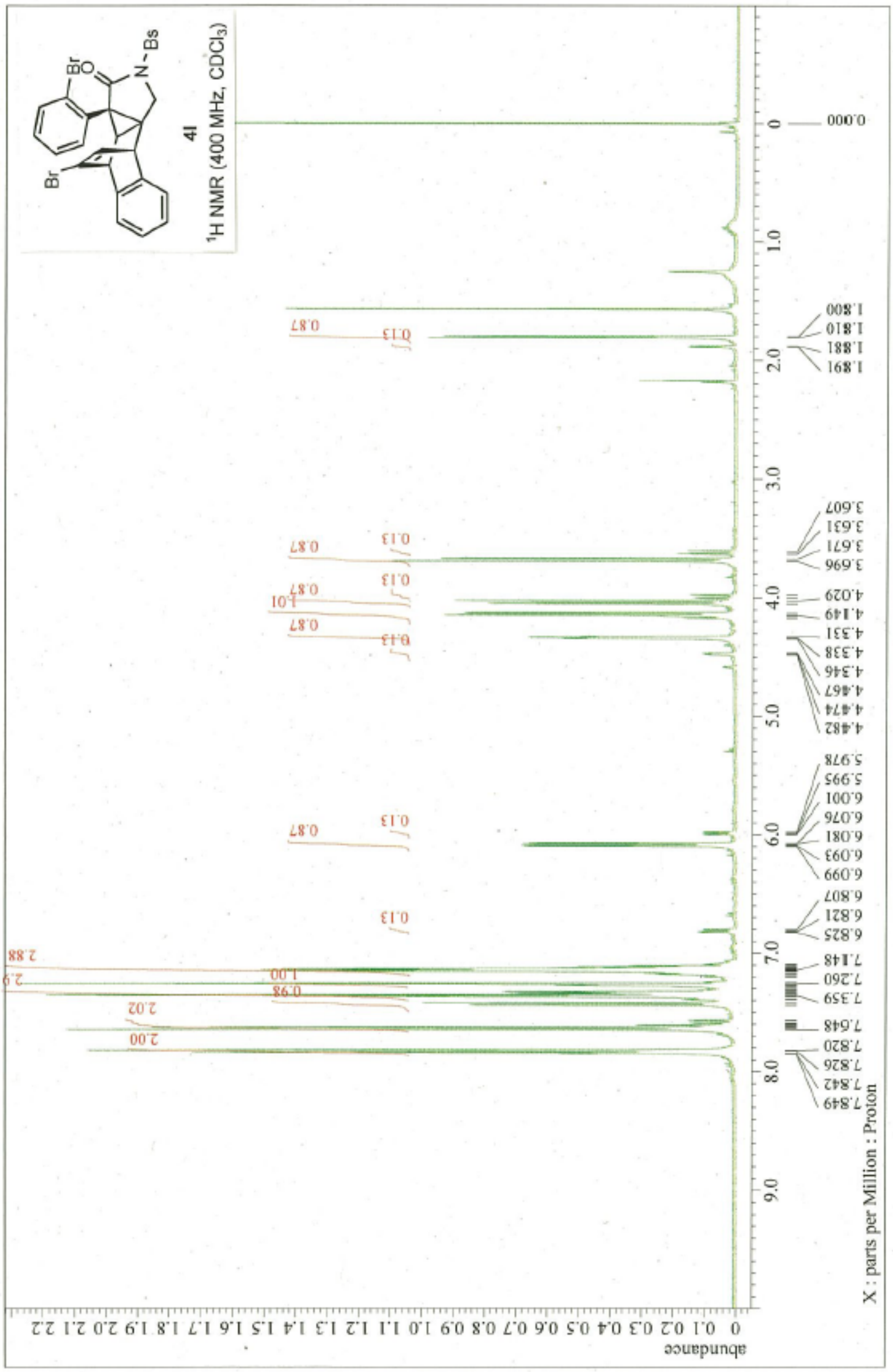
4l
1H NMR (400 MHz, CDCl3)
X : parts per Million : Proton
abundance

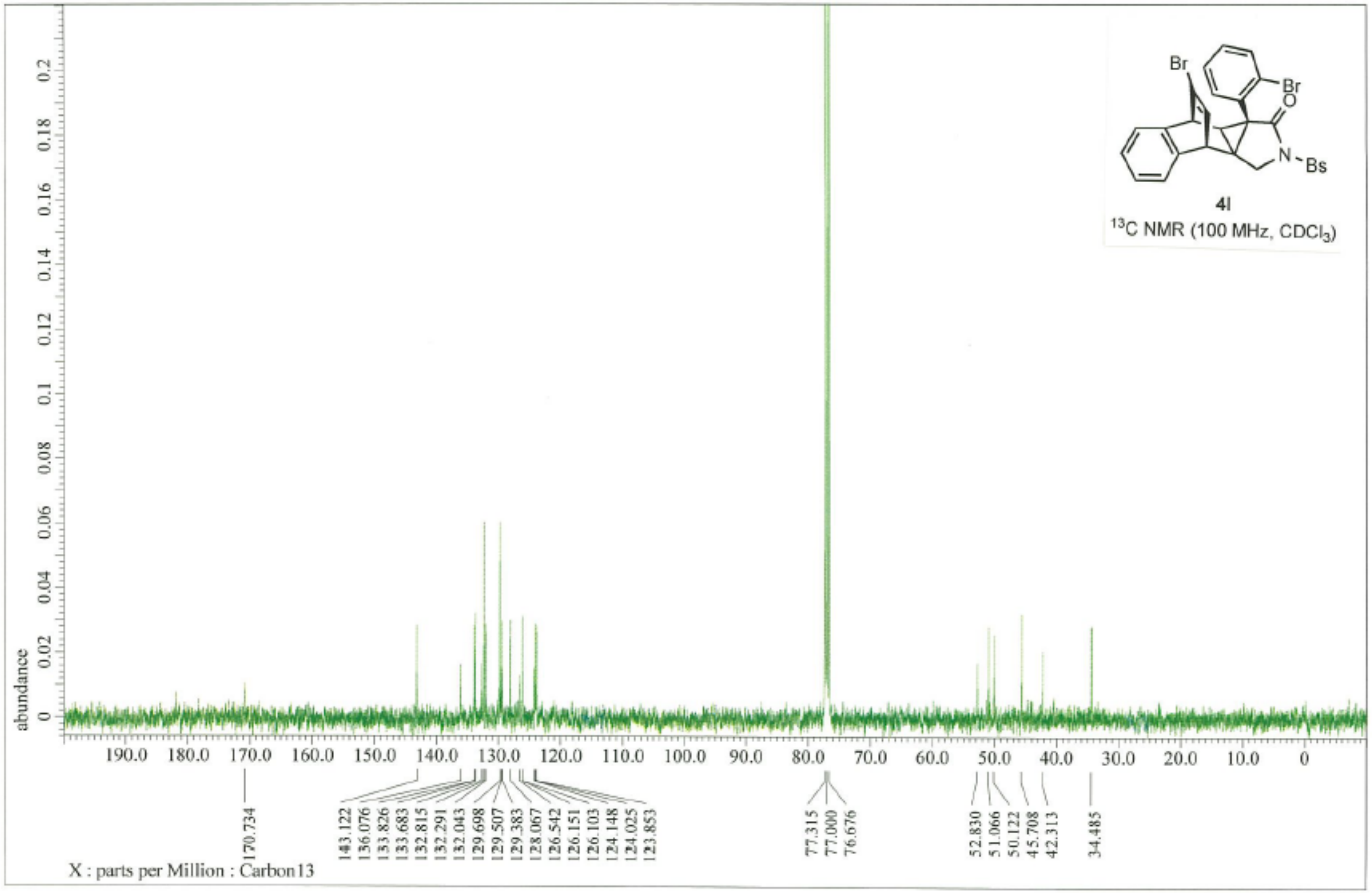
Br
Br
O
N
Bs
4l
^{13}C NMR (100 MHz, CDCl$_3$)
abundance
0.2
0.18
0.16
0.14
0.12
0.1
0.08
0.06
0.04
0.02
0
190.0 180.0 170.0 160.0 150.0 140.0 130.0 120.0 110.0 100.0 90.0 80.0 70.0 60.0 50.0 40.0 30.0 20.0 10.0 0
170.734
143.122
136.076
133.826
133.683
132.815
132.291
132.043
129.698
129.507
129.383
128.067
126.542
126.151
126.103
124.148
124.025
123.853
77.315
77.000
76.676
52.830
51.066
50.122
45.708
42.313
34.485
X : parts per Million : Carbon13

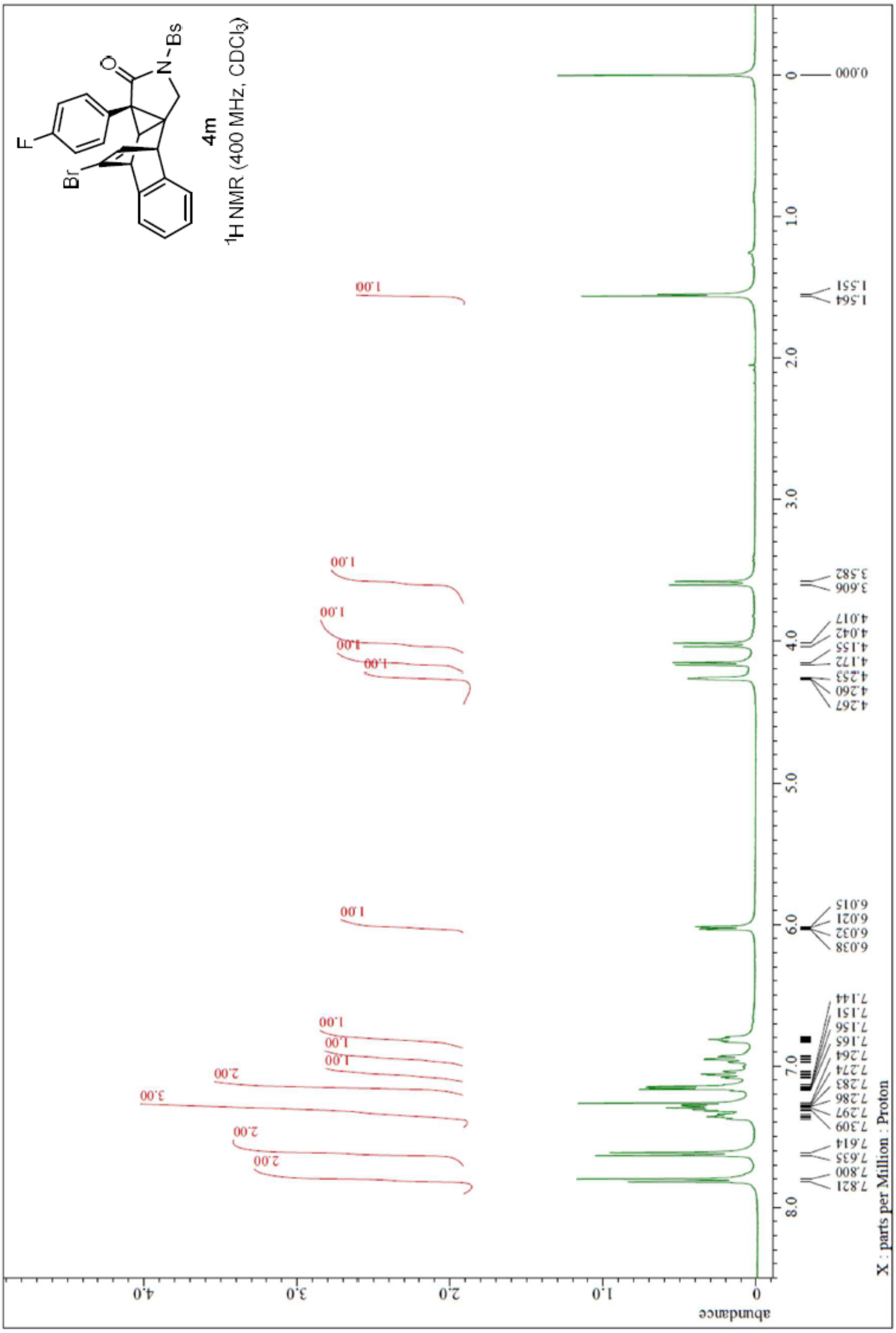

Br
F
O
N
Bs
4m
^{1}H NMR (400 MHz, CDCl$_3$)
0.000
1.551
1.564
3.582
3.606
4.017
4.042
4.155
4.172
4.253
4.260
4.267
6.015
6.021
6.032
6.038
7.144
7.151
7.156
7.165
7.264
7.274
7.283
7.286
7.297
7.309
7.614
7.635
7.800
7.821
X : parts per Million : Proton
abundance

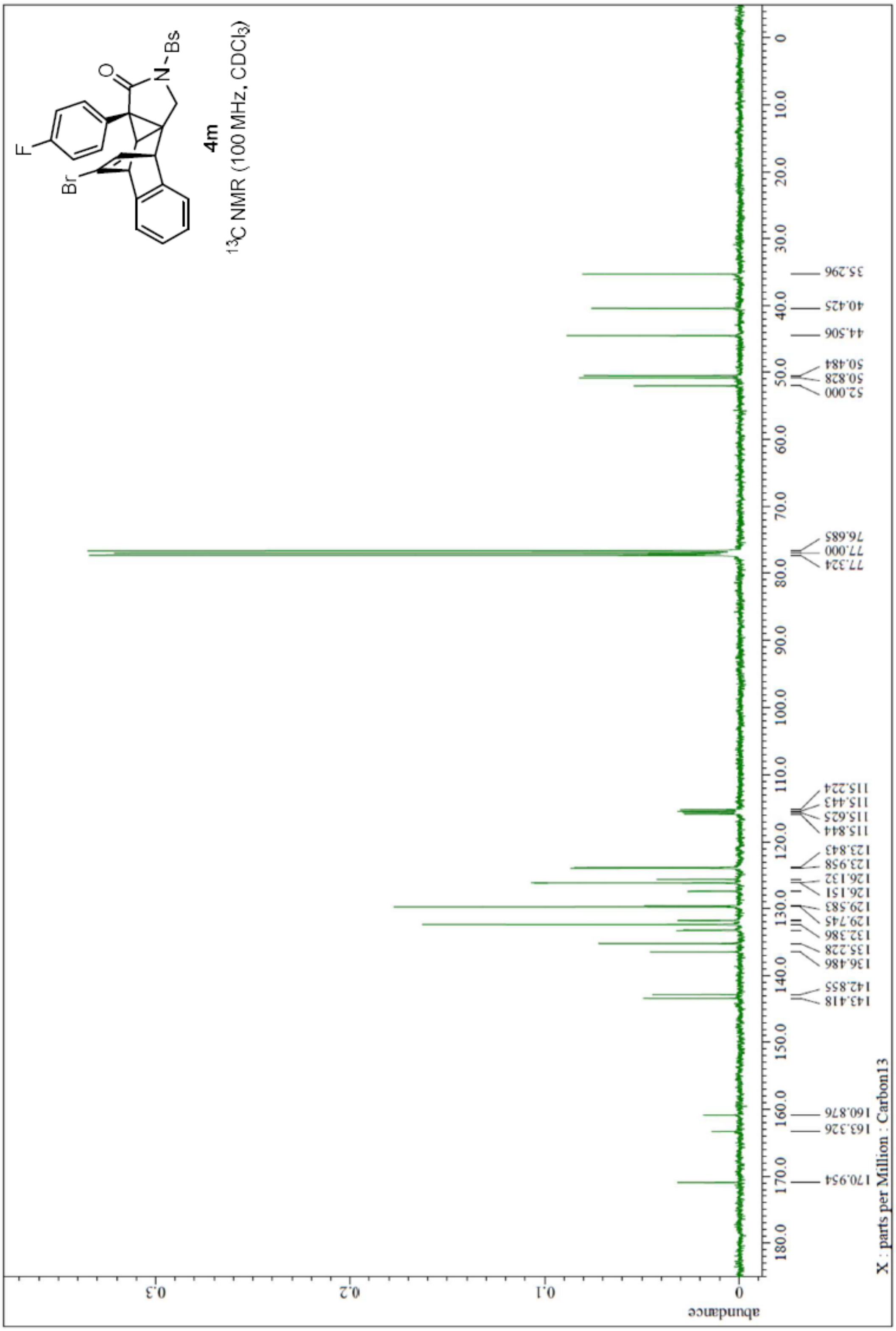

4m
13C NMR (100 MHz, CDCl3)
35.296
40.425
44.506
50.484
50.828
52.000
76.685
77.000
77.324
115.224
115.443
115.625
115.844
123.843
123.958
126.132
126.151
129.583
129.745
132.386
135.228
136.486
142.855
143.418
160.876
163.326
170.954
X : parts per Million : Carbon13
abundance

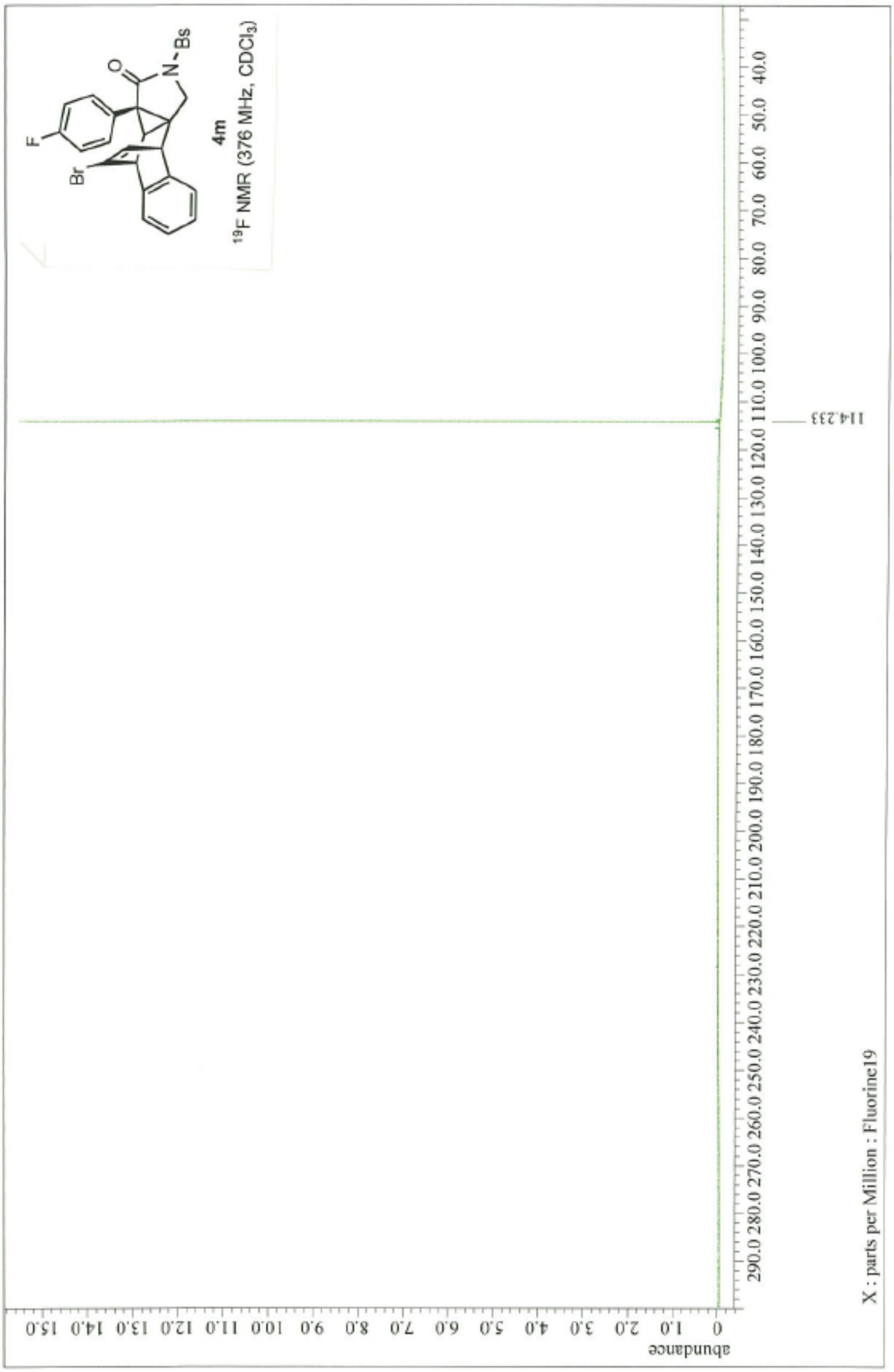
Br
F
N~Bs
O
4m
19F NMR (376 MHz, CDCl3)
114.233
X : parts per Million : Fluorine19
abundance

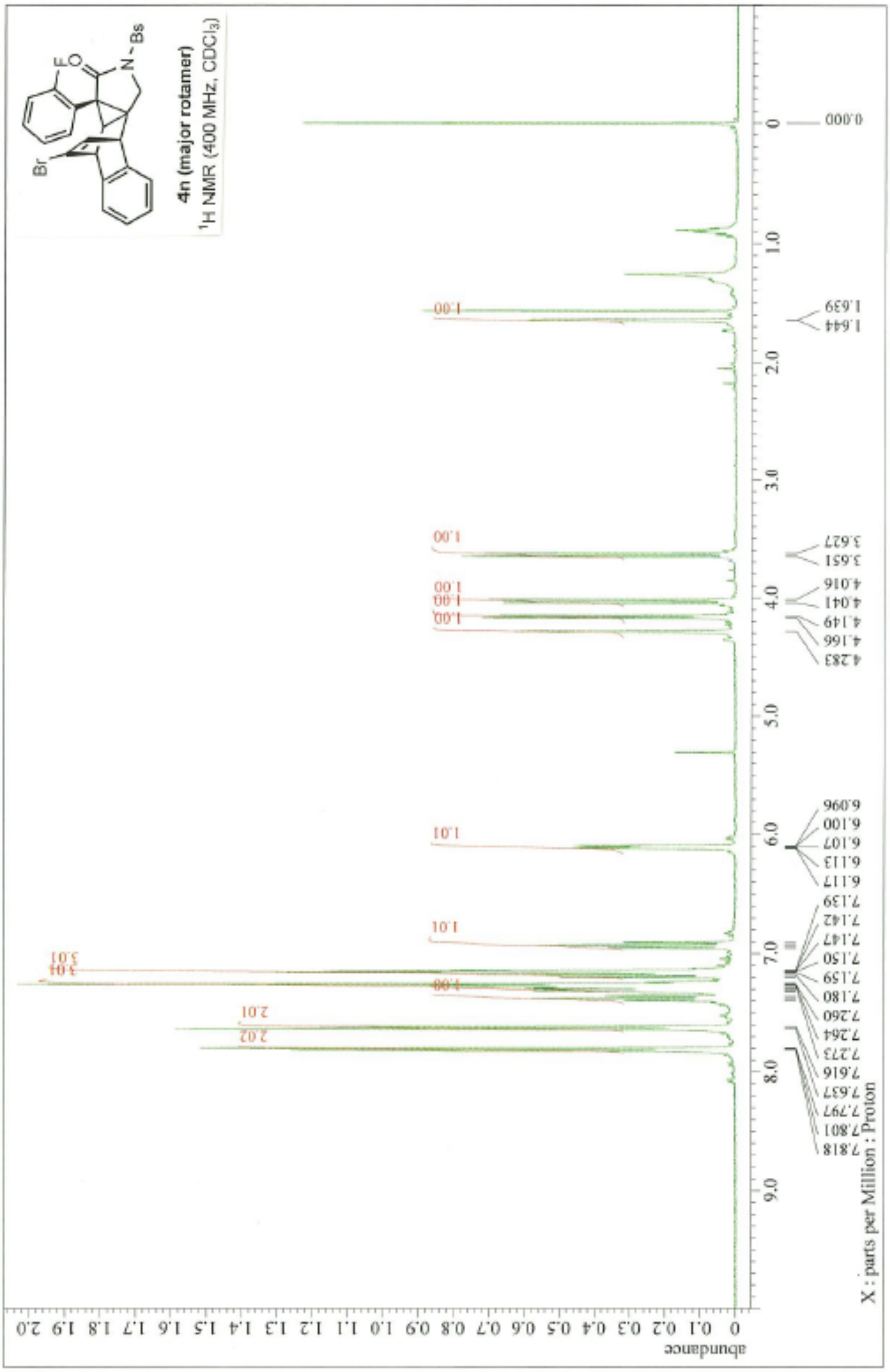

4n (major rotamer)
1H NMR (400 MHz, CDCl3)
X : parts per Million : Proton
abundance

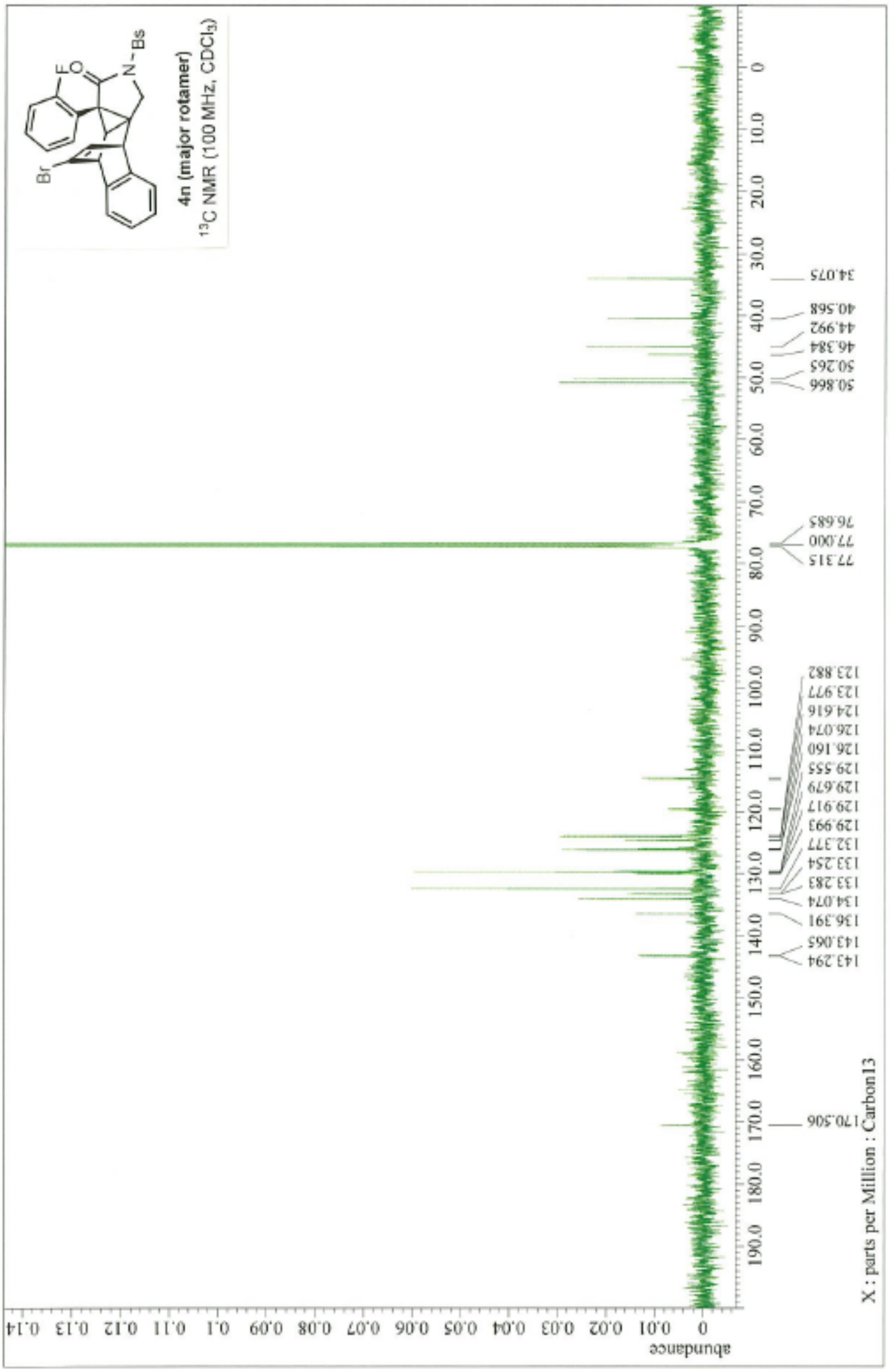

4n (major rotamer)
^{13}C NMR (100 MHz, CDCl$_3$)
Br
F
O
N~Bs
34.075
40.568
44.992
46.384
50.265
50.866
76.685
77.000
77.315
123.882
123.977
124.616
126.074
126.160
129.555
129.679
129.917
129.993
132.377
133.254
133.283
134.074
136.391
143.065
143.294
170.506
X : parts per Million : Carbon13
abundance

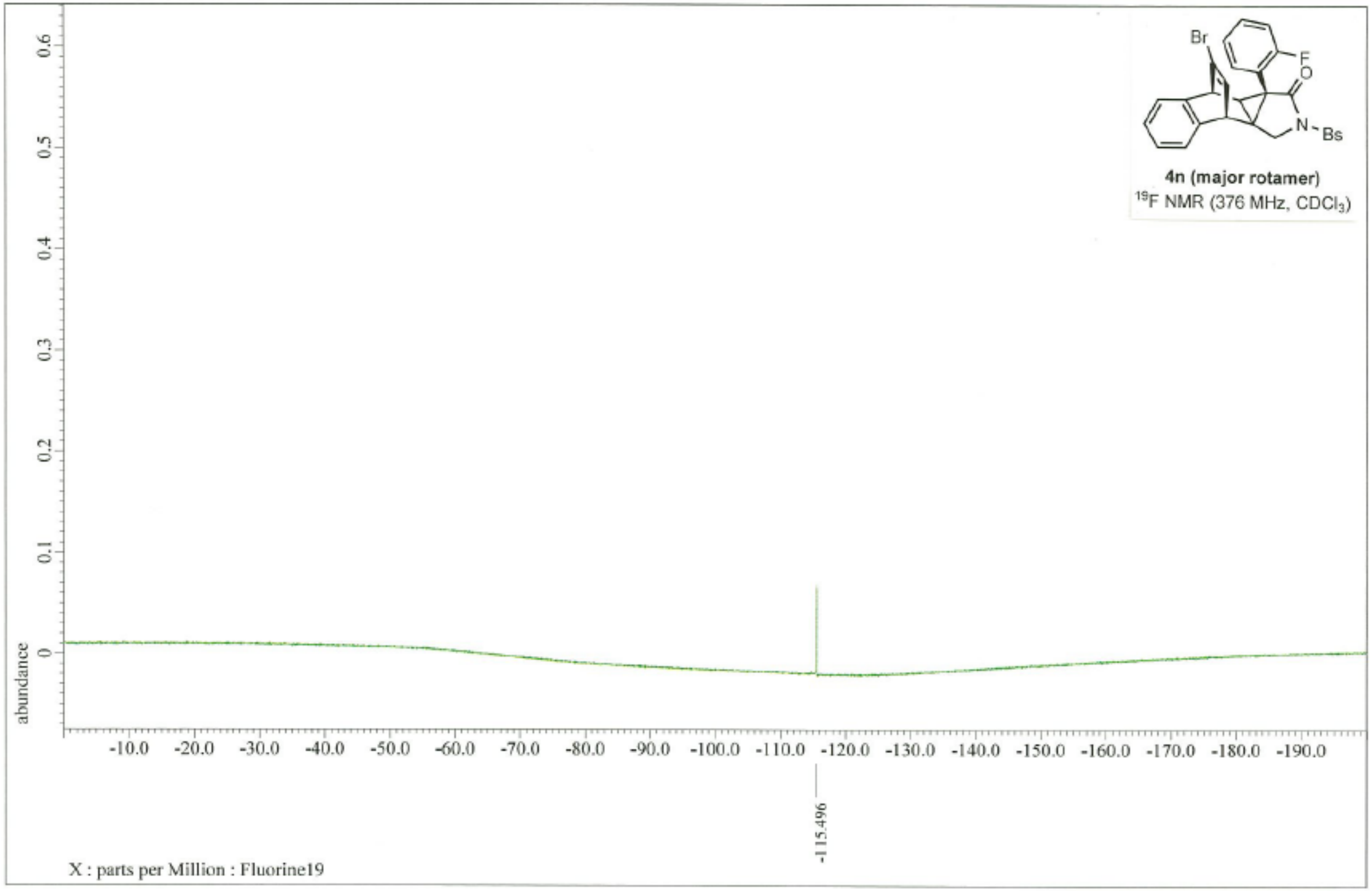
Br
F
O
N
Bs
4n (major rotamer)
19F NMR (376 MHz, CDCl3)
abundance
0
0.1
0.2
0.3
0.4
0.5
0.6
-10.0
-20.0
-30.0
-40.0
-50.0
-60.0
-70.0
-80.0
-90.0
-100.0
-110.0
-120.0
-130.0
-140.0
-150.0
-160.0
-170.0
-180.0
-190.0
-115.496
X : parts per Million : Fluorine19

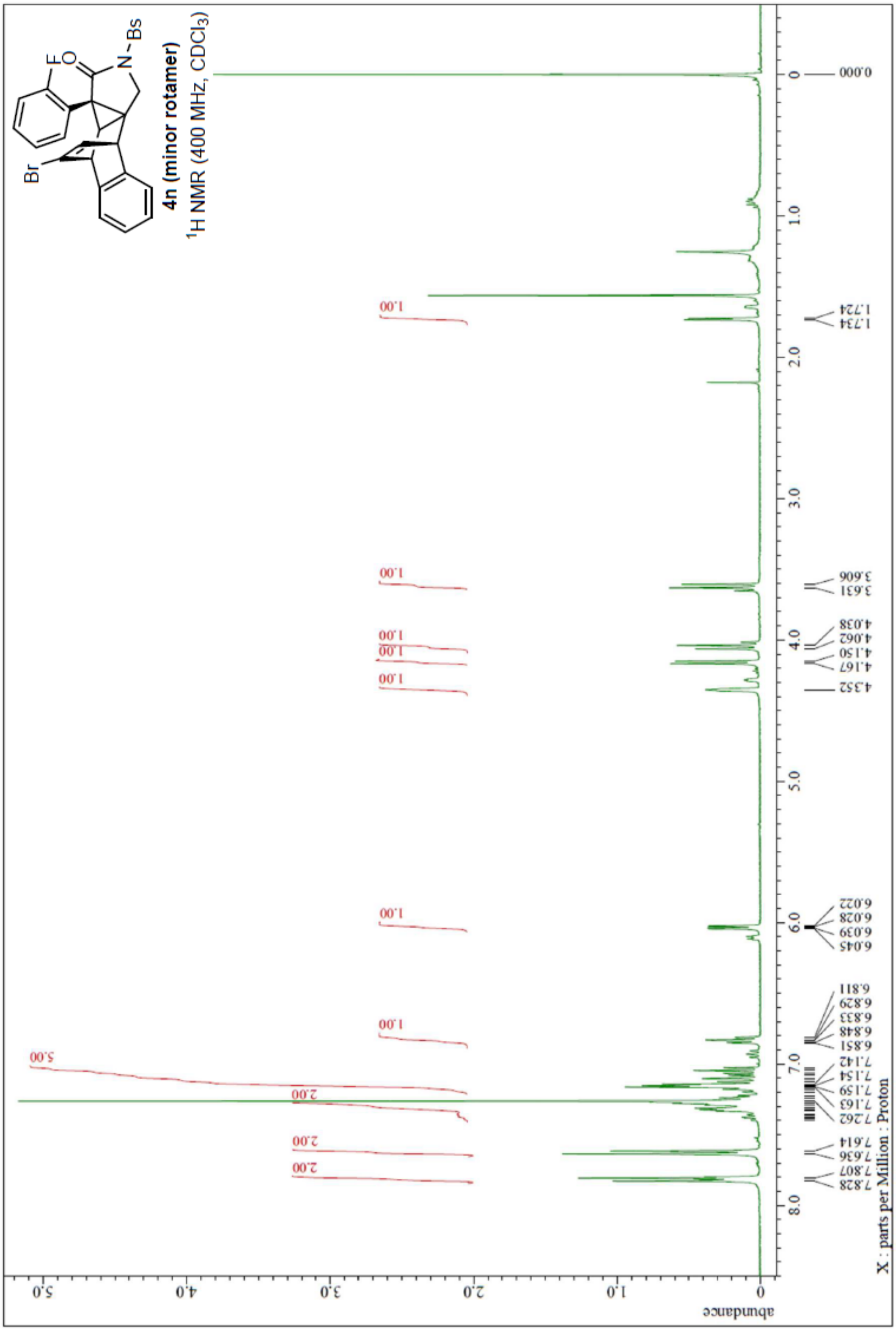

4n (minor rotamer)
1H NMR (400 MHz, CDCl3)
Br
F
O
N
Bs
0.000
1.724
1.734
3.606
3.631
4.038
4.062
4.150
4.167
4.352
6.022
6.028
6.039
6.045
6.811
6.829
6.833
6.848
6.851
7.142
7.154
7.159
7.163
7.262
7.614
7.636
7.807
7.828
X : parts per Million : Proton
abundance

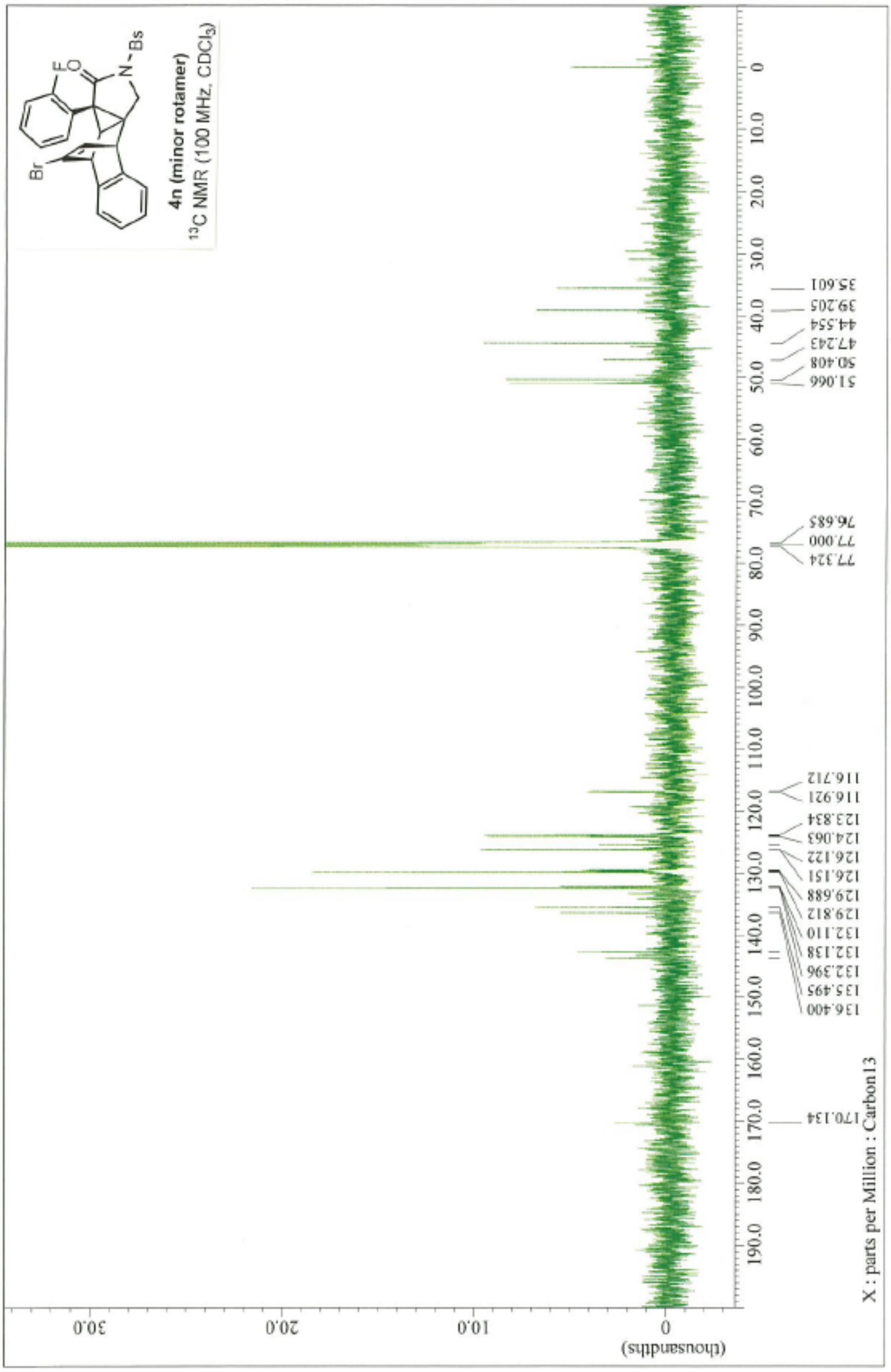

4n (minor rotamer)
^{13}C NMR (100 MHz, CDCl$_3$)
35.601
39.205
44.554
47.243
50.408
51.066
76.685
77.000
77.324
116.712
116.921
123.834
124.063
126.122
126.151
129.688
129.812
132.110
132.138
132.396
135.495
136.400
170.134
X : parts per Million : Carbon13
(thousandths)

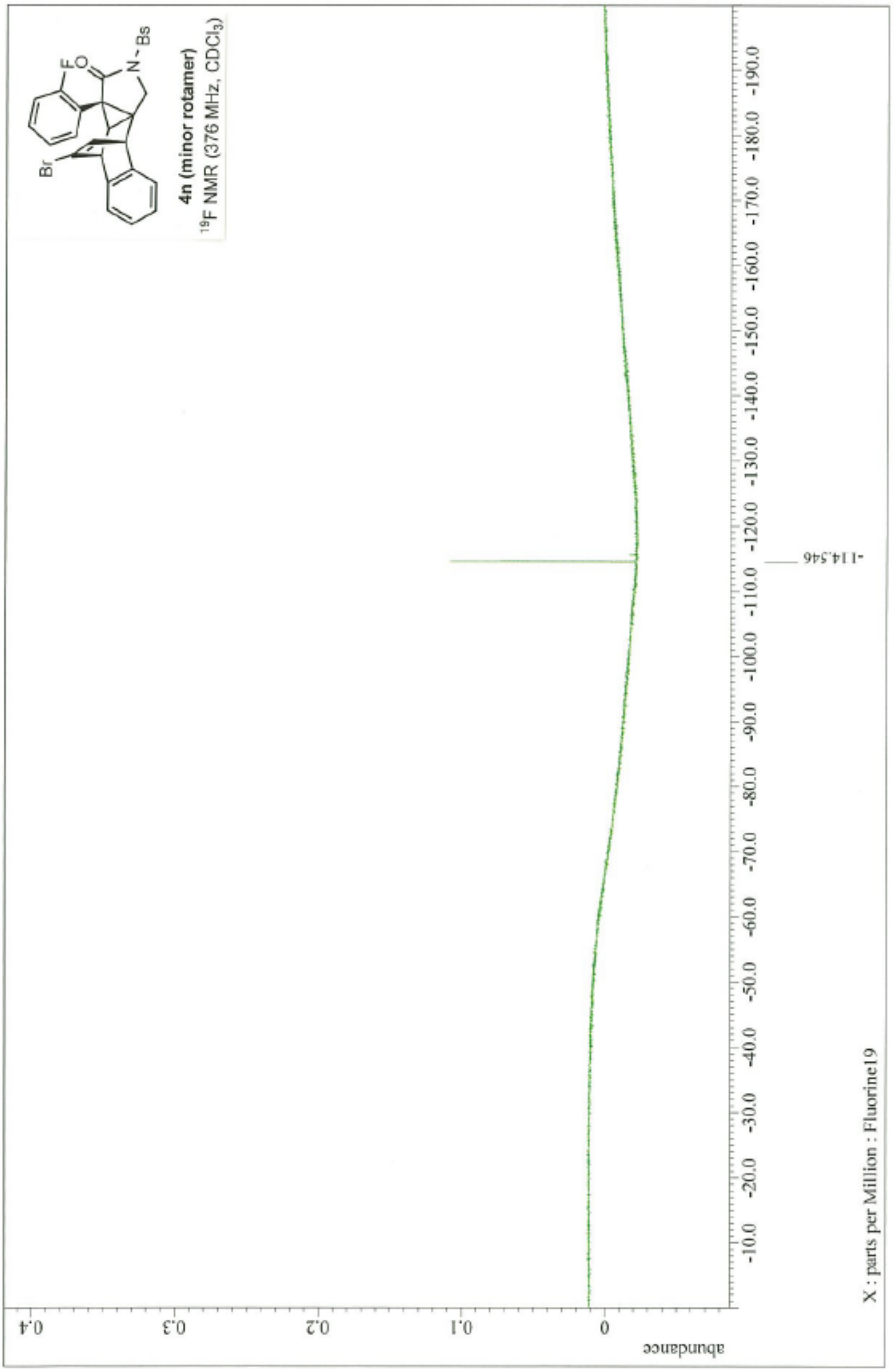
4n (minor rotamer)
19F NMR (376 MHz, CDCl3)
-114.546
X : parts per Million : Fluorine19
abundance

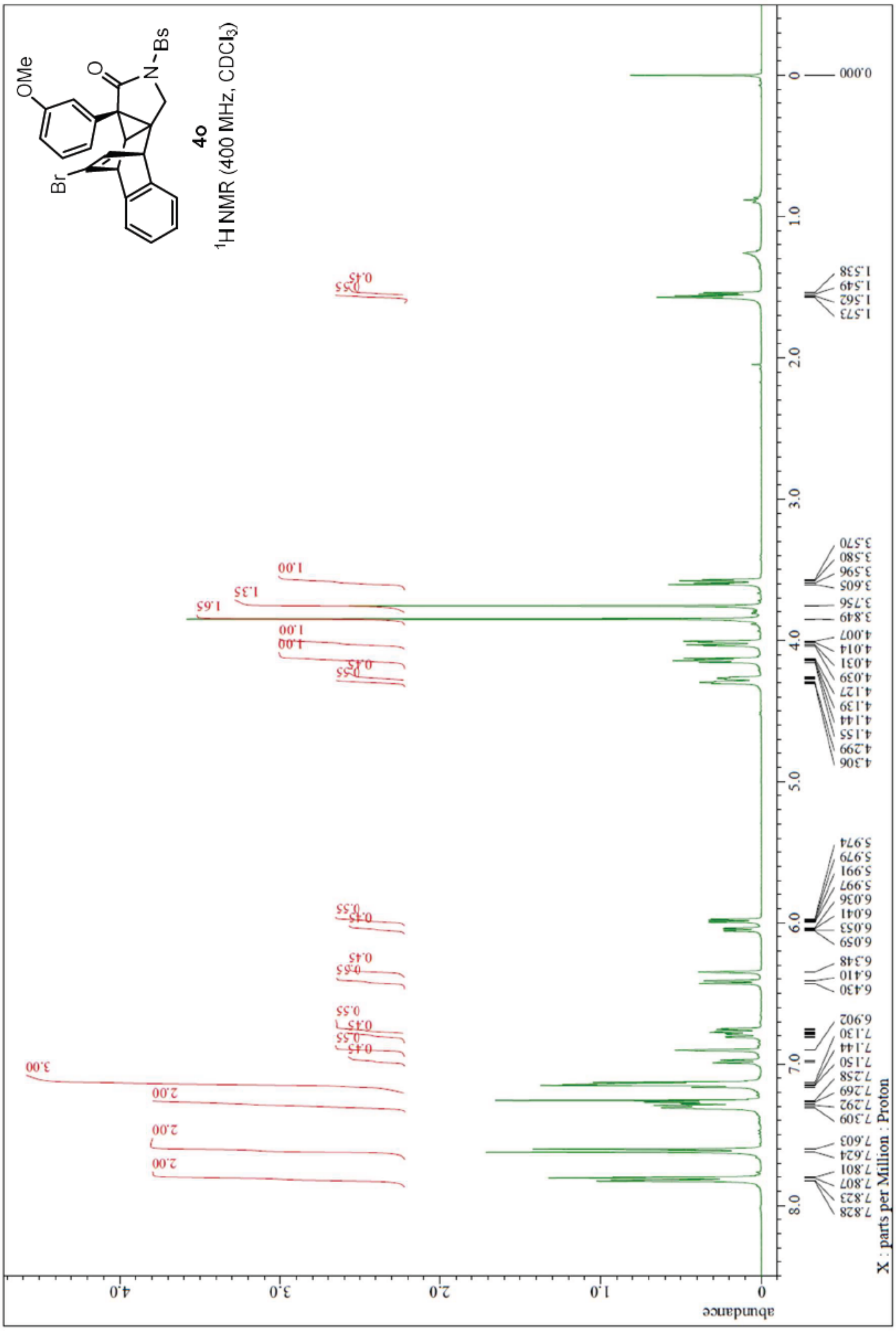
4o
¹H NMR (400 MHz, CDCl3)
X : parts per Million : Proton
abundance

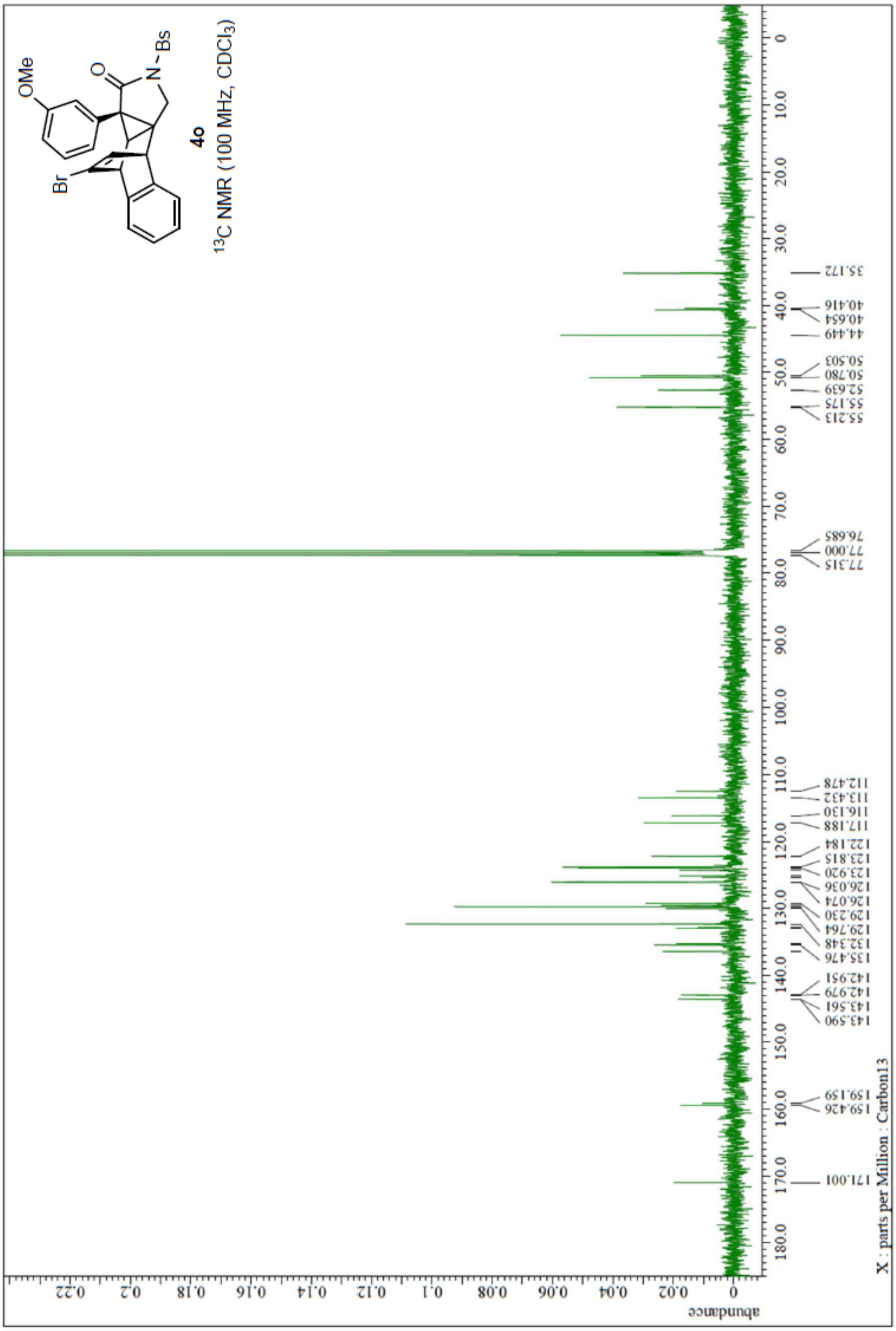
Br
OMe
O
N~Bs
4o
13C NMR (100 MHz, CDCl3)
35.172
40.416
40.654
44.449
50.503
50.780
52.639
55.175
55.213
76.685
77.000
77.315
112.478
113.432
116.130
117.188
122.184
123.815
123.920
126.036
126.074
129.230
129.764
132.348
135.476
142.951
142.979
143.561
143.590
159.159
159.426
171.001
X : parts per Million : Carbon13
abundance

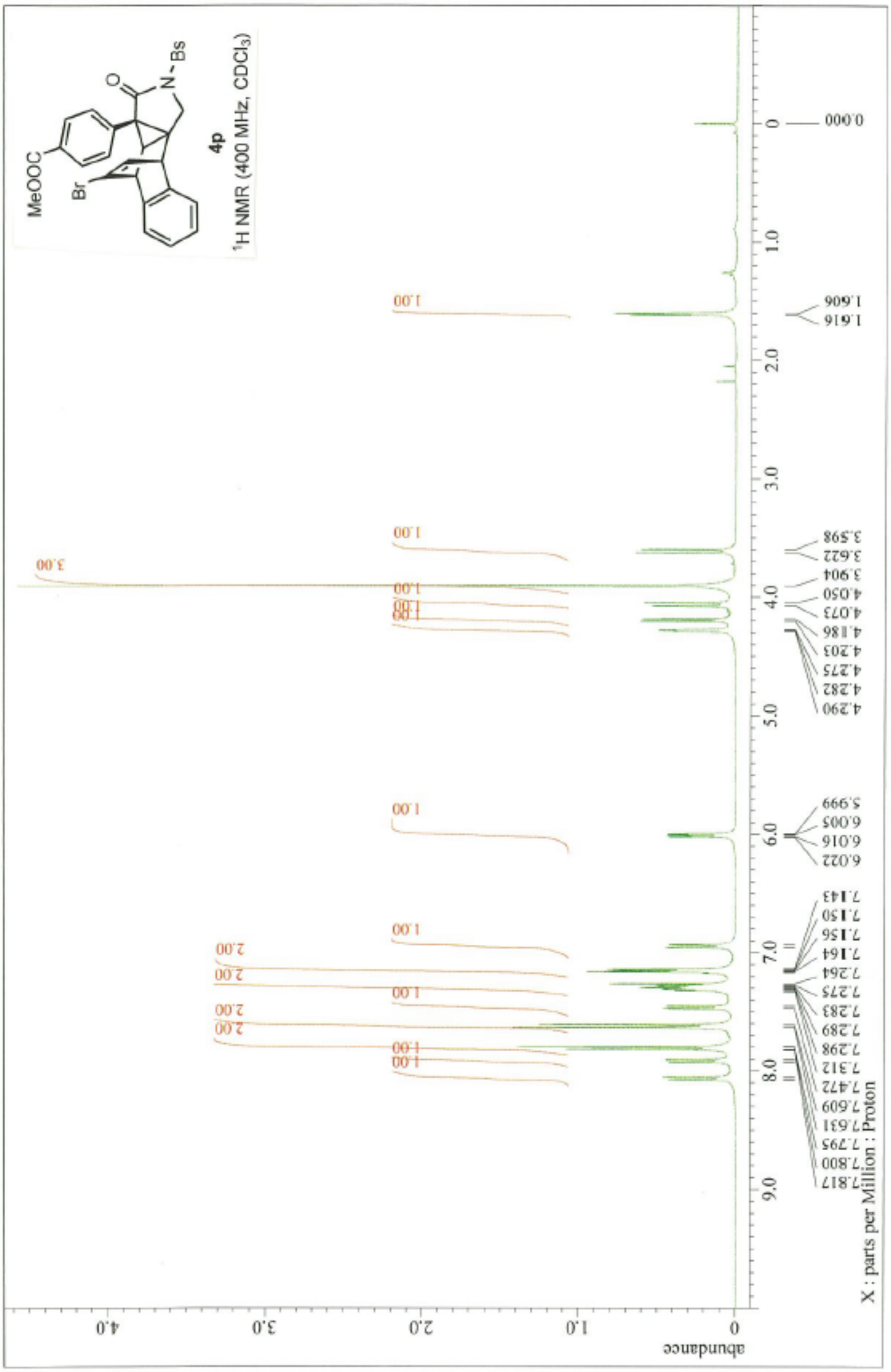
MeOOC
Br
O
N
Bs
4p
1H NMR (400 MHz, CDCl3)
0.000
1.606
1.616
3.598
3.622
3.904
4.050
4.073
4.186
4.203
4.275
4.282
4.290
5.999
6.005
6.016
6.022
7.143
7.150
7.156
7.164
7.264
7.275
7.283
7.289
7.298
7.312
7.472
7.609
7.631
7.795
7.800
7.817
X : parts per Million : Proton
abundance

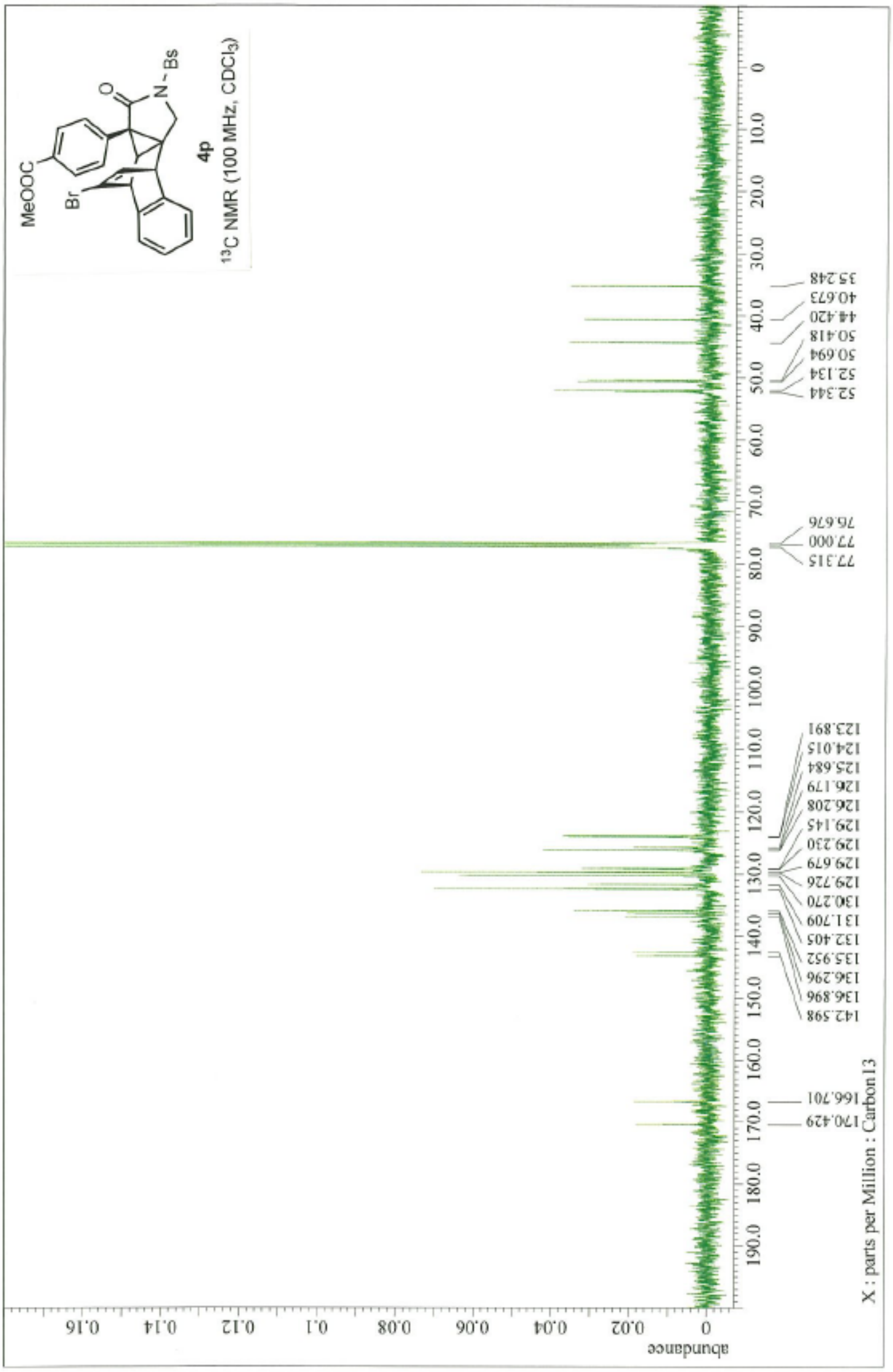

MeOOC
Br
N~Bs
O
4p
^{13}C NMR (100 MHz, CDCl$_3$)
35.248
40.673
44.420
50.418
50.694
52.134
52.344
76.676
77.000
77.315
123.891
124.015
125.684
126.179
126.208
129.145
129.230
129.679
129.726
130.270
131.709
132.405
135.952
136.296
136.896
142.598
166.701
170.429
X : parts per Million : Carbon13
abundance

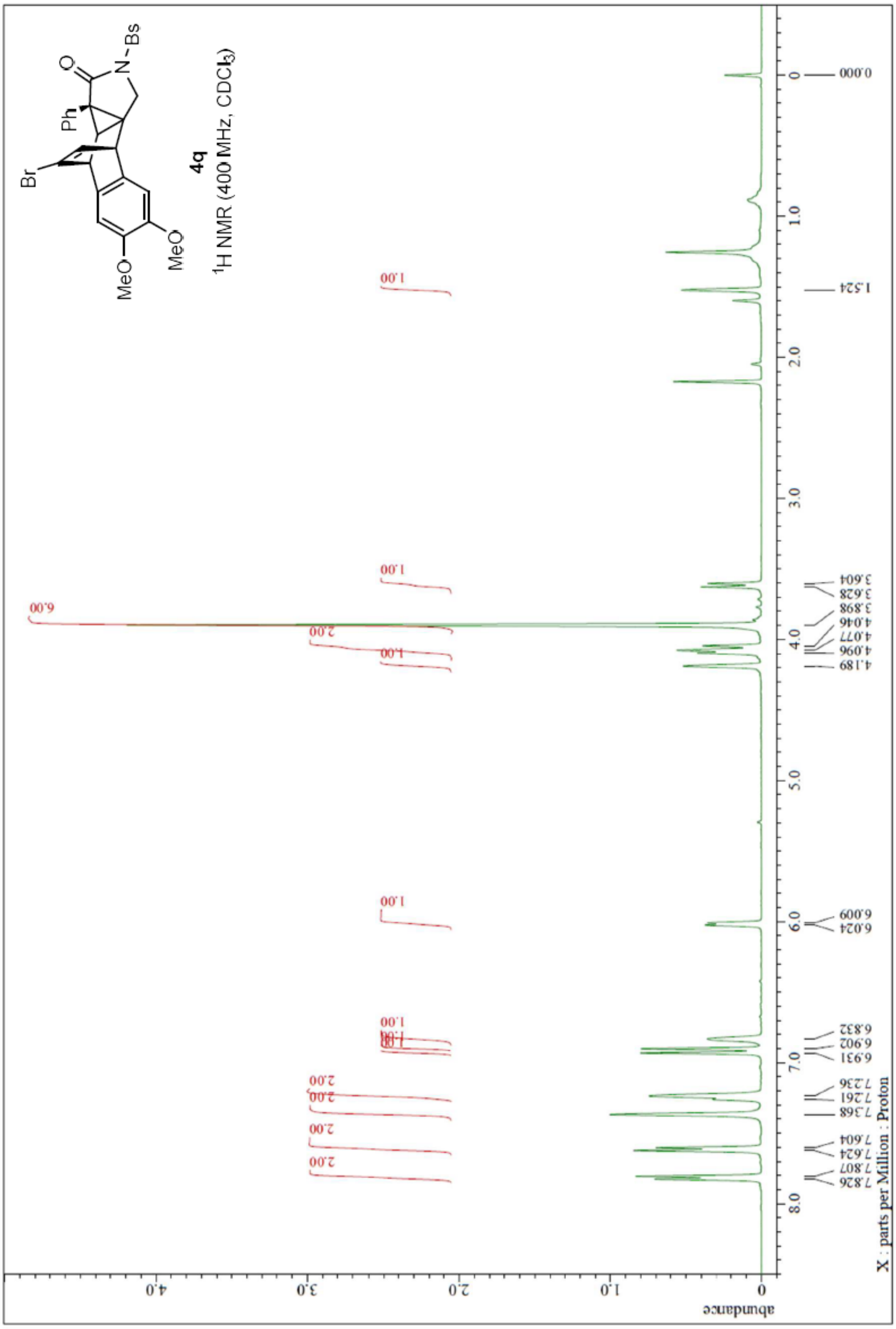
Br
Ph
O
N–Bs
MeO
MeO
4q
1H NMR (400 MHz, CDCl3)
X : parts per Million : Proton
abundance
0.000
1.524
3.604
3.628
3.898
4.046
4.077
4.096
4.189
6.009
6.024
6.832
6.902
6.931
7.236
7.261
7.368
7.604
7.624
7.807
7.826
1.00
6.00
2.00
2.00

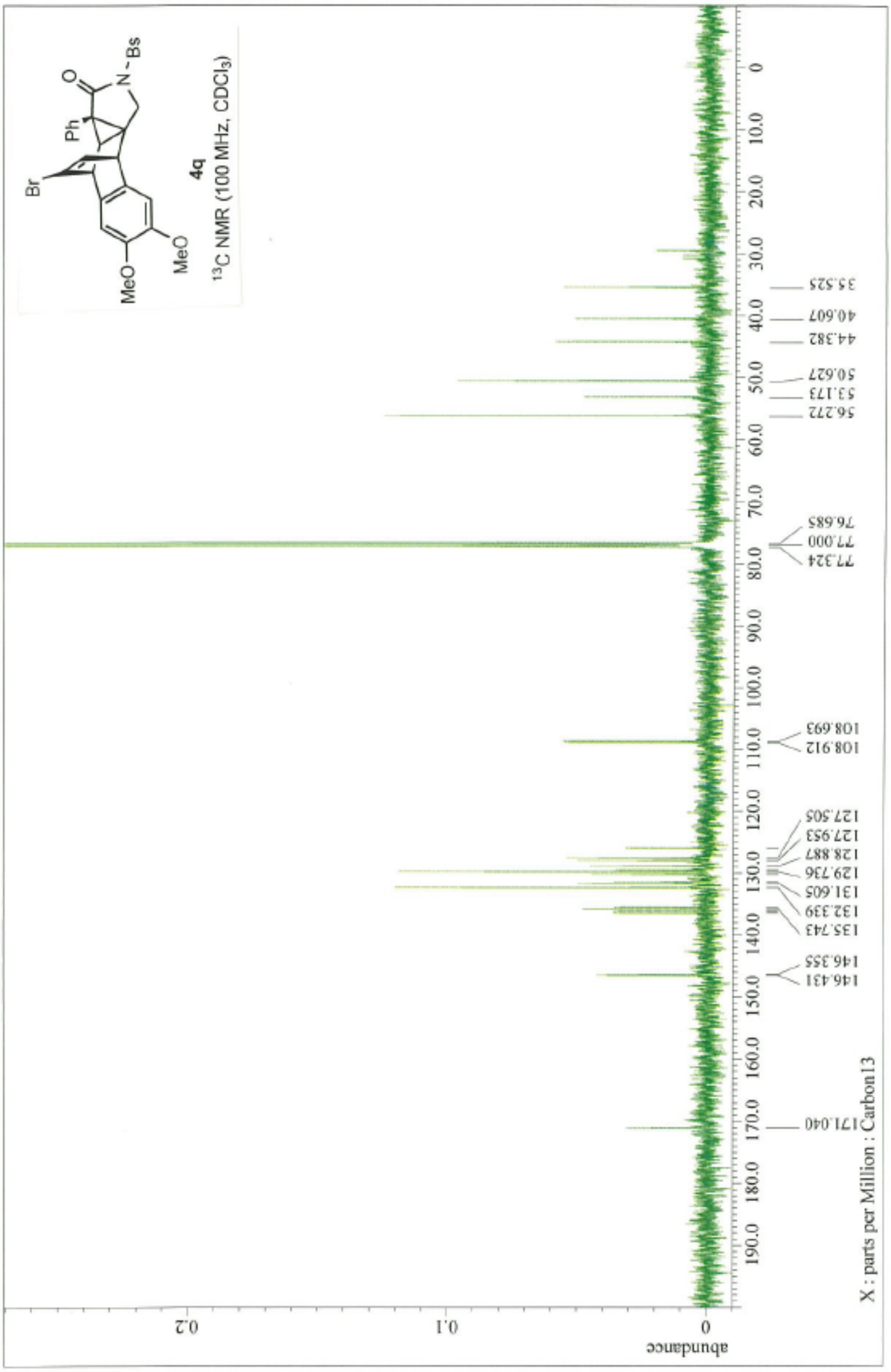

Br
Ph
O
N
Bs
MeO
MeO
4q
13C NMR (100 MHz, CDCl3)
35.525
40.607
44.382
50.627
53.173
56.272
76.685
77.000
77.324
108.693
108.912
127.505
127.953
128.887
129.736
131.605
132.339
135.743
146.355
146.431
171.040
X : parts per Million : Carbon13
abundance

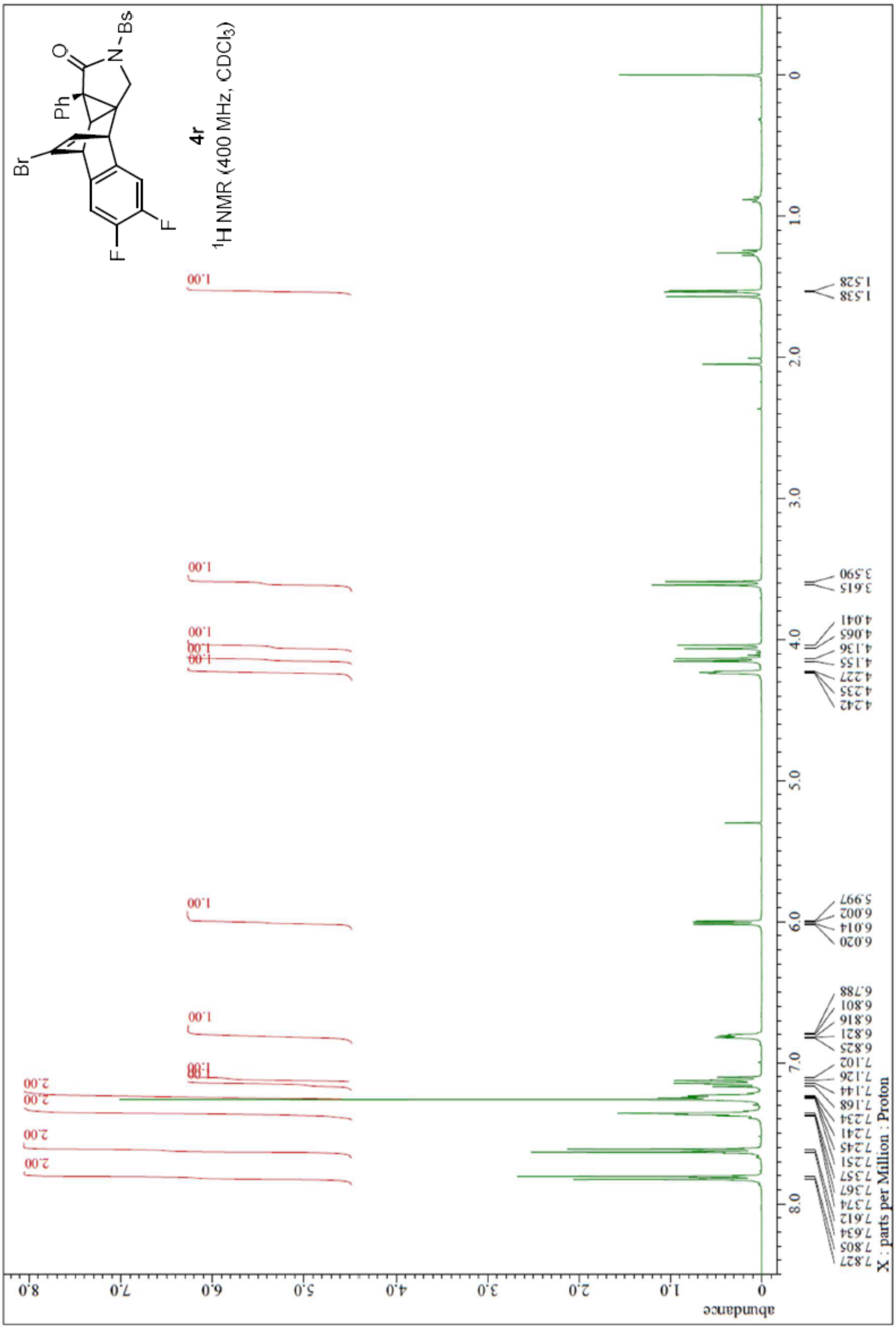
4r
1H NMR (400 MHz, CDCl3)
X : parts per Million : Proton
abundance

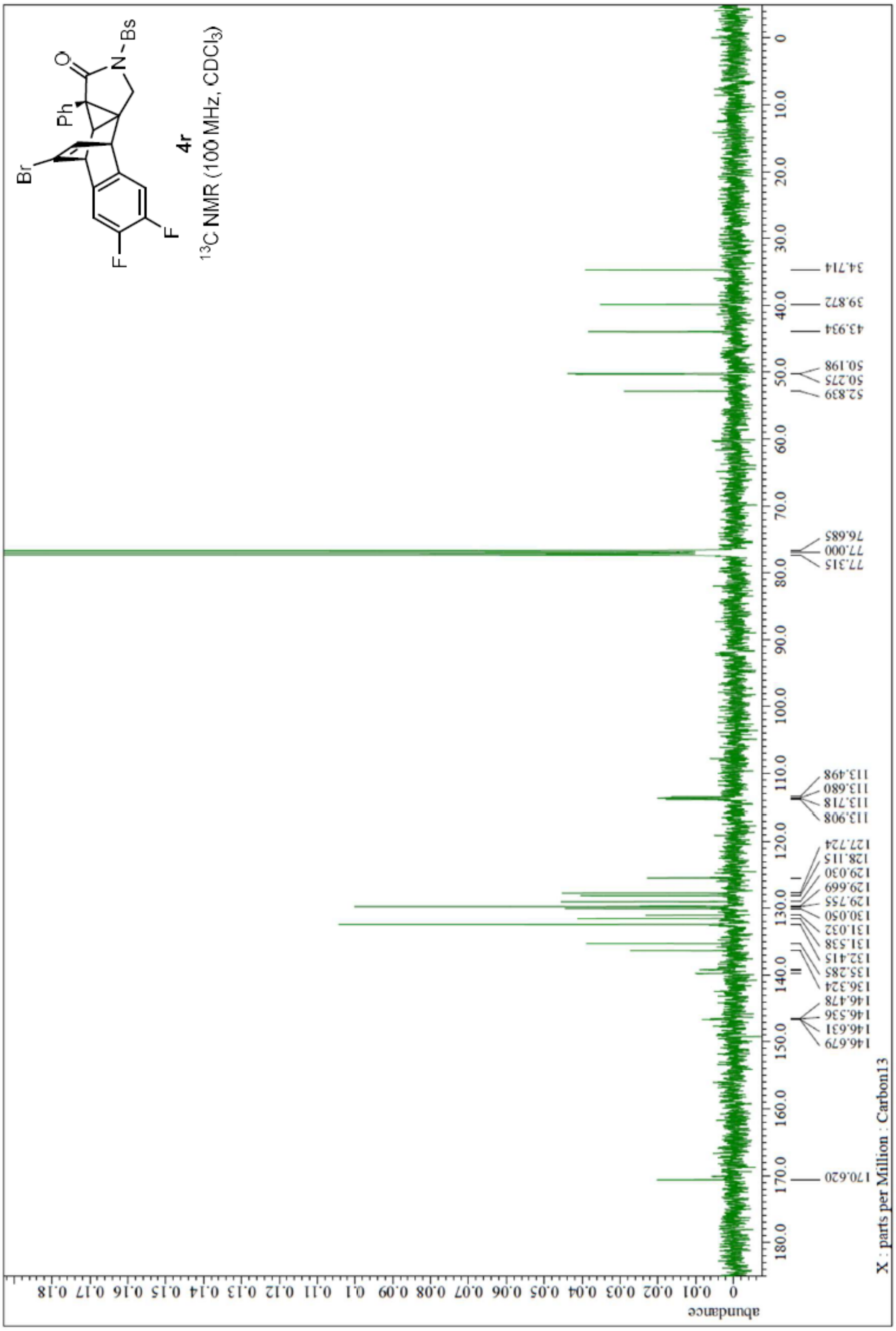
4r
13C NMR (100 MHz, CDCl3)
34.714
39.872
43.934
50.198
50.275
52.839
76.685
77.000
77.315
113.498
113.680
113.718
113.908
127.724
128.115
129.030
129.669
129.755
130.050
131.032
131.538
132.415
135.285
136.324
146.478
146.536
146.631
146.679
170.620
X : parts per Million : Carbon13
abundance

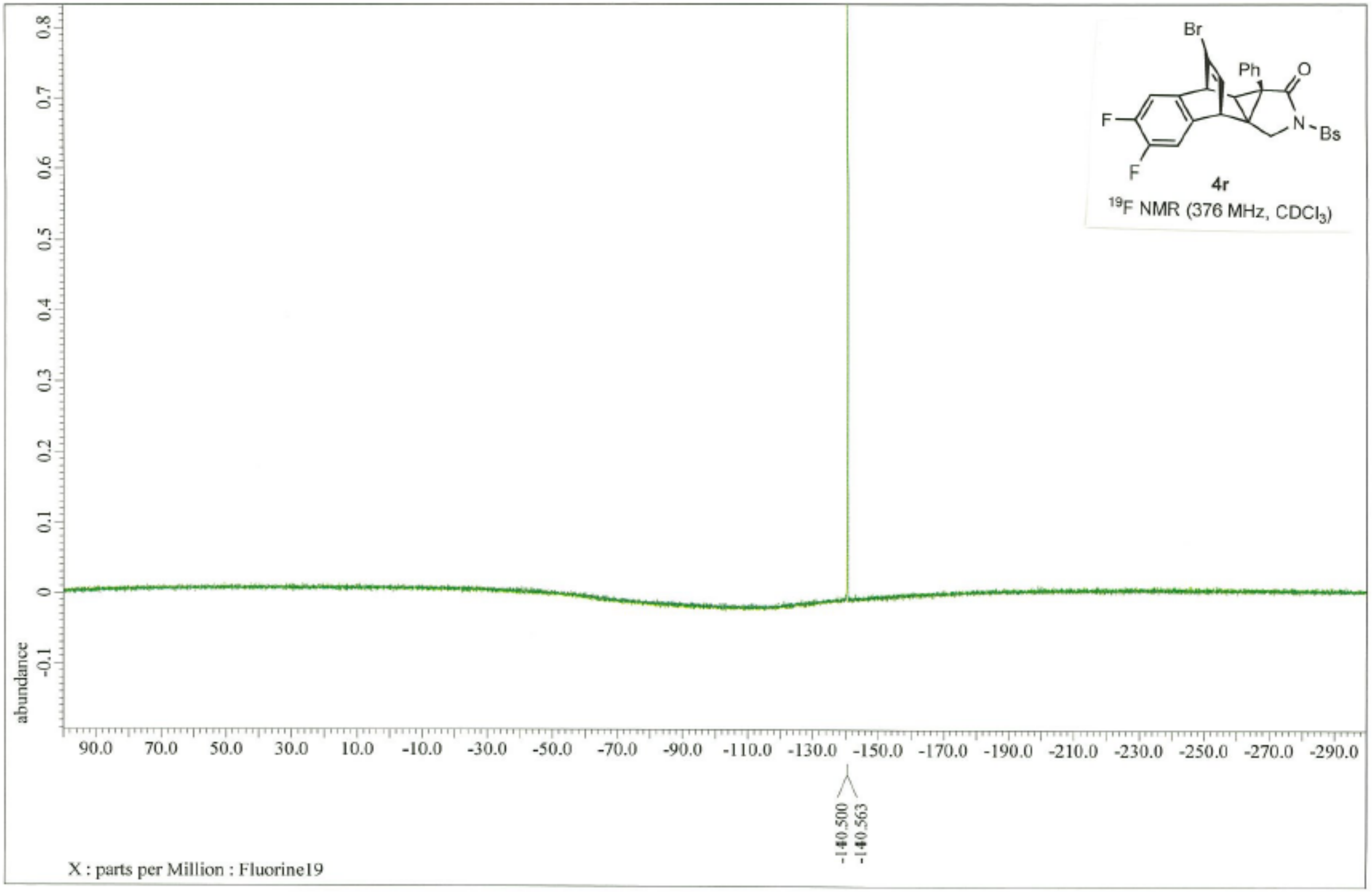

Br
Ph
O
F
N
Bs
F
4r
19F NMR (376 MHz, CDCl3)
abundance
0.8
0.7
0.6
0.5
0.4
0.3
0.2
0.1
0
-0.1
90.0 70.0 50.0 30.0 10.0 -10.0 -30.0 -50.0 -70.0 -90.0 -110.0 -130.0 -150.0 -170.0 -190.0 -210.0 -230.0 -250.0 -270.0 -290.0
-140.500
-140.563
X : parts per Million : Fluorine19

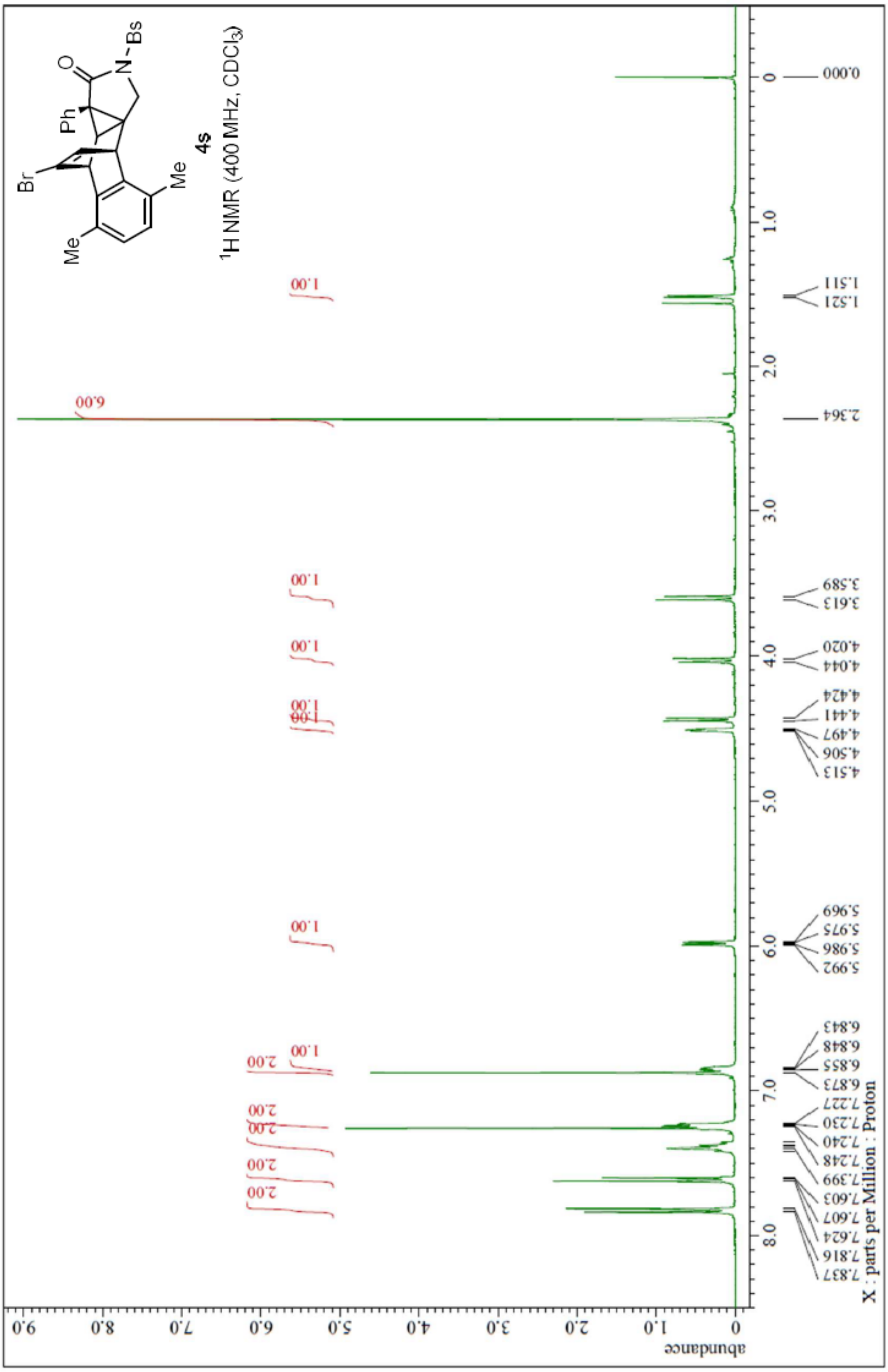

4s
1H NMR (400 MHz, CDCl3)
X : parts per Million : Proton
abundance

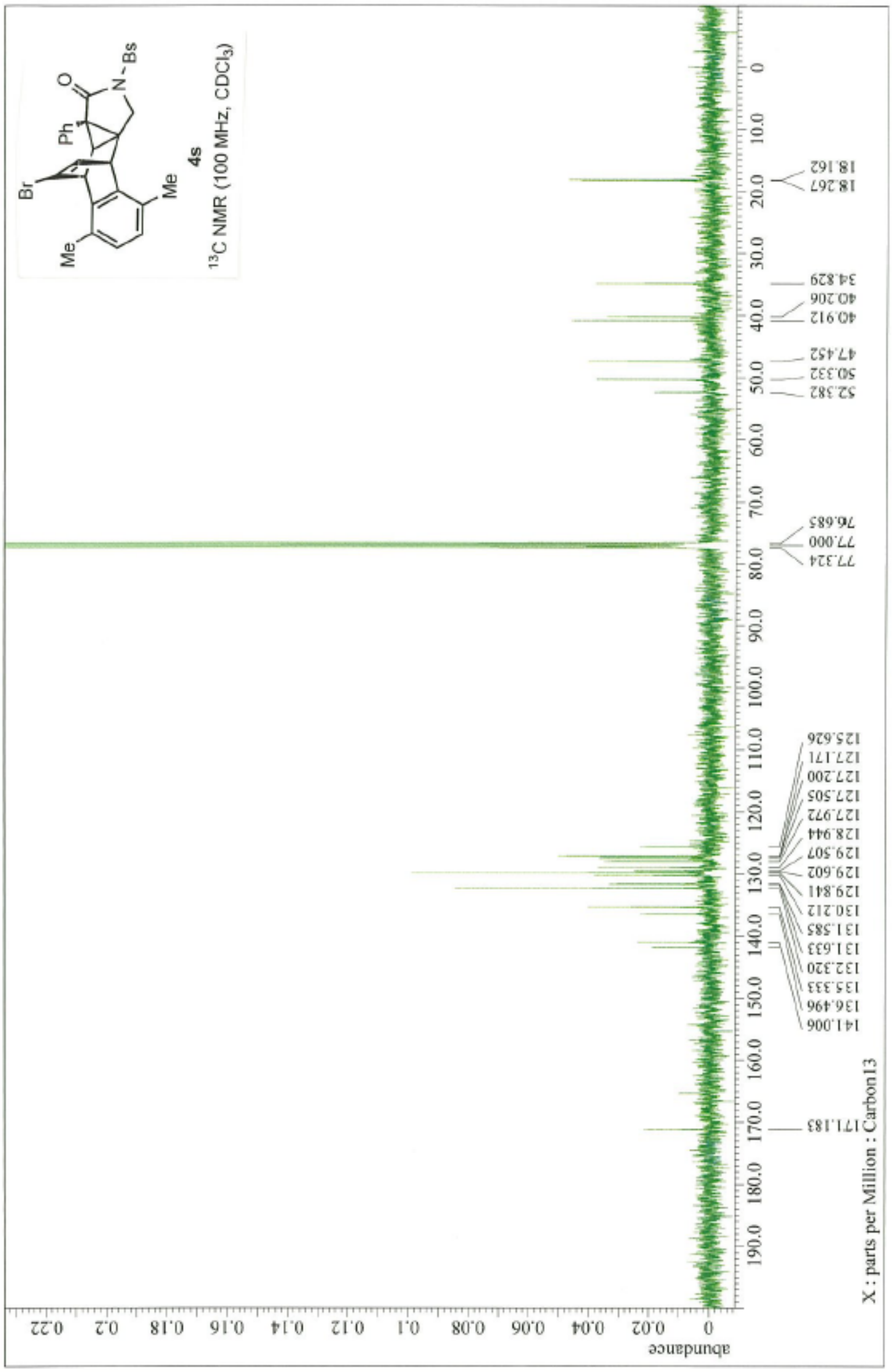

Br
Ph
O
N~Bs
Me
Me
4s
13C NMR (100 MHz, CDCl3)
18.162
18.267
34.829
40.206
40.912
47.452
50.332
52.382
76.685
77.000
77.324
125.626
127.171
127.200
127.505
127.972
128.944
129.507
129.602
129.841
130.212
131.585
131.633
132.320
135.333
136.496
141.006
171.183
X : parts per Million : Carbon13
abundance

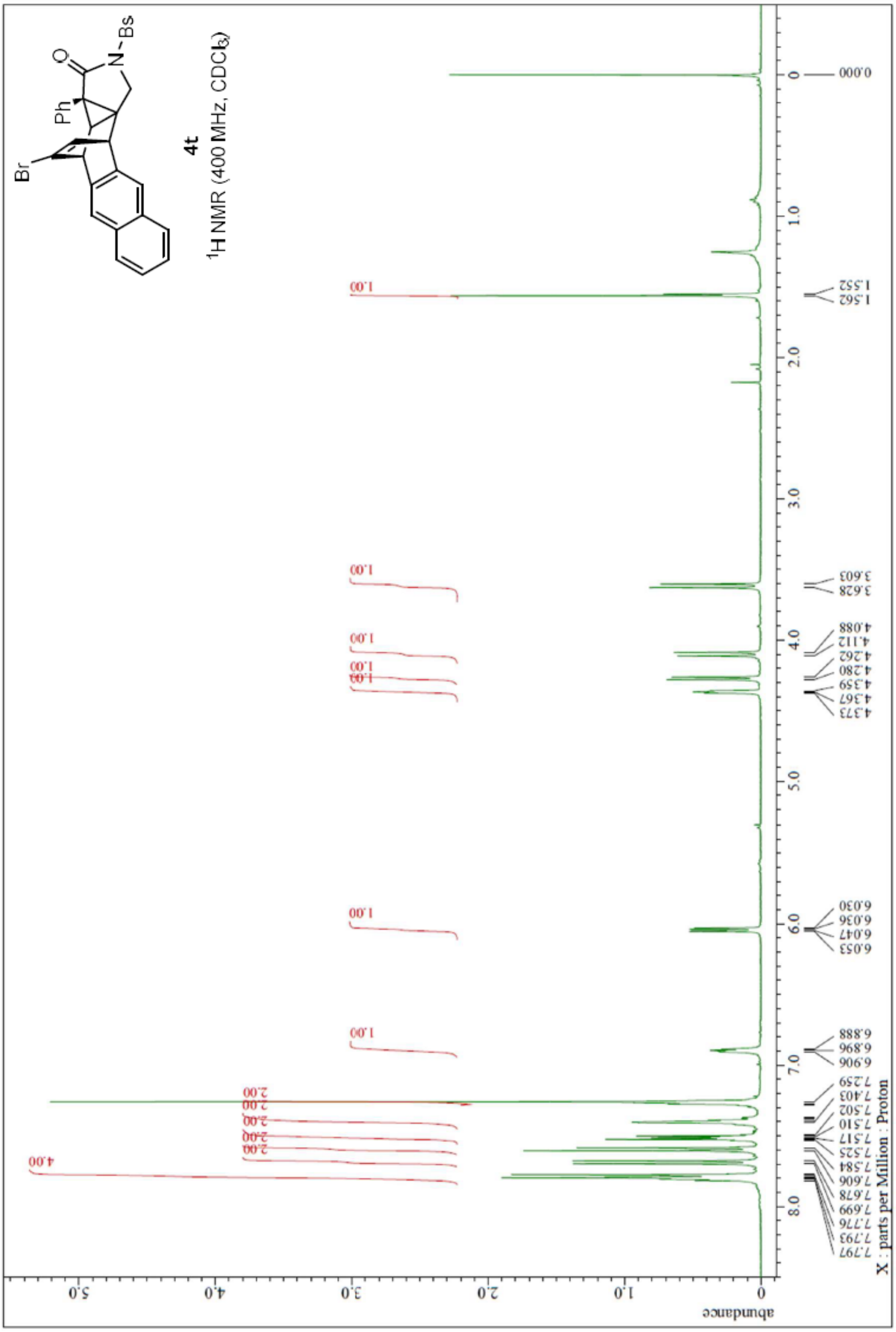

Br
Ph
O
N-Bs
4t
1H NMR (400 MHz, CDCl3)
X : parts per Million : Proton
abundance

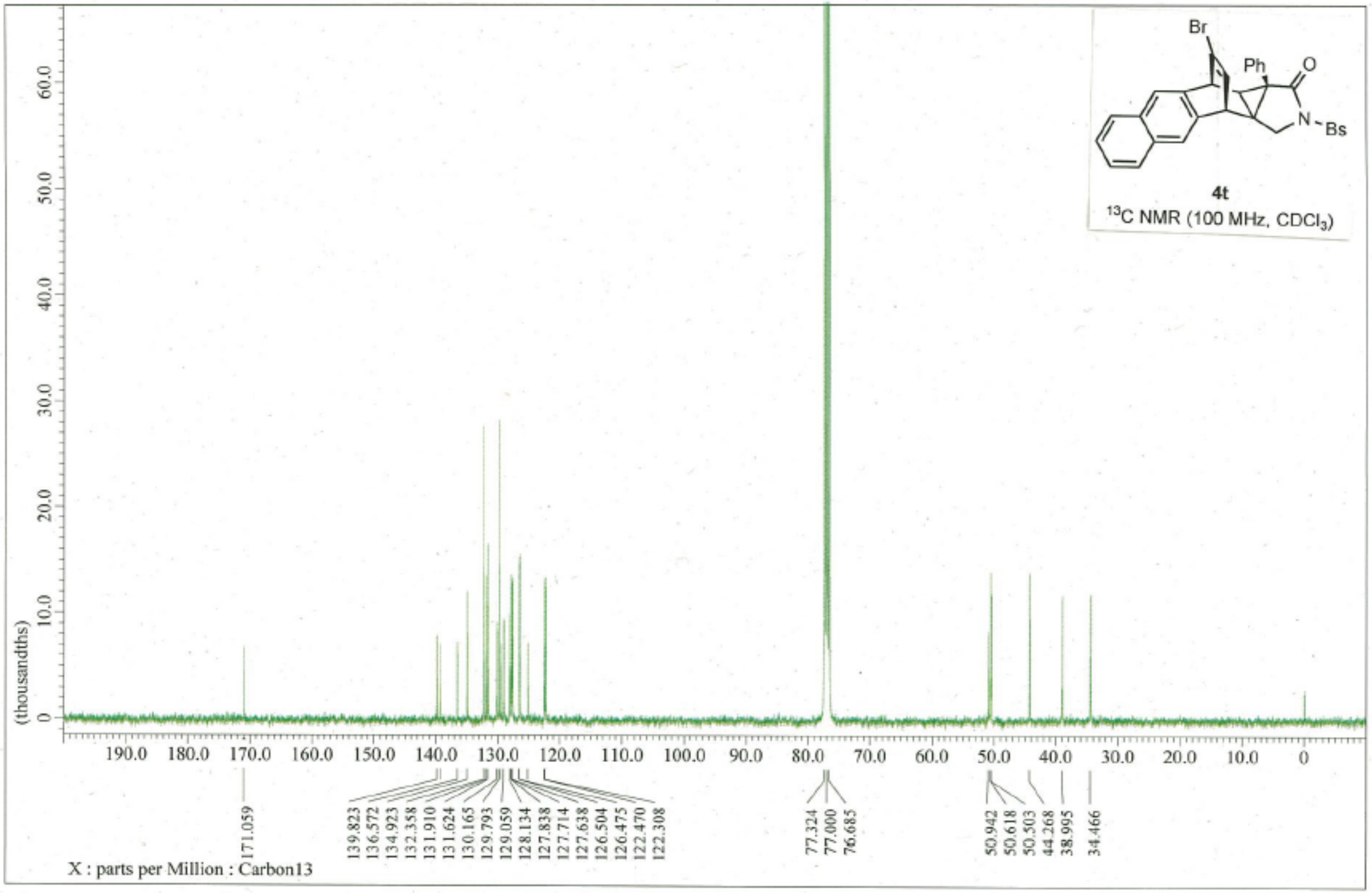
Br
Ph
O
N
Bs
4t
^{13}C NMR (100 MHz, CDCl$_3$)
(thousandths)
X : parts per Million : Carbon13
171.059
139.823
136.572
134.923
132.358
131.910
131.624
130.165
129.793
129.059
128.134
127.838
127.714
127.638
126.504
126.475
122.470
122.308
77.324
77.000
76.685
50.942
50.618
50.503
44.268
38.995
34.466

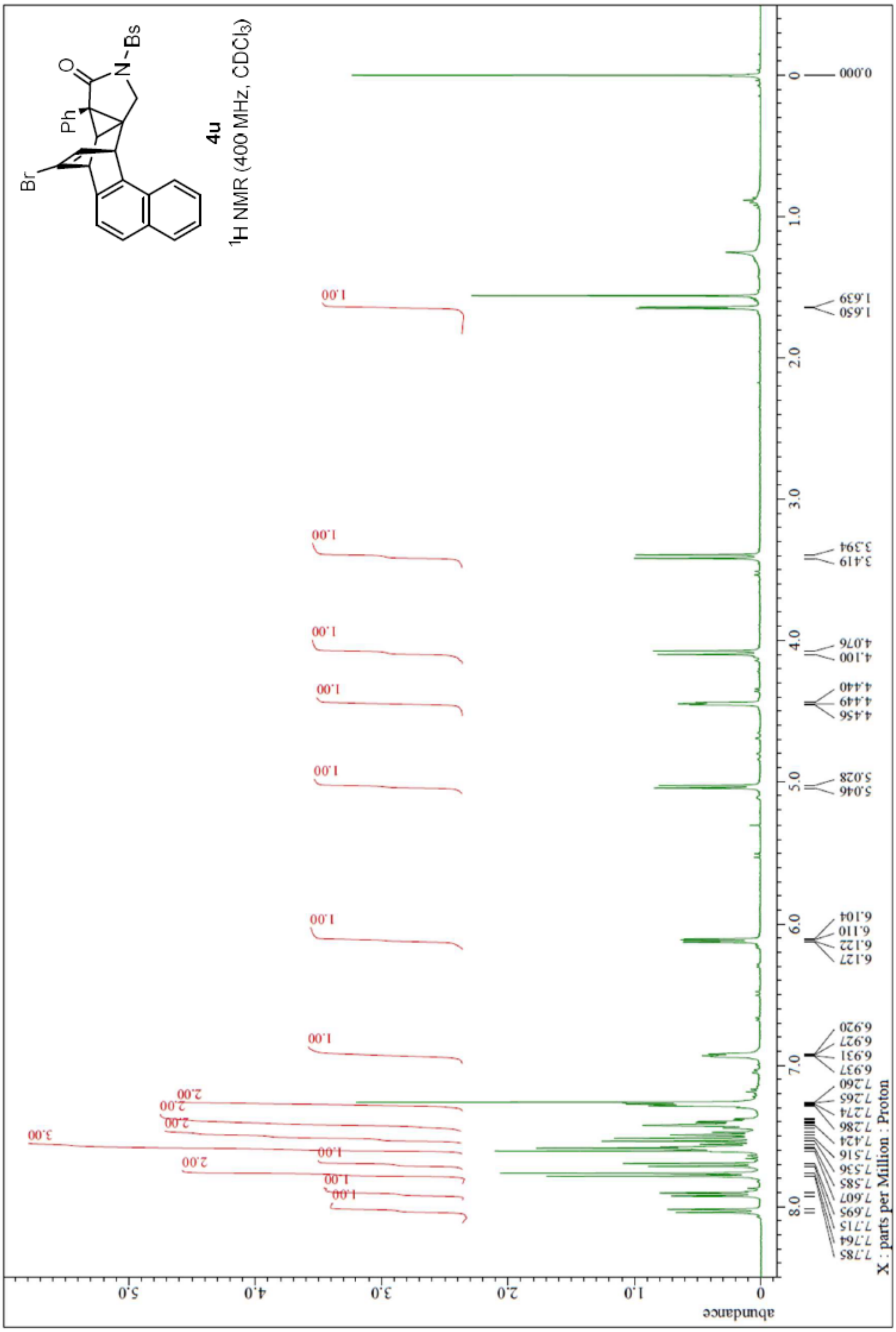

4u
1H NMR (400 MHz, CDCl3)
X : parts per Million : Proton
abundance

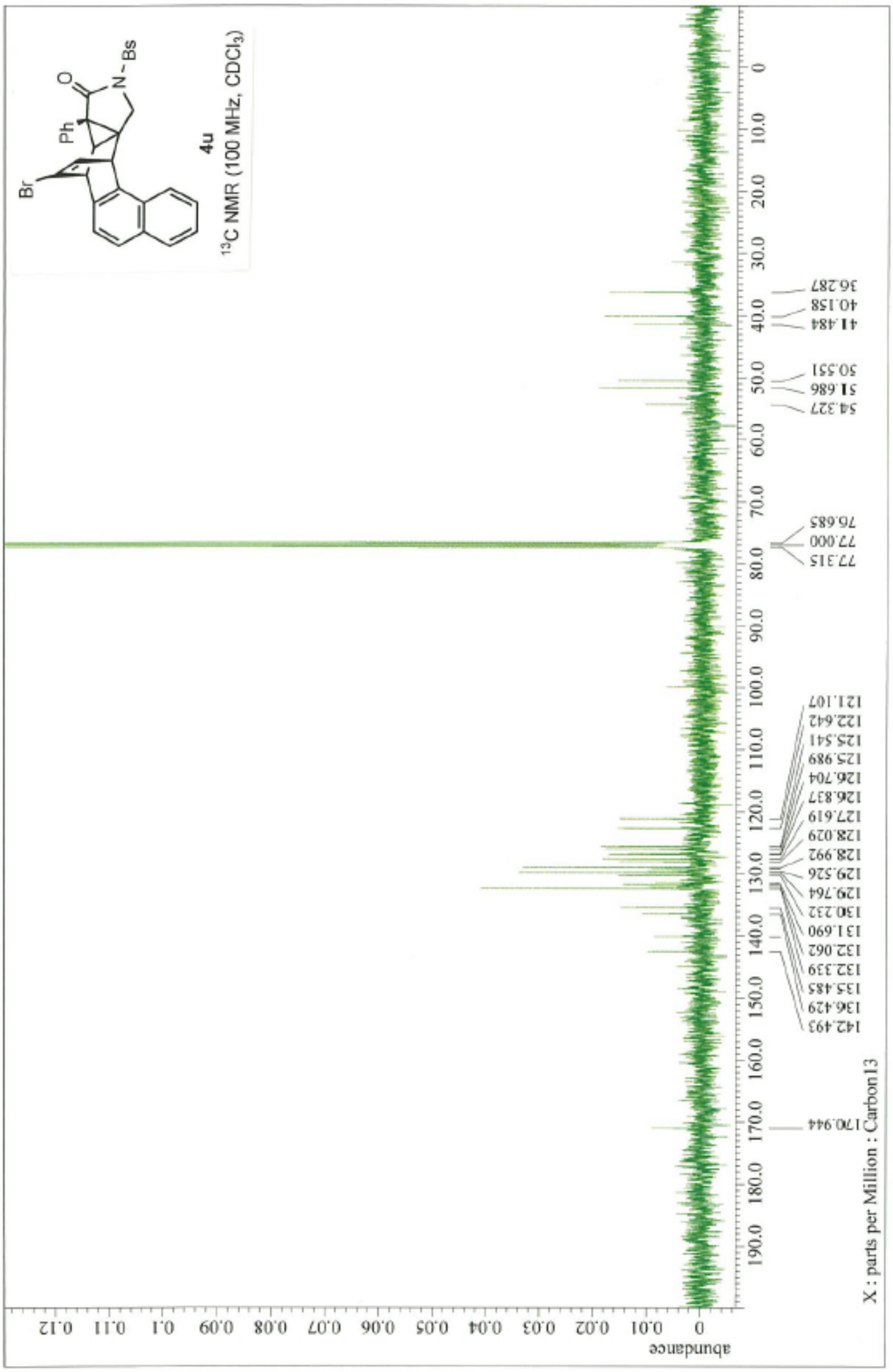

4u
13C NMR (100 MHz, CDCl3)
Br
Ph
O
N-Bs
36.287
40.158
41.484
50.551
51.686
54.327
76.685
77.000
77.315
121.107
122.642
125.541
125.989
126.704
126.837
127.619
128.029
128.992
129.526
129.764
130.232
131.690
132.062
132.339
135.485
136.429
142.493
170.944
X : parts per Million : Carbon13
abundance

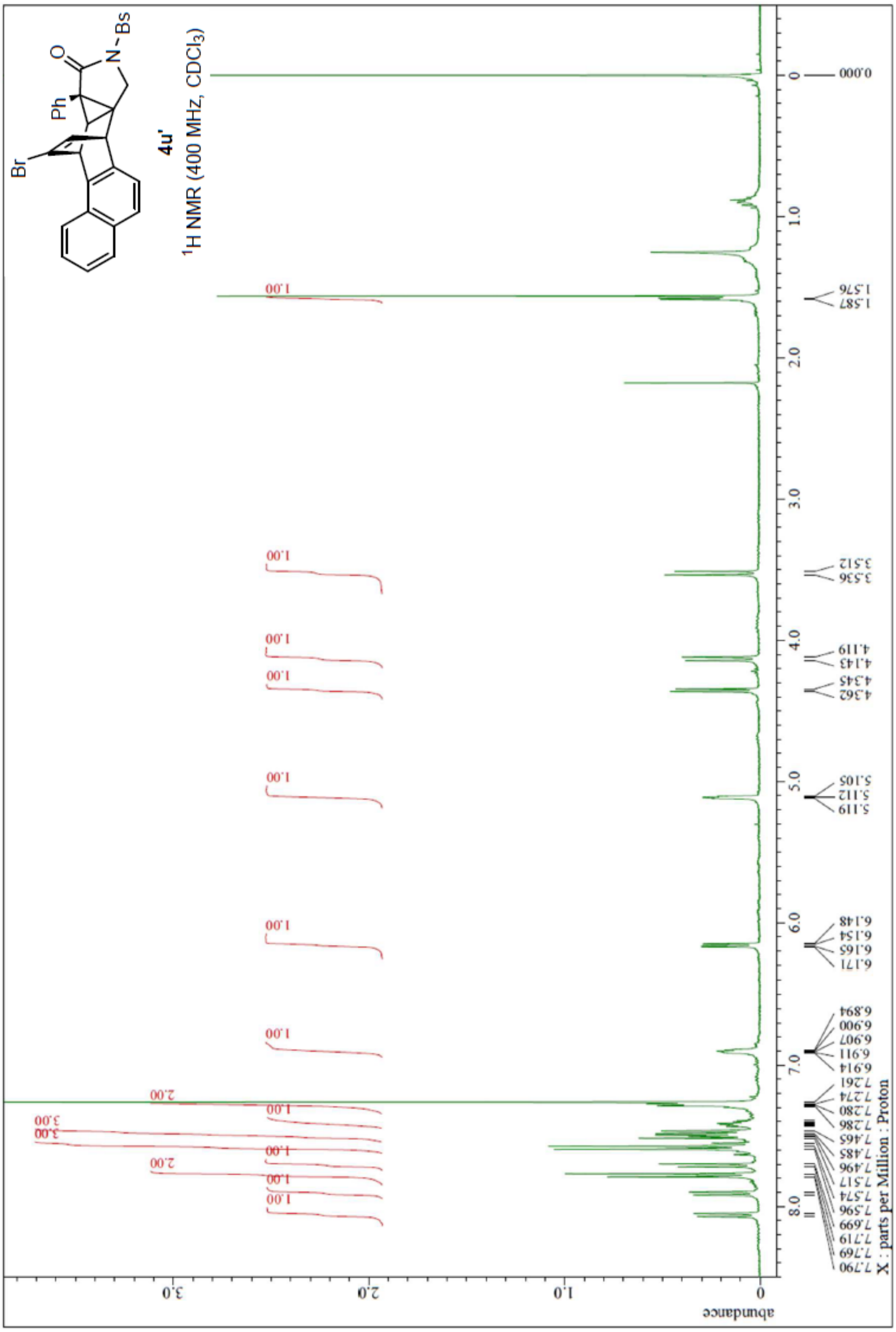

4u'
^{1}H NMR (400 MHz, CDCl$_3$)
Br
Ph
O
N-Bs
X : parts per Million : Proton
abundance

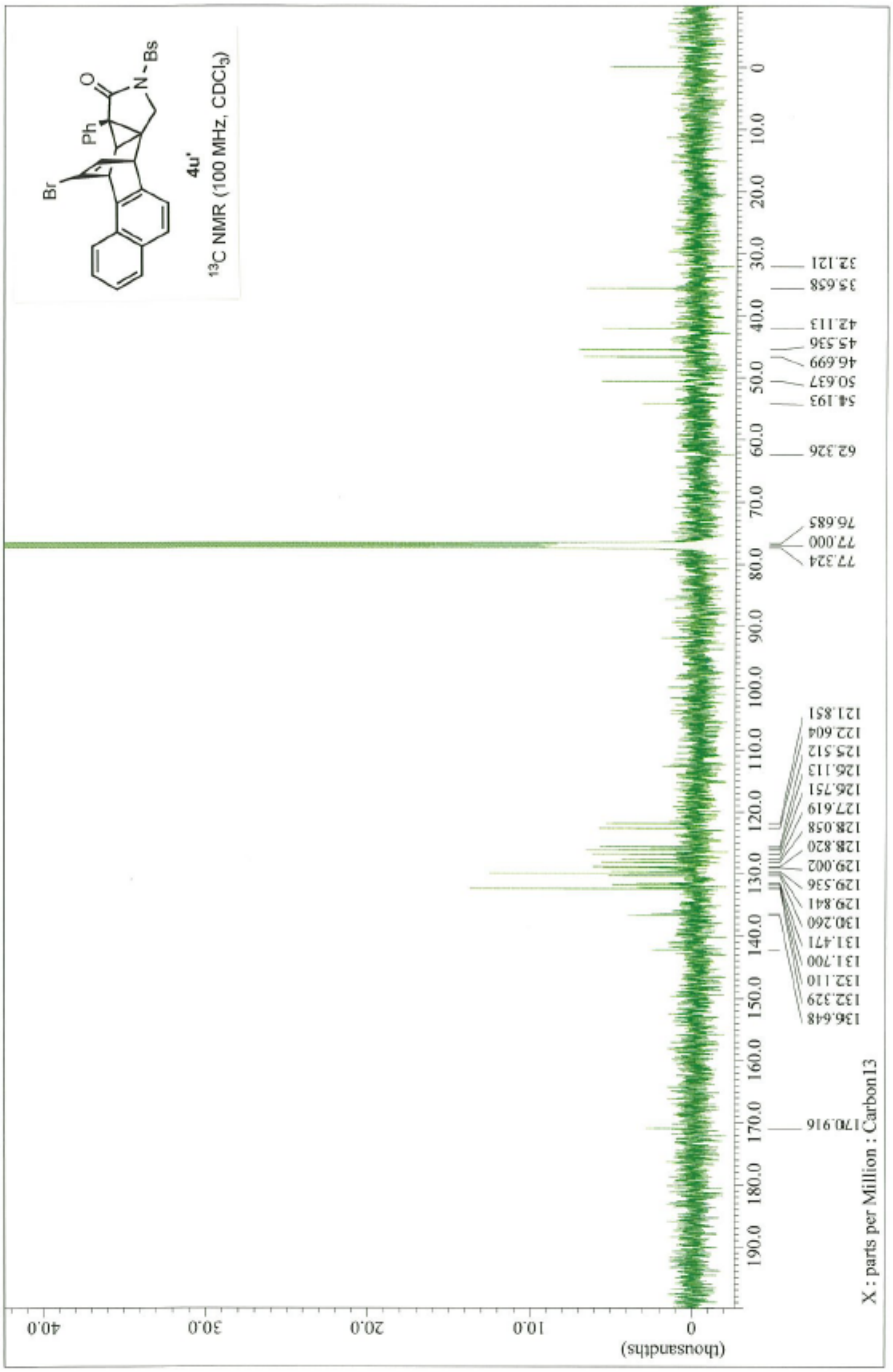
Br
Ph
O
N~Bs
4u'
^{13}C NMR (100 MHz, CDCl$_3$)
32.121
35.658
42.113
45.536
46.699
50.637
54.193
62.326
76.685
77.000
77.324
121.851
122.604
125.512
126.113
126.751
127.619
128.058
128.820
129.002
129.536
129.841
130.260
131.471
131.700
132.110
132.329
136.648
170.916
X : parts per Million : Carbon13
(thousandths)

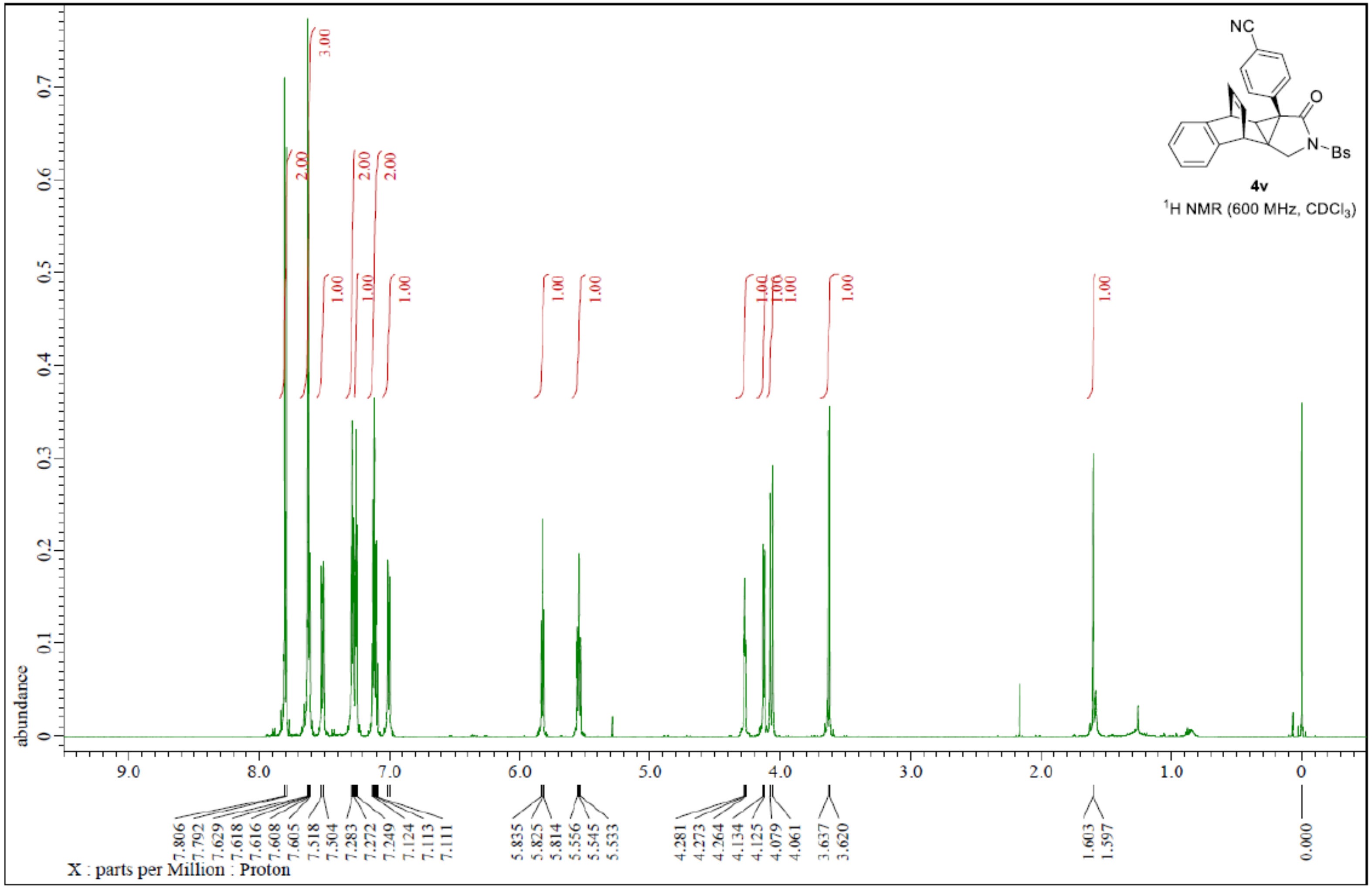
NC
O
N~Bs
4v
1H NMR (600 MHz, CDCl3)
abundance
X : parts per Million : Proton
7.806
7.792
7.629
7.618
7.616
7.608
7.605
7.518
7.504
7.283
7.272
7.249
7.124
7.113
7.111
5.835
5.825
5.814
5.556
5.545
5.533
4.281
4.273
4.264
4.134
4.125
4.079
4.061
3.637
3.620
1.603
1.597
0.000
3.00
2.00
2.00
2.00
1.00
1.00
1.00
1.00
1.00
1.00
1.00
1.00
1.00
1.00

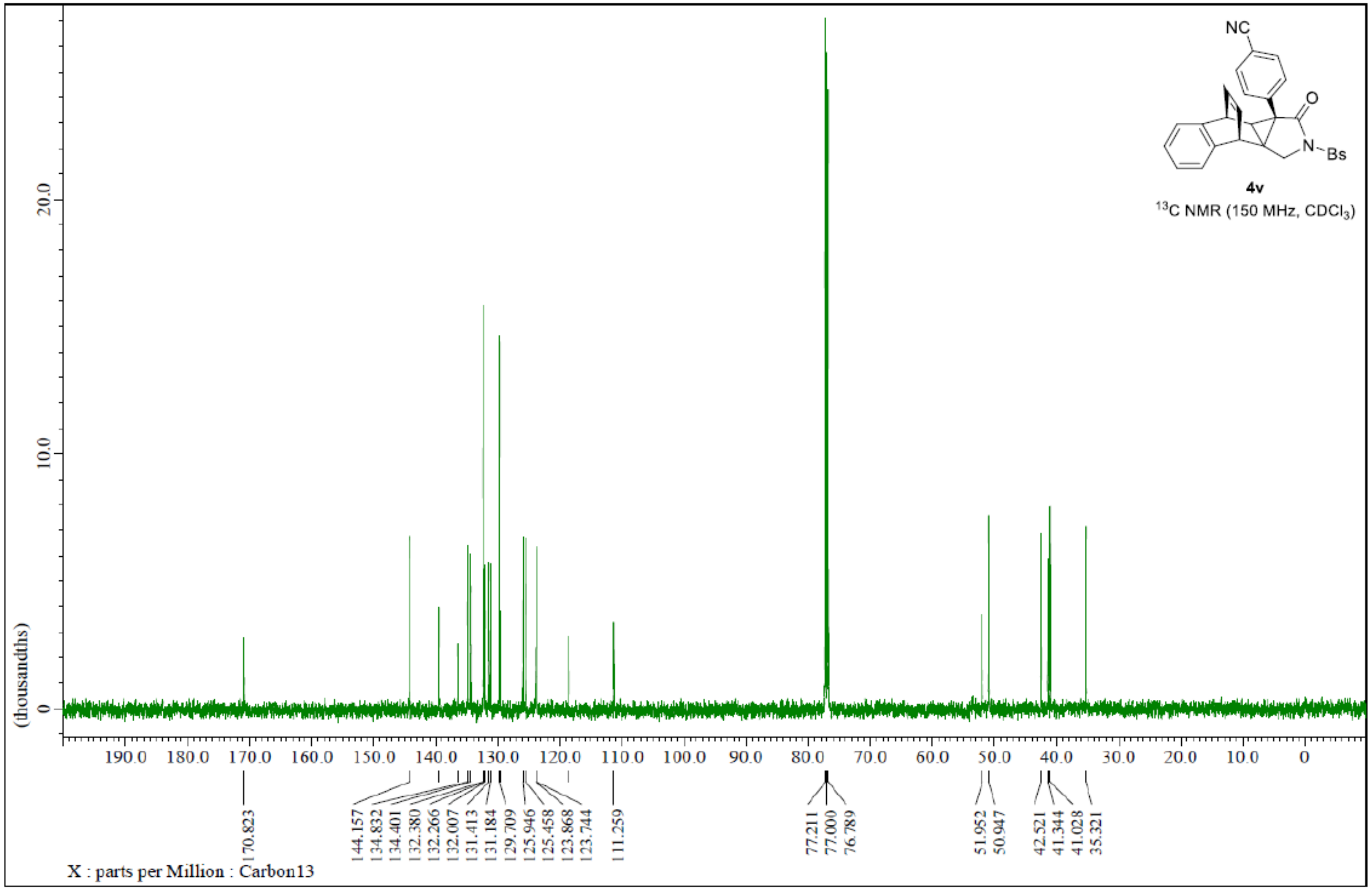
NC
O
N
Bs
4v
^{13}C NMR (150 MHz, CDCl$_3$)
20.0
10.0
0
(thousandths)
190.0 180.0 170.0 160.0 150.0 140.0 130.0 120.0 110.0 100.0 90.0 80.0 70.0 60.0 50.0 40.0 30.0 20.0 10.0 0
170.823
144.157
134.832
134.401
132.380
132.266
132.007
131.413
131.184
129.709
125.946
125.458
123.868
123.744
111.259
77.211
77.000
76.789
51.952
50.947
42.521
41.344
41.028
35.321
X : parts per Million : Carbon13

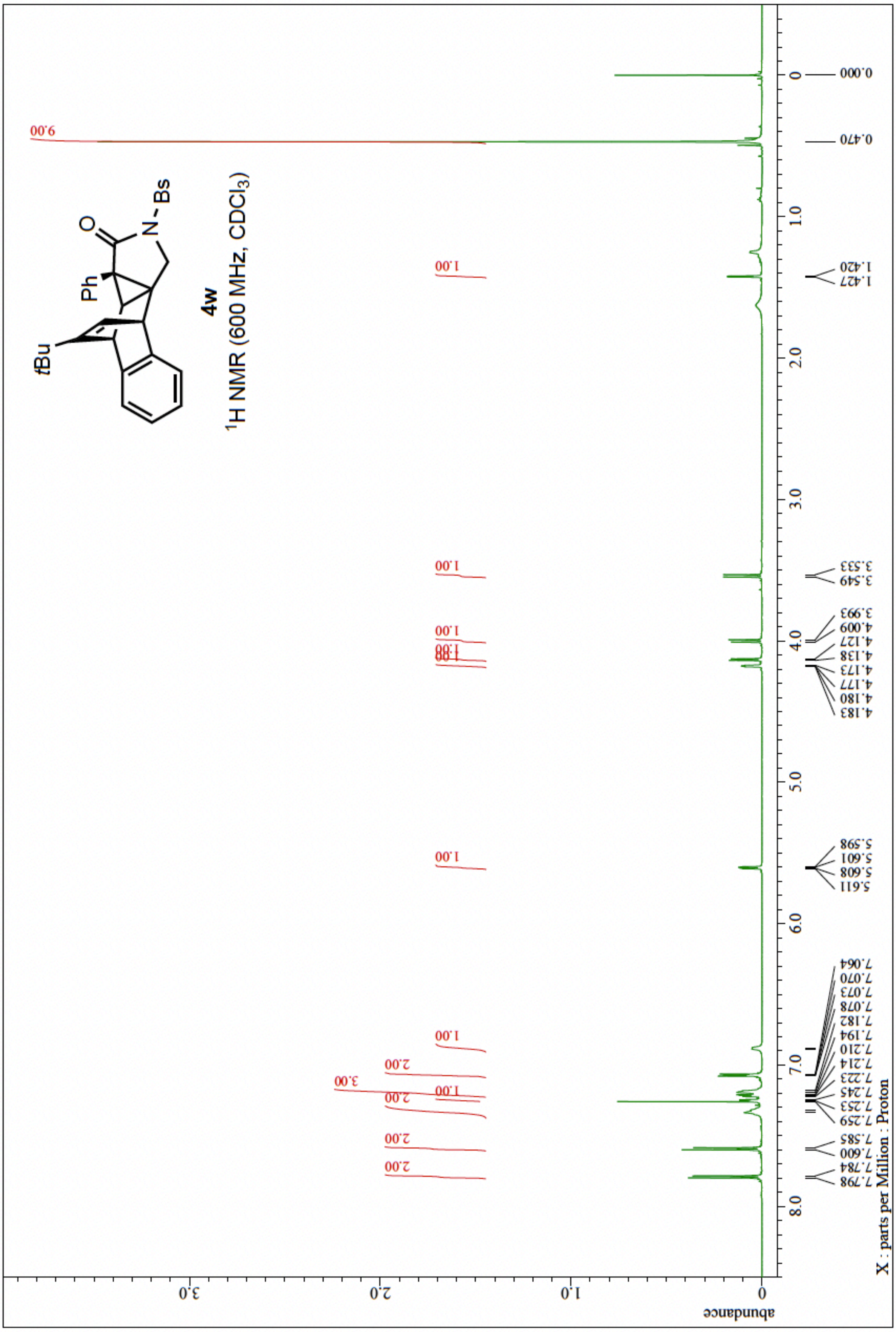

tBu
Ph
O
N
Bs
4w
1H NMR (600 MHz, CDCl3)
0.000
0.470
1.420
1.427
3.533
3.549
3.993
4.009
4.127
4.138
4.173
4.177
4.180
4.183
5.598
5.601
5.608
5.611
7.064
7.070
7.073
7.078
7.182
7.194
7.210
7.214
7.223
7.245
7.253
7.259
7.585
7.600
7.784
7.798
9.00
1.00
3.00
2.00
X : parts per Million : Proton
abundance

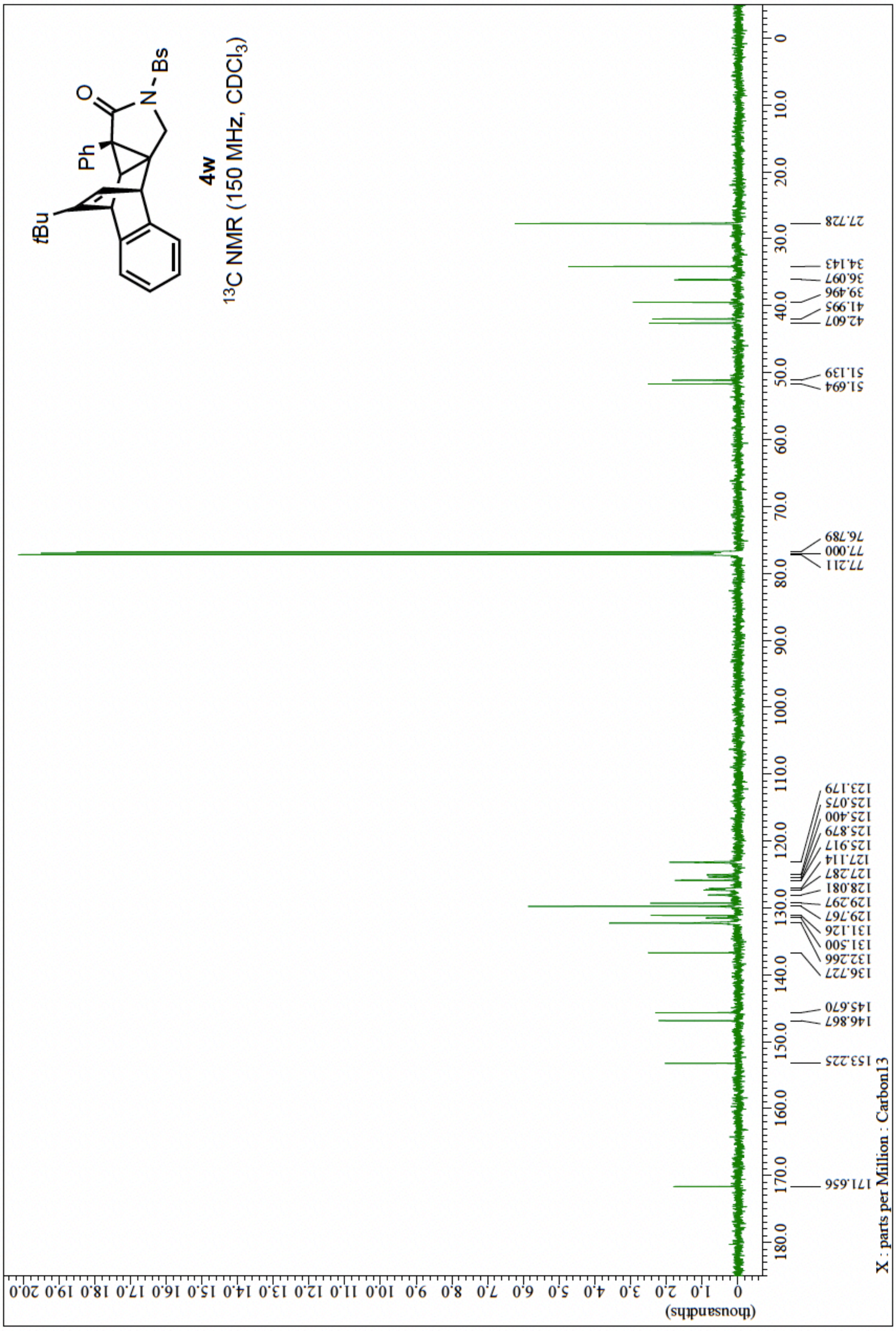
tBu
Ph
O
N
Bs
4w
^{13}C NMR (150 MHz, CDCl$_3$)
27.728
34.143
36.097
39.496
41.995
42.607
51.139
51.694
76.789
77.000
77.211
123.179
125.075
125.400
125.879
125.917
127.114
127.287
128.081
129.297
129.767
131.126
131.500
132.266
136.727
145.670
146.867
153.225
171.656
X : parts per Million : Carbon13
(thousandths)

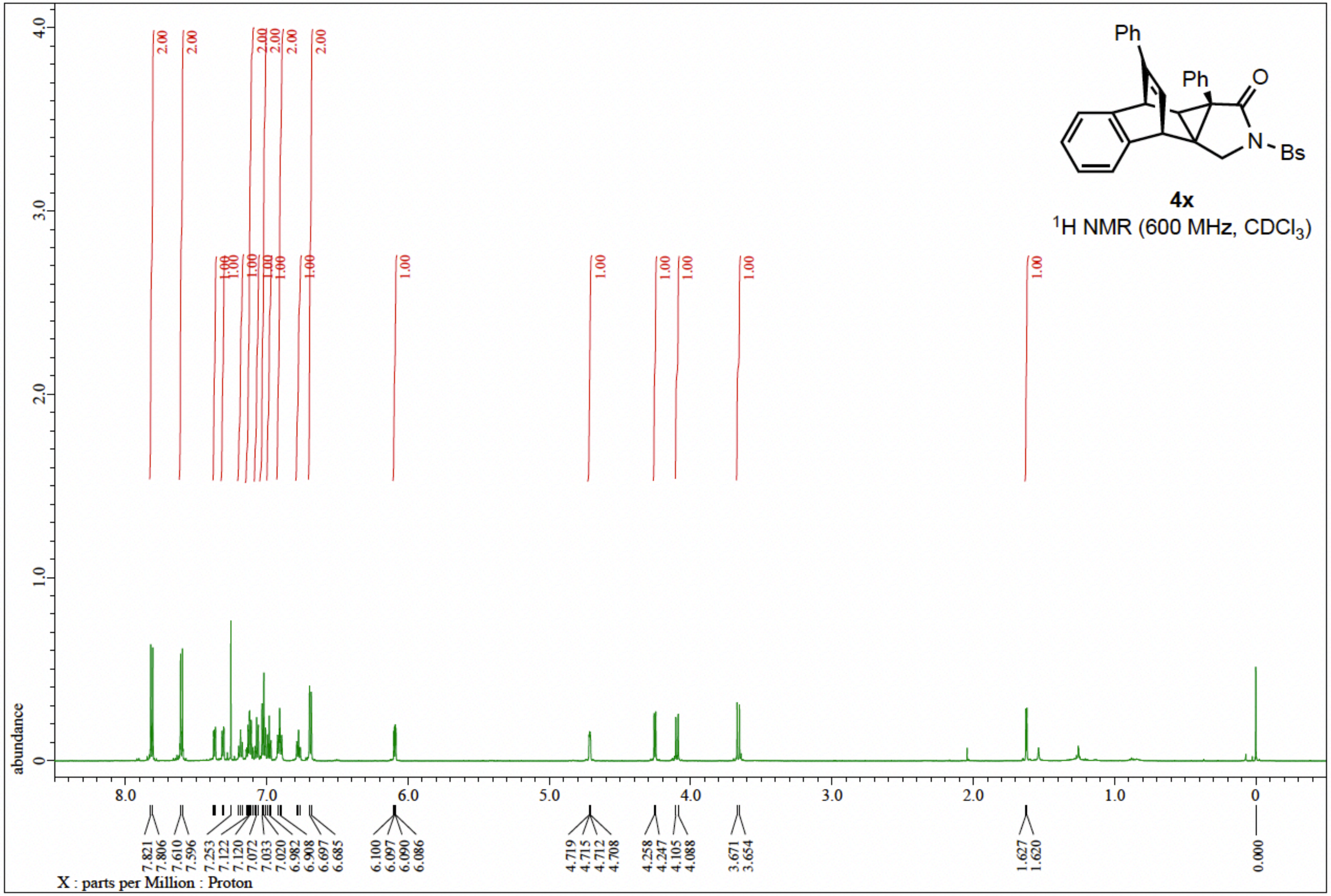
Ph
Ph
O
N
Bs
4x
^1H NMR (600 MHz, $CDCl_3$)
abundance
X : parts per Million : Proton
7.821 7.806 7.610 7.596 7.253 7.122 7.120 7.072 7.033 7.020 6.982 6.908 6.697 6.685
6.100 6.097 6.090 6.086
4.719 4.715 4.712 4.708
4.258 4.247 4.105 4.088
3.671 3.654
1.627 1.620
0.000

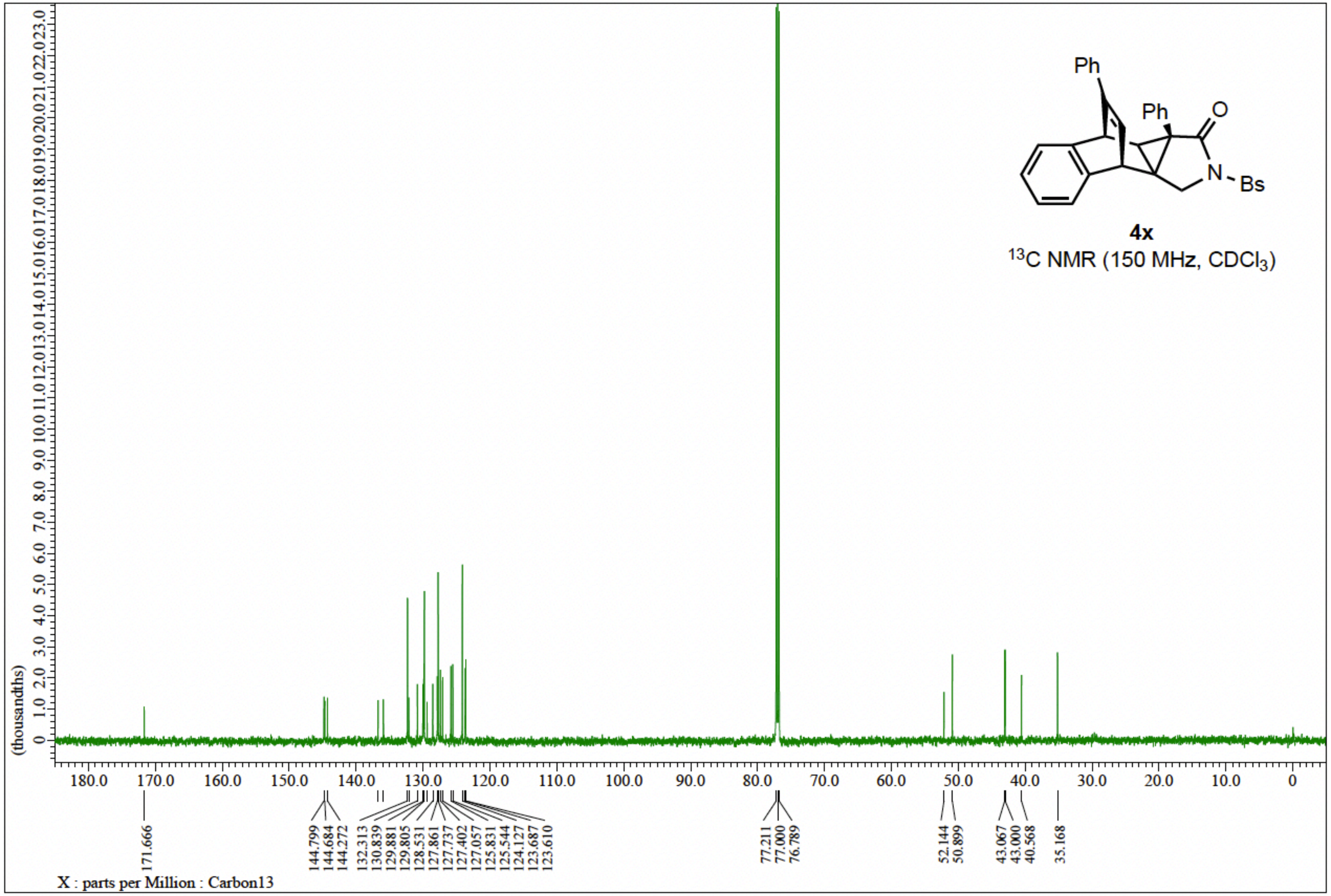
Ph
Ph
O
N
Bs
4x
13C NMR (150 MHz, CDCl3)
(thousandths)
0
1.0
2.0
3.0
4.0
5.0
6.0
7.0
8.0
9.0
10.0
11.0
12.0
13.0
14.0
15.0
16.0
17.0
18.0
19.0
20.0
21.0
22.0
23.0
180.0
170.0
160.0
150.0
140.0
130.0
120.0
110.0
100.0
90.0
80.0
70.0
60.0
50.0
40.0
30.0
20.0
10.0
0
171.666
144.799
144.684
144.272
132.313
130.839
129.881
129.805
128.531
127.861
127.737
127.402
127.057
125.831
125.544
124.127
123.687
123.610
77.211
77.000
76.789
52.144
50.899
43.067
43.000
40.568
35.168
X : parts per Million : Carbon13

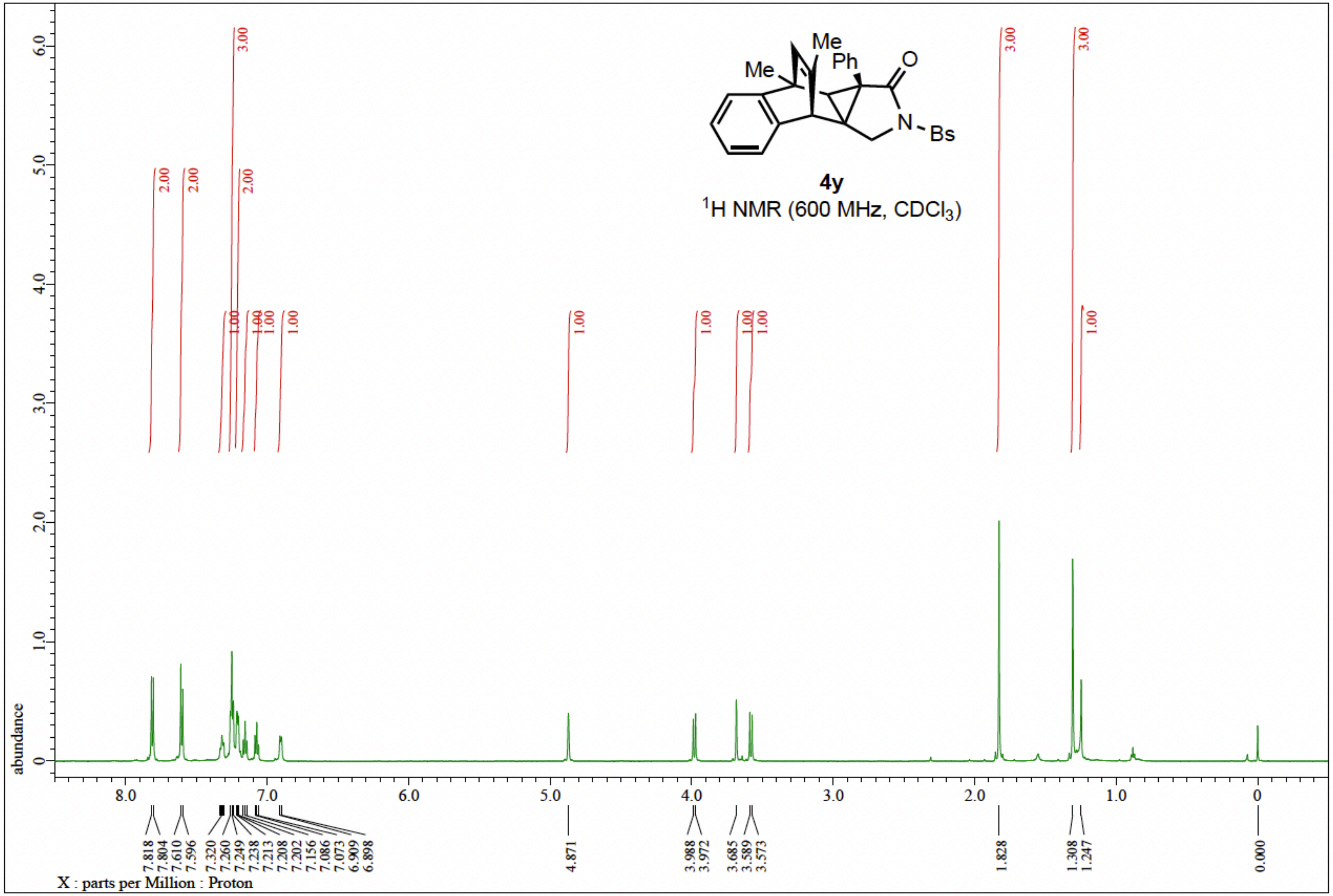

4y
^{1}H NMR (600 MHz, CDCl$_3$)
Me
Me
Ph
O
N
Bs
abundance
X : parts per Million : Proton
7.818
7.804
7.610
7.596
7.320
7.260
7.249
7.238
7.213
7.208
7.202
7.156
7.086
7.073
6.909
6.898
4.871
3.988
3.972
3.685
3.589
3.573
1.828
1.308
1.247
0.000
2.00
2.00
3.00
2.00
1.00
1.00
1.00
1.00
1.00
1.00
1.00
1.00
1.00
3.00
3.00
1.00

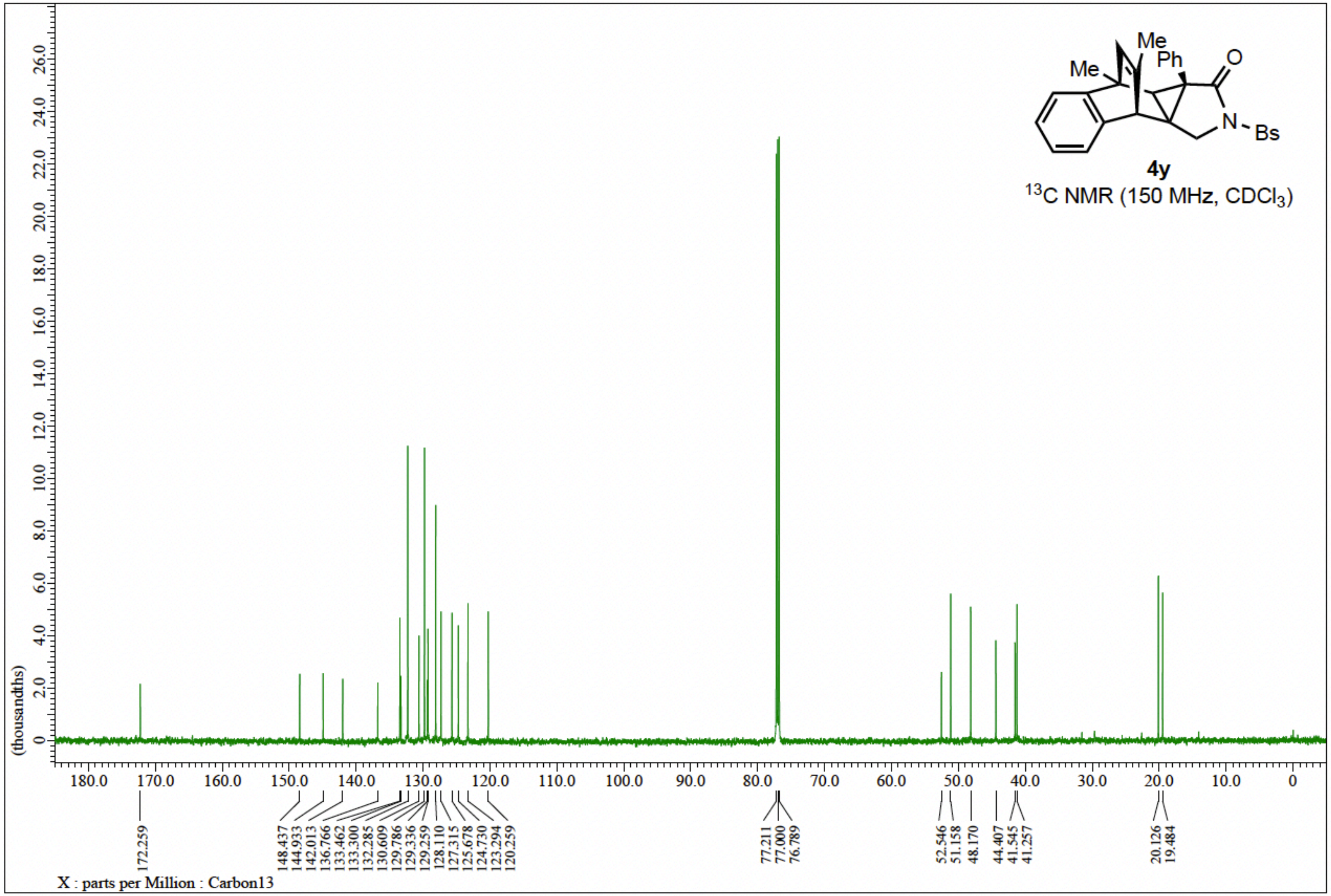
Me
Me
Ph
O
N
Bs
4y
13C NMR (150 MHz, CDCl3)
(thousandths)
X : parts per Million : Carbon13
172.259
148.437
144.933
142.013
136.766
133.462
133.300
132.285
130.609
129.786
129.336
129.259
128.110
127.315
125.678
124.730
123.294
120.259
77.211
77.000
76.789
52.546
51.158
48.170
44.407
41.545
41.257
20.126
19.484

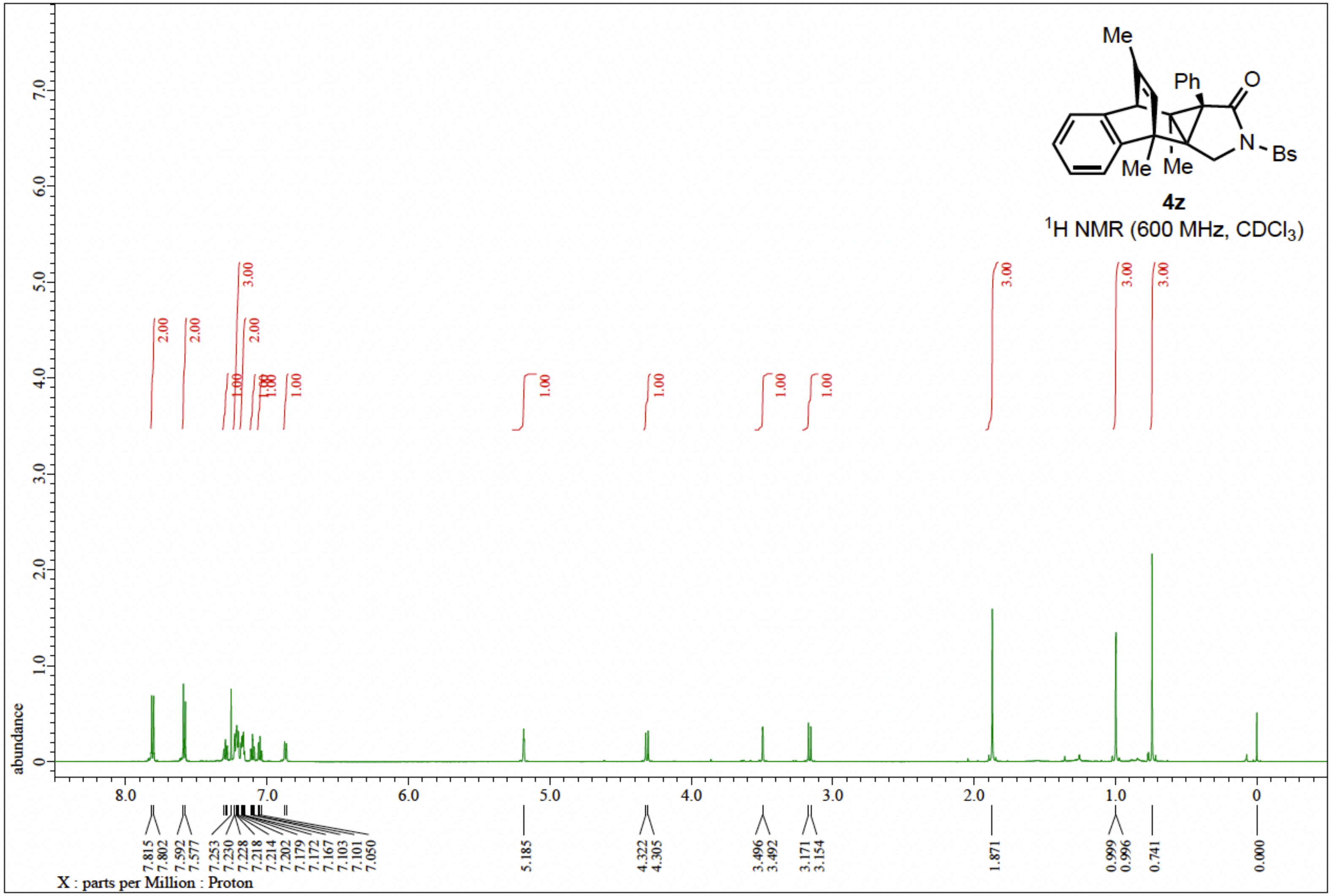
Me
Ph
O
N
Bs
Me
Me
4z
¹H NMR (600 MHz, CDCl3)
2.00
2.00
3.00
2.00
1.00
1.00
1.00
1.00
1.00
1.00
1.00
3.00
3.00
3.00
7.815
7.802
7.592
7.577
7.253
7.230
7.228
7.218
7.214
7.202
7.179
7.172
7.167
7.103
7.101
7.050
5.185
4.322
4.305
3.496
3.492
3.171
3.154
1.871
0.999
0.996
0.741
0.000
abundance
X : parts per Million : Proton

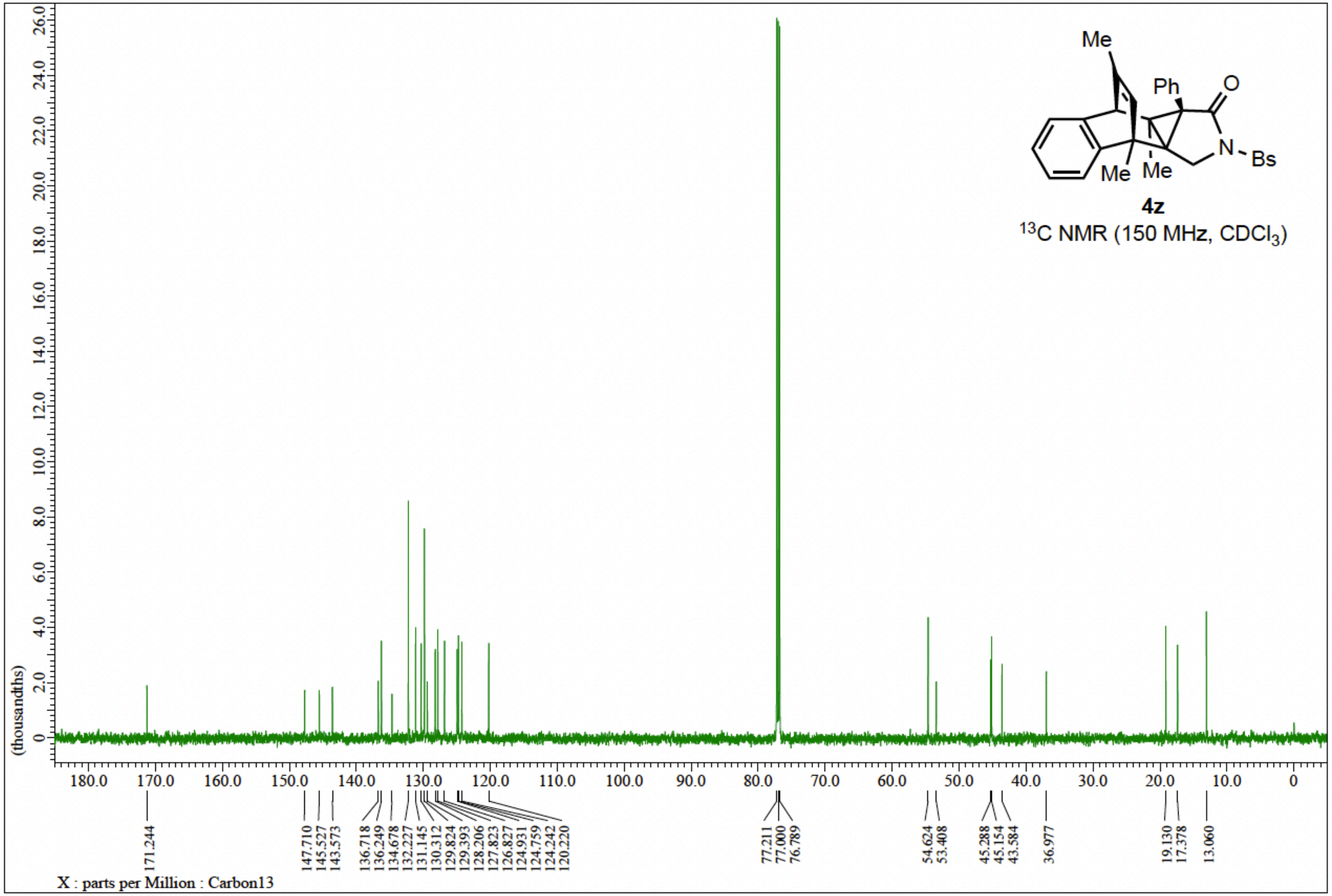
Me
Ph
O
N
Bs
Me
Me
4z
^{13}C NMR (150 MHz, $CDCl_3$)
171.244
147.710
145.527
143.573
136.718
136.249
134.678
132.227
131.145
130.312
129.824
129.393
128.206
127.823
126.827
124.931
124.759
124.242
120.220
77.211
77.000
76.789
54.624
53.408
45.288
45.154
43.584
36.977
19.130
17.378
13.060
(thousandths)
X : parts per Million : Carbon13

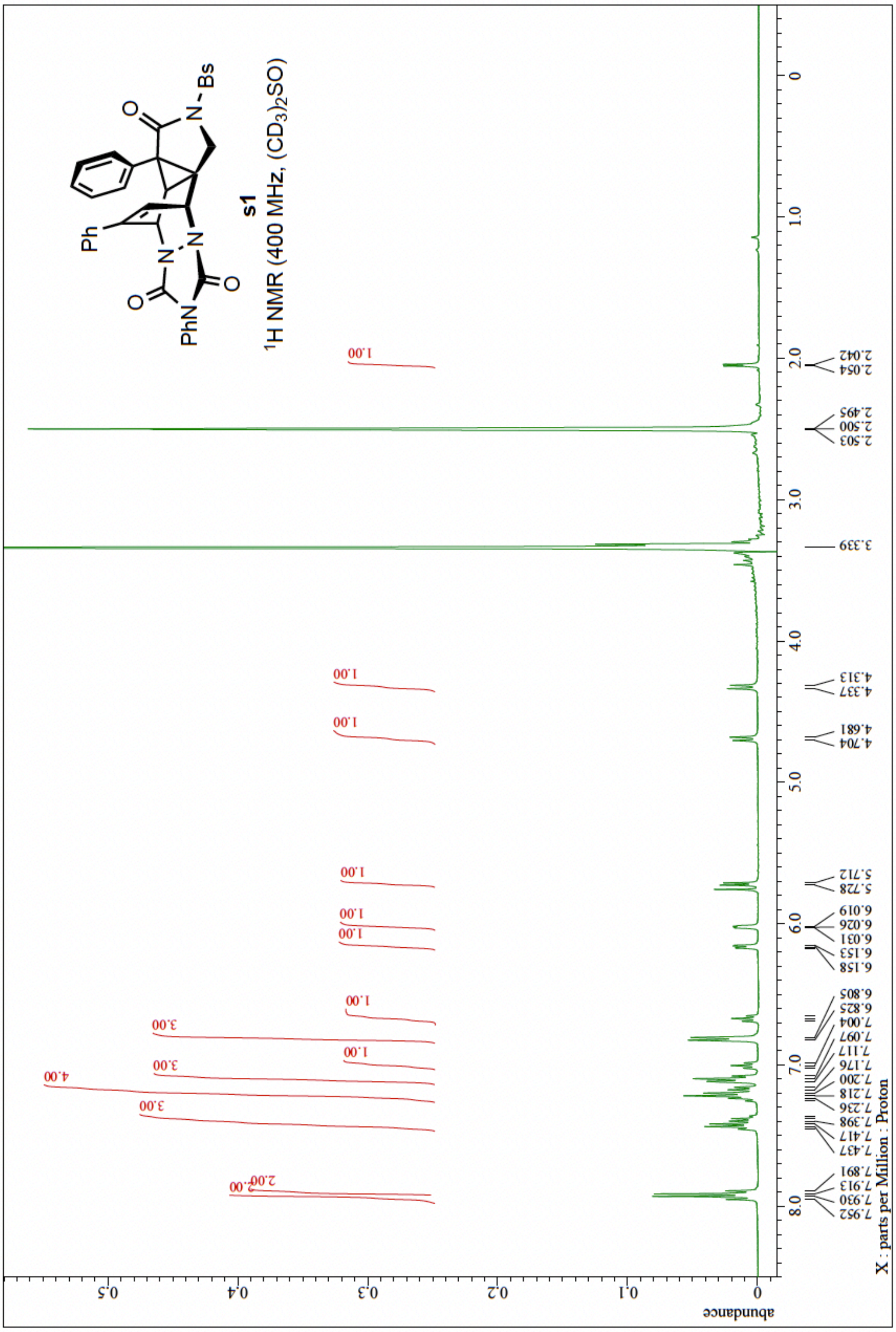
s1
1H NMR (400 MHz, (CD3)2SO)
X : parts per Million : Proton
abundance

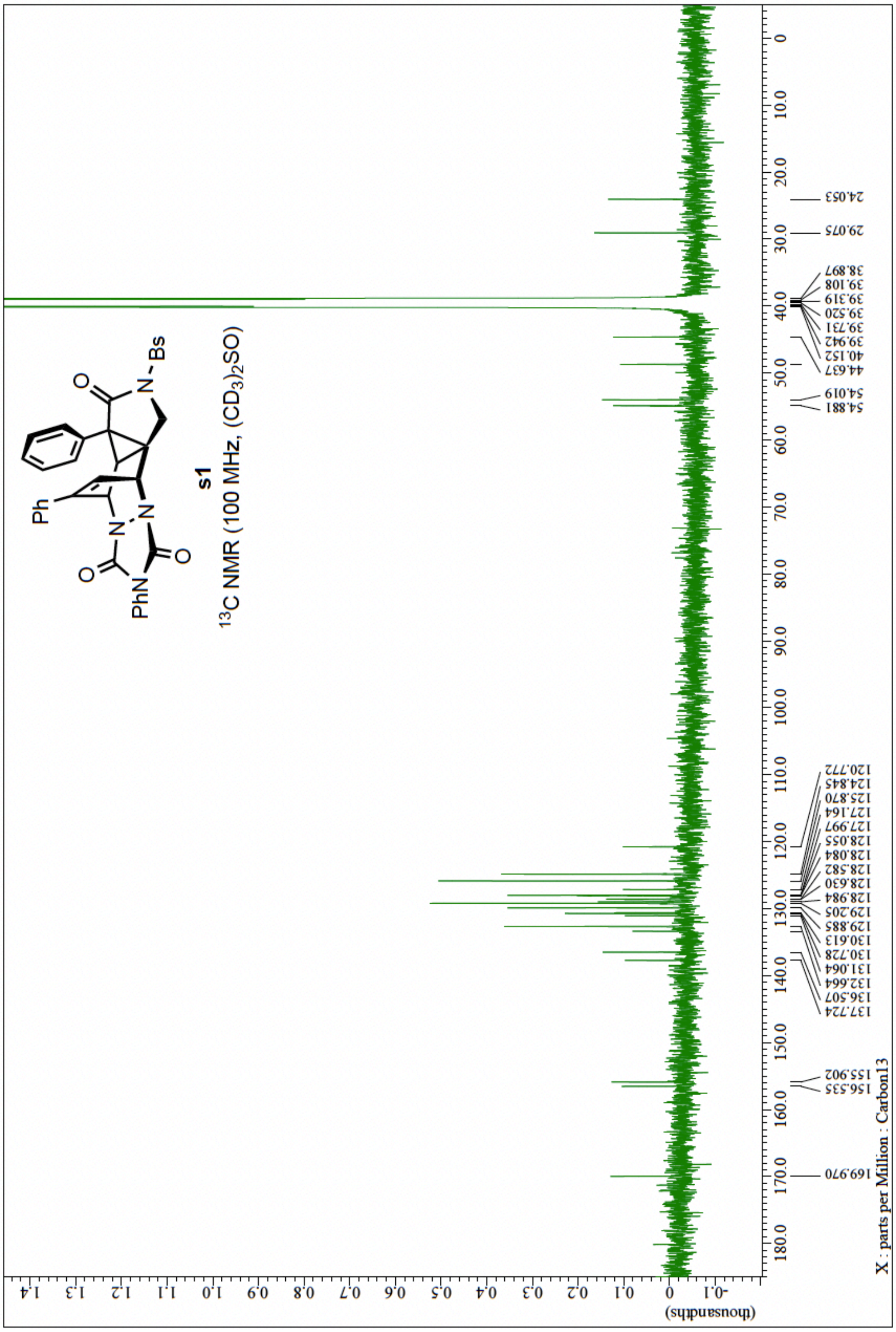

Ph
PhN
O
N
N
O
O
N
Bs
s1
^{13}C NMR (100 MHz, $(CD_3)_2SO$)
24.053
29.075
38.897
39.108
39.319
39.520
39.731
39.942
40.152
44.637
54.019
54.881
120.772
124.845
125.870
127.164
127.997
128.055
128.084
128.582
128.630
128.984
129.205
129.885
130.613
130.728
131.064
132.664
136.507
137.724
155.902
156.535
169.970
X : parts per Million : Carbon13
(thousandths)

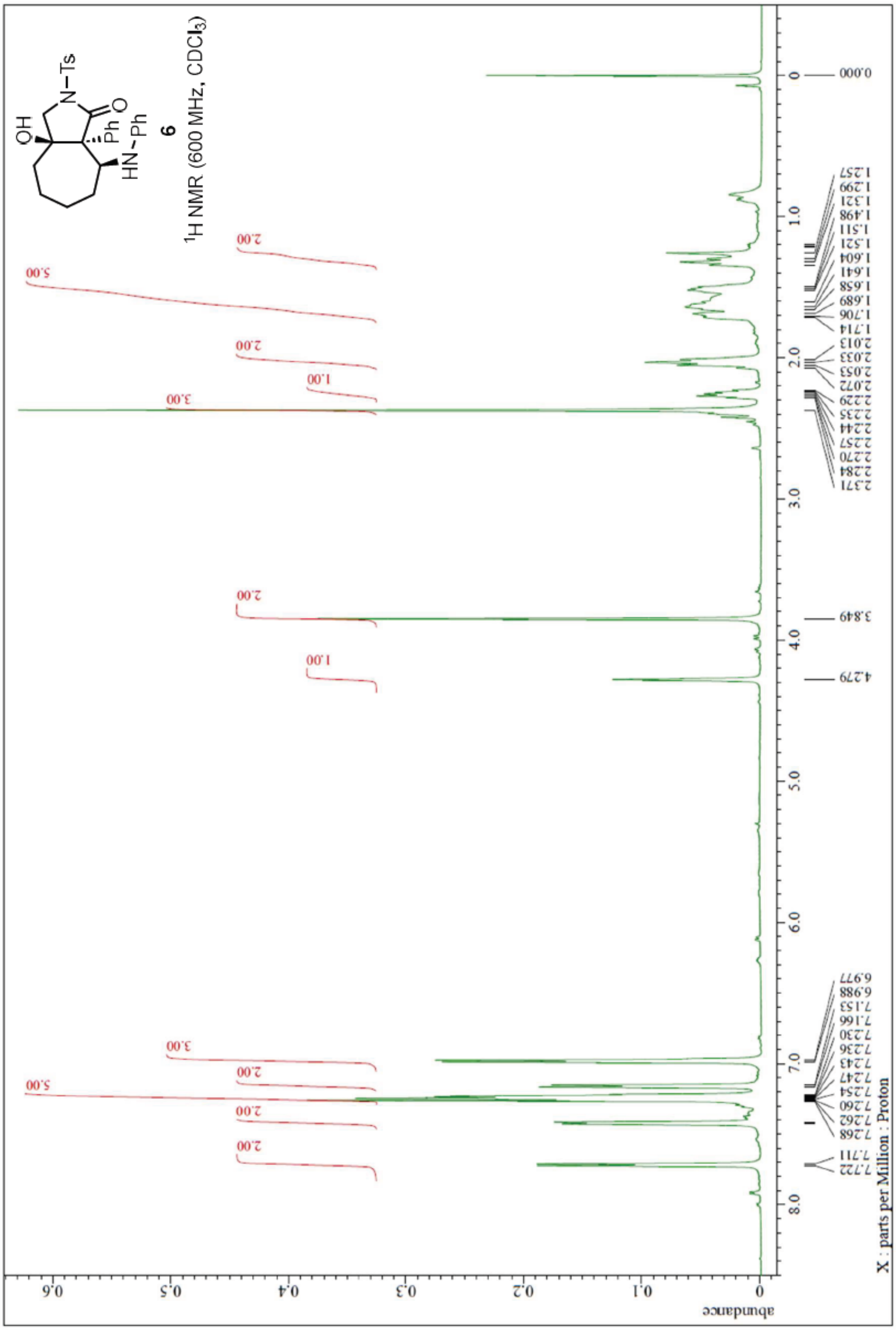
6
1H NMR (600 MHz, CDCl3)
X : parts per Million : Proton
abundance

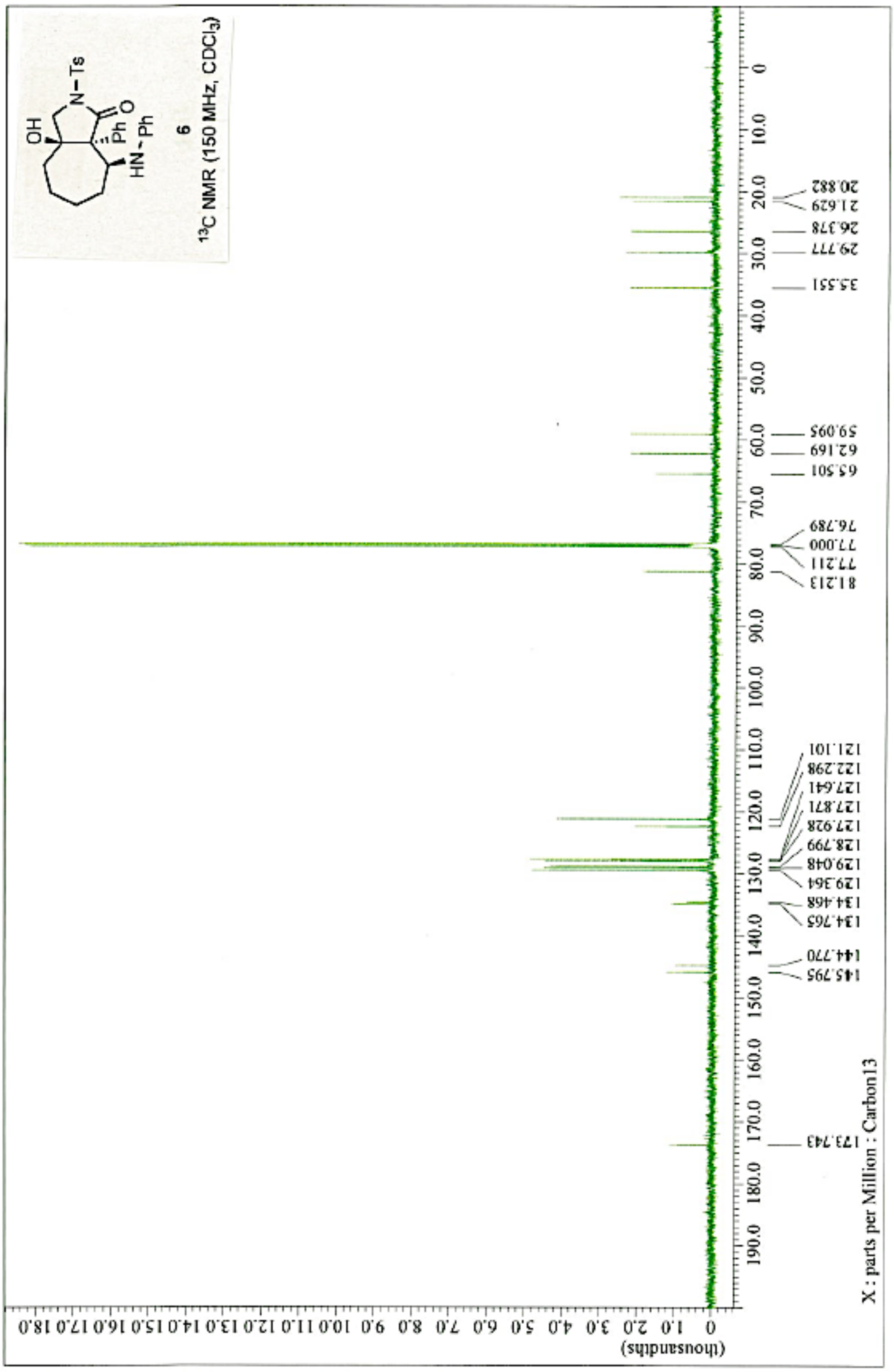

OH
N−Ts
O
Ph
HN
Ph
6
13C NMR (150 MHz, CDCl3)
20.882
21.629
26.378
29.777
35.551
59.095
62.169
65.501
76.789
77.000
77.211
81.213
121.101
122.298
127.641
127.871
127.928
128.799
129.048
129.364
134.468
134.765
144.770
145.795
173.743
X : parts per Million : Carbon13
(thousandths)

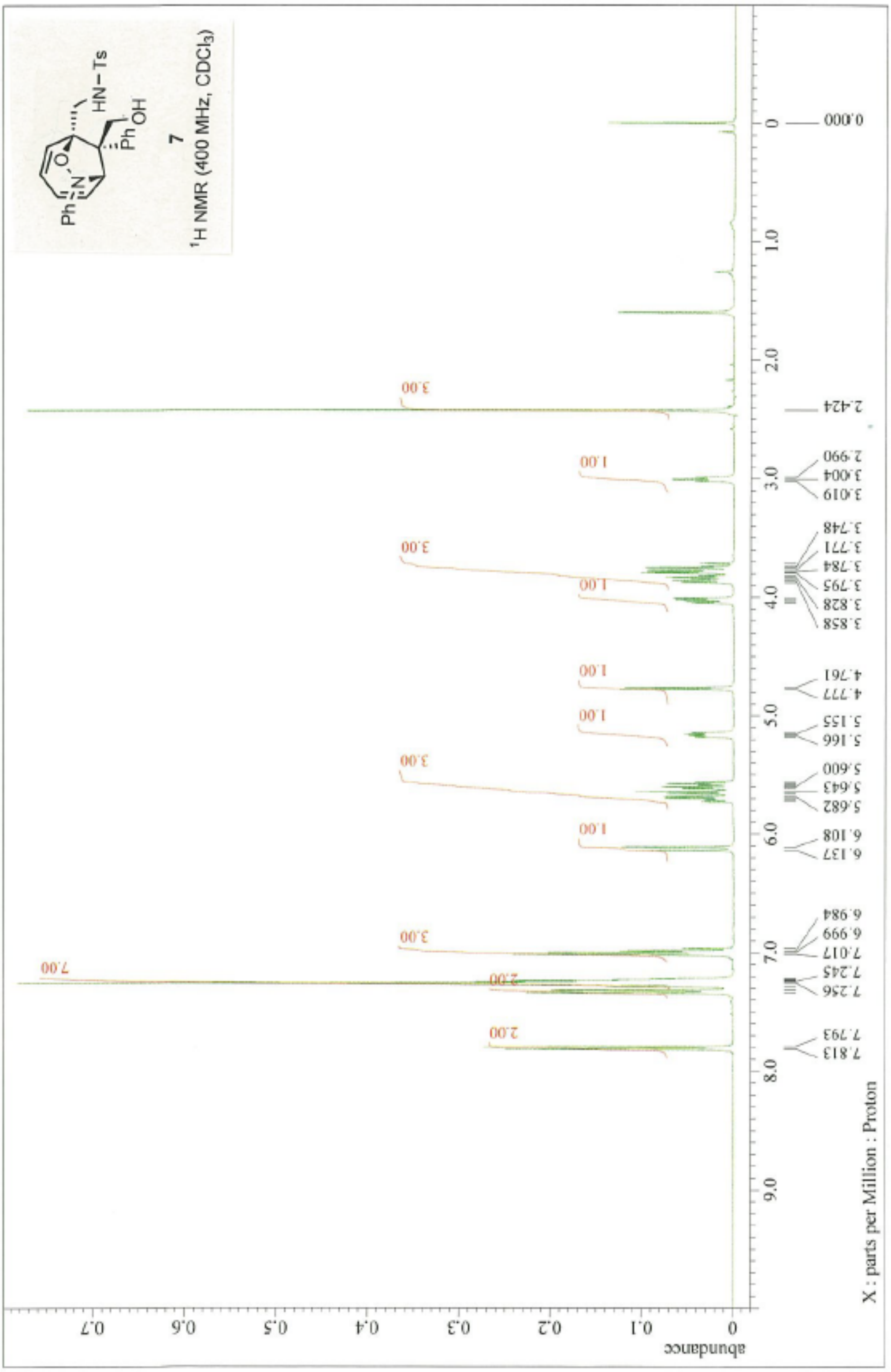
7
1H NMR (400 MHz, CDCl3)
X : parts per Million : Proton
abundance

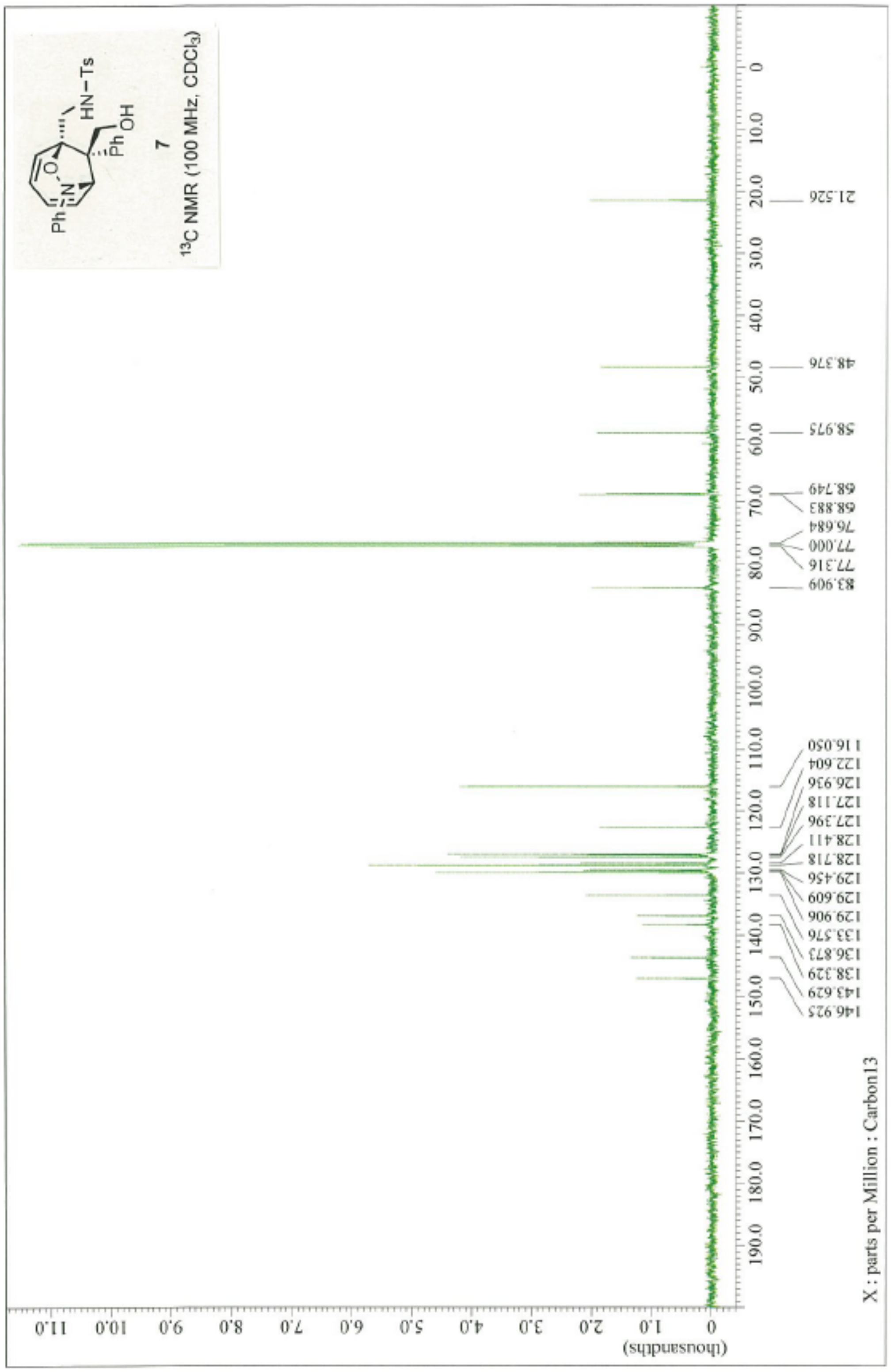
HN−Ts
Ph
OH
Ph
N
O
7
13C NMR (100 MHz, CDCl3)
21.526
48.376
58.975
68.749
68.883
76.684
77.000
77.316
83.909
116.050
122.604
126.936
127.118
127.396
128.411
128.718
129.456
129.609
129.906
133.576
136.873
138.329
143.629
146.925
X : parts per Million : Carbon13
(thousandths)

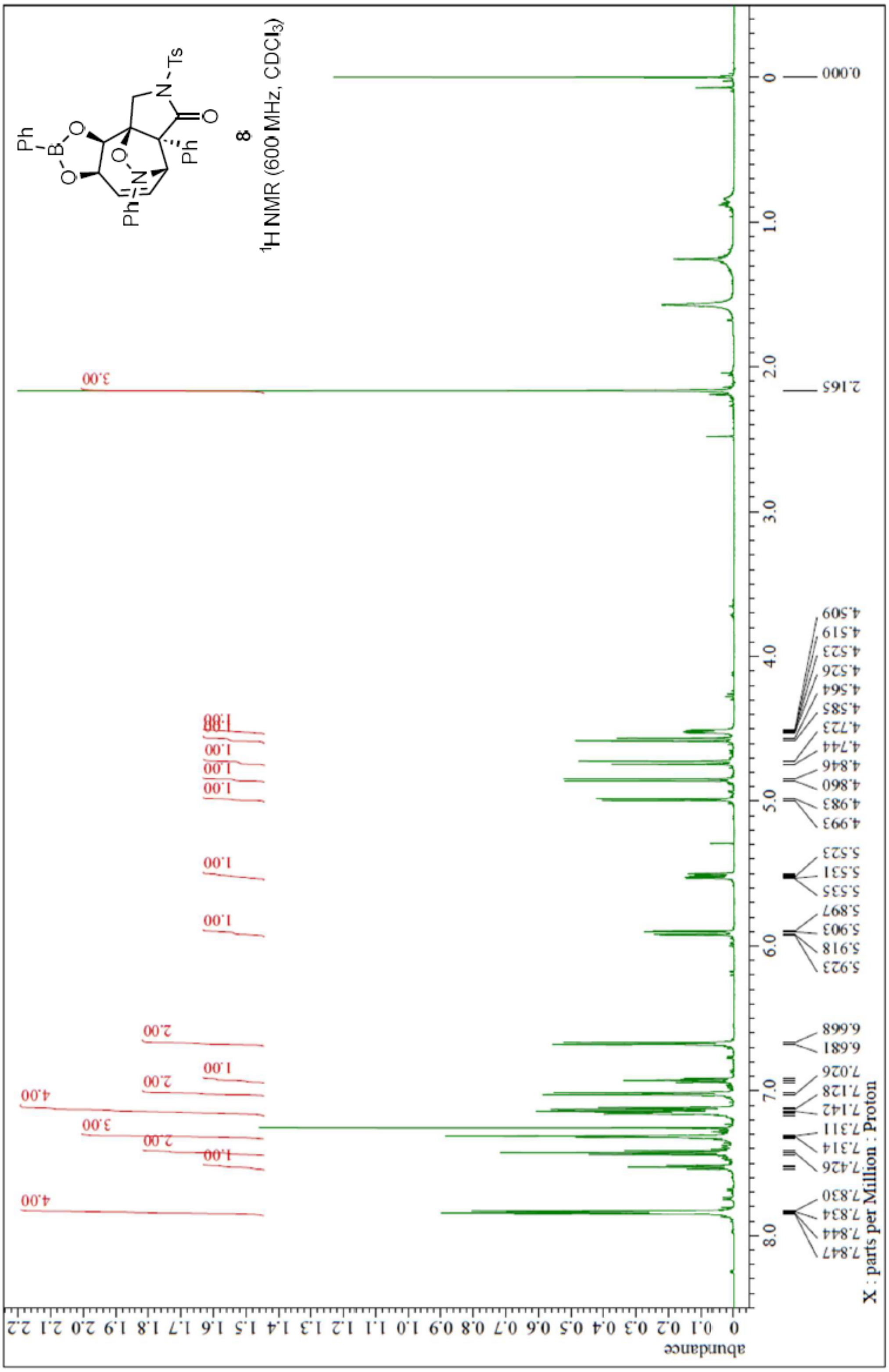
8
1H NMR (600 MHz, CDCl3)
X : parts per Million : Proton
abundance
0.000
2.165
4.509
4.519
4.523
4.526
4.564
4.585
4.723
4.744
4.846
4.860
4.983
4.993
5.523
5.531
5.535
5.897
5.903
5.918
5.923
6.668
6.681
7.026
7.128
7.142
7.311
7.314
7.426
7.830
7.834
7.844
7.847

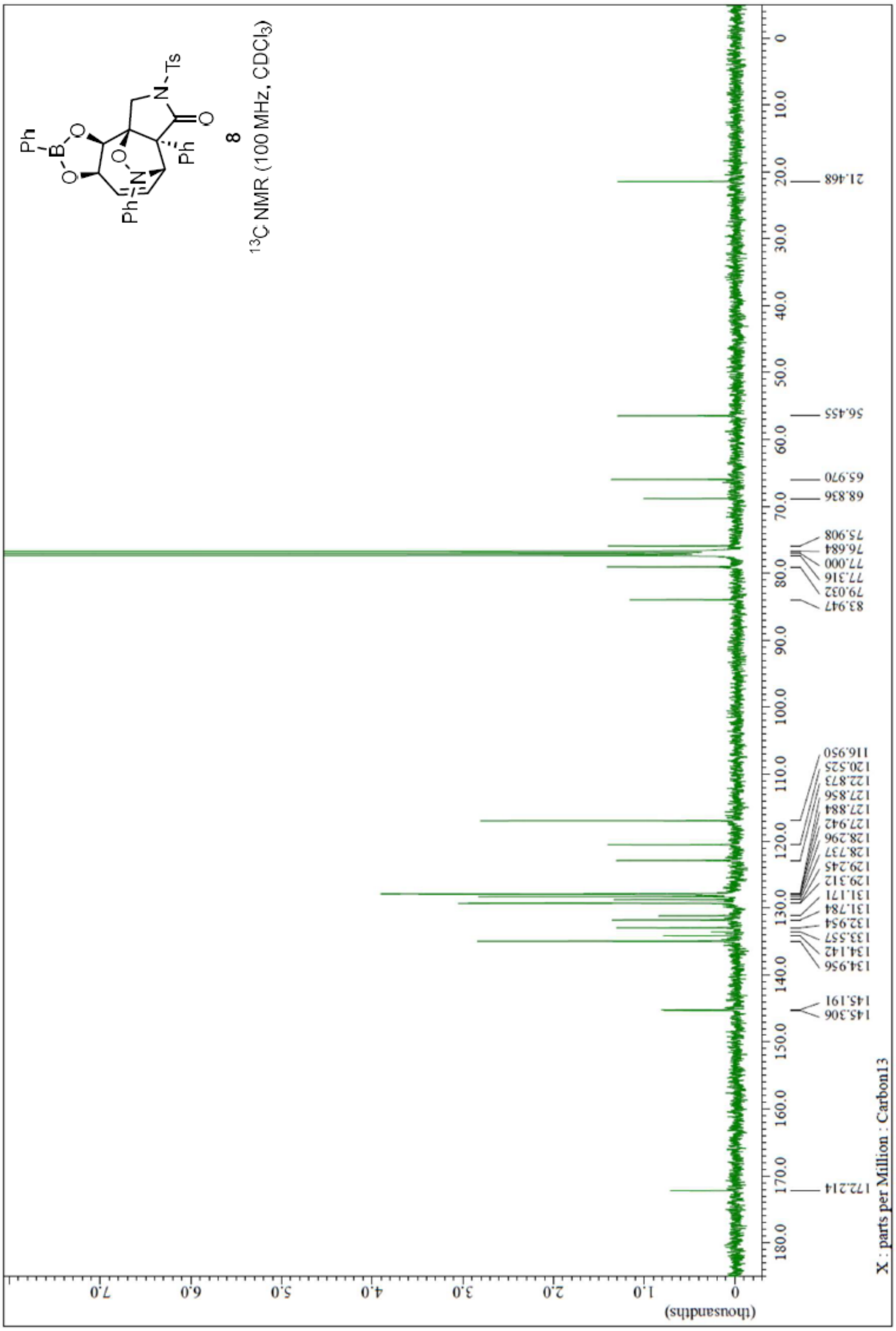
8
13C NMR (100 MHz, CDCl3)
172.214
145.306
145.191
134.956
134.142
133.557
132.954
131.784
131.171
129.312
129.245
128.737
128.296
127.942
127.884
127.856
122.873
120.525
116.950
83.947
79.032
77.316
77.000
76.684
75.908
68.836
65.970
56.455
21.468
X : parts per Million : Carbon13
(thousandths)

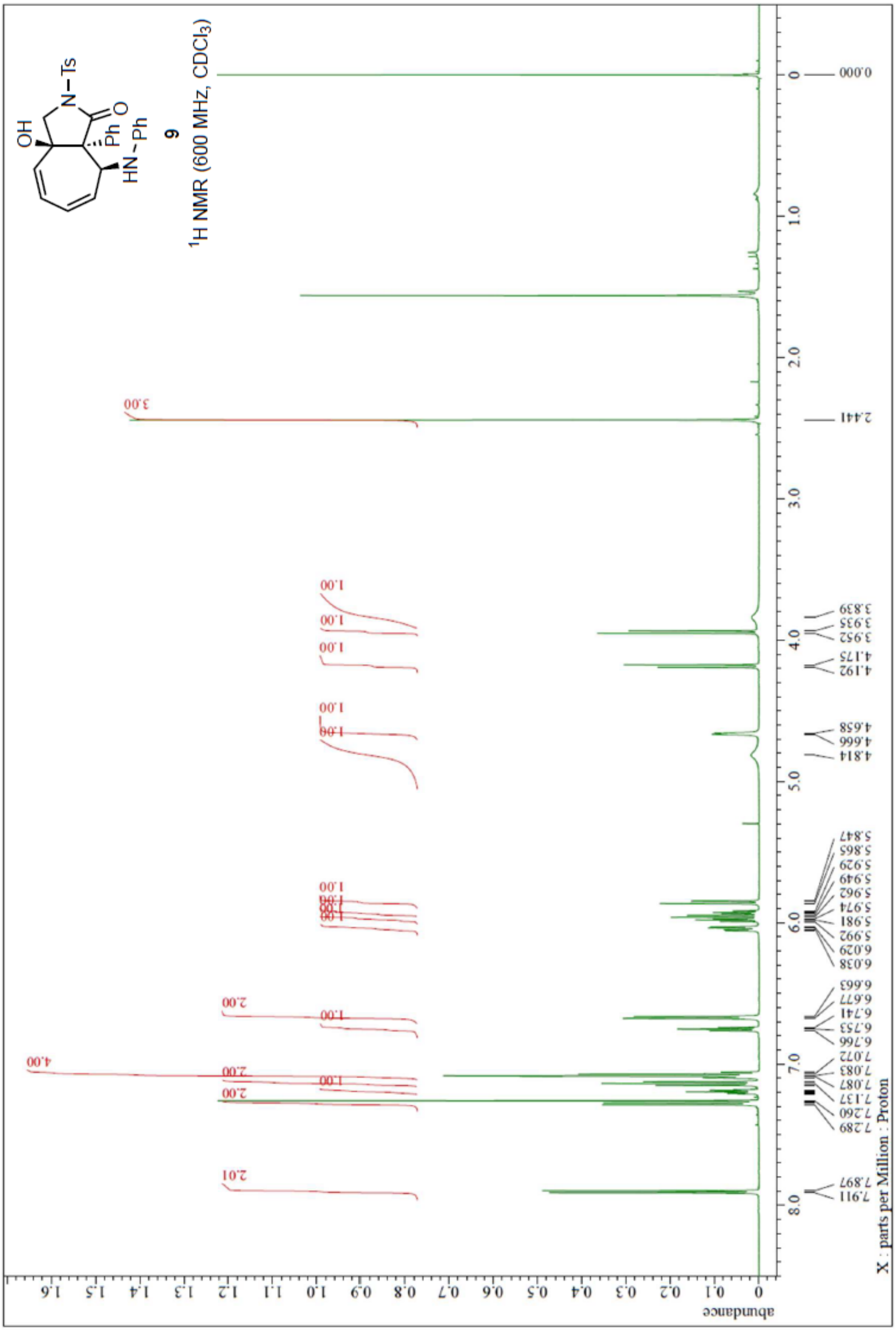

OH
N−Ts
O
Ph
HN
Ph
9
1H NMR (600 MHz, CDCl3)
X : parts per Million : Proton
abundance

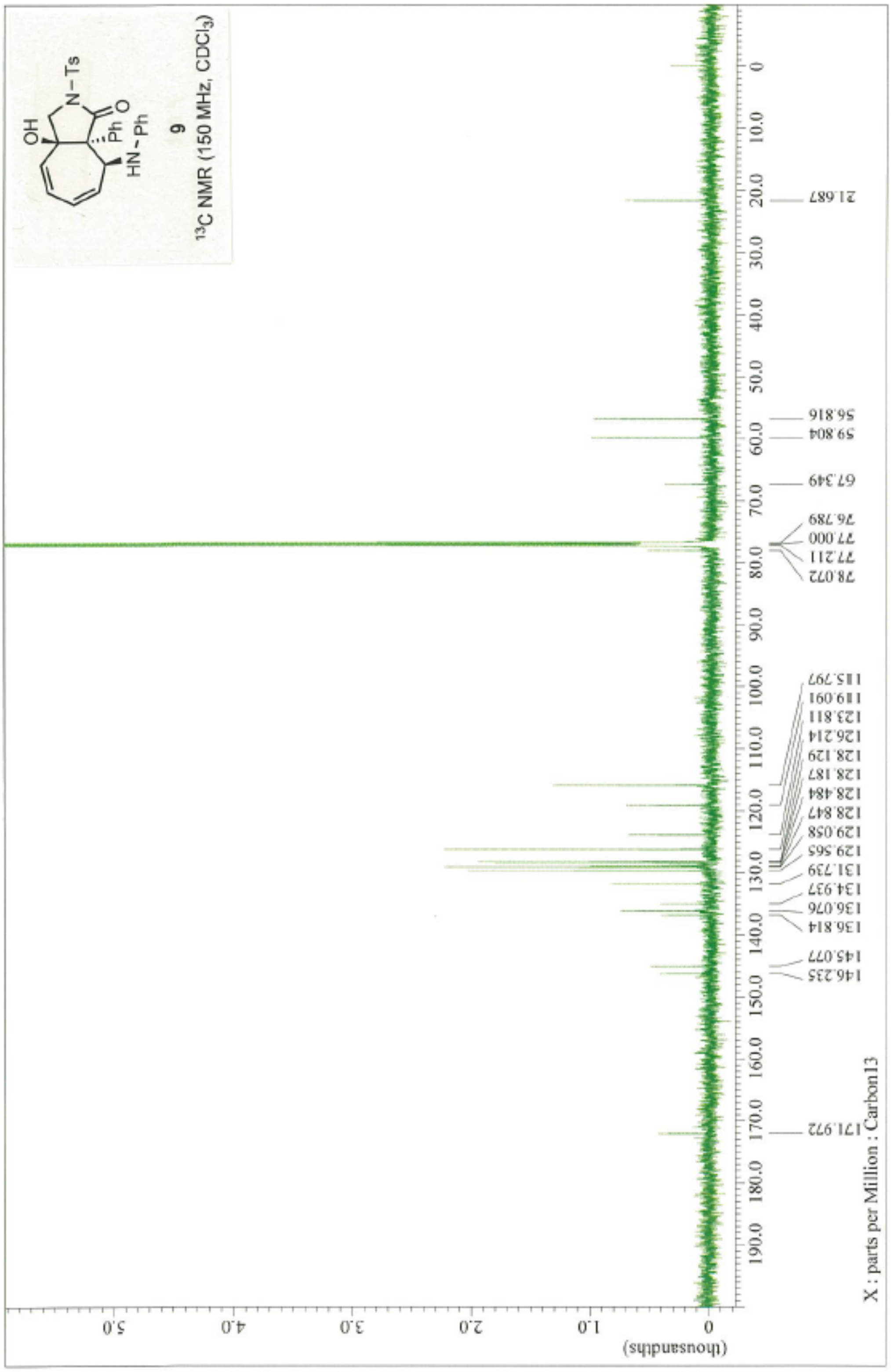
OH
N−Ts
O
Ph
HN−Ph
9
13C NMR (150 MHz, CDCl3)
21.687
56.816
59.804
67.349
76.789
77.000
77.211
78.072
115.797
119.091
123.811
126.214
128.129
128.187
128.484
128.847
129.058
129.565
131.739
134.937
136.076
136.814
145.077
146.235
171.972
X : parts per Million : Carbon13
(thousandths)

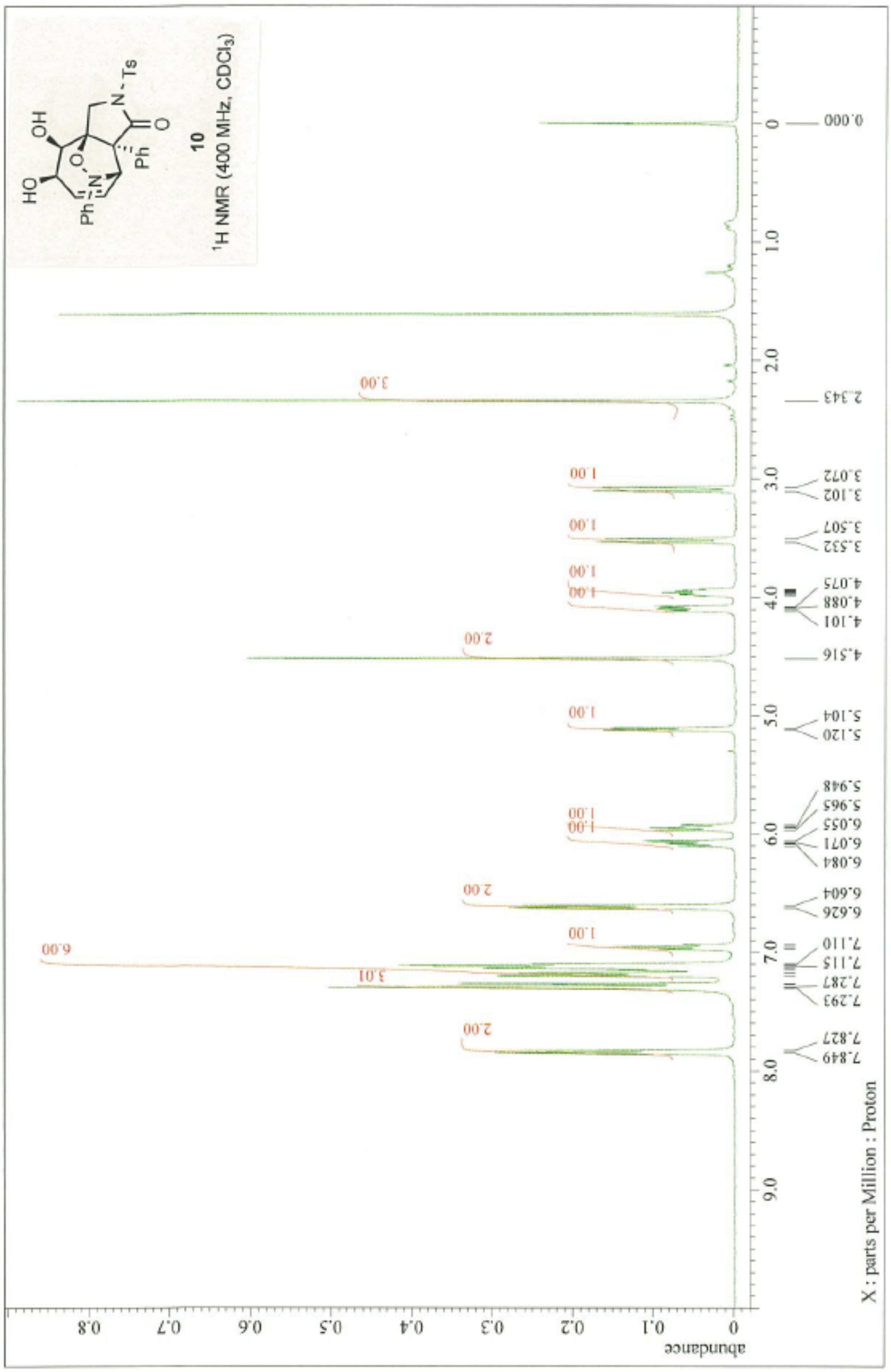

10
1H NMR (400 MHz, CDCl3)
X : parts per Million : Proton
abundance

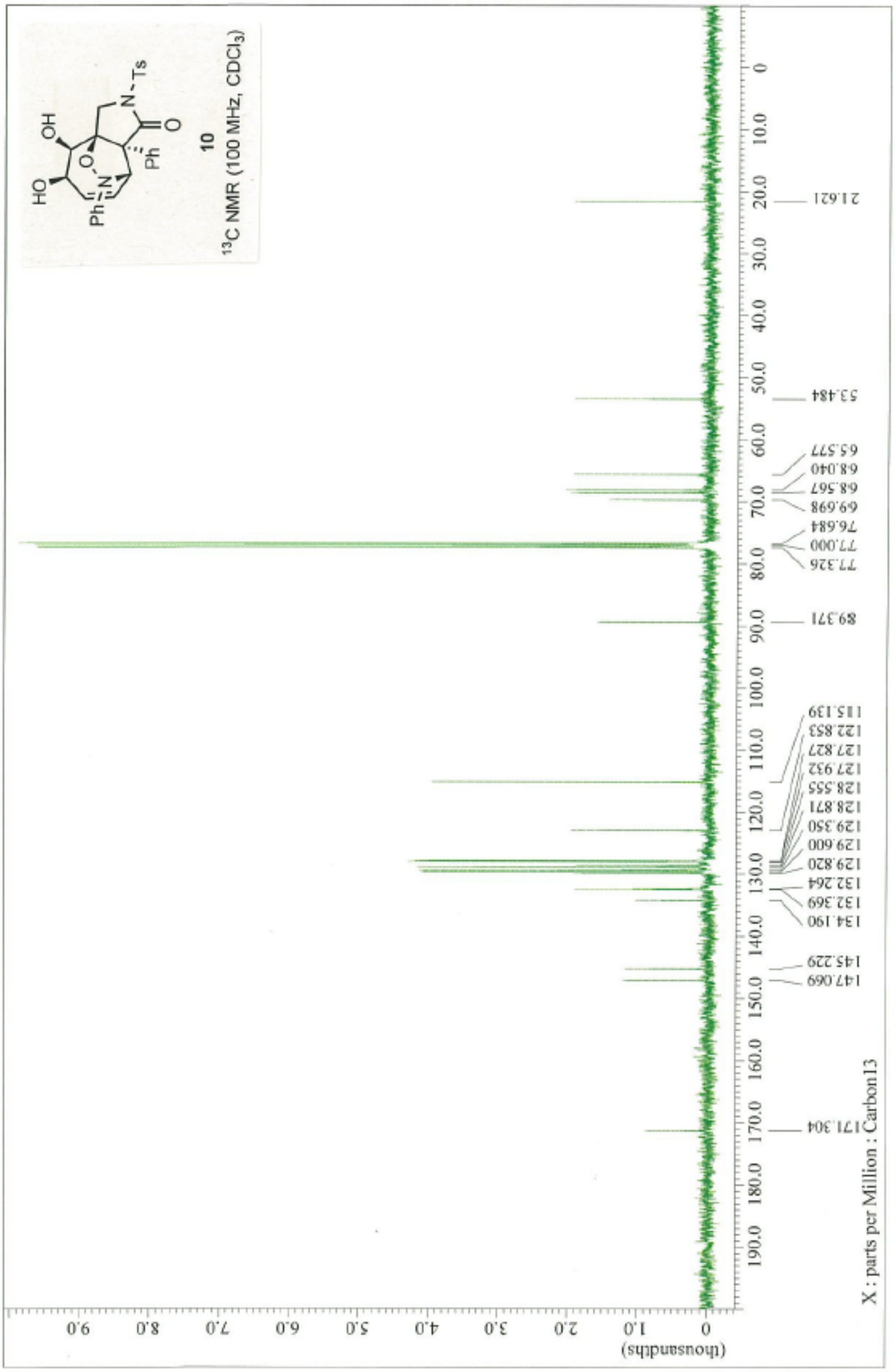

HO
OH
Ph
O
N
N
Ts
O
Ph
10
13C NMR (100 MHz, CDCl3)
21.621
53.484
65.577
68.040
68.567
69.698
76.684
77.000
77.326
89.371
115.139
122.853
127.827
127.932
128.555
128.871
129.350
129.600
129.820
132.264
132.369
134.190
145.229
147.069
171.304
X : parts per Million : Carbon13
(thousandths)

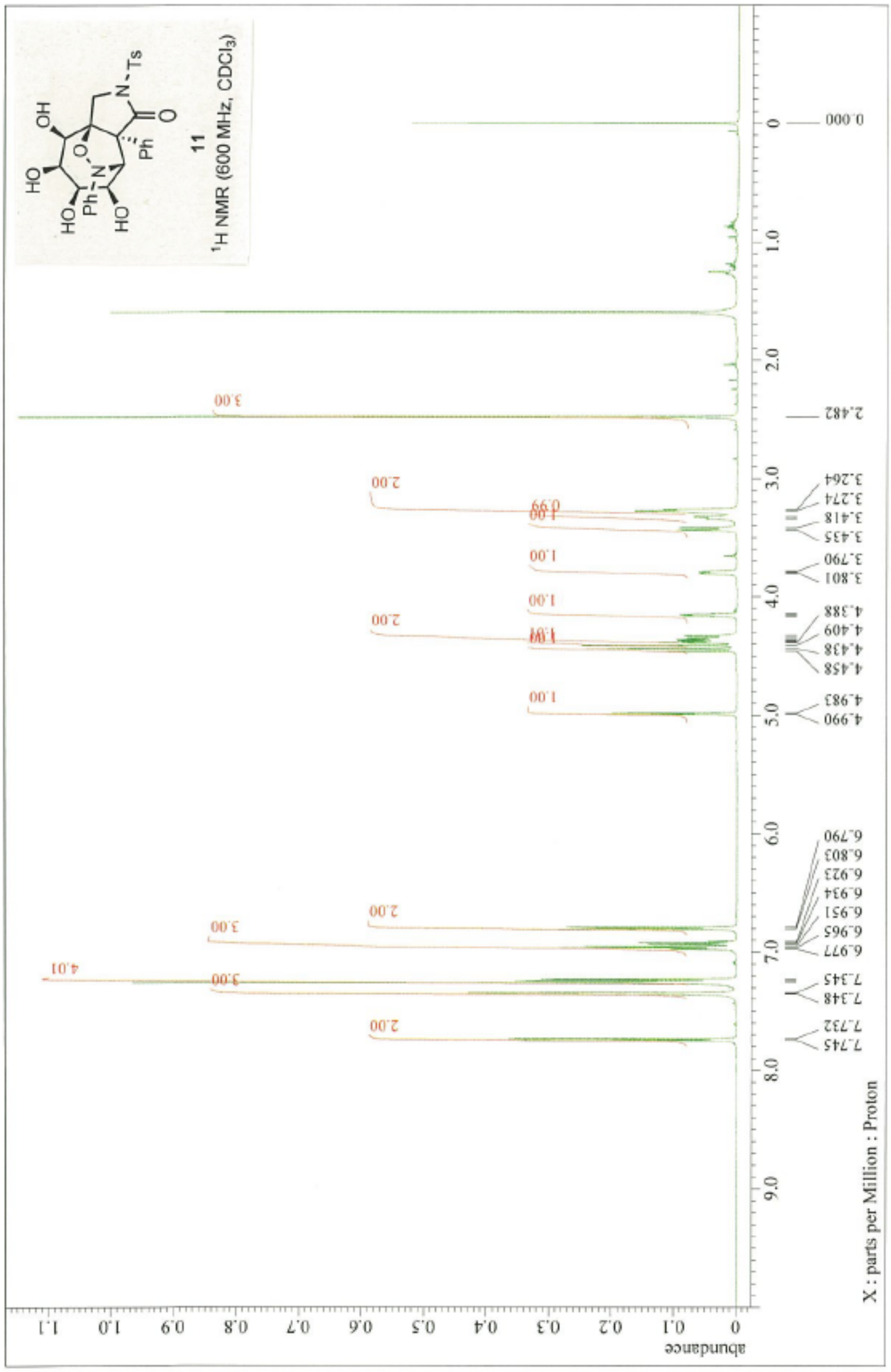
11
¹H NMR (600 MHz, CDCl₃)
X : parts per Million : Proton
abundance

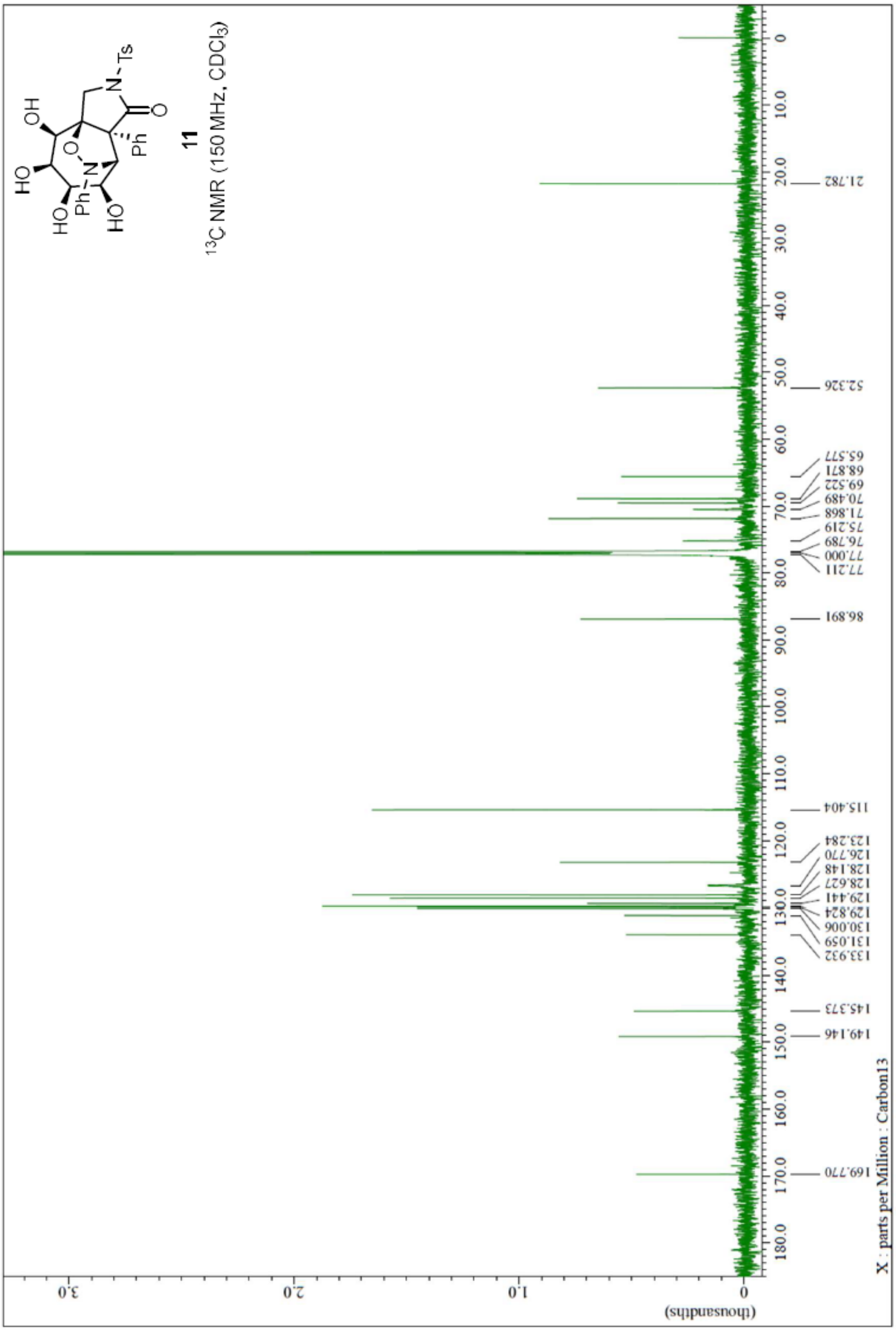
11
^{13}C NMR (150 MHz, CDCl$_3$)
HO
OH
Ph
N
O
Ts
21.782
52.326
65.577
68.871
69.522
70.489
71.868
75.219
76.789
77.000
77.211
86.891
115.404
123.284
126.770
128.148
128.627
129.441
129.824
130.006
131.059
133.932
145.373
149.146
169.770
X : parts per Million : Carbon13
(thousandths)

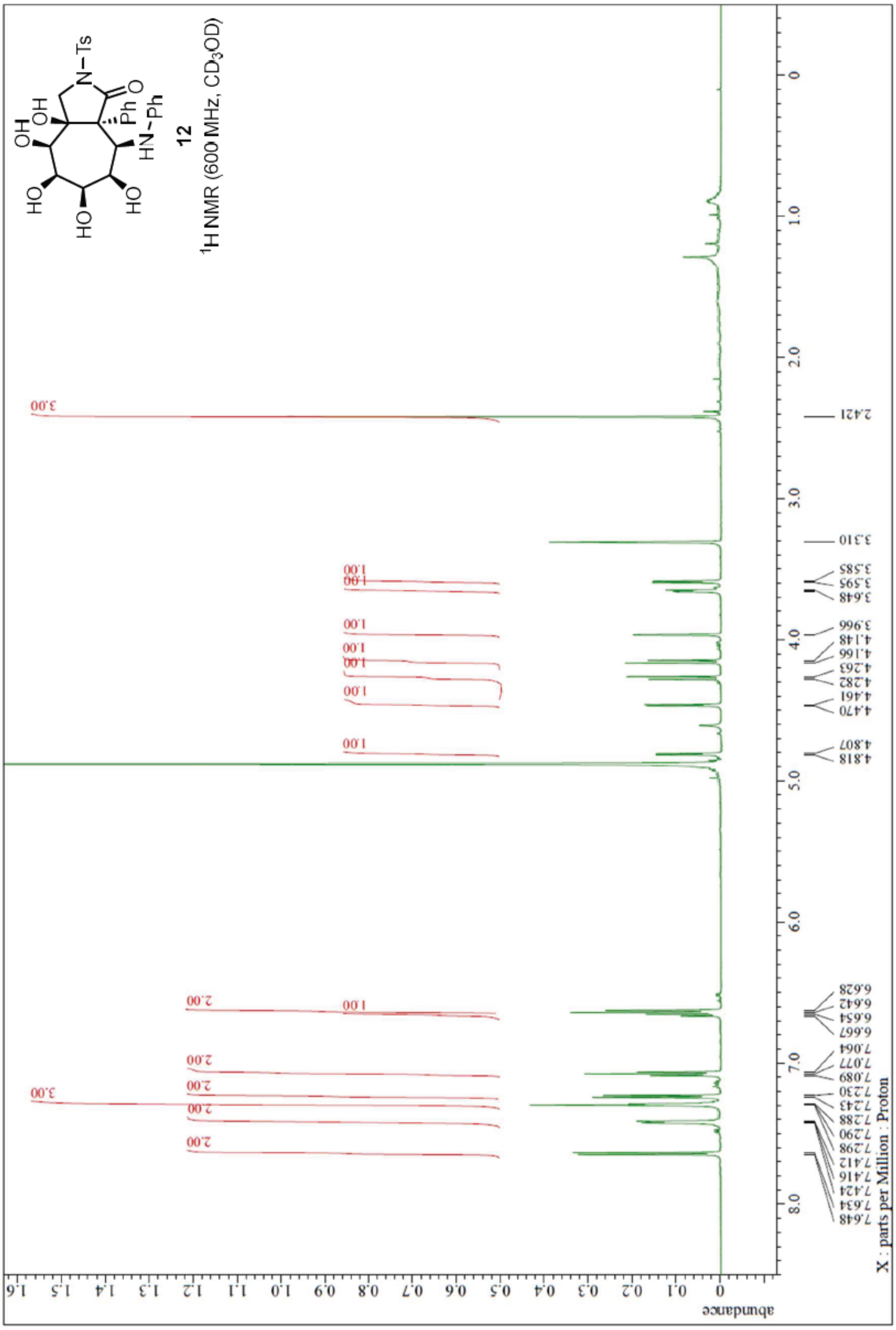

12
^{1}H NMR (600 MHz, CD_3OD)
X : parts per Million : Proton
abundance
2.421
3.310
3.585
3.595
3.648
3.966
4.148
4.166
4.263
4.282
4.461
4.470
4.807
4.818
6.628
6.642
6.654
6.667
7.064
7.077
7.089
7.230
7.243
7.288
7.290
7.298
7.412
7.416
7.424
7.634
7.648

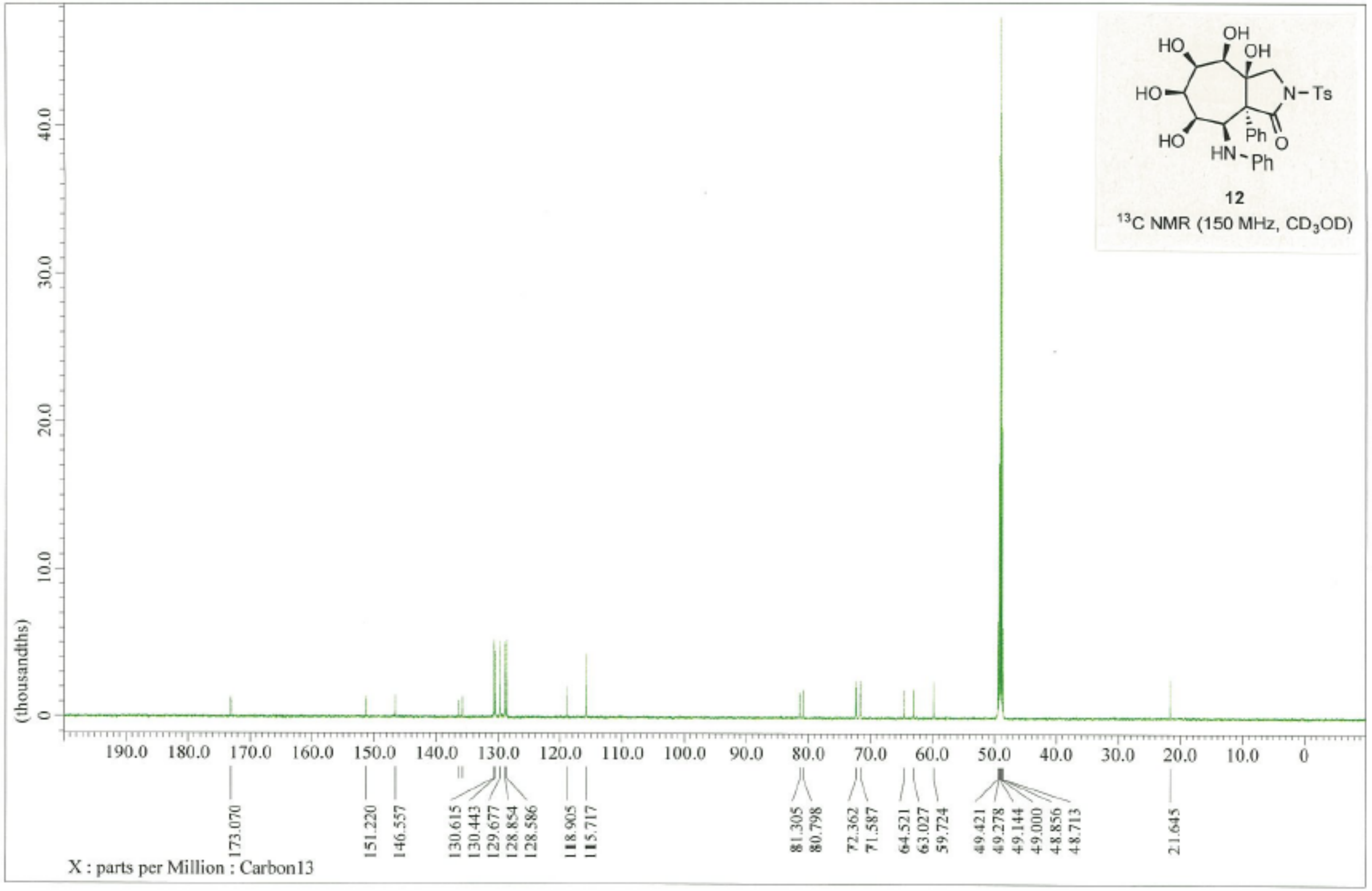
HO
OH
OH
HO
N−Ts
HO
Ph
O
HN−Ph
12
^{13}C NMR (150 MHz, CD_3OD)
(thousandths)
40.0
30.0
20.0
10.0
0
190.0 180.0 170.0 160.0 150.0 140.0 130.0 120.0 110.0 100.0 90.0 80.0 70.0 60.0 50.0 40.0 30.0 20.0 10.0 0
173.070
151.220
146.557
130.615
130.443
129.677
128.854
128.586
118.905
115.717
81.305
80.798
72.362
71.587
64.521
63.027
59.724
49.421
49.278
49.144
49.000
48.856
48.713
21.645
X : parts per Million : Carbon13

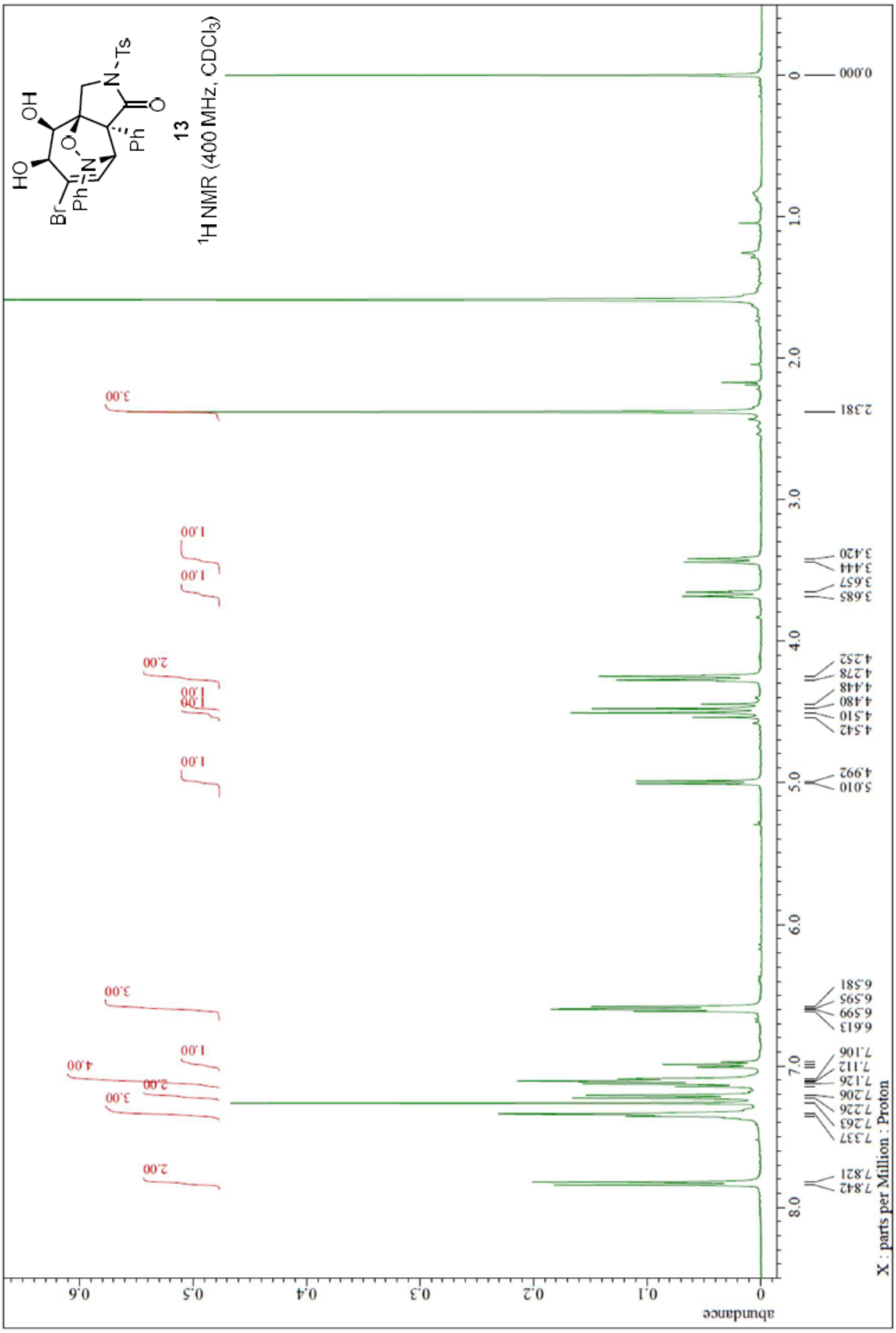
13
1H NMR (400 MHz, CDCl3)
X : parts per Million : Proton
abundance
0.000
2.381
3.420
3.444
3.657
3.685
4.252
4.278
4.448
4.480
4.510
4.542
4.992
5.010
6.581
6.595
6.599
6.613
7.106
7.112
7.126
7.206
7.226
7.263
7.337
7.821
7.842

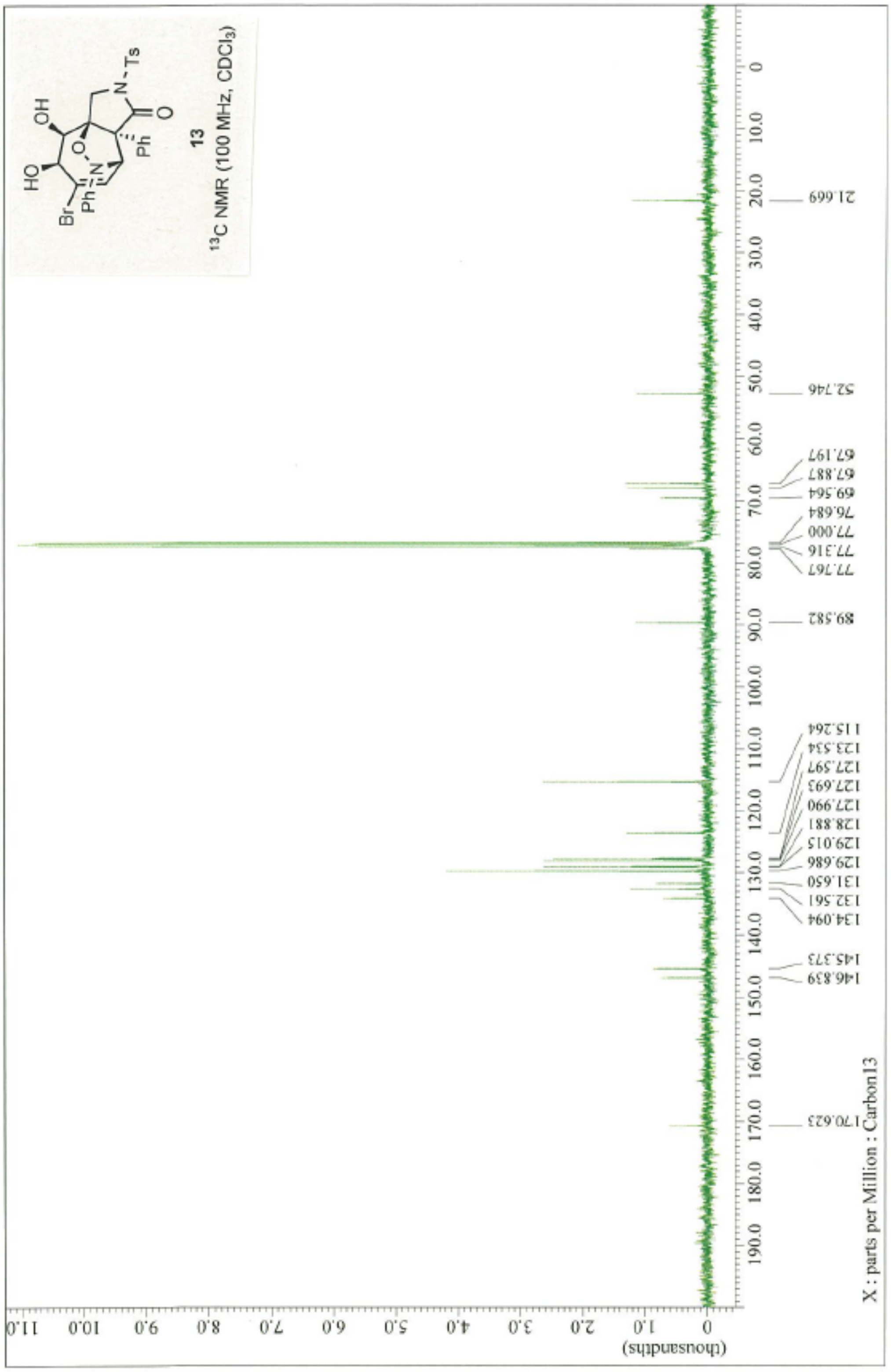

13
^{13}C NMR (100 MHz, CDCl$_3$)
21.669
52.746
67.197
67.887
69.564
76.684
77.000
77.316
77.767
89.582
115.264
123.534
127.597
127.693
127.990
128.881
129.015
129.686
131.650
132.561
134.094
145.373
146.839
170.623
X : parts per Million : Carbon13
(thousandths)

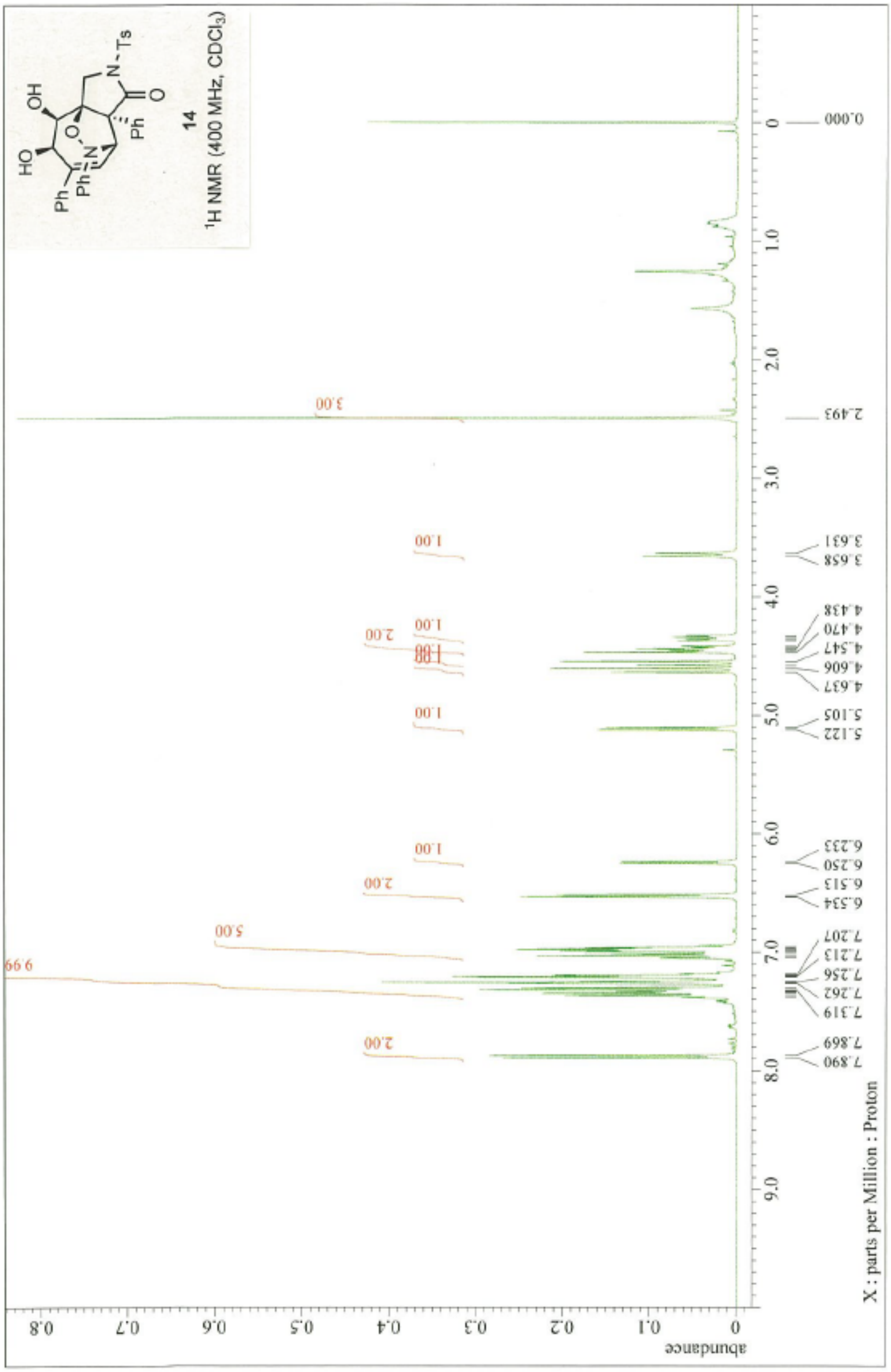
14
1H NMR (400 MHz, CDCl3)
X : parts per Million : Proton
abundance
0.000
2.493
3.631
3.658
4.438
4.470
4.547
4.606
4.637
5.105
5.122
6.233
6.250
6.513
6.534
7.207
7.213
7.256
7.262
7.319
7.869
7.890
3.00
1.00
2.00
1.00
1.88
1.00
1.00
2.00
5.00
9.99
2.00

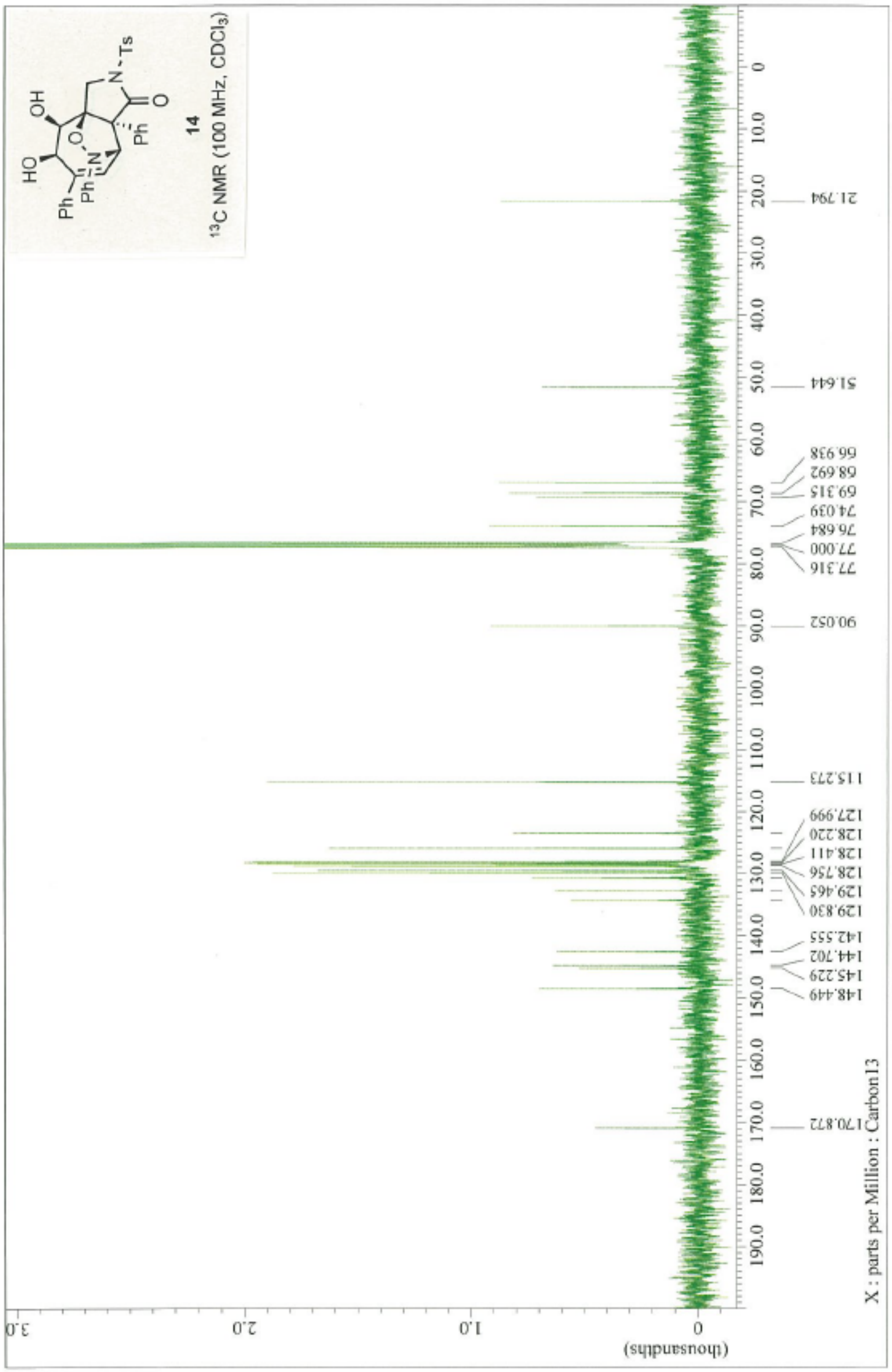

14
13C NMR (100 MHz, CDCl3)
21.794
51.644
66.938
68.692
69.315
74.039
76.684
77.000
77.316
90.052
115.273
127.999
128.220
128.411
128.756
129.465
129.830
142.555
144.702
145.229
148.449
170.872
X : parts per Million : Carbon13
(thousandths)

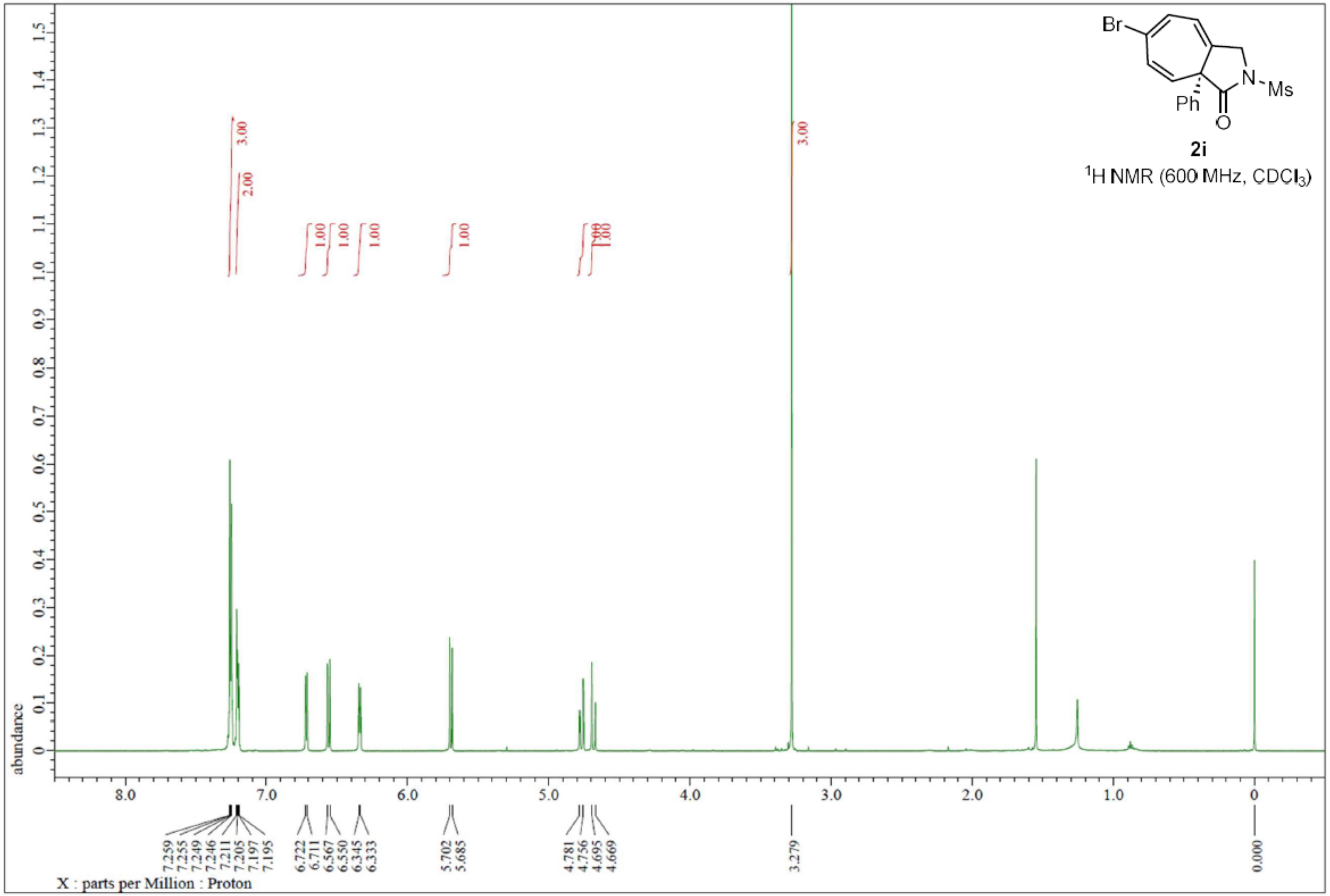
Br
N
Ms
Ph
O
2i
^{1}H NMR (600 MHz, $CDCl_3$)
3.00
2.00
1.00
1.00
1.00
1.00
1.00
1.00
3.00
abundance
X : parts per Million : Proton
7.259
7.255
7.249
7.246
7.211
7.205
7.197
7.195
6.722
6.711
6.567
6.550
6.345
6.333
5.702
5.685
4.781
4.756
4.695
4.669
3.279
0.000

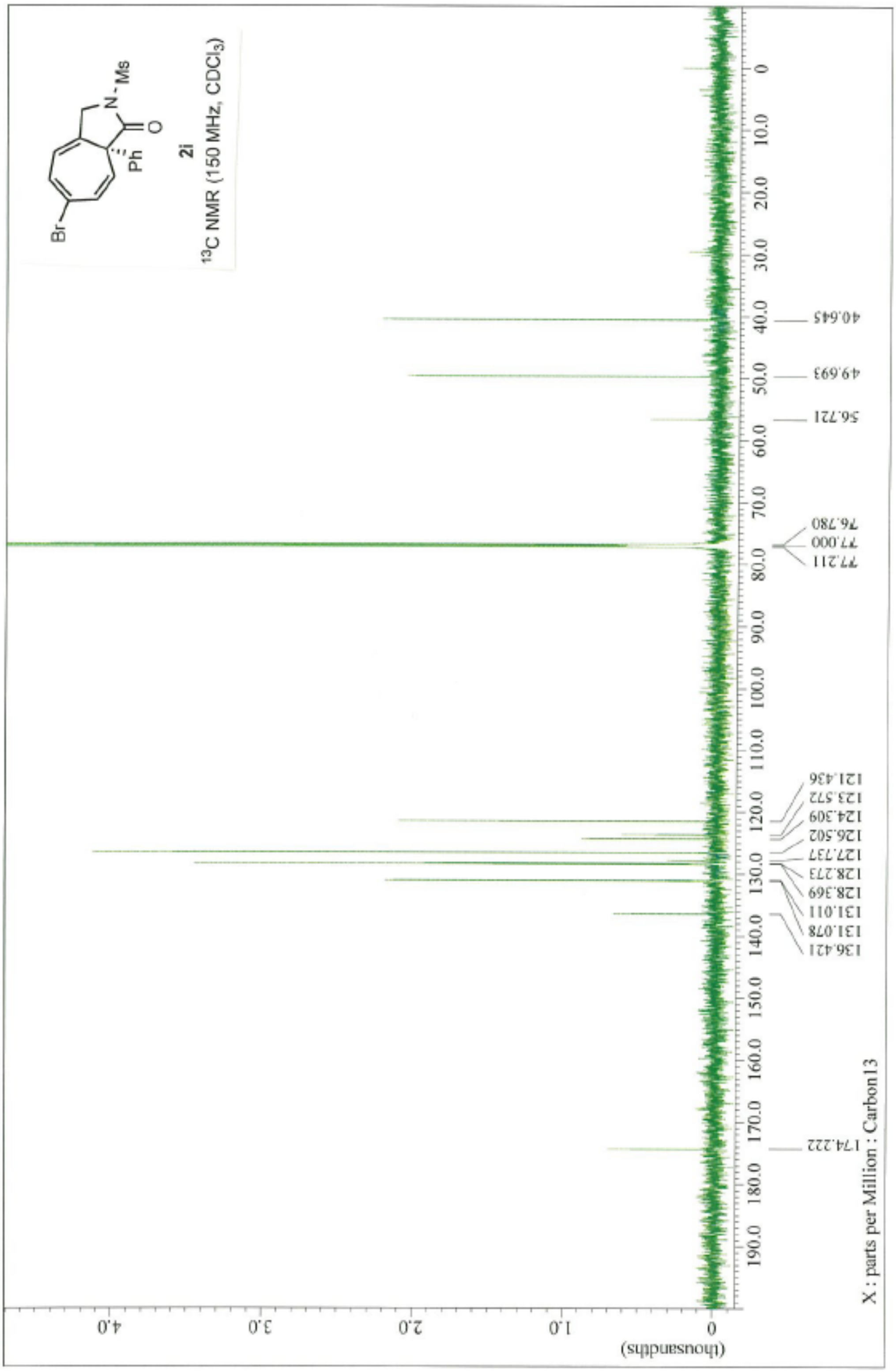
2i
13C NMR (150 MHz, CDCl3)
40.645
49.693
56.721
76.780
77.000
77.211
121.436
123.572
124.309
126.502
127.737
128.273
128.369
131.011
131.078
136.421
174.222
X : parts per Million : Carbon13
(thousandths)

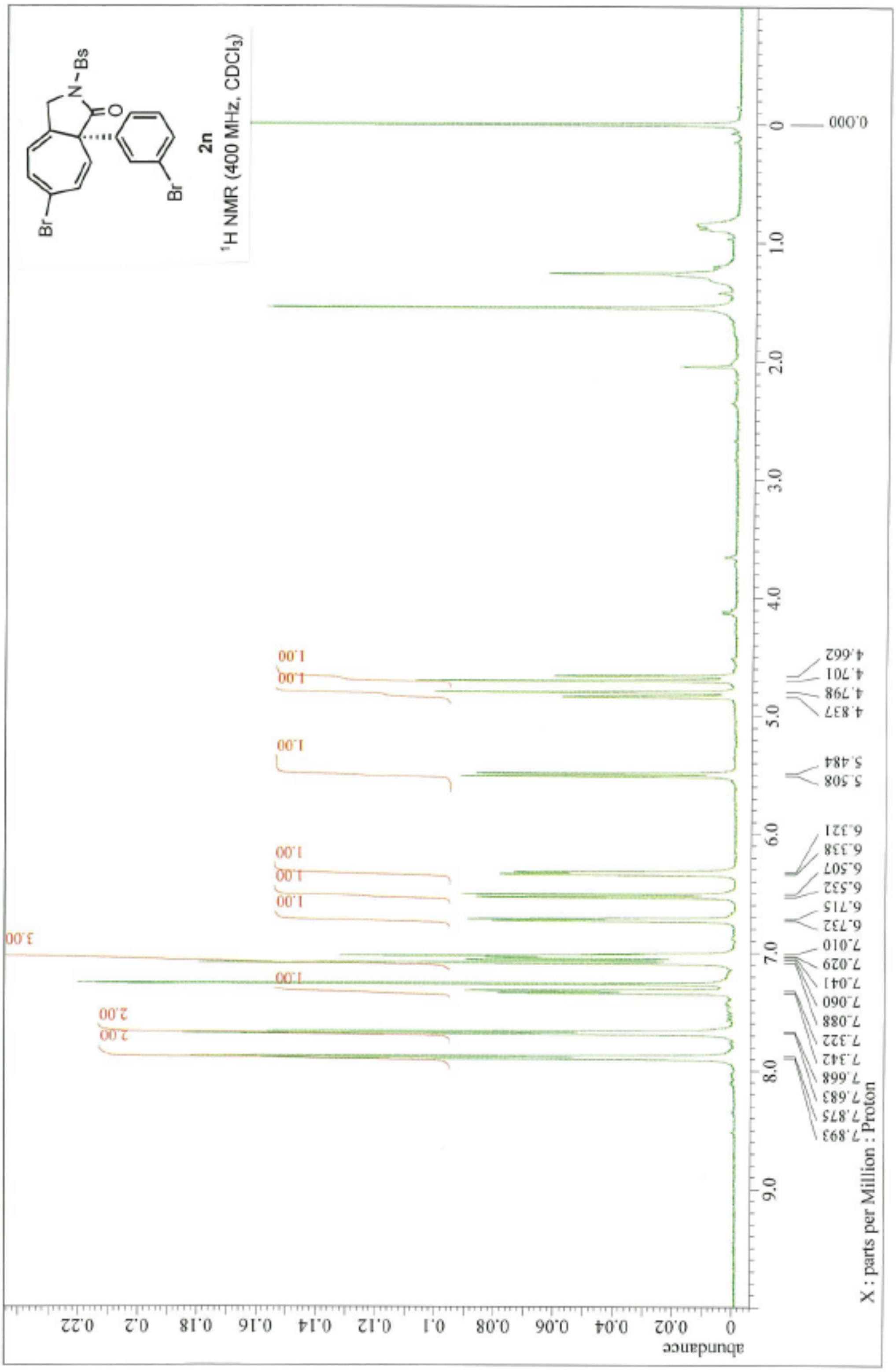

N–Bs
O
Br
Br
2n
¹H NMR (400 MHz, CDCl₃)
0.000
4.662
4.701
4.798
4.837
5.484
5.508
6.321
6.338
6.507
6.532
6.715
6.732
7.010
7.029
7.041
7.060
7.088
7.322
7.342
7.668
7.683
7.875
7.893
1.00
1.00
1.00
1.00
1.00
1.00
3.00
1.00
2.00
2.00
X : parts per Million : Proton
abundance

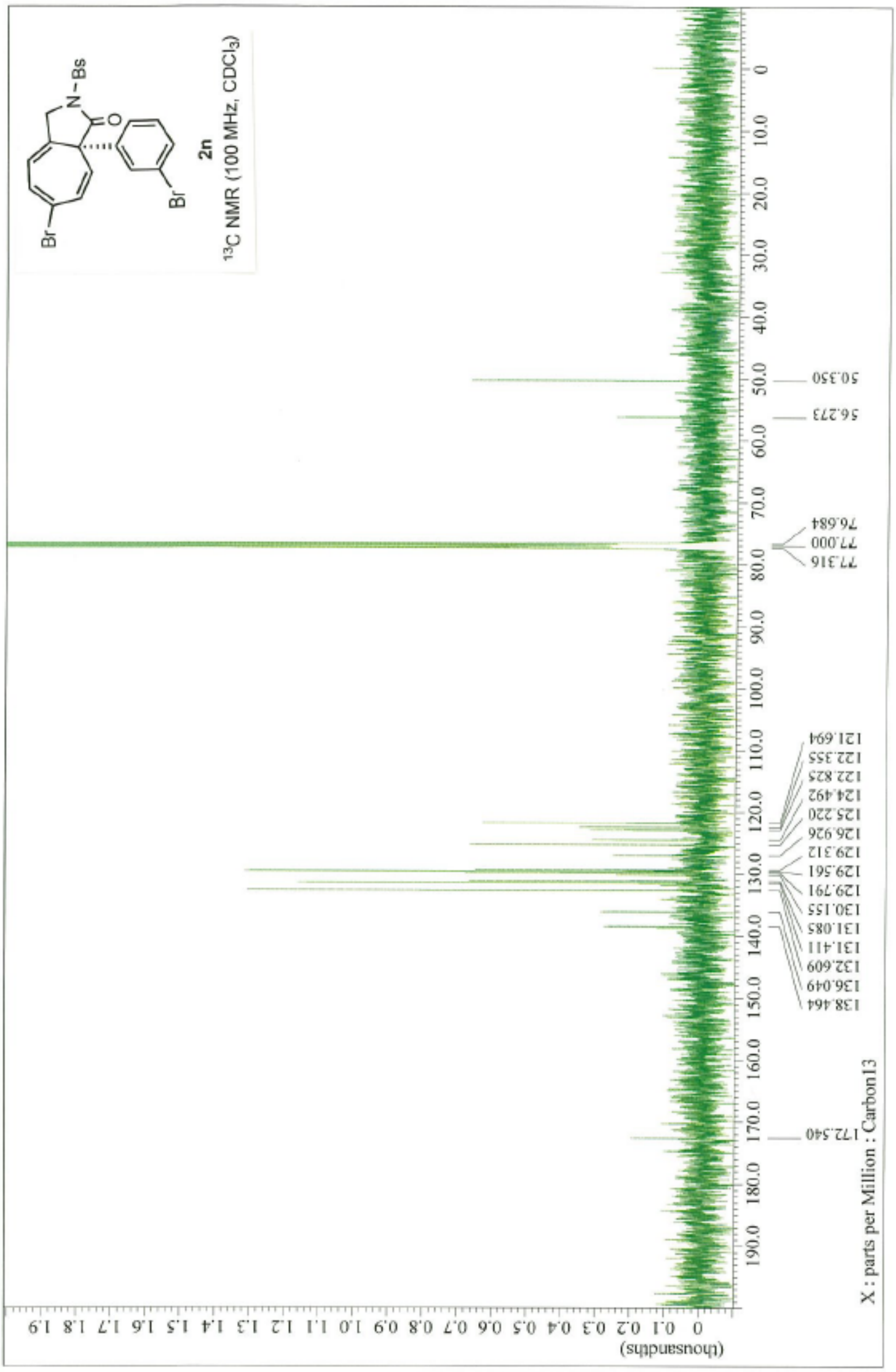
Br
N−Bs
O
Br
2n
13C NMR (100 MHz, CDCl3)
50.350
56.273
76.684
77.000
77.316
121.694
122.355
122.825
124.492
125.220
126.926
129.312
129.561
129.791
130.155
131.085
131.411
132.609
136.049
138.464
172.540
X : parts per Million : Carbon13
(thousandths)

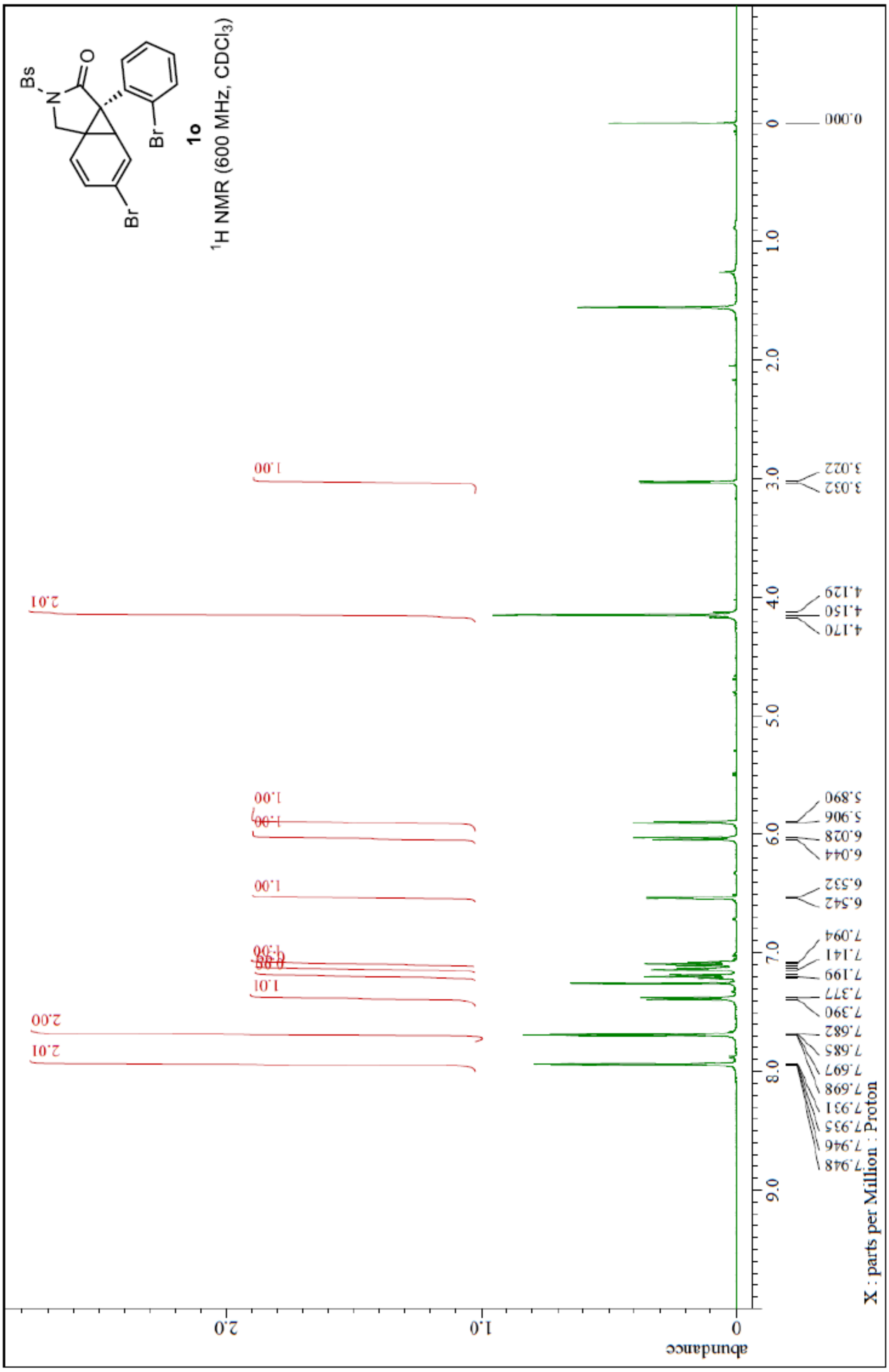

1o
1H NMR (600 MHz, CDCl3)
X : parts per Million : Proton
abundance

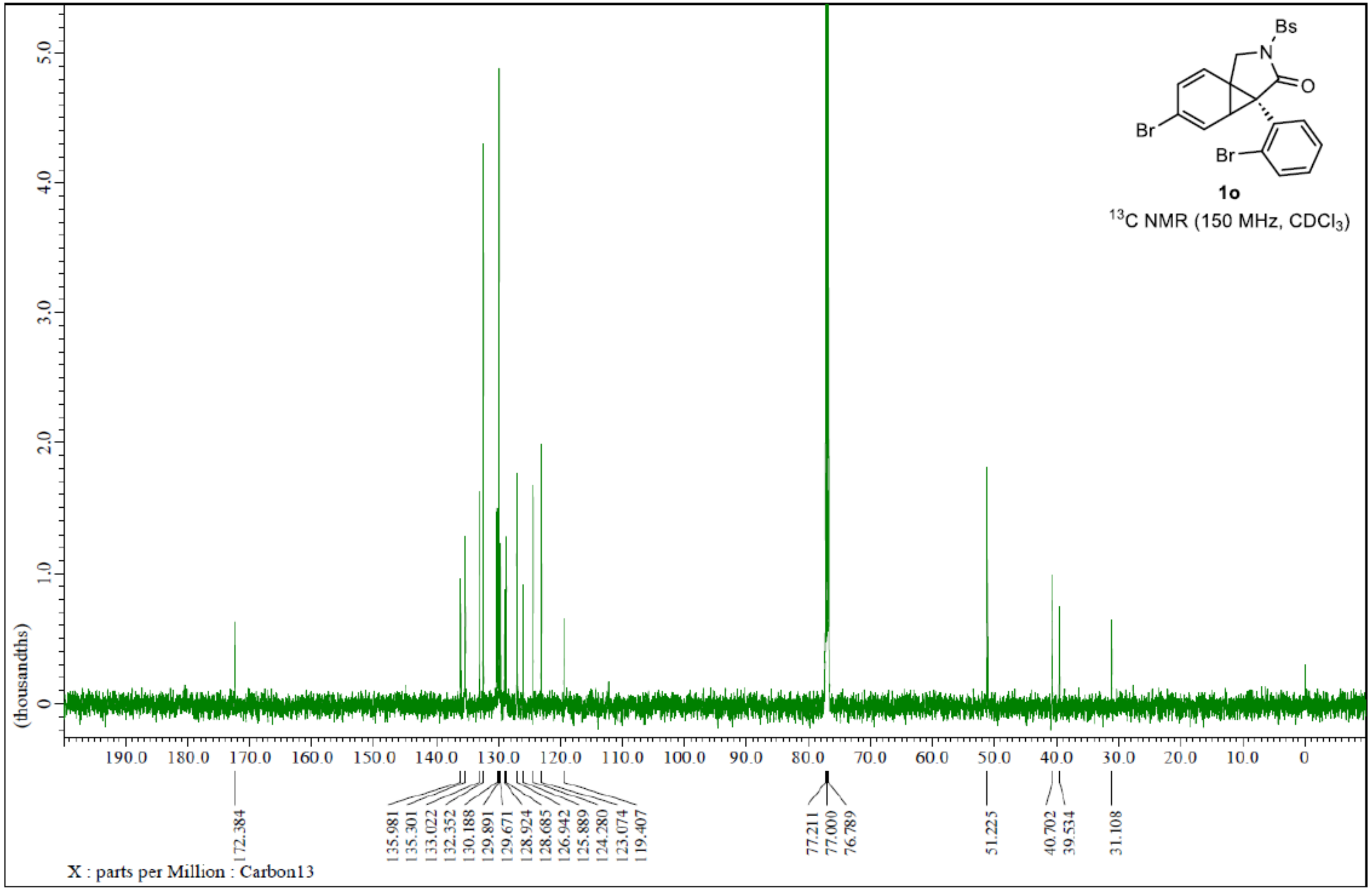

Bs
N
O
Br
Br
1o
^{13}C NMR (150 MHz, CDCl$_3$)
(thousandths)
5.0
4.0
3.0
2.0
1.0
0
190.0
180.0
170.0
160.0
150.0
140.0
130.0
120.0
110.0
100.0
90.0
80.0
70.0
60.0
50.0
40.0
30.0
20.0
10.0
0
172.384
135.981
135.301
133.022
132.352
130.188
129.891
129.671
128.924
128.685
126.942
125.889
124.280
123.074
119.407
77.211
77.000
76.789
51.225
40.702
39.534
31.108
X : parts per Million : Carbon13

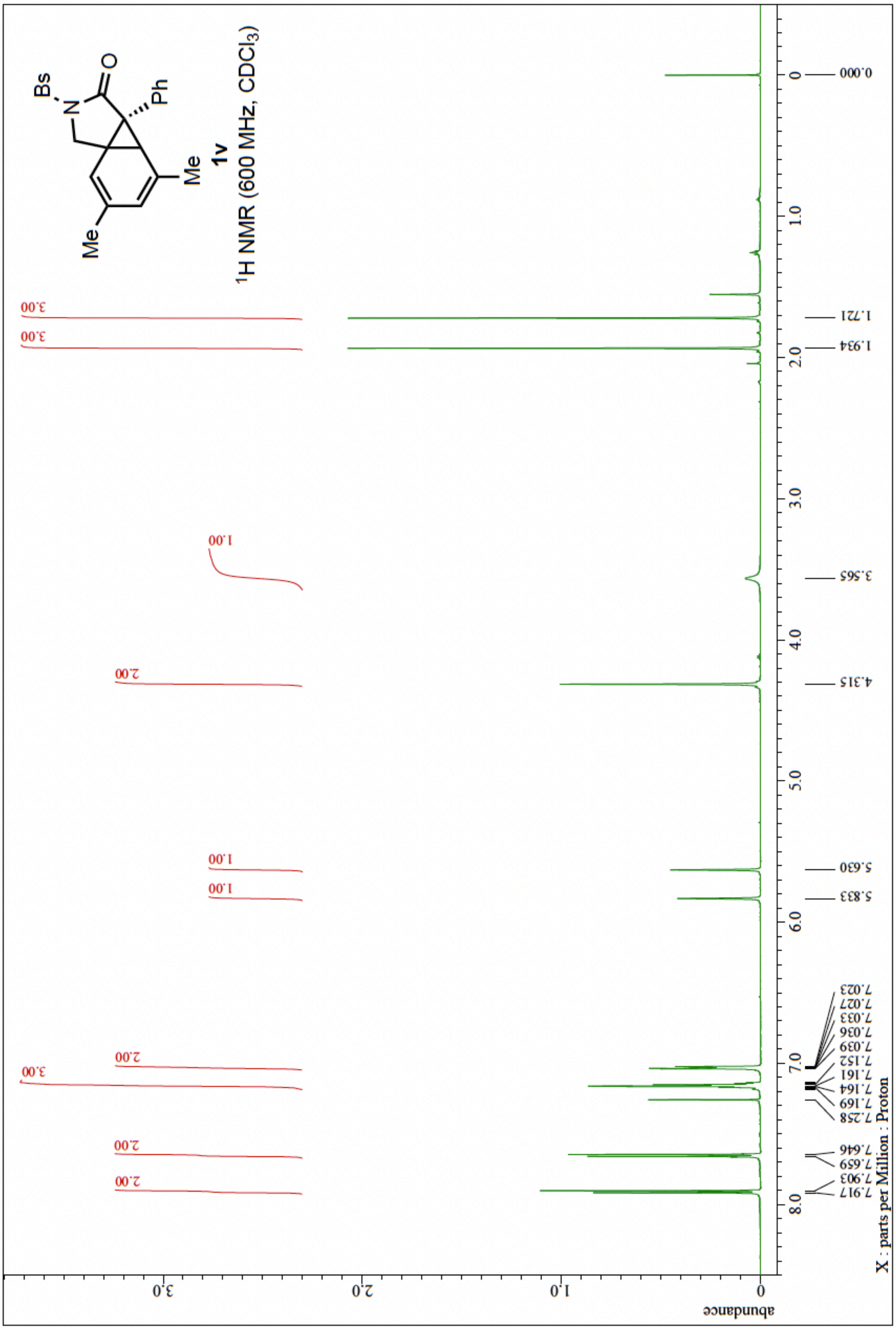

Bs
N
O
Ph
Me
Me
1v
¹H NMR (600 MHz, CDCl₃)
0.000
1.721
1.934
3.565
4.315
5.630
5.833
7.023
7.027
7.033
7.036
7.039
7.152
7.161
7.164
7.169
7.258
7.646
7.659
7.903
7.917
3.00
3.00
1.00
2.00
1.00
1.00
2.00
3.00
2.00
2.00
0
1.0
2.0
3.0
4.0
5.0
6.0
7.0
8.0
X : parts per Million : Proton
abundance

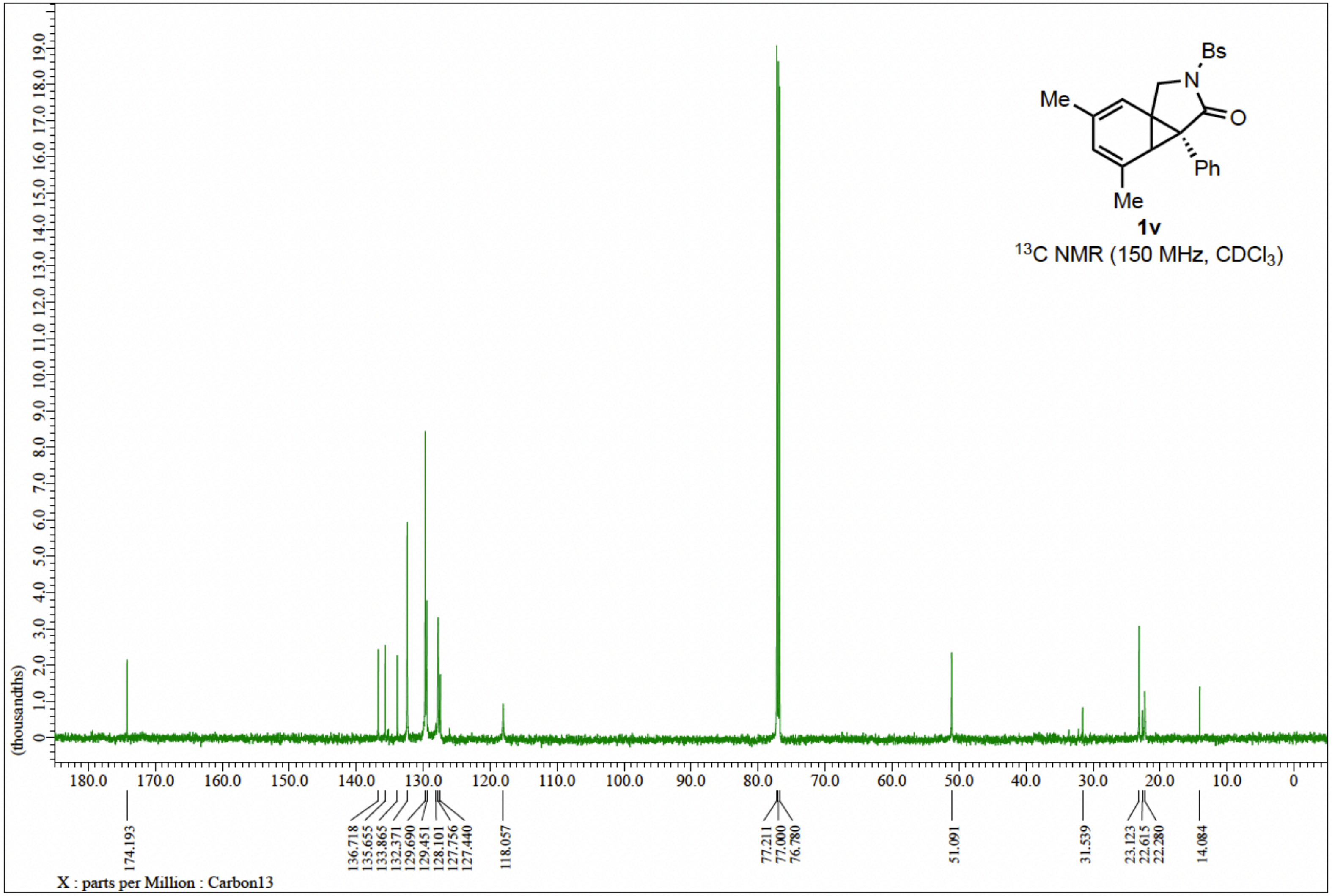

Bs
N
O
Me
Ph
Me
1v
13C NMR (150 MHz, CDCl3)
(thousandths)
174.193
136.718
135.655
133.865
132.371
129.690
129.451
128.101
127.756
127.440
118.057
77.211
77.000
76.780
51.091
31.539
23.123
22.615
22.280
14.084
X : parts per Million : Carbon13

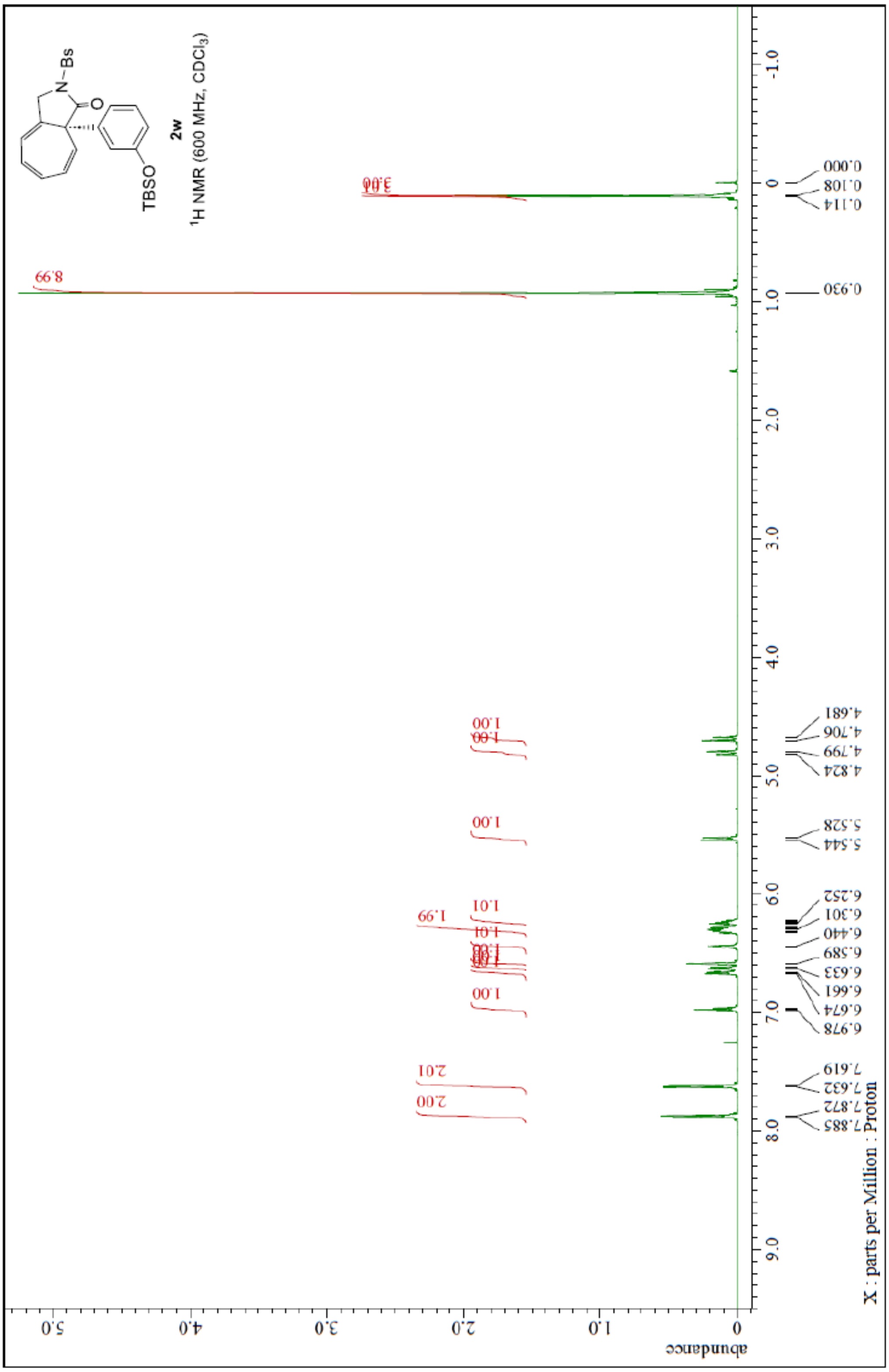

TBSO
N–Bs
2w
1H NMR (600 MHz, CDCl3)
0.000
0.108
0.114
0.930
4.681
4.706
4.799
4.824
5.528
5.544
6.252
6.301
6.440
6.589
6.633
6.661
6.674
6.978
7.619
7.632
7.872
7.885
X : parts per Million : Proton
abundance

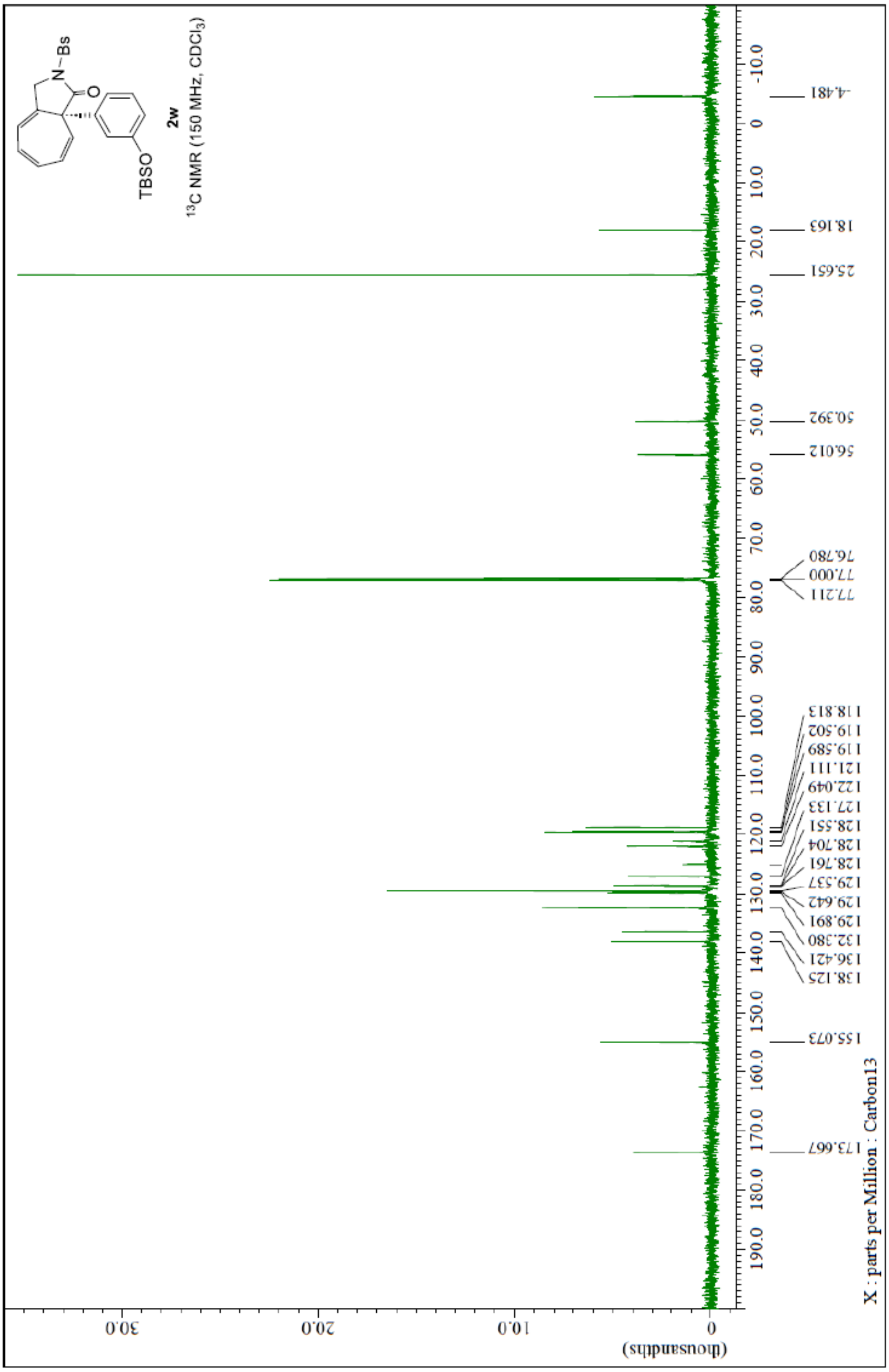
N~Bs
O
TBSO
2w
13C NMR (150 MHz, CDCl3)
-4.481
18.163
25.651
50.392
56.012
76.780
77.000
77.211
118.813
119.502
119.589
121.111
122.049
127.133
128.551
128.704
128.761
129.537
129.642
129.891
132.380
136.421
138.125
155.073
173.667
X : parts per Million : Carbon13
(thousandths)

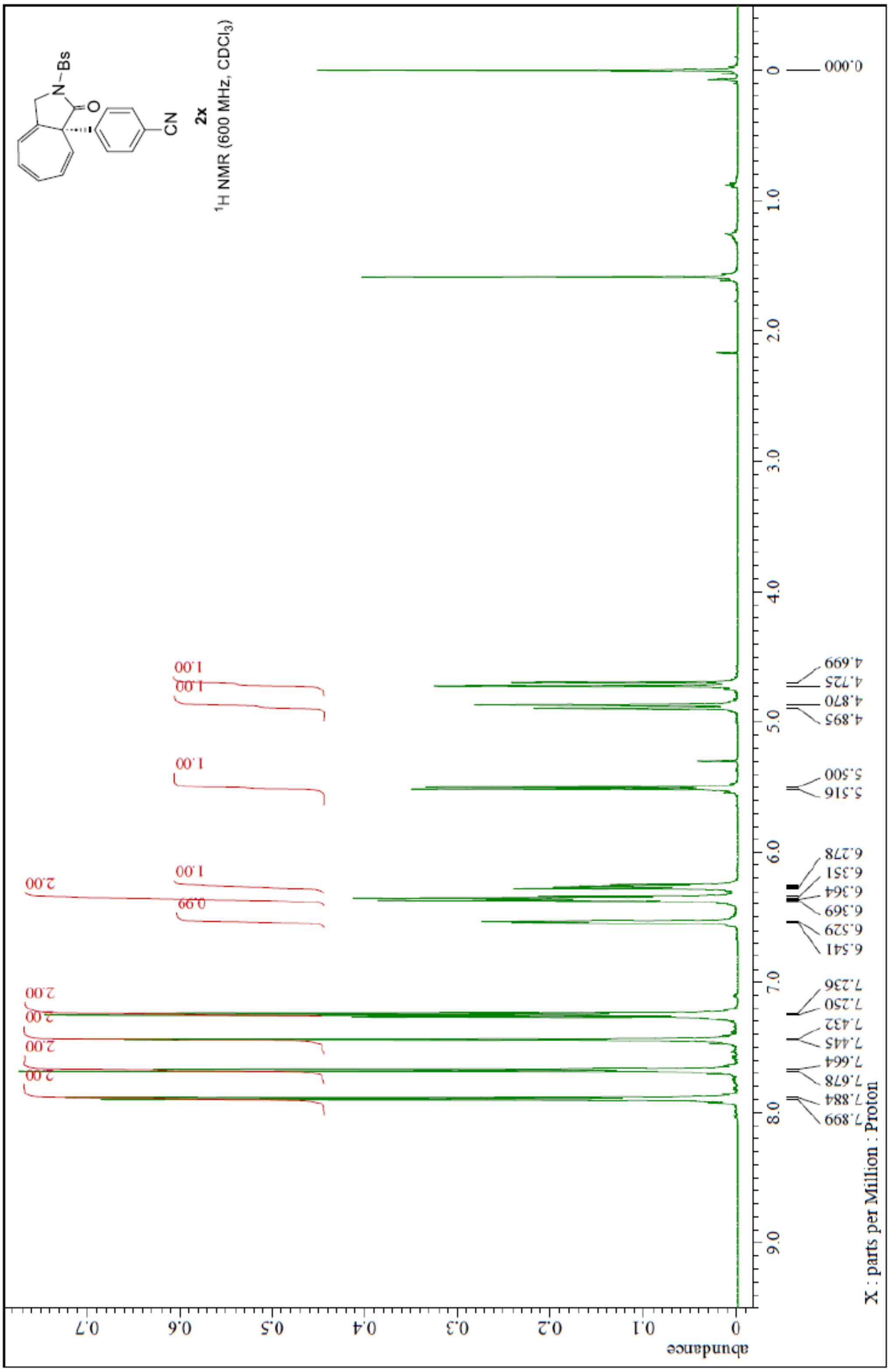
N–Bs
O
CN
2x
^{1}H NMR (600 MHz, CDCl$_3$)
0.000
4.699
4.725
4.870
4.895
5.500
5.516
6.278
6.351
6.364
6.369
6.529
6.541
7.236
7.250
7.432
7.445
7.664
7.678
7.884
7.899
1.00
1.00
1.00
1.00
0.99
2.00
2.00
2.00
2.00
2.00
X : parts per Million : Proton
abundance

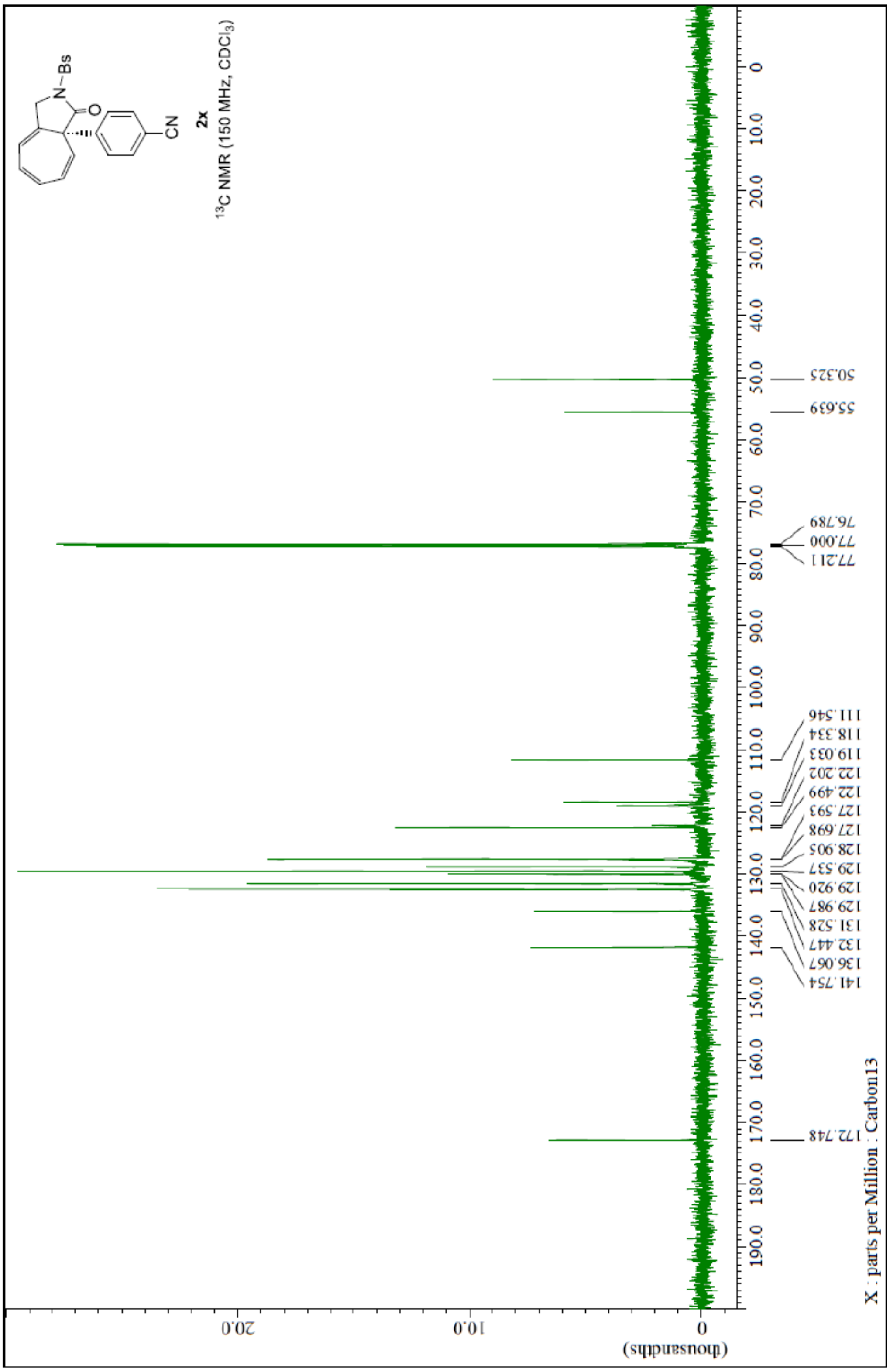
N–Bs
O
CN
2x
13C NMR (150 MHz, CDCl3)
50.325
55.639
76.789
77.000
77.211
111.546
118.334
119.033
122.202
122.499
127.593
127.698
128.905
129.537
129.920
129.987
131.528
132.447
136.067
141.754
172.748
X : parts per Million : Carbon13
(thousandths)

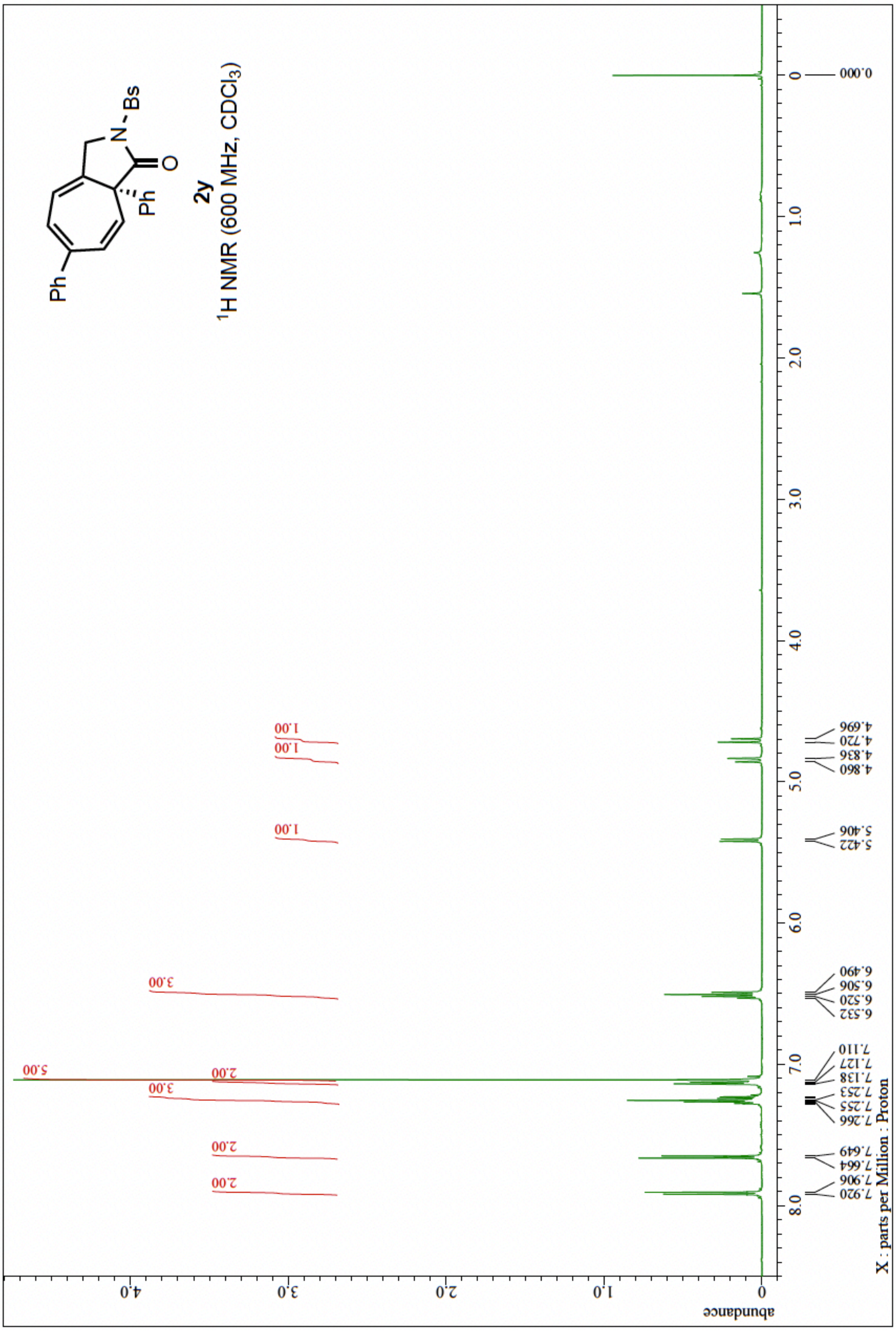

Ph
N-Bs
O
Ph
2y
^{1}H NMR (600 MHz, CDCl$_3$)
0.000
4.696
4.720
4.836
4.860
5.406
5.422
6.490
6.506
6.520
6.532
7.110
7.127
7.138
7.253
7.255
7.266
7.649
7.664
7.906
7.920
1.00
1.00
1.00
3.00
5.00
2.00
3.00
2.00
2.00
X : parts per Million : Proton
abundance

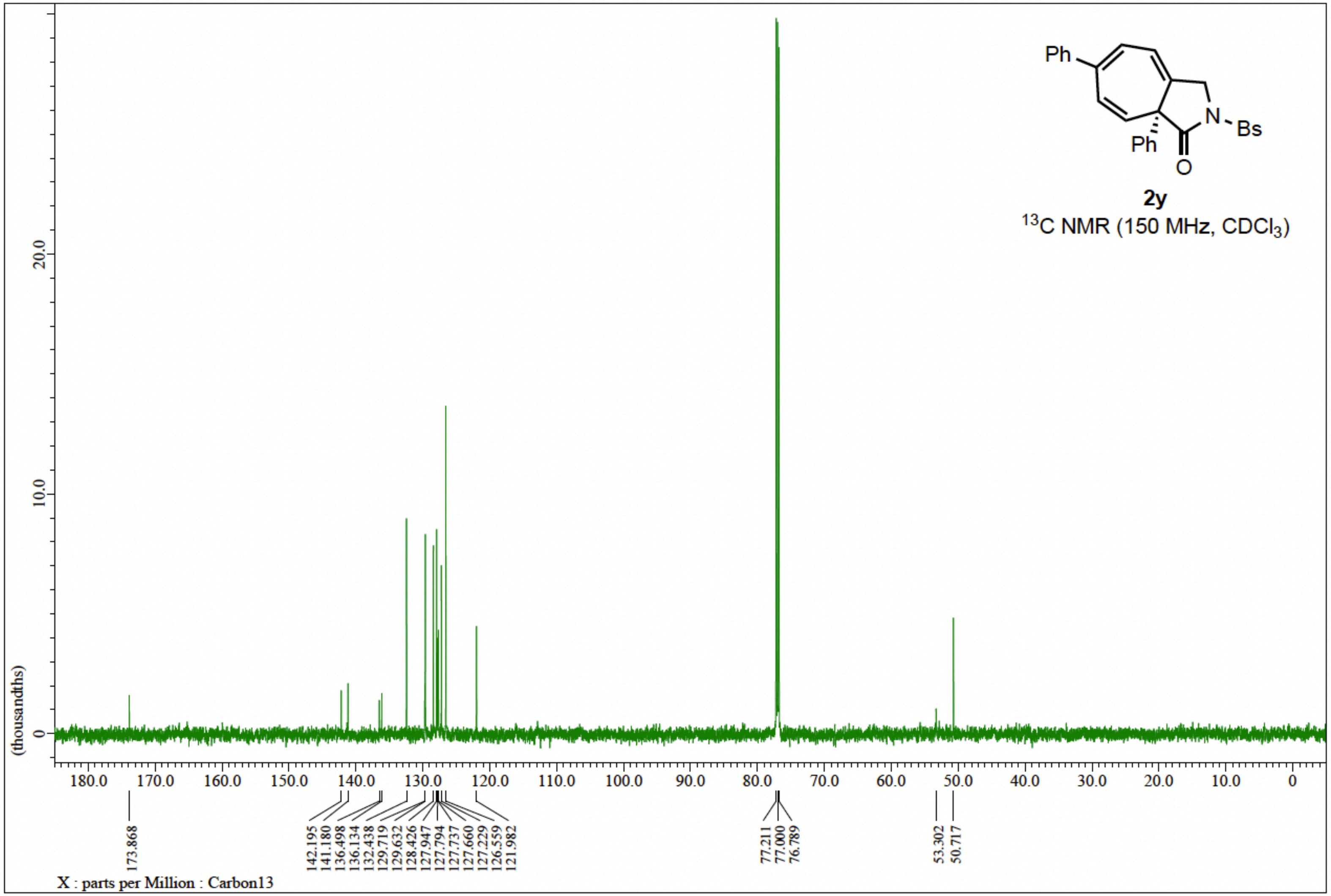

Ph
Ph
N
Bs
O
2y
^{13}C NMR (150 MHz, CDCl$_3$)
(thousandths)
0
10.0
20.0
180.0
170.0
160.0
150.0
140.0
130.0
120.0
110.0
100.0
90.0
80.0
70.0
60.0
50.0
40.0
30.0
20.0
10.0
0
173.868
142.195
141.180
136.498
136.134
132.438
129.719
129.632
128.426
127.947
127.794
127.737
127.660
127.229
126.559
121.982
77.211
77.000
76.789
53.302
50.717
X : parts per Million : Carbon13

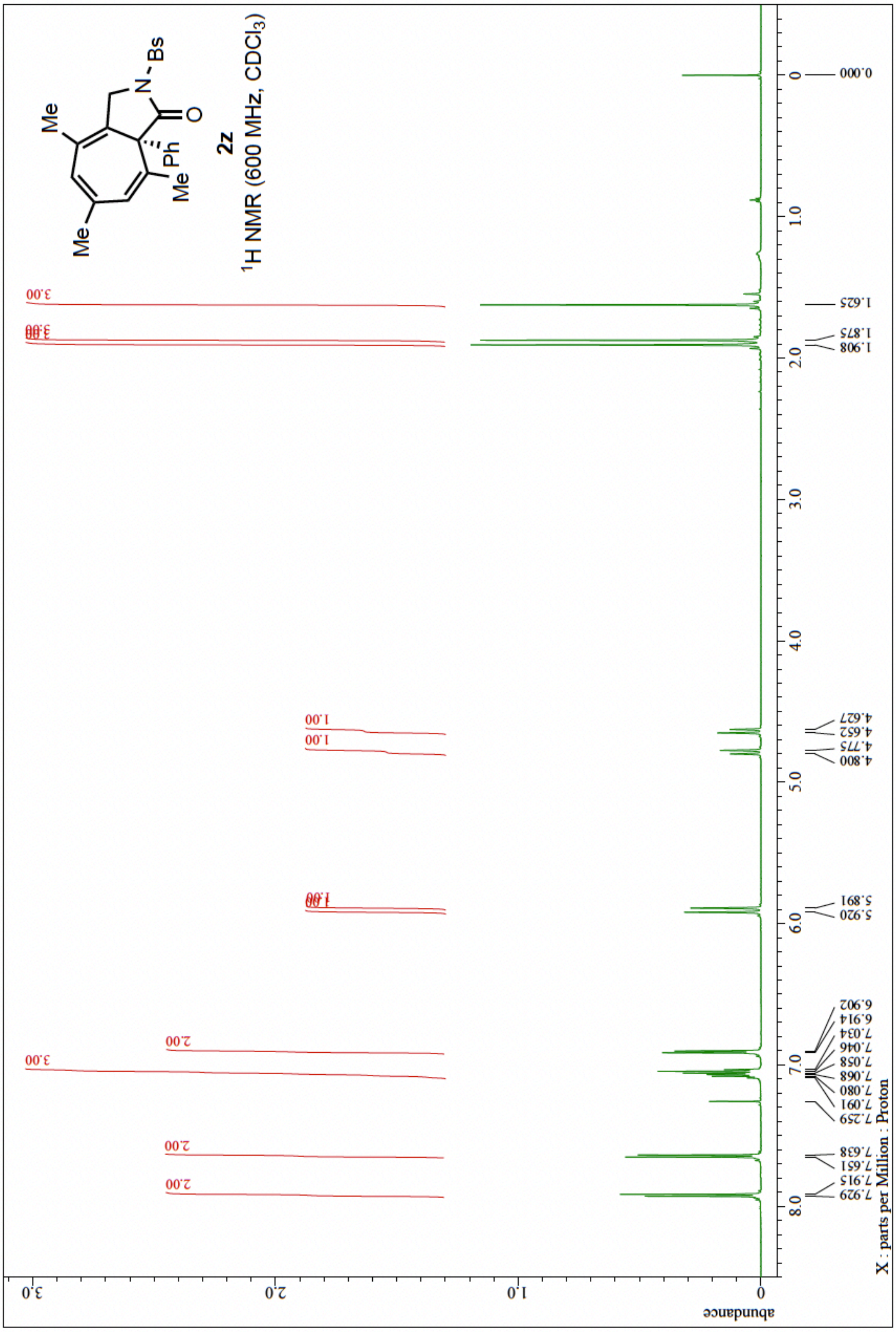
2z
1H NMR (600 MHz, CDCl3)
X : parts per Million : Proton
abundance

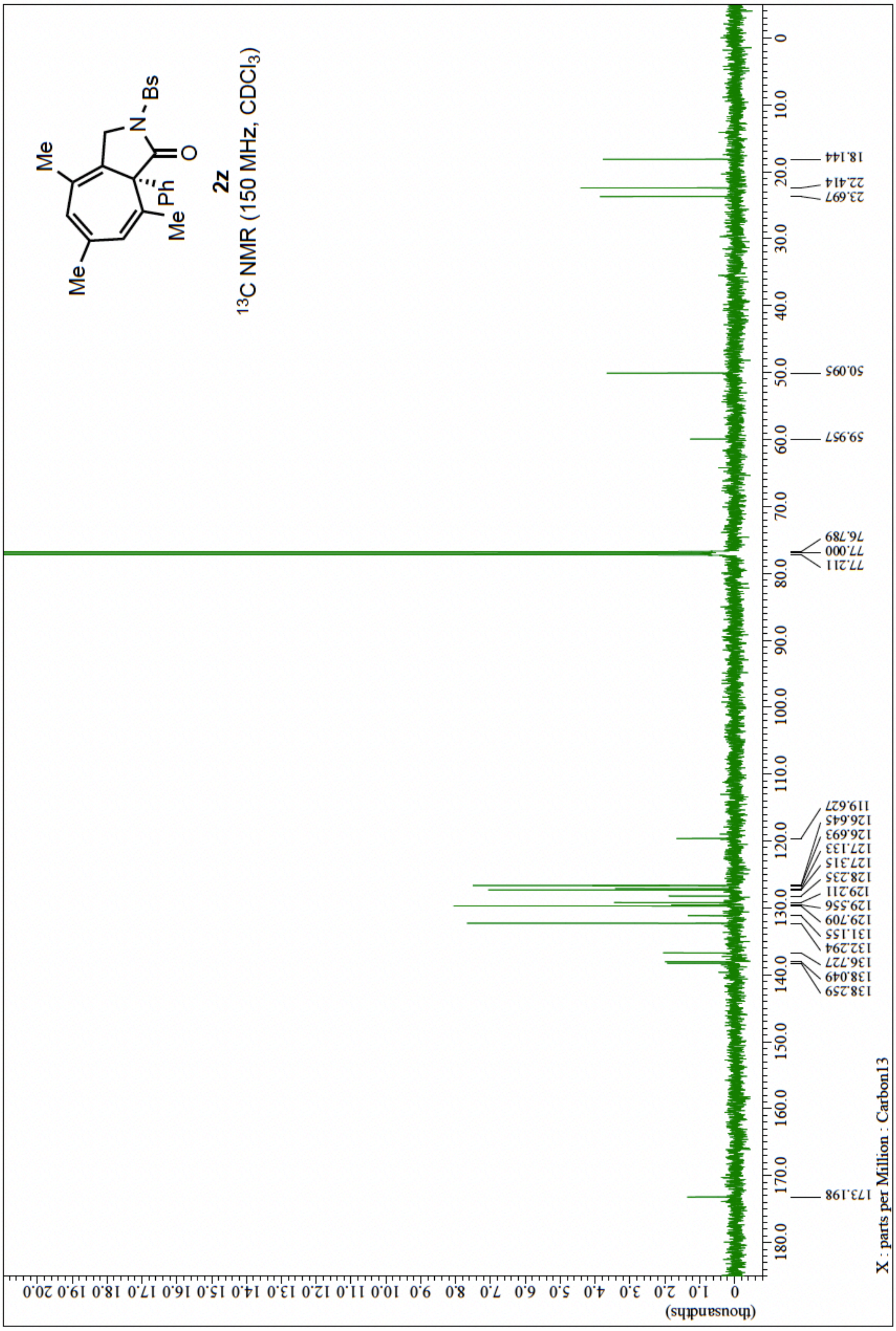
Me
N
Bs
O
Ph
Me
Me
2z
13C NMR (150 MHz, CDCl3)
18.144
22.414
23.697
50.095
59.957
76.789
77.000
77.211
119.627
126.645
126.693
127.133
127.315
128.235
129.211
129.556
129.709
131.155
132.294
136.727
138.049
138.259
173.198
X : parts per Million : Carbon13
(thousandths)

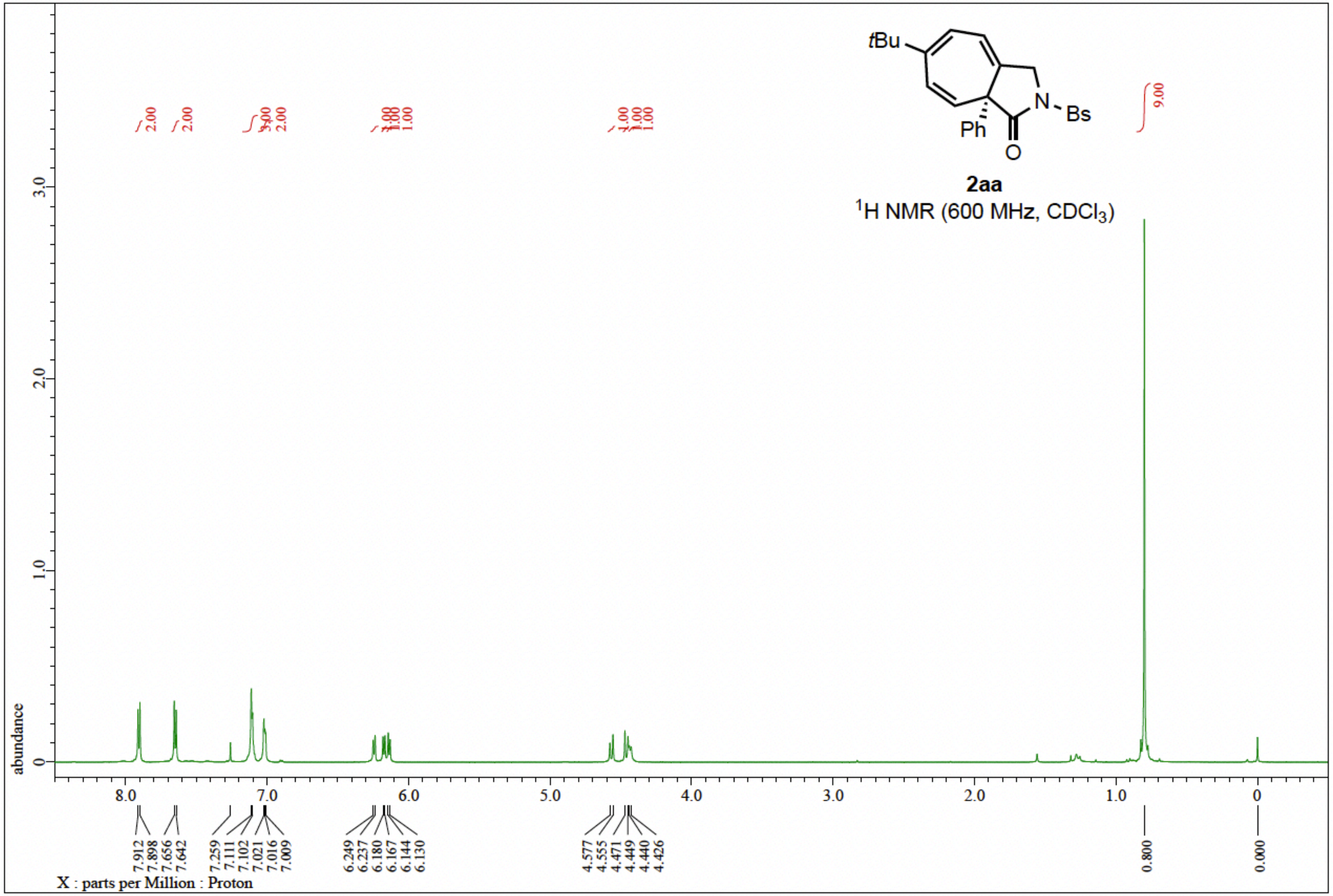
tBu
N
Bs
Ph
O
2aa
1H NMR (600 MHz, CDCl3)
2.00
2.00
2.00
1.00
1.00
1.00
1.00
1.00
1.00
9.00
abundance
0
1.0
2.0
3.0
8.0
7.0
6.0
5.0
4.0
3.0
2.0
1.0
0
7.912
7.898
7.656
7.642
7.259
7.111
7.102
7.021
7.016
7.009
6.249
6.237
6.180
6.167
6.144
6.130
4.577
4.555
4.471
4.449
4.440
4.426
0.800
0.000
X : parts per Million : Proton

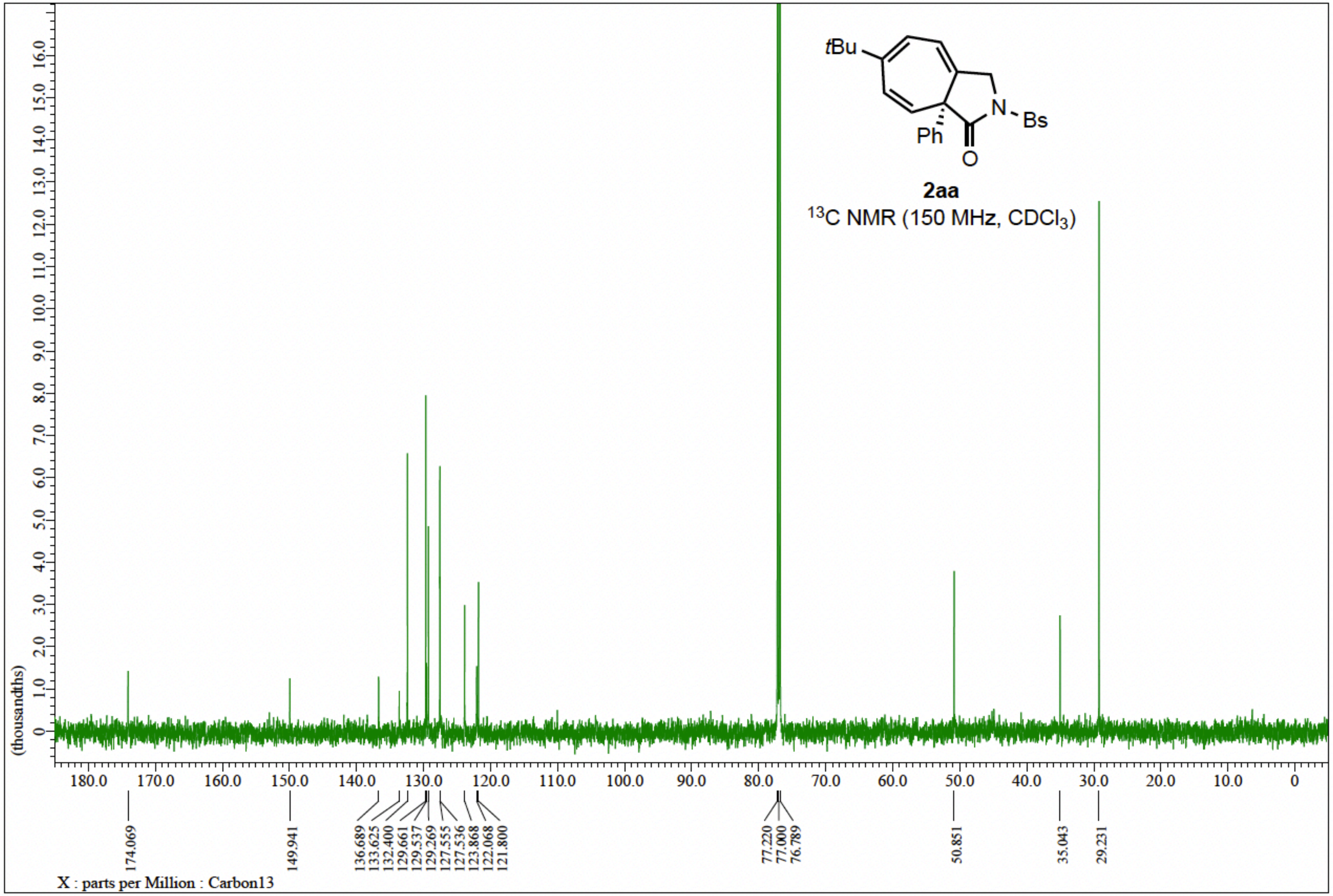

2aa

^{13}C NMR (150 MHz, $CDCl_3$)

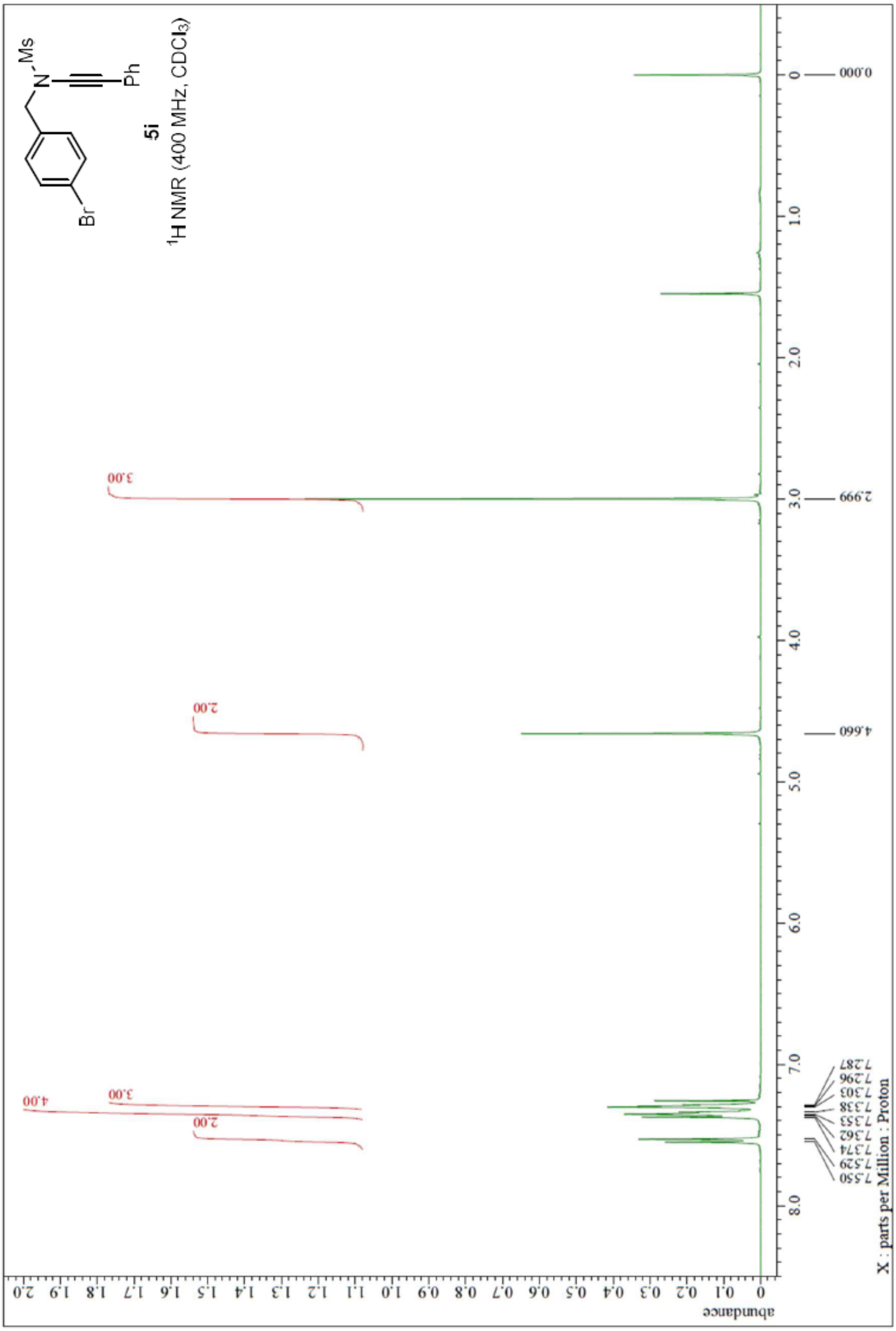
Br
Ms
N
Ph
5i
1H NMR (400 MHz, CDCl3)
0.000
2.999
4.660
7.287
7.296
7.303
7.338
7.353
7.362
7.374
7.529
7.550
3.00
2.00
4.00
3.00
2.00
X : parts per Million : Proton
abundance

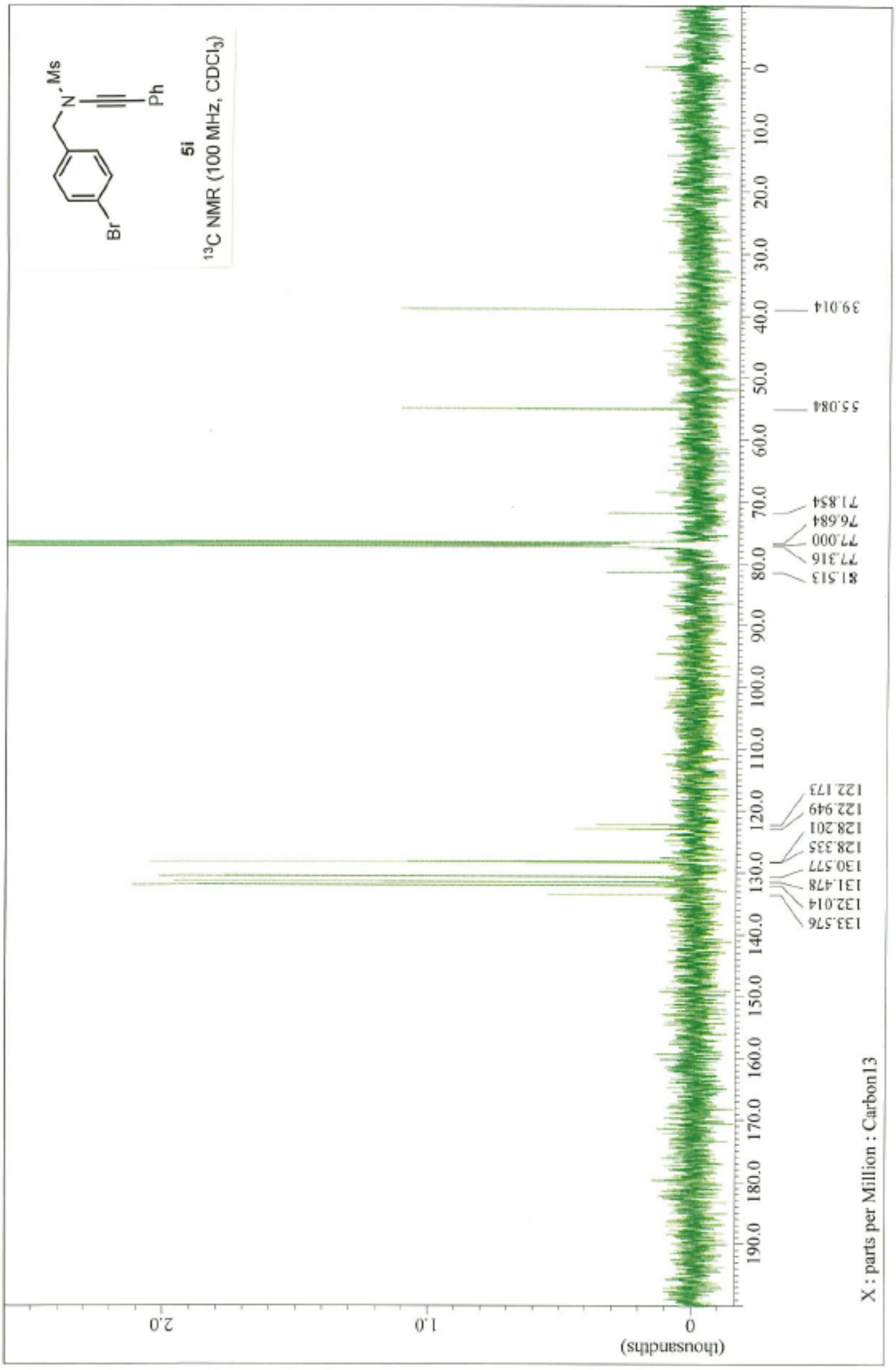
Br
N
Ms
Ph
5i
13C NMR (100 MHz, CDCl3)
39.014
55.084
71.854
76.684
77.000
77.316
81.513
122.173
122.949
128.201
128.335
130.577
131.478
132.014
133.576
X : parts per Million : Carbon13
(thousandths)

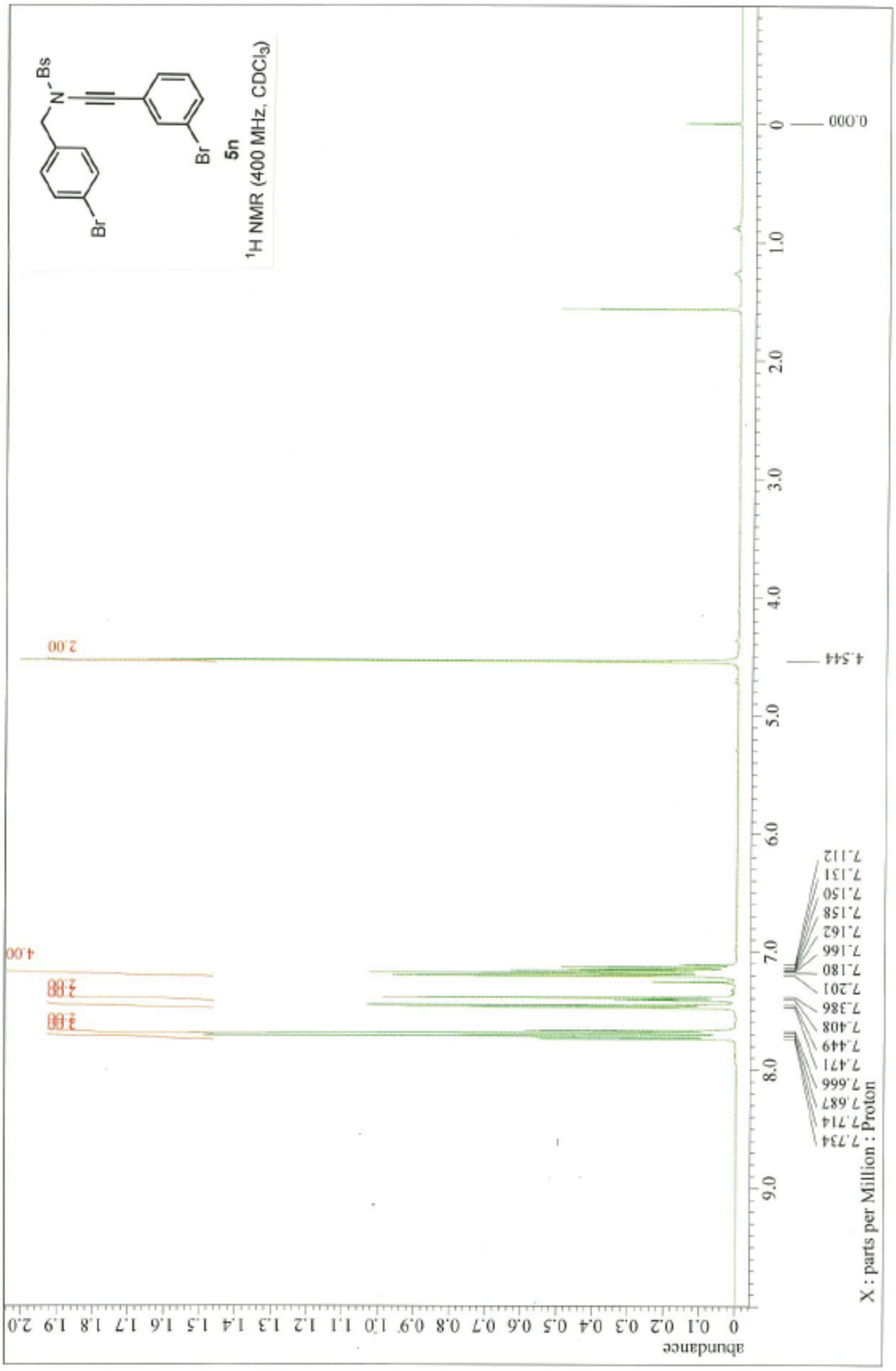
Br
Br
N
Bs
5n
1H NMR (400 MHz, CDCl3)
0.000
4.544
7.112
7.131
7.150
7.158
7.162
7.166
7.180
7.201
7.386
7.408
7.449
7.471
7.666
7.687
7.714
7.734
2.00
4.00
X : parts per Million : Proton
abundance

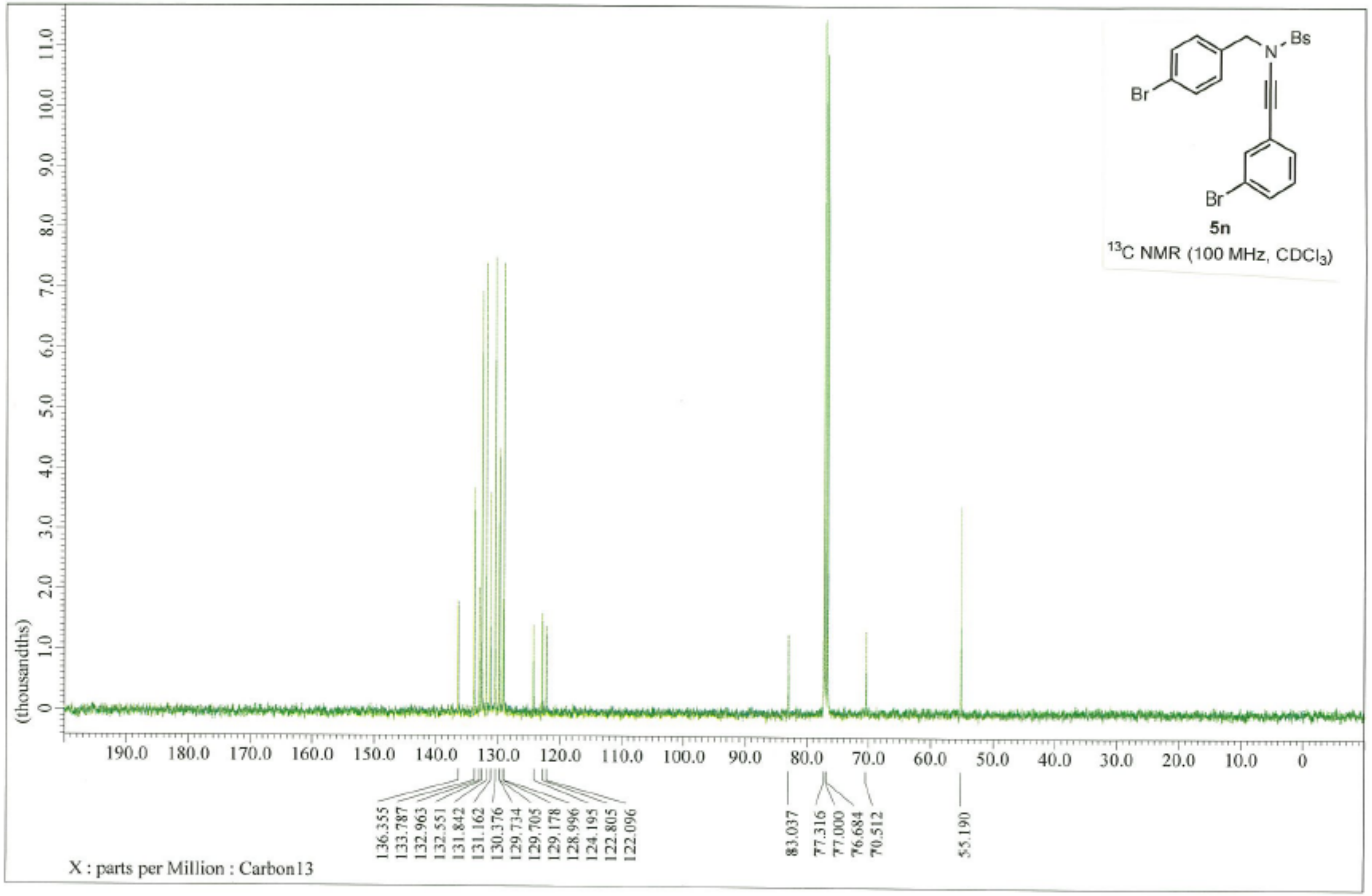
Br
N
Bs
Br
5n
^{13}C NMR (100 MHz, CDCl$_3$)
(thousandths)
0
1.0
2.0
3.0
4.0
5.0
6.0
7.0
8.0
9.0
10.0
11.0
190.0
180.0
170.0
160.0
150.0
140.0
130.0
120.0
110.0
100.0
90.0
80.0
70.0
60.0
50.0
40.0
30.0
20.0
10.0
0
136.355
133.787
132.963
132.551
131.842
131.162
130.376
129.734
129.705
129.178
128.996
124.195
122.805
122.096
83.037
77.316
77.000
76.684
70.512
55.190
X : parts per Million : Carbon13

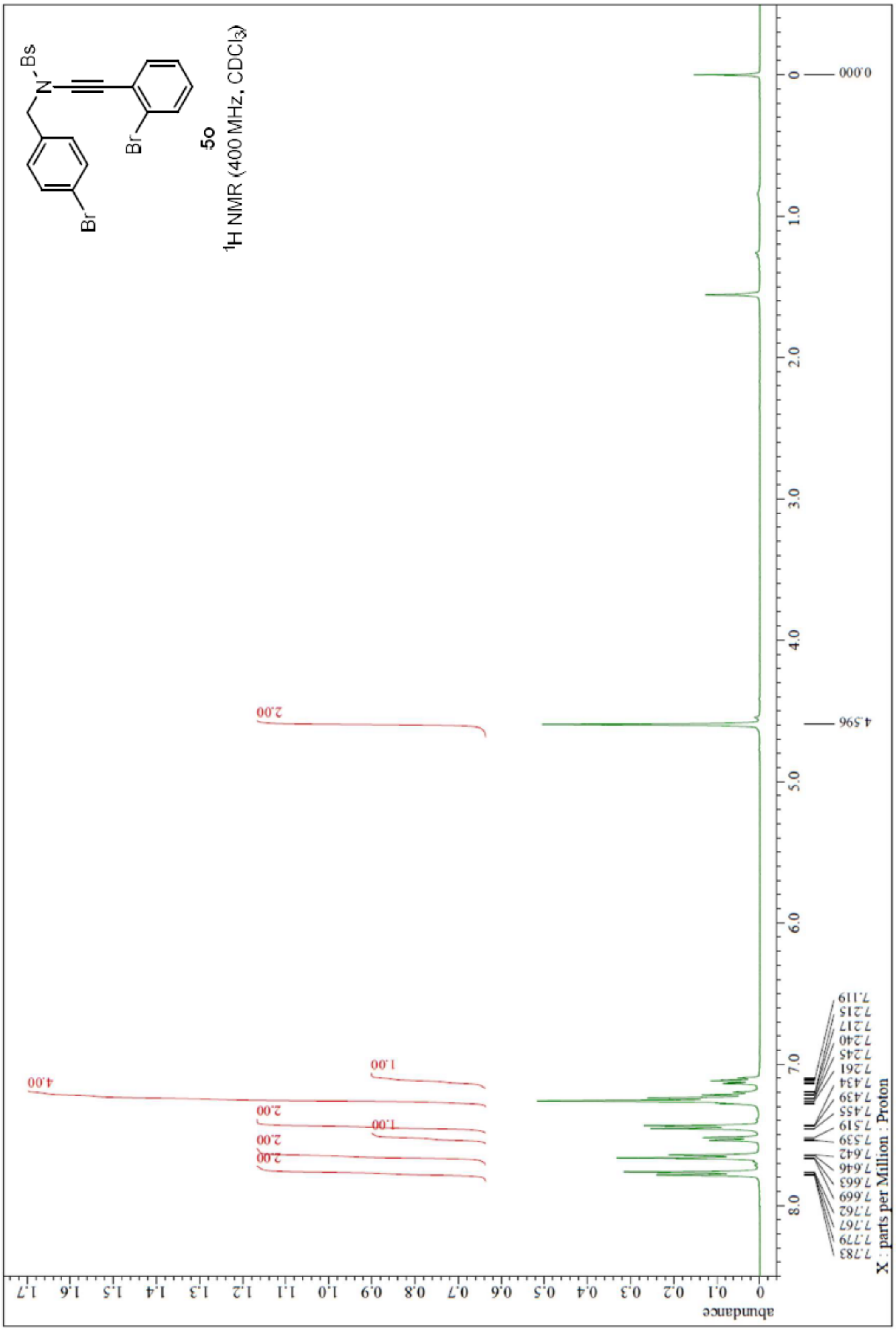
Bs
N
Br
Br
5o
1H NMR (400 MHz, CDCl3)
7.783
7.779
7.767
7.762
7.669
7.663
7.646
7.642
7.539
7.519
7.455
7.439
7.434
7.261
7.245
7.240
7.217
7.215
7.119
4.596
0.000
X : parts per Million : Proton
abundance

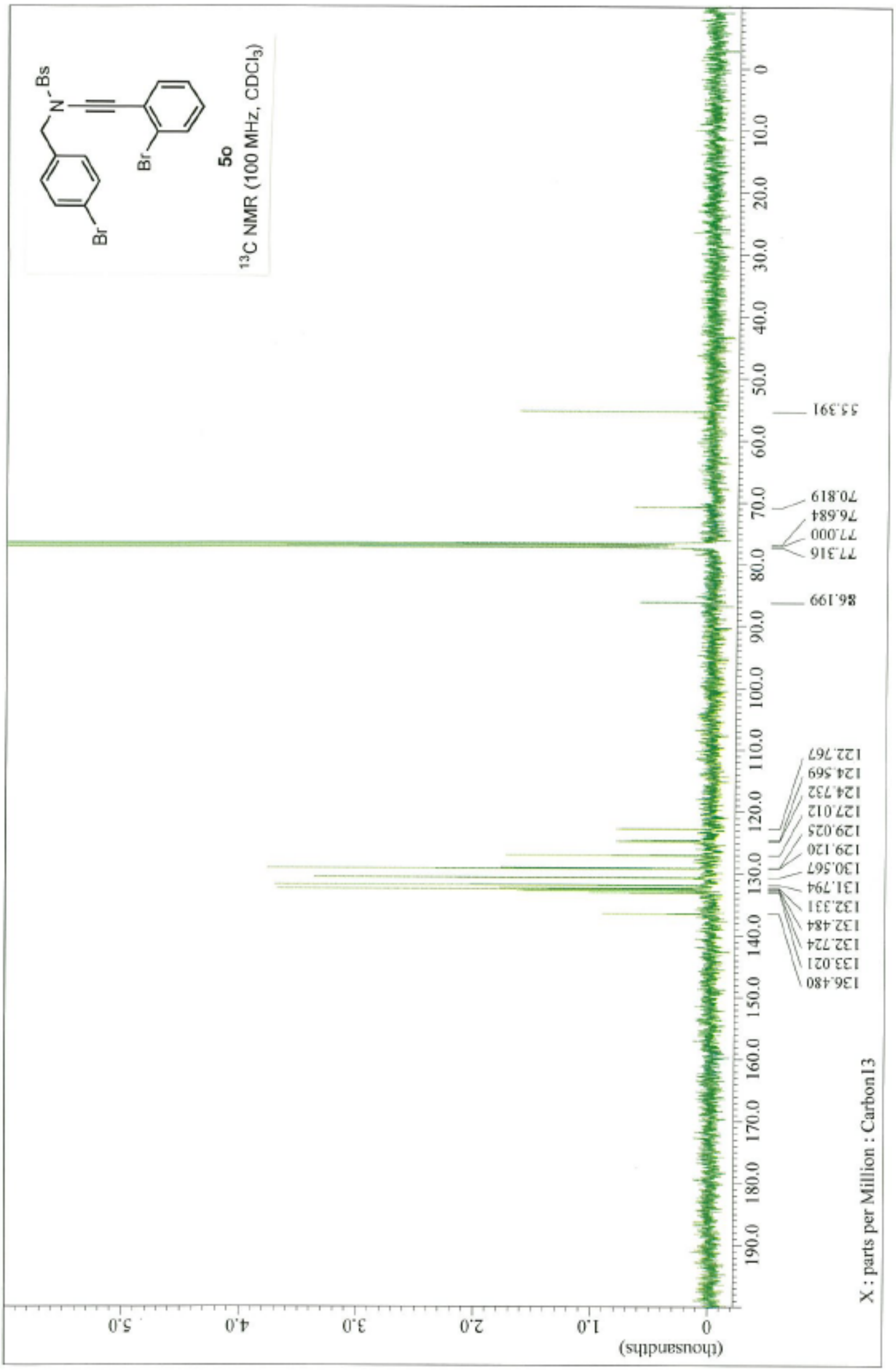
Br
Br
N
Bs
5o
13C NMR (100 MHz, CDCl3)
55.391
70.819
76.684
77.000
77.316
86.199
122.767
124.569
124.732
127.012
129.025
129.120
130.567
131.794
132.331
132.484
132.724
133.021
136.480
X : parts per Million : Carbon13
(thousandths)

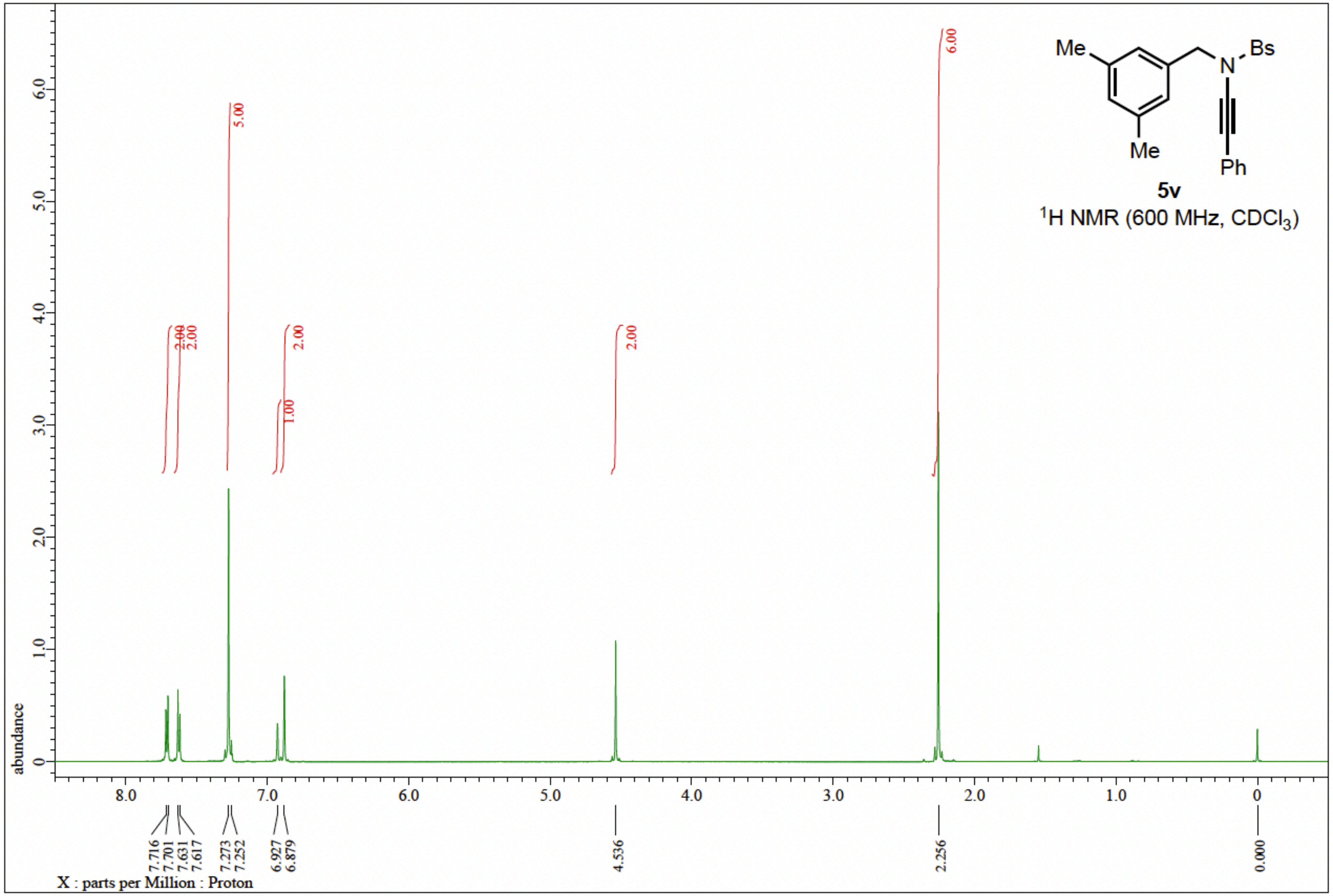
Me
Me
N
Bs
Ph
5v
1H NMR (600 MHz, CDCl3)
6.00
5.00
2.00
2.00
1.00
2.00
2.00
2.00
abundance
X : parts per Million : Proton
7.716
7.701
7.631
7.617
7.273
7.252
6.927
6.879
4.536
2.256
0.000
8.0
7.0
6.0
5.0
4.0
3.0
2.0
1.0
0

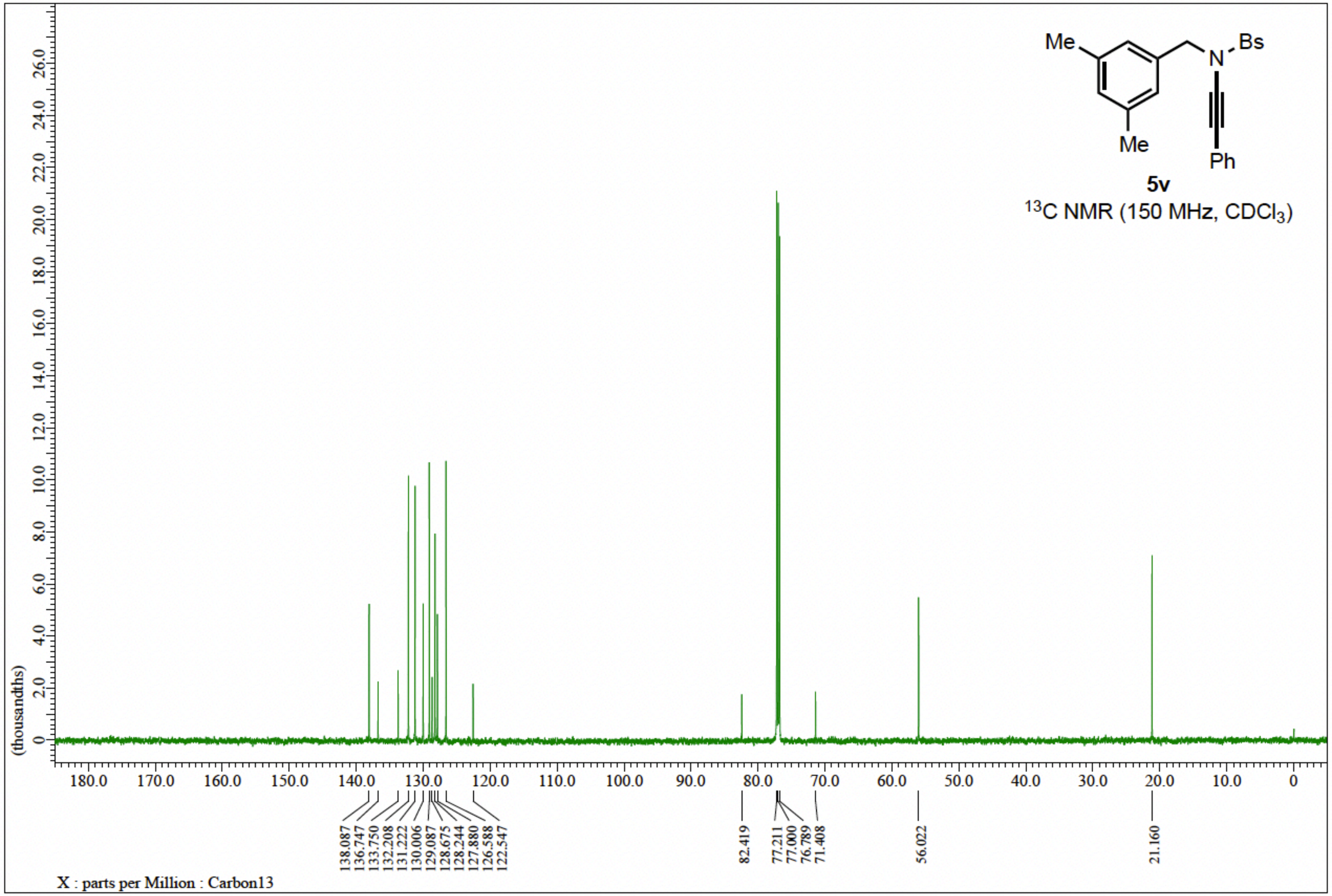

Me
Me
N
Bs
Ph
5v
13C NMR (150 MHz, CDCl3)
(thousandths)
0
2.0
4.0
6.0
8.0
10.0
12.0
14.0
16.0
18.0
20.0
22.0
24.0
26.0
180.0
170.0
160.0
150.0
140.0
130.0
120.0
110.0
100.0
90.0
80.0
70.0
60.0
50.0
40.0
30.0
20.0
10.0
138.087
136.747
133.750
132.208
131.222
130.006
129.087
128.675
128.244
127.880
126.588
122.547
82.419
77.211
77.000
76.789
71.408
56.022
21.160
X : parts per Million : Carbon13

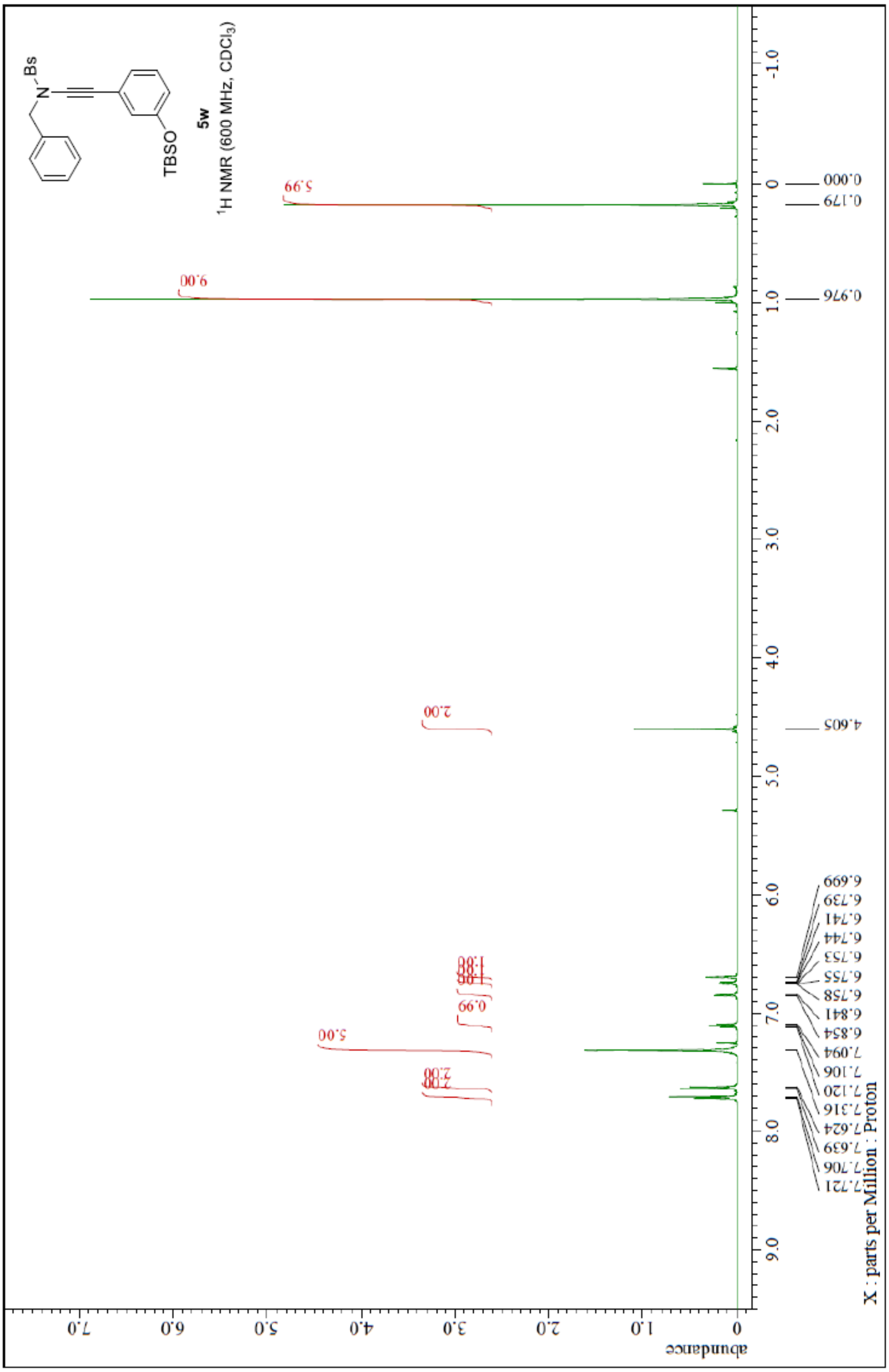

TBSO
N
Bs
5w
1H NMR (600 MHz, CDCl3)
0.000
0.179
0.976
4.605
6.699
6.739
6.741
6.744
6.753
6.755
6.758
6.841
6.854
7.094
7.106
7.120
7.316
7.624
7.639
7.706
7.721
5.99
9.00
2.00
5.00
0.99
2.00
X : parts per Million : Proton
abundance

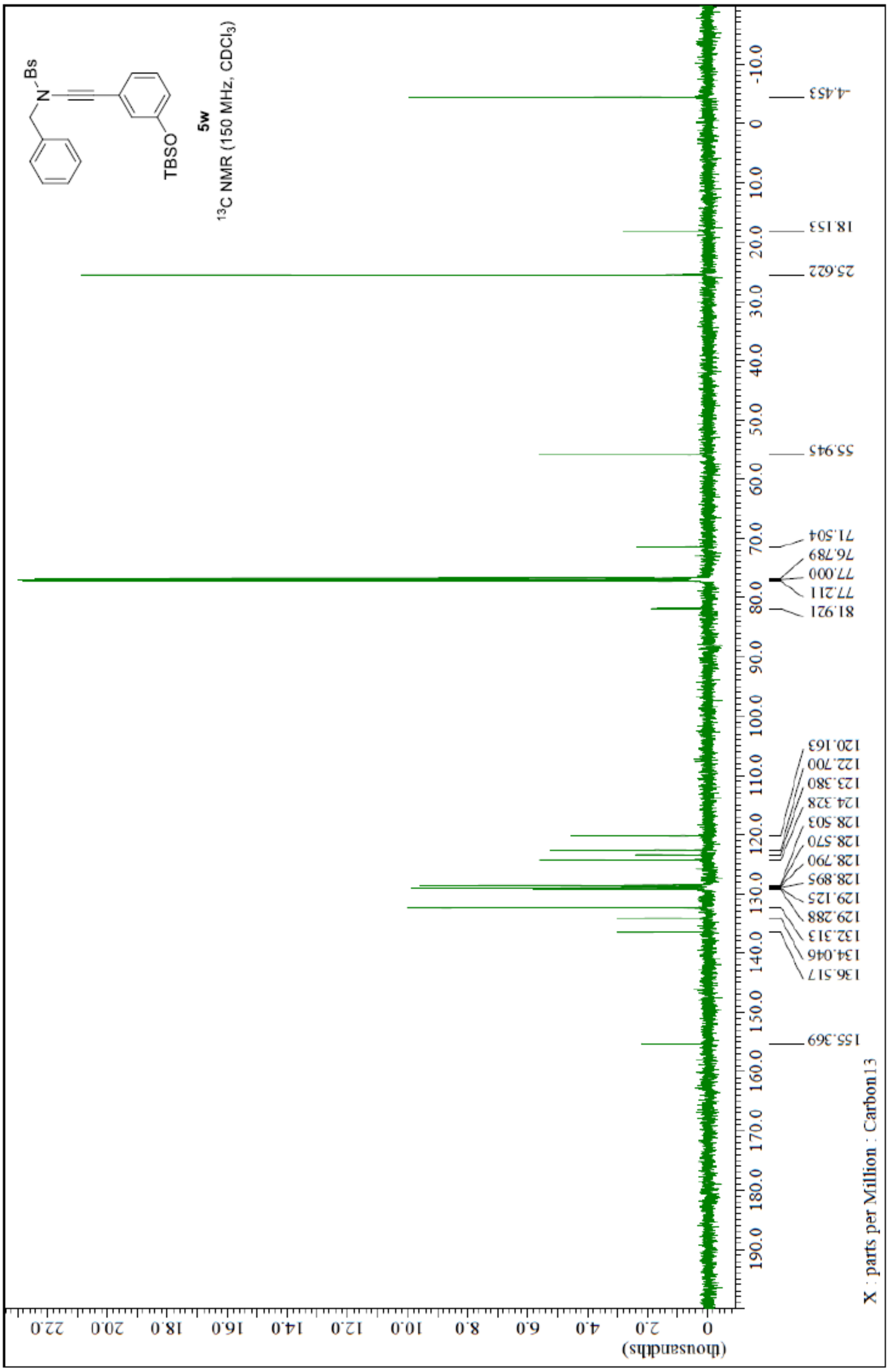
TBSO
N
Bs
5w
13C NMR (150 MHz, CDCl3)
-4.453
18.153
25.622
55.945
71.504
76.789
77.000
77.211
81.921
120.163
122.700
123.380
124.328
128.503
128.570
128.790
128.895
129.125
129.288
132.313
134.046
136.517
155.369
X : parts per Million : Carbon13
(thousandths)

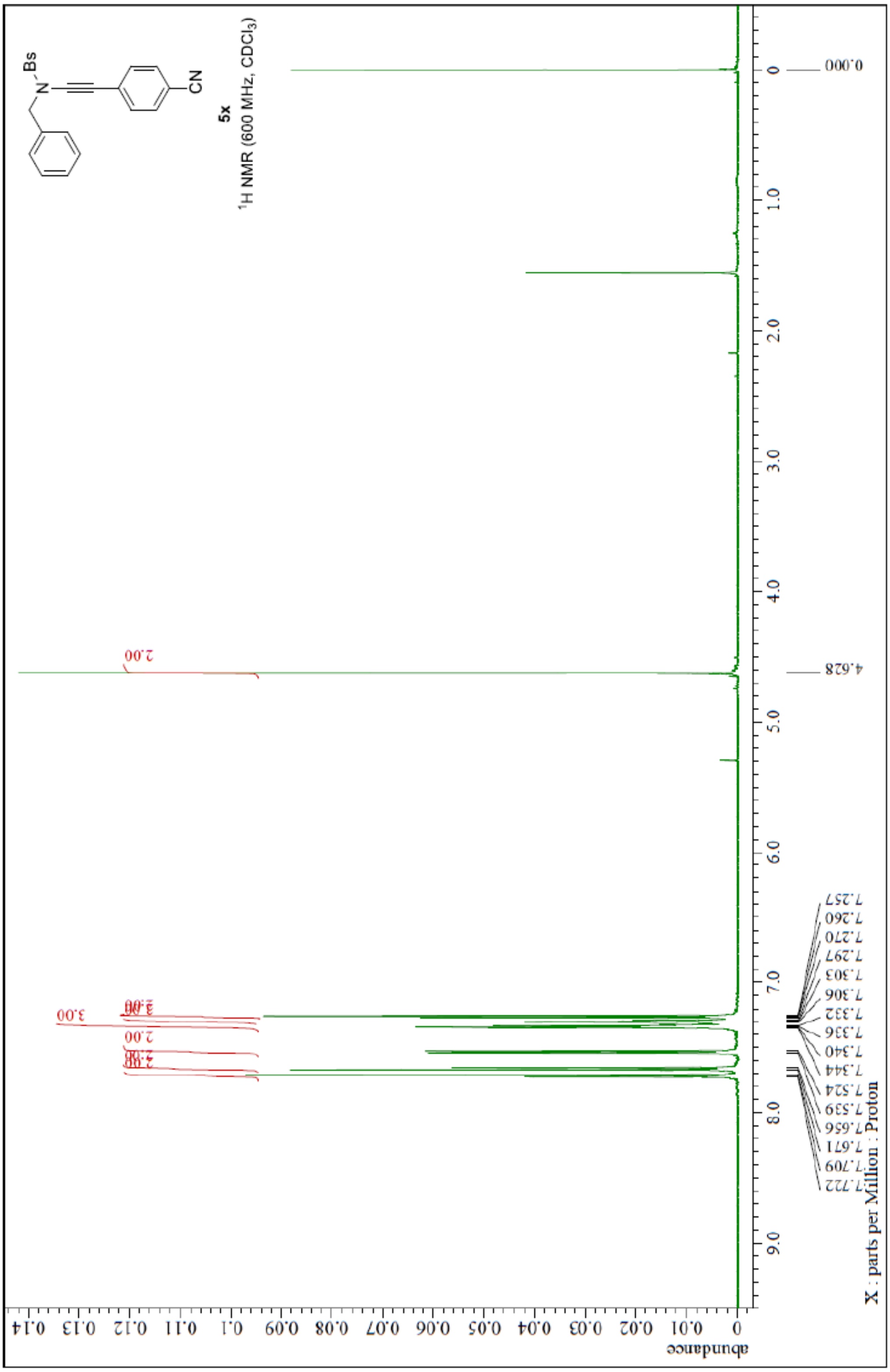

5x
^{1}H NMR (600 MHz, $CDCl_3$)
Bs
N
CN
X : parts per Million : Proton
abundance
0.000
4.628
7.257
7.260
7.270
7.297
7.303
7.306
7.332
7.336
7.340
7.344
7.524
7.539
7.656
7.671
7.709
7.722
2.00
3.00

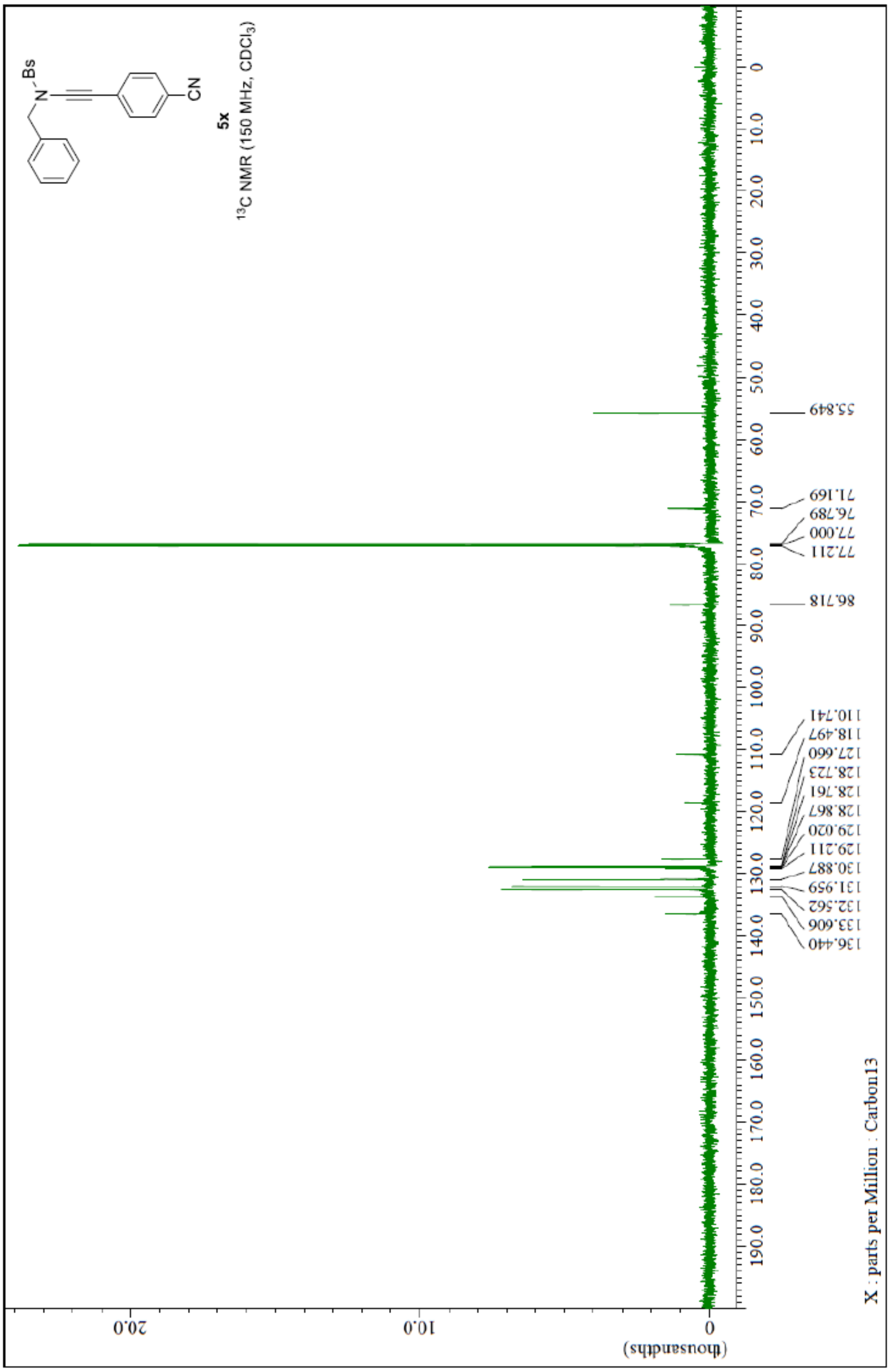
Bs
N
CN
5x
13C NMR (150 MHz, CDCl3)
55.849
71.169
76.789
77.000
77.211
86.718
110.741
118.497
127.660
128.723
128.761
128.867
129.020
129.211
130.887
131.959
132.562
133.606
136.440
X : parts per Million : Carbon13
(thousandths)

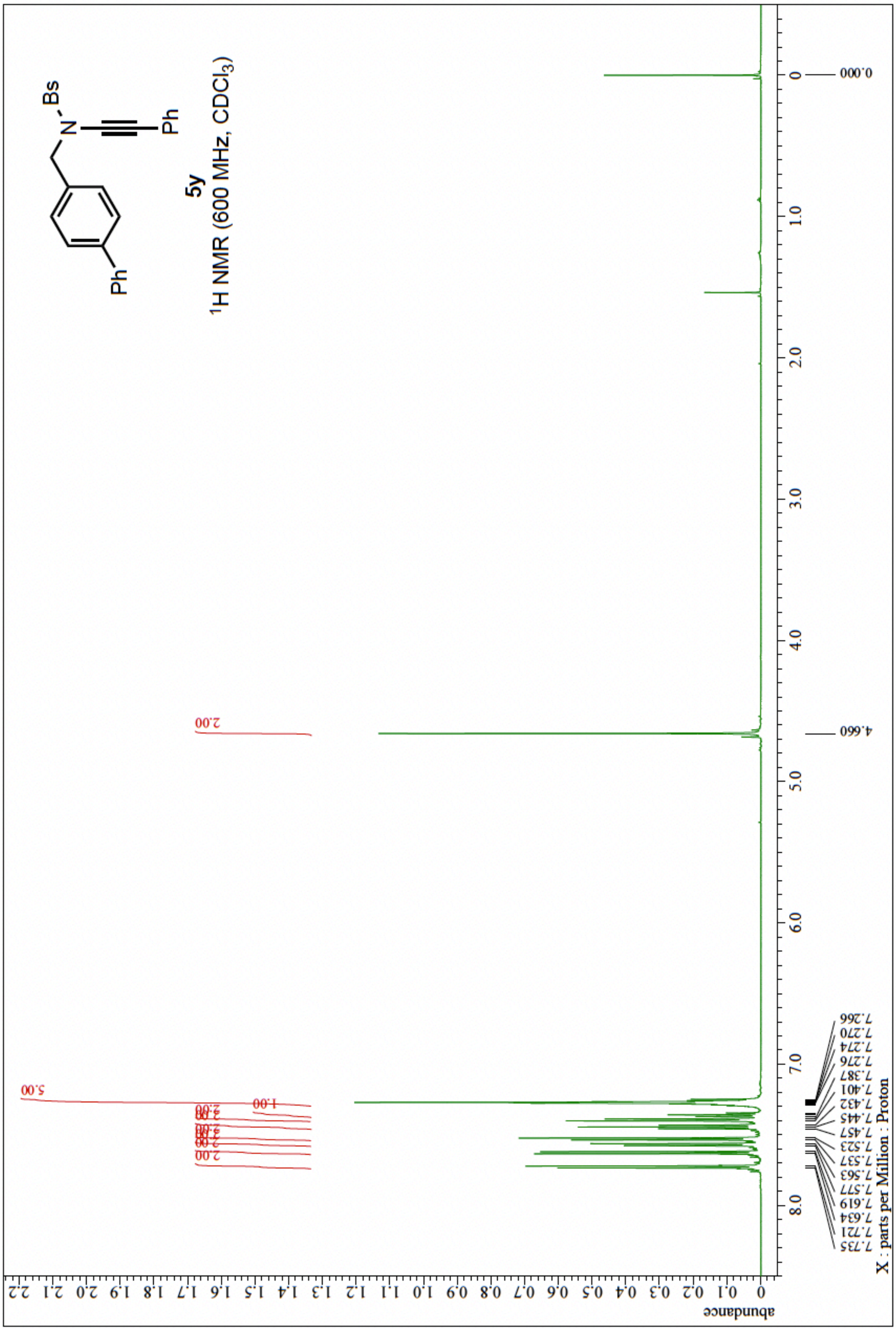

Bs
N
Ph
Ph
5y
1H NMR (600 MHz, CDCl3)
0.000
4.660
7.266
7.270
7.274
7.276
7.387
7.401
7.432
7.445
7.457
7.523
7.537
7.563
7.577
7.619
7.634
7.721
7.735
X : parts per Million : Proton
abundance

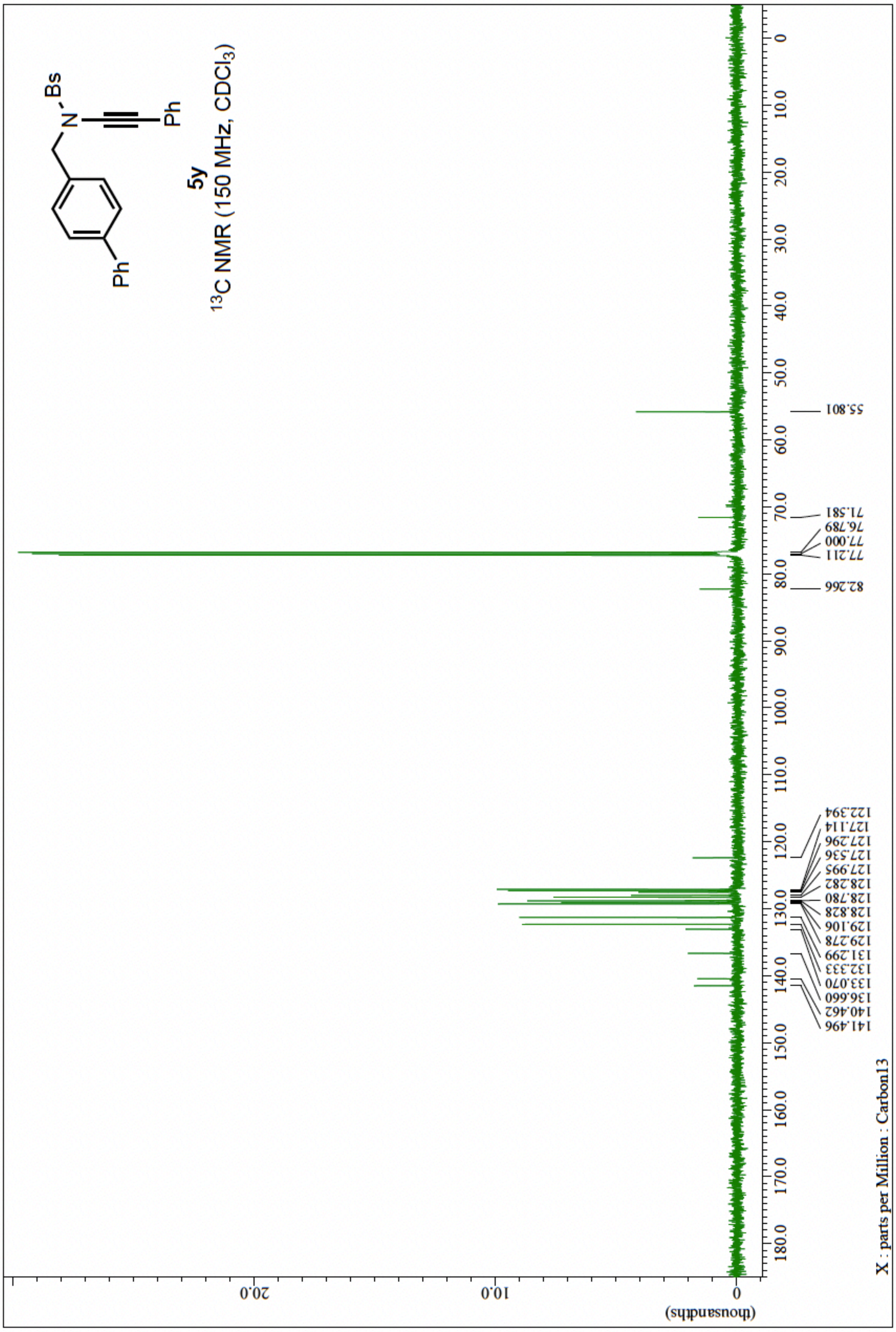
Ph
N
Bs
Ph
5y
13C NMR (150 MHz, CDCl3)
55.801
71.581
76.789
77.000
77.211
82.266
122.394
127.114
127.296
127.536
127.995
128.282
128.780
128.828
129.106
129.278
131.299
132.333
133.070
136.660
140.462
141.496
X : parts per Million : Carbon13
(thousandths)

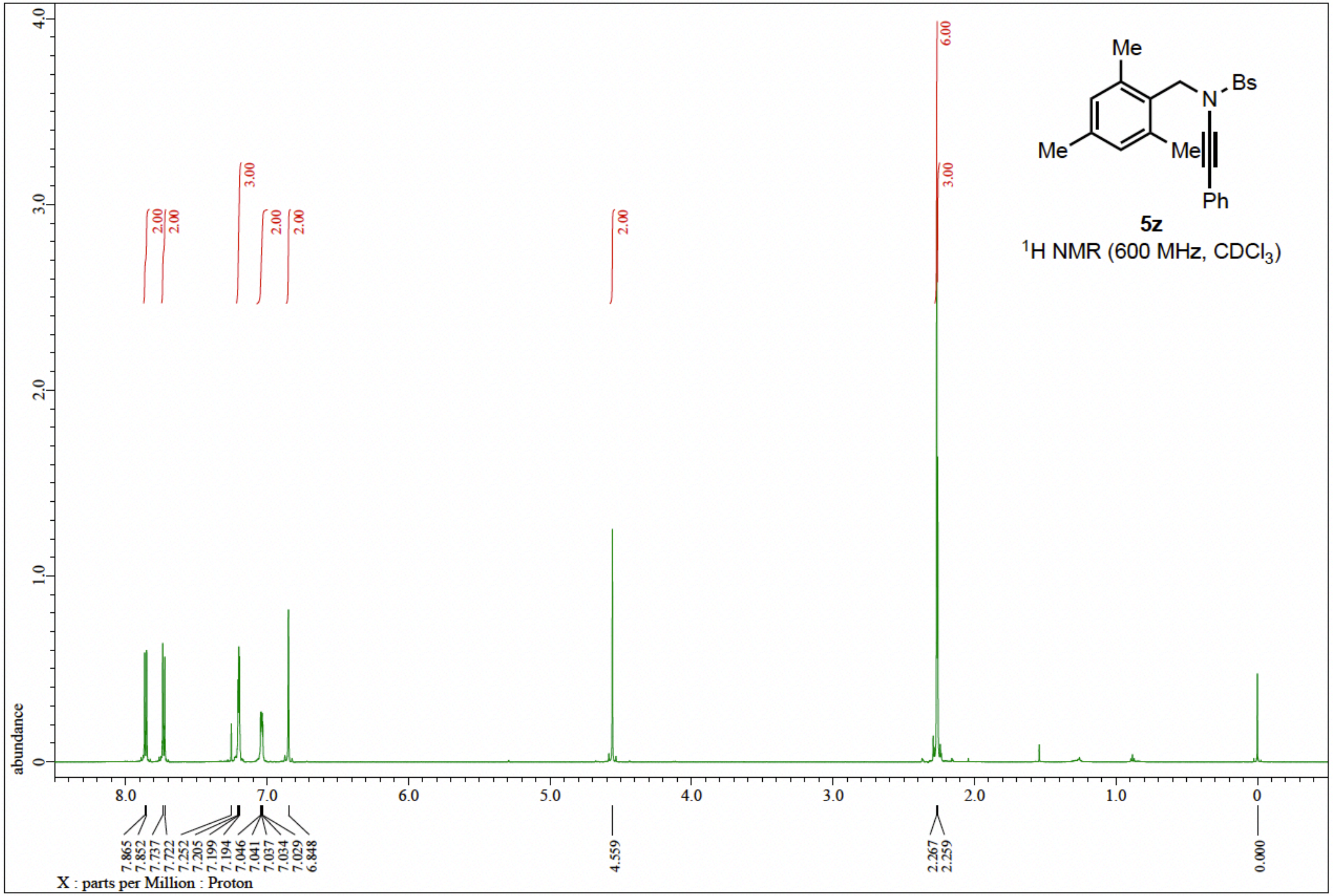
Me
N
Bs
Me
Me
Ph
5z
^{1}H NMR (600 MHz, $CDCl_3$)
abundance
X : parts per Million : Proton
7.865
7.852
7.737
7.722
7.252
7.205
7.199
7.194
7.046
7.041
7.037
7.034
7.029
6.848
4.559
2.267
2.259
0.000
2.00
2.00
3.00
2.00
2.00
2.00
3.00
6.00

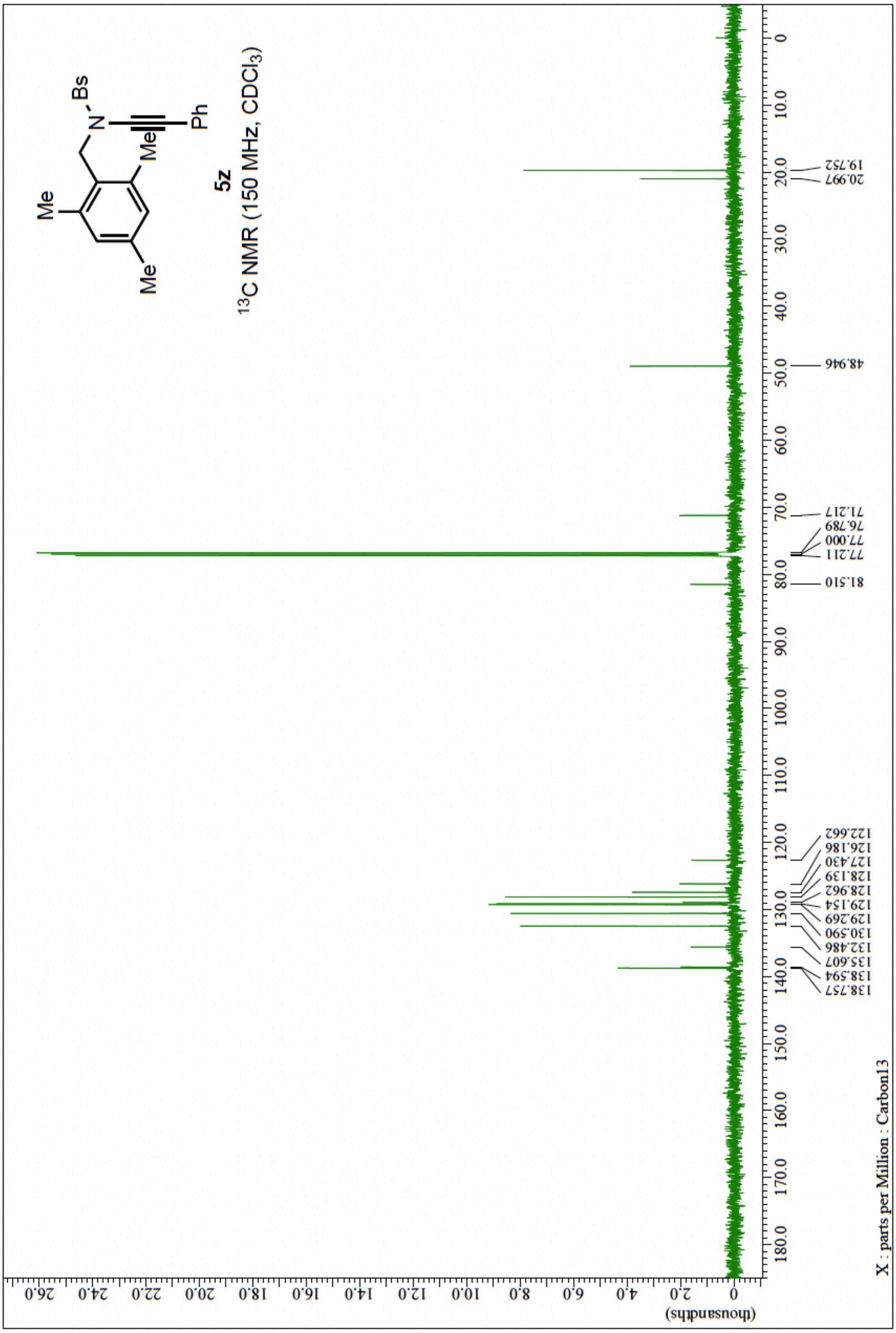
Me
Me
Me
N
Bs
Ph
5z
13C NMR (150 MHz, CDCl3)
19.752
20.997
48.946
71.217
76.789
77.000
77.211
81.510
122.662
126.186
127.430
128.139
128.962
129.154
129.269
130.590
132.486
135.607
138.594
138.757
X : parts per Million : Carbon13
(thousandths)

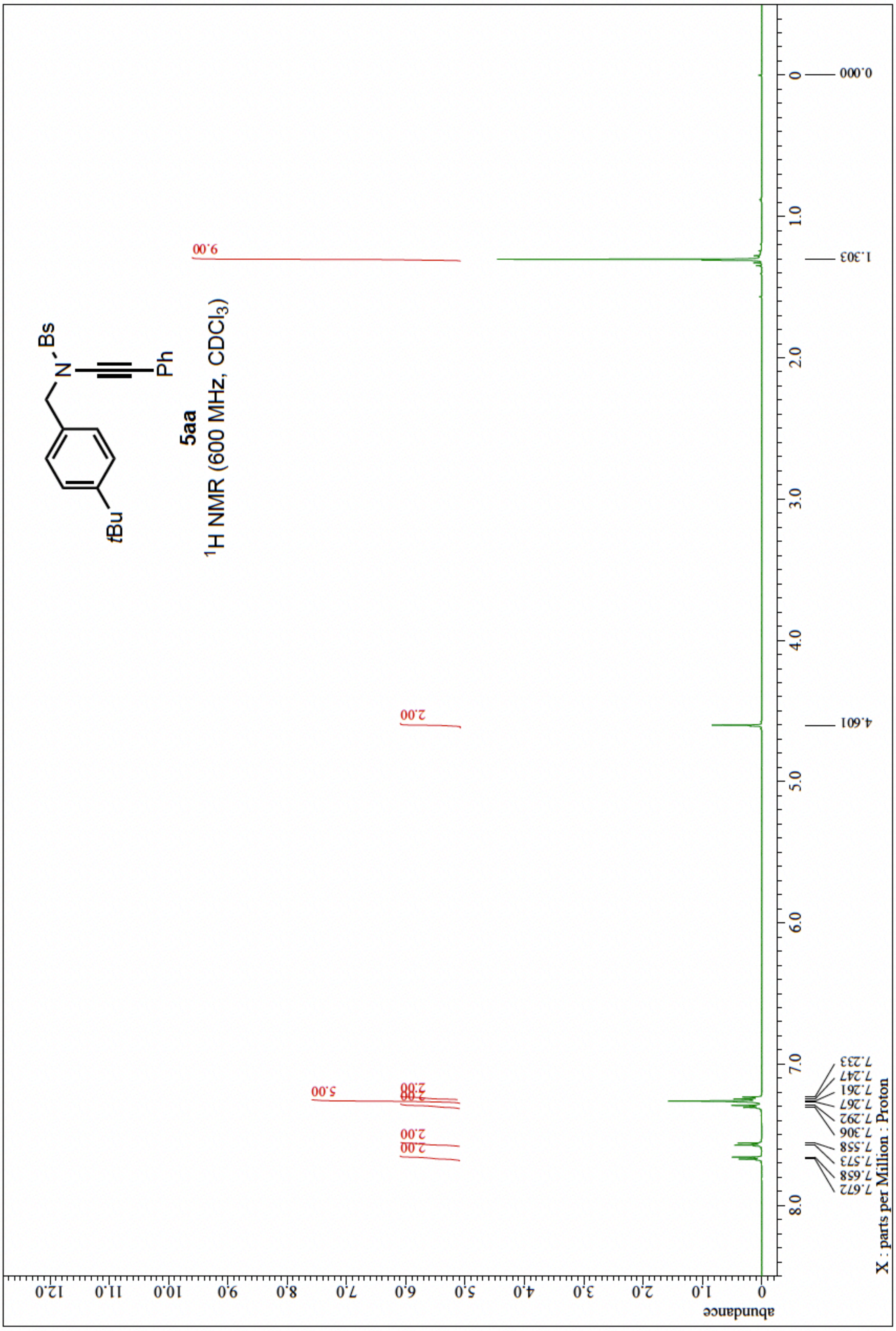
5aa
1H NMR (600 MHz, CDCl3)
tBu
Bs
N
Ph
X : parts per Million : Proton
abundance
0.000
1.303
4.601
7.233
7.247
7.261
7.267
7.292
7.306
7.558
7.573
7.658
7.672
9.00
2.00
5.00
2.00
2.00
2.00
2.00

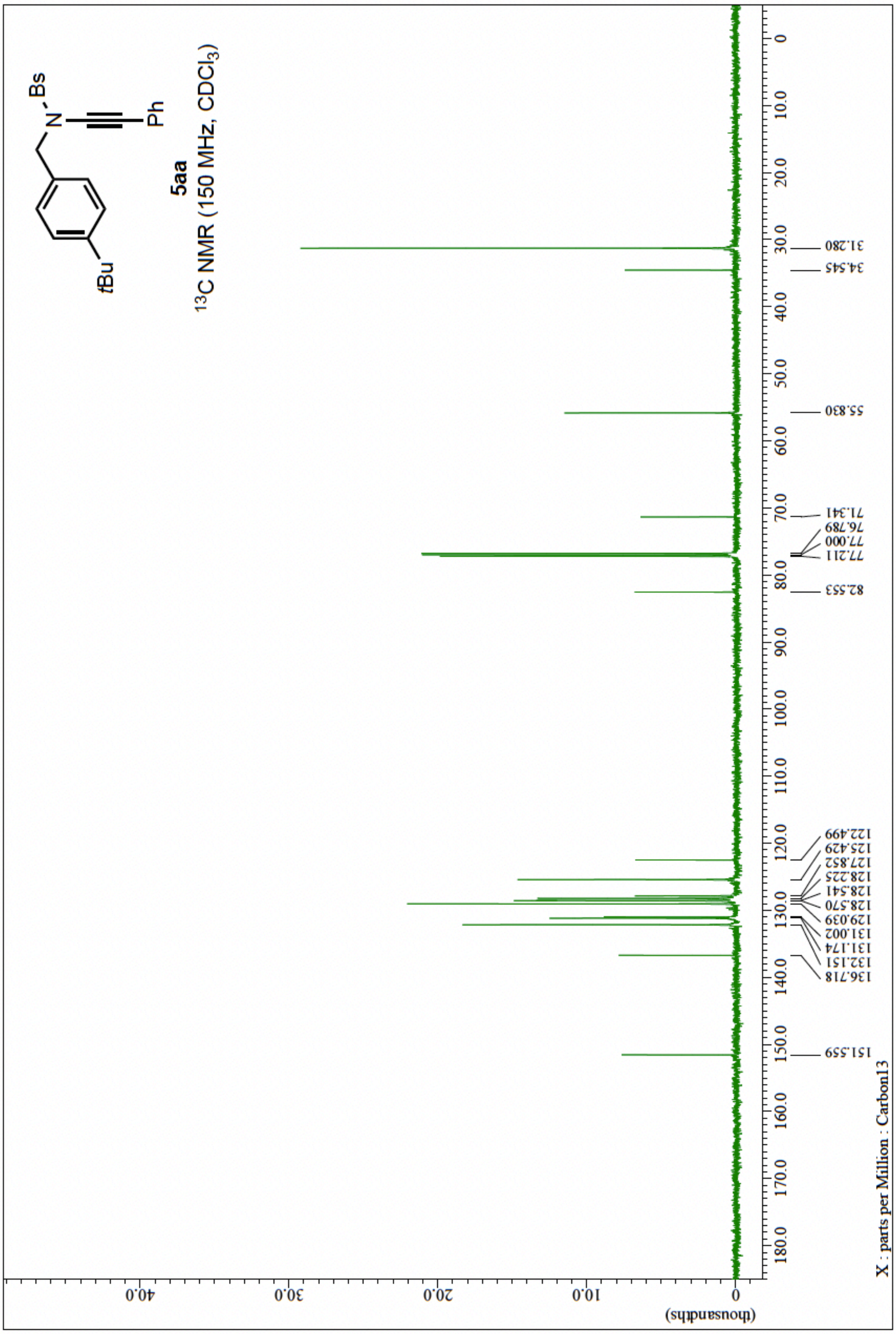

Bs
N
Ph
tBu
5aa
13C NMR (150 MHz, CDCl3)
31.280
34.545
55.830
71.341
76.789
77.000
77.211
82.553
122.499
125.429
127.852
128.225
128.541
128.570
129.039
131.002
131.174
132.151
136.718
151.559
X : parts per Million : Carbon13
(thousandths)

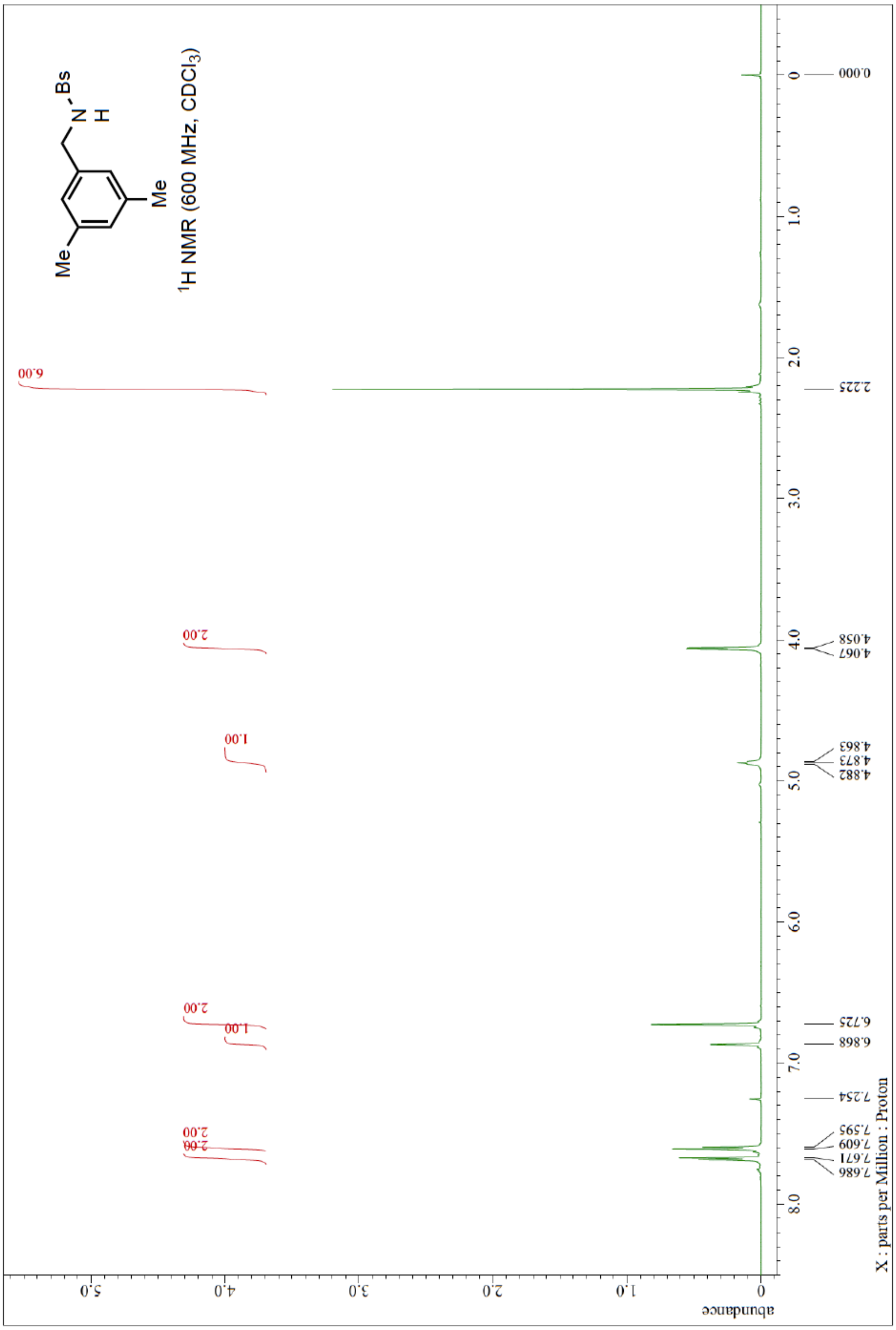
1H NMR (600 MHz, CDCl3)
Me
Me
N
H
Bs
0.000
2.225
4.058
4.067
4.863
4.873
4.882
6.725
6.868
7.254
7.595
7.609
7.671
7.686
6.00
2.00
1.00
2.00
1.00
2.00
2.00
X : parts per Million : Proton
abundance

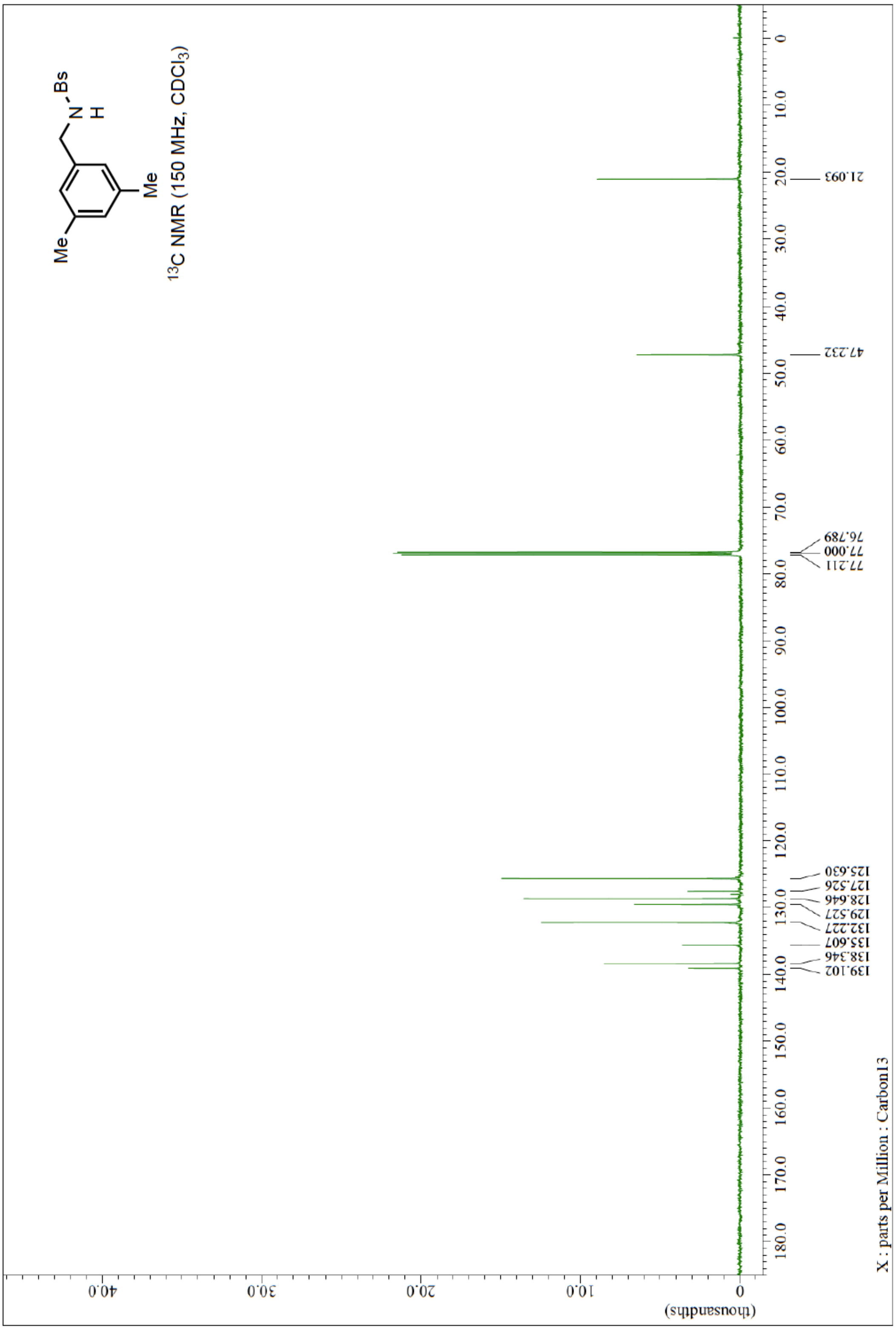

Me
Me
N
H
Bs
13C NMR (150 MHz, CDCl3)
21.093
47.232
76.789
77.000
77.211
125.630
127.526
128.646
129.527
132.227
135.607
138.346
139.102
X : parts per Million : Carbon13
(thousandths)

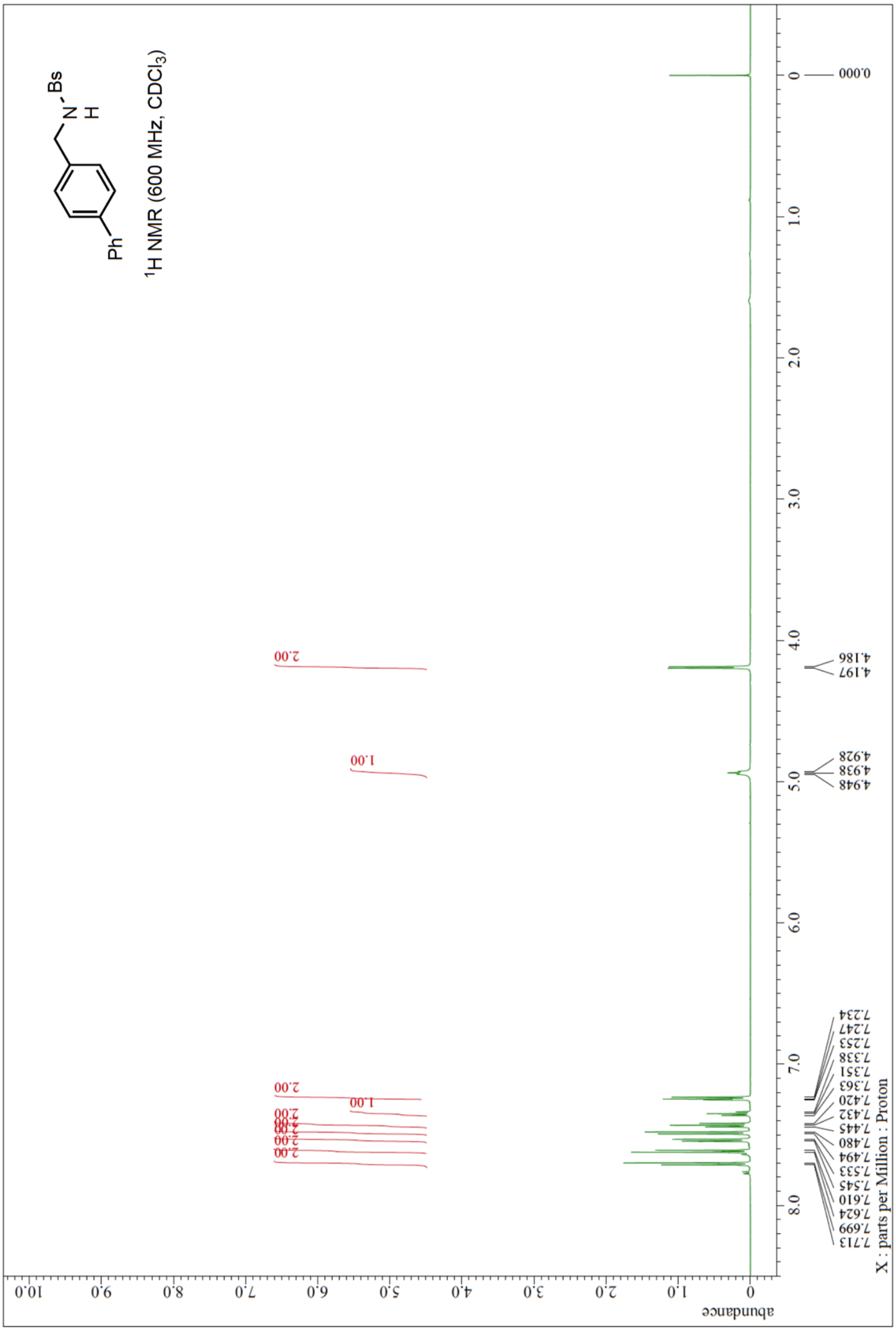

Ph
N
H
Bs
1H NMR (600 MHz, CDCl3)
abundance
X : parts per Million : Proton
0.000
4.186
4.197
4.928
4.938
4.948
7.234
7.247
7.253
7.338
7.351
7.363
7.420
7.432
7.445
7.480
7.494
7.533
7.545
7.610
7.624
7.699
7.713
2.00
1.00

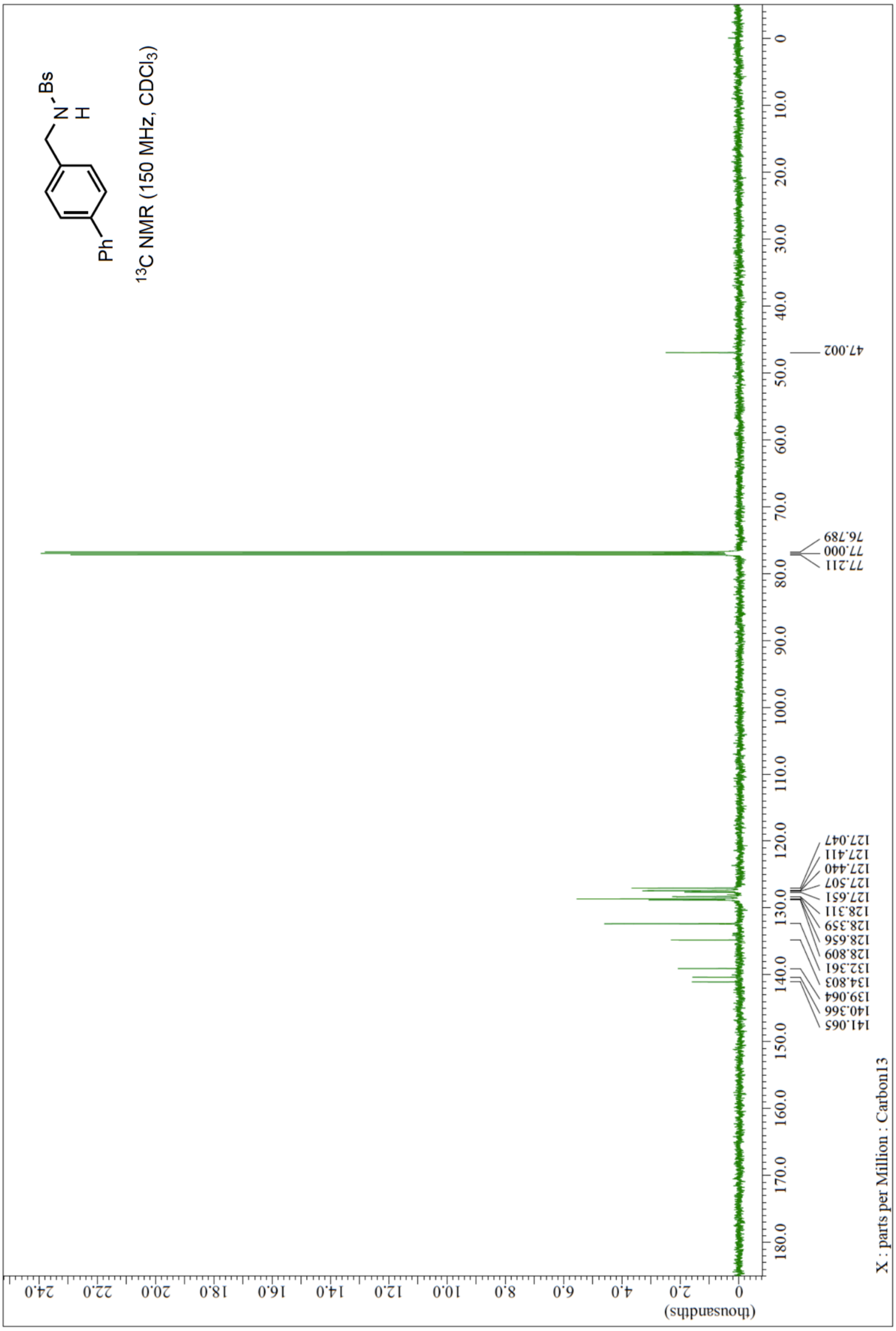
Ph
N
H
Bs
13C NMR (150 MHz, CDCl3)
47.002
77.211
77.000
76.789
127.047
127.411
127.440
127.507
127.651
128.311
128.359
128.656
128.809
132.361
134.803
139.064
140.366
141.065
X : parts per Million : Carbon13
(thousandths)

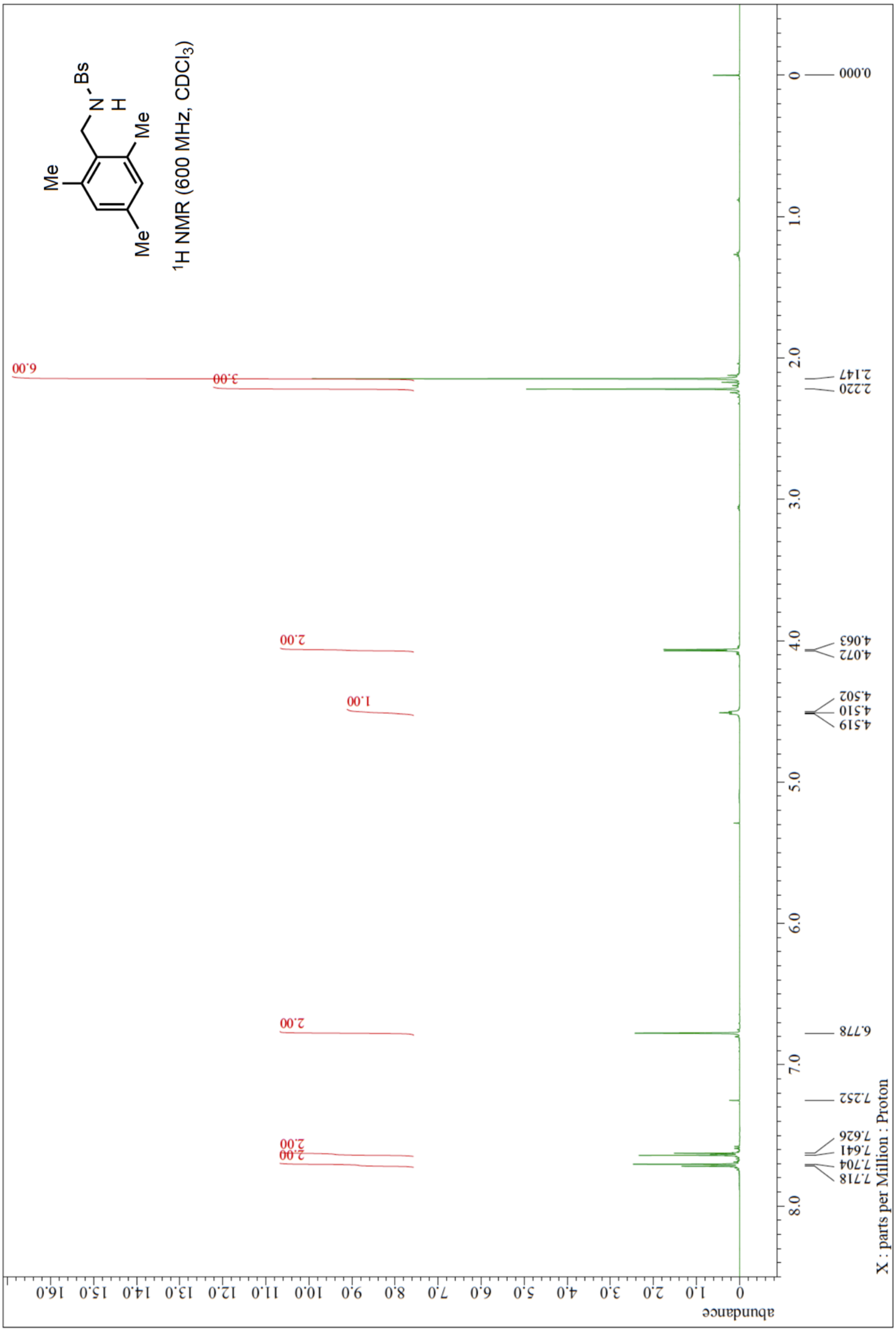

Me
Me
Me
N
H
Bs
^{1}H NMR (600 MHz, CDCl$_3$)
0.000
2.147
2.220
4.063
4.072
4.502
4.510
4.519
6.778
7.252
7.626
7.641
7.704
7.718
6.00
3.00
2.00
1.00
2.00
2.00
2.00
X : parts per Million : Proton
abundance

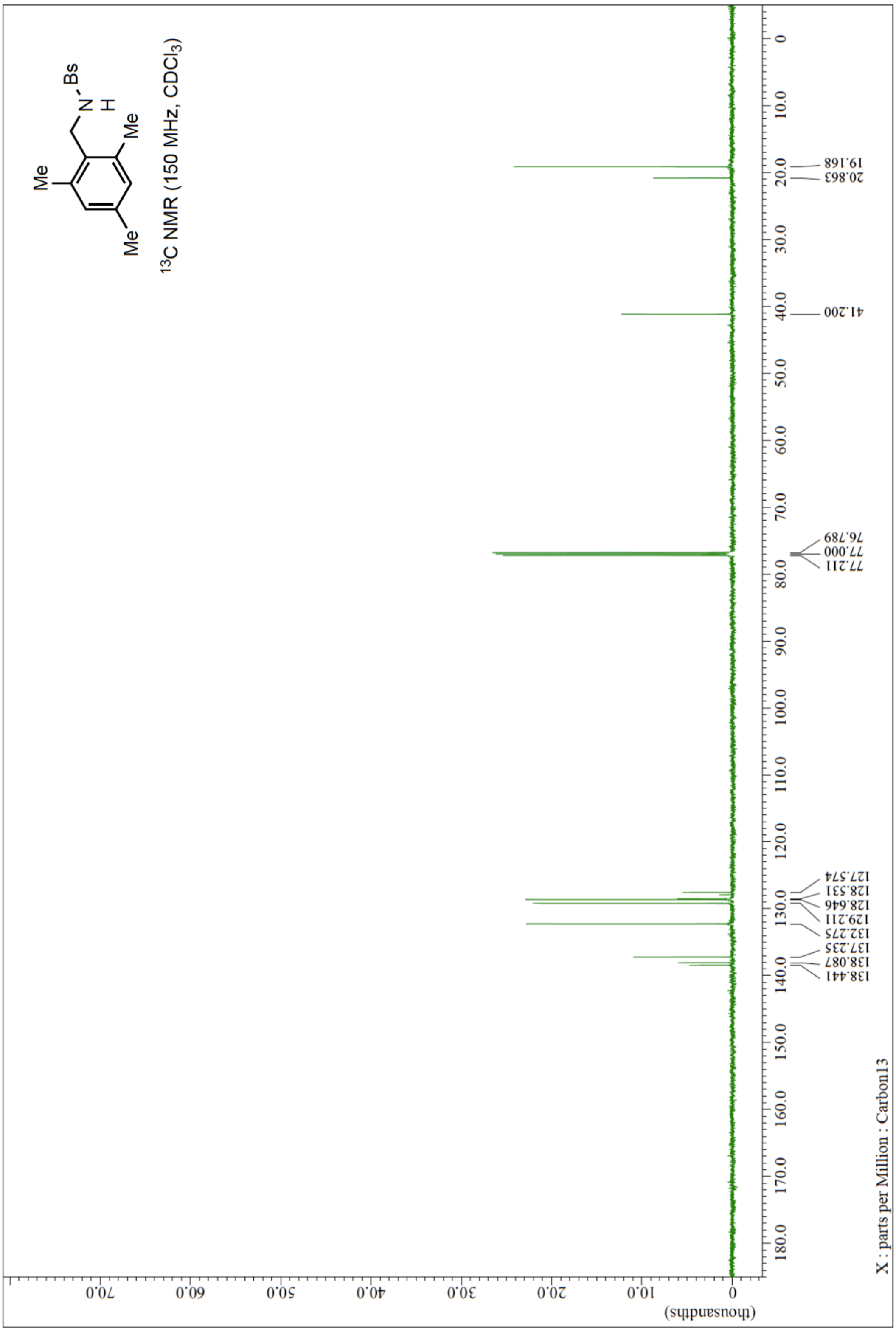
Me
Me
Me
N
H
Bs
^{13}C NMR (150 MHz, CDCl$_3$)
19.168
20.863
41.200
76.789
77.000
77.211
127.574
128.531
128.646
129.211
132.275
137.235
138.087
138.441
X : parts per Million : Carbon13
(thousandths)

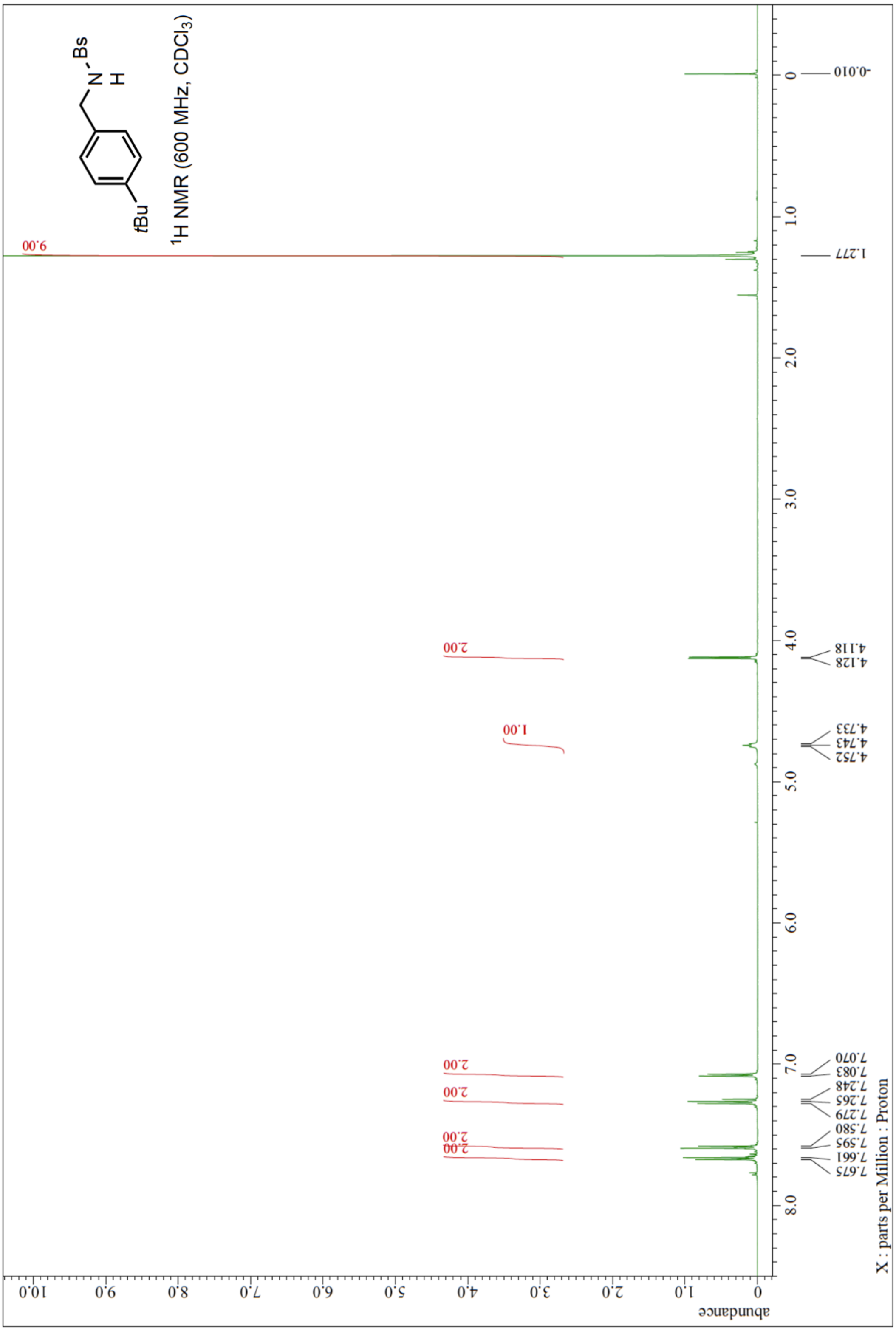
tBu
N
H
Bs
1H NMR (600 MHz, CDCl3)
9.00
2.00
1.00
2.00
2.00
2.00
2.00
-0.010
1.277
4.118
4.128
4.733
4.743
4.752
7.070
7.083
7.248
7.265
7.279
7.580
7.595
7.661
7.675
X : parts per Million : Proton
abundance

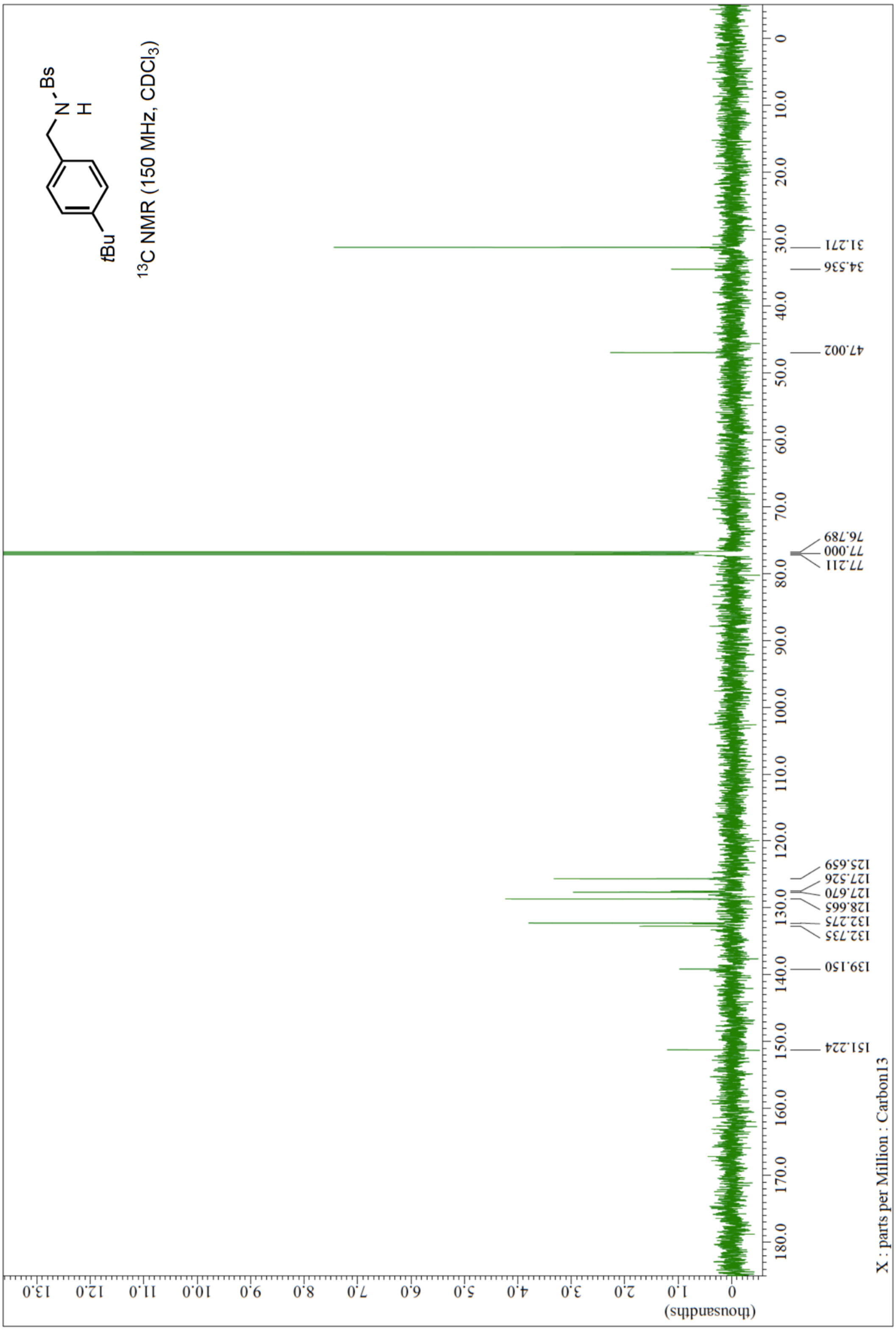

tBu
N
H
Bs
13C NMR (150 MHz, CDCl3)
31.271
34.536
47.002
76.789
77.000
77.211
125.659
127.526
127.670
128.665
132.275
132.735
139.150
151.224
X : parts per Million : Carbon13
(thousandths)

13. Copy of HPLC Spectra

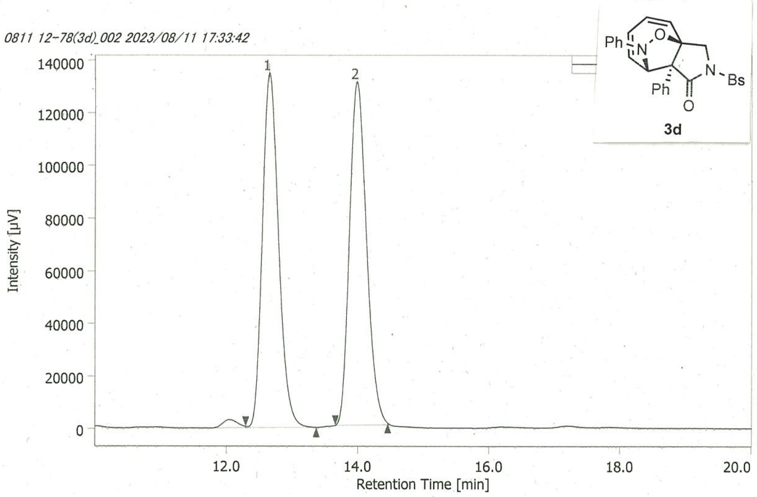

#	tR [min]	Area [μV·sec]	Area%	Height [μV]	Height%
1	12.642	2308166	49.563	134678	50.804
2	13.983	2348905	50.437	130414	49.196

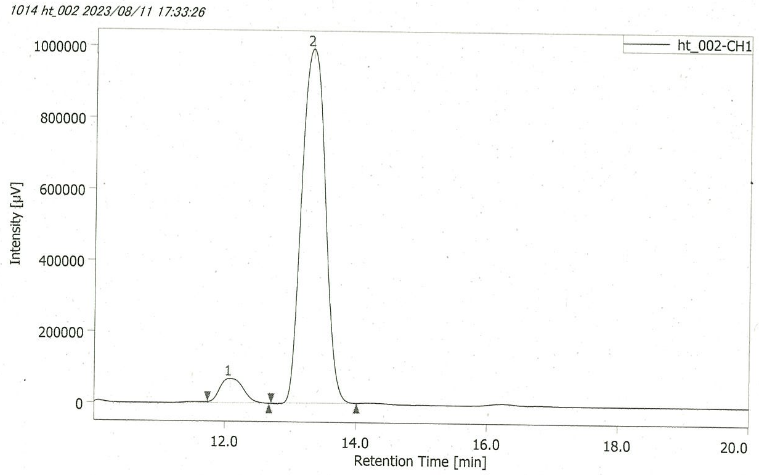

#	tR [min]	Area [μV·sec]	Area%	Height [μV]	Height%
1	12.083	1671897	6.295	68163	6.429
2	13.317	24886144	93.705	992019	93.571

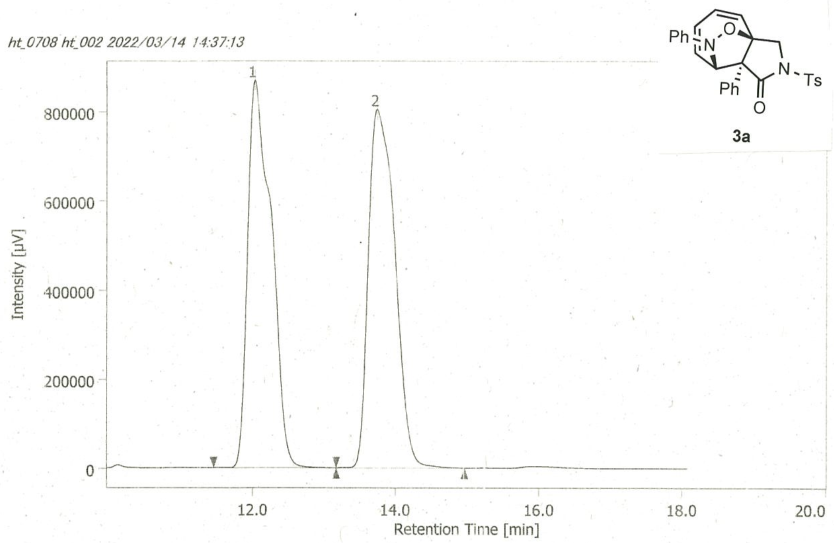

3a

#	tR [min]	Area [μV·sec]	Area%	Height [μV]	Height%
1	12.033	19843997	49.062	868502	51.932
2	13.750	20602641	50.938	803886	48.068

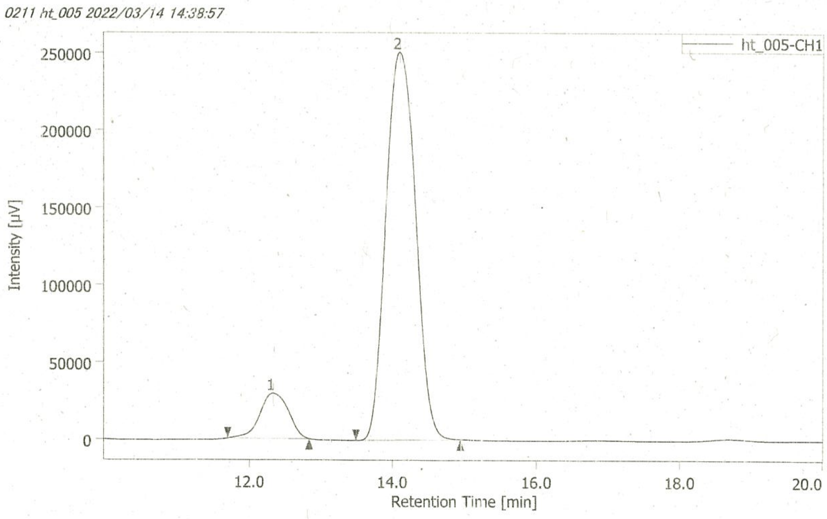

#	tR [min]	Area [μV·sec]	Area%	Height [μV]	Height%
1	12.333	824164	10.313	29429	10.502
2	14.100	7167238	89.687	250792	89.498

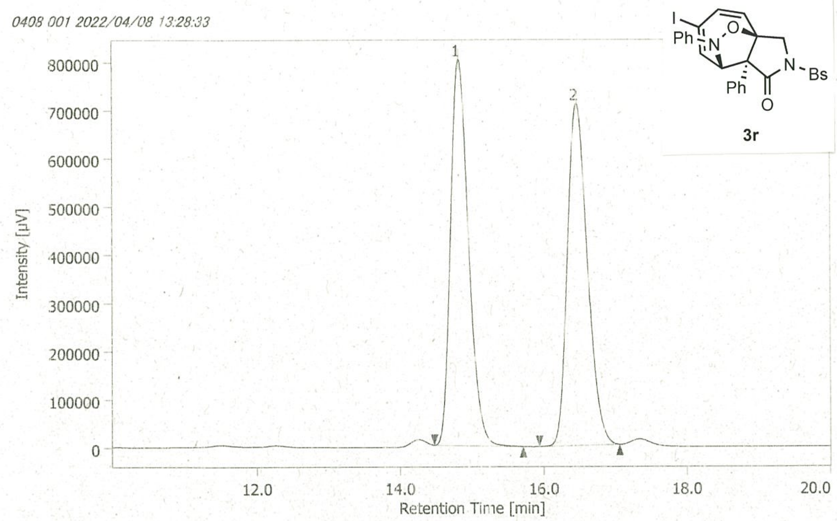

3r

#	tR [min]	Area [µV·sec]	Area%	Height [µV]	Height%
1	14.825	13824111	50.044	801831	53.034
2	16.467	13799693	49.956	710074	46.966

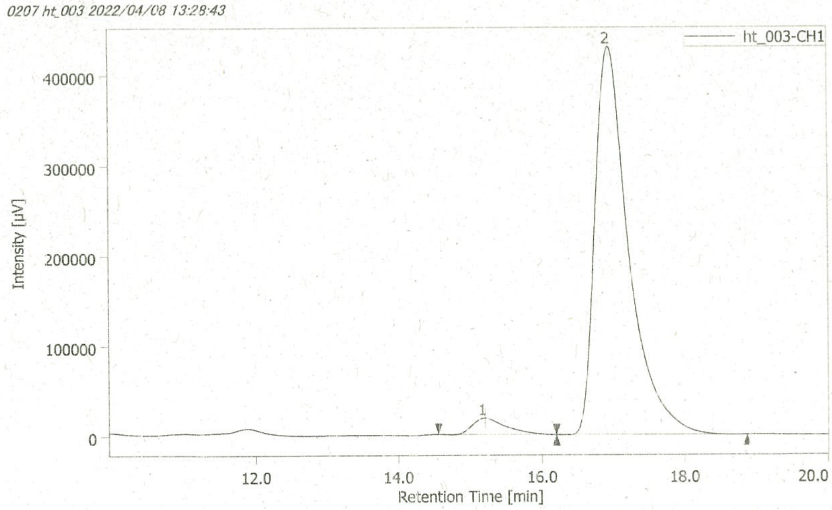

#	tR [min]	Area [µV·sec]	Area%	Height [µV]	Height%
1	15.200	560771	3.826	17974	4.018
2	16.950	14094263	96.174	429391	95.982

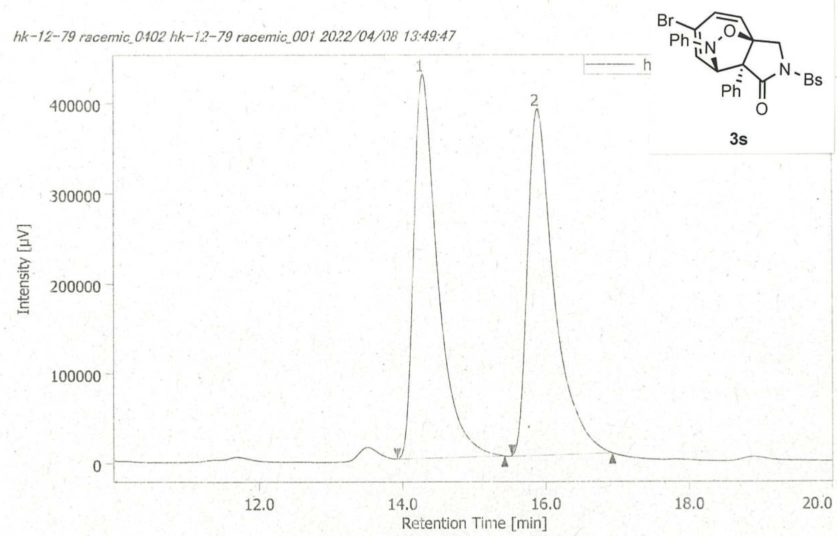

#	tR [min]	Area [μV·sec]	Area%	Height [μV]	Height%
1	14.292	9947646	49.869	425974	52.546
2	15.892	9999850	50.131	384701	47.454

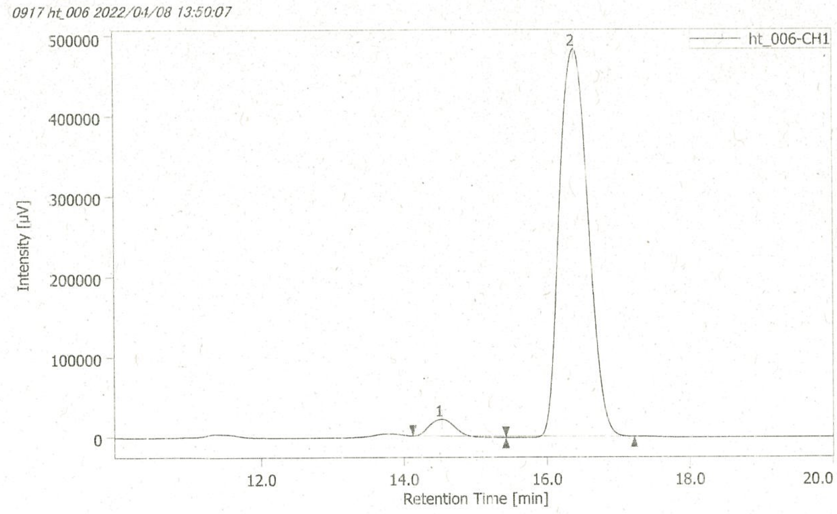

#	tR [min]	Area [μV·sec]	Area%	Height [μV]	Height%
1	14.533	499121	3.590	20890	4.154
2	16.392	13402199	96.410	481970	95.846

1014 ht_005 2022/03/14 14:49:41

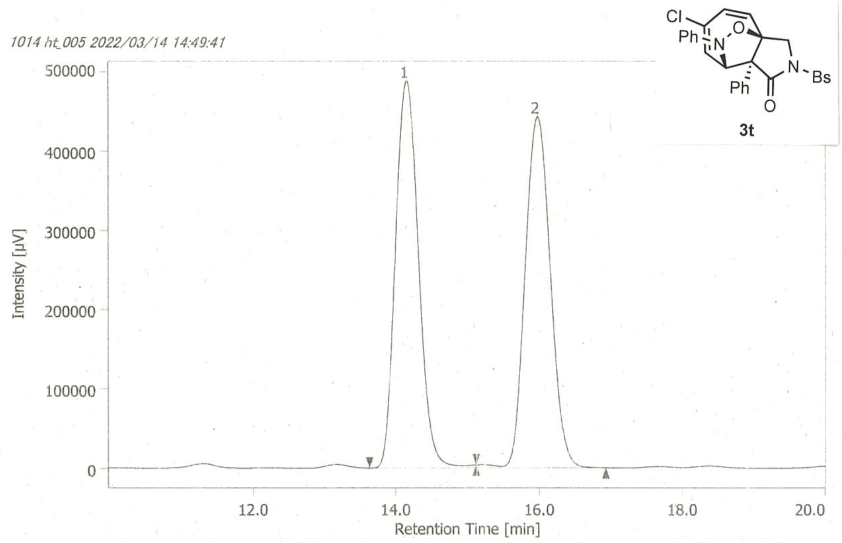

#	tR [min]	Area [μV·sec]	Area%	Height [μV]	Height%
1	14.158	10890266	50.090	487880	52.441
2	15.975	10851170	49.910	442455	47.559

1014 ht_003 2022/03/14 14:50:01

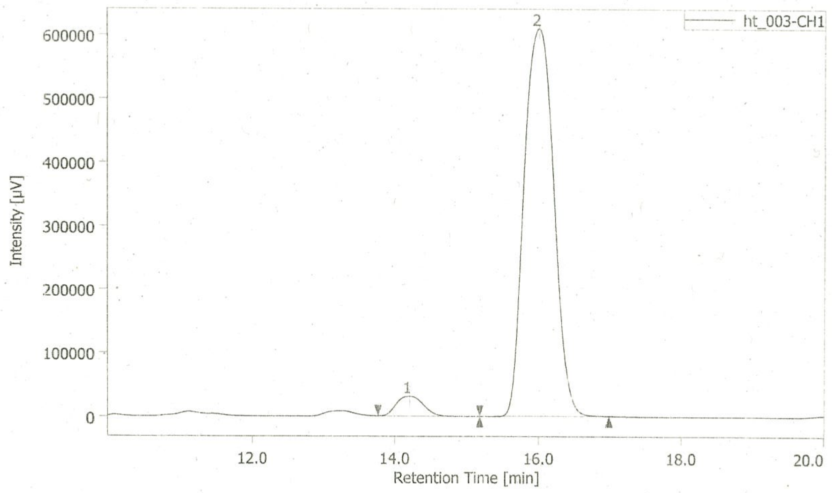

#	tR [min]	Area [μV·sec]	Area%	Height [μV]	Height%
1	14.208	784524	4.280	30418	4.760
2	16.000	17545355	95.720	608556	95.240

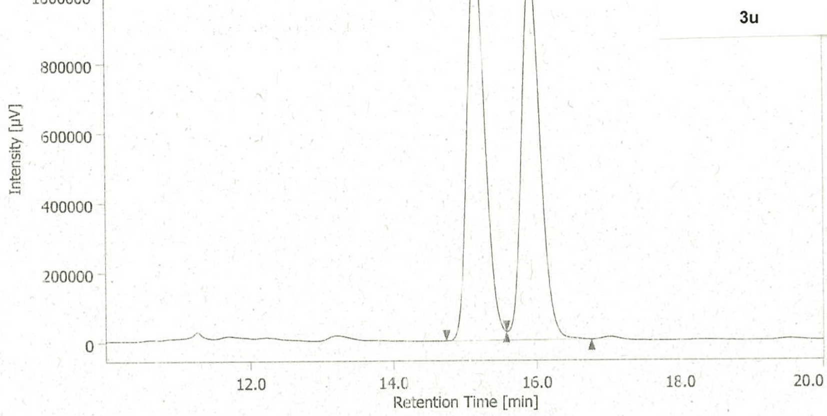

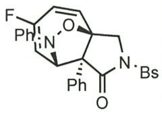

3u

#	tR [min]	Area [µV·sec]	Area%	Height [µV]	Height%
1	15.158	19301896	50.093	1111978	51.133
2	15.917	19230428	49.907	1062713	48.867

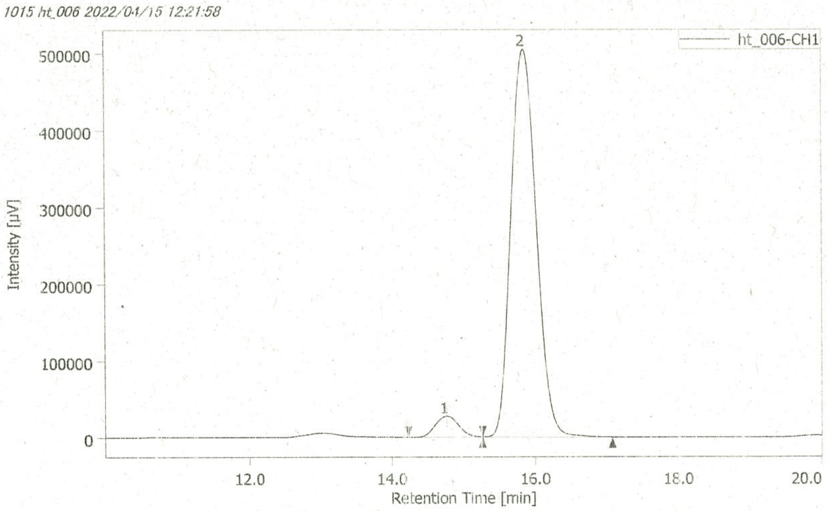

#	tR [min]	Area [µV·sec]	Area%	Height [µV]	Height%
1	14.758	617137	4.702	27386	5.146
2	15.842	12507891	95.298	504846	94.854

0207 ht_001 2022/03/14 14:56:38

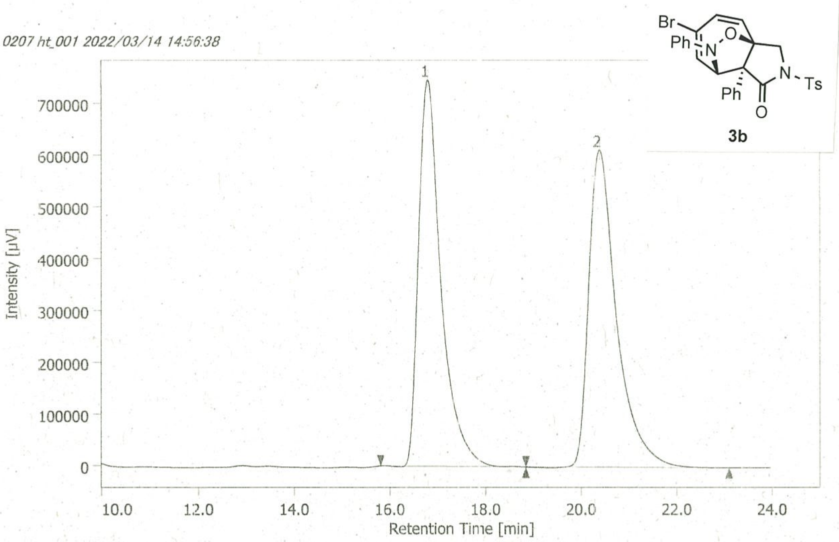

#	tR [min]	Area [μV·sec]	Area%	Height [μV]	Height%
1	16.783	24364673	49.713	746867	54.945
2	20.383	24646296	50.287	612432	45.055

1015 ht_011 2022/03/14 14:57:15

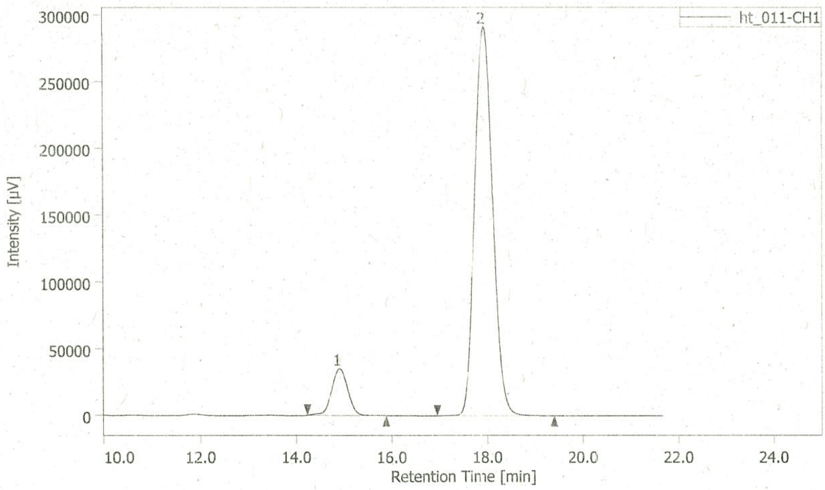

#	tR [min]	Area [μV·sec]	Area%	Height [μV]	Height%
1	14.917	818507	9.696	35217	10.783
2	17.925	7623087	90.304	291369	89.217

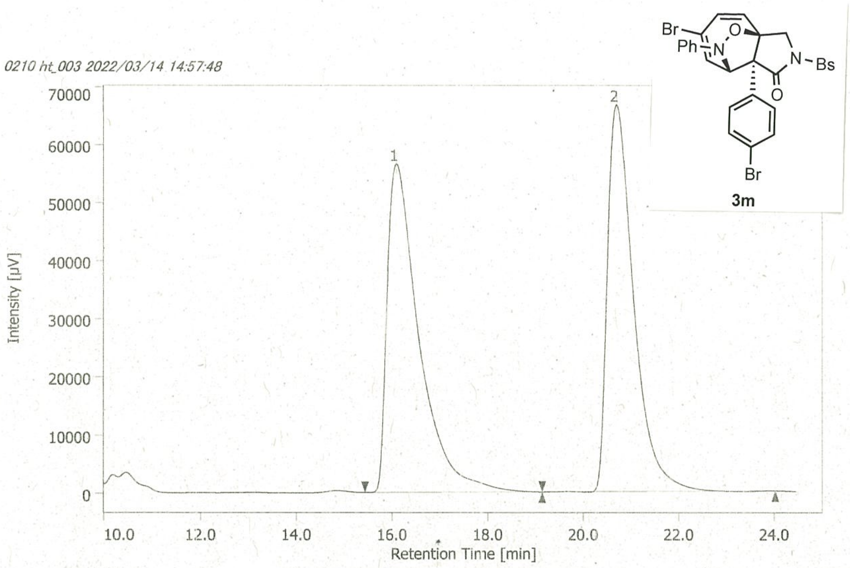

3m

#	tR [min]	Area [µV·sec]	Area%	Height [µV]	Height%
1	16.092	2605017	50.537	56532	45.921
2	20.700	2549675	49.463	66574	54.079

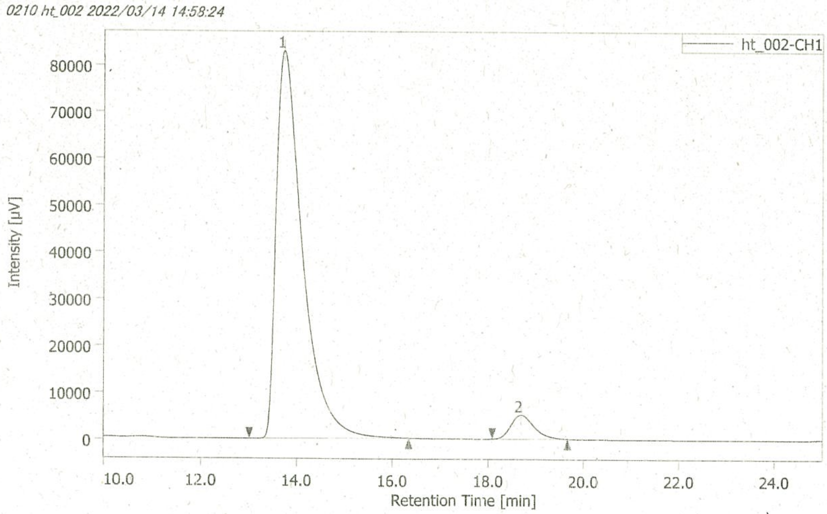

#	tR [min]	Area [µV·sec]	Area%	Height [µV]	Height%
1	13.733	3176826	94.822	82830	94.180
2	18.692	173486	5.178	5119	5.820

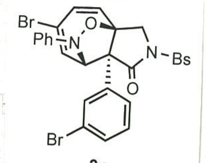

3n

1015 ht_001 2022/03/14 16:59:55

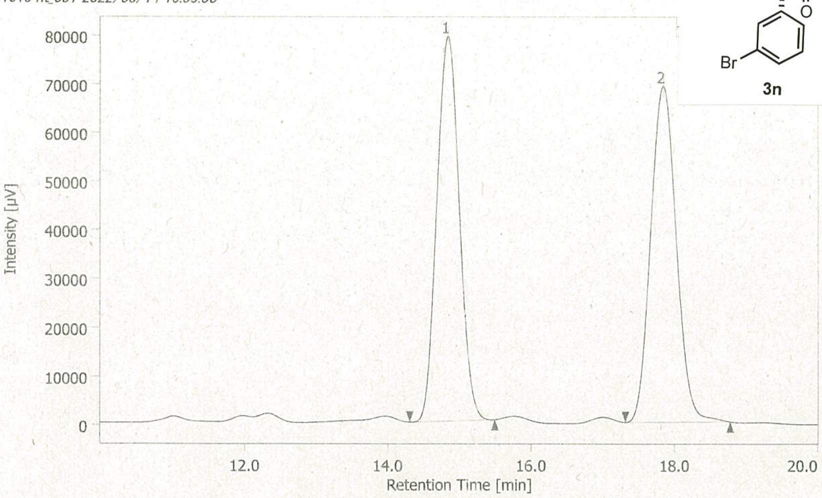

#	tR [min]	Area [µV·sec]	Area%	Height [µV]	Height%
1	14.842	1717403	50.060	79026	53.378
2	17.842	1713300	49.940	69024	46.622

1015 ht_008 2022/03/14 17:00:25

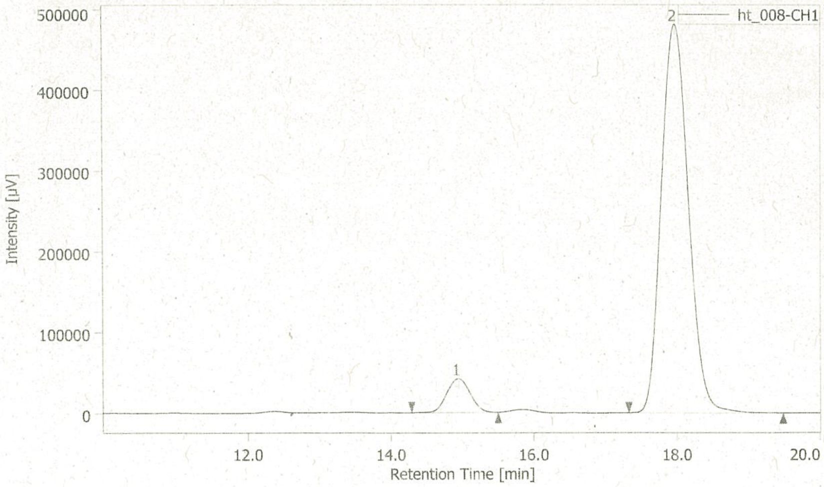

#	tR [min]	Area [µV·sec]	Area%	Height [µV]	Height%
1	14.950	948861	6.960	42042	8.042
2	17.967	12683960	93.040	480725	91.958

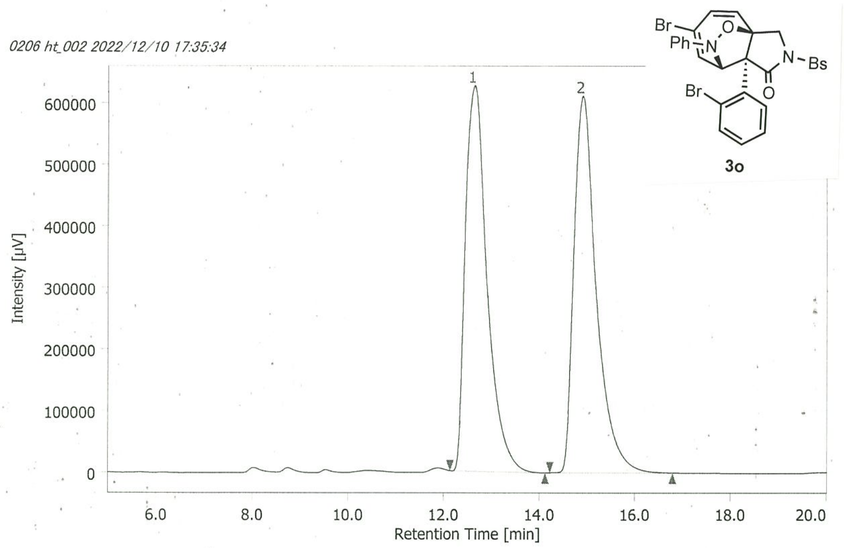

#	tR [min]	Area [µV·sec]	Area%	Height [µV]	Height%
1	12.650	19680233	49.692	626267	50.605
2	14.908	19924112	50.308	611304	49.395

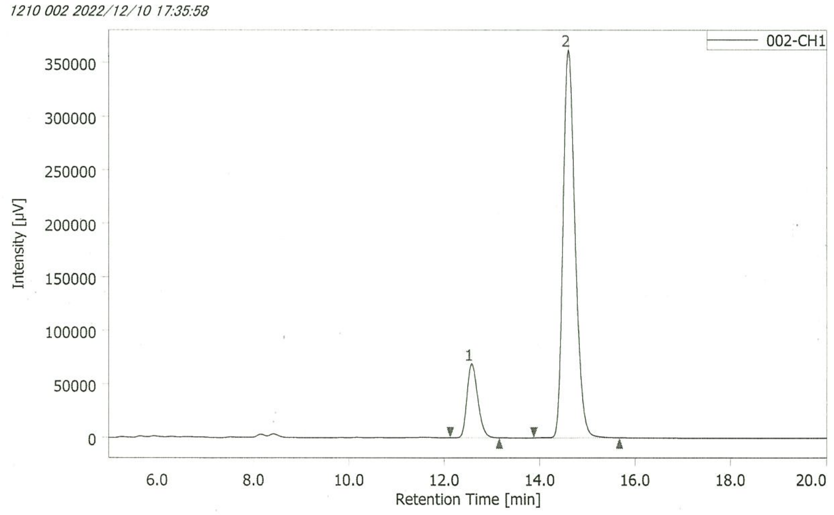

#	tR [min]	Area [µV·sec]	Area%	Height [µV]	Height%
1	12.567	1081672	14.564	69188	16.036
2	14.592	6345524	85.436	362258	83.964

1014 ht_004 2022/03/14 15:01:53

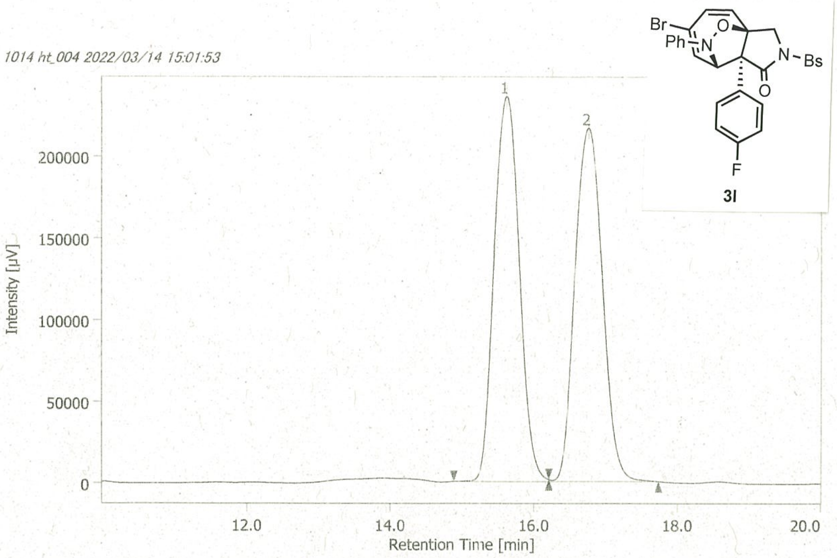

#	tR [min]	Area [µV·sec]	Area%	Height [µV]	Height%
1	15.617	5763165	49.995	235428	52.137
2	16.767	5764384	50.005	216131	47.863

1015 ht_010 2022/03/14 15:02:16

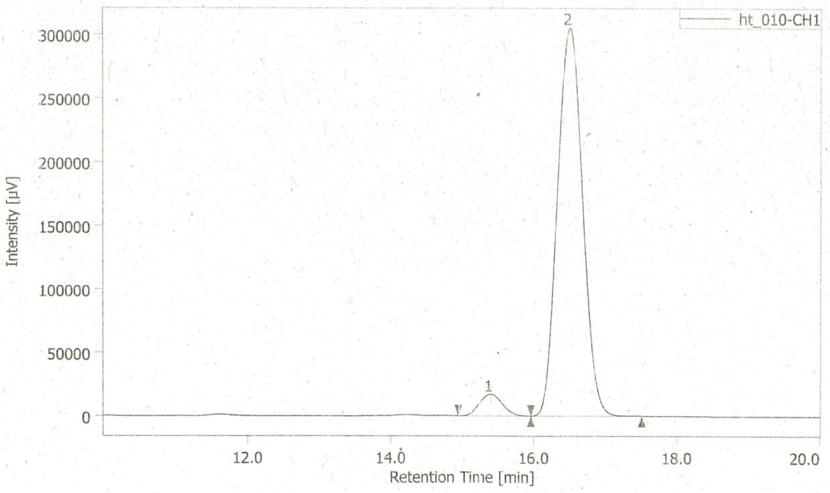

#	tR [min]	Area [µV·sec]	Area%	Height [µV]	Height%
1	15.400	373847	4.778	16836	5.228
2	16.500	7449769	95.222	305186	94.772

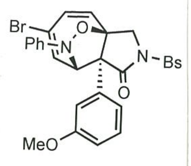

3k

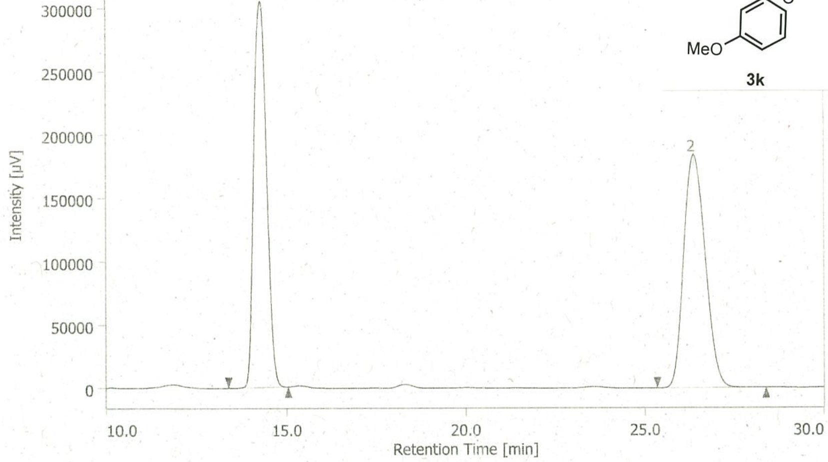

#	tR [min]	Area [μV·sec]	Area%	Height [μV]	Height%
1	14.283	7631898	49.817	305236	62.407
2	26.358	7688021	50.183	183871	37.593

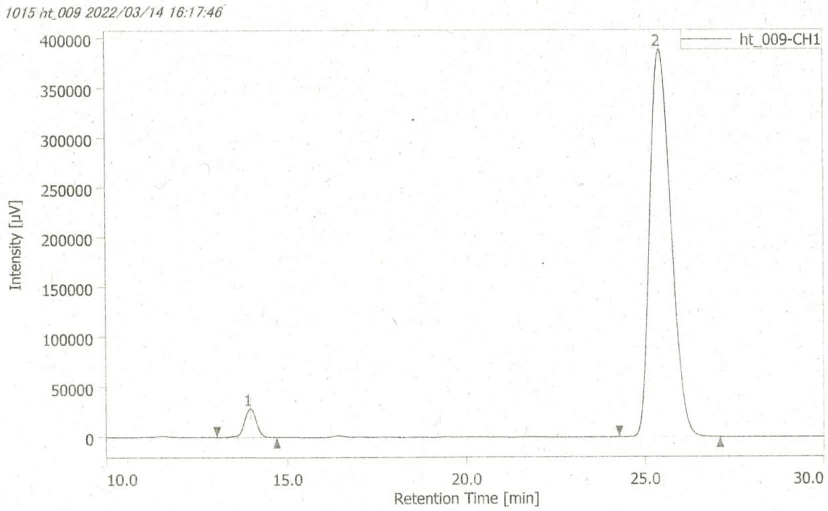

#	tR [min]	Area [μV·sec]	Area%	Height [μV]	Height%
1	13.967	666398	4.033	28723	6.882
2	25.408	15857694	95.967	388651	93.118

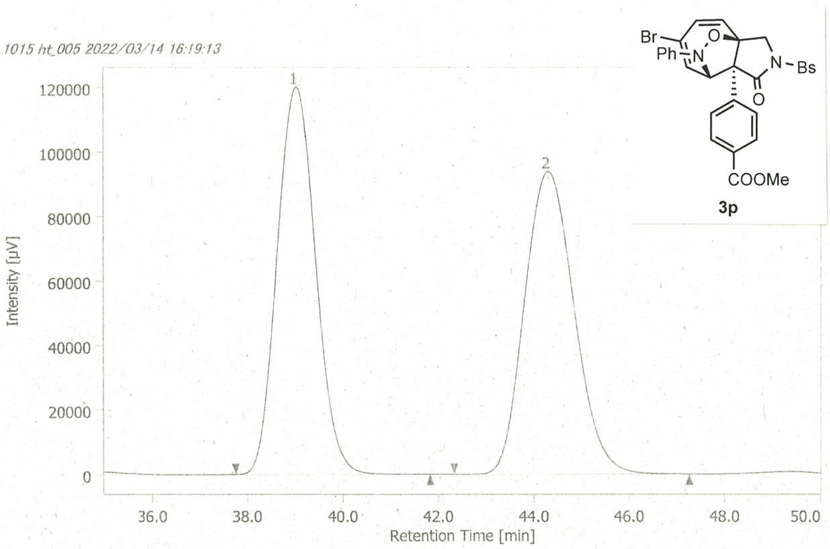

#	tR [min]	Area [μV·sec]	Area%	Height [μV]	Height%
1	39.033	6694174	49.803	120030	56.151
2	44.300	6747093	50.197	93733	43.849

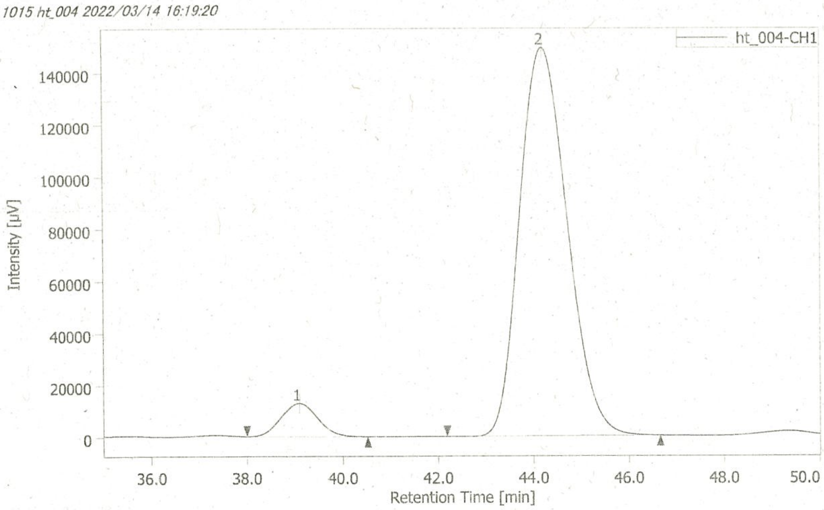

#	tR [min]	Area [μV·sec]	Area%	Height [μV]	Height%
1	39.100	678165	6.163	12867	7.942
2	44.192	10325296	93.837	149135	92.058

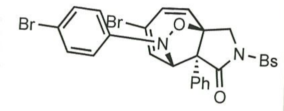

3f

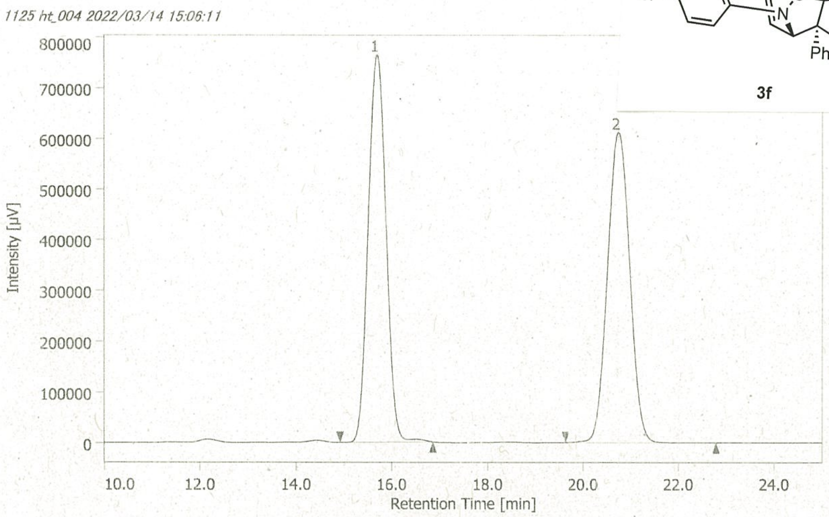

#	tR [min]	Area [µV·sec]	Area%	Height [µV]	Height%
1	15.692	19854452	50.024	763888	55.542
2	20.733	19835507	49.976	611438	44.458

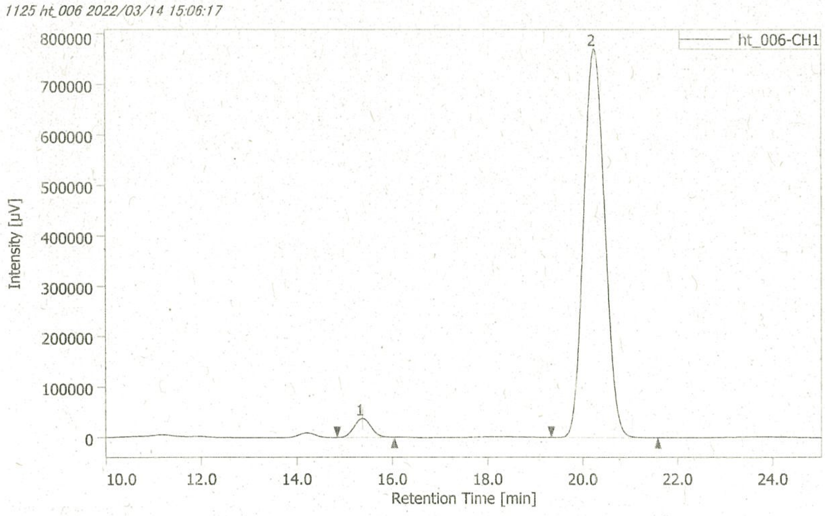

#	tR [min]	Area [µV·sec]	Area%	Height [µV]	Height%
1	15.367	927852	3.748	37269	4.616
2	20.233	23830999	96.252	770084	95.384

1125 ht_002 2022/03/14 15:09:53

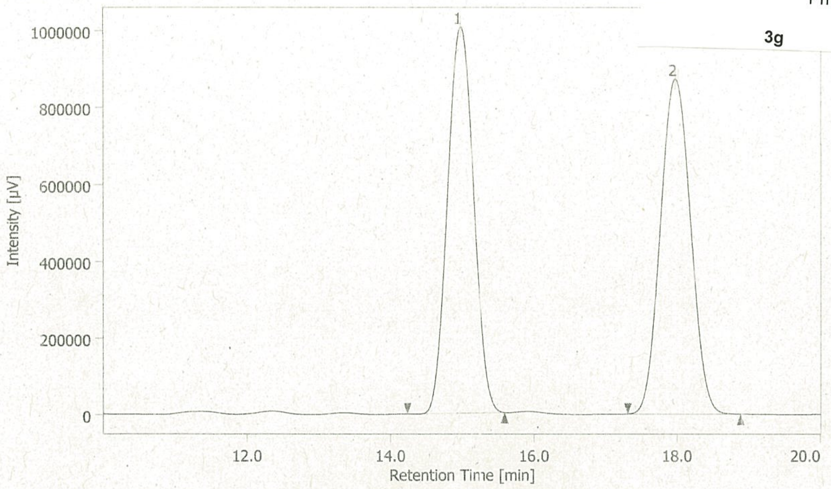

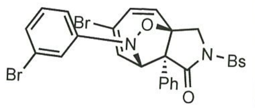

3g

#	tR [min]	Area [µV·sec]	Area%	Height [µV]	Height%
1	14.967	25299933	49.759	1005621	53.547
2	17.967	25545119	50.241	872394	46.453

1125 ht_007 2022/03/14 15:10:10

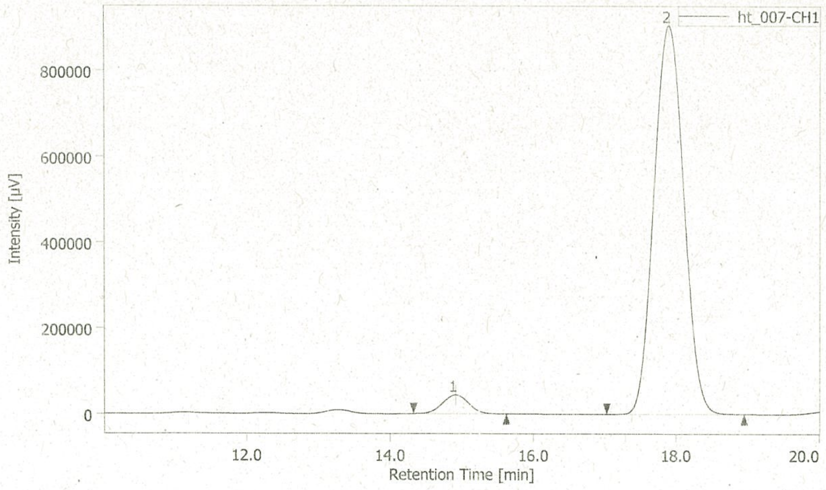

#	tR [min]	Area [µV·sec]	Area%	Height [µV]	Height%
1	14.908	1102611	4.042	43771	4.613
2	17.892	26175532	95.958	905098	95.387

1125 ht_001 2022/03/14 15:10:39

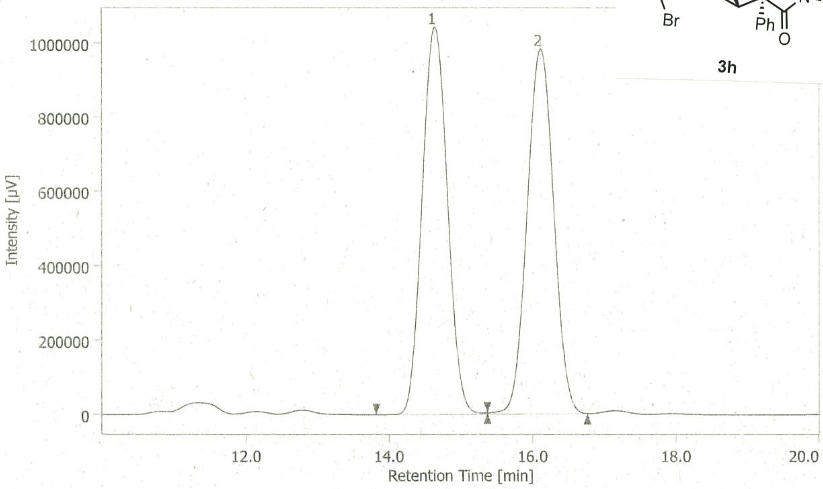

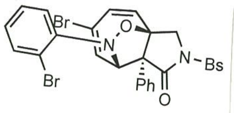

3h

#	tR [min]	Area [µV·sec]	Area%	Height [µV]	Height%
1	14.633	25448974	50.081	1042836	51.505
2	16.108	25366291	49.919	981874	48.495

1125 ht_008 2022/03/14 15:10:58

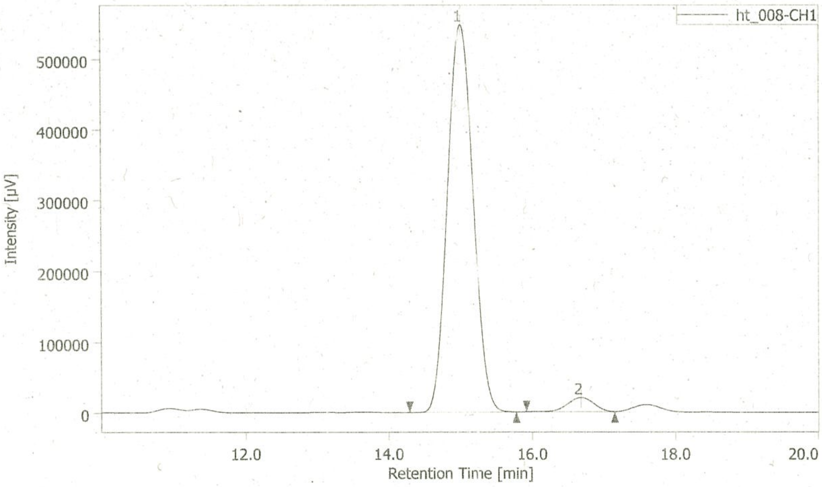

#	tR [min]	Area [µV·sec]	Area%	Height [µV]	Height%
1	15.000	13566839	96.415	547677	96.516
2	16.675	504486	3.585	19773	3.484

1125 ht_005 2022/03/14 15:13:38

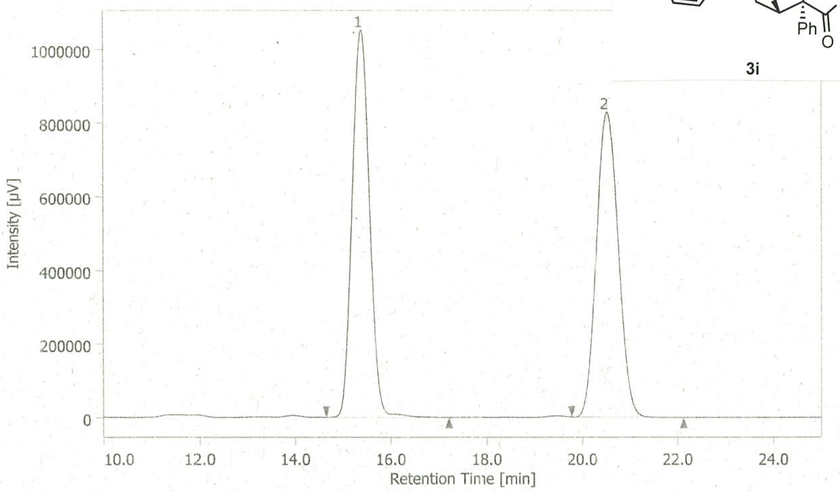

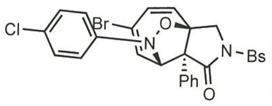

3i

#	tR [min]	Area [μV·sec]	Area%	Height [μV]	Height%
1	15.375	26579383	50.208	1051158	55.946
2	20.508	26359056	49.792	827727	44.054

1125 ht_009 2022/03/14 15:13:22

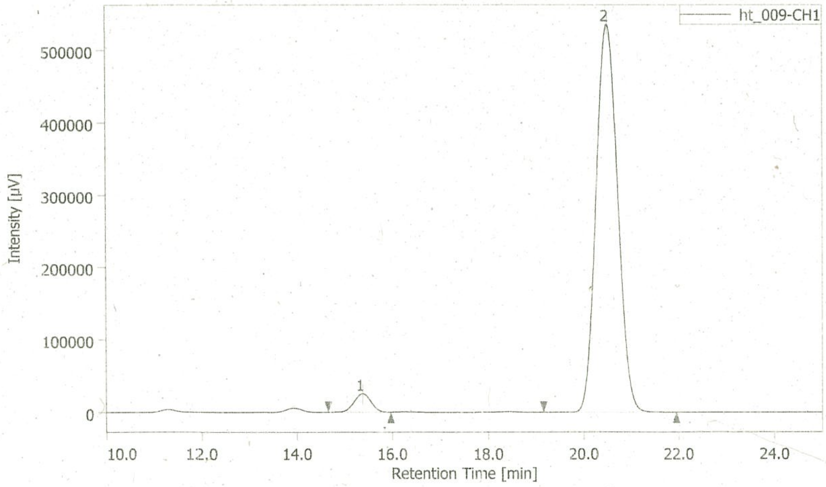

#	tR [min]	Area [μV·sec]	Area%	Height [μV]	Height%
1	15.375	629567	3.585	25349	4.520
2	20.492	16933785	96.415	535434	95.480

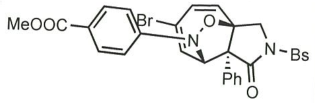

3j

1125 ht_003 2022/03/14 15:14:54

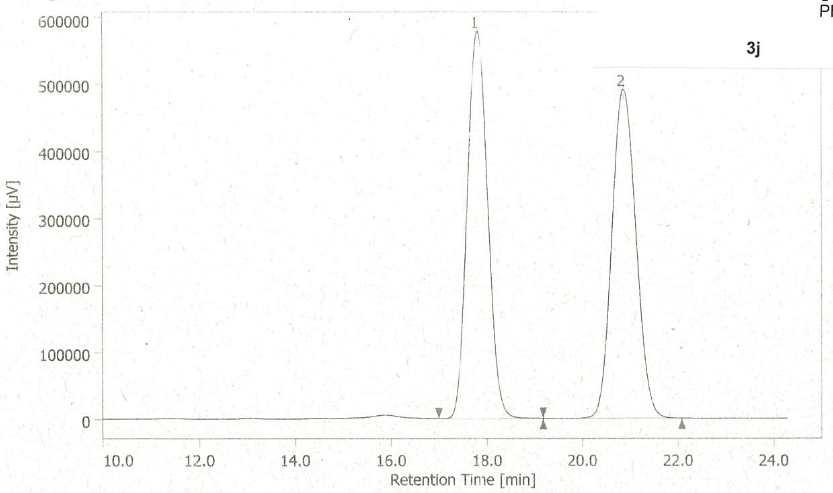

#	tR [min]	Area [µV·sec]	Area%	Height [µV]	Height%
1	17.792	17212645	49.942	577106	54.073
2	20.858	17252801	50.058	490164	45.927

0207 ht_005 2022/03/14 15:15:07

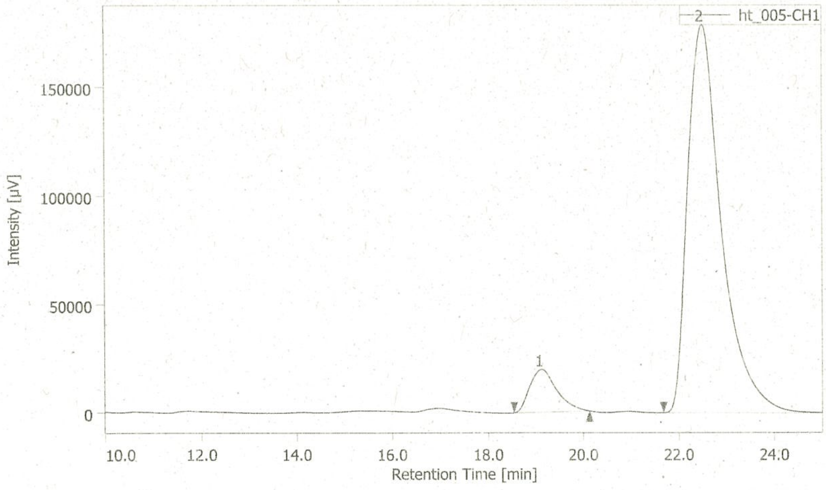

#	tR [min]	Area [µV·sec]	Area%	Height [µV]	Height%
1	19.125	780430	8.128	19813	9.973
2	22.492	8820765	91.872	178849	90.027

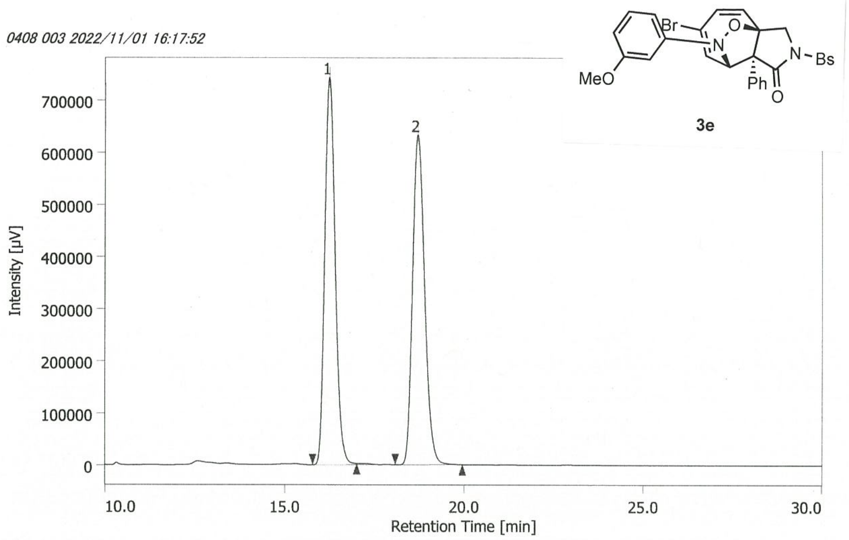

#	tR [min]	Area [µV·sec]	Area%	Height [µV]	Height%
1	16.242	14913844	50.042	742469	53.997
2	18.708	14888580	49.958	632547	46.003

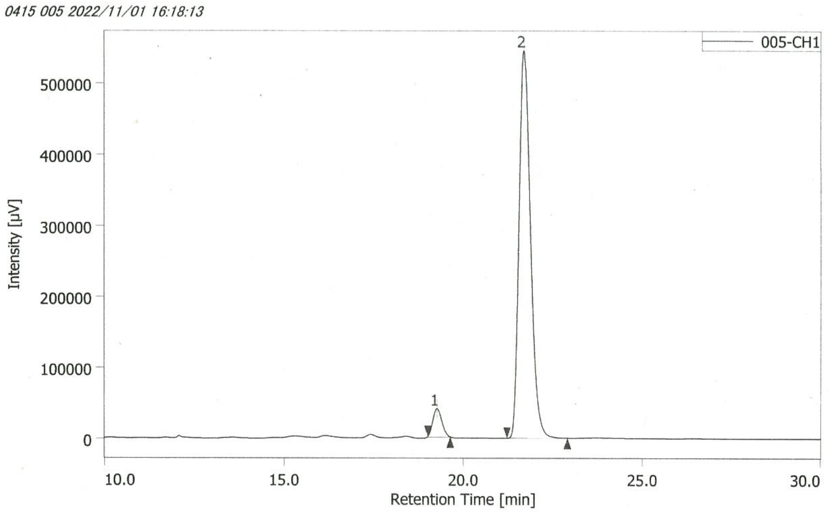

#	tR [min]	Area [µV·sec]	Area%	Height [µV]	Height%
1	19.275	697364	5.492	40182	6.866
2	21.700	11999619	94.508	545049	93.134

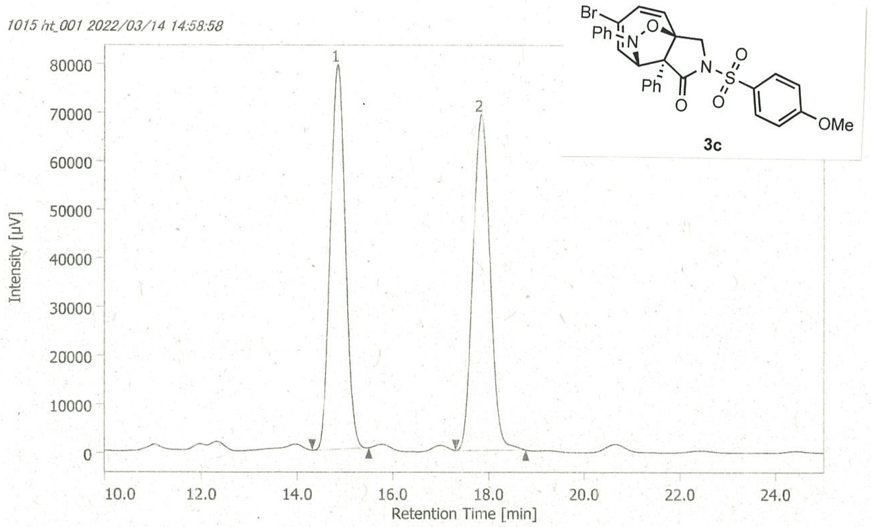

#	tR [min]	Area [µV·sec]	Area%	Height [µV]	Height%
1	14.842	1717403	50.060	79026	53.378
2	17.842	1713300	49.940	69024	46.622

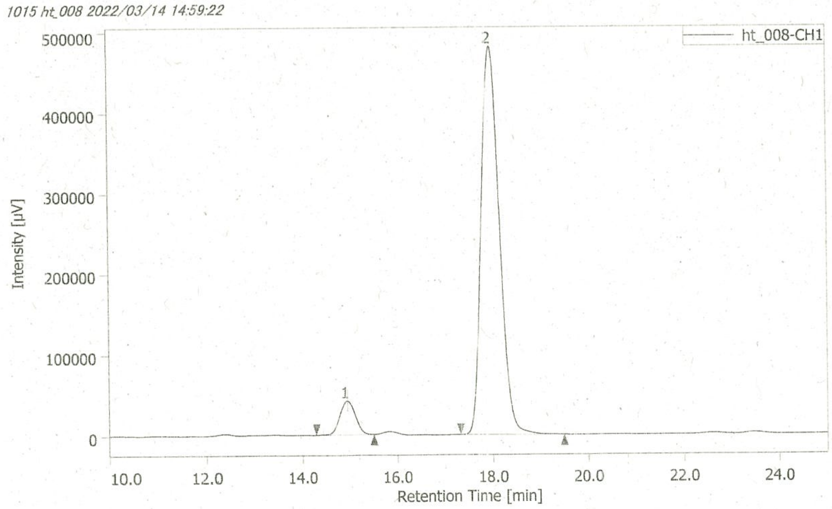

#	tR [min]	Area [µV·sec]	Area%	Height [µV]	Height%
1	14.950	948861	6.960	42042	8.042
2	17.967	12683960	93.040	480725	91.958

1101 005 2022/11/01 15:04:48

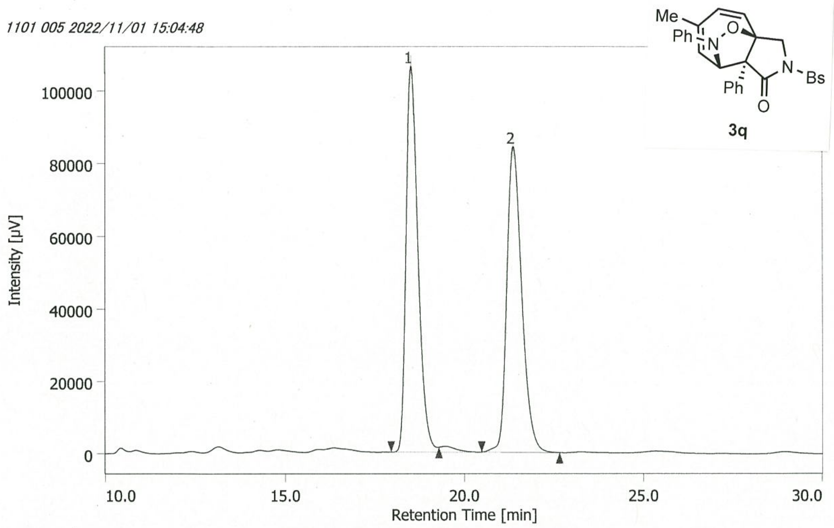

3q

#	tR [min]	Area [µV·sec]	Area%	Height [µV]	Height%
1	18.533	2465109	50.130	106348	55.794
2	21.367	2452283	49.870	84261	44.206

1101 006 2022/11/01 15:10:26

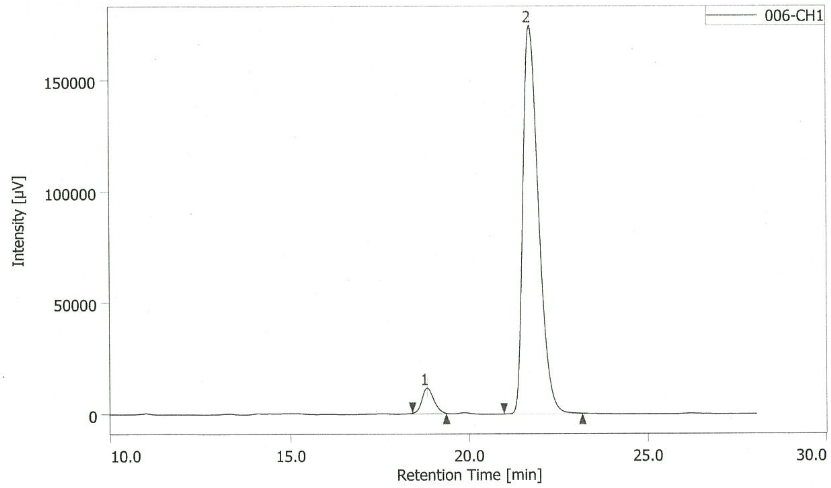

#	tR [min]	Area [µV·sec]	Area%	Height [µV]	Height%
1	18.833	272541	4.923	11674	6.272
2	21.717	5263115	95.077	174461	93.728

1215 xBsm-OTBS racemi_002 2024/01/06 17:45:10

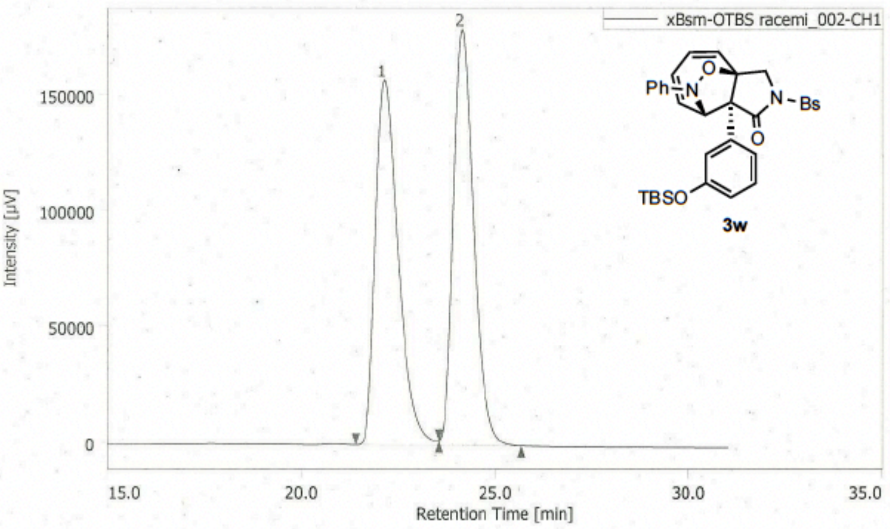

#	tR [min]	Area [μV·sec]	Area%	Height [μV]	Height%
1	22.142	6458975	49.704	157380	46.696
2	24.150	6536008	50.296	179653	53.304

1221 hk-11-847_002 2024/01/06 17:44:58

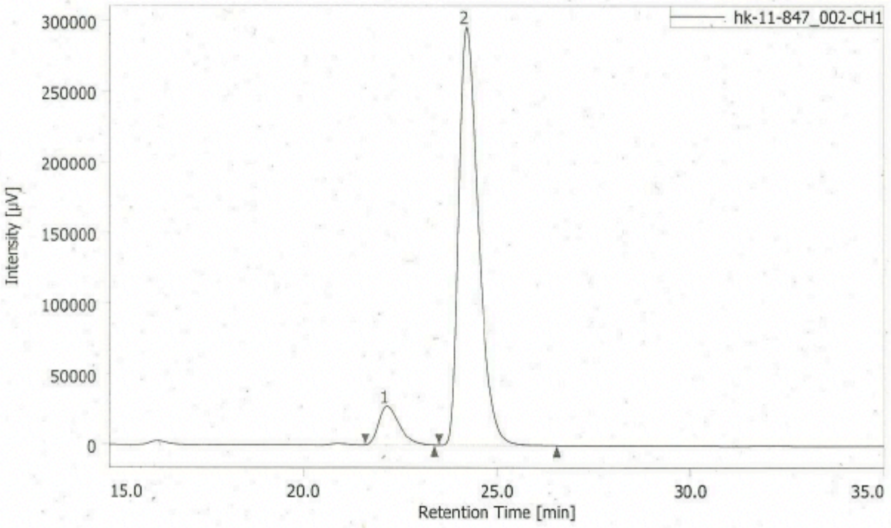

#	tR [min]	Area [μV·sec]	Area%	Height [μV]	Height%
1	22.167	1026818	8.965	27633	8.544
2	24.217	10427101	91.035	295801	91.456

1217 x Bs p-CN PhNO racemi_001 2024/01/06 17:41:58

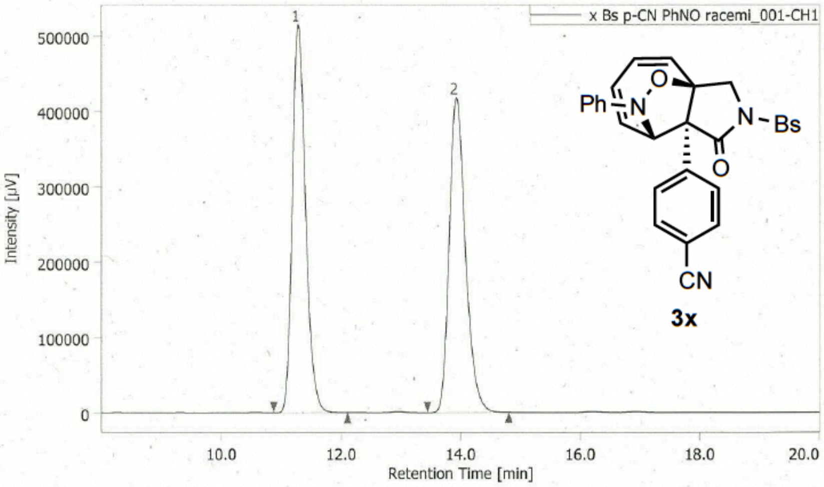

#	tR [min]	Area [µV·sec]	Area%	Height [µV]	Height%
1	11.283	7868331	50.003	514653	55.194
2	13.925	7867462	49.997	417784	44.806

1221 hk-11-848_001 2024/01/06 17:41:32

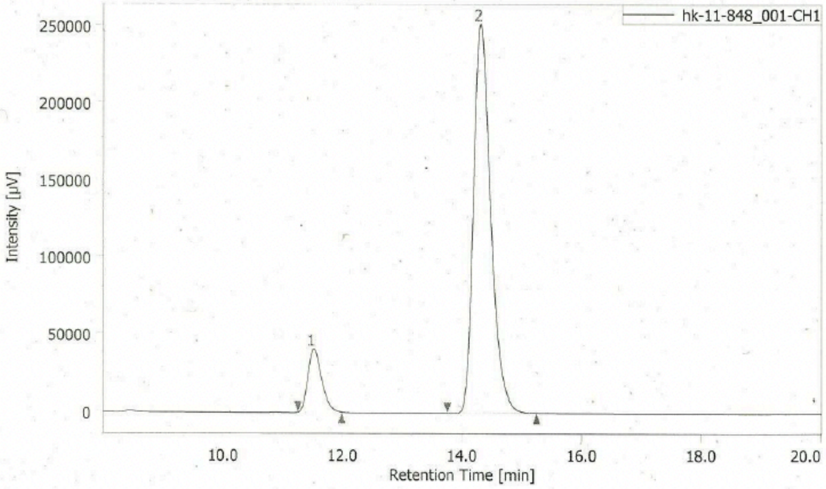

#	tR [min]	Area [µV·sec]	Area%	Height [µV]	Height%
1	11.525	650093	11.463	40660	13.893
2	14.317	5021278	88.537	252017	86.107

4 Ph PhNO racamate_1228 IBN3 HD 7030_004 2023/12/28 15:31:38

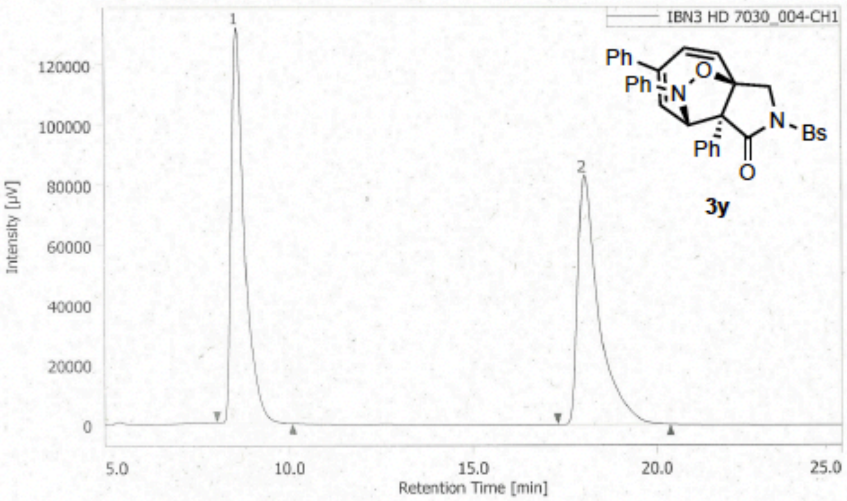

#	tR [min]	Area [µV·sec]	Area%	Height [µV]	Height%
1	8.600	3303086	49.993	131855	61.346
2	18.033	3303982	50.007	83082	38.654

4 Ph PhNO asymmetric_1228 IBN3 HD 7030_005 2023/12/28 16:15:30

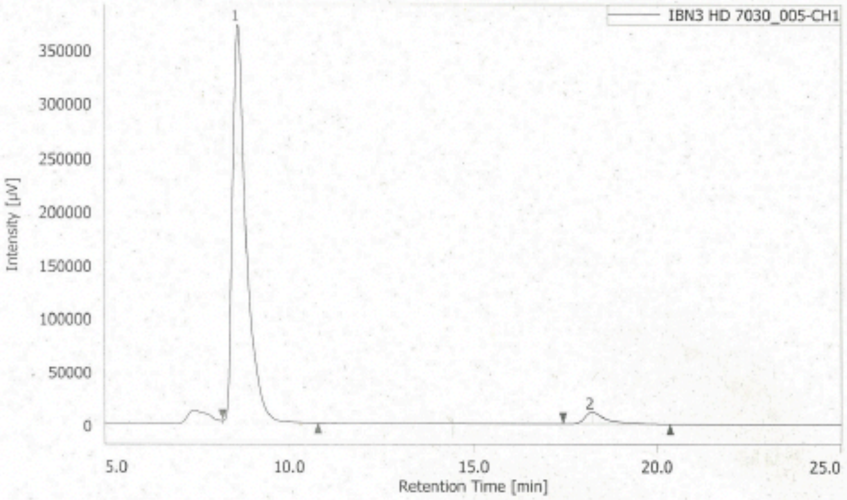

#	tR [min]	Area [µV·sec]	Area%	Height [µV]	Height%
1	8.633	9753854	95.699	372616	97.158
2	18.233	438375	4.301	10900	2.842

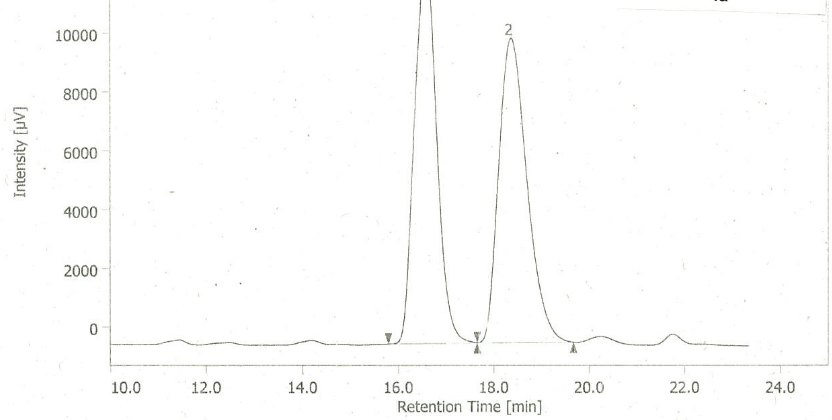

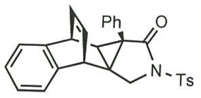

4a

#	tR [min]	Area [µV·sec]	Area%	Height [µV]	Height%
1	16.575	426467	49.955	13122	55.878
2	18.367	427232	50.045	10361	44.122

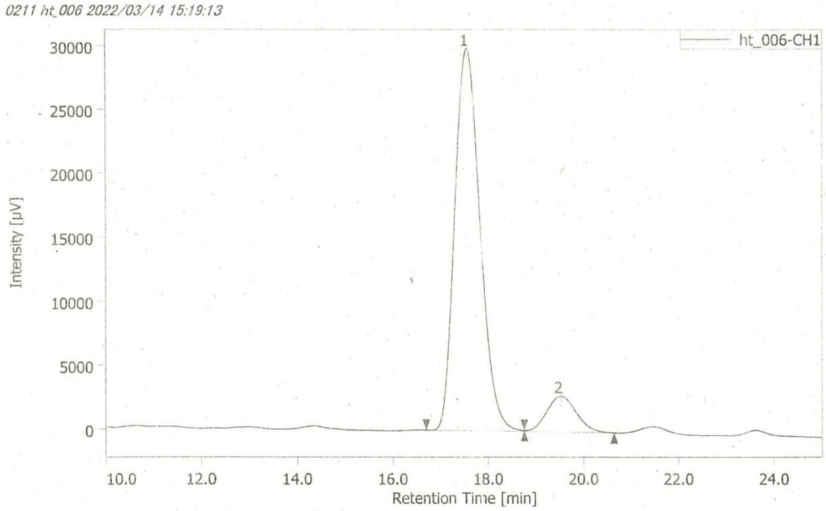

#	tR [min]	Area [µV·sec]	Area%	Height [µV]	Height%
1	17.550	1098539	90.140	29890	91.388
2	19.525	120166	9.860	2817	8.612

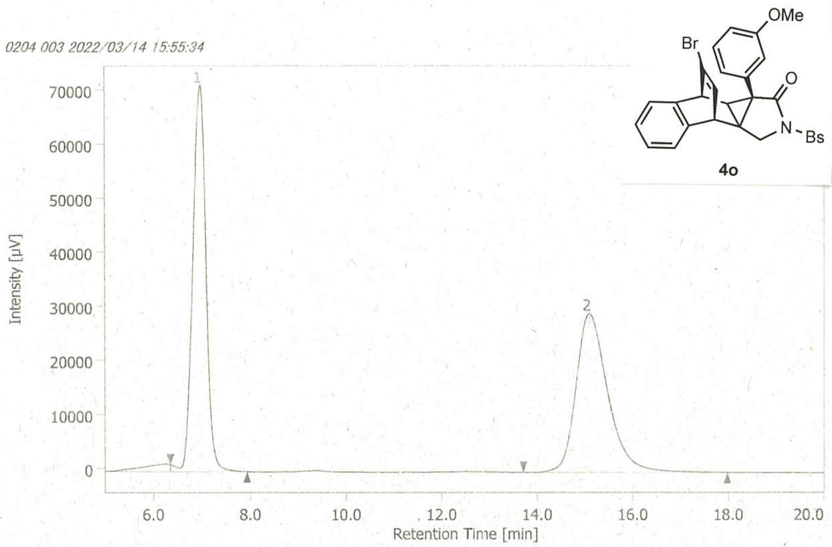

#	tR [min]	Area [µV·sec]	Area%	Height [µV]	Height%
1	6.975	1360547	50.097	71405	70.925
2	15.092	1355266	49.903	29271	29.075

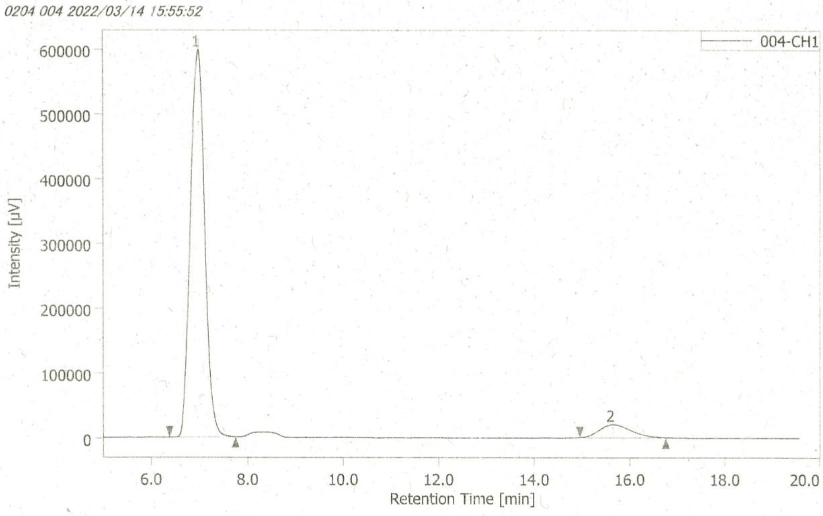

#	tR [min]	Area [µV·sec]	Area%	Height [µV]	Height%
1	6.983	12401044	93.159	597796	96.781
2	15.650	910590	6.841	19880	3.219

0930 hk5-361_003 2022/11/08 15:55:10

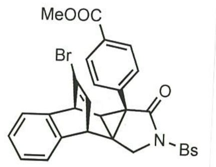

4p

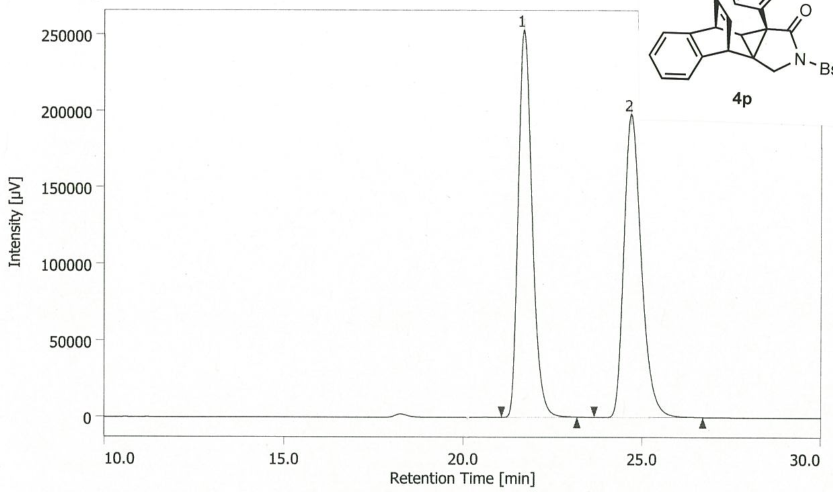

#	tR [min]	Area [µV·sec]	Area%	Height [µV]	Height%
1	21.692	6814358	50.210	253120	56.097
2	24.683	6757313	49.790	198097	43.903

1108 001 2022/11/08 15:54:09

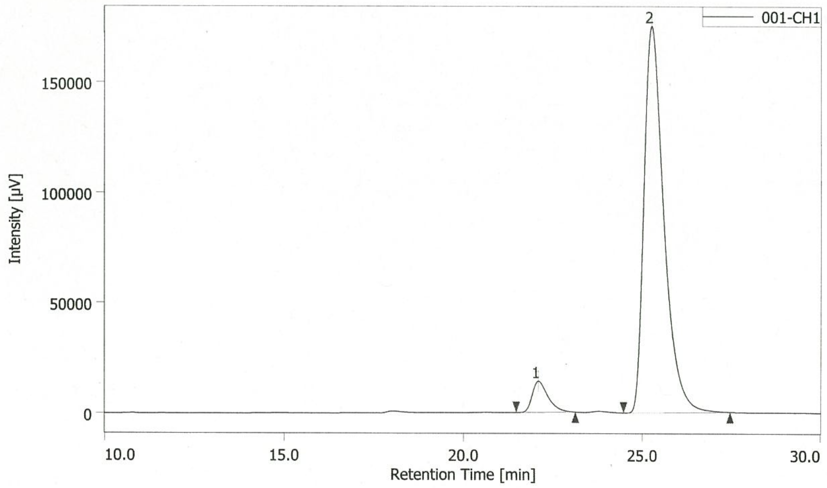

#	tR [min]	Area [µV·sec]	Area%	Height [µV]	Height%
1	22.092	429611	5.950	14127	7.472
2	25.250	6791184	94.050	174939	92.528

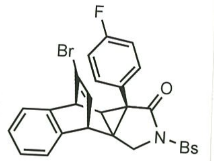

4m

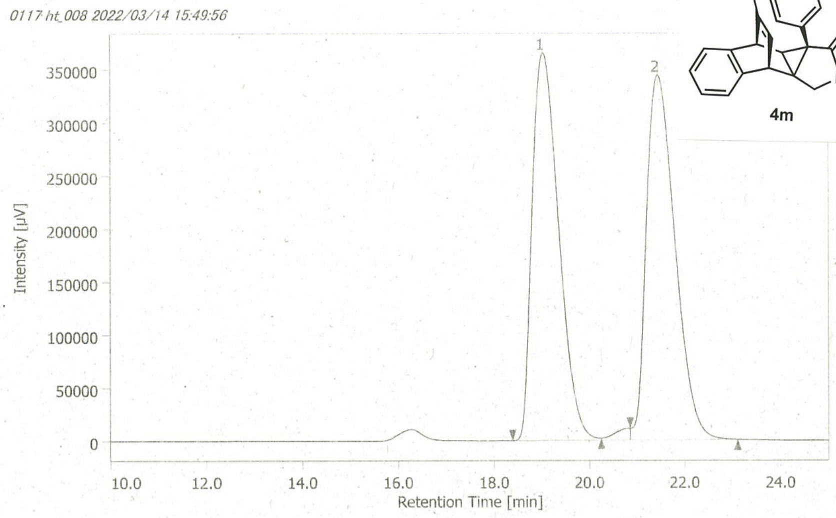

#	tR [min]	Area [µV·sec]	Area%	Height [µV]	Height%
1	19.025	14062505	49.589	365022	51.518
2	21.442	14295421	50.411	343510	48.482

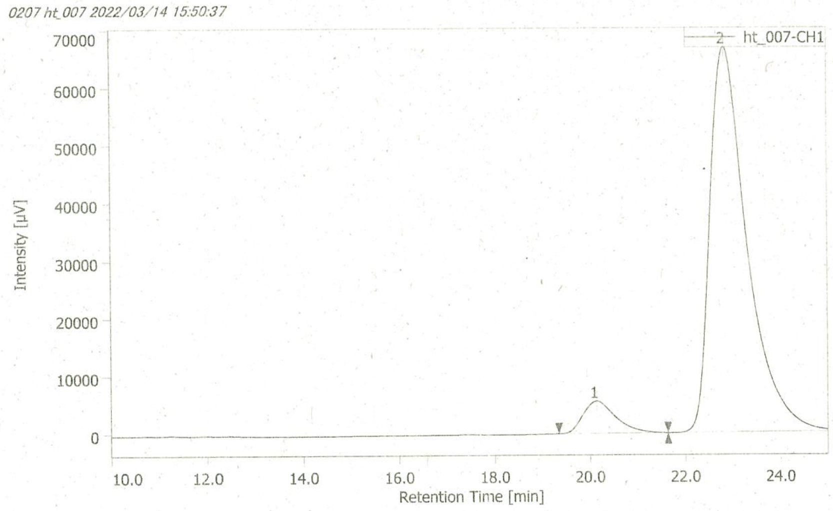

#	tR [min]	Area [µV·sec]	Area%	Height [µV]	Height%
1	20.133	264331	6.825	5634	7.785
2	22.842	3608886	93.175	66739	92.215

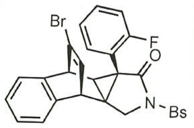

4n (major rotamer)

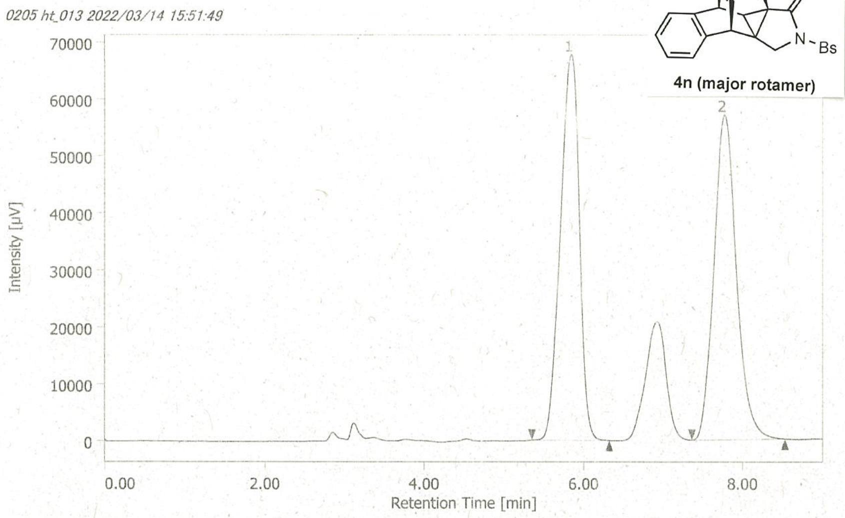

#	tR [min]	Area [µV·sec]	Area%	Height [µV]	Height%
1	5.842	1112545	49.748	67783	54.287
2	7.767	1123802	50.252	57078	45.713

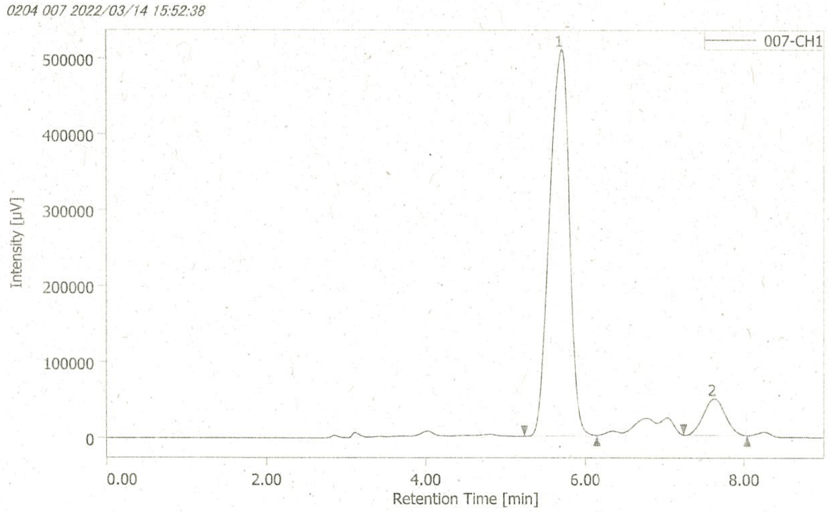

#	tR [min]	Area [µV·sec]	Area%	Height [µV]	Height%
1	5.700	8762221	90.387	508592	91.296
2	7.625	931907	9.613	48489	8.704

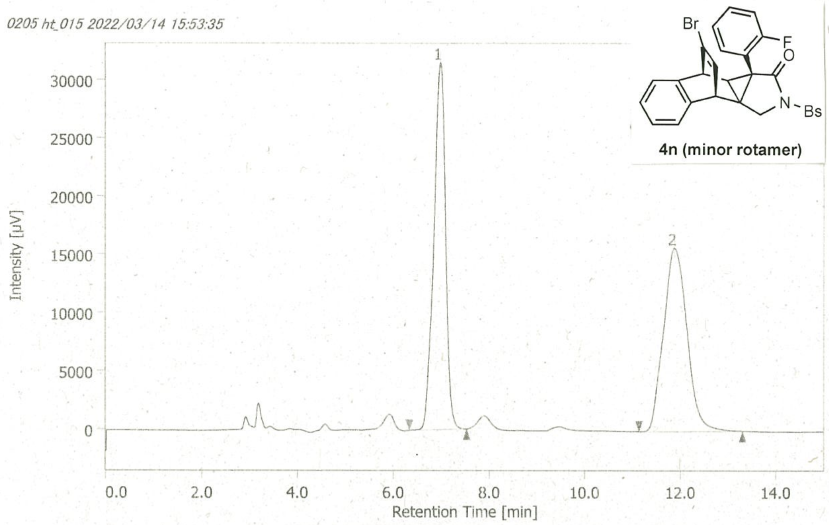

#	tR [min]	Area [μV·sec]	Area%	Height [μV]	Height%
1	6.975	534618	49.955	31485	66.724
2	11.875	535584	50.045	15702	33.276

0204 008 2022/03/14 15:54:02

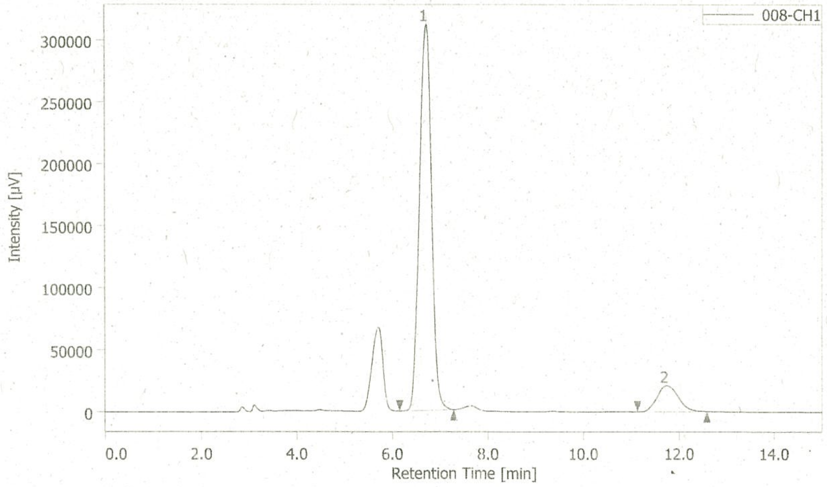

#	tR [min]	Area [μV·sec]	Area%	Height [μV]	Height%
1	6.708	5484141	89.503	311528	93.678
2	11.733	643208	10.497	21023	6.322

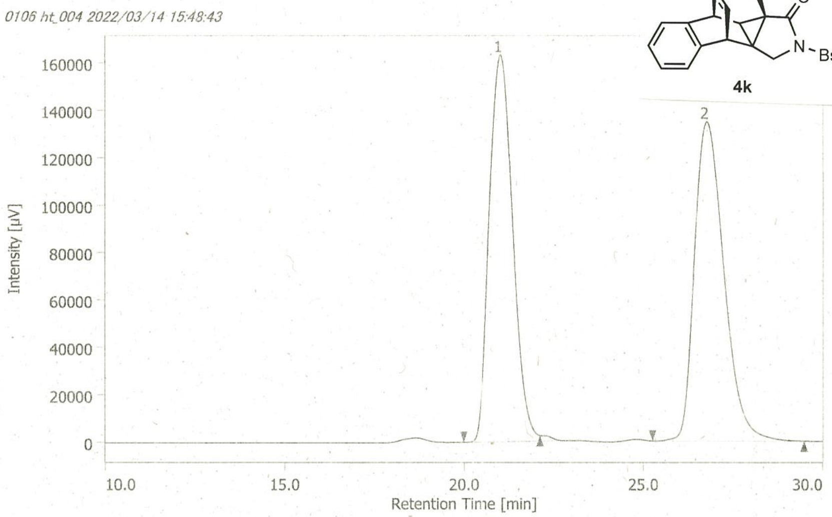

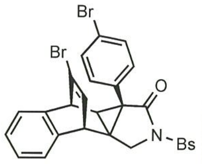

4k

#	tR [min]	Area [µV·sec]	Area%	Height [µV]	Height%
1	21.008	7373627	49.068	162922	54.749
2	26.767	7653653	50.932	134659	45.251

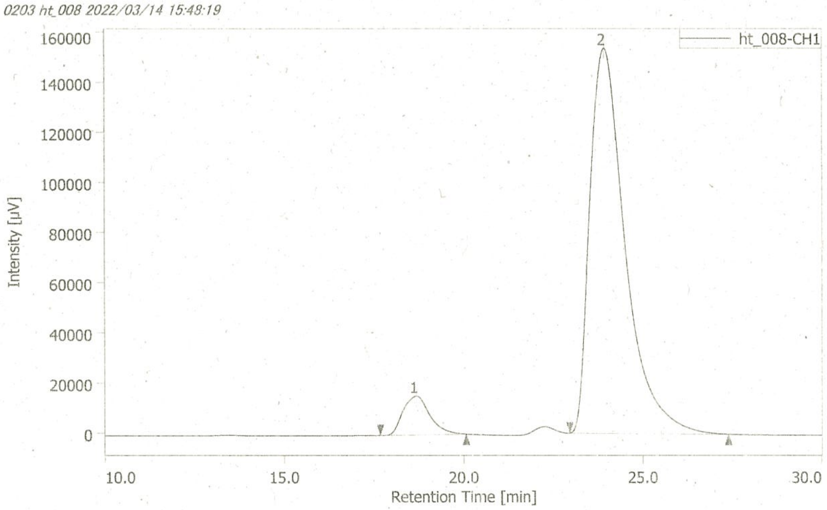

#	tR [min]	Area [µV·sec]	Area%	Height [µV]	Height%
1	18.692	806061	7.115	15542	9.188
2	23.917	10523682	92.885	153609	90.812

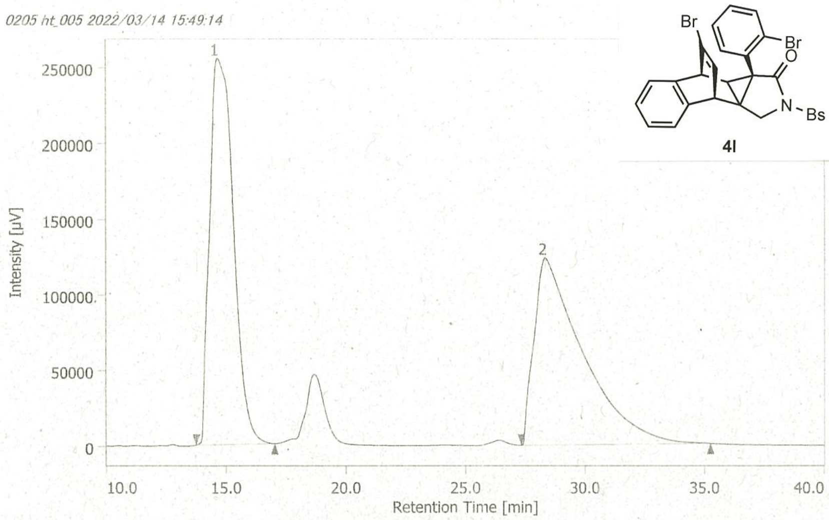

4l

#	tR [min]	Area [µV·sec]	Area%	Height [µV]	Height%
1	14.650	17393154	49.933	254885	67.359
2	28.325	17439924	50.067	123513	32.641

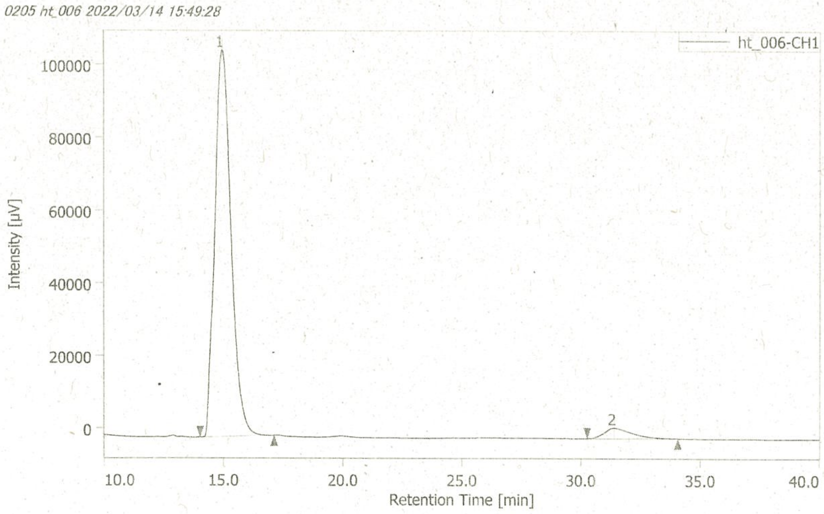

#	tR [min]	Area [µV·sec]	Area%	Height [µV]	Height%
1	14.942	5031067	95.522	106292	97.342
2	31.392	235845	4.478	2903	2.658

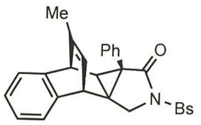

4g

0117 ht_005 2022/03/14 15:42:35

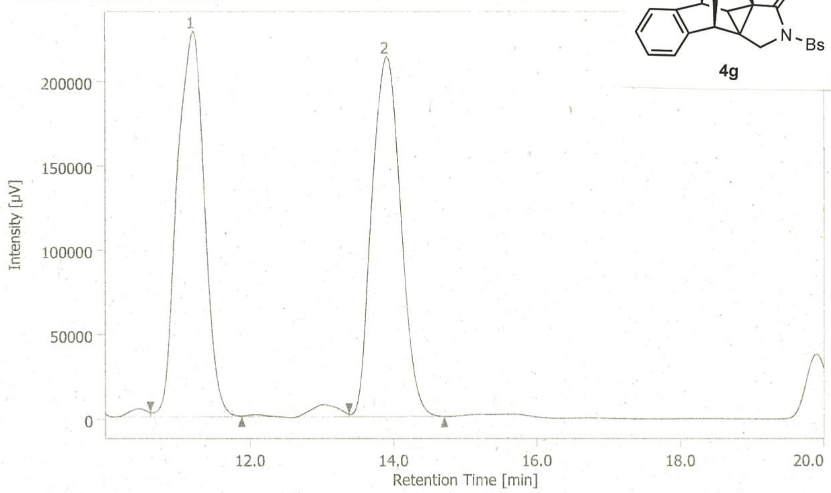

#	tR [min]	Area [µV·sec]	Area%	Height [µV]	Height%
1	11.217	6036194	50.192	228257	51.718
2	13.908	5989930	49.808	213095	48.282

0203 ht_006 2022/03/14 15:43:10

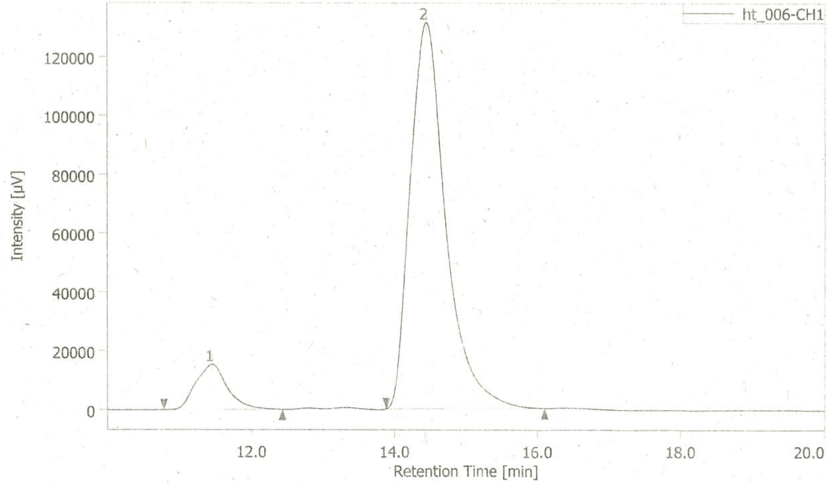

#	tR [min]	Area [µV·sec]	Area%	Height [µV]	Height%
1	11.458	465779	9.303	15367	10.487
2	14.442	4540740	90.697	131174	89.513

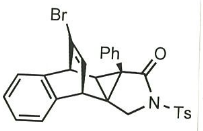

4h

1210 ht_001 2022/03/14 16:10:38

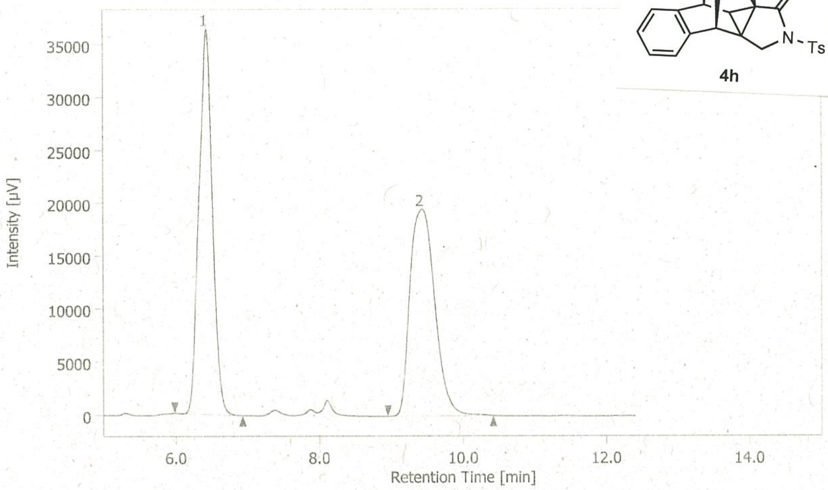

#	tR [min]	Area [μV·sec]	Area%	Height [μV]	Height%
1	6.433	473297	50.188	36389	65.128
2	9.433	469746	49.812	19484	34.872

0202 ht_001 2022/03/14 16:10:44

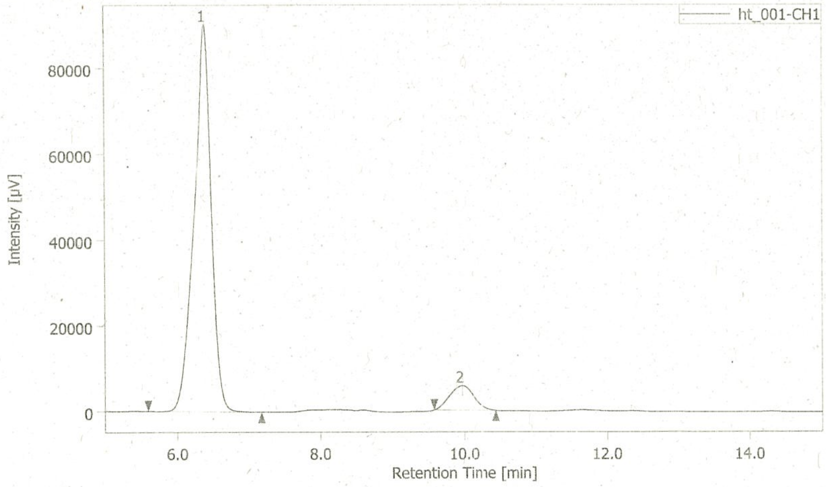

#	tR [min]	Area [μV·sec]	Area%	Height [μV]	Height%
1	6.392	1492418	91.728	90272	93.975
2	9.967	134586	8.272	5788	6.025

0117 ht_013 2022/03/14 15:45:14

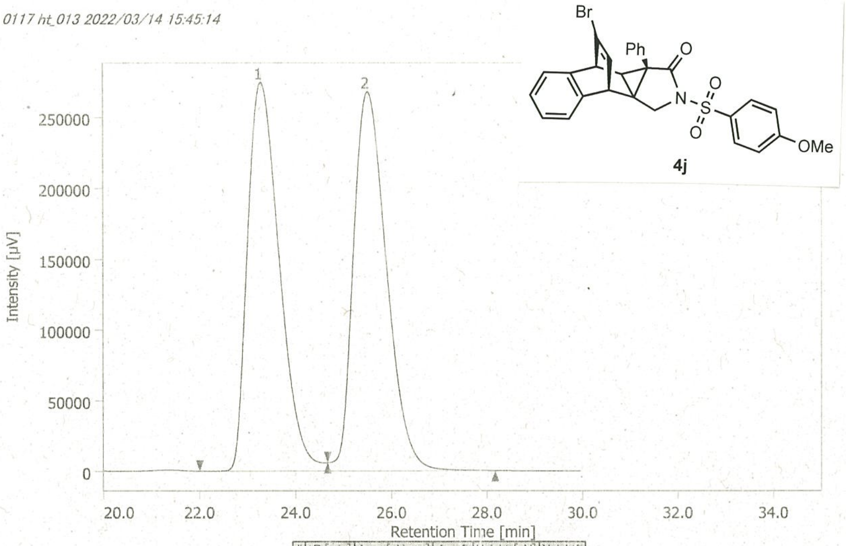

#	tR [min]	Area [µV·sec]	Area%	Height [µV]	Height%
1	23.275	12666343	49.655	274554	50.637
2	25.500	12842440	50.345	267642	49.363

0211 ht_007 2022/03/14 15:45:28

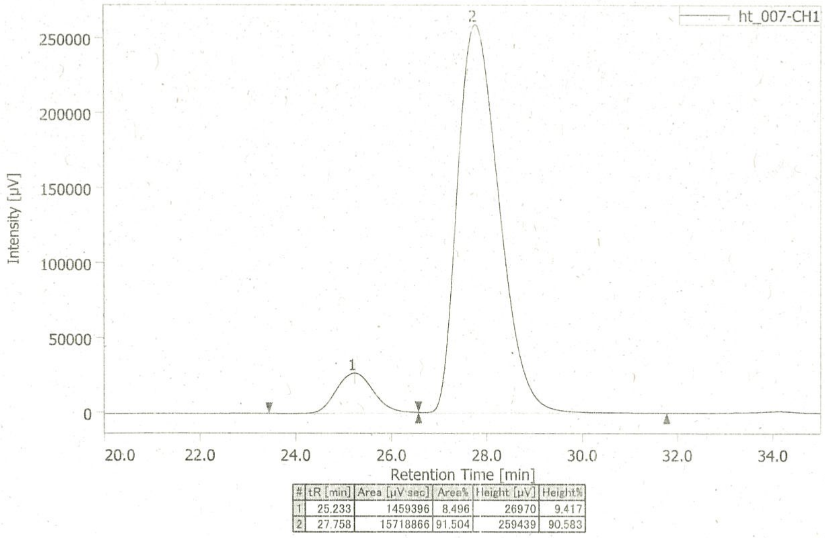

#	tR [min]	Area [µV·sec]	Area%	Height [µV]	Height%
1	25.233	1459396	8.496	26970	9.417
2	27.758	15718866	91.504	259439	90.583

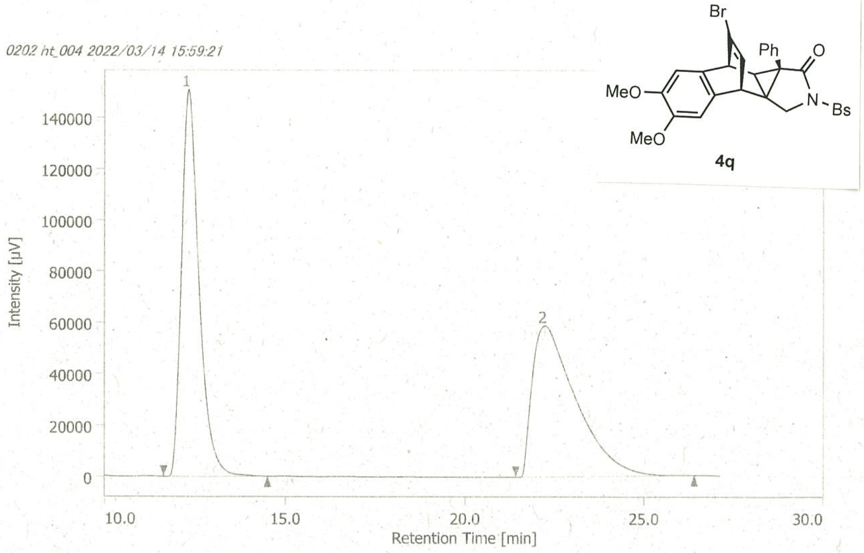

#	tR [min]	Area [µV·sec]	Area%	Height [µV]	Height%
1	12.325	5051387	50.435	150842	71.960
2	22.242	4964305	49.565	58778	28.040

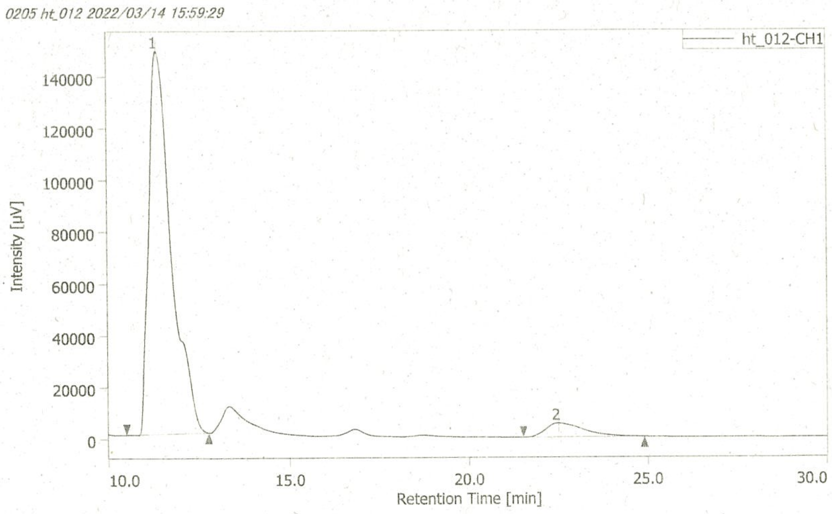

#	tR [min]	Area [µV·sec]	Area%	Height [µV]	Height%
1	11.358	6269591	94.061	147883	96.487
2	22.500	395861	5.939	5384	3.513

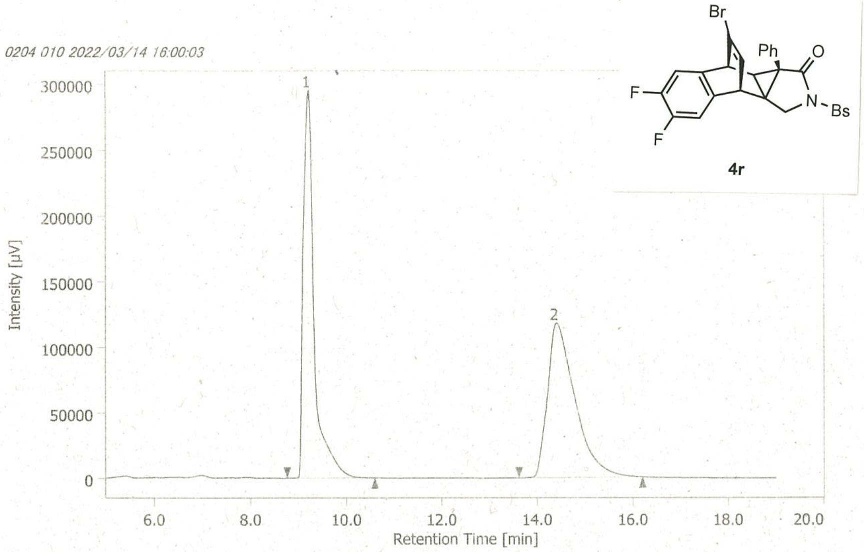

#	tR [min]	Area [μV·sec]	Area%	Height [μV]	Height%
1	9.233	4686460	50.029	295119	71.437
2	14.408	4681116	49.971	117997	28.563

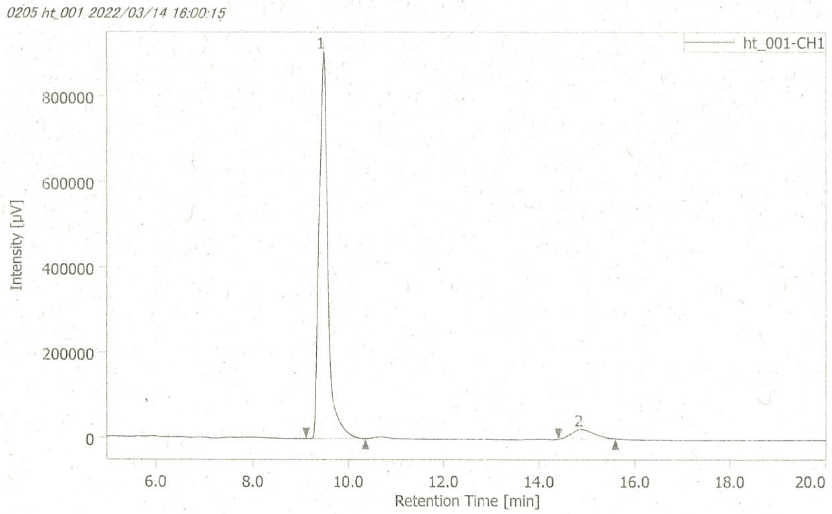

#	tR [min]	Area [μV·sec]	Area%	Height [μV]	Height%
1	9.500	11505787	93.853	907224	97.565
2	14.875	753637	6.147	22638	2.435

0205 ht_007 2022/03/14 16:01:53

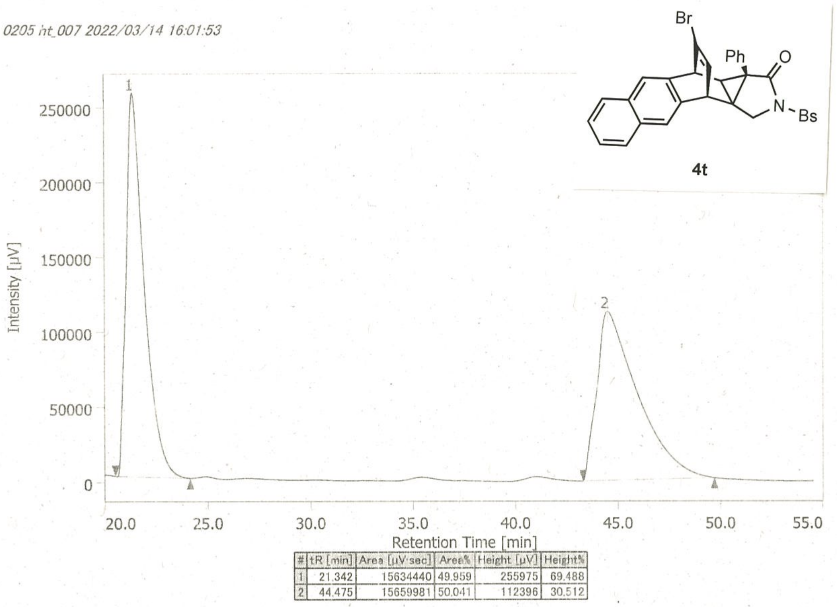

#	tR [min]	Area [μV·sec]	Area%	Height [μV]	Height%
1	21.342	15634440	49.959	255975	69.488
2	44.475	15659981	50.041	112396	30.512

0205 ht_008 2022/03/14 16:02:05

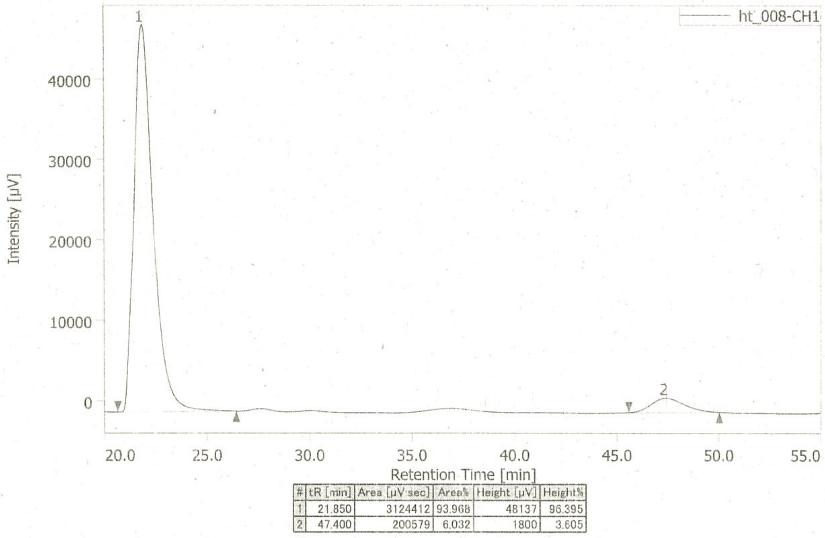

#	tR [min]	Area [μV·sec]	Area%	Height [μV]	Height%
1	21.850	3124412	93.968	48137	96.395
2	47.400	200579	6.032	1800	3.605

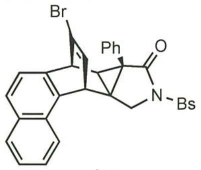

4u

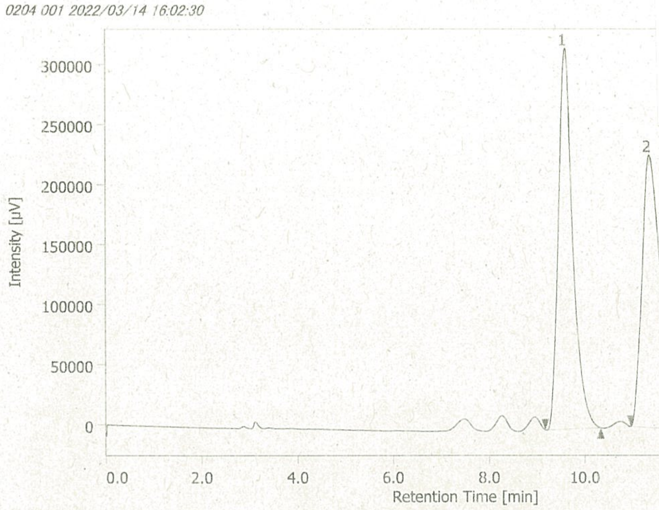

#	tR [min]	Area [µV·sec]	Area%	Height [µV]	Height%
1	9.575	6610445	50.221	316680	58.270
2	11.333	6552140	49.779	226790	41.730

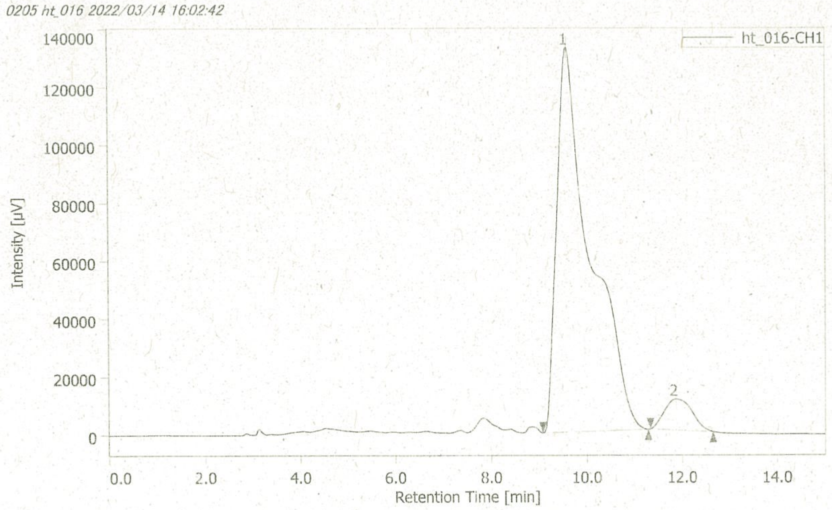

#	tR [min]	Area [µV·sec]	Area%	Height [µV]	Height%
1	9.583	6441885	93.718	132517	92.543
2	11.867	431794	6.282	10678	7.457

0204 002 2022/03/14 16:03:00

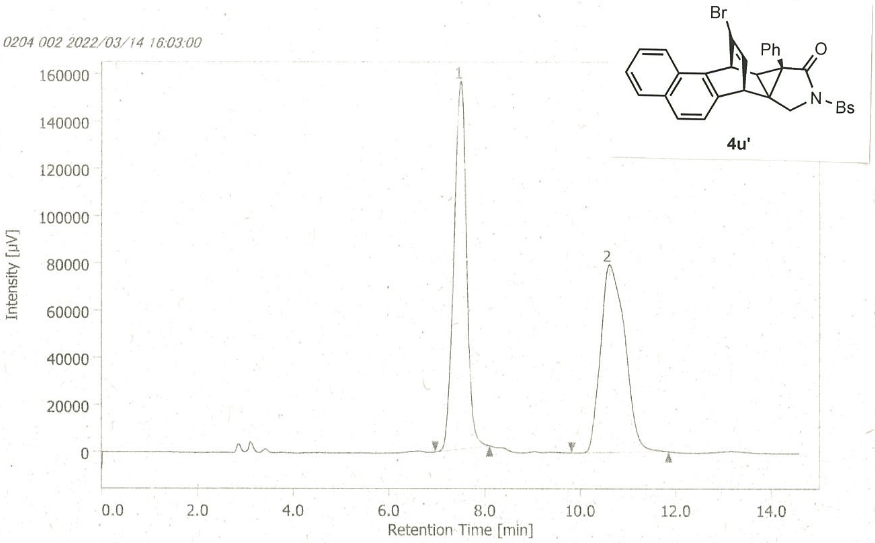

4u'

#	tR [min]	Area [µV·sec]	Area%	Height [µV]	Height%
1	7.500	2828118	50.028	155517	66.140
2	10.600	2824959	49.972	79615	33.860

0205 ht_017 2022/03/14 16:03:15

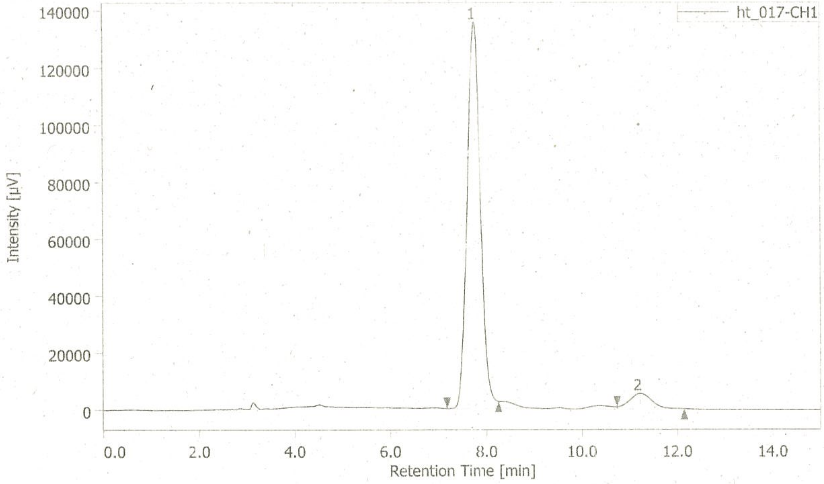

#	tR [min]	Area [µV·sec]	Area%	Height [µV]	Height%
1	7.758	2601983	93.784	135539	96.210
2	11.217	172459	6.216	5339	3.790

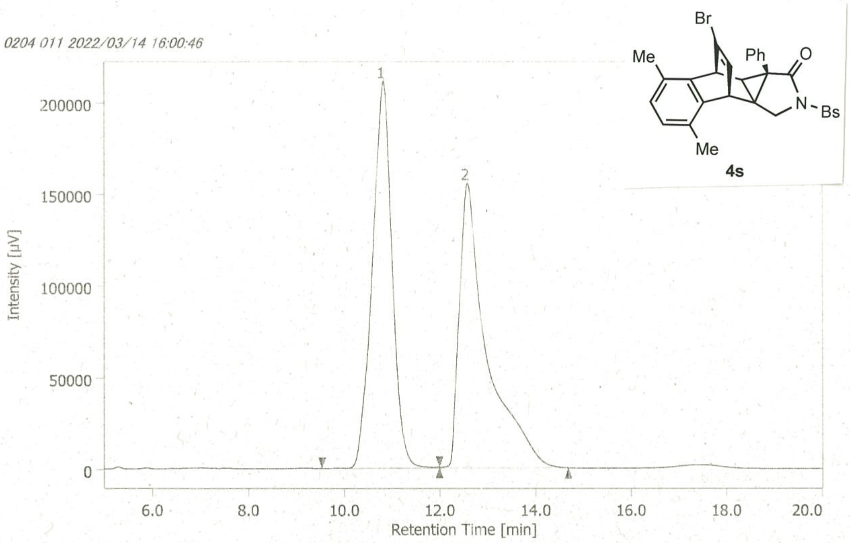

#	tR [min]	Area [µV·sec]	Area%	Height [µV]	Height%
1	10.833	6054242	49.349	211358	57.648
2	12.583	6214004	50.651	155278	42.352

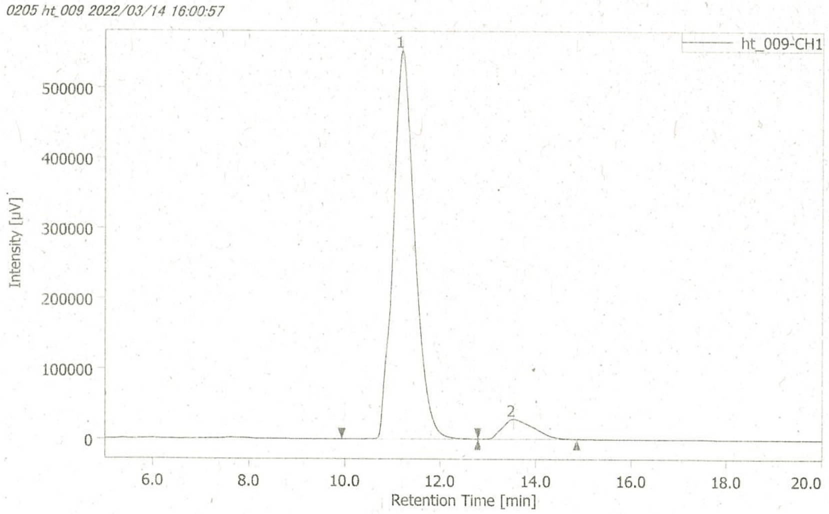

#	tR [min]	Area [µV·sec]	Area%	Height [µV]	Height%
1	11.208	17347584	93.081	552895	95.114
2	13.533	1289463	6.919	28400	4.886

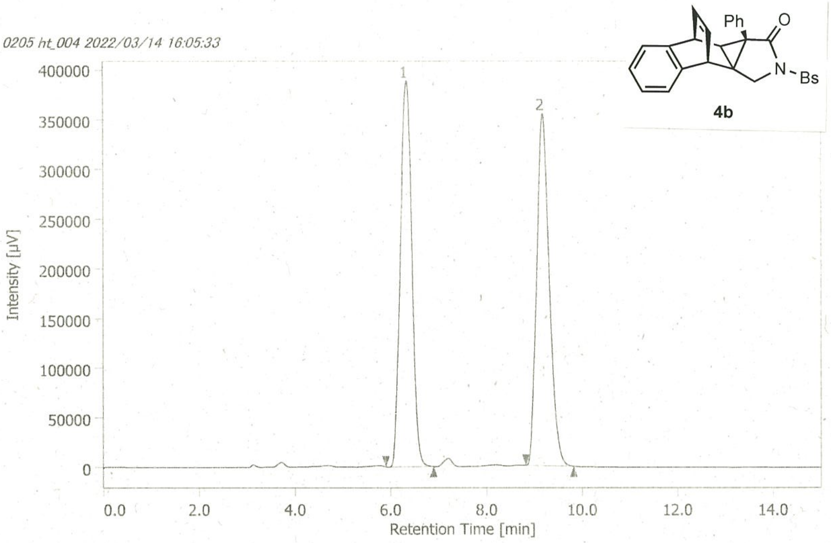

4b

#	tR [min]	Area [µV·sec]	Area%	Height [µV]	Height%
1	6.333	6507582	49.992	388956	52.281
2	9.167	6509651	50.008	355018	47.719

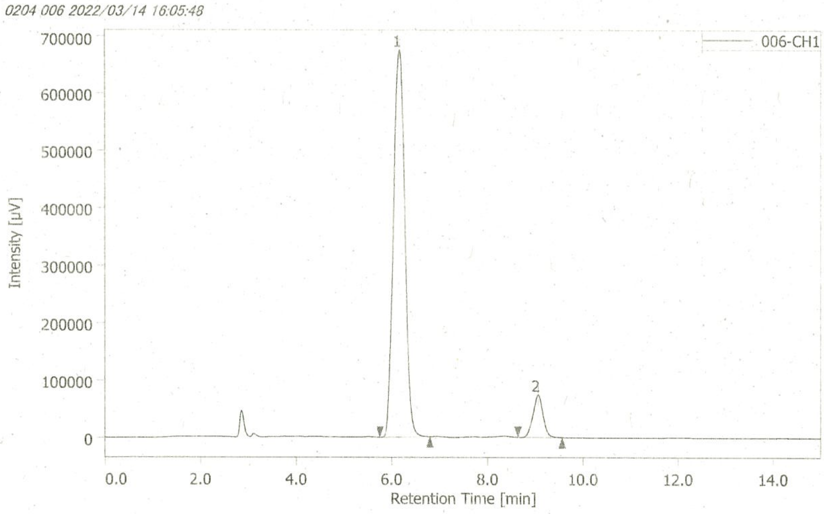

#	tR [min]	Area [µV·sec]	Area%	Height [µV]	Height%
1	6.167	11205257	91.149	674975	90.032
2	9.058	1088070	8.851	74728	9.968

0106 ht_003 2022/03/14 15:27:42

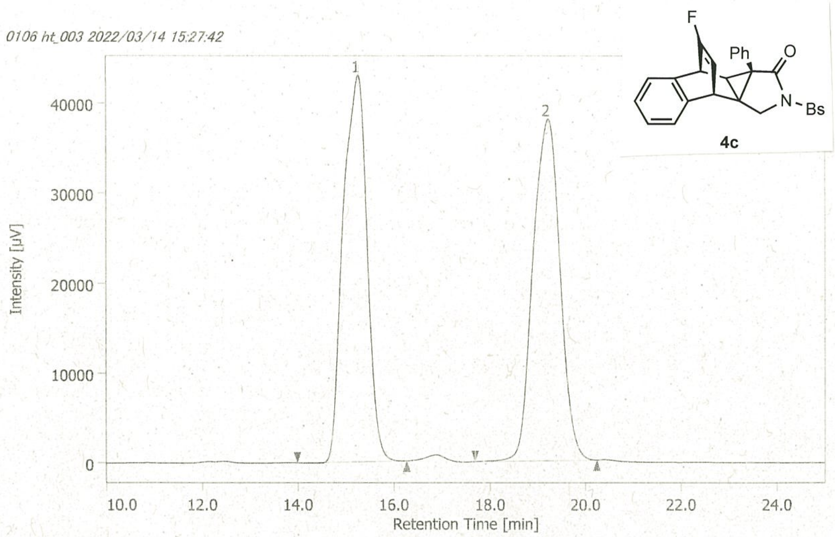

#	tR [min]	Area [μV·sec]	Area%	Height [μV]	Height%
1	15.250	1487412	49.836	42952	53.103
2	19.217	1497230	50.164	37932	46.897

0203 ht_004 2022/03/14 16:07:36

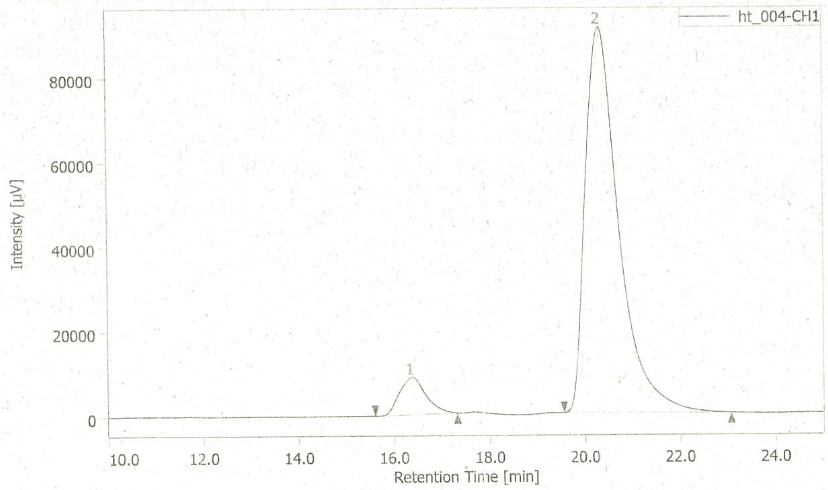

#	tR [min]	Area [μV·sec]	Area%	Height [μV]	Height%
1	16.383	341513	7.152	8894	8.903
2	20.325	4433817	92.848	91003	91.097

1217 ht_006 2022/03/14 15:28:33

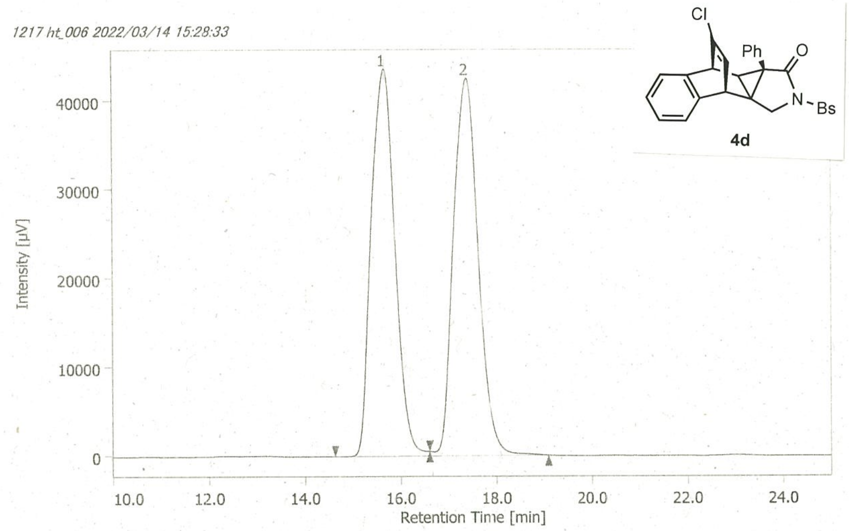

4d

#	tR [min]	Area [µV·sec]	Area%	Height [µV]	Height%
1	15.658	1499053	49.606	43599	50.651
2	17.383	1522882	50.394	42479	49.349

0203 ht_005 2022/03/14 15:30:17

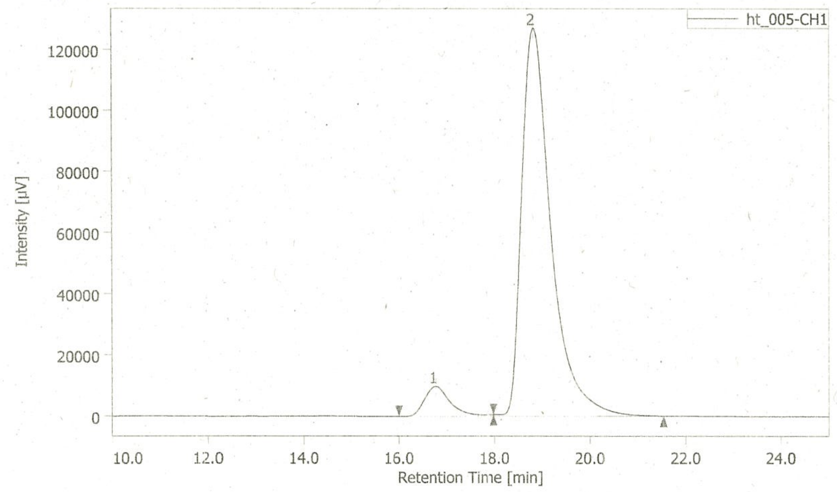

#	tR [min]	Area [µV·sec]	Area%	Height [µV]	Height%
1	16.775	371250	6.423	9892	7.225
2	18.808	5408486	93.577	127017	92.775

0111 ht_001 2022/03/14 15:38:33

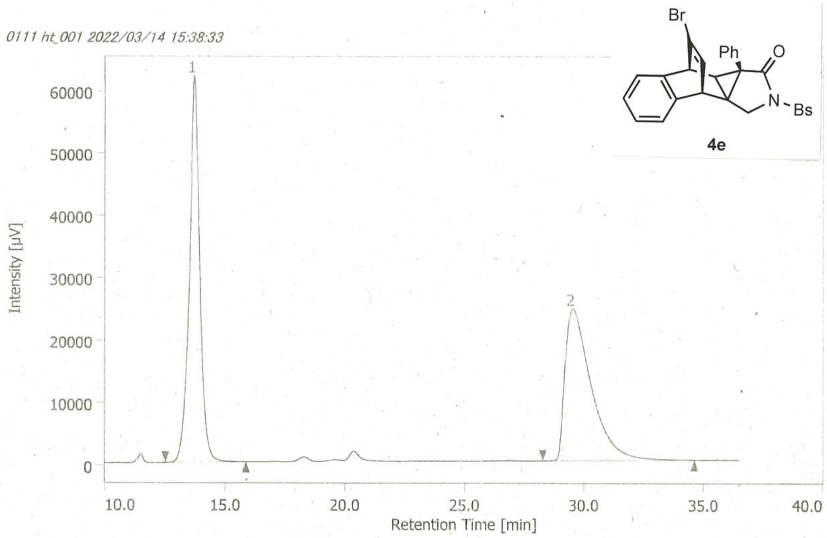

#	tR [min]	Area [µV·sec]	Area%	Height [µV]	Height%
1	13.725	1853856	50.136	61976	71.840
2	29.533	1843777	49.864	24294	28.160

0202 ht_005 2022/03/14 15:39:23

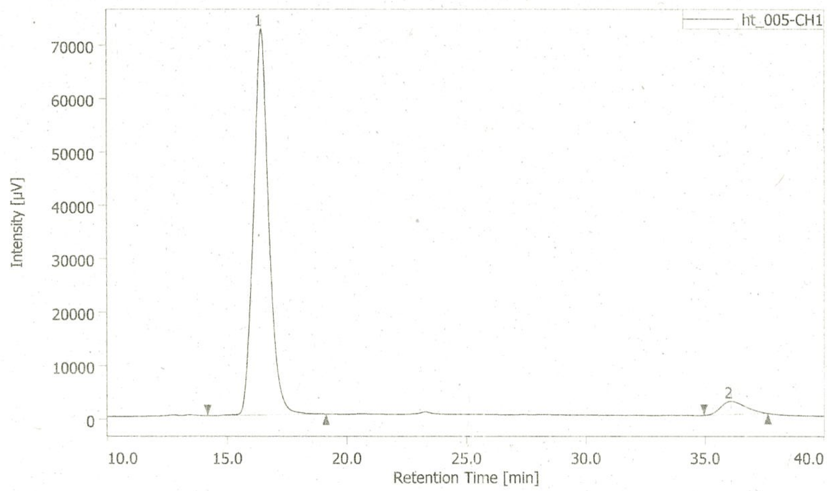

#	tR [min]	Area [µV·sec]	Area%	Height [µV]	Height%
1	16.433	3206183	94.660	72229	96.701
2	36.075	180881	5.340	2464	3.299

1210 ht_009 2022/03/14 15:40:00

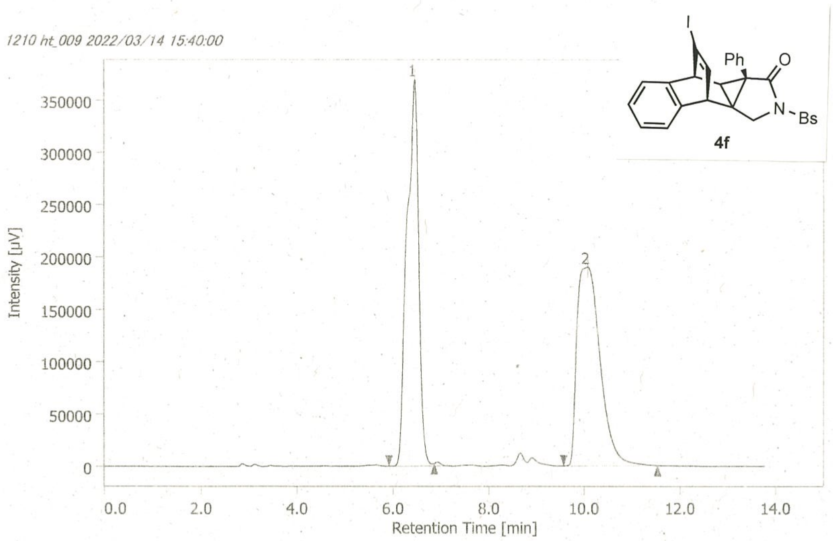

#	tR [min]	Area [μV·sec]	Area%	Height [μV]	Height%
1	6.450	6300096	49.748	369531	66.059
2	10.075	6364021	50.252	189867	33.941

0202 ht_002 2022/03/14 15:39:35

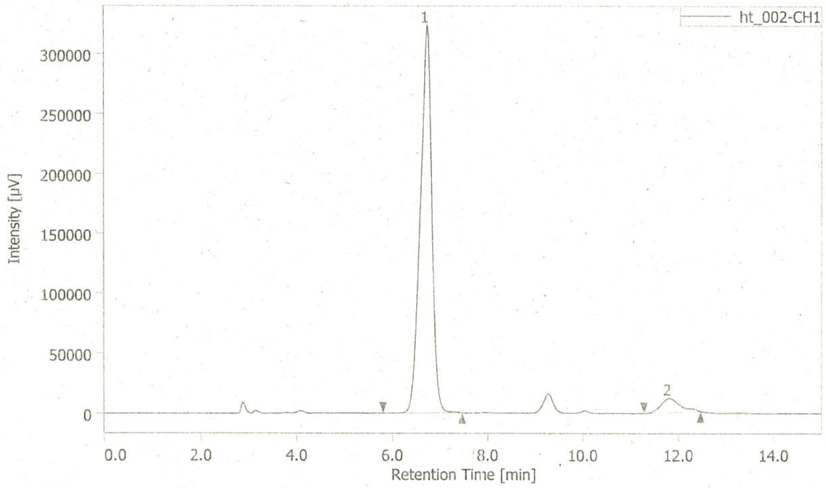

#	tR [min]	Area [μV·sec]	Area%	Height [μV]	Height%
1	6.733	5382802	94.192	323226	96.533
2	11.808	331917	5.808	11607	3.467

1216 x Bs p-CN Aryne racemi_001 2024/01/06 17:46:12

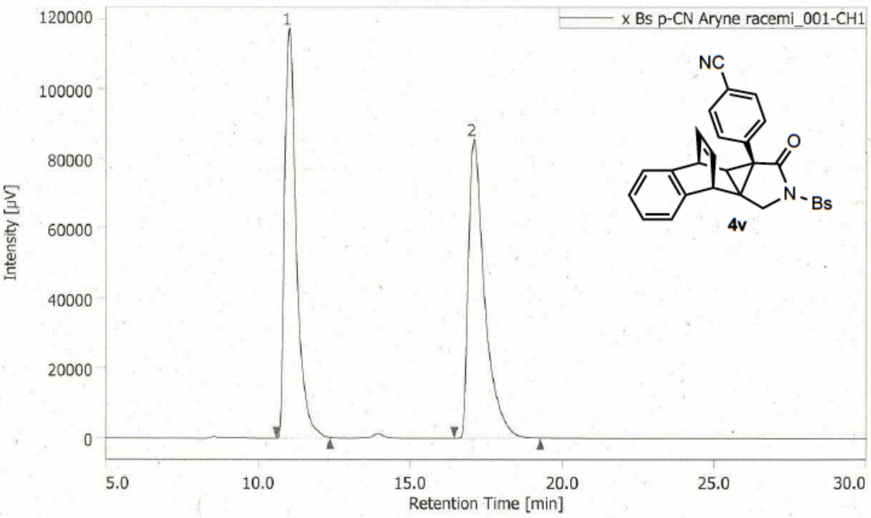

#	tR [min]	Area [µV·sec]	Area%	Height [µV]	Height%
1	11.025	3183587	50.164	117677	57.856
2	17.092	3162757	49.836	85720	42.144

1221 hk-11-849_003 2024/01/06 17:46:05

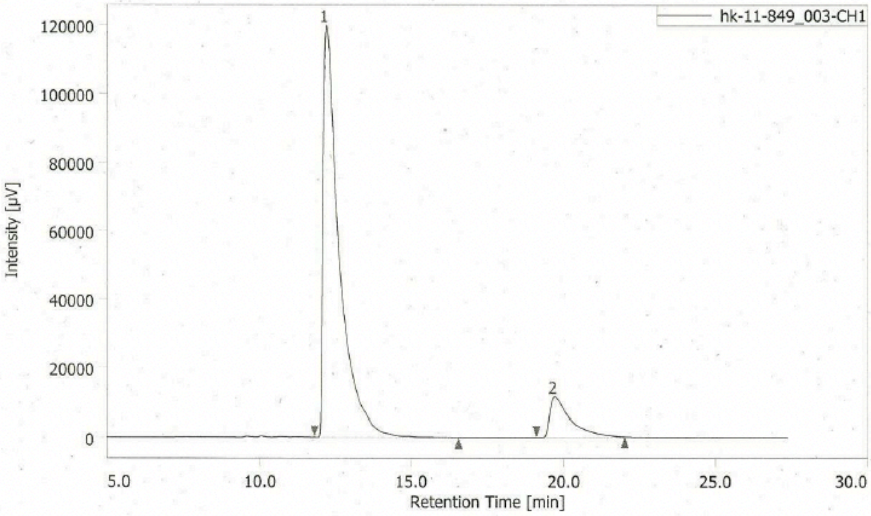

#	tR [min]	Area [µV·sec]	Area%	Height [µV]	Height%
1	12.208	4444940	88.986	119760	91.292
2	19.708	550171	11.014	11424	8.708

TI 4tBu aryne racemate_0104 sys2 IH3 HD 8020_001 2024/01/04 19:32:17

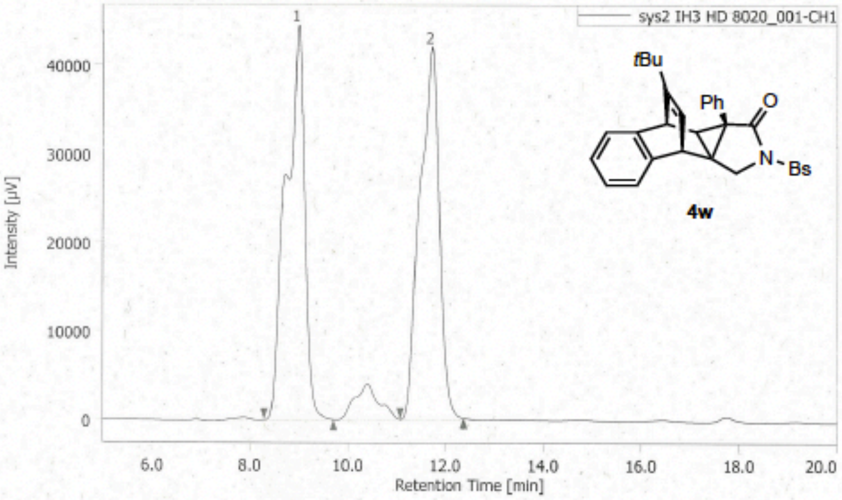

#	tR [min]	Area [μV·sec]	Area%	Height [μV]	Height%
1	9.008	1171689	50.027	44505	51.467
2	11.733	1170440	49.973	41967	48.533

TI 4tBu aryne asymmetric_0104 sys2 IH3 HD 8020_003 2024/01/04 19:32:56

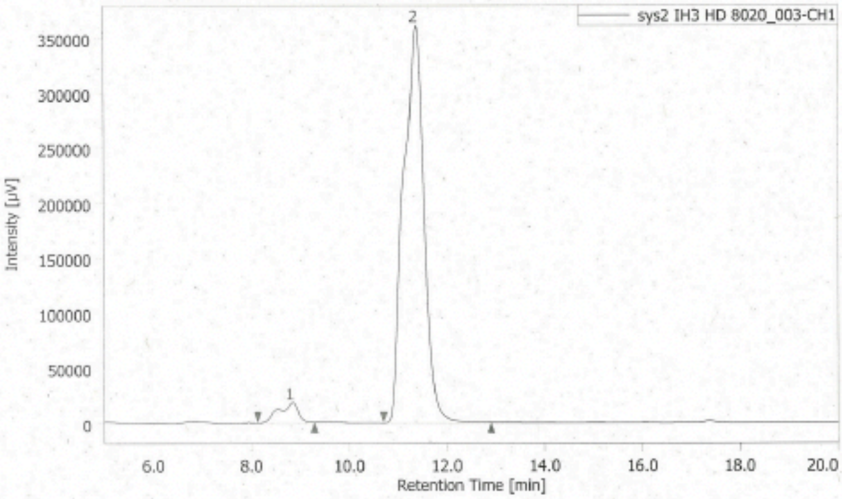

#	tR [min]	Area [μV·sec]	Area%	Height [μV]	Height%
1	8.850	525264	4.724	18693	4.920
2	11.392	10592686	95.276	361274	95.080

4 Ph aryne racemate_1228 IBN3 HD 8020_007 2023/12/28 17:57:42

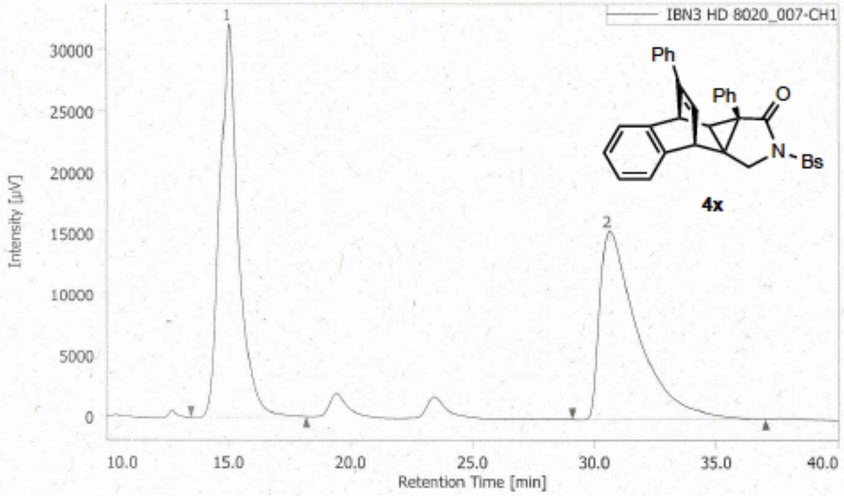

#	tR [min]	Area [μV·sec]	Area%	Height [μV]	Height%
1	15.042	1694980	50.265	32196	67.573
2	30.608	1677099	49.735	15450	32.427

TI 4Ph aryne asymmetric_0105 sys2 IBN3 HD 8020_001 2024/01/05 14:40:43

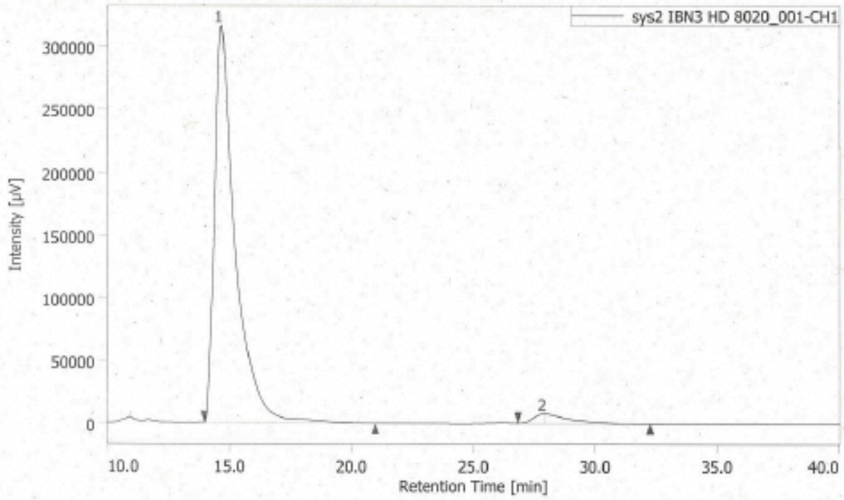

#	tR [min]	Area [μV·sec]	Area%	Height [μV]	Height%
1	14.667	18559596	96.160	316074	97.670
2	27.917	741088	3.840	7539	2.330

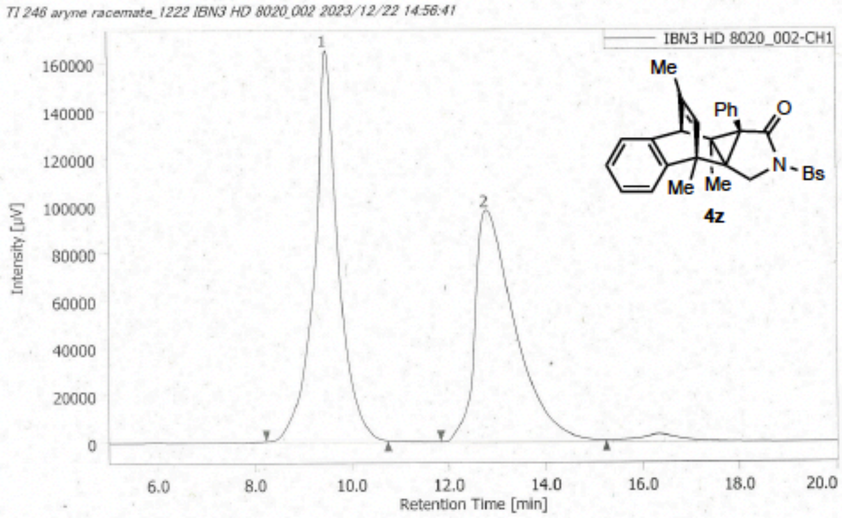

4z

#	tR [min]	Area [µV·sec]	Area%	Height [µV]	Height%
1	9.492	5797738	50.687	165460	62.823
2	12.800	5640522	49.313	97917	37.177

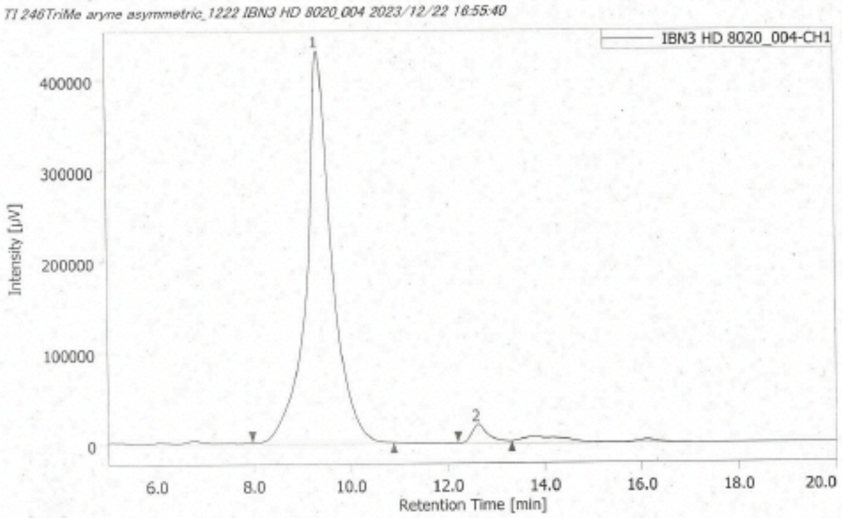

#	tR [min]	Area [µV·sec]	Area%	Height [µV]	Height%
1	9.358	15804158	96.933	431177	95.503
2	12.633	499992	3.067	20303	4.497

14. References

1. Humphrey, W., Dalke, A. & Schulten, K. VMD: Visual Molecular Dynamics. *J. Mol. Graphics* **14**, 33–38 (1996).
2. Ito, T., Harada, S., Homma, H., Takenaka, H., Hirose, S. & Nemoto, T. Asymmetric Intramolecular Dearomatization of Nonactivated Arenes with Ynamides for Rapid Assembly of Fused Ring System under Silver Catalysis. *J. Am. Chem. Soc.* **143**, 604–611 (2021).
3. González-Soria, M. J. & Alonso, F. Substrate-Controlled Divergent Synthesis of Enaminones and Pyrroles from Indolizines and Nitroso Compounds. *Adv. Synth. Catal.* **361**, 5005–5017 (2019).
4. Jiang, H., Zhang, Y., Xiong, W., Cen, J., Wang, L., Cheng, R., Qi, C. & Wu, W. A Three-Phase Four-Component Coupling Reaction: Selective Synthesis of *o*-Chloro Benzoates by KCl, Arynes, CO_2, and Chloroalkanes. *Org. Lett.* **21**, 345−349 (2019).
