# Supplementary material for: Valence-isomer selective cycloaddition reaction of cycloheptatrienes-norcaradienes
Source: Nat Commun. 2024 Mar 14;15:2309. doi: 10.1038/s41467-024-46523-1 (PMC10940685; doi:10.1038/s41467-024-46523-1)
Supplement: Supplementary file 4 — Supplementary Software 1 [file 41467_2024_46523_MOESM4_ESM.zip › Supplementary Software 1/Description of Additional Supplementary Files.pdf]

Description of Additional Supplementary Files

- README.md: A markdown file explaining how to use the predictor.
- CHTNCD_HOBO.pkl: A pickle file of the saved model for HOBO of CHT/NCD.
- EP_LUBO.pkl: A pickle file of the saved model for LUBO of EP.
- Final_CHT_Submit: Source code of the machine learning for CHT.
- Final_EP_Submit: Source code of the machine learning for EP.
- DEMO.csv: A demonstration file to test the predictor.
- HOBO_LUBO_Predictor.py: A python file of the predictor.
- S1.png: A portable network graphic file for README.md (image S1).
- S2.png: A portable network graphic file for README.md (image S2).
- S3.png: A portable network graphic file for README.md (image S3).
- S4.png: A portable network graphic file for README.md (image S4).
- S5.png: A portable network graphic file for README.md (image S5).
- S6.png: A portable network graphic file for README.md (image S6).
- S7.png: A portable network graphic file for README.md (image S7).
- S8.png: A portable network graphic file for README.md (image S8).
