# Supplementary material for: Valence-isomer selective cycloaddition reaction of cycloheptatrienes-norcaradienes
Source: Nat Commun. 2024 Mar 14;15:2309. doi: 10.1038/s41467-024-46523-1 (PMC10940685; doi:10.1038/s41467-024-46523-1)
Supplement: Supplementary file 4 — Supplementary Software 1 [file 41467_2024_46523_MOESM4_ESM.zip › Supplementary Software 1/Final_EP_Submit.pdf]

```python
# Tsubasa Ito, Chiba Univ.

import pandas as pd
import matplotlib.pyplot as plt
from multiprocessing import get_context,Pool
import pickle, os, warnings
from sklearn.metrics import mean_absolute_error
from sklearn.model_selection import train_test_split
from sklearn.neural_network  import MLPRegressor
import lightgbm as lgb
from sklearn.linear_model import ElasticNet

##############################################################################################################
warnings.simplefilter('ignore')
path_1 = str(os.path.dirname(os.path.abspath(__file__)) + "/")
path_2 = path_1 + "Result/"
path_3 = path_1 + "csv/"
Range_01 = range(31)
##############################################################################################################

class Final_Try():

    # def01 Writing result of tuning on txt-files.
    def Writeindex(self, content, path = path_2):

        f = open(path + self.Name.split(".")[0] + "_Final" + ".txt", "a")
        f.write(str(content) + "\n")
        f.close

    # def02 Creating feature values and answer data from a data frame
    @classmethod
    def Mk_xt(cls, Pro_df):

        x = Pro_df.iloc[:,4:]
        t = Pro_df['LUBO']

        return x, t

    # def03 Sorting in ascending and descending order, referring to a specific order of elements in the list
    @classmethod
    def sorting(cls,list_1, num, Keyword = "descending"):

        sortvalues = (lambda val : val[num])
        if Keyword == "ascending":
            list_2 = sorted(list_1, reverse=False, key=sortvalues)
        else:
            list_2 = sorted(list_1, reverse=True, key=sortvalues)

        return list_2

    # def04 calculating the average
    @classmethod
    def AVERAGE_STATUS(cls, list_1):

        Status = sum(list_1) / len(list_1)
        return Status

```

```python
    # def05 Detailed Machine learning model
    def ML(self, train_x, train_t, test_x, test_t, Random_state_num):

        # Model information
        self.model.fit(train_x, train_t)
        train_score = self.model.score(train_x, train_t)
        test_score = self.model.score(test_x,test_t)
        MAE = mean_absolute_error(test_t, self.model.predict(test_x))
        Result_list = [train_score, test_score, MAE, self.model, Random_state_num]

        return Result_list

    # def06 Creating training data and labeled training data, and running machine learning
    def Final_Analysis(self, Random_state_num):

        train_val, test = train_test_split(self.df, test_size = 0.20, random_state = Random_state_num)
        train_x, train_t = self.Mk_xt(train_val) # def02
        test_x, test_t = self.Mk_xt(test) # def02
        Result_list = self.ML(train_x, train_t, test_x, test_t, Random_state_num) # def05
        Data_list = [Random_state_num, test_x, test_t, Result_list[3]]
        Result_list.append(Data_list)

        return Result_list

    # def07 Running Model tuneing processes in parallel
    def ParallelProcessing(self, values, range_01 = 30):

        p = get_context('forkserver').Pool(range_01) # Runing 30 processes in parallel
        list_1 = p.map(self.Final_Analysis, values) # def06
        p.close()

        return list_1

    # def08  running def07
    def Main_func1(self):

        values = [a for a in Range_01]
        Result_list = self.ParallelProcessing(values) # def07
        Result_list = self.sorting(Result_list, 4, "ascending") # def03 refer to R2 element in Result_list and sort in descending order

        return Result_list

    # def09 Organizing and outputting machine learning results
    def Main_func2(self, Result_list):

        train_score_list = []
        test_score_list = []
        MAE_list = []

        for i in Result_list:
            comment1 = f'Random_state: {i[4]}'
            comment2 = f'R2 of all train deta: {i[0]}, R2 of all test deta: {i[1]}, MAE:{i[2]}'
            print(comment1,comment2)
```

```
                self.Writeindex(comment1) # def01
                self.Writeindex(comment2) # def01
                train_score_list.append(float(i[0]))
                test_score_list.append(float(i[1]))
                MAE_list.append(float(i[2]))

        train_last_status = self.AVERAGE_STATUS(train_score_list) # def04
        test_last_status  = self.AVERAGE_STATUS(test_score_list)  # def04
        MAE_last_status   = self.AVERAGE_STATUS(MAE_list)         # def04
        comment3 = f'\nThe average R2 of the all train deta:{train_last_status}\nThe average R2 of the all test deta:{test_last_status}\nMAE:{MAE_last_status}'
        print(comment3)
        self.Writeindex(comment3) # def01

        For_SAVE_List = self.sorting(Result_list, 1)[0][5] # def03

        return For_SAVE_List # Result

    # def10 Saving the best model, creating a diagram for predictive performance evaluation
    def MODEL_SAVE(self, Random_state_num, test_x, test_t, Learned_model):

     with open (self.pkl, 'wb') as f:
        pickle.dump(Learned_model, f)
        fig = plt.figure()
        plt.scatter(test_t, Learned_model.predict(test_x), alpha = 0.2, c = "blue")
        plt.plot([test_t.min(), test_t.max()], [test_t.min(), test_t.max()], c = "black")
        plt.grid()
        plt.xlabel("Real Y")
        plt.ylabel("Predicted Y")
        fig.savefig(self.pg + "_" + str(Random_state_num) + ".png")

    # def11
    def RUN(self):

        Result_list = self.Main_func1()
        For_Save_list = self.Main_func2(Result_list)
        self.MODEL_SAVE(For_Save_list[0], For_Save_list[1], For_Save_list[2], For_Save_list[3])

    # def12
    def __init__(self, model=None, BASE_NAME=None, PKL_NAME=None):

        self.model = model
        self.pg =  path_2 + BASE_NAME
        cf =  path_3 + BASE_NAME + ".csv"
        self.df = pd.read_csv(cf) # Extract Data frame
        self.Name = PKL_NAME
        self.pkl = path_2 + str(PKL_NAME) + ".pkl"


class Neural_Network(Final_Try):
    def __init__(self, BASE_NAME=None, PKL_NAME=None, Activation=None, hls = None):

        model = MLPRegressor(hidden_layer_sizes= hls, activation= Activation, solver= "lbfgs", alpha= 0.001, batch_size= 'auto', learning_rate= "constant",
```

```
            learning_rate_init= 0.001, power_t= 0.5, max_iter= 200, shuffle= True, random_state= 0, tol= 0.0001, verbose= False, warm_start= False, momentum= 0.9,
            nesterovs_momentum= True, early_stopping= True, validation_fraction= 0.1, beta_1= 0.9, beta_2= 0.999, epsilon= 1e-08, n_iter_no_change= 10, max_fun= 15000)
        super().__init__(model, BASE_NAME, PKL_NAME)


class LGBM(Final_Try):

    def __init__(self, BASE_NAME=None, PKL_NAME=None, Bo=None, Nu=None, Al=None, La=None, Mi=None, N_e=None):

        model = lgb.LGBMRegressor(boosting_type= Bo, num_leaves= Nu, max_depth= -1, learning_rate= 0.1, n_estimators= N_e,
                    subsample_for_bin= 200000, objective= None, class_weight= None, min_split_gain= 0.0, min_child_weight= 0.001,
                    min_child_samples= Mi, subsample= 1.0, subsample_freq=0, colsample_bytree= 1.0, reg_alpha= Al, reg_lambda= La,
                    random_state= 0, n_jobs= None, importance_type= "split")
        super().__init__(model, BASE_NAME, PKL_NAME)


if __name__ =='__main__' :

    No_1 = Neural_Network(BASE_NAME="EP_Avalon_2048", PKL_NAME="EP_NN_Avalon_2048", Activation="relu", hls=(70,25,90))
    No_1.RUN()
    No_2 = Neural_Network(BASE_NAME="EP_Avalon_4096", PKL_NAME="EP_NN_Avalon_4096", Activation="relu", hls=(70,35,90))
    No_2.RUN()
    No_3 = LGBM(BASE_NAME="EP_Avalon_2048", PKL_NAME="EP_LGBM_Avalon_2048", Bo="gbdt", Nu=14, Al=0, La=0.01, Mi=2, N_e=187)
    No_3.RUN()
    No_4 = Neural_Network(BASE_NAME="EP_Avalon_1024", PKL_NAME="EP_NN_Avalon_1024", Activation="relu", hls=(70,5,60))
    No_4.RUN()
    No_5 = Neural_Network(BASE_NAME="EP_MACCS", PKL_NAME="EP_NN_MACCS", Activation="logistic", hls=(80,5,5))
    No_5.RUN()
```
