# Supplementary figures and images for: Valence-isomer selective cycloaddition reaction of cycloheptatrienes-norcaradienes
Source: Nat Commun. 2024 Mar 14;15:2309. doi: 10.1038/s41467-024-46523-1 (PMC10940685; doi:10.1038/s41467-024-46523-1)

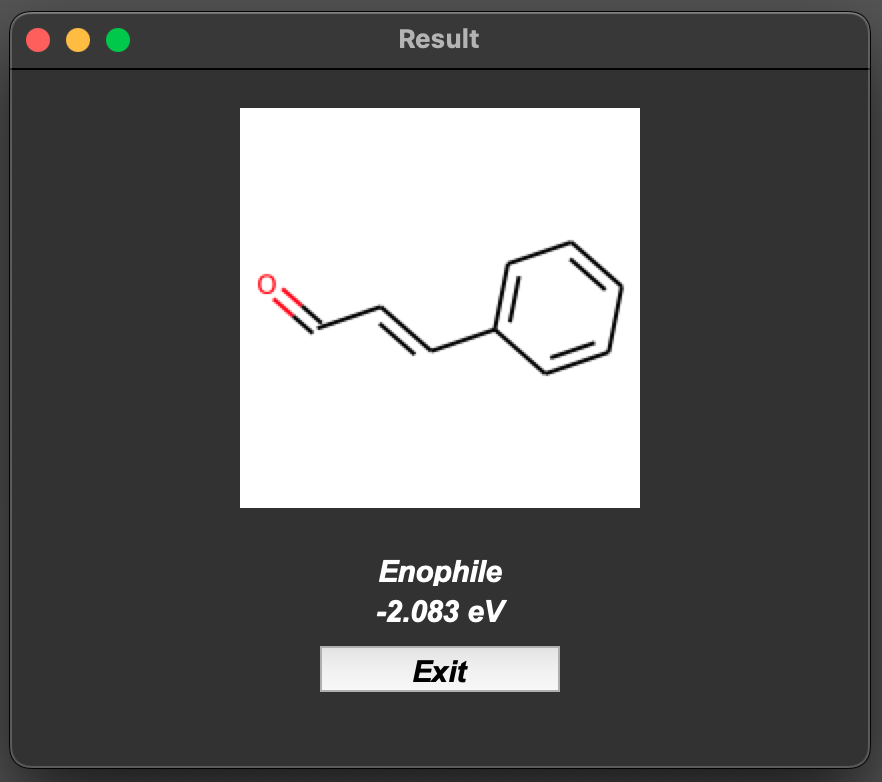

Supplement: Supplementary file 4 — Supplementary Software 1 [file 41467_2024_46523_MOESM4_ESM.zip › Supplementary Software 1/S6.png]

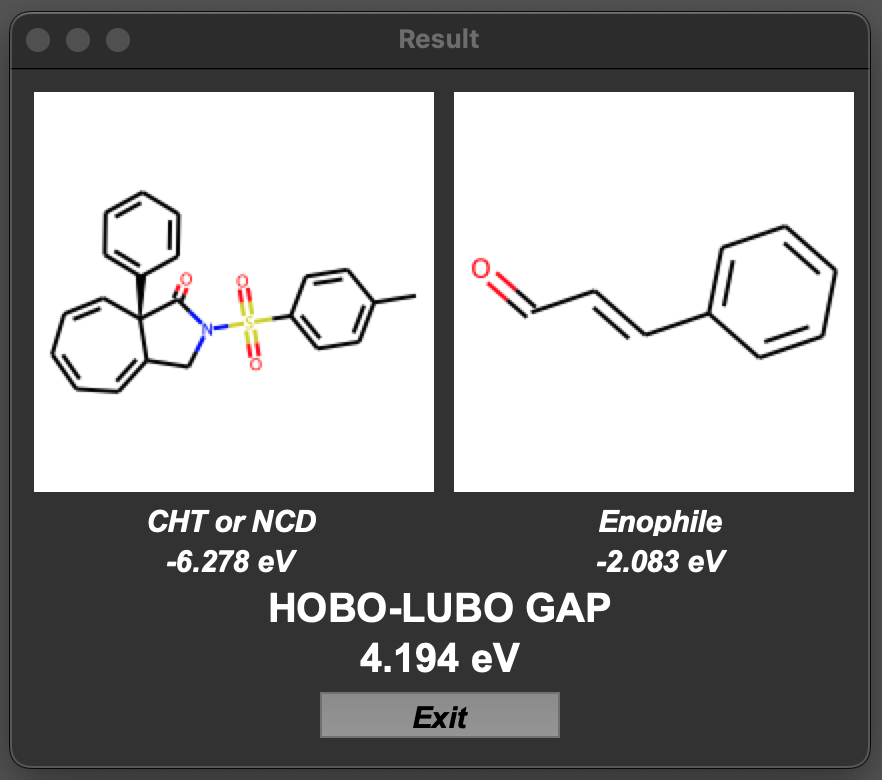

Supplement: Supplementary file 4 — Supplementary Software 1 [file 41467_2024_46523_MOESM4_ESM.zip › Supplementary Software 1/S7.png]

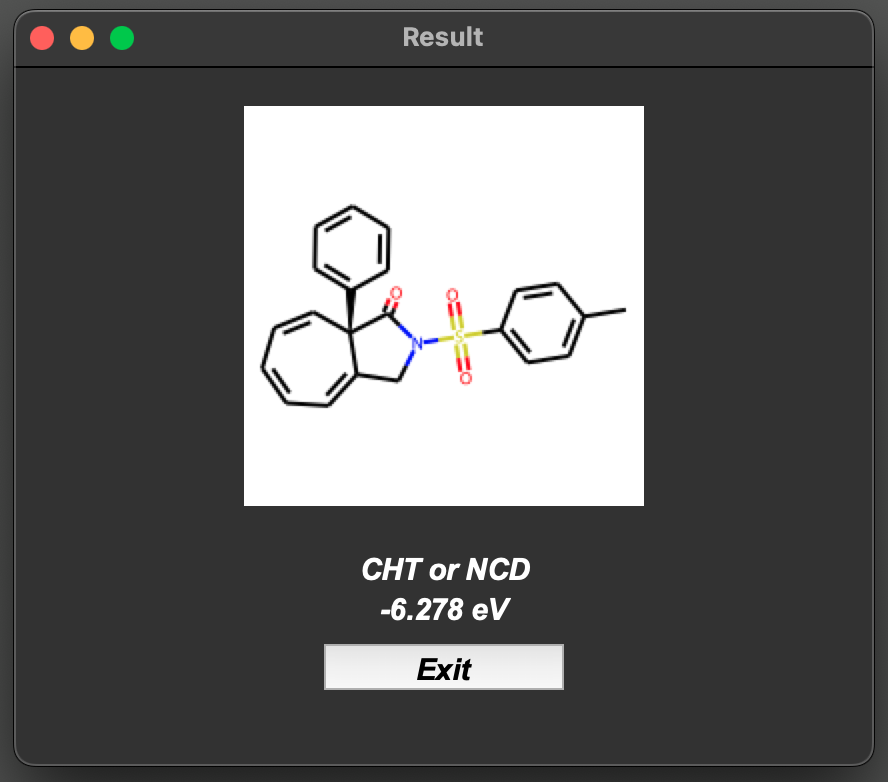

Supplement: Supplementary file 4 — Supplementary Software 1 [file 41467_2024_46523_MOESM4_ESM.zip › Supplementary Software 1/S5.png]

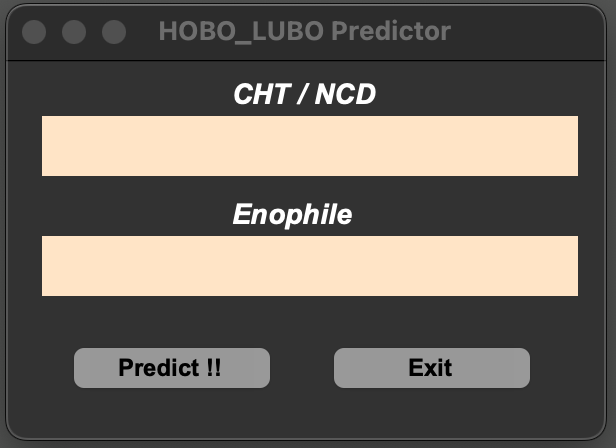

Supplement: Supplementary file 4 — Supplementary Software 1 [file 41467_2024_46523_MOESM4_ESM.zip › Supplementary Software 1/S4.png]

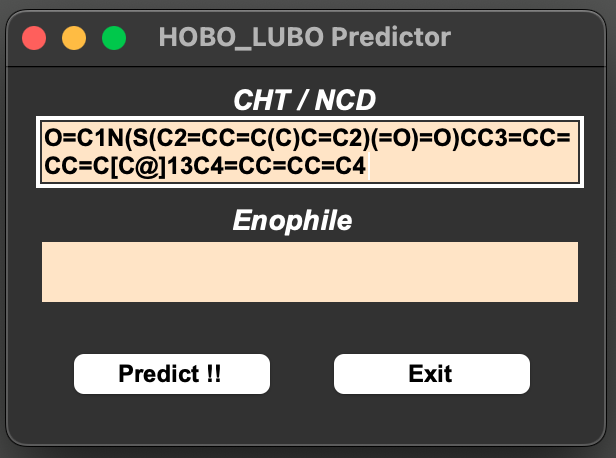

Supplement: Supplementary file 4 — Supplementary Software 1 [file 41467_2024_46523_MOESM4_ESM.zip › Supplementary Software 1/S1.png]

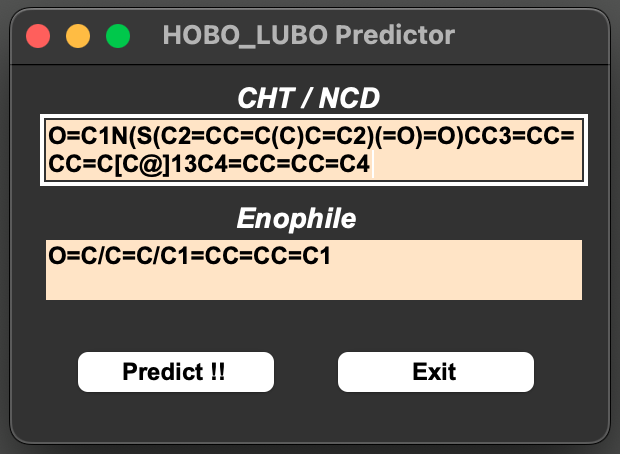

Supplement: Supplementary file 4 — Supplementary Software 1 [file 41467_2024_46523_MOESM4_ESM.zip › Supplementary Software 1/S3.png]

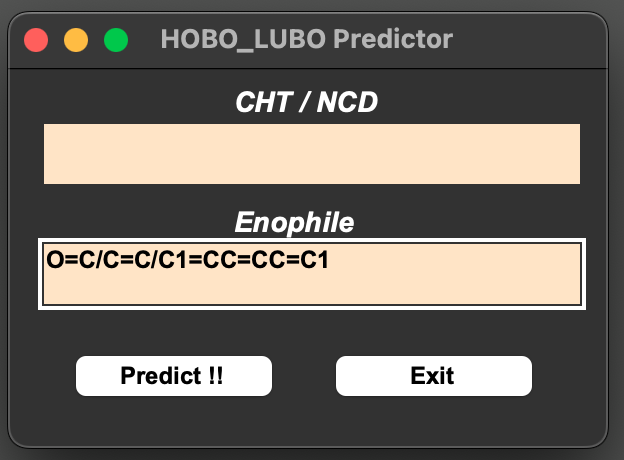

Supplement: Supplementary file 4 — Supplementary Software 1 [file 41467_2024_46523_MOESM4_ESM.zip › Supplementary Software 1/S2.png]

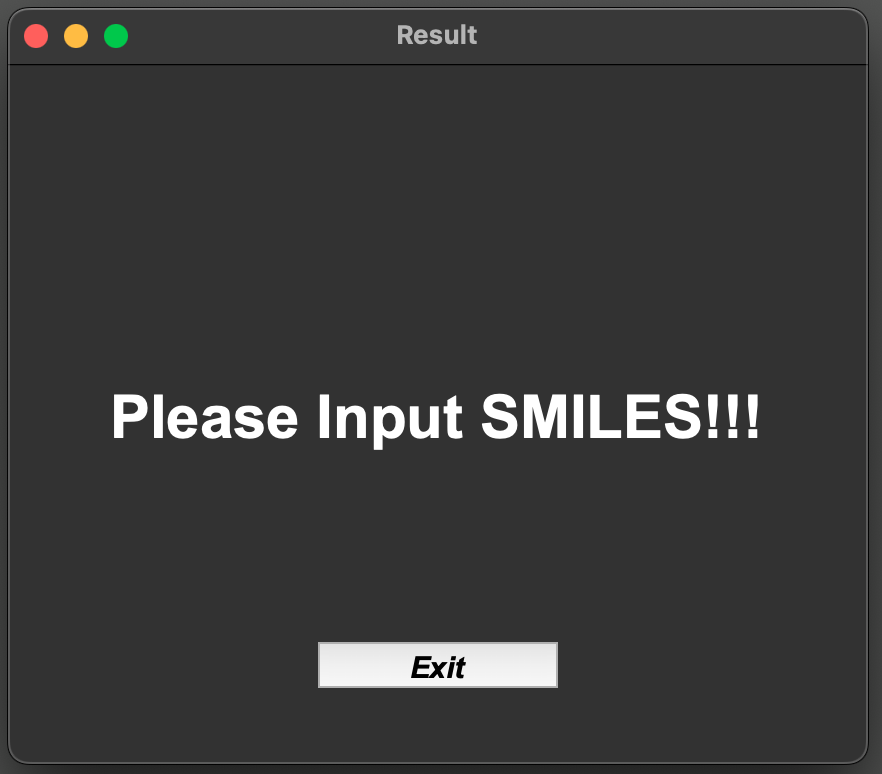

Supplement: Supplementary file 4 — Supplementary Software 1 [file 41467_2024_46523_MOESM4_ESM.zip › Supplementary Software 1/S8.png]
